# Haplotype-resolved genome analyses of a heterozygous diploid potato

**DOI:** 10.1038/s41588-020-0699-x

**Published:** 2020-09-28

**Authors:** Qian Zhou, Dié Tang, Wu Huang, Zhongmin Yang, Yu Zhang, John P. Hamilton, Richard G. F. Visser, Christian W. B. Bachem, C. Robin Buell, Zhonghua Zhang, Chunzhi Zhang, Sanwen Huang

**Affiliations:** 1 Shenzhen Branch, Guangdong Laboratory of Lingnan Modern Agriculture, Genome Analysis Laboratory of the Ministry of Agriculture and Rural Area, Agricultural Genomics Institute at Shenzhen, Chinese Academy of Agricultural Sciences, Shenzhen, China; 2 Peng Cheng Laboratory, Shenzhen, China; 3 Key Laboratory of Biology and Genetic Improvement of Horticultural Crops of the Ministry of Agriculture, Sino-Dutch Joint Laboratory of Horticultural Genomics, Institute of Vegetables and Flowers, Chinese Academy of Agricultural Sciences, Beijing, China; 4 College of Horticulture, Northwest Agriculture and Forest University, Yangling, China; 5 Department of Plant Biology, Michigan State University, East Lansing, MI USA; 6 Plant Breeding, Wageningen University and Research, Wageningen, the Netherlands; 7 College of Horticulture, Qingdao Agricultural University, Qingdao, China

**Keywords:** Genetics, Genomics, Plant breeding, Plant genetics, Sequencing

## Abstract

Potato (*Solanum tuberosum* L.) is the most important tuber crop worldwide. Efforts are underway to transform the crop from a clonally propagated tetraploid into a seed-propagated, inbred-line-based hybrid, but this process requires a better understanding of potato genome. Here, we report the 1.67-Gb haplotype-resolved assembly of a diploid potato, RH89-039-16, using a combination of multiple sequencing strategies, including circular consensus sequencing. Comparison of the two haplotypes revealed ~2.1% intragenomic diversity, including 22,134 predicted deleterious mutations in 10,642 annotated genes. In 20,583 pairs of allelic genes, 16.6% and 30.8% exhibited differential expression and methylation between alleles, respectively. Deleterious mutations and differentially expressed alleles were dispersed throughout both haplotypes, complicating strategies to eradicate deleterious alleles or stack beneficial alleles via meiotic recombination. This study offers a holistic view of the genome organization of a clonally propagated diploid species and provides insights into technological evolution in resolving complex genomes.

## Abstract (web summary)

Haplotype-resolved assembly of a heterozygous diploid potato, RH89-039-16, and identification of deleterious mutations provide insights into the genome organization and breeding of a clonally propagated diploid species.

## Main

Tetrasomic inheritance and clonal propagation via tubers are two structural challenges in *S. tuberosum* L. breeding and propagation. Genetic analyses in tetraploids are very complicated and thus genetic gains in potato breeding are limited. The widespread use of century-old varieties, such as Russet Burbank (a somatic mutant bred from a cultivar released in the 1870s in the United States) and Bintje (bred in 1904 in the Netherlands)^1^, indicates that there has been little progress in developing key traits, such as yield, quality and disease resistance in modern tetraploids.

To accelerate genetic improvement in potato, several projects have been initiated to redomesticate potato from a tuber-propagated, tetraploid crop into a seed-propagated, inbred-line-based diploid crop^2–5^. To facilitate inbred line development, an improved understanding of the genome landscape of potato clones is required. While genome heterozygosity in diploid potato has been surveyed^6–9^, these efforts were limited to bacterial artificial chromosome (BAC) clones and short-read sequences and lacked a genome-wide assessment of haplotype diversity.

Despite recent advances in genome assembly^10,11^, construction of a haplotype-resolved genome for highly heterozygous species remains a challenge^12^. Current phasing strategies rely on the alignment of sequenced reads to a reference genome to infer regional haplotypes^13–17^; such efforts are limited by the continuity of an available reference assembly. Koren et al. have developed an alternative approach, trio binning, that can recover both parental haplotypes from an F_1_ individual by partitioning parental unique reads before assembly^18^, in which case parental information is required. Recently, high-throughput/resolution chromosome conformation capture (Hi-C) technology has helped to provide allele-resolved assemblies^19,20^.

**Fig. 1:**
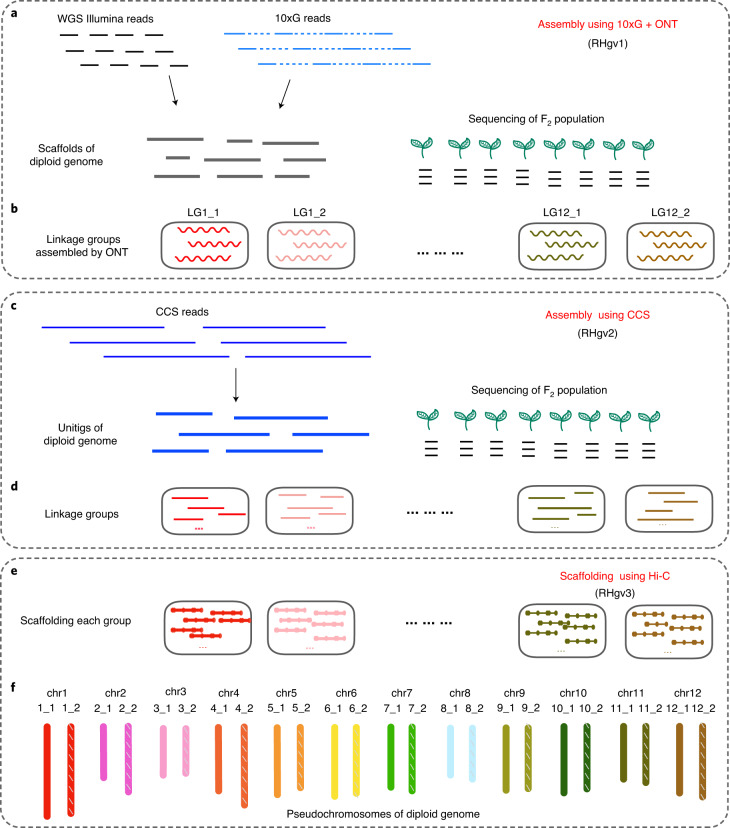
Hybrid de novo assembly and phasing of the diploid potato genome. **a**,**b**, The genome draft RHgv1 was assembled from WGS Illumina reads and 10xG linked reads, and the derived scaffolds were assigned into 24 haplotype-specific groups through genetic mapping based on a sequenced F_2_ population. The 24 groups represent chromosomes of the diploid potato (2*n* = 24). ONT reads were aligned to each linkage group and assembled to improve scaffold contiguity. **c**,**d**, A second genome sequence, RHgv2, was assembled from CCS reads. Similarly, unitigs were assigned into 24 groups through genetic mapping. **e**,**f**, The two assemblies were merged to generate a more comprehensive genome, RHgv3. Hi-C data were used to scaffold the sequences of each group into pseudochromosomes.

The heterozygous diploid potato *S. tuberosum* group Tuberosum RH89-039-16 (2*n* = 2*x* = 24, hereafter referred as to RH; Supplementary Fig. 1) has a pedigree from dihaploidized tetraploid commercial varieties, such as Katahdin, Chippewa and Primura. RH was partially assembled by the Potato Genome Sequencing Consortium (PGSC) in 2011 (refs. ^6,21^). To resolve the RH genome at the haplotype level, we sequenced it using Illumina whole-genome sequencing (WGS), 10x Genomics (10xG) linked-read sequencing, Oxford Nanopore Technologies (ONT) and Hi-C technology (Supplementary Table 1). However, our attempts to de novo assemble the two haplotypes of RH using ONT reads, and scaffolding using Hi-C reads, were unsuccessful (Supplementary Fig. 2 and Supplementary Table 2). Thus, we developed an integrated strategy to generate a haplotype-resolved assembly (Fig. 1a,b and Supplementary Fig. 3). First, the diploid genome was assembled into scaffolds using Illumina reads (WGS and 10xG data; Supplementary Table 3). Second, an RH selfing population was sequenced to provide genetic information to phase the assembled fragments. Through the genetic groupings, we assigned the scaffolds into 24 linkage groups, corresponding to the 12 chromosome pairs of RH (Supplementary Figs. 4–6). Last, for each linkage group, the ONT and 10xG reads were retrieved and reassembled to generate an improved scaffold assembly. After polishing^22,23^, the hybrid assembly yielded the genome draft version 1.0 (RHgv1) with a total length of 1.69 Gb and a scaffold N50 length of 920 kb.

Recently, accurate circular consensus sequencing (CCS) has provided impressive results on assembly and variant detection^24^, showing its potential in resolving complex genome regions. Here, we generated 29 Gb of CCS data and assembled them using CANU^25^, resulting in 1.53 Gb unitigs (contigs, split at alternate paths in the assembly graph) with an N50 size of 2.19 Mb (Supplementary Table 4). Assisted by the RH selfing population, 1.31 Gb of unitigs were assigned into 24 groups, termed version 2.0 (RHgv2; Fig. 1c,d).

We assessed the accuracy of RHgv1 and RHgv2 using previously generated paired BAC-ends (BEs) and BAC clones^6^. In total, 95% and 99% of 54,902 BEs support the sequence correctness of RHgv1 and RHgv2, respectively (Supplementary Table 5). In a total of 184 BACs previously assembled and ordered, 126 and 169 BACs mapped to a single fragment on RHgv1 and RHgv2, respectively, with 113 and 152 BACs showing perfect collinearity with the scaffolds or unitigs (Supplementary Data 1 and 2). Notably, there were 10 BACs showing structural disagreements with both RHgv1 and RHgv2, while the latter two assemblies shared consistent structure, indicating potential errors in previous BAC assembly. The evaluation of base-level accuracy assessed by aligned BAC sequences was 99.127% and 99.936% for RHgv1 and RHgv2, respectively. Taken together, RHgv2 outperforms RHgv1 on both sequence continuity and accuracy.

To ensure the completeness of the final genome assembly, RHgv1 and RHgv2 were combined, generating a new 1.67 Gb assembly with an N50 length of 1.74 Mb (RHgv3; Supplementary Table 5). After grouping by genetic map and anchoring by the Hi-C data, 1.62 Gb of sequence constituted 24 pseudochromosomes, exceeding current assemblies of potato genomes^6,7,9^ (Fig. 1e,f, Supplementary Fig. 7, Supplementary Tables 6–9 and Supplementary Data 3). Hereafter, all analyses were performed on RHgv3.

The phasing quality of the haplotype-resolved assembly was assessed on pseudochromosomes of RHgv3. The BEs were realigned to chromosomes and 46,058 (95.1%) of 48,410 aligned BEs were in same phase. A total of 1,639 BACs with unordered contigs were used to assess haplotype partitioning. Among 1,624 BACs that mapped with at least 60% of BAC length, 1,573 BACs (96.8%) aligned to a single phase of the RH assembly. Previously, Boer et al. reported a phased assembly of RH chromosome 5 based on BAC-by-BAC sequencing^21^. The alignment of 26.7-Mb haplotype RH{0} BAC minimal tiling paths (MTPs) and 25.0-Mb haplotype RH{1} MTPs with the pseudochromosome chr5_1 and chr5_2 showed that 26.0-Mb (97%) RH{0} MTPs have the best hit on chromosome 5_1 and 23.9-Mb (96%) RH{1} MTPs have the best hit on chromosome 5_2. Collectively, these analyses demonstrated that the accuracy of haplotype determination is more than 95% at the chromosome level.

A total of 76,394 protein-coding genes were annotated in the RH genome, and evaluation with BUSCO^26^ genes revealed that 97.0% (1,398) of 1,440 examined genes were complete, with 74.1% (1,306) duplicated. Comparative analyses among the gene models of RH, M6 (a diploid potato with an assembled genome^9^) and DM (the potato reference genome) identified 18,377, 3,842 and 10,742 lineage-specific genes in the three genomes, respectively, constituting 24.1%, 10.2% and 27.5% of their annotated genes, respectively (Supplementary Fig. 8). For example, the dominant tuber shape gene *Ro*, which was reported absent in the DM clone^27^, has two homozygous copies (*RHC10H1G1859.2* and *RHC10H2G2643.2*) on two RH haplotypes and has one copy (*g7634.t1*) on M6.

**Fig. 2:**
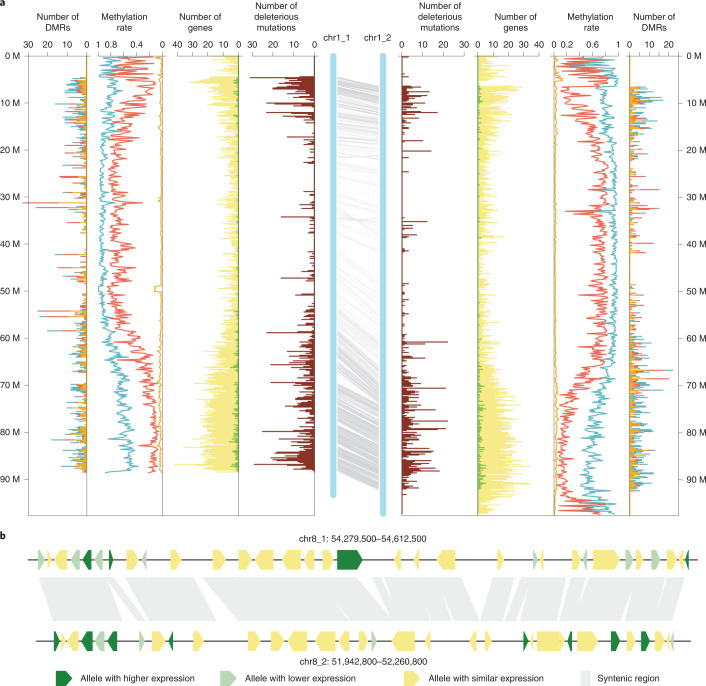
Haplotype divergence in a diploid potato genome. **a**, The central blue bars represent the two haplotypes of chromosome 1. The gray lines indicate paired allelic genes. Distribution of deleterious or dysfunctional mutations (brown), annotated genes (yellow), preferentially expressed alleles (green), methylation level of three contexts and differentially methylated regions are arranged symmetrically for each haplotype. Methylation level and the number of DMRs of methylated sites in CG (light blue), CHG (red) and CHH (orange) contexts are indicated by cumulative column chart. The number of DMRs on one haplotype only involves the DMRs with hypermethylation. All numbers were determined in 200-kb windows. **b**, Zoomed-in view of a syntenic block showing the mosaic pattern of preferentially expressed alleles (dark green) and alleles with lower expression (light green) on the two haplotypes. Source data

**Extended Data Fig. 1:**
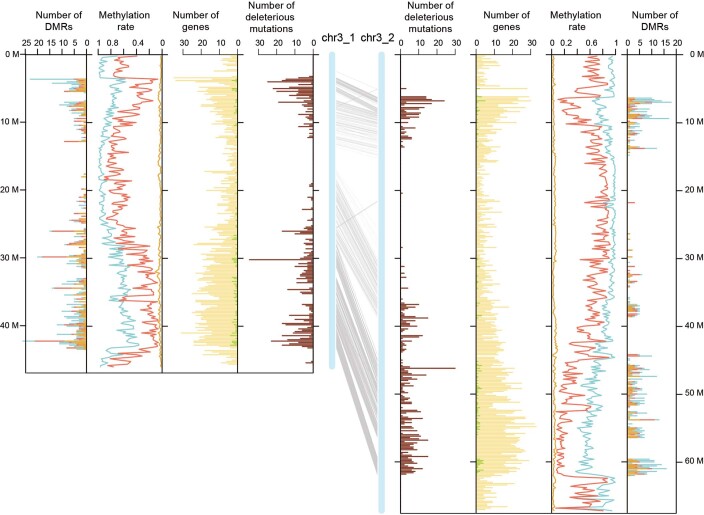
Haplotype alignment of RH chromosome 3. The central blue bars represent the two haplotypes of chromosome 3 with the gray lines indicating the paired allelic genes. The distribution of deleterious or dysfunctional mutations (brown), annotated genes (yellow), preferentially expressed alleles (green), methylation level of three contexts and differentially methylated regions are arranged symmetrically for each haplotype. The methylation level and the number of DMRs of methylated sites in CG (light blue), CHG (red) and CHH (orange) contexts are indicated by cumulative column chart. Number of DMRs on one haplotype only involves the DMRs with hyper-methylation. All of the numbers were determined in 200 kb windows.

**Extended Data Fig. 2:**
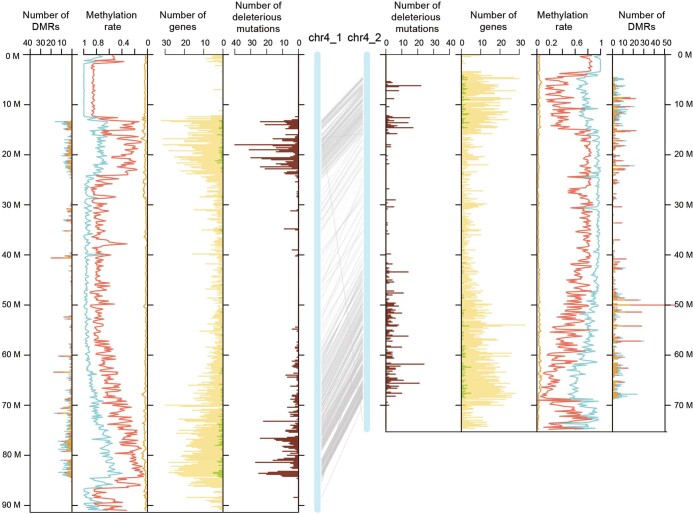
Haplotype alignment of RH chromosome 4. The central blue bars represent the two haplotypes of chromosome 4 with the gray lines indicating the paired allelic genes. The distribution of deleterious or dysfunctional mutations (brown), annotated genes (yellow), preferentially expressed alleles (green), methylation level of three contexts and differentially methylated regions are arranged symmetrically for each haplotype. The methylation level and the number of DMRs of methylated sites in CG (light blue), CHG (red) and CHH (orange) contexts are indicated by cumulative column chart. Number of DMRs on one haplotype only involves the DMRs with hyper-methylation. All of the numbers were determined in 200 kb windows.

**Extended Data Fig. 3:**
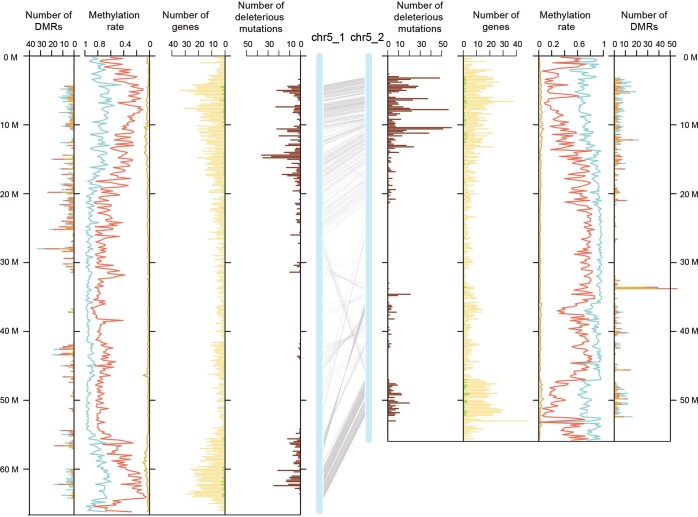
Haplotype alignment of RH chromosome 5. The central blue bars represent the two haplotypes of chromosome 5 with the gray lines indicating the paired allelic genes. The distribution of deleterious or dysfunctional mutations (brown), annotated genes (yellow), preferentially expressed alleles (green), methylation level of three contexts and differentially methylated regions are arranged symmetrically for each haplotype. The methylation level and the number of DMRs of methylated sites in CG (light blue), CHG (red) and CHH (orange) contexts are indicated by cumulative column chart. Number of DMRs on one haplotype only involves the DMRs with hyper-methylation. All of the numbers were determined in 200 kb windows.

**Extended Data Fig. 4:**
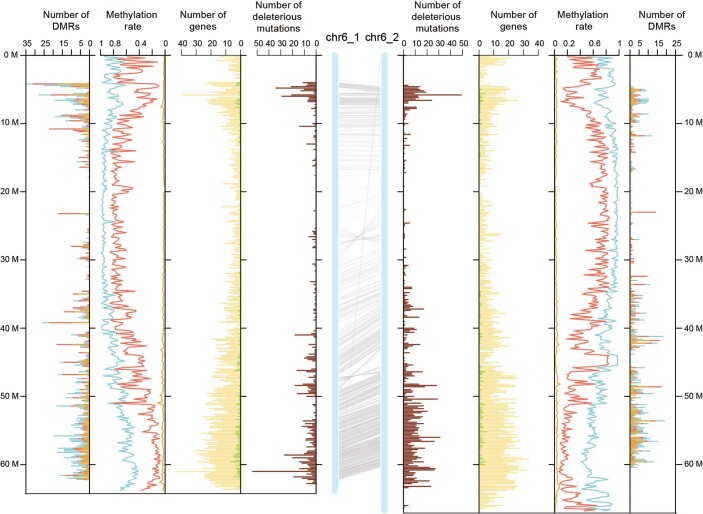
Haplotype alignment of RH chromosome 6. The central blue bars represent the two haplotypes of chromosome 6 with the gray lines indicating the paired allelic genes. The distribution of deleterious or dysfunctional mutations (brown), annotated genes (yellow), preferentially expressed alleles (green), methylation level of three contexts and differentially methylated regions are arranged symmetrically for each haplotype. The methylation level and the number of DMRs of methylated sites in CG (light blue), CHG (red) and CHH (orange) contexts are indicated by cumulative column chart. Number of DMRs on one haplotype only involves the DMRs with hyper-methylation. All of the numbers were determined in 200 kb windows.

**Extended Data Fig. 5:**
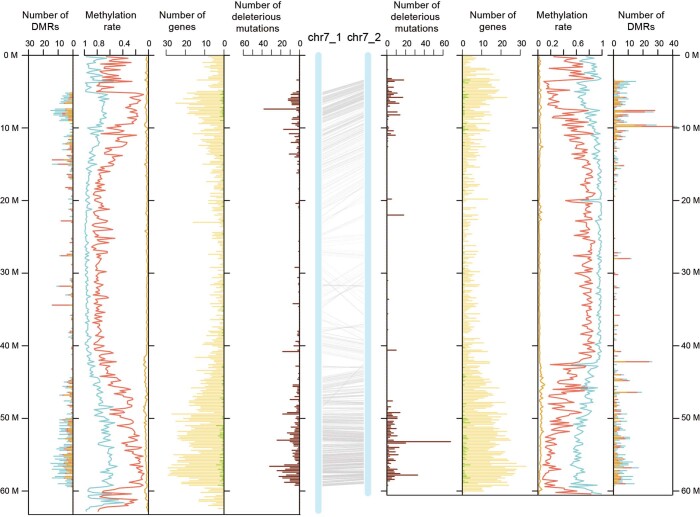
Haplotype alignment of RH chromosome 7. The central blue bars represent the two haplotypes of chromosome 7 with the gray lines indicating the paired allelic genes. The distribution of deleterious or dysfunctional mutations (brown), annotated genes (yellow), preferentially expressed alleles (green), methylation level of three contexts and differentially methylated regions are arranged symmetrically for each haplotype. The methylation level and the number of DMRs of methylated sites in CG (light blue), CHG (red) and CHH (orange) contexts are indicated by cumulative column chart. Number of DMRs on one haplotype only involves the DMRs with hyper-methylation. All of the numbers were determined in 200 kb windows.

**Extended Data Fig. 6:**
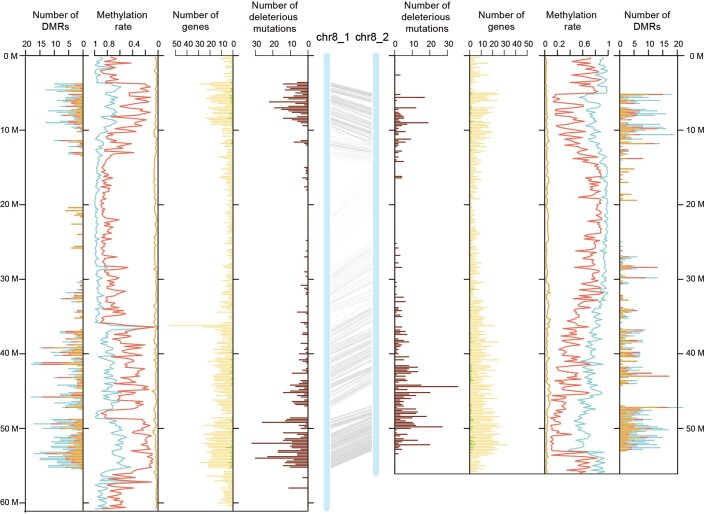
Haplotype alignment of RH chromosome 8. The central blue bars represent the two haplotypes of chromosome 8 with the gray lines indicating the paired allelic genes. The distribution of deleterious or dysfunctional mutations (brown), annotated genes (yellow), preferentially expressed alleles (green), methylation level of three contexts and differentially methylated regions are arranged symmetrically for each haplotype. The methylation level and the number of DMRs of methylated sites in CG (light blue), CHG (red) and CHH (orange) contexts are indicated by cumulative column chart. Number of DMRs on one haplotype only involves the DMRs with hyper-methylation. All of the numbers were determined in 200 kb windows.

**Extended Data Fig. 7:**
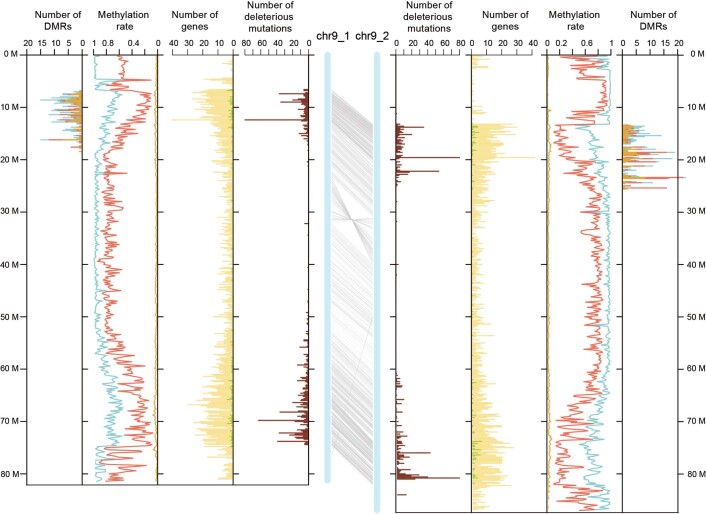
Haplotype alignment of RH chromosome 9. The central blue bars represent the two haplotypes of chromosome 9 with the gray lines indicating the paired allelic genes. The distribution of deleterious or dysfunctional mutations (brown), annotated genes (yellow), preferentially expressed alleles (green), methylation level of three contexts and differentially methylated regions are arranged symmetrically for each haplotype. The methylation level and the number of DMRs of methylated sites in CG (light blue), CHG (red) and CHH (orange) contexts are indicated by cumulative column chart. Number of DMRs on one haplotype only involves the DMRs with hyper-methylation. All of the numbers were determined in 200 kb windows.

**Extended Data Fig. 8:**
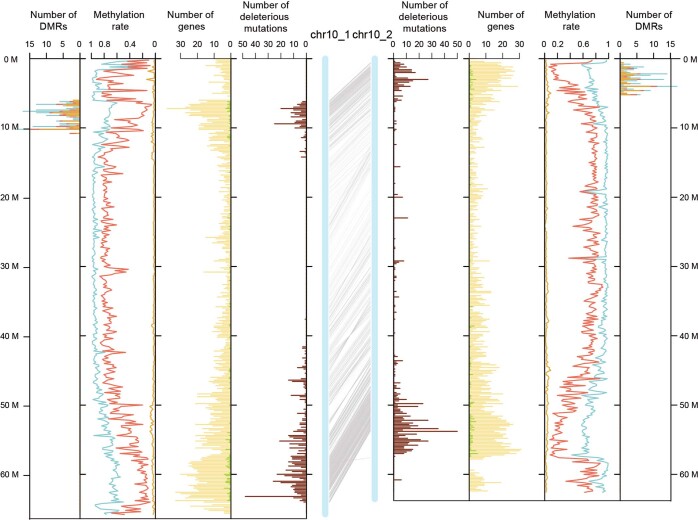
Haplotype alignment of RH chromosome 10. The central blue bars represent the two haplotypes of chromosome 10 with the gray lines indicating the paired allelic genes. The distribution of deleterious or dysfunctional mutations (brown), annotated genes (yellow), preferentially expressed alleles (green), methylation level of three contexts and differentially methylated regions are arranged symmetrically for each haplotype. The methylation level and the number of DMRs of methylated sites in CG (light blue), CHG (red) and CHH (orange) contexts are indicated by cumulative column chart. Number of DMRs on one haplotype only involves the DMRs with hyper-methylation. All of the numbers were determined in 200 kb windows.

**Extended Data Fig. 9:**
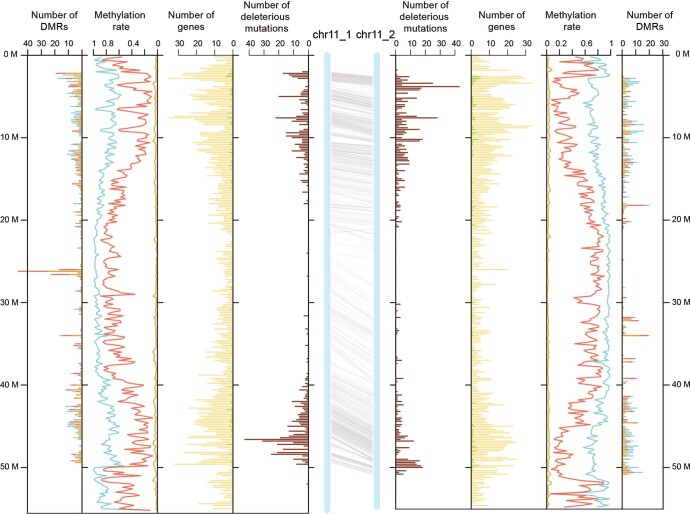
Haplotype alignment of RH chromosome 11. The central blue bars represent the two haplotypes of chromosome 11 with the gray lines indicating the paired allelic genes. The distribution of deleterious or dysfunctional mutations (brown), annotated genes (yellow), preferentially expressed alleles (green), methylation level of three contexts and differentially methylated regions are arranged symmetrically for each haplotype. The methylation level and the number of DMRs of methylated sites in CG (light blue), CHG (red) and CHH (orange) contexts are indicated by cumulative column chart. Number of DMRs on one haplotype only involves the DMRs with hyper-methylation. All of the numbers were determined in 200 kb windows.

**Extended Data Fig. 10:**
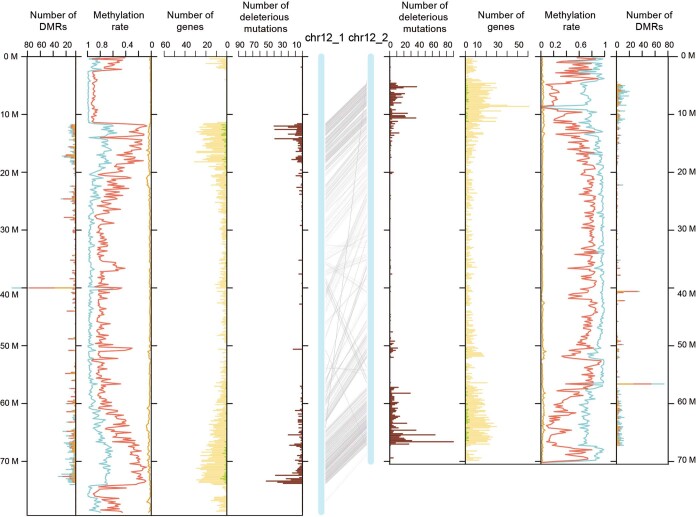
Haplotype alignment of RH chromosome 12. The central blue bars represent the two haplotypes of chromosome 12 with the gray lines indicating the paired allelic genes. The distribution of deleterious or dysfunctional mutations (brown), annotated genes (yellow), preferentially expressed alleles (green), methylation level of three contexts and differentially methylated regions are arranged symmetrically for each haplotype. The methylation level and the number of DMRs of methylated sites in CG (light blue), CHG (red) and CHH (orange) contexts are indicated by cumulative column chart. Number of DMRs on one haplotype only involves the DMRs with hyper-methylation. All of the numbers were determined in 200 kb windows.

To provide an accurate evaluation of the divergence between the two RH haplotypes, we identified polymorphisms between the 12 homologous chromosome pairs (Fig. 2a, Supplementary Figs. 9 and 10 and Extended Data Figs. 1–10). Based on the alignment of genes on the two haplotypes^28^, 198 syntenic blocks were detected, covering 1.3 Gb (80.2%) of anchored sequence (Supplementary Table 10). Between syntenic blocks, 12,299,445 SNPs, 1,393,680 indels (~1–50 bp), 38,999 structural variants (SVs, >50 bp) and 1,878 genes showing presence and absence variation (PAV) were identified^29,30^, including 106 large SVs spanning more than 100 kb. Overall, the intragenomic diversity was estimated at ~2.1%, a level higher than the diversity among out-crossing maize lines^31^. Based on synteny and annotation, 59,907 genes (78.4% of all annotated genes) were identified as having homologs on the two haplotypes, and 20,583 pairs (41,166 genes) of those were considered as reliable allelic genes. Among them, alleles of 17,092 gene pairs showed variants within the coding sequence (CDS), including amino acid alternation and premature termination^32^. Based on amino acid conservation modeling^33,34^, 4,761 and 1,753 pairs of allelic genes were predicted to have potential deleterious substitutions in one and both alleles, respectively, indicating substantial accumulation of mutations in this clonally propagated crop^35^ (Supplementary Table 11).

To understand the expression landscape of allelic genes, RNA-sequencing (RNA-seq) data of ten tissues were analyzed using Kallisto pipeline^36,37^. Overall, 48,361 genes (63.3% of total) expressed in at least one tissue and 3,417 gene pairs (16.6% of allelic genes) exhibited unequal expression between two alleles, termed differentially expressed loci (DEL). The DEL were distributed randomly throughout the genome with higher and lower expressed alleles occurring alternatively on the two haplotypes (Fig. 2b and Supplementary Table 12). From methylation sequencing of mature leaves, immature small tubers (transection diameter <1 cm) and immature large tubers (transection diameter ~1–5 cm), on average, 24,929 differential methylated regions (DMRs) were identified between paired syntenic regions, resulting in allelic methylation differences in 6,345 gene pairs (30.8% of allelic genes; Supplementary Fig. 11)^38^. By comparing the DMRs with the DEL, we found the methylation difference explained only a fraction of the expression difference of alleles. For example, in immature small tuber tissue, only 292 DEL (27.5% of all DEL) showed both differential expression and methylation (Supplementary Fig. 12).

**Fig. 3:**
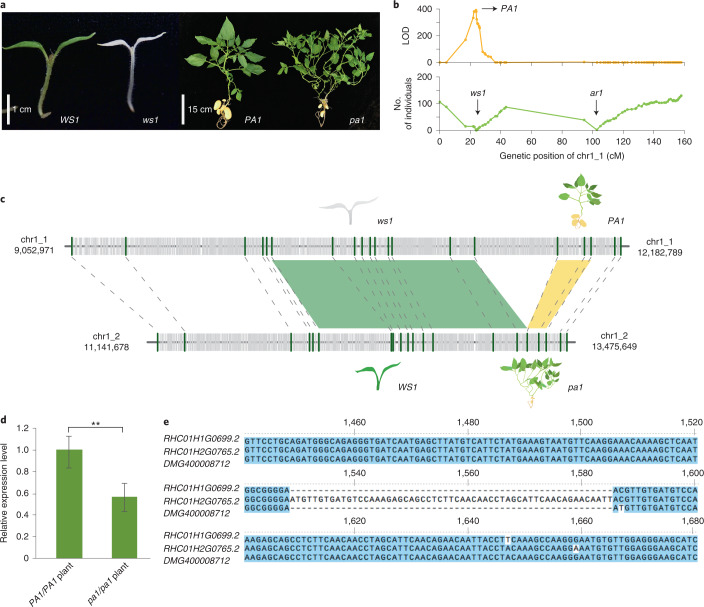
Tight linkage of two deleterious genes in the repulsion phase. **a**, Phenotype of normal seedling (*WS1*) and white seedling (*ws1*), normal plant architecture (*PA1*) and more branched architecture (*pa1*). **b**, Genetic mapping of *PA1* and *ws1* on chromosome 1_1. The top graph shows the likelihood of odd (LOD) value of *PA1* mapping using R/qtl software^41^, and the bottom graph represents the number of individuals with a homozygous recessive allele. Dots in the graphs present the genetic markers. The *ar1* locus has been reported previously^39^. **c**, Fine mapping of *ws1* and *pa1* using indel markers (green bars). Gray segments represent the repeat elements; green and yellow blocks indicate the positions of *WS1/ws1* and *PA1/pa1*, respectively. **d**, Quantitative PCR (qPCR) result of *RHC01H1G0699.2* in normal (*PA1*/*PA1*) and more-branched (*pa1*/*pa1*) plants. Error bars represent the standard deviation from four biological replicates, and asterisks indicate significant differences between normal and more-branched plants (*t*-test, **P value < 0.01). **e**, CDS alignment of *RHC01H1G0699.2*, *RHC01H2G0765.2* and their homolog in DM (*DMG400008712*), showing the 57-bp indel between alleles. Source data

Through the analysis of an RH selfing population, 25.7% of genomic regions (430.8 Mb) exhibited strong segregation distortion (SD; *χ*^2^ test, *P* < 0.001; Supplementary Fig. 13). Large-effect recessive deleterious mutations are the main cause of zygotic selection, which caused 71.4% of the SD regions. Using the selfed progeny of RH, we identified several loci affecting survival (white seedling 1 (*ws1*), abnormal rooting 1 (*ar1*), lethal allele 2 (*la2*)) or growth vigor (plant architecture 1 (*pa1*), plant architecture 2 (*pa2*) and weak vigor 1 (*wv1*)). Except for *wv1* (Supplementary Fig. 14), the other five loci have been previously reported^39^. Here, we relocated these loci on the phased RH genome, clarifying which haplotype contained the dominant or recessive allele (Fig. 3a,b and Supplementary Fig. 15)^40,41^. All six loci were located in the SD regions (Supplementary Table 13). Generally, large-effect deleterious mutations are relatively dispersed in the genome, which could be removed by sexual selection.

However, two recessive detrimental alleles, *ws1* and *pa1*, are tightly linked but in repulsion on the short arm of chromosome 1. Phenotype-based selection will not be sufficient to break the linkage of these two alleles, as demonstrated in practical breeding efforts; therefore, additional genetic analysis of this locus is required. In the mapping of *pa1* and *ws1*, using 880 F_2_ progeny, the two loci remain linked (Fig. 3b). Genotyping of an additional 1,200 F_2_ individuals identified two recombinant plants, which delimited *ws1* and *pa1* into two adjacent regions. The *WS1* locus was mapped to a 1.19 Mb region (chr1_2: 12,061,229–13,249,054) that contained 76 annotated genes, including six DEL and 30 allelic genes harboring exonic variants. The *PA1* locus was mapped to a 191-kb region (chr1_1: 11,821,758–12,012,928) containing 11 genes (Fig. 3c). Among them, one gene, *RHC01H1G0699.2*, encoding the ETHYLENE INSENSITIVE3 (EIN3) protein was identified as a candidate. *AtEIN3* has been reported to regulate plant growth in *Arabidopsis thaliana*^42^ by acting as a transcriptional regulator in the ethylene signaling pathway. *RHC01H1G0699.2* showed decreased expression in more-branched plants (*pa1*/*pa1*) than in normal plants (*PA1*/*PA1*; Fig. 3d), and this expression pattern was consistent with observations on the *A. thaliana ein3* mutant. Between the dominant allele *RHC01H1G0699.2* and the recessive allele *RHC01H2G0765.2*, there was a 57-bp insertion that might result in additional translation of 19 amino acids in RHC01H2G0765.2 (Fig. 3e and Supplementary Figs. 16 and 17). Genome-assisted analyses of these two large-effect deleterious alleles provide tools to break the tight linkage in the repulsive phase for a better inbred line from RH.

In the current study, we combined multiple sequencing technologies to achieve the de novo assembly and haplotype determination of the heterozygous diploid potato. Compared with short reads or the longer but more error-prone ONT reads, CCS reads generated higher resolution and accuracy in differentiating haplotypes, which is particularly useful in resolving complex genomes. However, for a complex genome like diploid potato, there is still no tool that can build near-complete haplotypes from long-read sequencing or Hi-C sequencing without the assistance of genetic information, which requires further improvement in assembly algorithms.

The haplotype-resolved genome of the diploid potato provides a holistic view of the genome organization of a clonally propagated, heterozygous plant species. Haplotype-resolved identification of deleterious mutations, especially tightly linked genes in repulsion, provides insights into purging mutation burden by efficient molecular selection and/or genome-editing technologies^43^. As such, this study could facilitate the exploitation of heterosis, using inbred lines with complementary haplotypes, which is the core of diploid potato breeding.

## Methods

### Genome, transcriptome and methylome sequencing

To construct an Illumina sequencing library, genomic DNA was extracted from RH leaves by using the cetyltrimethylammonium bromide (CTAB) method^44^. The library was sequenced on the Illumina HiSeq 2500 platform, generating 155 Gb of 250-nucleotide paired-end reads with an insert size of ~400 bp.

About 1.2 ng high molecular weight DNA (>50 kb) was isolated and loaded for 10xG library construction, following the manufacturer’s recommended protocols (https://support.10xgenomics.com/de-novo-assembly/library-prep/doc/user-guide-chromium-genome-reagent-kit-v1-chemistry/). The 10xG library was sequenced on the Illumina HiSeq X Ten platform, yielding 122 Gb of 150-nucleotide paired-end data.

In construction of the ONT library, an optimized protocol for long plant DNA enrichment was applied^45^. The library was constructed using LSK108 kit (SQK-LSK108, Oxford) and sequenced using 38 R9.4 flow cells on the Nanopore GridION X5 sequencer. The base calling was performed using Albacore in MinKNOW package, and 10.7 million nanopore reads with an N50 length of 25.3 kb were available for assembly.

For CCS, genomic DNA was extracted from in vitro seedlings using the DNeasy Plant Mini Kit (Qiagen). The integrity of the DNA was determined with the Agilent 4200 Bioanalyzer (Agilent Technologies). Genomic DNA (15 μg) was sheared using g-Tubes (Covaris) and concentrated with AMPure PB magnetic beads. Two SMRTbell libraries were constructed using the Pacific Biosciences SMRTbell Template Prep Kit 1.0. The libraries were size selected on a BluePippin system for molecules with a size of 11 Kb, followed by primer annealing and the binding of SMRTbell templates to polymerases with the DNA/Polymerase Binding Kit. Libraries sequencing was carried out on the Pacific Bioscience Sequel II platform (Annoroad Gene Technology) and 29-Gb CCS reads with an N50 size of 13 kb were generated using ccs software v.3.0.0 (https://github.com/pacificbiosciences/unanimity/).

The Hi-C libraries were constructed at Annoroad Gene Technology using the in situ method^46^. DNA from in vitro seedlings of RH was digested with MboI using the standard Hi-C library preparation protocol. The Hi-C libraries were sequenced on an Illumina HiSeq X Ten platform, yielding 150 Gb of data.

The selfing population (S_1_ population, equivalent to an F_2_ population) of RH was constructed by forced self-pollination^39^ and 880 F_2_ individuals were sequenced at ~1× depth using an Illumina HiSeq X Ten platform. On average, ~2 Gb of data were obtained from each individual.

Samples from the young leaf, mature leaf, stem, perianth, anther, carpel, stolon, immature small tuber (transection diameter of <1 cm), immature big tuber (transection diameter of 1–5 cm) and root tissue were collected for transcriptome sequencing. All tissues were isolated and sequenced in three biological replicates. Total RNA was extracted from the samples using the TIANGEN Kit with DNase I and processed for the library construction using NEBNext UltraTM RNA Library Prep Kit. Following the removal of low-quality data, ~3 Gb of 150-nucleotide paired-end data for each sample were used for further RNA-seq analysis.

In addition to the transcriptome sequencing, samples from three tissues—mature leaf, immature small tuber (transection diameter <1 cm) and immature large tuber (transection diameter of 1–5 cm)—were used for whole-genome bisulfite sequencing with three biological replicates. Genomic DNA was extracted using the CTAB method and fragmented to ~200–300 bp by sonication before library construction. The barcoded DNA was treated twice with bisulfite using the EZ DNA Methylation-Gold Kit (Zymo Research) according to the manufacturer’s instructions. The libraries were sequenced on a HiSeq X Ten platform and 50 Gb of 150-nucleotide paired-end data were generated.

### Hierarchical assembly and phasing of diploid potato genome using 10xG and ONT data

#### Whole-genome de novo assembly

The WGS Illumina reads were assembled using DISCOVAR de novo (https://software.broadinstitute.org/software/discovar/blog/), resulting in 1.3-Gb sequences with a scaffold N50 size of 14.9 kb. The 10xG reads were assembled using Supernova (https://github.com/10XGenomics/supernova/) with the ‘megabubbles’ output; 1.58 Gb of assembled sequence data were generated.

#### Genetic genotyping and grouping

In the pipeline, the 880 F_2_ selfing progeny were sequenced at ~1×–2× coverage for genetic mapping (Supplementary Fig. 5). First, for each progeny, the sequenced reads were mapped to the assembled RH scaffolds using BWA-MEM^47^. For each scaffold, the raw number of mapped reads with mapping quality >50 was normalized according to the scaffold length, the total assembled length and the total mapped reads of this progeny. Then the normalized read number for all scaffolds was transformed to genotype scores. The genetic groups were built using the software JoinMap (v4.0)^48^. A total of 1,408 Mb sequences were genotyped and grouped into 12 linkage groups, corresponding to the 12 chromosomes of monoploid potato.

For each linkage group, we applied the R function *hclust* (method = ‘ward.D2’, *k* = 2) to separate each group into two clusters, corresponding to the two haplotypes of the diploid potato. To assign the residual 310 Mb of scaffolds, which displayed obscure read distribution and failed in genotype calling, we calculated the correlation between grouped scaffolds (target) and residual scaffolds (query) on the number of mapped reads using the *cor* function in R (Supplementary Fig. 5). If the query scaffold and the target scaffold shared a similar pattern (correlation > 0.7) on read distribution in the population, they were deemed to belong to same linkage group. For each query scaffold, we determined its group using two criteria: (1) the top two correlation values with target scaffolds should be larger than 0.7 and (2) the top two target scaffolds showing the highest correlation values should be located on the same group. After this process, 117.8 Mb of residual scaffolds were assigned to 24 linkage groups. In total, 1.52 Gb of 1.7 Gb sequences were grouped into 24 clusters, accounting for 90% of the assembled genome.

#### Simplified reassembly within each group

One effective way to simplify the assembly for a complex genome is to dilute the genome into multiple parts and separately assemble each part. In this project, we leveraged a similar simplification by separately reassembling the 24 clusters. First, the ONT long reads were mapped to the scaffolds using minimap2 (ref. ^49^). Second, the reads belonging to each genetic group were retrieved and assembled into contigs using SMARTdenovo (https://github.com/ruanjue/smartdenovo/). Only reads with properly paired mapping and less than two mismatched bases reads were collected for the reassembly. Third, the contigs were polished iteratively using Racon^22^ and Pilon^23^. Last, the 10xG reads were aligned to the contigs using Long Ranger (https://support.10xgenomics.com/genome-exome/software/downloads/latest) to generate scaffolds using the ARCS + LINKS pipeline^50^, which increased the assembly continuity from contig N50 length of 636 kb to 921 kb. The hybrid assembly yielded the genome draft RHgv1.

### Genome assembly and phasing using PacBio CCS reads

A total of 29 Gb CCS reads were assembled using Canu (v1.91)^25^ with the parameter --pacbio-hifi. Canu generated two assemblies composed of contigs and unitigs (Supplementary Table 4), and the unitig assembly consisted of the contigs that split at any alternative paths in the assembly graph. The contig assembly had longer continuity but more chimeric fragments as revealed in the genetic mapping analysis. To avoid the mis-joining of two haplotypes, the unitig assembly rather than the contig assembly was chosen for the subsequent analysis. The unitigs were then polished iteratively using two rounds of Pilon^23^ with ~150 Gb of WGS Illumina data, generating the genome draft RHgv2.

Similarly, the sequenced reads of RH selfing progeny were mapped to unitigs of RHgv2 to perform genetic grouping. Because the unitigs were relatively long (N50 = 2 Mb), windows with a size of 200 kb rather than the whole unitig were used. If the adjacent windows of one unitig showed contrary read distribution, the unitig was defined as chimeric and broken between windows; 40 chimeric unitigs with a total length of 95 Mb were broken. In total, 1.31 Gb of 1.53 Gb sequences were assigned to 24 linkage groups.

After merging, 141 Mb sequences and 5,252 annotated genes of RHgv1 were added to the RHgv2, yielding a 1.67 Gb genome draft with 1.54 Gb sequences assigned to 24 groups, termed RHgv3 (Supplementary Table 6). The sequences from the RHgv1 and RHgv2 assemblies were named as ontctg* and unitig* in the AGP file, respectively (Supplementary Data 3).

### Construction of pseudochromosomes

As no approach generated satisfactory results on the RH genome, we introduced the group information derived from the genetic mapping to assist the Hi-C application on chromosome-level assembly. The process was performed on RHgv3 including three steps as follows:

1. Align. The 24 previously determined groups were divided into two haplotypes to generate two pseudohaploid genome drafts. The 132 Mb sequences that could not be assigned to any group were added to two pseudohaploid genomes. Total Hi-C reads were aligned to each pseudohaploid genome using HiC-Pro^51^ to calculate the contact frequency. This step yielded two bam files for the two pseudohaploid genomes.
2. Rescue. Using the bam file as input, the *rescue* function in ALLHiC^20^ was applied to assign unplaced sequences to known groups. Because the 132 Mb unplaced sequences were added to two pseudohaploid genomes and processed twice, the rescued results were redundant. For every unplaced sequence, we considered its best Hi-C signal density to decide the group to which it belonged. After this step, the sequence content of 24 groups was updated with an extra 75.6 Mb sequences assigned to proper groups.
3. Optimize and build. For each pseudohaploid genome, using the bam file and the updated group file as input, the *optimize* function in ALLHiC decided the order and orientation of scaffolds for each group; thus, the *build* function generated fasta sequences on that basis. By performing this step, we identified the pseudochromosomes for 24 groups. The order and orientation of scaffolds on chromosomes are provided in Supplementary Data 3.

### Genome assembly assessment

The BAC clones and BEs of RH were downloaded from http://solanaceae.plantbiology.msu.edu/pgsc_download.shtml (ref. ^6^) to assess the assembly.

#### Assess scaffold assembly

Using Sanger technology, the 54,902 paired BEs were sequenced with the average length of 714 nucleotides^6^. The BEs were aligned to the assembled scaffolds using BLASR (v1.3.1)^52^, and those aligned with >98% query coverage and >98% identity were considered as the successful alignments. A reasonable distance between end sequences (set to ~30–300 kb) and correct orientation (± for each of them), when mapped on to the genome, were used as criteria for assessing the correctness of the assemblies.

The 184 BACs that assembled with ordered contigs and a total length of 21,734,426 bp were aligned to RH scaffolds using MUMmer (v3.23)^29^. The alignment was filtered using the cutoff criteria: identity >98% and alignment length >2 kb. Some BACs completely aligned with single scaffolds, while others were fragmented or repeatedly mapped to multiple scaffolds. To evaluate the correctness of scaffolds or contigs, only the BACs that mapped to single scaffolds of genome assembly were used in the statistics. The mapping structure between BACs and scaffolds was manually checked to determine if the alignments were complete and collinear (Supplementary Data 1 and 2). SNPs and indels were identified from the alignment between BACs and scaffolds using the *show-snps* function in MUMmer.

#### Assess phasing quality

The BE sequences and 1,639 RH BACs with a total length of 205 Mb and an average size of 125 kb were aligned to the pseudochromosomes of RHgv3 using BLASR (v5.1) to assess the haplotypes. Because most of the BACs contained only unordered contigs, we considered the alignment length and identity when selecting the best hits for BACs. Only alignments with a mapQV of >50 and identity of >95% were retained for downstream analyses.

A total of 55.4 Mb nonredundant BAC MTPs with an N50 length of 336 kb were extracted from the assembly of the previously published RH chromosome 5 (ref. ^21^). MTPs were aligned to the RH chromosomes using MUMmer (v3.23)^29^ and filtered using the criteria of identity >95% and alignment length >2 kb. For each MTP, only the best hit was considered to compare the phases.

### Genome annotation

Repeat-sequence masking was performed using RepeatMasker (v4.0.6) with default parameters. The reference repeat libraries included plant short fragment repeats and DM annotated repeats^6^. The RNA-seq data were aligned to the reference genome with HISAT2 (v2.0.4) and assembled using StringTie (v1.2.2)^53–55^. Trinity (v2.4.0) was used to assemble transcripts with (--genome_guided_max_intron 15,000 --genome_guided_bam --min_kmer_cov 2 --trimmomatic --normalize_reads) and without (--min_kmer_cov 2 --trimmomatic --normalize_reads --no_bowtie) reference guidance. To perform ab initio gene prediction, PASA (v2.2.0)^56^ was used to build the coding region model. This PASA step utilized assembled transcripts from Trinity as the library and trained the model. The ab initio predictions included SNAP (--categorize 100, --export 1,000 and --plus)^57^, AUGUSTUS (v2.7)^58^ and GlimmerHMM (v3.0.4)^59^. Two Trinity assemblies combined with ab initio gene prediction results were fed into EVM software (v1.1.1) to merge into a final gene set.

The annotated CDSs of the RH, DM and M6 genomes were aligned using BLAT^60^, and the homologous genes were screened in each genome using coverage of >75% and identity of >75% as the criteria.

### Haplotype comparison and diversity analysis

To identify the homologous regions between two haplotypes, we applied the MCScanX package^28^ to construct the syntenic blocks based on well-aligned genes. We screened the syntenic regions according to the following criteria: (1) paired regions must be on homologous haplotypes, (2) one segment should not be larger than three times the length of its counterpart and (3) aligned regions must cover over 50% of the whole region. Regions meeting these criteria were trusted as syntenic regions. One gene and its best homologous gene on the complementary haplotype were considered as allelic genes.

The syntenic regions were then subjected to LASTZ (v1.02.00)^30^ with the parameters --chain --format = diff --matchcount = 3,000 --rdotplot --strand = plus/minus --ambiguous = n. The homologous chromosomes were aligned using MUMmer (4.0)^29^, and the SVs were detected from the differences reported by the *show-diff* function. To reduce the number of false positives, we only identified the PAV genes in syntenic regions and defined a PAV gene as one that lacked a homolog at the complementary haplotype, while its surrounding genes had homologs that were arranged in good collinearity between two haplotypes.

The SNPs and indels between haplotypes were annotated using SnpEff^32^. To detect the mutations that were potentially deleterious, we aligned the RH chromosomes to the potato reference genome DM using LASTZ and performed in silico prediction on the SNPs through the ‘sorting intolerant from tolerant’ (SIFT) algorithm^33,34^. The underlying premise of this algorithm is based on the evolutionary conservation of the amino acid within protein families: highly conserved positions tend to be intolerant to substitution, whereas those within a low degree of conservation tolerate most substitutions.

### Gene expression analysis

The allele-specific mapping of Kallisto^36^ was used in the comparison of homologous expression in polyploid wheat^37^, and we applied the software to the RNA-seq data to obtain the expression levels in transcripts per million (TPM) of genes on both haplotypes. Only genes that showed <30% variance of expression levels in biological replicates were retained for further analysis. Genes with a summed TPM value of >1 for all tissues were taken as expressed genes. We then tested the expression difference between allelic genes with the *binom.test* using an R script.

### Methylation analysis

The whole-genome bisulfite sequencing reads from each sample were mapped to the RH genome using BSMAP^38^, allowing only unique mapping and mismatches of up to 4%. Positional DNA methylation levels were computed using the methratio.py script in the BSMAP package. To define differentially methylated positions (DMPs) between two haplotypes, we compared the methylation level of pairwise C sites in syntenic regions using Fisher’s exact test. We empirically used the reads depth of ≥5, CG difference of <0.4, CHG difference of <0.2, CHH difference of <0.1 and a *P* value < 0.01 derived from two-tailed Fisher’s exact test to screen the DMPs^61,62^. For each tissue, only the DMPs supported by all the three replicates were retained for further analysis. Then, the DMPs with the same content were collapsed into a DMR, only if the distance on the chromosome of the nearest two DMPs was less than 100 bp.

### Gene mapping of six loci related to inbreeding depression

#### Mapping based on the sequencing

To construct the genetic map, we scanned the chromosome using a 300-kb window, and the windows were genotyped as markers using the method described above. For each of the given 24 linkage groups, the markers were ordered on the genetic map using IciMapping (v4.0)^40^ with the parameters: LOD ≥ 3 and algorithm = nnTwOpt.

For *pa1*, *pa2* and *wv1*, the regular genetic mapping was performed using R/qtl^41^ (https://www.rqtl.org/) with the *cim* function, and the candidate interval was defined by the peak LOD bin and its adjacent two bins.

For the loci controlling growth vigor, *ar1*, *la2* and *ws1*, homozygous recessive genotypes were lethal and absent in the selfing population, impeding the effectiveness of the regular linkage mapping. Thus, we localized *ar1*, *la2* and *ws1* by screening the regions that excluded the homozygous recessive genotype.

#### Fine mapping using indel markers

To identify the recombinant plants of *ws1* and *pa1*, we sowed another 1,200 selfed seeds from RH on culture medium, and the seedlings were genotyped using the newly designed heterozygous indel primers in the candidate region (Supplementary Table 14).

#### Reverse transcription qPCR analysis of *RHC01H1G0699.2*

Total RNA was extracted from the leaves of *PA1*/*PA1* and *pa1*/*pa1* plants at the seedling stage using the TIANGEN kit with DNase I. The RNA was reverse transcribed using PrimeScript RT reagent kit with gDNA Eraser (Takara). Reverse transcription qPCR analysis was conducted with the StepOnePlus System (Applied Biosystems) using TB Green Premix Ex Taq GC (Takara). The reaction procedure was 95 °C for 30 s, 95 °C for 5 s and 60 °C for 30 s for 40 cycles. Actin was used as the internal control gene. All analyses were conducted with four biological replicates. The relative gene expression levels were calculated using the 2 − ΔCt method, and a *t*-test was performed to compare the results of *PA1*/*PA1* and *pa1*/*pa1* plants.

### Statistical analysis

The *χ*^2^ test statistic was performed using the *chisq.test* function in R. The expression difference between alleles was determined using the *binom.test* function in R (parameters: p = 0.5, alternative = two.sided, conf. level = 0.95). Two-tailed Student’s *t*-tests were calculated using *t.test* in R. The two-tailed Fisher’s test was performed using the *fisher.test* function in R.

### Reporting Summary

Further information on research design is available in the Nature Research Reporting Summary linked to this article.

## Online content

Any methods, additional references, Nature Research reporting summaries, source data, extended data, supplementary information, acknowledgements, peer review information; details of author contributions and competing interests; and statements of data and code availability are available at 10.1038/s41588-020-0699-x.

## Supplementary information

Supplementary Information Supplementary Notes, Figs. 1–17 and Tables 1–14

Reporting Summary

Supplementary Data 1 Graphs showing the alignment of BACs (*y* axis) and RHgv1 scaffolds (*x* axis).

Supplementary Data 2 Graphs showing the alignment of BACs (*y* axis) and RHgv2 unitigs (*x* axis).

Supplementary Data 3 Description of the construction of pseudochromsomes from smaller assembled fragments. The AGP file includes the order, position of contigs and gaps. The ontctg* and unitig* represent the contigs assembled from ONT and CCS data, respectively.

## Data Availability

The final RH genome assembly (RHgv3), annotation and a genome browser are available at Spud DB (https://solanaceae.plantbiology.msu.edu/rh_potato_download.shtml/). This whole-genome shotgun project has been deposited at GenBank under accession numbers JACDXL000000000 and JACDXM000000000. The raw sequencing data have been deposited in the NCBI Sequence Read Archive (https://www.ncbi.nlm.nih.gov/sra/) under BioProject accession number PRJNA573826. The data have also been submitted to the Chinese National Genomics Data Center (https://bigd.big.ac.cn/) under accession number CRA002005. Source data are provided with this paper.

## Source data

Source Data Fig. 2 The source data of Fig 2. Source Data Fig. 3 The source data of Fig 3.

## Code availability

The custom pipelines and scripts used in the project are deposited in GitHub (https://github.com/zhouqiansolab/Haplotype-resolved-potato-genome/).
