# Supplementary material for: Haplotype-resolved genome analyses of a heterozygous diploid potato
Source: Nat Genet. 2020 Sep 28;52(10):1018–23. doi: 10.1038/s41588-020-0699-x (PMC7527274; doi:10.1038/s41588-020-0699-x)
Supplement: Supplementary file 1 — Supplementary Notes, Figs. 1–17 and Tables 1–14 [file 41588_2020_699_MOESM1_ESM.pdf]

natureresearch

https://doi.org/10.1038/s41588-020-0699-x

Supplementary information

Haplotype-resolved genome analyses of a heterozygous diploid potato

In the format provided by the authors and unedited

Haplotype-resolved genome analyses of a heterozygous diploid potato

Qian Zhou[1,2#], Die Tang [1#], Wu Huang[3], Zhongmin Yang[4], Yu Zhang[1], John P. Hamilton[5], Richard G. F. Visser[6], Christian W. B. Bachem[6], C. Robin Buell[5], Zhonghua Zhang[7,3], Chunzhi Zhang[1], Sanwen Huang[1,3*]

[1] Shenzhen Branch, Guangdong Laboratory of Lingnan Modern Agriculture, Genome Analysis Laboratory of the Ministry of Agriculture and Rural Area, Agricultural Genomics Institute at Shenzhen, Chinese Academy of Agricultural Sciences, Shenzhen, China.

[2] Peng Cheng Laboratory, Shenzhen, China.

[3] Key Laboratory of Biology and Genetic Improvement of Horticultural Crops of the Ministry of Agriculture, Sino-Dutch Joint Laboratory of Horticultural Genomics, Institute of Vegetables and Flowers, Chinese Academy of Agricultural Sciences, Beijing, China.

[4] College of Horticulture, Northwest Agriculture and Forest University, Yangling, China.

[5] Department of Plant Biology, Michigan State University, East Lansing, MI 48824, USA.

[6] Plant Breeding, Wageningen University and Research, Droevendaalsesteeg 1, 6708 PB Wageningen, The Netherlands.

[7] College of Horticulture, Qingdao Agricultural University, Qingdao 266109, China.

[#] These authors contributed equally.

[*] For correspondence: huangsanwen@caas.cn

Supplementary Notes

Experiments of whole genome assembly of RH genome through ONT data. The raw 5,451,174 ONT reads (114 Gb, >5kb) were fed into SMARTdenovo (https://github.com/ruanjue/smartdenovo, default parameters), wtdbg[1] (-m 500, -bins 1), and miniasm (https://github.com/lh3/miniasm, -i 0.1) for *de novo* assembly. In addition, the raw ONT reads were corrected using CANU[2] (-minReadLength to 2000, -minOverlapLength to 500). After correction, 63 Gb ONT data were assembled using SMARTdenovo, wtdbg2, and Flye (version 2.4.2)[3]. The results are showed in Supplementary Table 2.

Pipeline to integrate the assemblies from Illumina WGS reads and 10XG reads. Because the assembly derived from the Illumina WGS data was very fragmented (contig N50=14.9 kb), we used its contigs as accurate long reads to improve the 10XG assembly. The WGS contigs were aligned to 10XG scaffolds using BLASR (v1.3.1)[4], and only alignments with 254 mapQV and >98% identity were used in the following process. To merge the two assemblies, we used a three-step process: 1) In the scaffolding step, the selected alignments that a WGS contig connects two 10XG scaffolds at the end were used to construct a Best Overlap Graph (BOG). After cutting tips and merging bubbles, the BOG was solved to be a genome draft; 2) In the gap filling step, we detected the alignment that a WGS contig spans a gap inside a 10XG scaffold and filled the gap using the contig sequence; 3) Lastly, the WGS contigs that could not be aligned to any 10XG scaffold were added into the merged assembly. The process was performed by custom Python scripts that could be found at Github https://github.com/zhouqiansolab/Merge-assemblies.

Principle to genotype assembled sequences through population sequencing. To determine the two haplotypes of the heterozygous potato, we sequenced a selfing population to provide the linkage information in haplotypes. In the absence of available molecular markers to distinguish the two haplotypes, a novel method was developed in this study. Among the assembled fragments, which can be considered as genetic markers, those derived from different haplotypes would segregate repulsively in homozygous regions, whereas those derived from the same haplotype would

co-segregate. This principle allowed us to determine the haplotypes of assembled fragments by genetic grouping. In the heterozygous diploid (the genotype is Aa), for the fragment representing the ‘A’ allele, according to the Mendelian inheritance rules, it would segregate as three genotypes, zero A (aa), single-copy A (Aa) and double-copy A (AA) in the segregating population, with the relationship of the three genotypes being 1:2:1. Thus, we could detect the aa, Aa or AA genotype of each fragment by examining its copy number. Because the individuals were sequenced at ~1X, the copy number could not be inferred correctly from the mapping depth in our study. We established that the copy number of a fragment can be reflected by the accumulation of reads number, which showed a tri-peak distribution indicating absent/single/double-copied fragment in each individual, separately (Supplementary Fig. 4). In this way, the aa/Aa/AA genotypes of assembled scaffolds were inferred. The related custom Python and R scripts could be found at Github https://github.com/zhouqiansolab/Haplotype-resolved-potato-genome/tree/master/04. Genetic_grouping.

Pipeline to integrate the assemblies from Illumina reads and CCS reads. Considering the RHv2 was generated from only 29Gb CCS reads, covering the diploid genome with ~17X (29Gb/1.7Gb), its completeness may be limited. By comparing the RHgv1 and the RHgv2 using nucmer, we found 275 Mb RH gv1scaffolds that mapped on RHgv2 unitigs with <20% coverage, indicating the completeness of RHgv2 needs to be improved.

Because the RHgv2 outperforms the RHgv1 on both sequence continuity and accuracy, we used RHgv2 as the principal assembly and RHgv1 as a supplementary assembly to generate a more complete, final assembly. To avoid including the expanded gaps that overestimated by the 10XG reads in the RHgv1 scaffolds, we used the RHgv1 contigs rather than scaffolds, to integrate with RHgv2 unitigs. As a rapidly genome aligner, nucmer reported fragmented exact matches between two ultra-long sequences, which was not suitable for deciding the accurate mapping endpoints or calculating the whole identity. So, the RHgv1 contigs were broken into 40kb chunks with remainders shorter than 40 kb unchanged and mapped to the RHgv2 assembly like the long read using BLASR[5]. We defined the fragments that aligned with <90%

identity or 50% coverage as supplemental sequences. If two or more adjacent fragments were defined as supplemental, they were merged as one.

Experiments of haplotype-resolved chromosome assembly on diploid genome using Hi-C. To test the application of Hi-C data on diploid genome assembly, the Hi-C processing software 3D-DNA[6] and ALLHIC (version 0.8.12)[7] were used to perform haplotype separation, based on the draft assembly produced by Flye[3] (Supplementary Table 2). The 3D-DNA pipeline generated 37 long-length super-scaffolds (>10 Mb) and 544 short-length super-scaffolds (-m diploid -i 15000 -r 0). To run the ALLHIC pipeline, the Flye contigs was aligned to the DM (version 4.03) reference genome using MUMmer[8] to generate allele.ctg.table according to aligned regions. Given an expected number of groups as 24, finally 6,917 contigs were grouped into 24 groups, accounting for a 98% assembly size. To assess the groups determined by 3D-DNA and ALLHIC, the Flye contigs were compared with the DM genome and plotted to show content of the groups (Supplementary Fig 2).

The overall view of the comparisons between homologous chromosomes. The homologous chromosomes, they were aligned in pairwise using MUMmer 4.0[8] to get coordinates of shared sequences. To view the genome-wide distribution of heterozygosity, the haplotypes with a longer assembly length for each chromosome were combined to generate a pseudo-haploid genome and as a reference in the following SNP calling. Then, the WGS Illumina reads of RH were mapped to this pseudo-haploid genome using BWA-MEM[9] to screen the heterozygous SNPs. The pairwise alignment of homologous haplotypes revealed that some haplotype pairs display significant inequality of length, such as chr3_1 and chr3_2 (Supplementary Fig. 9). Analyses of heterozygosity and sequence alignments revealed that such length inequalities were usually in regions with low heterozygosity, which impeded the haplotype partitioning and assembly.

Supplementary Figures

Supplementary Figure 1. The 35-mer analysis of RH genome

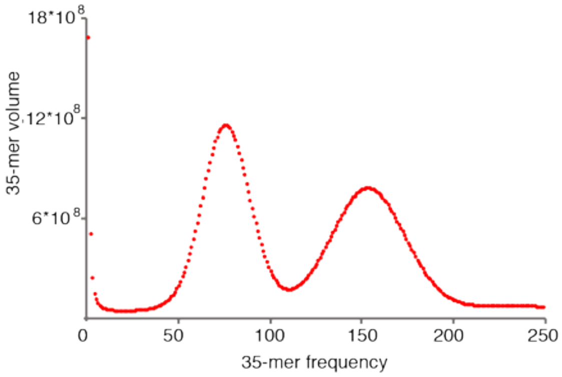

The frequency of occurrence of 35-mers was generated from the whole genome Illumina reads of RH. Different from the homozygous genome or a lowly heterozygous genome, the volume histogram of RH displays clear bimodal distribution caused by the high heterozygosity. The first and second peaks represent the volume of k-mers derived from the heterozygous and homozygous regions on genome, respectively. Excluding sequencing errors (k-mer with frequency=1), the total 35-mer number is 122,201,033,905. The first and second frequency peaks are at 74 and 152, respectively. Thus, the diploid genome size can be estimated as 1,651Mb using (total 35-mer number)/(the first frequency peak).

Supplementary Figure 2. Test of haplotype partitioning of the RH genome using Hi-C data.

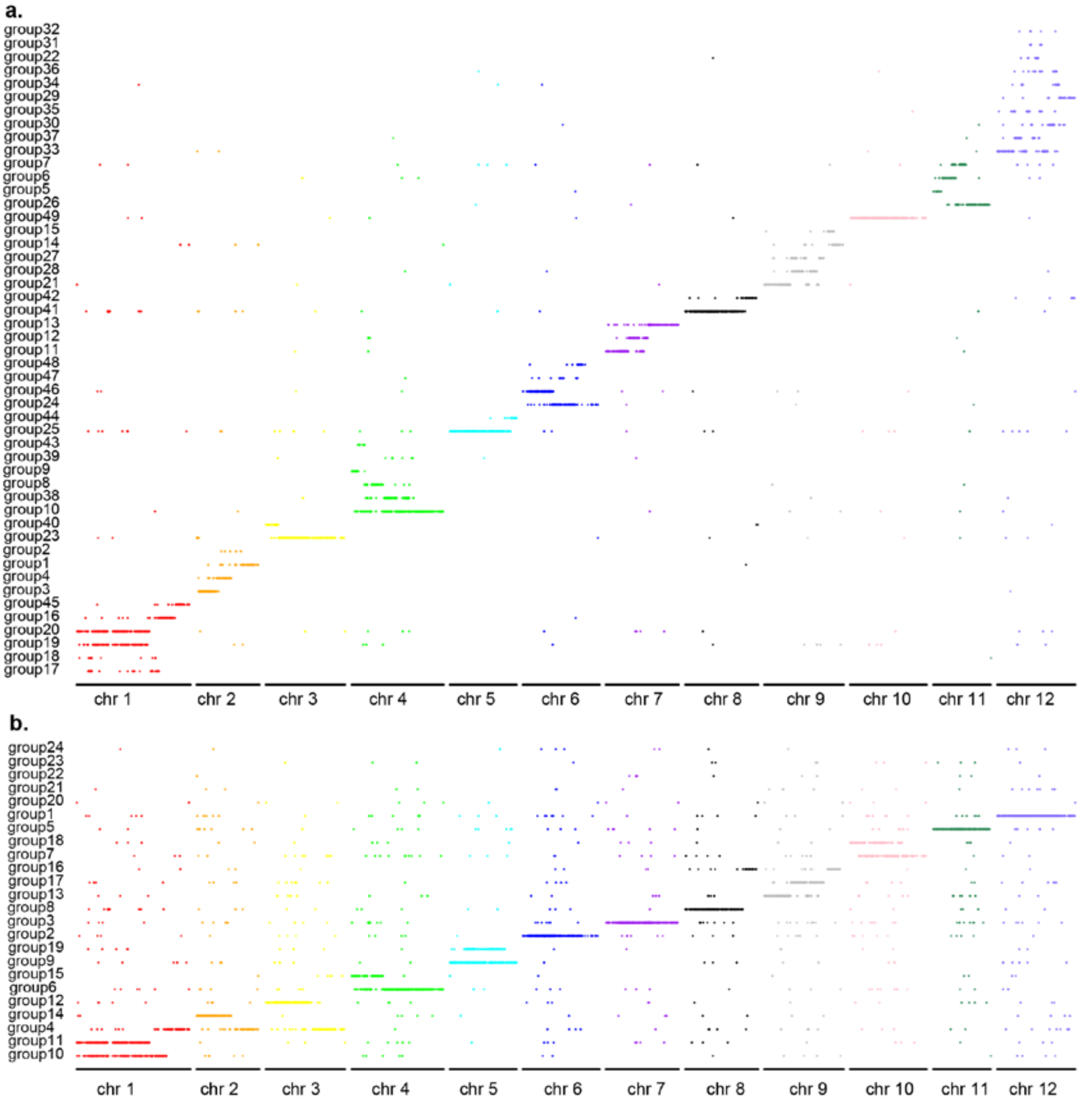

The groups are generated using 3D-DNA (a) and ALLHIC (b). Each group (y-axis) contains dots arranged in one row, which represent the RH contigs assembled from ONT reads, using Flye. The dots with same color are considered to belong to same chromosome according to the alignment between contigs and the haploid potato reference genome (x-axis). From the dots plot, the 3D-DNA tends to produce fragmented groups while the ALLHIC tends to generate fused groups for the two haplotypes.

Supplementary Figure 3. A general assembly and phasing pipeline for haplotype-resolved diploid genome.

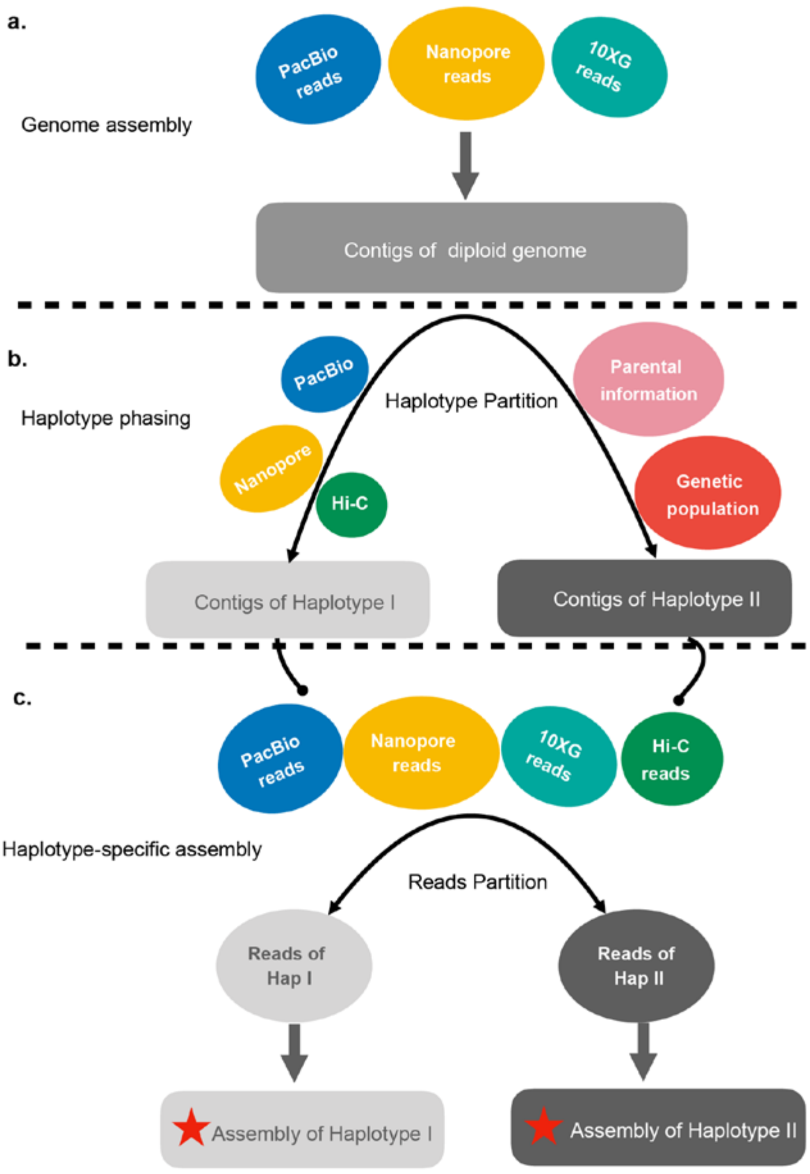

The pipeline includes three stages, Genome assembly (a), Haplotype phasing (b) and Haplotype-specific assembly (c). Colored ellipses represent the input sequencing data; gray rounded rectangles represent assembly output. Considering the data accessibility for specific species, the type and quantity of input sequencing data is optional at each stage. Based on the purpose of a project, the pipeline can be terminated at any stage. For example, if the outcome of stage (b) is satisfying, the stage (c) can be omitted.

Supplementary Figure 4. Inferring genotype from distribution of read number.

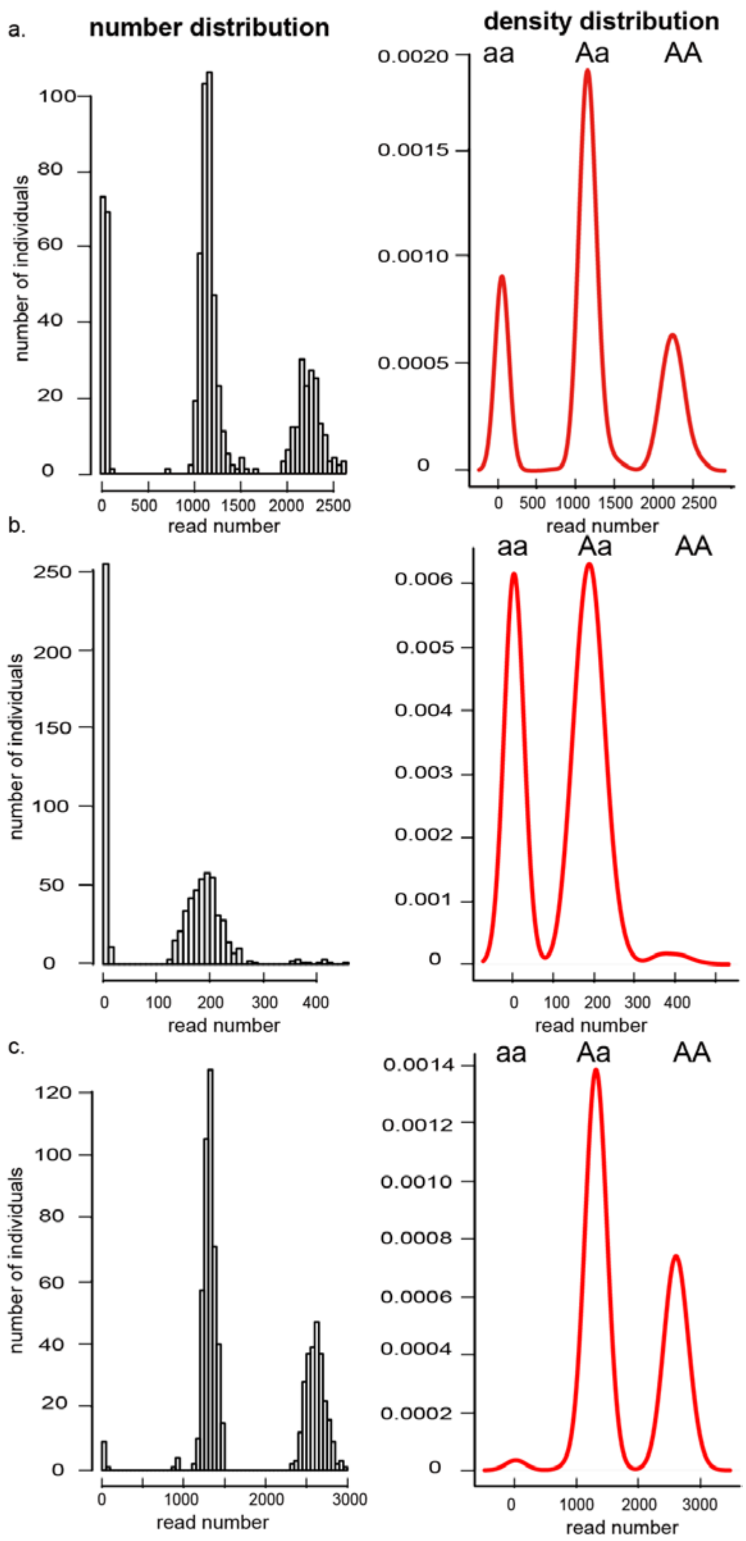

For one fragment, the read number mapped on it is calculated independently for 880 individuals, and the grouped histogram exhibits the frequency at which a specific read number occurs in the population (left). The density distribution is calculated from the number distribution to display the

distribution more clearly (right). **a**, For one fragment that segregates via Mendel's law, the distribution of the read number shows a three-peak distribution in population (left), indicating 0/1/2 copy number of this fragment in individuals, representing the aa/Aa/AA genotype, respectively (right). **b-c**, For the fragments with distorted segregation, the read number exhibits a more bimodal distribution. Thus, the genotype of one fragment in individual is inferred according to the crest that its read number belongs to.

Supplementary Figure 5. Flowchart of genetic genotyping and grouping.

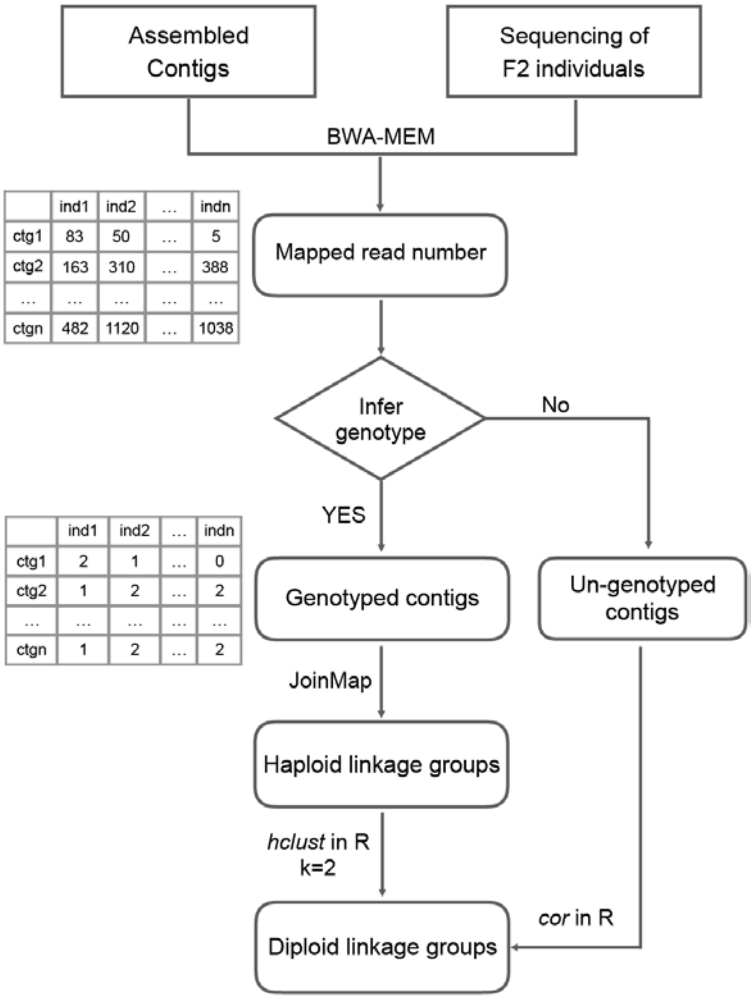

The 'Assembled contigs' and 'Sequencing of F_2 individuals' are the primary input of this pipeline. The 'Mapped read number' is calculated from the sam files generated using BWA-MEM. A matrix is generated to store the data with rows representing contigs (ctg1, ctg2, ..., ctgn) and columns representing individuals (ind1, ind2, ..., indn). After the 'Infer genotype' stage, some contigs are genotyped and the read number matrix is transformed to a genotype matrix. The 'Genotyped contigs' are processed using JoinMap and *hclust* in R to generate 'Diploid linkage group'. To assign the 'Un-genotyped contigs' that are unable to be genotyped in 'Infer genotype' stage, the correlation of 'Un-genotyped contigs' and 'Genotyped contigs' is calculated using the *cor* in R based on the 'Mapped read number' matrix.

Supplementary Figure 6. Haplotype partitioning of the RH genome using genetic grouping.

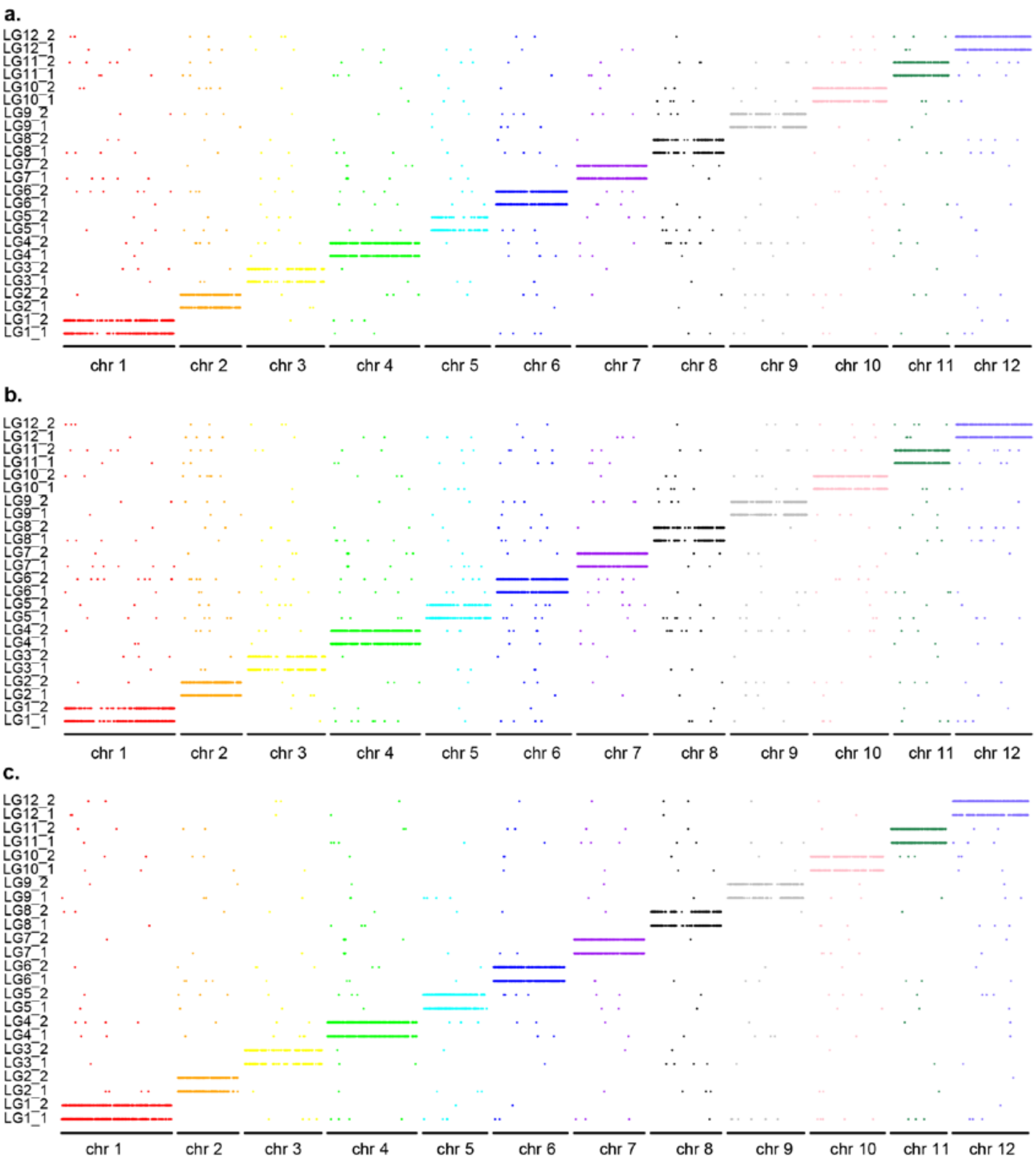

The linkage groups generated using 100 (a),150 (b) and 880 (c) F_2 individuals showed partitioning results of RH assembly, generated from 10XG reads. Each group (y-axis) contains dots arranged in one row, which represent the scaffolds of RH assembly. The dots with same color are considered to belong to same chromosome according to the alignment between scaffolds and the haploid potato reference genome (x-axis). The three genetic maps include 1,405 Mb, 1,404 Mb and 1,523 Mb sequences, respectively.

Supplementary Figure 7. Genome-wide Hi-C contact matrix at 500 kb resolution in the chromosome-level assembly of RH genome (RHgv3)

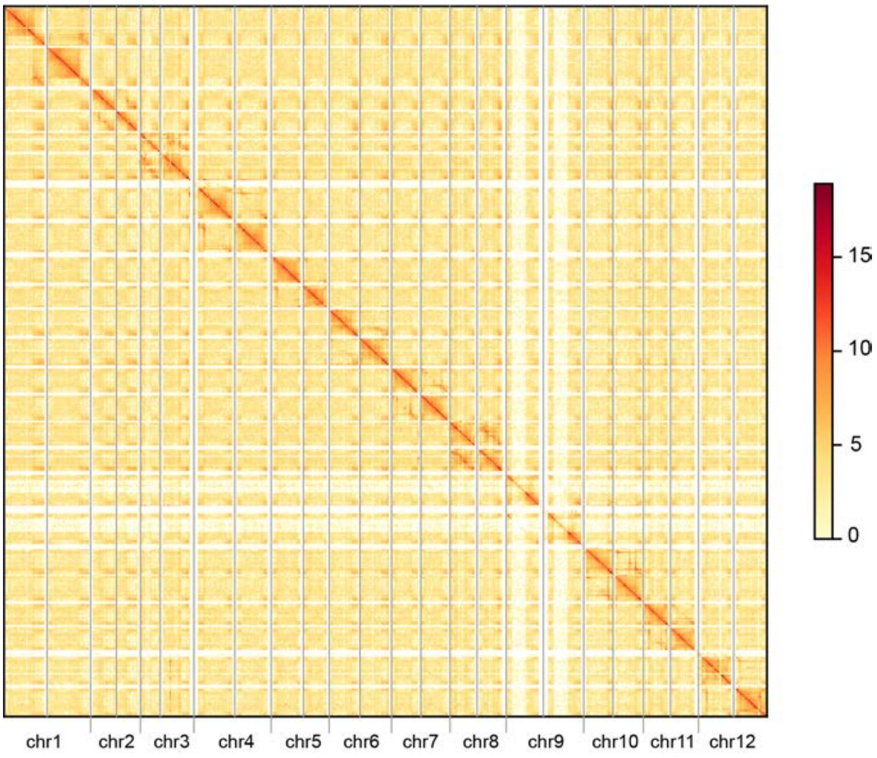

The diagonal lines represent the frequency of contact between two 500 kb loci on a chromosome.

Supplementary Figure 8. Tuber shape and the number of orthologous genes in RH, M6 and DM.

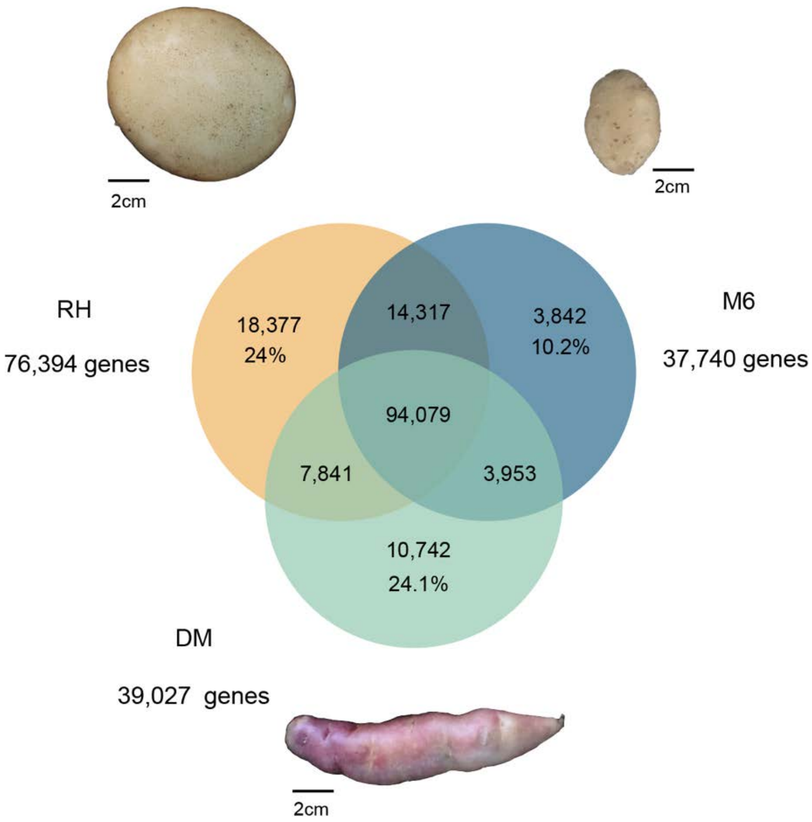

The Venn diagram shows the number of homologous genes in RH, M6 and DM genomes identified in the three annotations. The number in each component is the sum of all of the potatoes within the component.

Supplementary Figure 9. Heterozygosity and synteny between homologous chromosomes.

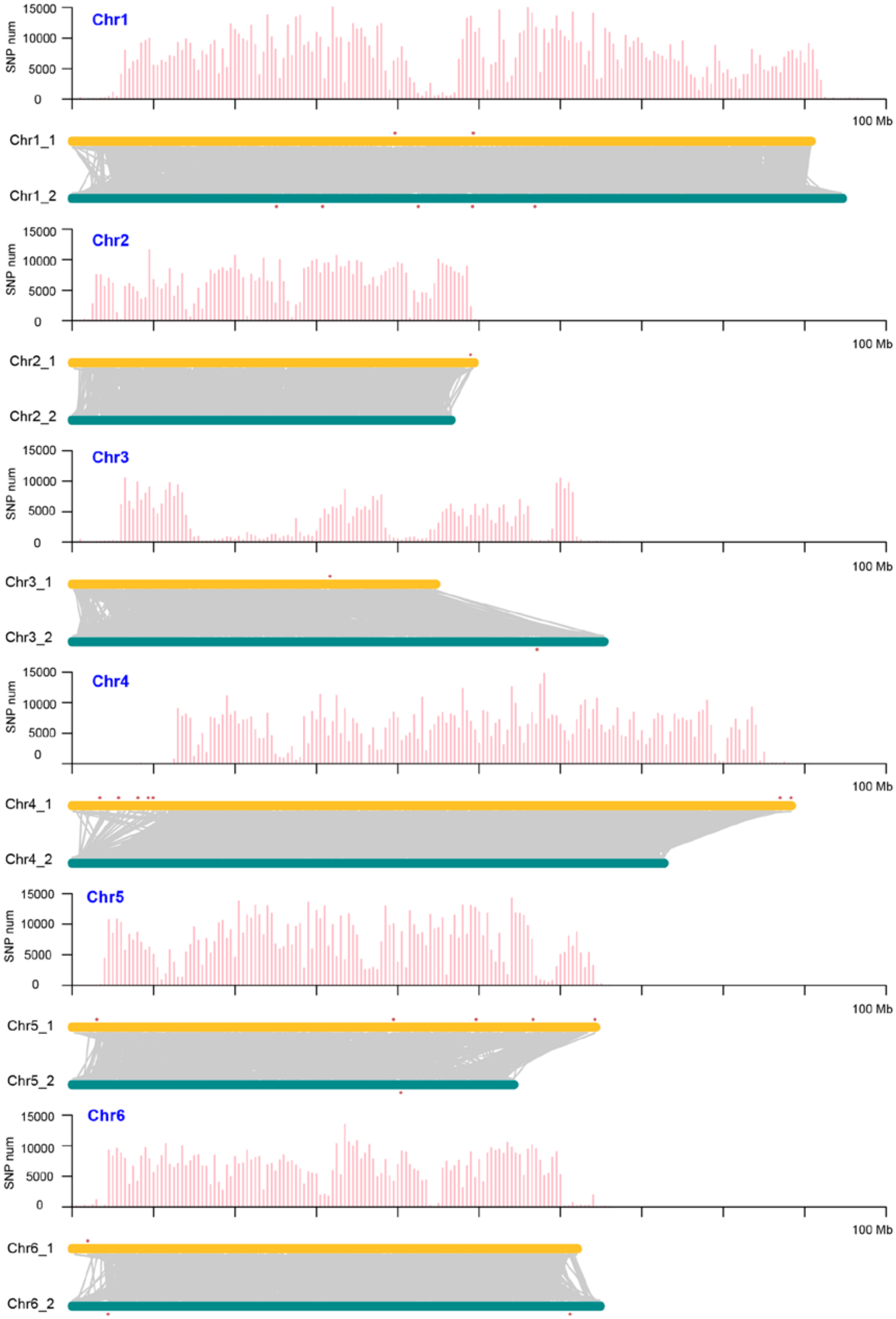

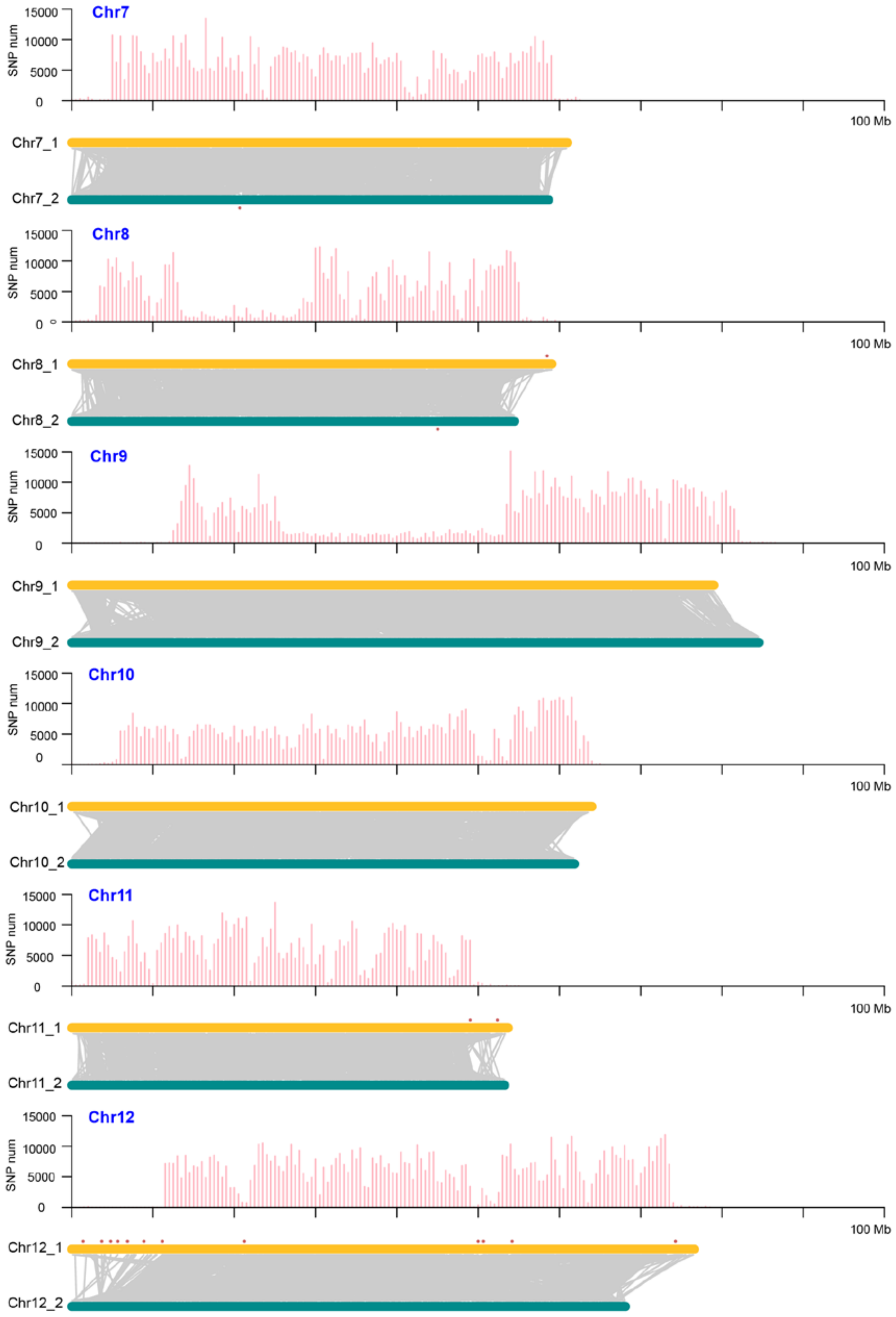

The upper histogram shows the number of heterozygous SNP detected between homologous haplotypes using whole genome Illumina reads. The numbers are counted in each 500 kb window. Lower bars represent the two haplotypes of potato chromosomes; gray lines indicate the aligned

sequences between the haplotypes. Red dots above and below the colored bars mark regions (>500 kb) that lack well-aligned sequences on opposite haplotype. Histogram and bar plot share the same x-axis coordinate.

Supplementary Figure 10. Haplotype divergence of RH chromosome 2

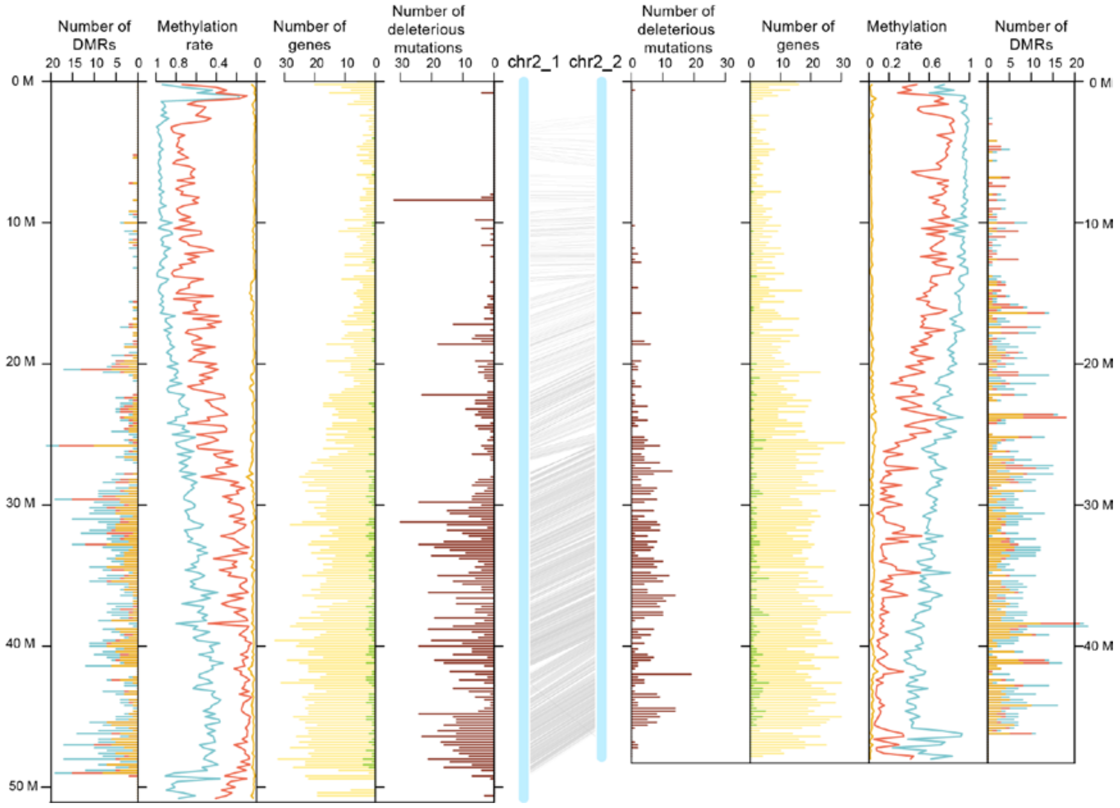

The central blue bars represent the two haplotypes of chromosome 2 with the gray lines indicating the paired allelic genes. The distribution of deleterious or dysfunctional mutations (brown), annotated genes (yellow), preferentially expressed alleles (green), methylation level of three contexts and differentially methylated regions are arranged symmetrically for each haplotype. The methylation level and the number of DMRs of methylated sites in CG (light blue), CHG (red) and CHH (orange) contexts are indicated by cumulative column chart. Number of DMRs on one haplotype only involves the DMRs with hyper-methylation. All of the numbers were determined in 200 kb windows.

Supplementary Figure 11. Distribution of differentially methylated regions (DMRs).

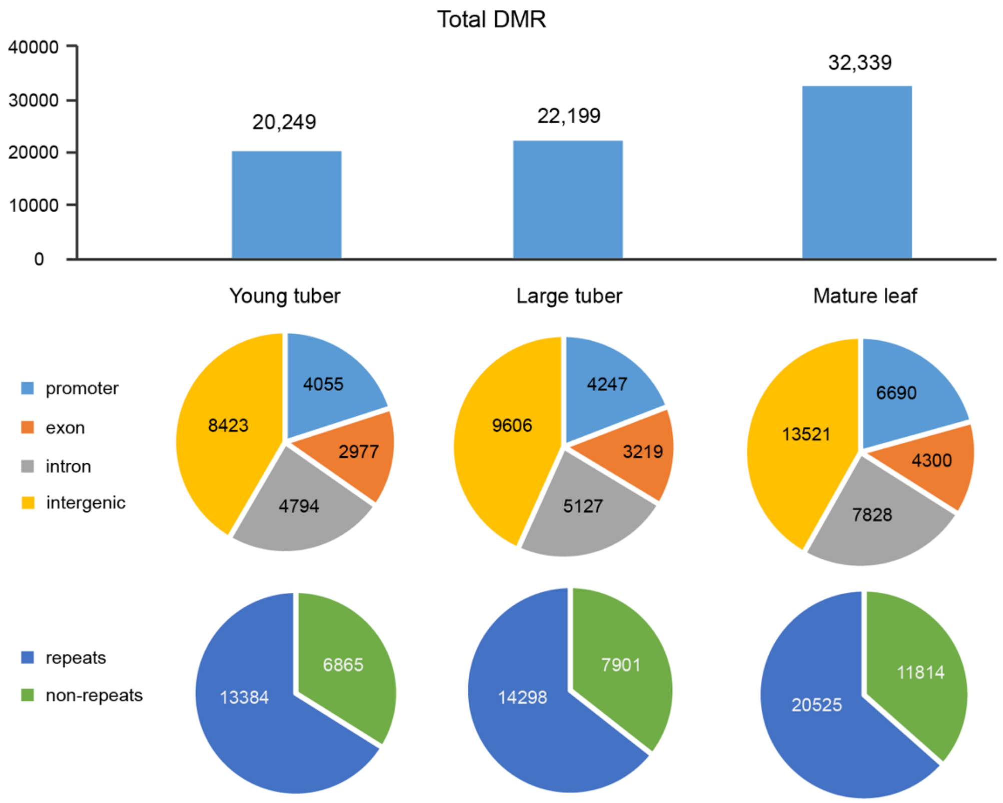

The histogram indicates the number of DMRs in three tissues and the pie charts below indicate the categories of the location of DMRs.

Supplementary Figure 12. Number of DELs located in DMRs and the correlation between expression and methylation.

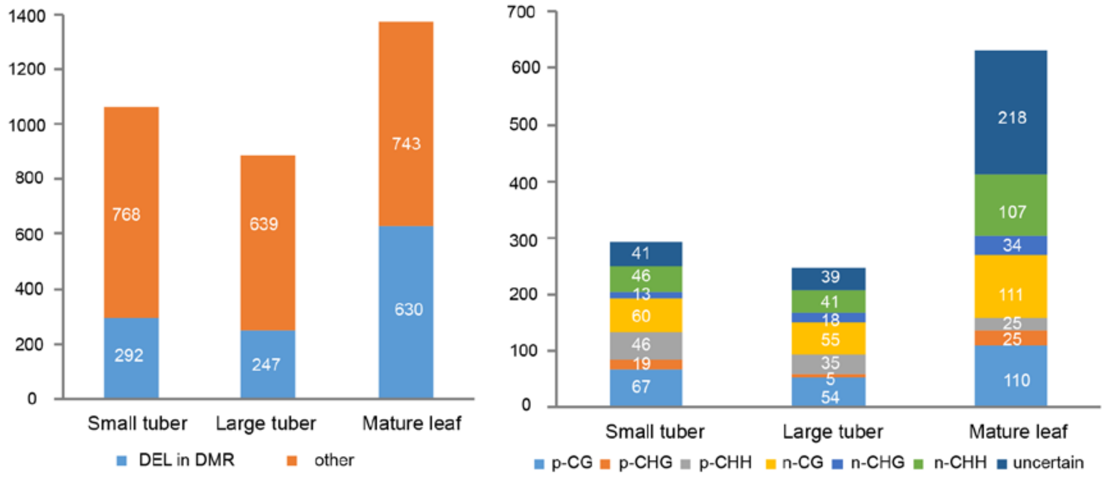

The left bar chart shows the number of DEL in three tissues. The right bar chart display the correlation between expression and methylation level of DEL in DMRs. p-CG, the number of DELs whose expression level shows positive correlation with CG methylation level; n-CG, the number of DEL whose expression level shows negative correlation with CG methylation level; p-CHG, p-CHH, n-CHG and n-CHH have similar interpretation with p-CG and n-CG; uncertain, represents the number of genes with multiple methylation types.

Supplementary Figure 13. Segregation distortion in the RH F_2 population.

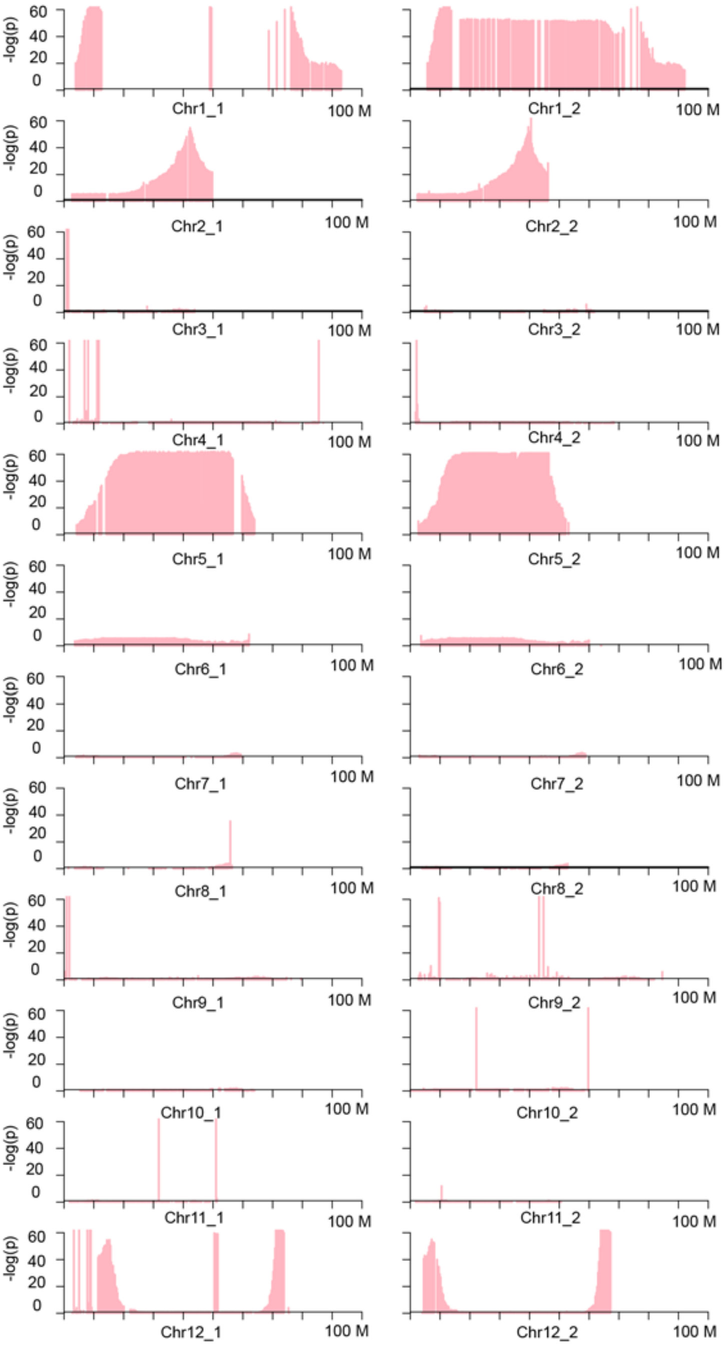

The segregation ratio of genotypes is calculated in 300 kb window based on the sequencing of 880 F_2 progeny. The y-axis represents the -log(p) and p is calculated in the chi-square ($\chi 2$) test for each window.

Supplementary Figure 14. The *WV1* and *wv1* phenotypes.

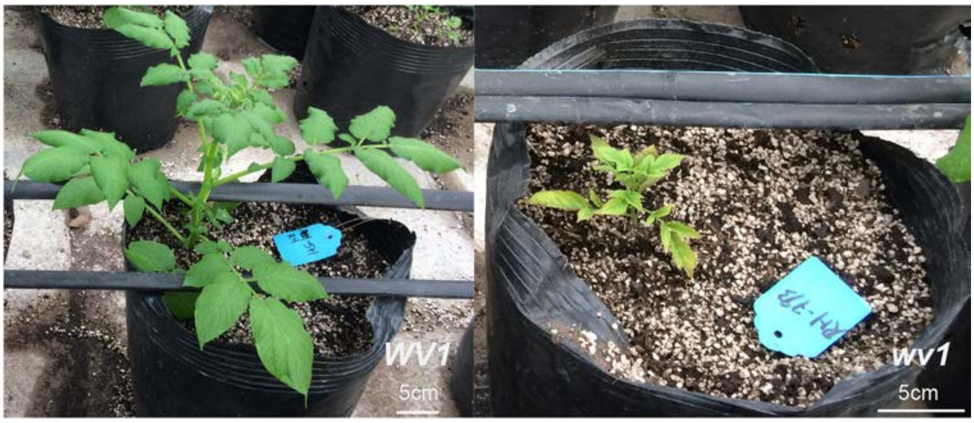

The plants are grown in same greenhouse and the photos are taken at 30 days after the transplanting of seedlings.

Supplementary Figure 15. Genetic mapping of *WV1*, *PA2* and *la2*.

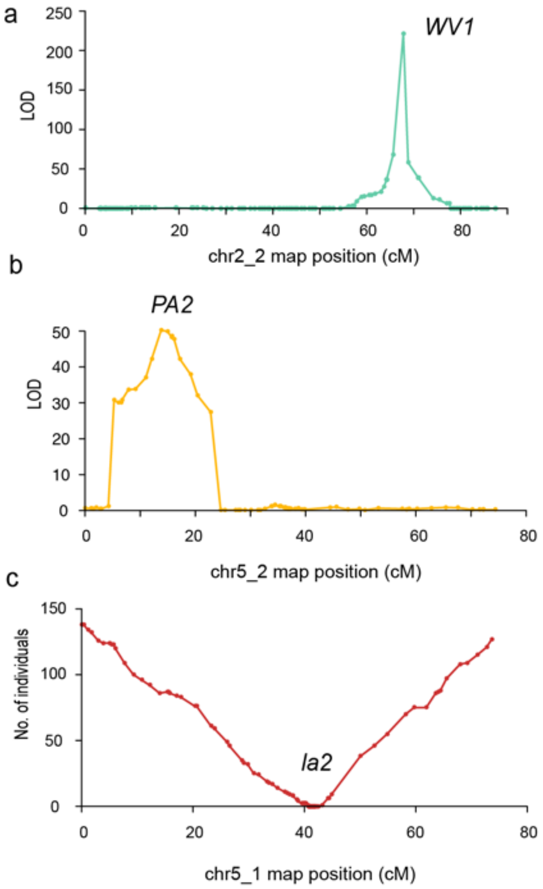

a-**b**, The LOD values of *WV1* and *PA2* gene mapping performed using *Rqtl*. **c**. Number of individuals harboring homozygous recessive alleles for each genetic marker. For the lethal allele *la2*, there is no recessive phenotype data for mapping; thus, its location was inferred from the absence of homozygous alleles in the population. Dots in the graphs represent the continuous 300 kb genomic windows, which are genotyped and employed as genetic markers in gene mapping.

Supplementary Figure 16. Full-length CDS alignment of *RHC01H1G0699.2*, *RHC01H2G0765.2* and *PGSC0003DMG400008712*.

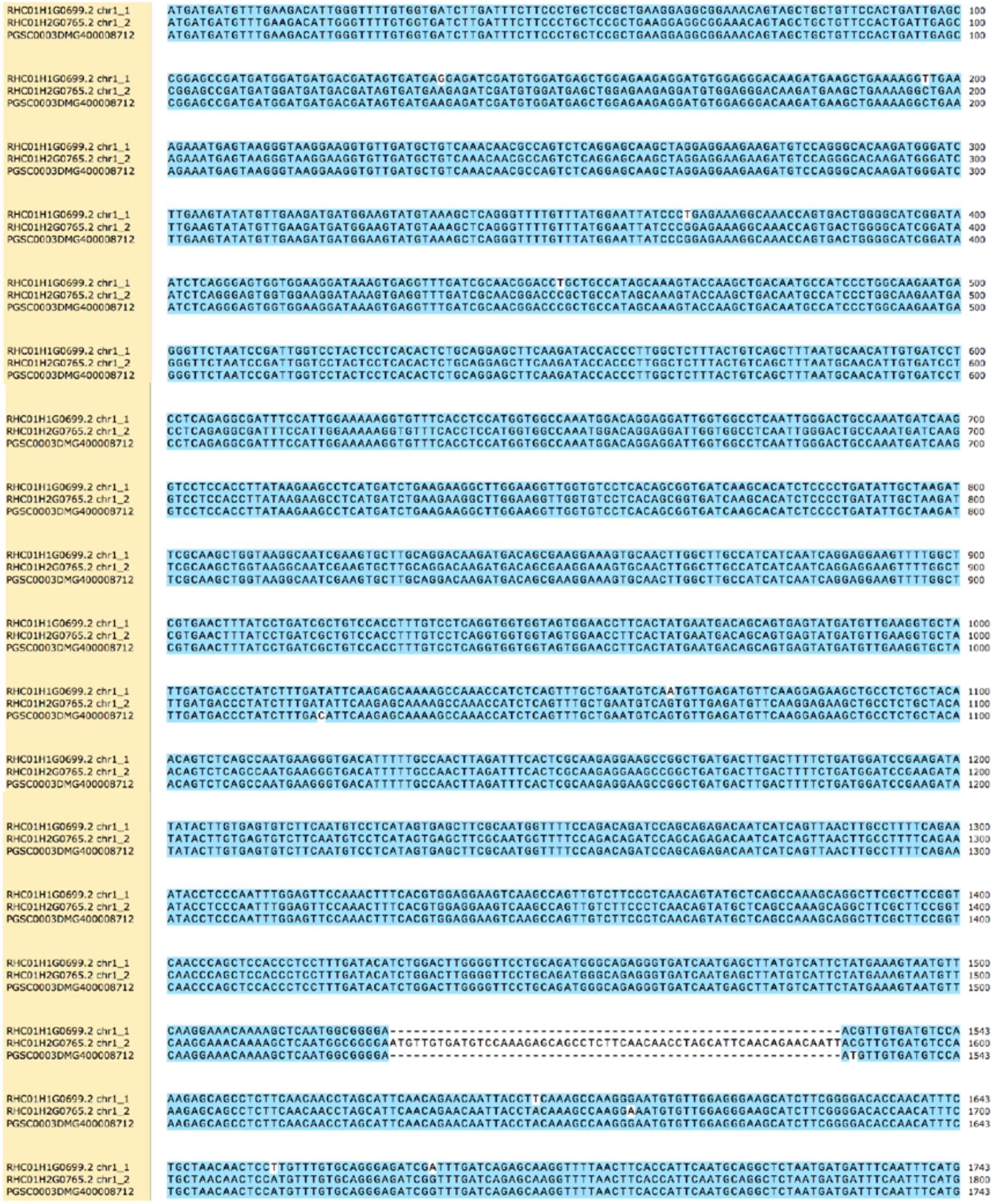

The *RHC01H1G0699.2* and *RHC01H2G0765.2* are the candidate alleles of *PA1* and *pa1*, respectively. Compared with *RHC01H1G0699.2* and *PGSC0003DMG400008712*, *RHC01H2G0765.2* harbors a 57 bp insertion at the 1,529 bp of the CDS.

Supplementary Figure 17. Conceptual protein alignment of RHC01H1G0699.2, RHC01H2G0765.2 and PGSC0003DMG400008712

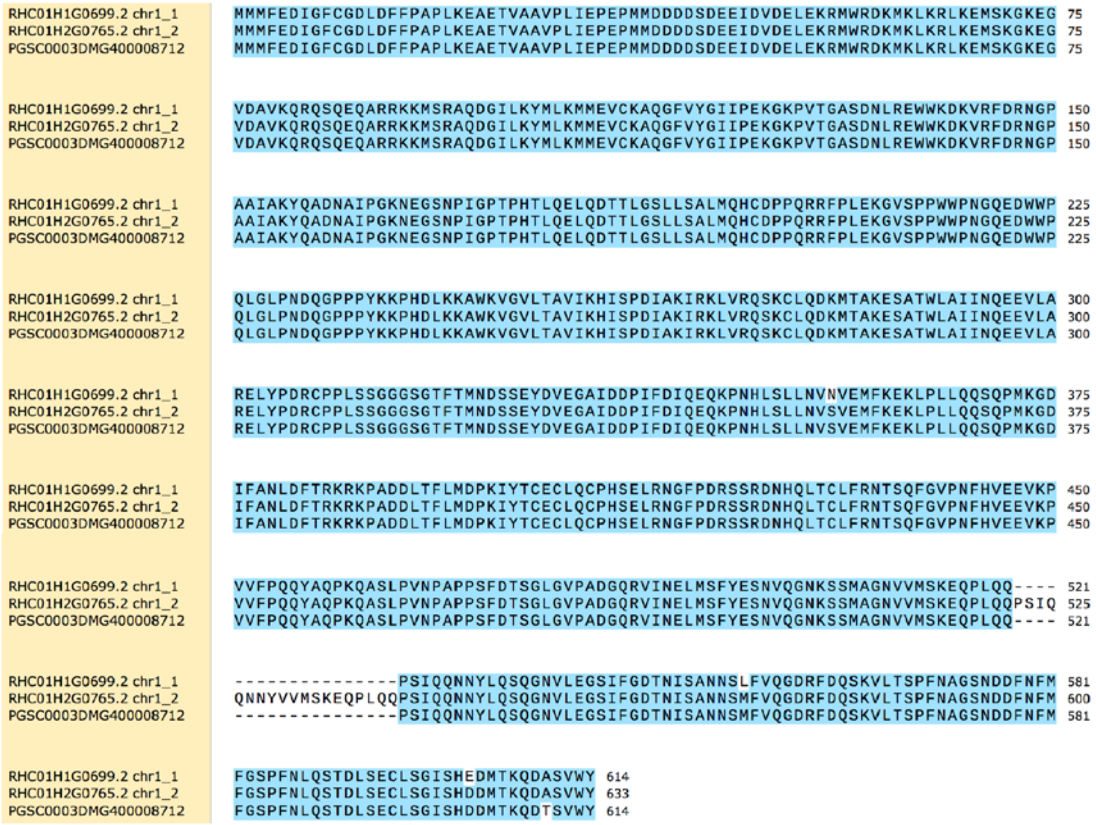

Supplementary Tables

Supplementary Table 1. Summary of sequenced data used for assembly.

Source	Library	Sequencer	Data size(Gb)
RH	WGS	Illumina Hiseq 2500	150
RH	10X Genomics	Illumina X Ten	122
RH	Nanopore	ONT MinION	120
RH	Hi-C	Illumina X Ten	150
RH	SMRTbell	PacBio Sequel II	29
880 F_2 progeny	WGS	Illumina X Ten	2,000
Total	-	-	2,571

Supplementary Table 2. Statistics of *de novo* assembly of the RH genome using ONT reads.

Data	Raw ONT reads			Corrected ONT reads[a]		
Software[b]	smartdenovo	wtdbg[1]	miniasm[c]	smartdenovo	wtdbg	[d]Flye[3]
Total	1,177 Mb	1,674 Mb	1,478 Mb	1,020 Mb	1,050 Mb	1,537 Mb
N10	1,756,648	408,319	2,849,939	2,183,518	1,430,494	1,436,404
N30	995,159	184,922	1,376,227	1,197,383	558,134	722,653
N50	640,366	95,590	744,012	687,574	246,882	408,984
N70	391,685	14,481	398,268	396,594	91,687	213,446
N90	153,559	3,705	147,997	171,599	28,226	90,837
Minimum	11,354	3,705	1,649	16,609	2,718	126

a. ONT reads were corrected using CANU[2]. The process of CANU assembly was terminated after running more than one month on a pan node.

b. For each software, we tested several parameters and displayed the best result with the longest Total or N50 length.

c. This assembly showed a poor BUSCO[10] evaluation result (C:84.3%, D:4.6%) after three rounds of polishing, so the assembly was discarded.

d. This assembly showed an acceptable BUSCO[10] evaluation result (C:91.2%, D:49.5%) after three rounds of polishing.

Supplementary Table 3. Statistics of *de novo* assembly of the RH genome using Illumina reads.

	Scaffold statistics			Contig statistics		
Draft	WGS_asm	10XG_asm	Merged_asm	WGS_asm	10XG_asm	Merged_asm
Total	1,329 Mb	1,580 Mb	1,718 Mb	1,329 Mb	1,299 Mb	1,464 Mb
N10	60,322	1,511,035	1,524,930	60,322	67,755	110,875
N30	28,190	652,161	640,480	28,190	35,931	55,799
N50	14,971	318,415	300,544	14,971	20,286	31,608
N70	6,323	85,621	61,786	6,323	9,222	16,276
N90	401	3,357	6,751	401	2,502	4,675

Supplementary Table 4. Statistics of *de novo* assembly of the RH genome using PacBio CCS reads.

Statistics	Contig	Unitig
Total	1,489 Mb	1,531 Mb
N10	19,688,908	9,261,245
N30	11,546,204	4,218,983
N50	7,097,129	2,192,152
N70	2,825,298	853,109
N90	391,723	99,251

Supplementary Table 5. Assessment metrics of genome assemblies using BACs and BAC-ends (BEs).

Genome draft	RHgv1 [a]	RHgv2 [b]	RHgv3[c]
Total /Kb	1,688	1,531	1,673
Contig N50 /Kb	582	2,192	1,743
Scaffold N50 /Kb	921	2,192	1,743
Grouped in genetic mapping	1,520	1,312	1,540
Total BE pairs	54,902	54,902	54,902
Uniquely mapped BE pairs	54,490	54,761	54,759
BE pairs with both ends hit the same scaffold (ratio)	23,874 (100%)	39,914 (100%)	37,521 (100%)
BE pairs mapped with reasonable distance (ratio)	22,976 (96.2%)	39,513 (99.0%)	37,134 (99.0%)
BE pairs mapped with reasonable distance and orientation (ratio)	22,551 (94.5%)	39,475 (98.9%)	37,094 (98.9%)
Total BACs	184	184	184
Mapped to a single fragment	126	169	170
Mapped with perfect structure	113	152	153
Mapped with structure variation	13	17	17
Base-level accuracy	99.127%	99.936%	99.938%
Complete BUSCOs[d]	1,351	1,397	1,398
Complete and single-copy BUSCOs	551	381	362
Complete and duplicated BUSCOs	800	1,016	1,036
Fragmented BUSCOs	49	23	23
Missing BUSCOs	40	20	19

[a] RHgv1 is the previous assembly generated from 10XG and ONT reads.

[b] RHgv2 is the assembly generated from CCS reads.

[c] RHgv2 is the assembly merged from RHgv1 and RHgv2. The assessment is performed on the scaffolds of RHgv3.

[d] The evaluations were performed on the genome annotations of the three assemblies with a total of 1440 BUSCOs[10].

Supplementary Table 6. The length of 24 linkage groups of RHgv3.

Group	Length	Group	Length
LG1_1	90,695,541	LG1_2	95,194,308
LG2_1	48,403,993	LG2_2	46,744,060
LG3_1	40,389,235	LG3_2	58,667,064
LG4_1	86,391,033	LG4_2	71,829,293
LG5_1	63,675,086	LG5_2	54,592,690
LG6_1	63,060,731	LG6_2	65,986,297
LG7_1	61,287,105	LG7_2	58,689,328
LG8_1	51,179,054	LG8_2	48,127,672
LG9_1	78,936,687	LG9_2	84,155,889
LG10_1	63,164,883	LG10_2	61,410,769
LG11_1	50,906,189	LG11_2	52,883,149
LG12_1	75,467,413	LG12_2	68,672,349

Supplementary Table 7. Statistics of Hi-C data mapping[a].

Items	Number of Reads	Ratio
Total_pairs_processed	456,408,559	100.00%
Unmapped_pairs	2,621,158	0.57%
Low_qual_pairs	281,271,961	61.62%
Unique_paired_alignments	140,713,827	30.83%
Pairs_with_singleton	31,801,613	6.97%
Reported_pairs	140,713,827	30.83%
**Items**	**Valid pairs**	**Ratio**
valid_interaction	111,984,338	100.00%
valid_interaction_rmdup	84,699,970	75.64%
trans_interaction	46,375,165	41.41%
cis_interaction	38,324,805	34.22%
cis_shortRange	13,165,006	11.76%
cis_longRange	25,159,799	22.47%

[a] The Hi-C reads were mapped to scaffolds of RHgv3.

Supplementary Table 8. Metrics of haplotype-resolved assembly of RH genome.

RH Haplotype I				
Chr.	Scaffold length	Scaffold N50	Chr. length	Gene number
chr1_1	88,553,395	3,077,756	88,578,195	4,548
chr2_1	50,747,354	3,320,060	50,771,054	3,303
chr3_1	45,882,374	960,847	45,923,574	2,596
chr4_1	90,789,252	943,739	90,839,752	3,448
chr5_1	66,090,562	3,574,845	66,116,962	2,863
chr6_1	63,775,926	4,388,815	63,795,926	3,208
chr7_1	62,664,674	2,038,752	62,696,774	2,696
chr8_1	60,492,270	416,374	60,552,370	2,726
chr9_1	81,152,403	960,269	81,196,603	3,188
chr10_1	65,685,148	1,600,624	65,721,548	2,948
chr11_1	55,161,317	1,857,140	55,192,017	2,656
chr12_1	78,696,427	2,716,915	78,738,127	2,935
Total	809,691,102	1,725,596	810,122,902	37,115
RH Haplotype II				
Chr.	Scaffold length	Scaffold N50	Chr. length	Gene number
chr1_1	97,247,411	3,572,217	97,287,411	4,771
chr2_2	47,836,841	3,476,250	47,857,541	3,158
chr3_2	67,013,793	739,908	67,071,493	3,413
chr4_2	74,701,631	1,911,641	74,744,231	3,252
chr5_2	55,705,483	5,231,540	55,732,883	2,441
chr6_2	66,682,274	2,882,834	66,706,574	3,523
chr7_2	60,269,170	3,000,000	60,296,770	2,597
chr8_2	55,933,944	891,528	55,985,344	2,757
chr9_2	86,853,815	773,391	86,903,315	3,313
chr10_2	62,580,608	6,413,953	62,608,408	2,630
chr11_2	54,713,364	2,030,822	54,740,564	2,426
chr12_2	69,722,361	4,742,410	69,752,061	2,813
Total	799,260,695	2,059,535	799,686,595	37,094

Supplementary Table 9. Overview of assembled potato genomes.

Species	*S.tuberosum* L.	*S.commersoni*	*S.chacoense*	*S. tuberosum* L.
Clone	DM[11]	cmm1t[12]	M6[13]	RH
Ploidy	doubled monoploid	diploid	diploid	diploid
Heterozygosity	0	1.49%	0.68%	2.1%
Genome size	884 Mb	830 Mb	826 Mb	1,672 Mb
Anchor method	BAC+genetic map	-	genetic map	genetic map,HiC
Anchor rate	82%	-	62%	97%
Haplotype-resolved	Yes	No	No	Yes

Supplementary Table 10. Statistics of inter-haplotype synteny and variation.

Specification	Results
Syntenic block size	657 Mb (hap0) [a], 658 Mb (hap1)
Syntenic block number	198
Number of paired genes from BLAST	59,907
Number of allelic genes	44,166 (20,583 pairs)
Number of SNPs	12,299,445 (1.9%)
Numbers of InDels	1,393,680 (0.2%)
Numbers of SV	38,999
Number of PAV genes	1,878

[a] The hap0 is composed of the pseudo-chromsome chr1_1, chr2_1, ... and chr12_1; the hap1 is composed of the pseudo-chromsome chr1_2, chr2_2, ... and chr12_2.

Supplementary Table 11. Numbers of predicted deleterious mutations (DEM) on each haplotype

Chr.	Coding sequencs	No. of DEM	Ratio of DEM	Chr.	Coding sequencs	No. of DEM	Ratio of DEM
chr1_1	5,566,963	1465	0.03%	chr1_2	5,219,831	973	0.02%
chr2_1	4,030,268	1289	0.03%	chr2_2	3,870,098	581	0.01%
chr3_1	2,950,447	772	0.03%	chr3_2	3,770,385	774	0.02%
chr4_1	3,694,758	1065	0.03%	chr4_2	3,499,320	576	0.02%
chr5_1	2,911,761	830	0.03%	chr5_2	2,579,484	1076	0.04%
chr6_1	3,415,399	878	0.03%	chr6_2	3,899,277	1446	0.04%
chr7_1	3,022,340	820	0.03%	chr7_2	2,908,798	615	0.02%
chr8_1	2,992,384	829	0.03%	chr8_2	3,065,657	691	0.02%
chr9_1	3,344,098	1180	0.04%	chr9_2	3,464,920	1200	0.03%
chr10_1	2,935,025	804	0.03%	chr10_2	2,637,100	839	0.03%
chr11_1	2,854,170	738	0.03%	chr11_2	2,622,937	694	0.03%
chr12_1	3,049,985	864	0.03%	chr12_2	2,934,640	1135	0.04%

Supplementary Table 12. Numbers of higher expressed alleles (HEAs) on each haplotype.

Chr.	No. of Gene	No. of HEA	Ratio of HEA	Chr.	No. of Gene	Ratio of HEA	Ratio of HEA
chr1_1	4,548	247	5.20%	chr1_2	4,771	245	5.14%
chr2_1	3,303	187	5.66%	chr2_2	3,158	210	6.65%
chr3_1	2,596	126	4.85%	chr3_2	3,413	120	3.50%
chr4_1	3,448	154	4.47%	chr4_2	3,252	169	5.19%
chr5_1	2,863	91	3.18%	chr5_2	2,441	130	5.26%
chr6_1	3,208	203	6.33%	chr6_2	3,523	178	5.05%
chr7_1	2,696	122	4.52%	chr7_2	2,597	132	5.08%
chr8_1	2,726	122	4.40%	chr8_2	2,757	117	4.24%
chr9_1	3,188	133	4.16%	chr9_2	3,313	166	5.01%
chr10_1	2,948	123	4.17%	chr10_2	2,630	126	4.75%
chr11_1	2,656	90	3.39%	chr11_2	2,426	108	4.45%
chr12_1	2,935	154	5.25%	chr12_2	2,813	135	4.66%

Supplementary Table 13. Genetic mapping of six loci.

Gene	Chr.	Position
PA1	chr1_1	10.8 - 12.0 Mb
pa1	chr1_2	12.9 - 13.8 Mb
WS1	chr1_2	12.0 - 12.8 Mb
ws1	chr1_1	10.2 - 11.1 Mb
WV1	chr2_2	43.5 - 44.4 Mb
wv1	chr2_1	39.0 - 39.9 Mb
PA2	chr5_2	6.9 - 7.8 Mb
pa2	chr5_1	7.2 - 8.1 Mb
ar1[a]	chr1_1	71.1 - 72.0 Mb
la2[b]	chr5_1	21.9 - 55.8 Mb

[a] *ar1* was cloned in previous study.

[b] *la2* was only mapped on one haplotype by detecting the absent homozygous genotype.

341 **Supplementary Table 14. Primers used in fine mapping of *ws1* and *pa1*.**

342

Primer	Primer sequence (5'-3')	chr1_1 position	chr1_2 position
4482970_F	TGACTGCACAGAAGATTTGA	9,052,971	11,141,678
4482970_R	AGTCCTTCCTTCCTTTCTTG		
4637970_F	GCCAATAGTACGCAACTTGT	9,360,727	11,296,356
4637970_R	GCCATAAAATCTGTCTTTGG		
4912176_F	ACATATTCCTCCGACATCAC	10,040,238	11,902,745
4912176_R	TATAATTTCCACGGAACCAC		
5067755_F	TTGATTGTGCTGGTGATTAG	10,141,891	12,007,961
5067755_R	GACGAGGGTACTGATGTGTT		
5087498_F	ACATTTAGTTTTGCACACCC	10,163,985	12,027,319
5087498_R	GTTGCACTAGTTCTTCCTGG		
5121475_F	TGAAGTAGGCATTGGCTTAT	10,193,019	12,061,229
5121475_R	CAAGGGCCAAACACCAGTTA		
5351542_F	GCCAGAACATAATACTTTCACCT	10,539,603	12,474,357
5351542_R	GATCTAGGCAGAGCCAAATA		
5358404_F	ACCAAAGCTTAGACAAATGG	10,665,003	12,484,293
5358404_R	TGCAAGGTTGAACATTAGGT		
5407435_F	TTCTGGTAGACCATCAGCTC	10,707,770	12,528,529
5407435_R	GACAGCTAAAATGGTCAAGG		
5447005_F	TAGGAACTTCCGAGGTGTTA	10,753,802	12,569,399
5447005_R	TGCACTTTGTGTTGTATTCC		
5475472_F	TTTCTTTGTGTGTGTGTGTG	10,780,947	12,597,474
5475472_R	AGCCATCTAACAAGGAGTGA		
5.536_F	ATTGGACTGCTCTAAATGGA	10,857,355	12,656,378
5.536_R	AGTGACTGACACTCTCCAGG		
5637799_F	TGGAATGTAGCTGTGAAAGA	10,878,527	12,711,979
5637799_R	ACAGAGCATGGATACAAAGC		
5988625_F	GGGTTATGAAGAGGGTTTGT	11,210,117	13,055,261
5988625_R	CCATTTCCTTCAATCTCTCCC		

Primer	Primer sequence (5'-3')	chr1_1 position	chr1_2 position
6016264_F	GAACGAACATTGAAGGACTC	11,348,914	13,191,234
6016264_R	TGGCAAGGAAAAGTATGTCT		
6070946_F	TGCAAGGGAAGTCCTATAAA	11,821,758	13,249,054
6070946_R	ACTCGAGCCCTTCTCTAGTC		
6145074_F	GGCCATAAAGAAAGTGAAGCA	11,975,547	13,315,064
6145074_R	CTTTTAGTTGAGCTCCATGTC		
6190159_F	GTGCCGTTTGAAATTGCCAT	12,012,928	13,357,412
6190159_R	ACTGGGGTAAATACAAGAAGCA		
6.29_F	ATAGCCGGTACTTTTTGATG	12,148,366	13,438,791
6.29_R	GAAATATGCAGGGTTTTCAG		
6.462_F	CATTCTTTTTCCAGCTTCAC	12,182,789	13,475,649
6.462_R	TGAAGTGTACCCATAGGCTT		

REFERENCES

1. Ruan, J. & Li, H. Fast and accurate long-read assembly with wtdbg2. *Nature Methods* **17**, 155-158 (2020).
2. Koren, S. *et al.* Canu: scalable and accurate long-read assembly via adaptive k-mer weighting and repeat separation. *Genome Res.* **27**, 722-736 (2017).
3. Kolmogorov, M., Yuan, J., Lin, Y. & Pevzner, P.A. Assembly of long, error-prone reads using repeat graphs. *Nat Biotechnol* **37**, 540-546 (2019).
4. Chaisson, M.J. & Tesler, G. Mapping single molecule sequencing reads using basic local alignment with successive refinement (BLASR): application and theory. *BMC Bioinformatics* **13**, 238 (2012).
5. Chaisson, M.J. & Tesler, G. Mapping single molecule sequencing reads using basic local alignment with successive refinement (BLASR). *BMC Bioinformatics* **13**(2012).
6. Dudchenko, O. *et al. De novo* assembly of the *Aedes aegypti* genome using Hi-C yields chromosome-length scaffolds. *Science* **356**, 92-95 (2017).
7. Zhang, X., Zhang, S., Zhao, Q., Ming, R. & Tang, H. Assembly of allele-aware, chromosomal-scale autopolyploid genomes based on Hi-C data. *Nature Plants* **5**, 833-845 (2019).
8. Kurtz, S. *et al.* Versatile and open software for comparing large genomes. *Genome Biology* **5**, R12 (2004).
9. Li, H. Aligning sequence reads, clone sequences and assembly contigs with BWA-MEM. *arXiv:1303.3997v2* (2013).
10. Simao, F.A., Waterhouse, R.M., Ioannidis, P., Kriventseva, E.V. & Zdobnov, E.M. BUSCO: assessing genome assembly and annotation completeness with single-copy orthologs. *Bioinformatics* **31**, 3210-2 (2015).
11. Potato Genome Sequencing Consortium. Genome sequence and analysis of the tuber crop potato. *Nature* **475**, 189-95 (2011).
12. Aversano, R. *et al.* The *Solanum commersonii g*enome sequence provides insights into adaptation to stress conditions and genome evolution of wild potato relatives. *Plant Cell* **27**, 954-68 (2015).
13. Leisner, C.P. *et al.* Genome sequence of M6, a diploid inbred clone of the high-glycoalkaloid-producing tuber-bearing potato species *Solanum*

chacoense, reveals residual heterozygosity. *The Plant Journal* **94**, 562-570 (2017).
