# Supplementary material for: Haplotype-resolved genome analyses of a heterozygous diploid potato
Source: Nat Genet. 2020 Sep 28;52(10):1018–23. doi: 10.1038/s41588-020-0699-x (PMC7527274; doi:10.1038/s41588-020-0699-x)
Supplement: Supplementary file 2 — Reporting Summary [file 41588_2020_699_MOESM2_ESM.pdf]

nature research

Corresponding author(s): Sanwen Huang

Last updated by author(s): Jul 30, 2020

Reporting Summary

Nature Research wishes to improve the reproducibility of the work that we publish. This form provides structure for consistency and transparency in reporting. For further information on Nature Research policies, see our Editorial Policies and the Editorial Policy Checklist.

Statistics

For all statistical analyses, confirm that the following items are present in the figure legend, table legend, main text, or Methods section.

n/a	Confirmed	
☐	☒	The exact sample size (*n*) for each experimental group/condition, given as a discrete number and unit of measurement
☐	☒	A statement on whether measurements were taken from distinct samples or whether the same sample was measured repeatedly
☐	☒	The statistical test(s) used AND whether they are one- or two-sided<br>*Only common tests should be described solely by name; describe more complex techniques in the Methods section.*
☒	☐	A description of all covariates tested
☒	☐	A description of any assumptions or corrections, such as tests of normality and adjustment for multiple comparisons
☒	☐	A full description of the statistical parameters including central tendency (e.g. means) or other basic estimates (e.g. regression coefficient) AND variation (e.g. standard deviation) or associated estimates of uncertainty (e.g. confidence intervals)
☐	☒	For null hypothesis testing, the test statistic (e.g. *F*, *t*, *r*) with confidence intervals, effect sizes, degrees of freedom and *P* value noted<br>*Give P values as exact values whenever suitable.*
☒	☐	For Bayesian analysis, information on the choice of priors and Markov chain Monte Carlo settings
☒	☐	For hierarchical and complex designs, identification of the appropriate level for tests and full reporting of outcomes
☒	☐	Estimates of effect sizes (e.g. Cohen's *d*, Pearson's *r*), indicating how they were calculated

Our web collection on statistics for biologists contains articles on many of the points above.

Software and code

Policy information about availability of computer code

Data collection	No software was used to collect data.
Data analysis	All of softwares used in this study have been fully described including specifying versions in the Methods. The published tools have been cited and the open source has been described with the website links. The custom pipelines and scripts used in the project are deposited in GitHub https://github.com/zhouqiansolab/Haplotype-resolved-potato-genome.

For manuscripts utilizing custom algorithms or software that are central to the research but not yet described in published literature, software must be made available to editors and reviewers. We strongly encourage code deposition in a community repository (e.g. GitHub). See the Nature Research guidelines for submitting code & software for further information.

Data

Policy information about availability of data

All manuscripts must include a data availability statement. This statement should provide the following information, where applicable:

- Accession codes, unique identifiers, or web links for publicly available datasets
- A list of figures that have associated raw data
- A description of any restrictions on data availability

The genome assembly, annotation and a genome browser are available at SpudDB (http://solanaceae.plantbiology.msu.edu). This Whole Genome Shotgun project has been deposited at GenBank under the accession JACDXL000000000 and JACDXM000000000. The raw sequencing data has been deposited in the NCBI Sequence Read Archive (https://www.ncbi.nlm.nih.gov/sra), with BioProject accession number PRJNA573826. The data has also been submitted to the Chinese National Genomics Data Center (https://bigd.big.ac.cn), with accession number CRA002005.

Field-specific reporting

Please select the one below that is the best fit for your research. If you are not sure, read the appropriate sections before making your selection.

☒ Life sciences ☐ Behavioural & social sciences ☐ Ecological, evolutionary & environmental sciences

For a reference copy of the document with all sections, see nature.com/documents/nr-reporting-summary-flat.pdf

Life sciences study design

All studies must disclose on these points even when the disclosure is negative.

Sample size	For genome assembly and comparative analysis of haplotypes, a single sample of RH is enough. For genetic groups construction, we applied 880 selfed progeny and for fine mapping of genes. To locate the candidate genes for PA1 and WS1, we applied another 1200 progeny, which provides sufficient segregations in genetic mapping.
Data exclusions	No data was excluded.
Replication	For transcriptome sequencing, methylome sequencing and RT-qPCR analysis, the analyses were conducted on three replicates for each tissue.
Randomization	The sequencing samples of RH were collected randomly from the cultured seedlings and plants grown in green house.
Blinding	A blinded-experiment is not needed in this study because there is no comparing analysis between different groups.

Reporting for specific materials, systems and methods

We require information from authors about some types of materials, experimental systems and methods used in many studies. Here, indicate whether each material, system or method listed is relevant to your study. If you are not sure if a list item applies to your research, read the appropriate section before selecting a response.

Materials & experimental systems

n/a	Involved in the study	
☒	☐	Antibodies
☒	☐	Eukaryotic cell lines
☒	☐	Palaeontology and archaeology
☒	☐	Animals and other organisms
☒	☐	Human research participants
☒	☐	Clinical data
☒	☐	Dual use research of concern

Methods

n/a	Involved in the study	
☒	☐	ChIP-seq
☒	☐	Flow cytometry
☒	☐	MRI-based neuroimaging
