# Supplementary material for: Haplotype-resolved genome analyses of a heterozygous diploid potato
Source: Nat Genet. 2020 Sep 28;52(10):1018–23. doi: 10.1038/s41588-020-0699-x (PMC7527274; doi:10.1038/s41588-020-0699-x)
Supplement: Supplementary file 3 — Graphs showing the alignment of BACs (y axis) and RHgv1 scaffolds (x axis). [file 41588_2020_699_MOESM3_ESM.pdf]

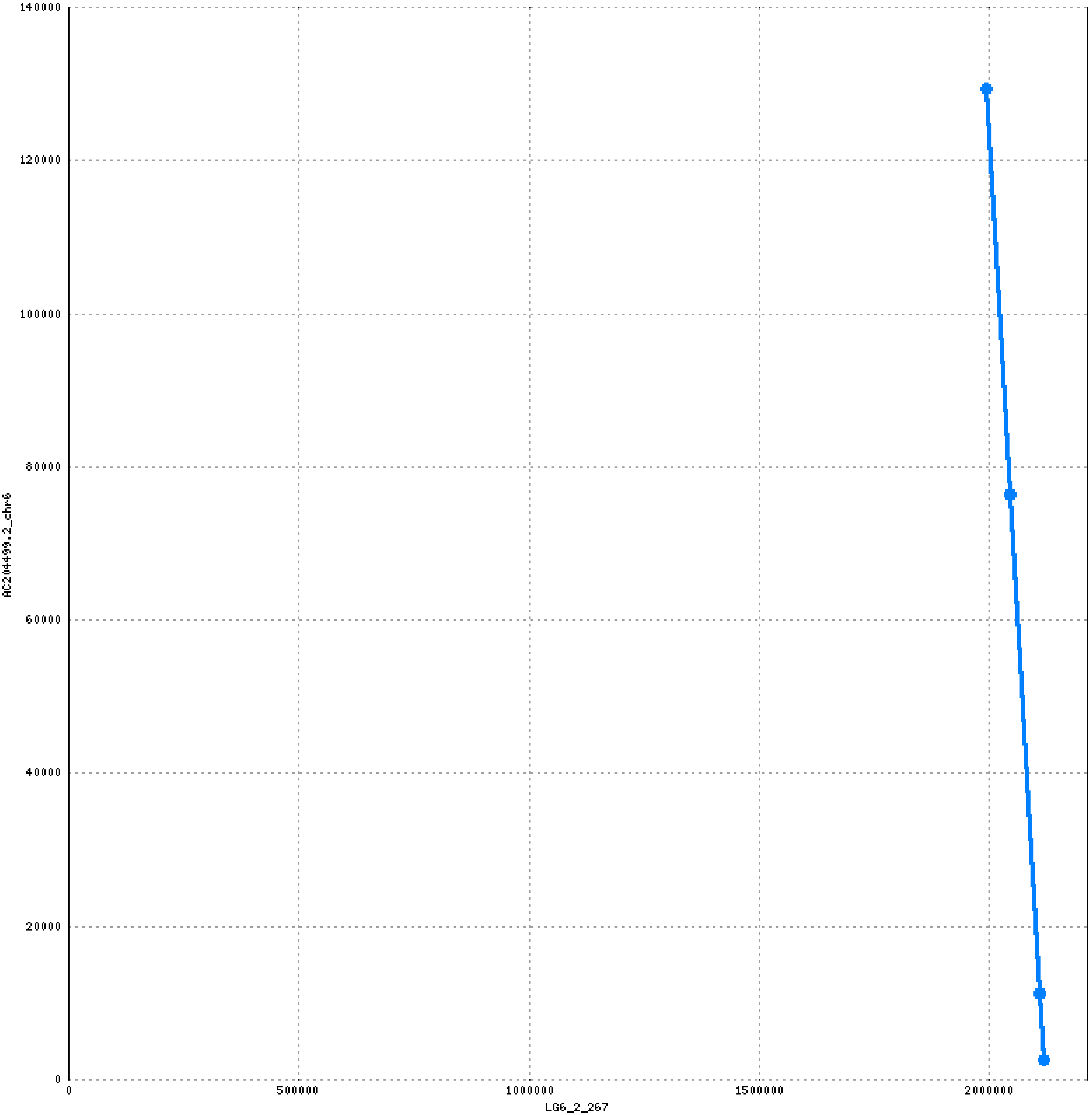

140000
120000
100000
80000
60000
40000
20000
0
AC204499.2_chr6
0
500000
1000000
1500000
2000000
LG6_2_267

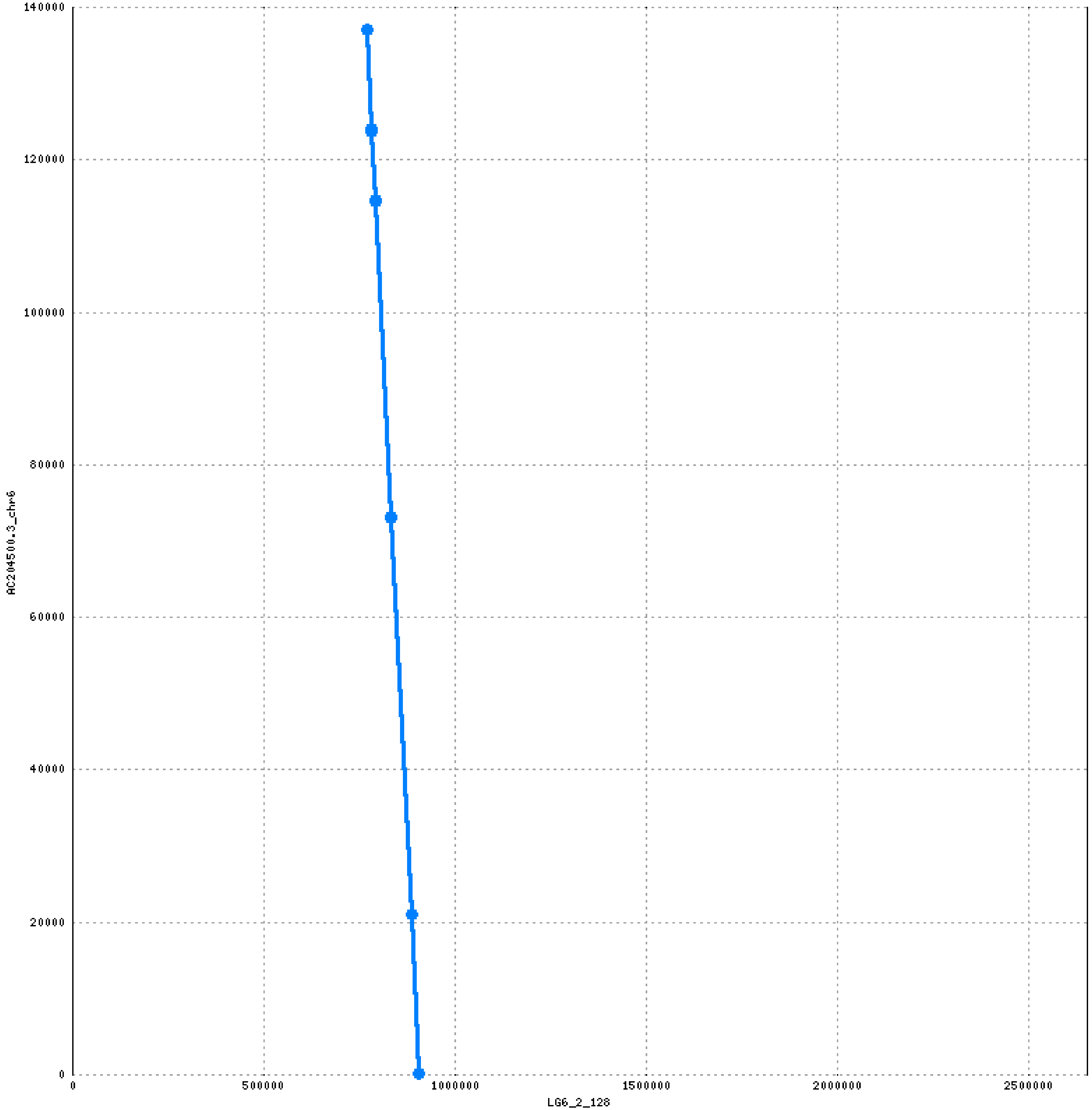

140000
120000
100000
80000
60000
40000
20000
0
AC204500.3_chr6
0
500000
1000000
1500000
2000000
2500000
LG6_2_128

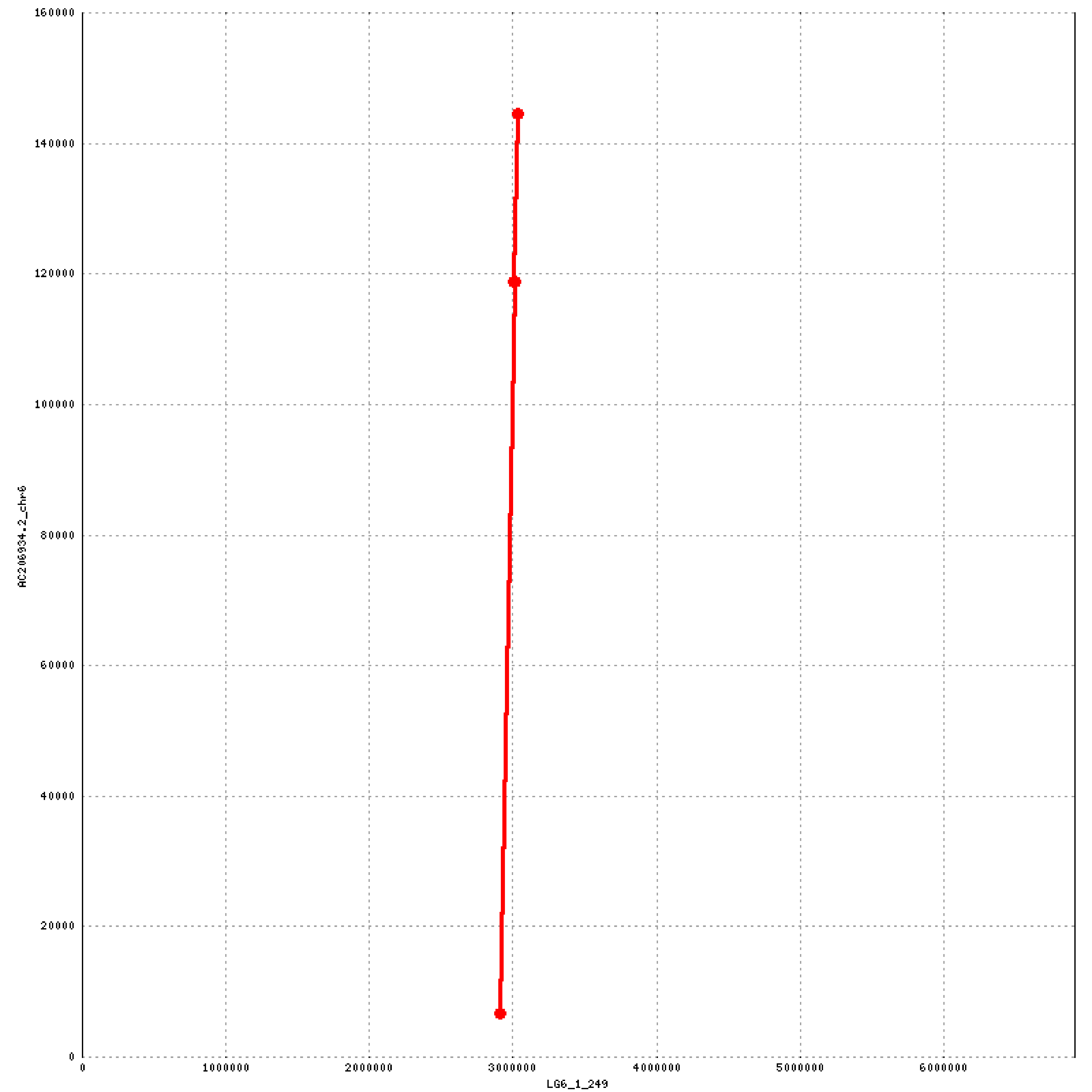

160000
140000
120000
100000
80000
60000
40000
20000
0
AC206934.2_chr6
0
1000000
2000000
3000000
4000000
5000000
6000000
LG6_1_249

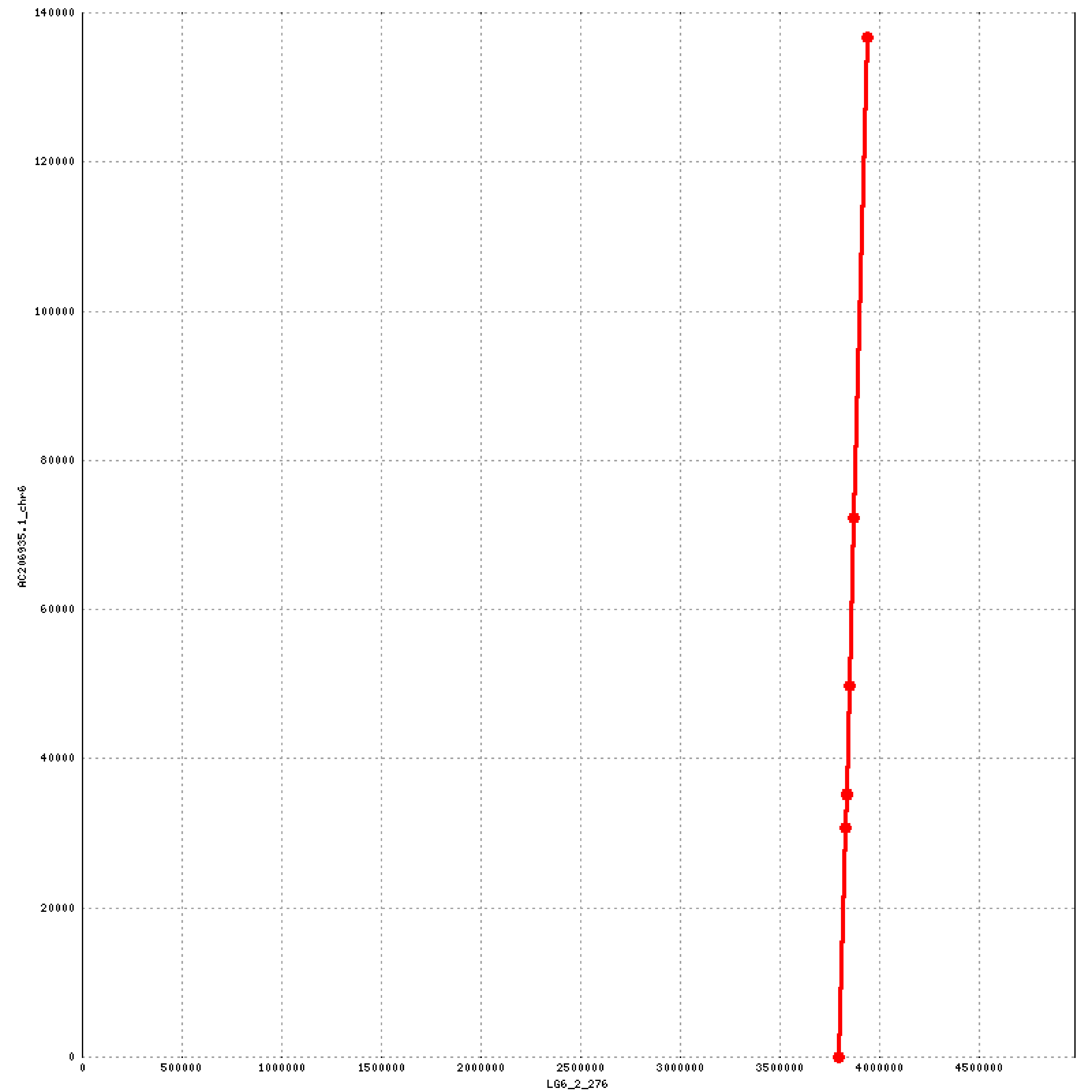

140000
120000
100000
80000
60000
40000
20000
0
AC206935.1_chr6
0
500000
1000000
1500000
2000000
2500000
3000000
3500000
4000000
4500000
LG6_2_276

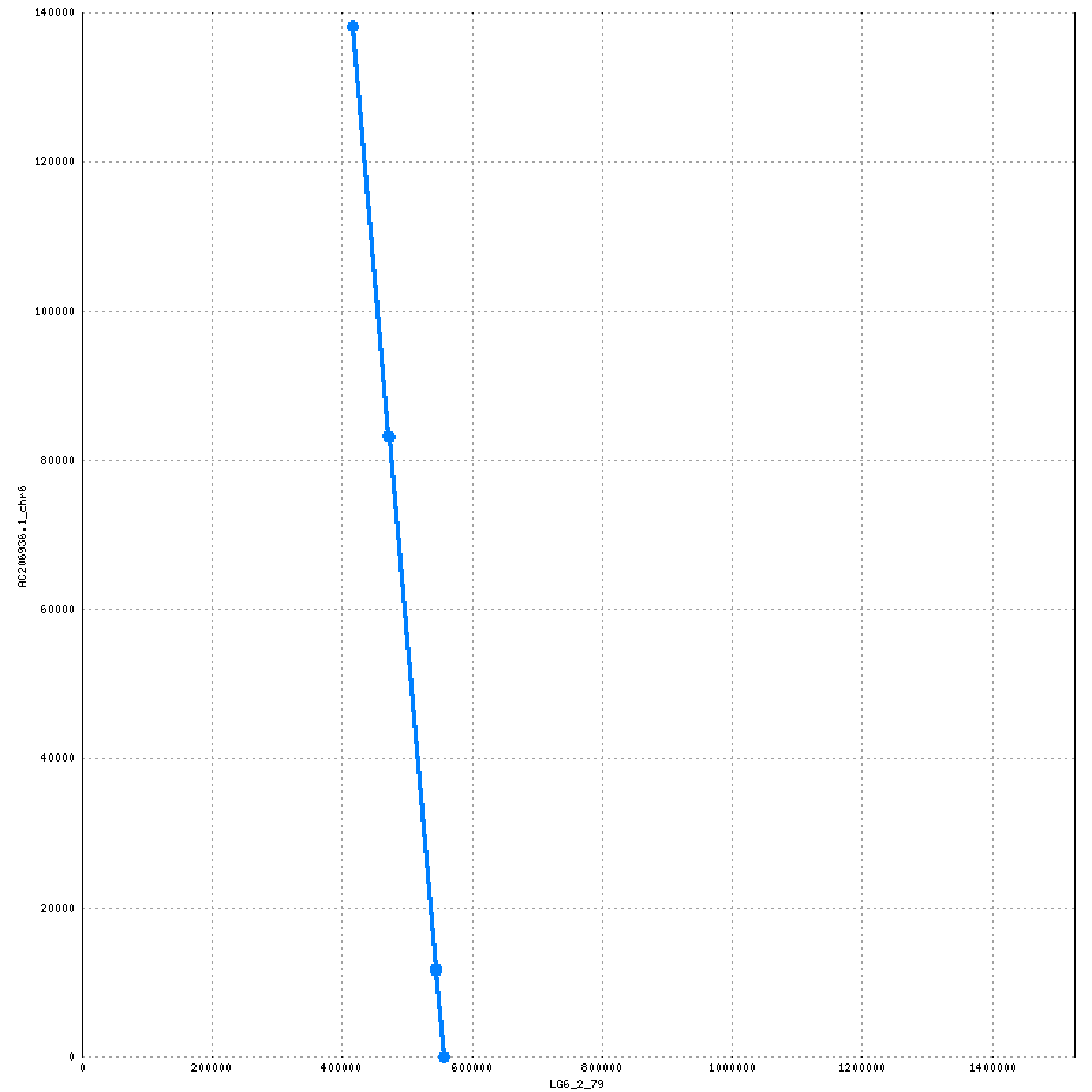
140000
120000
100000
80000
60000
40000
20000
0
AC206936.1_chr6
0
200000
400000
600000
800000
1000000
1200000
1400000
LG6_2_79

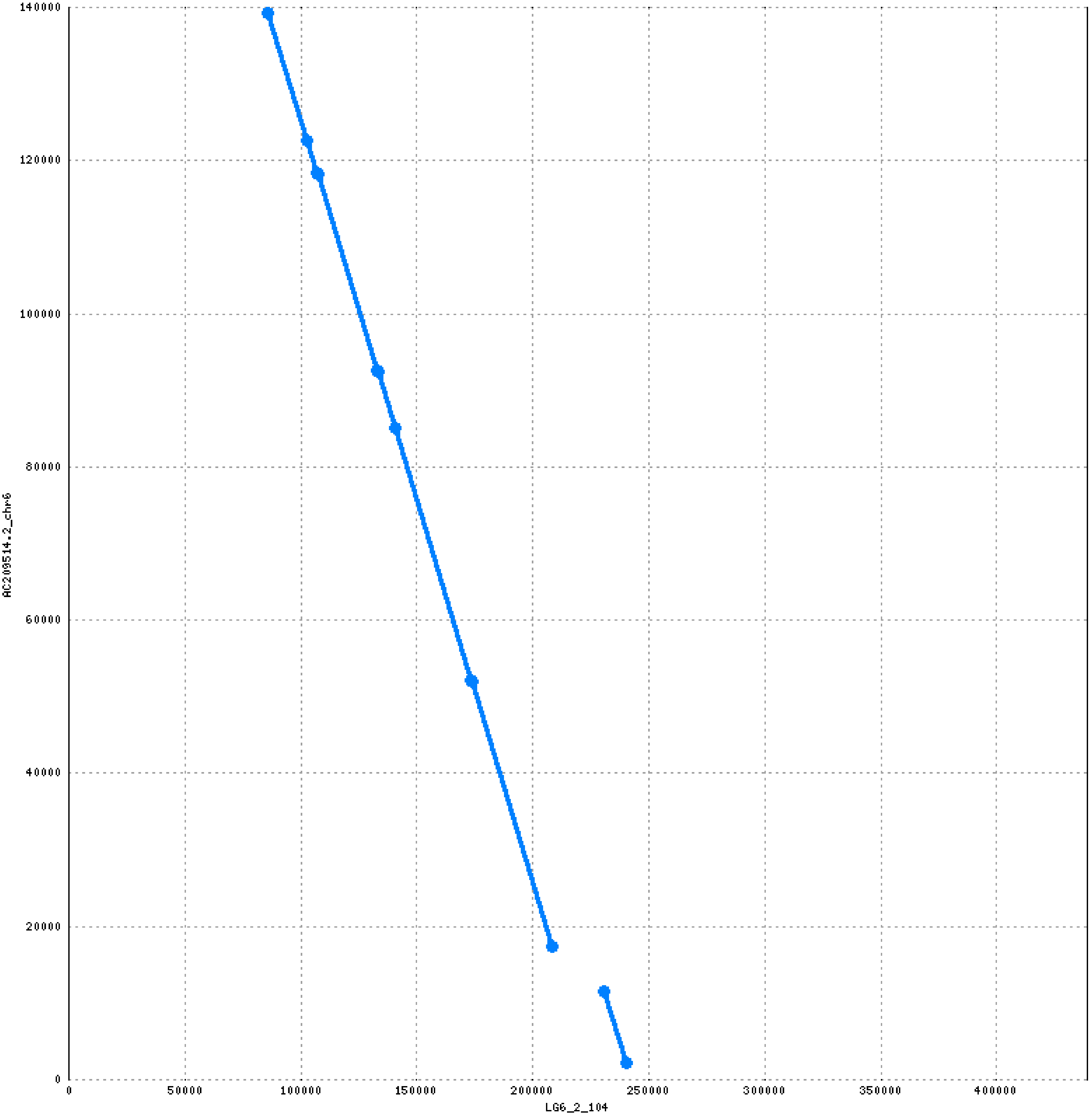

140000
120000
100000
80000
60000
40000
20000
0
AC209514.2_chr6
0
50000
100000
150000
200000
250000
300000
350000
400000
LG6_2_104

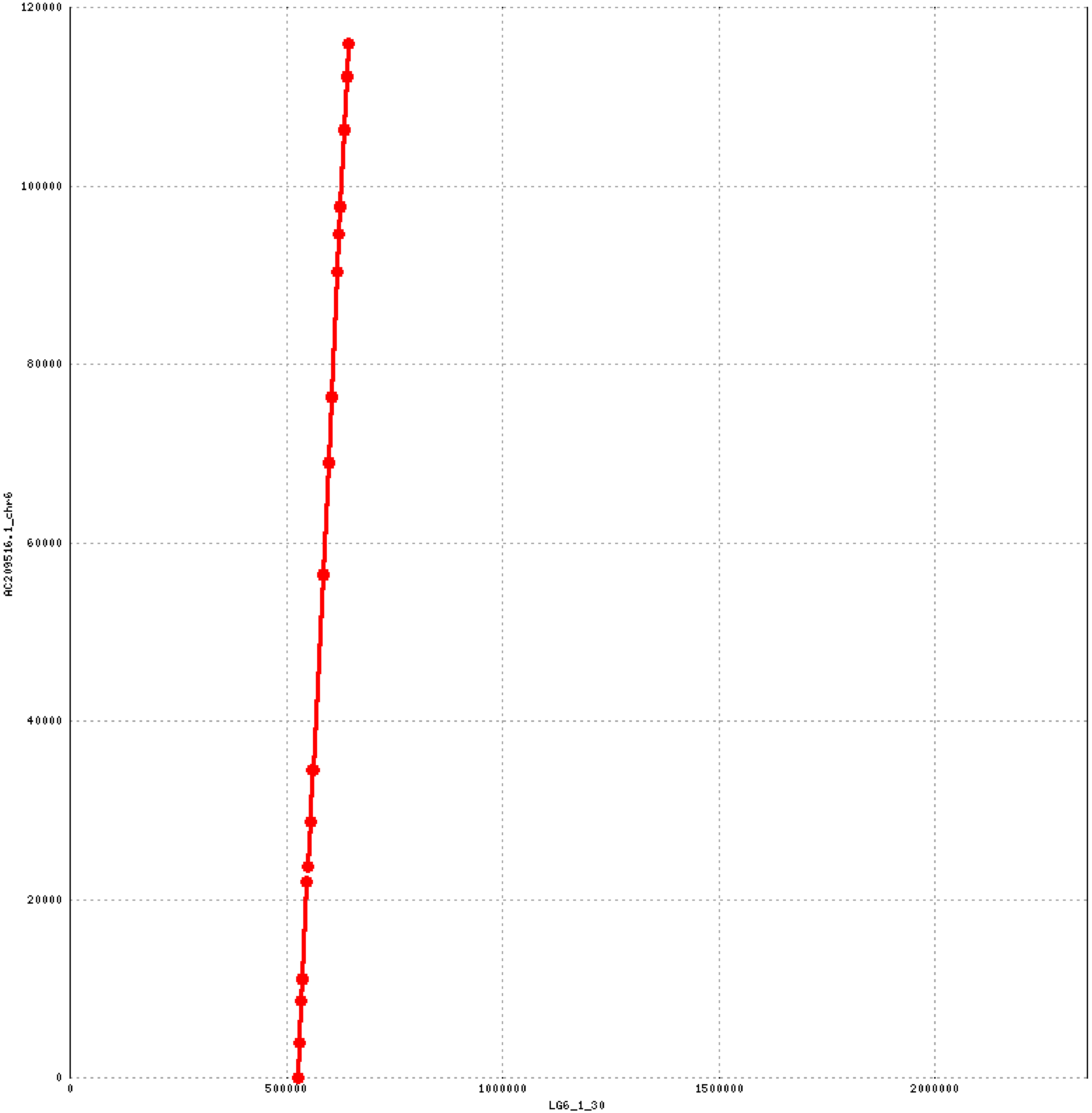

120000
100000
80000
60000
40000
20000
0
AC209516.1_chr6
0
500000
1000000
1500000
2000000
LG6_1_30

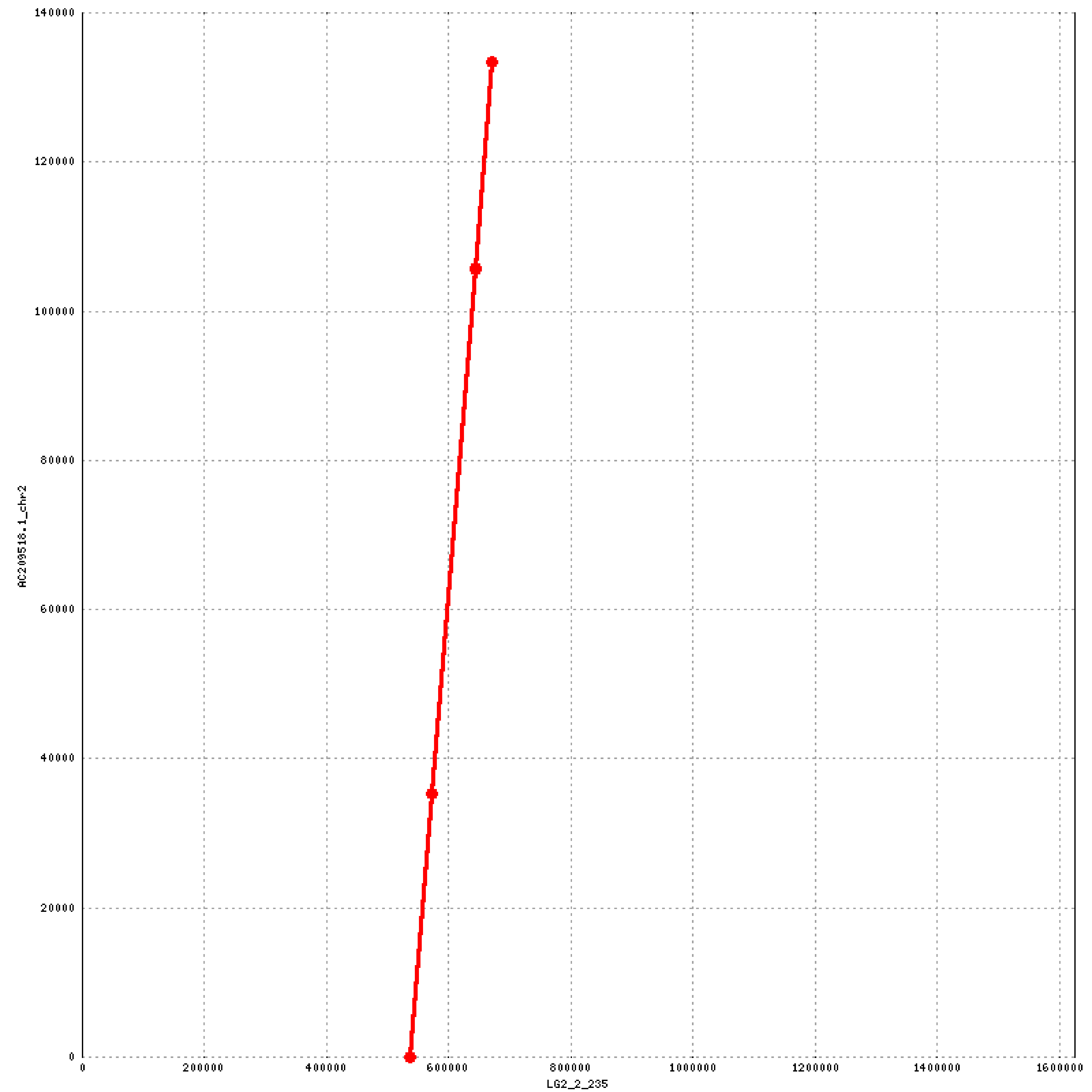

140000
120000
100000
80000
60000
40000
20000
0
AC209518.1_chr2
0
200000
400000
600000
800000
1000000
1200000
1400000
1600000
LG2_2_235

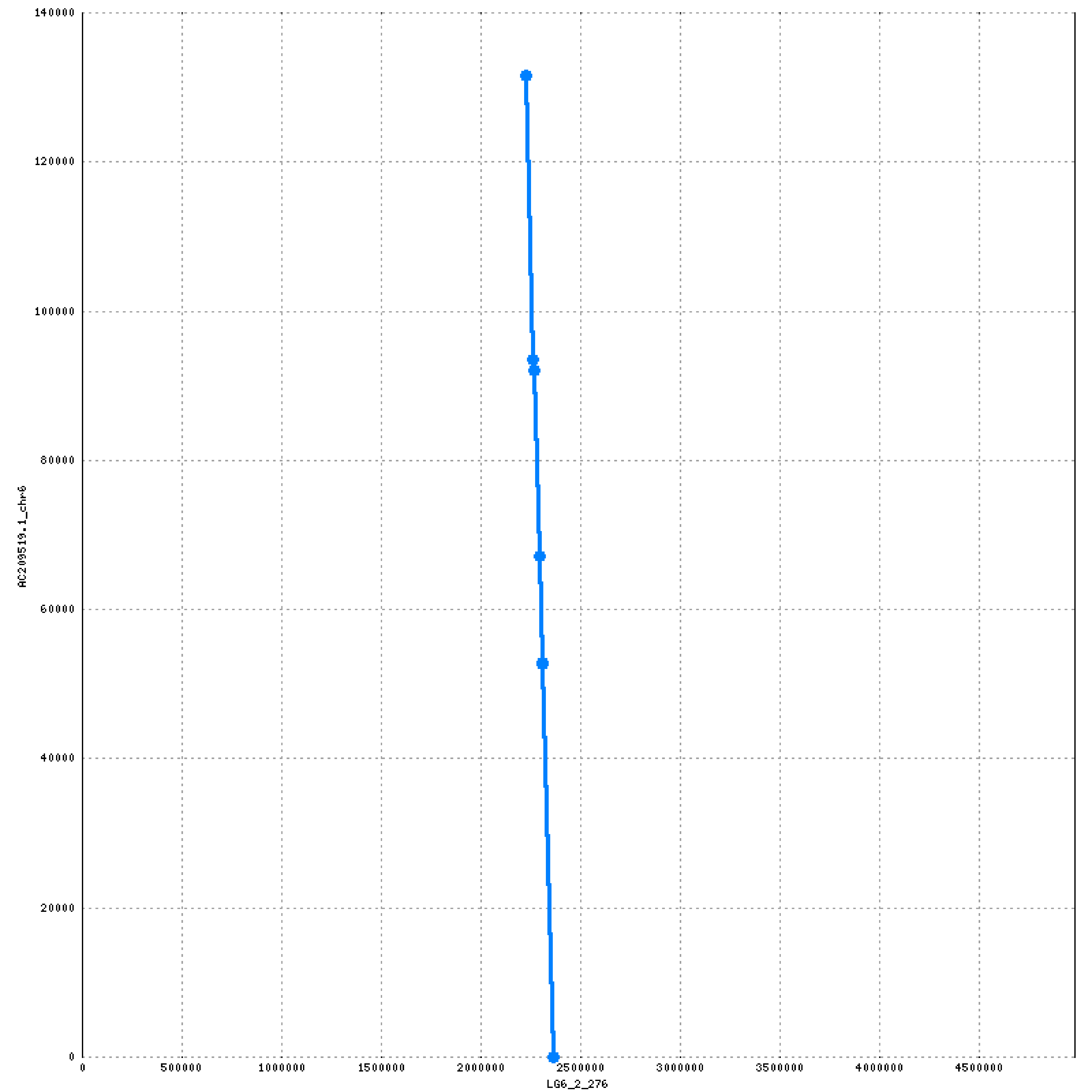

140000
120000
100000
80000
60000
40000
20000
0
AC209519.1_chr6
0
500000
1000000
1500000
2000000
2500000
3000000
3500000
4000000
4500000
LG6_2_276

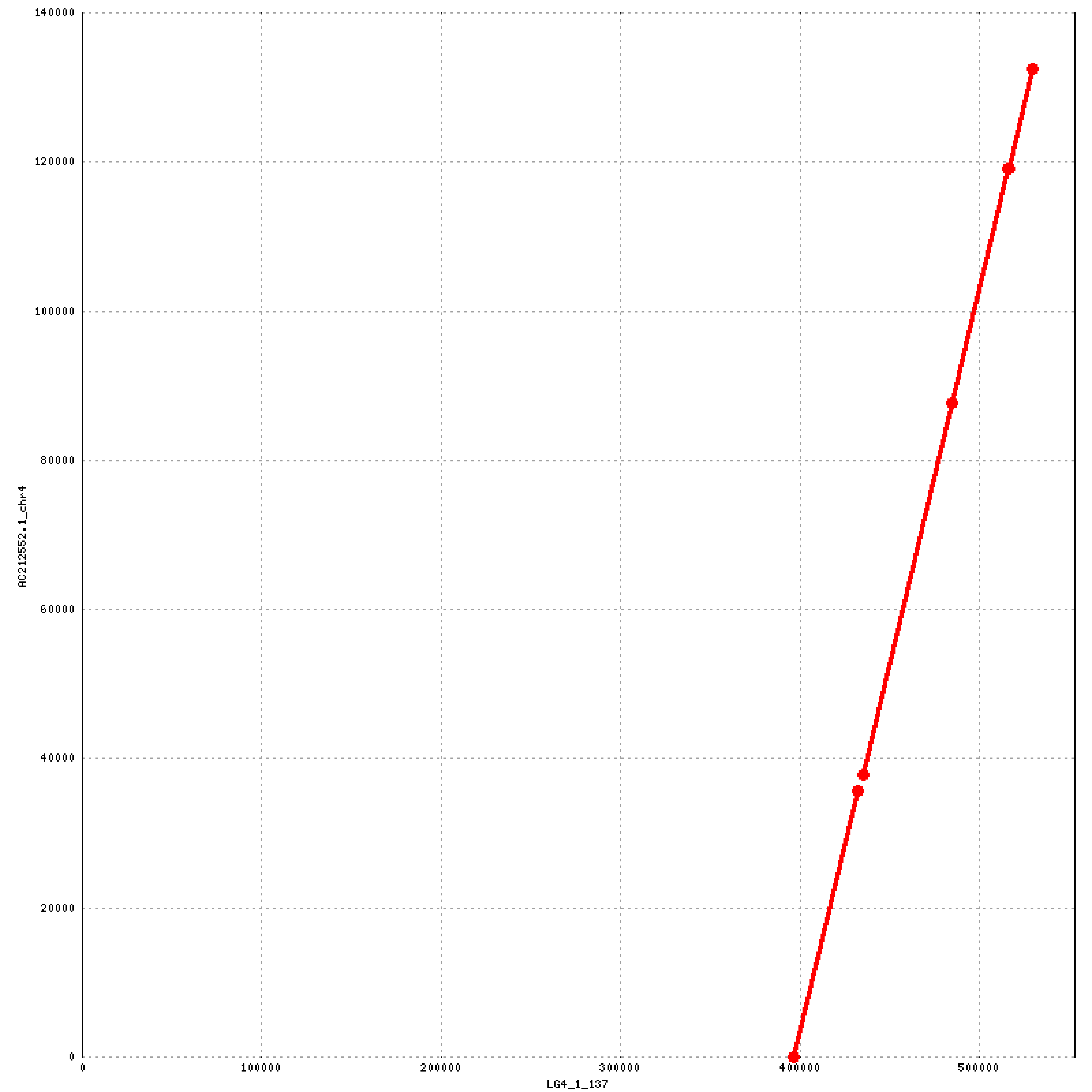

140000
120000
100000
80000
60000
40000
20000
0
AC212552.1_chr4
0
100000
200000
300000
400000
500000
LG4_1_137

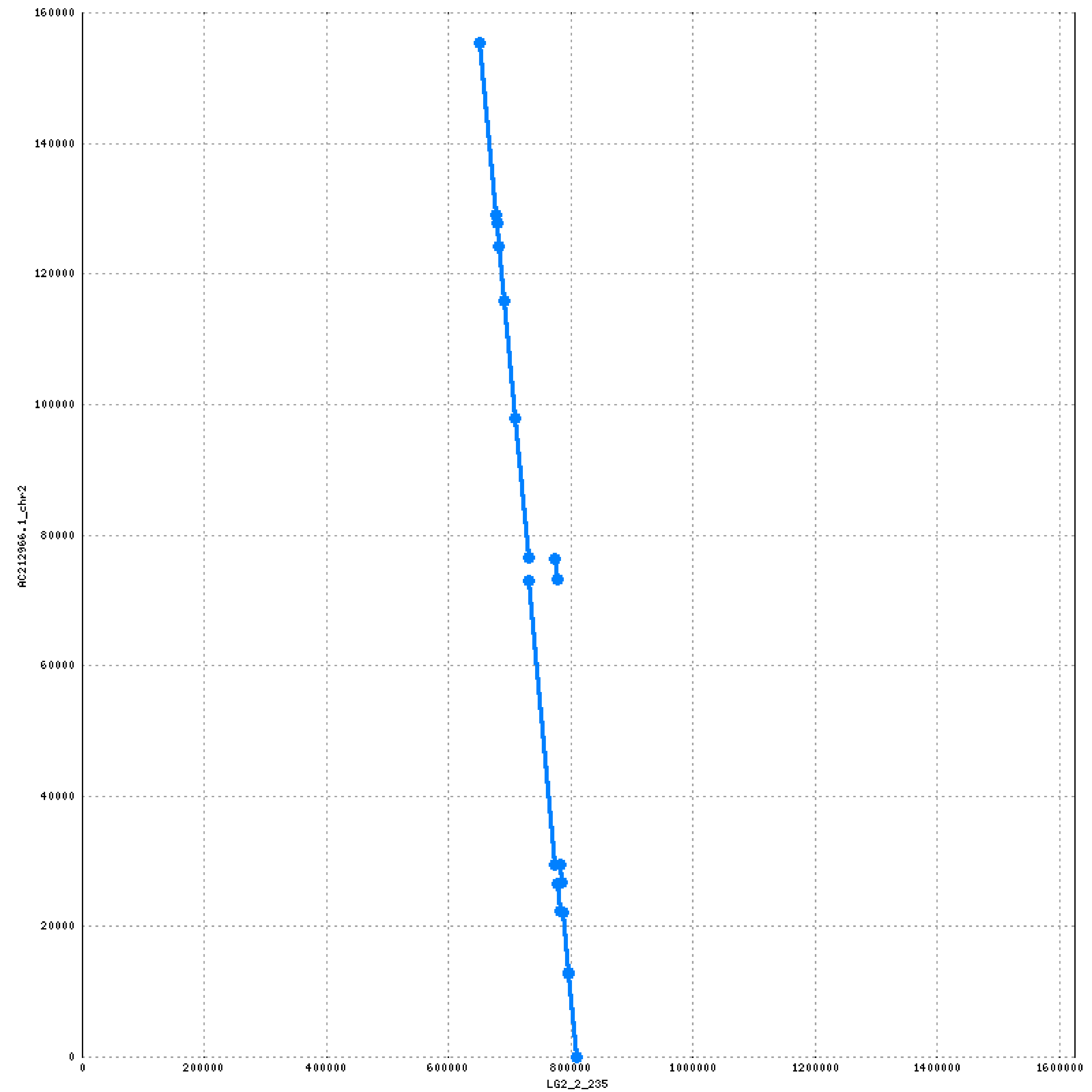

160000
140000
120000
100000
80000
60000
40000
20000
0
AC212966.1_chr2
0
200000
400000
600000
800000
1000000
1200000
1400000
1600000
LG2_2_235

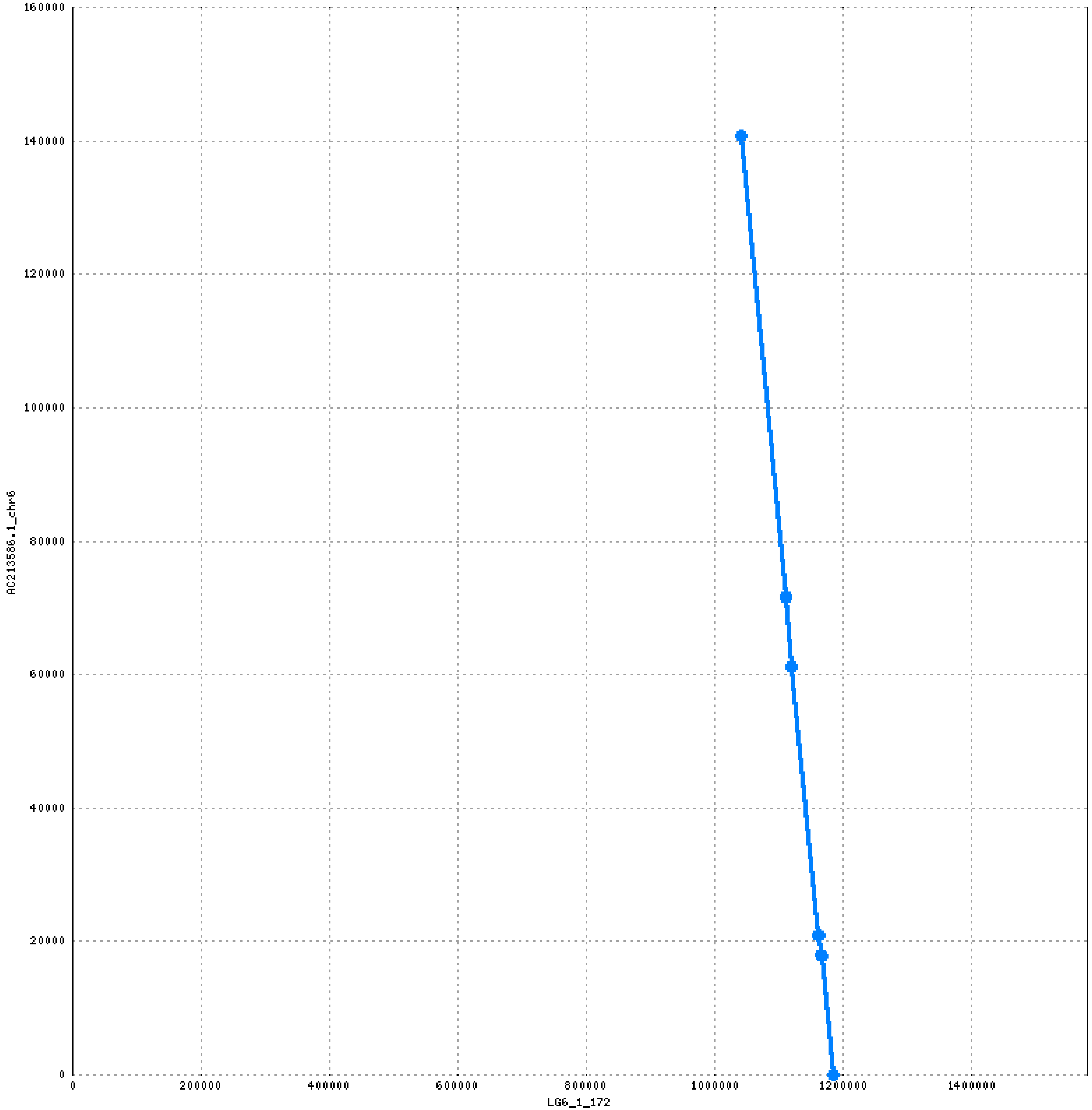

160000
140000
120000
100000
80000
60000
40000
20000
0
AC213586.1_chr6
0
200000
400000
600000
800000
1000000
1200000
1400000
LG6_1_172

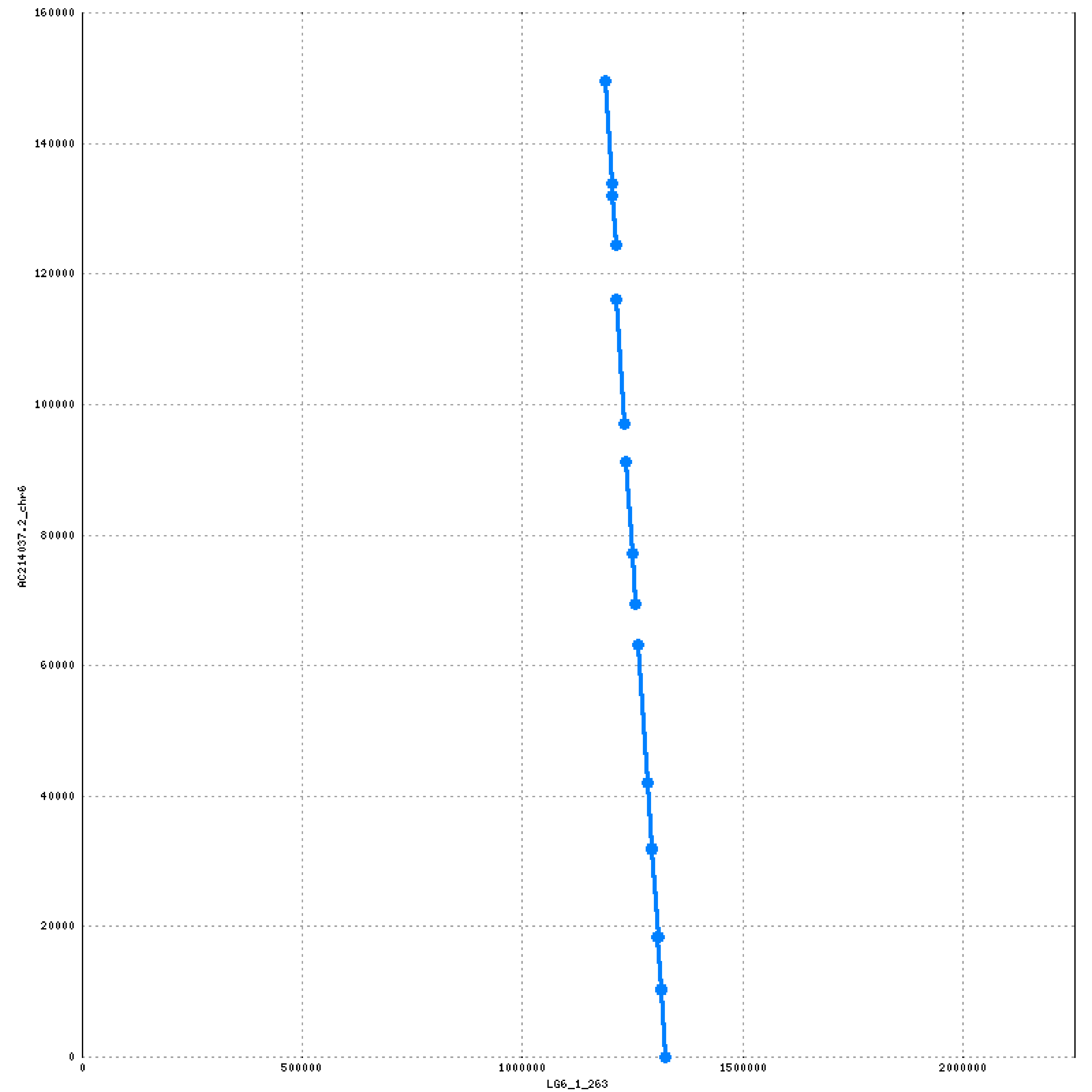
160000
140000
120000
100000
80000
60000
40000
20000
0
AC214037.2_chr6
0
500000
1000000
1500000
2000000
LG6_1_263

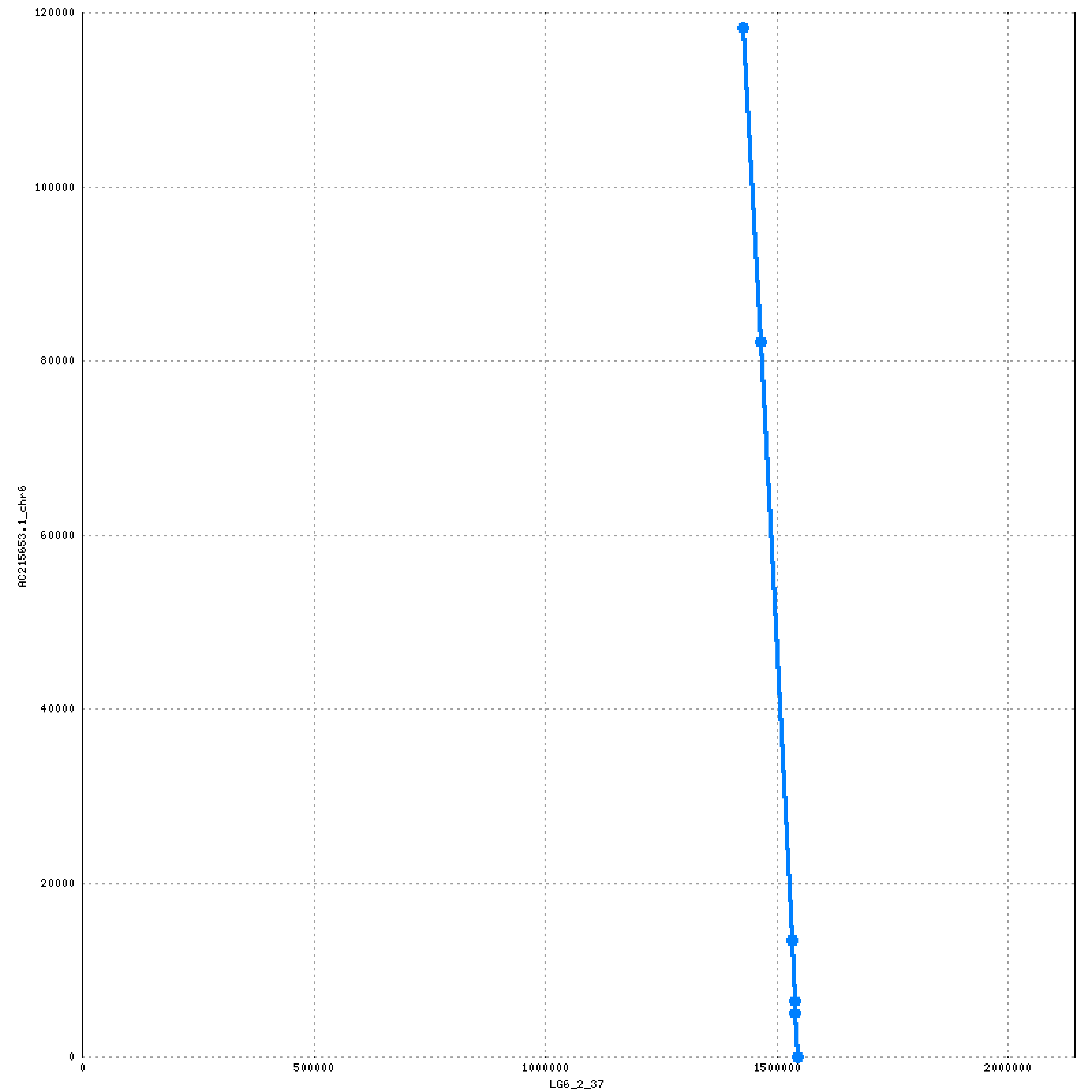

120000
100000
80000
60000
40000
20000
0
AC215653.1_chr6
0
500000
1000000
1500000
2000000
LG6_2_37

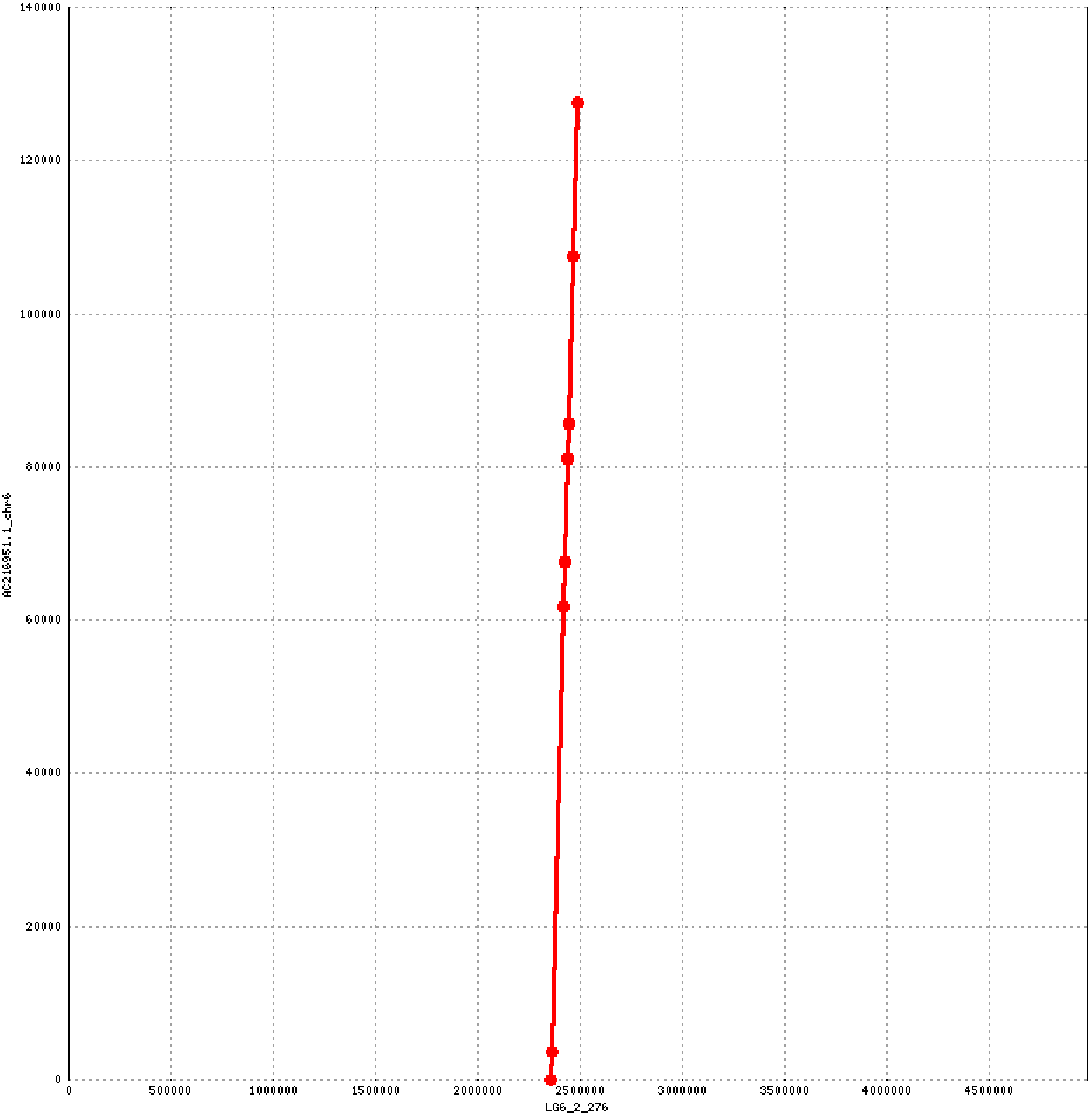

140000
120000
100000
80000
60000
40000
20000
0
AC216851.1_chr6
0
500000
1000000
1500000
2000000
2500000
3000000
3500000
4000000
4500000
LG6_2_276

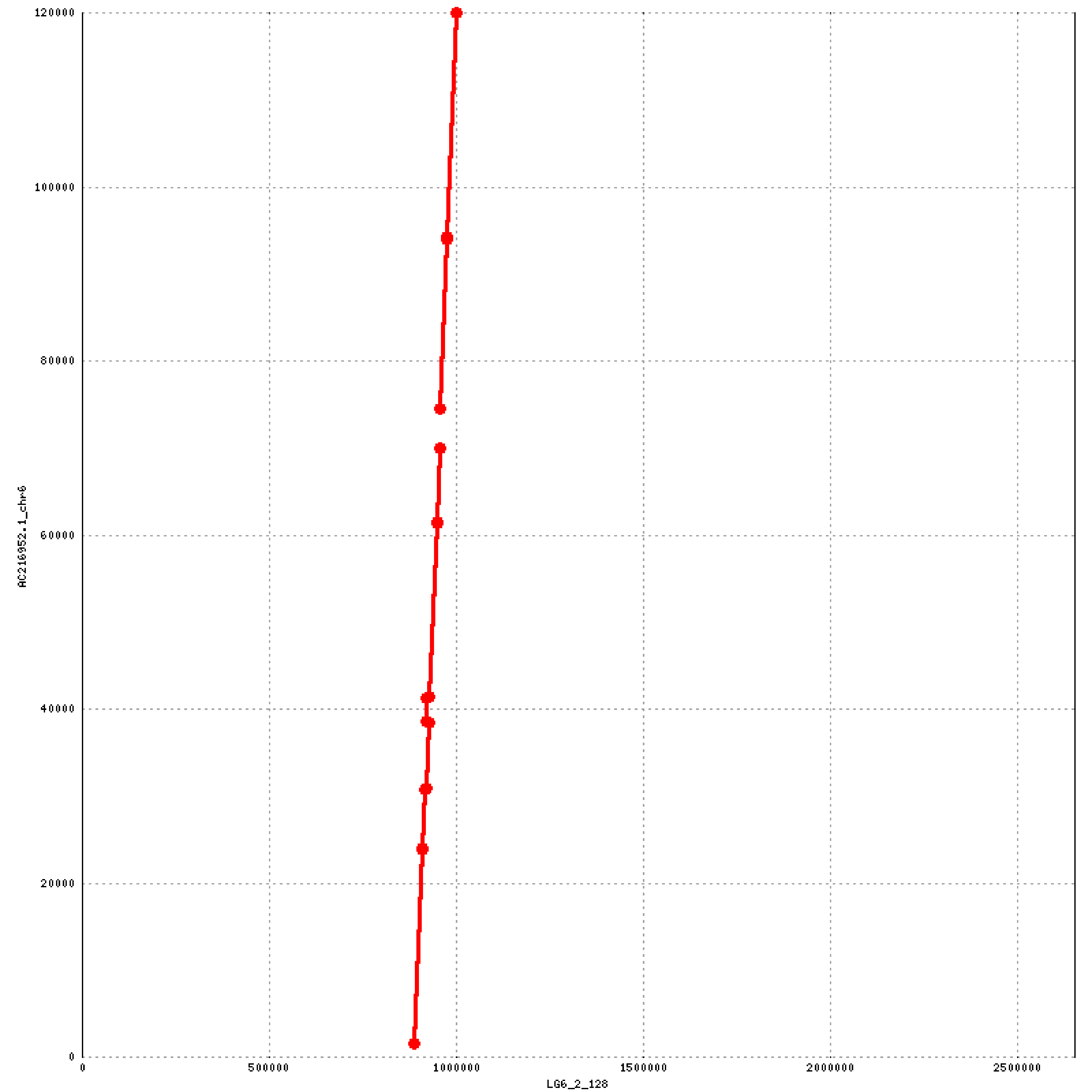

120000
100000
80000
60000
40000
20000
0
AC216952.1_chr6
0
500000
1000000
1500000
2000000
2500000
LG6_2_128

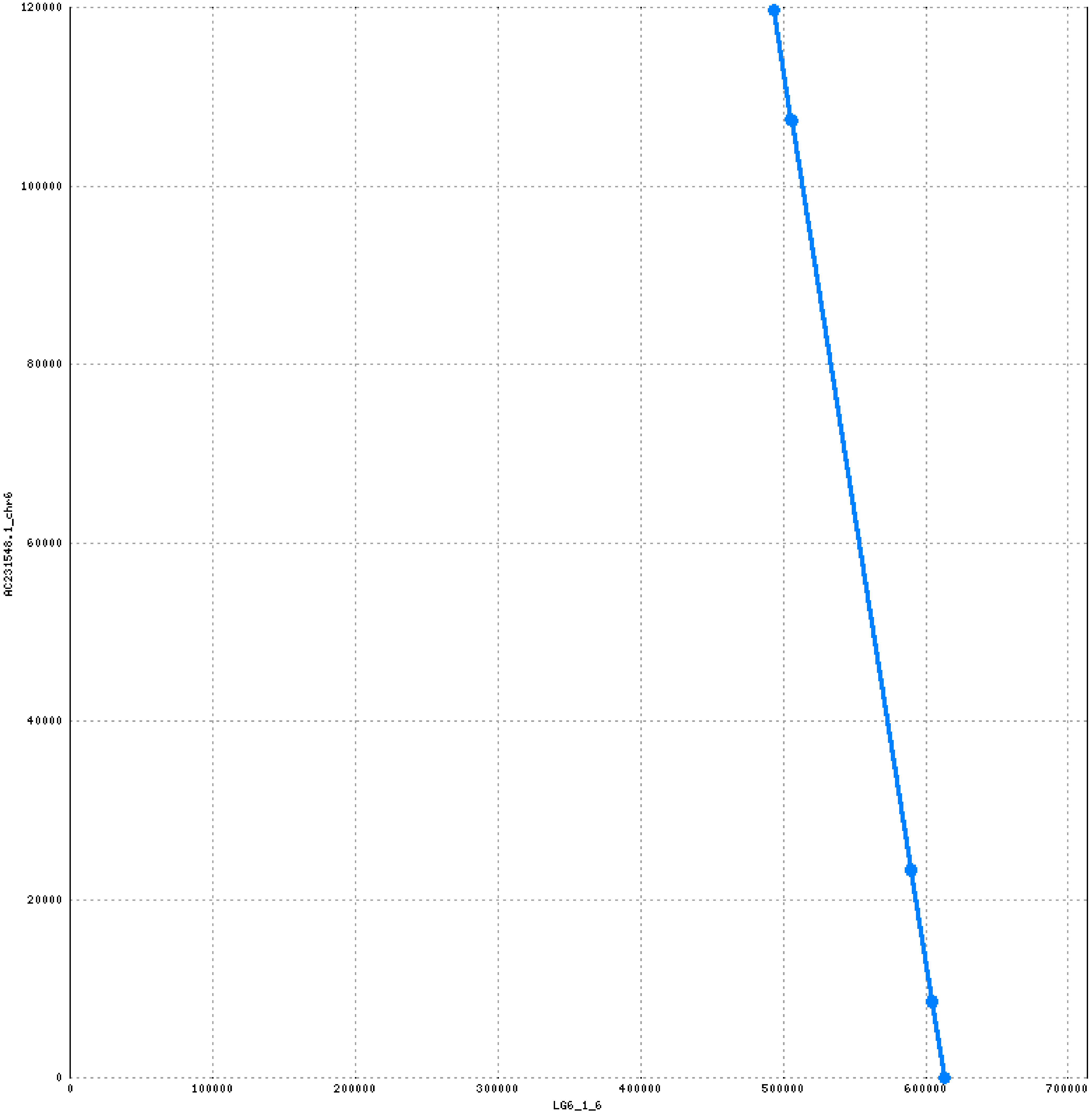

120000
100000
80000
60000
40000
20000
0
AC231548.1_chr6
0
100000
200000
300000
400000
500000
600000
700000
LG6_1_6

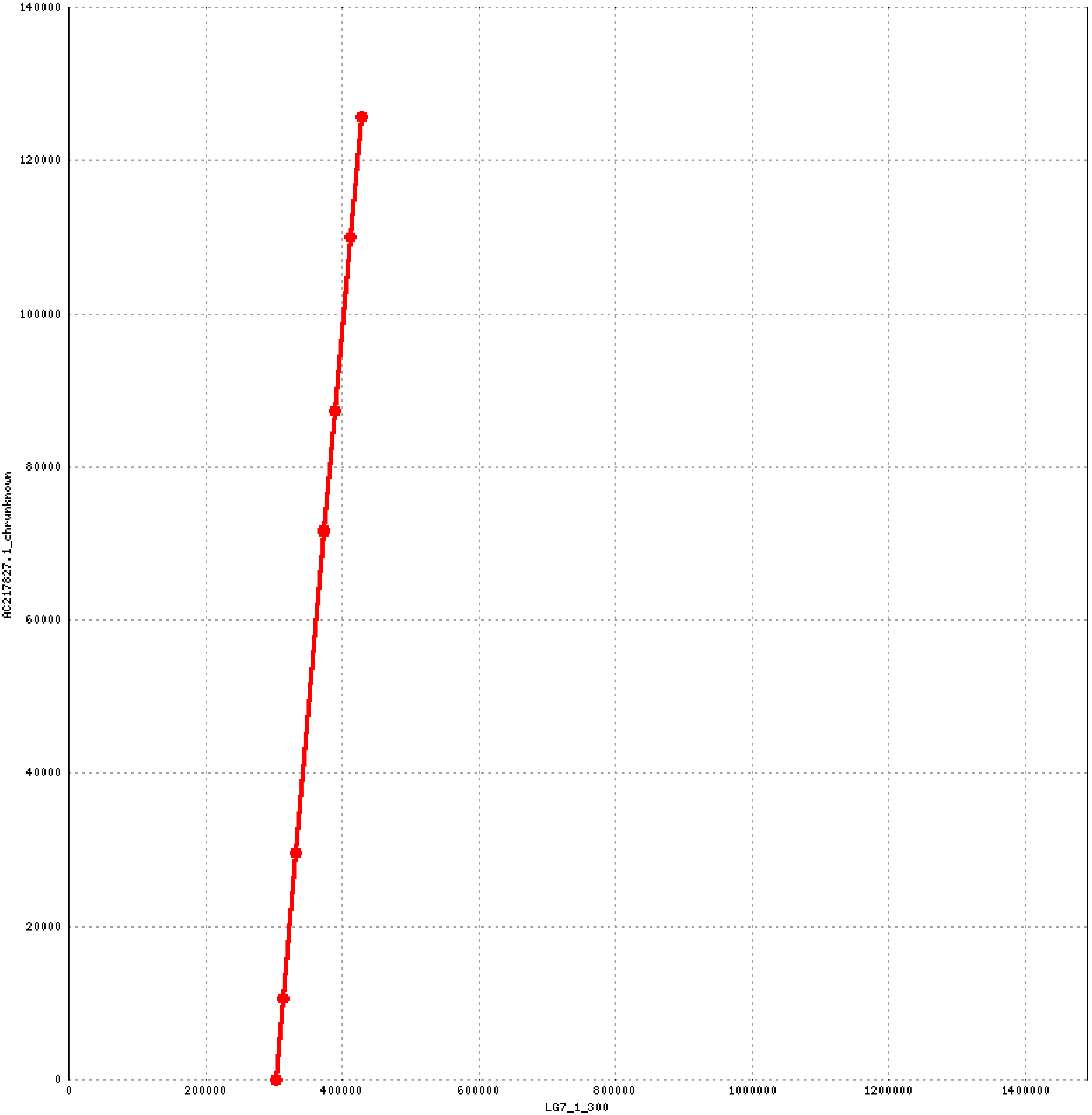

140000
120000
100000
80000
60000
40000
20000
0
AC217827.1_chrunknown
0
200000
400000
600000
800000
1000000
1200000
1400000
LG7_1_300

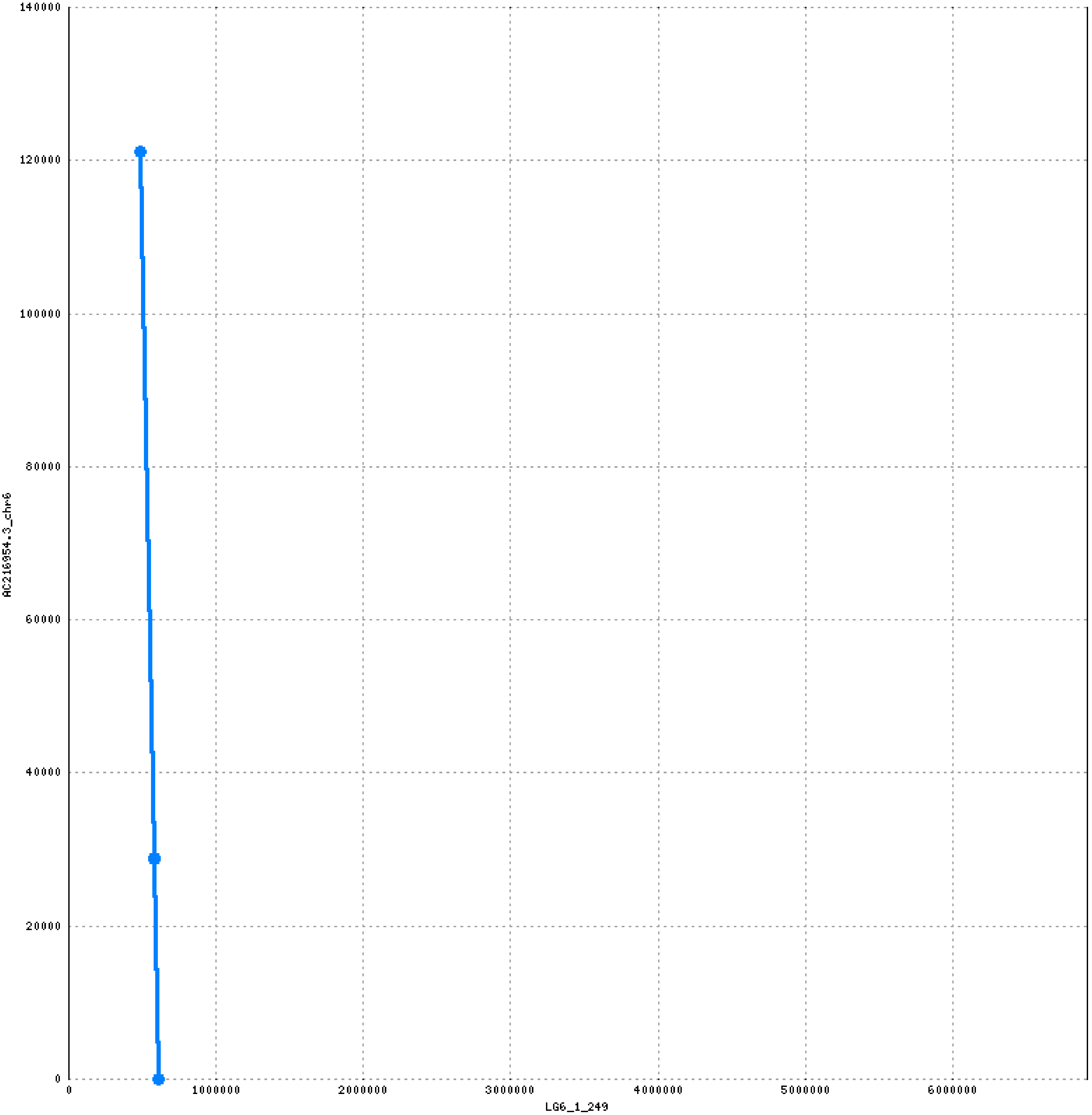

140000
120000
100000
80000
60000
40000
20000
0
AC216954.3_chr6
0
1000000
2000000
3000000
4000000
5000000
6000000
LG6_1_249

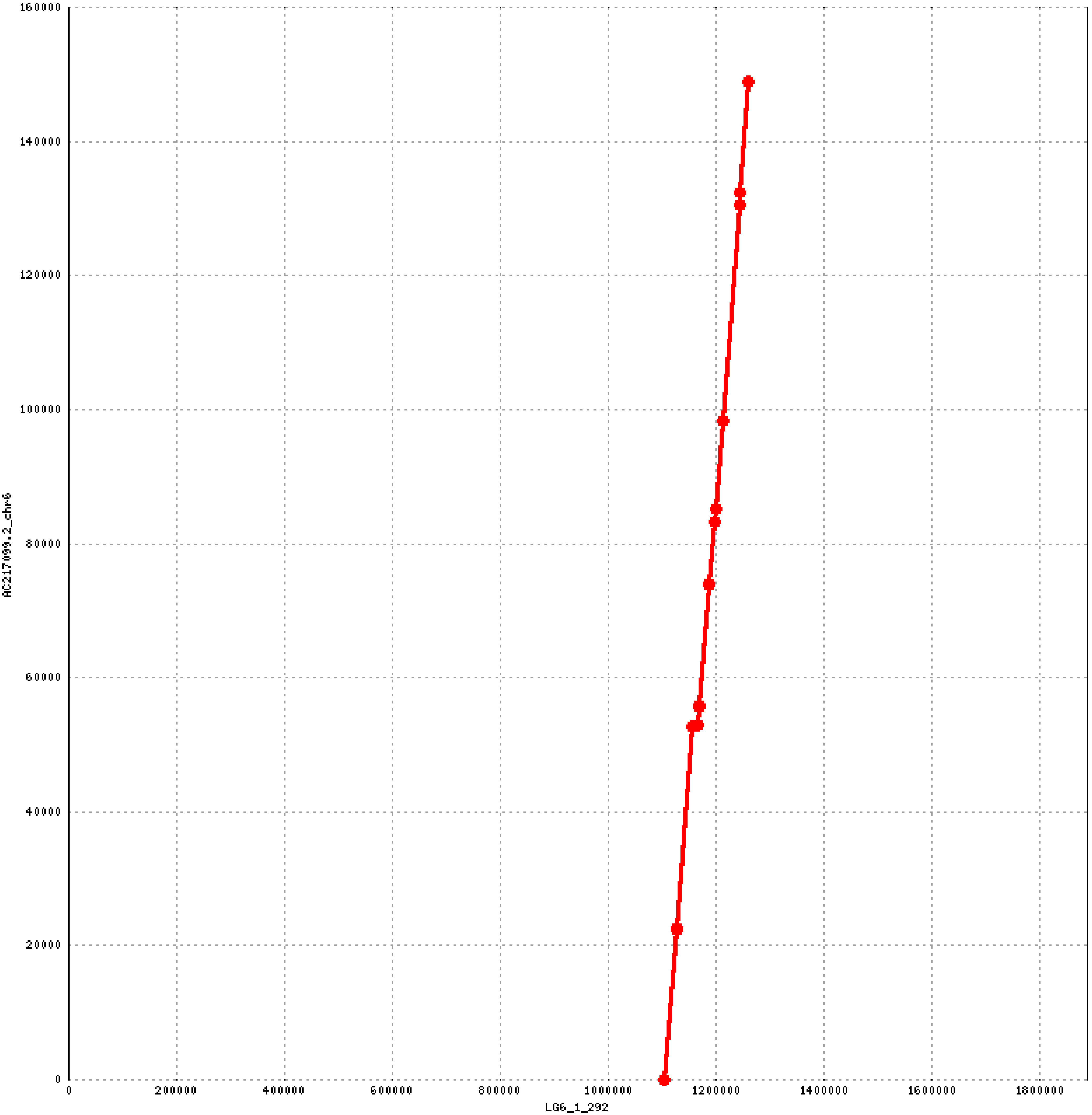

160000
140000
120000
100000
80000
60000
40000
20000
0
AC217099.2_chr6
0
200000
400000
600000
800000
1000000
1200000
1400000
1600000
1800000
LG6_1_292

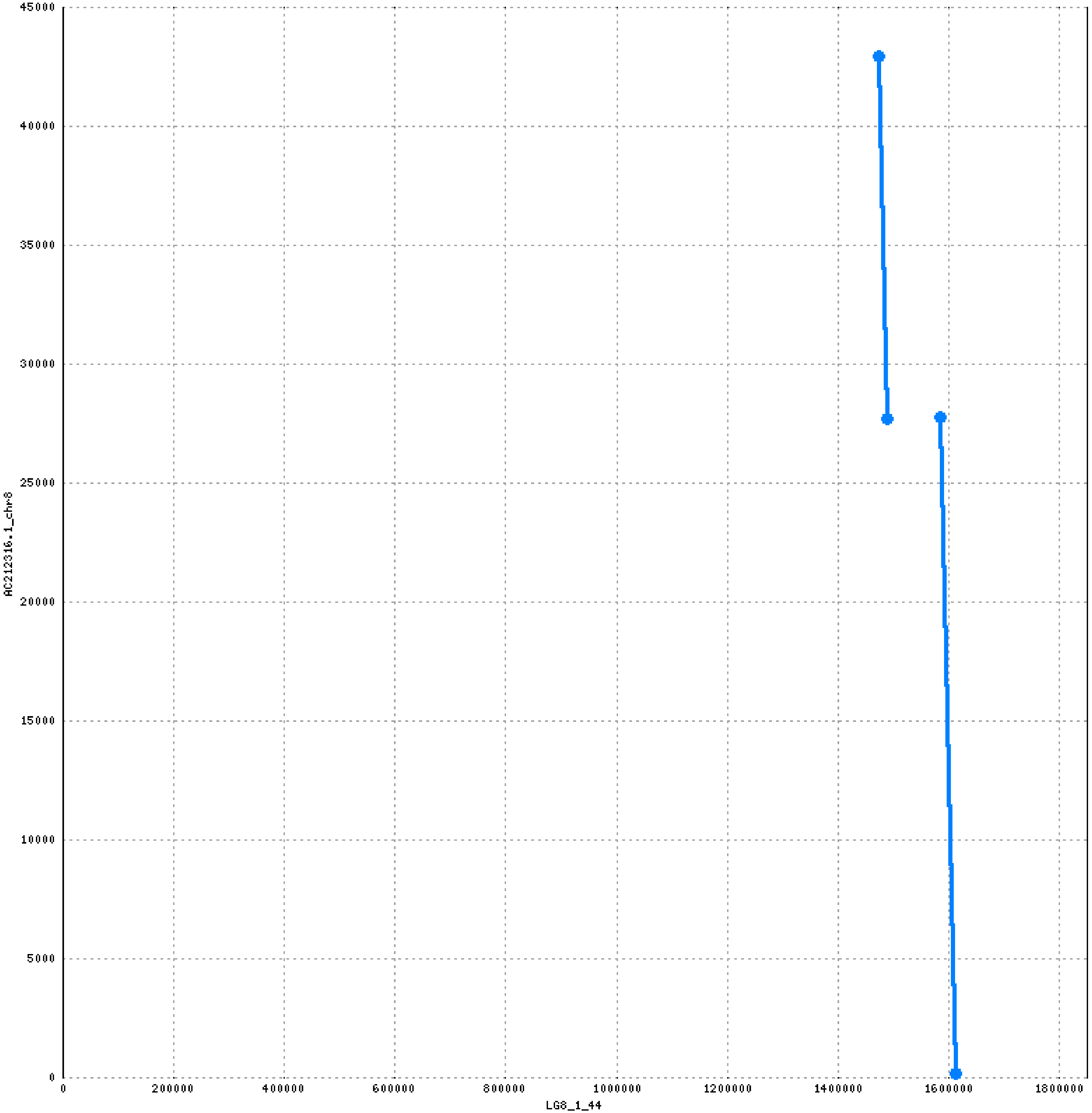

AC212316.1_chr8
LG8_1_44
45000
40000
35000
30000
25000
20000
15000
10000
5000
0
0
200000
400000
600000
800000
1000000
1200000
1400000
1600000
1800000

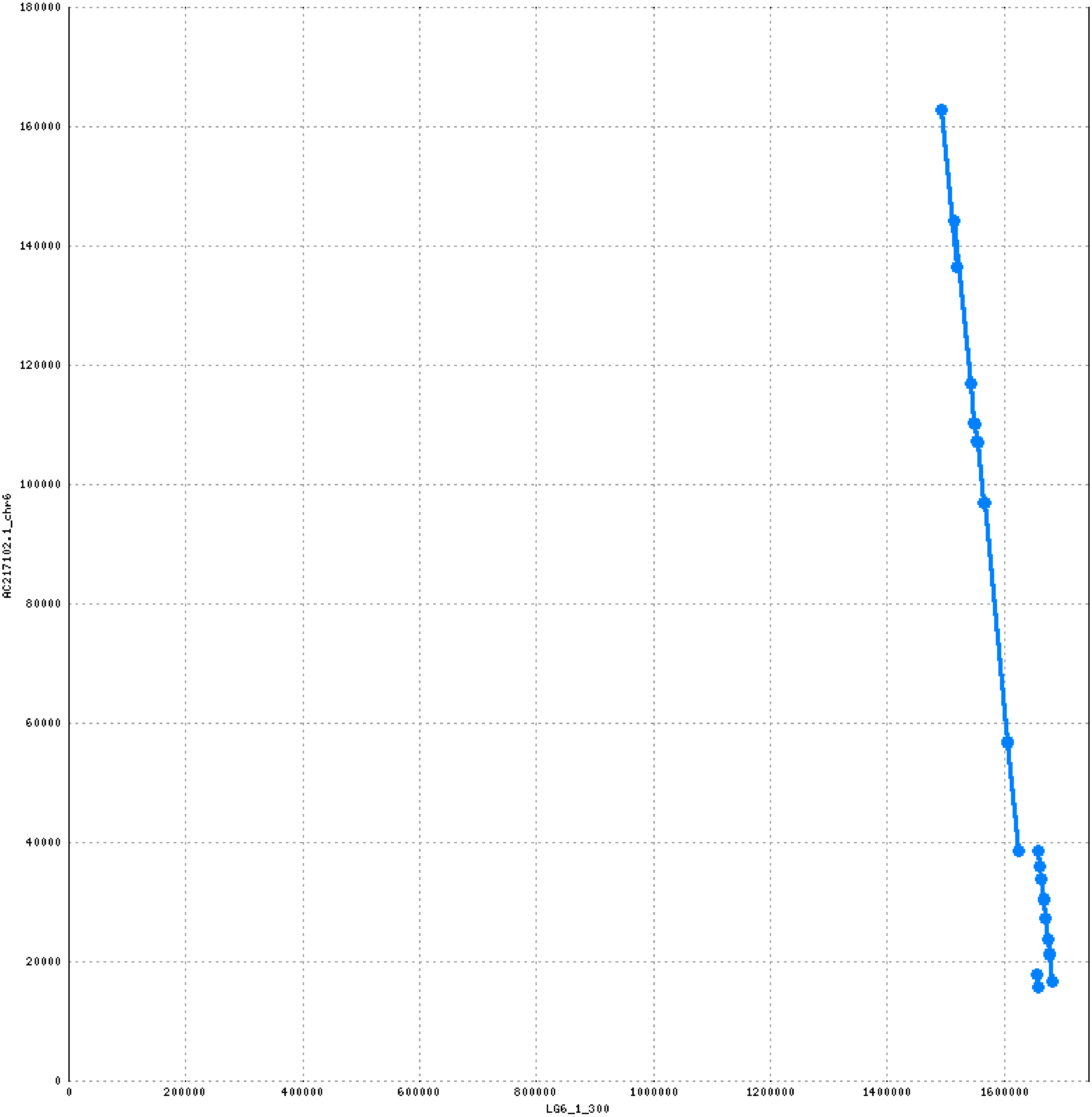

180000
160000
140000
120000
100000
80000
60000
40000
20000
0
AC217102.1_chr6
0
200000
400000
600000
800000
1000000
1200000
1400000
1600000
LG6_1_300

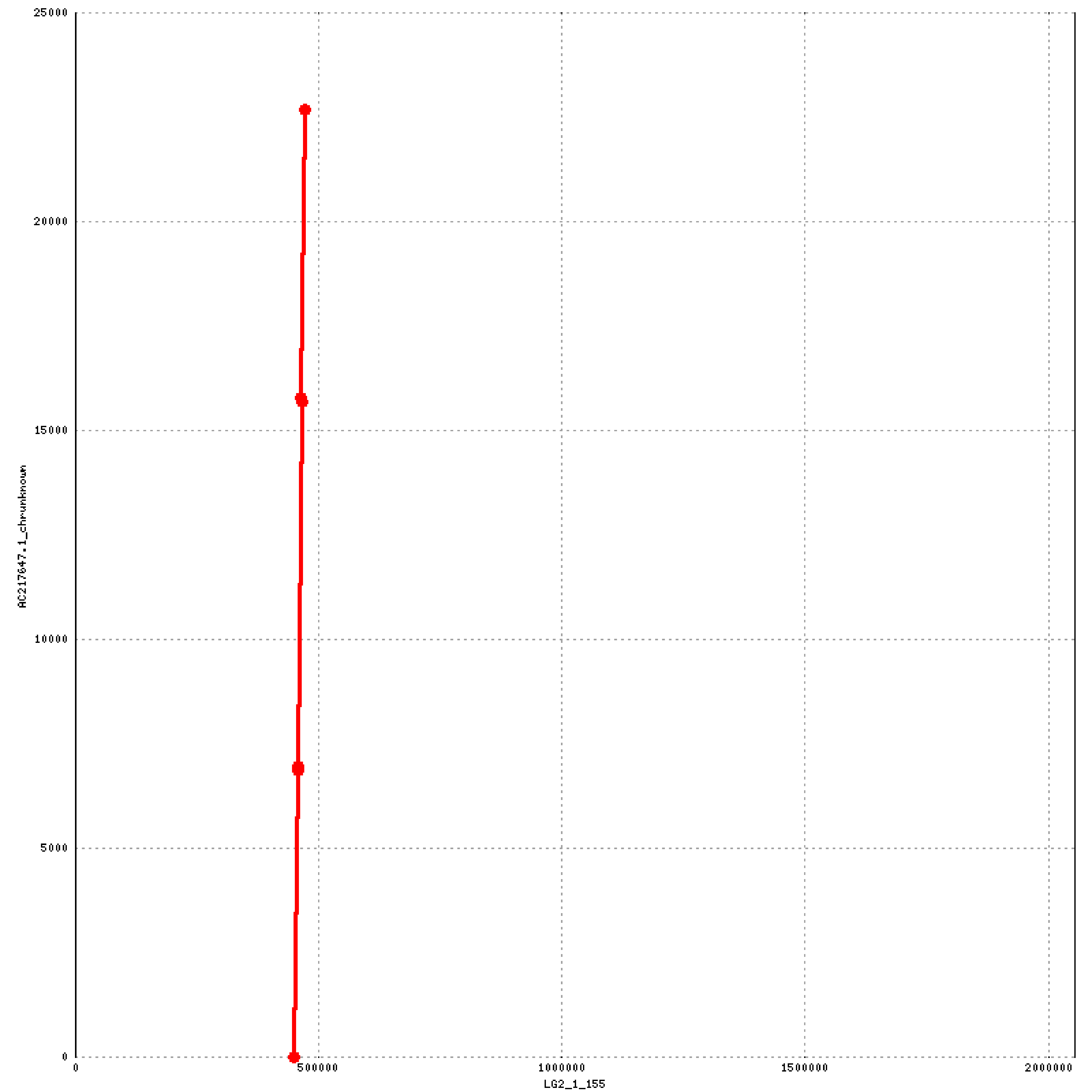

25000
20000
15000
10000
5000
0
AC217647.1_chrunknown
0
500000
1000000
1500000
2000000
LG2_1_155

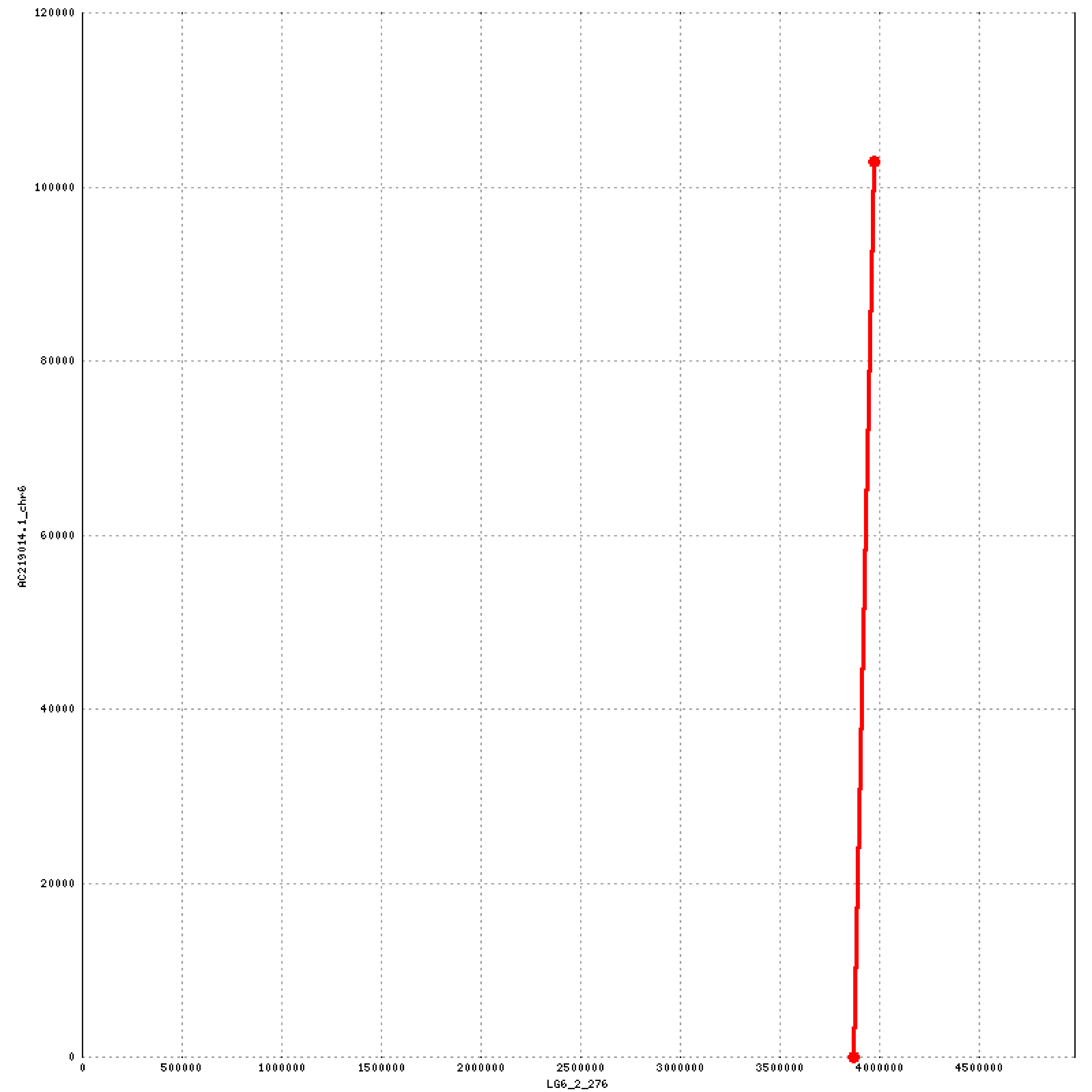
120000
100000
80000
60000
40000
20000
0
AC219014.1_chr6
0
500000
1000000
1500000
2000000
2500000
3000000
3500000
4000000
4500000
LG6_2_276

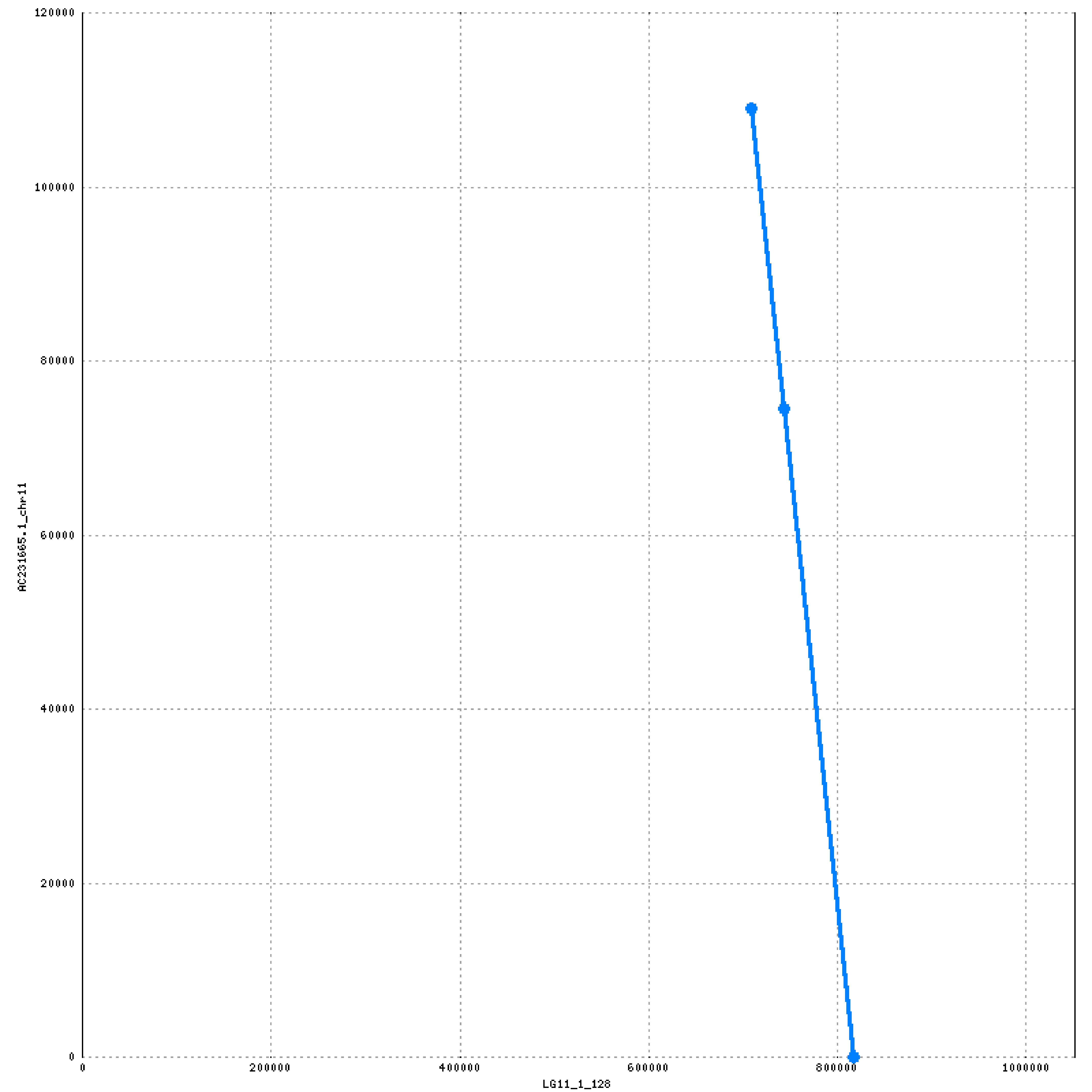

120000
100000
80000
60000
40000
20000
0
AC231665.1_chr11
0
200000
400000
600000
800000
1000000
LG11_1_128

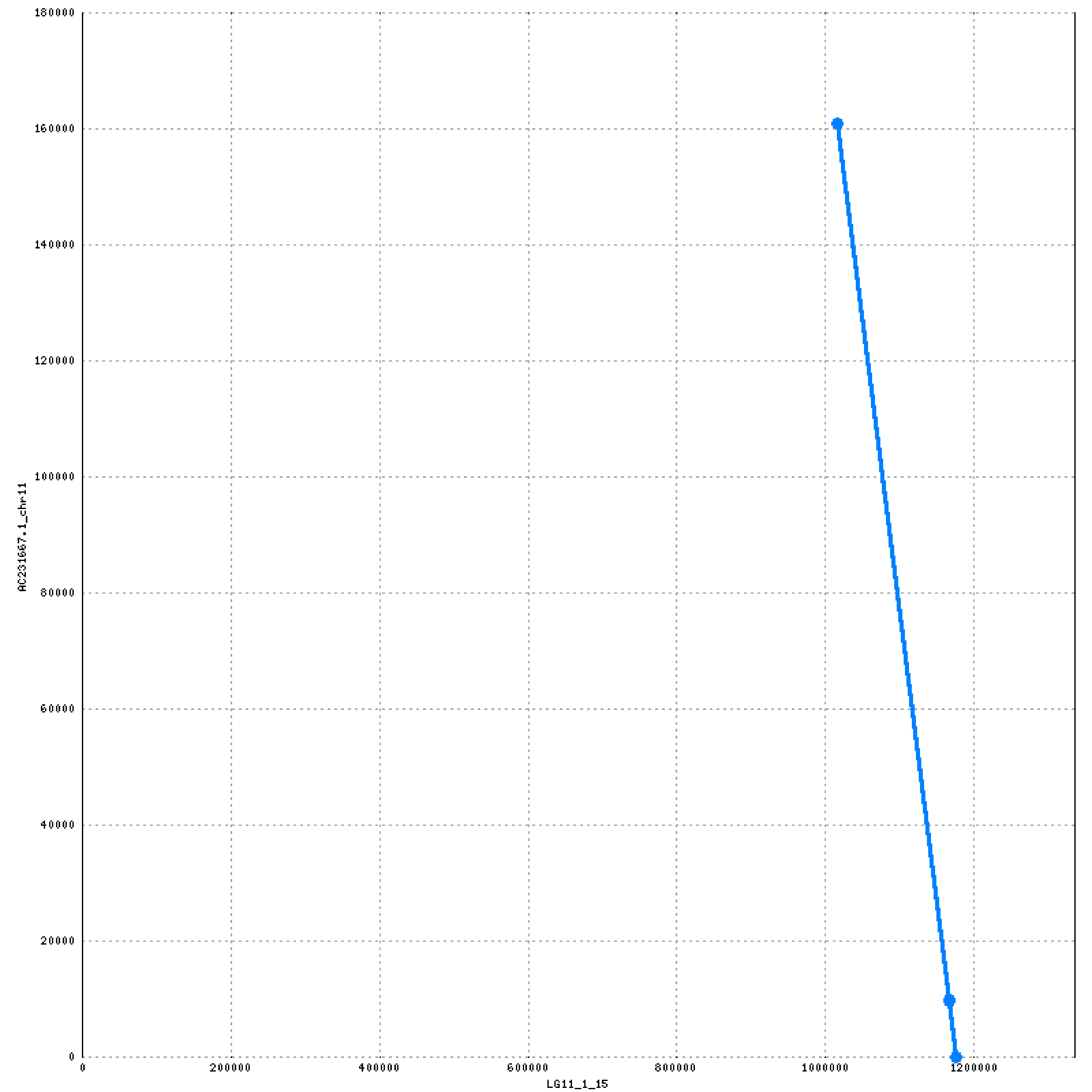
180000
160000
140000
120000
100000
80000
60000
40000
20000
0
AC231667.1_chr11
0
200000
400000
600000
800000
1000000
1200000
LG11_1_15

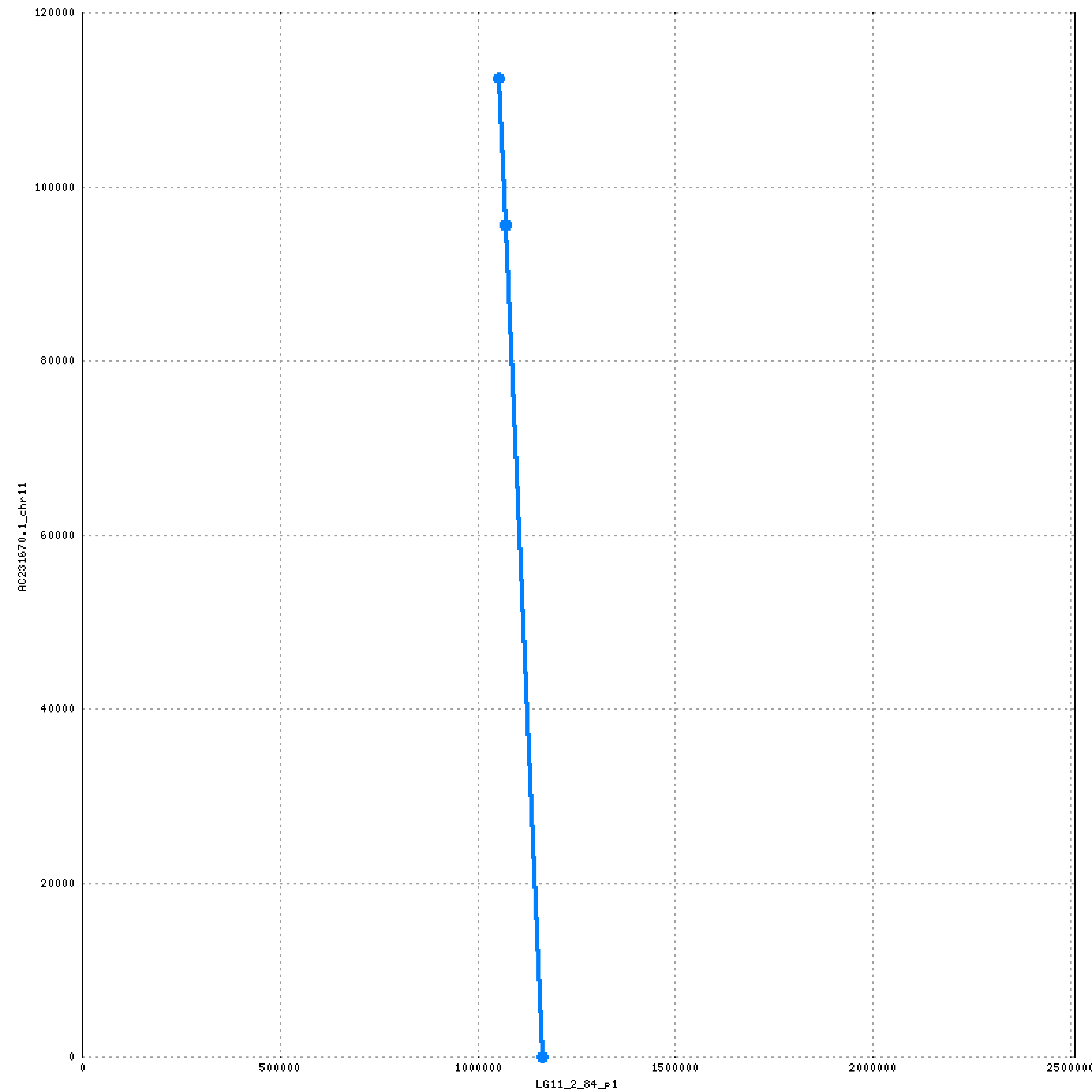

120000
100000
80000
60000
40000
20000
0
AC231670.1_chr11
0
500000
1000000
1500000
2000000
2500000
LG11_2_84_p1

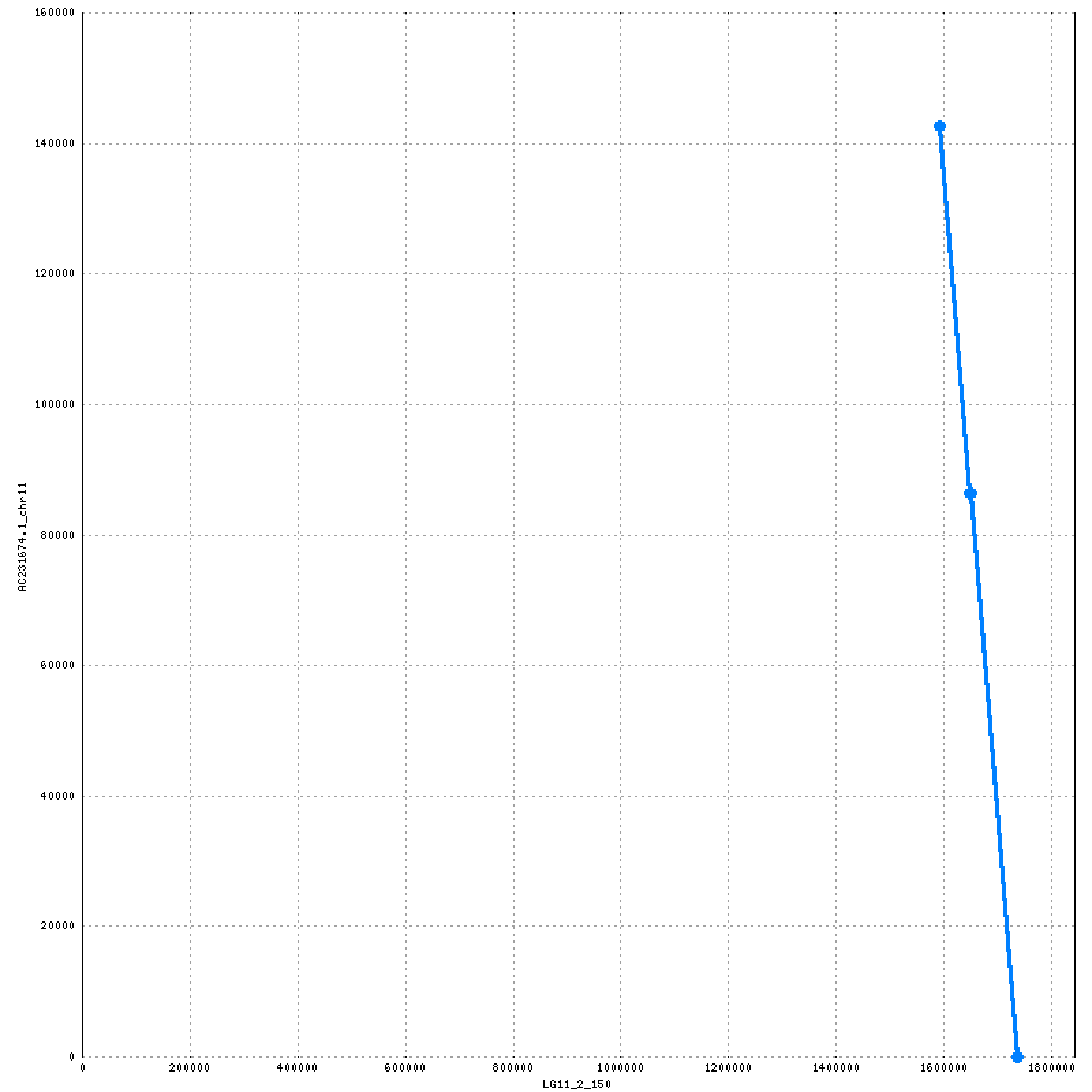

160000
140000
120000
100000
80000
60000
40000
20000
0
AC231674.1_chr11
0
200000
400000
600000
800000
1000000
1200000
1400000
1600000
1800000
LG11_2_150

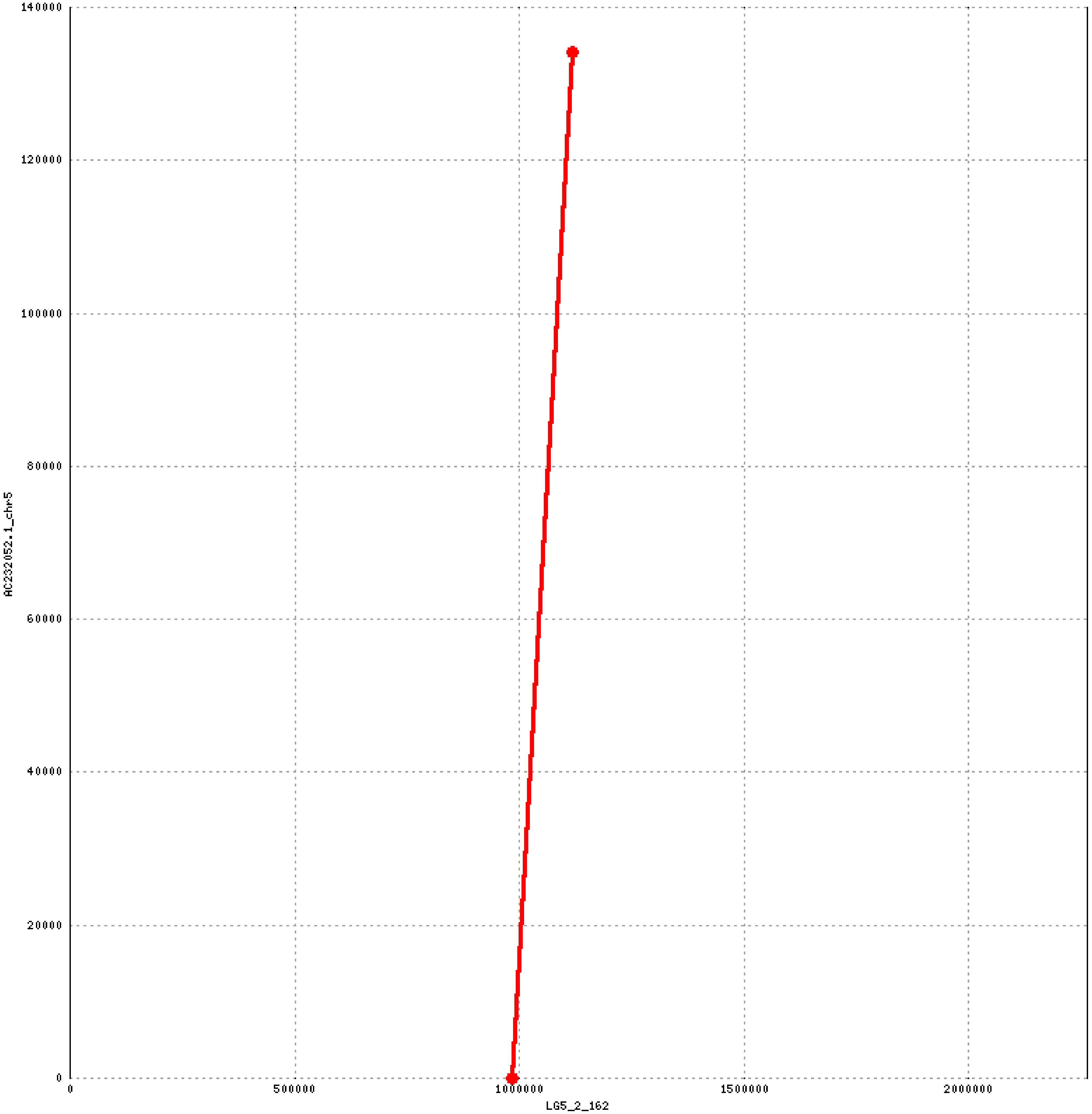

140000
120000
100000
80000
60000
40000
20000
0
AC232052.1_chr5
0
500000
1000000
1500000
2000000
LG5_2_162

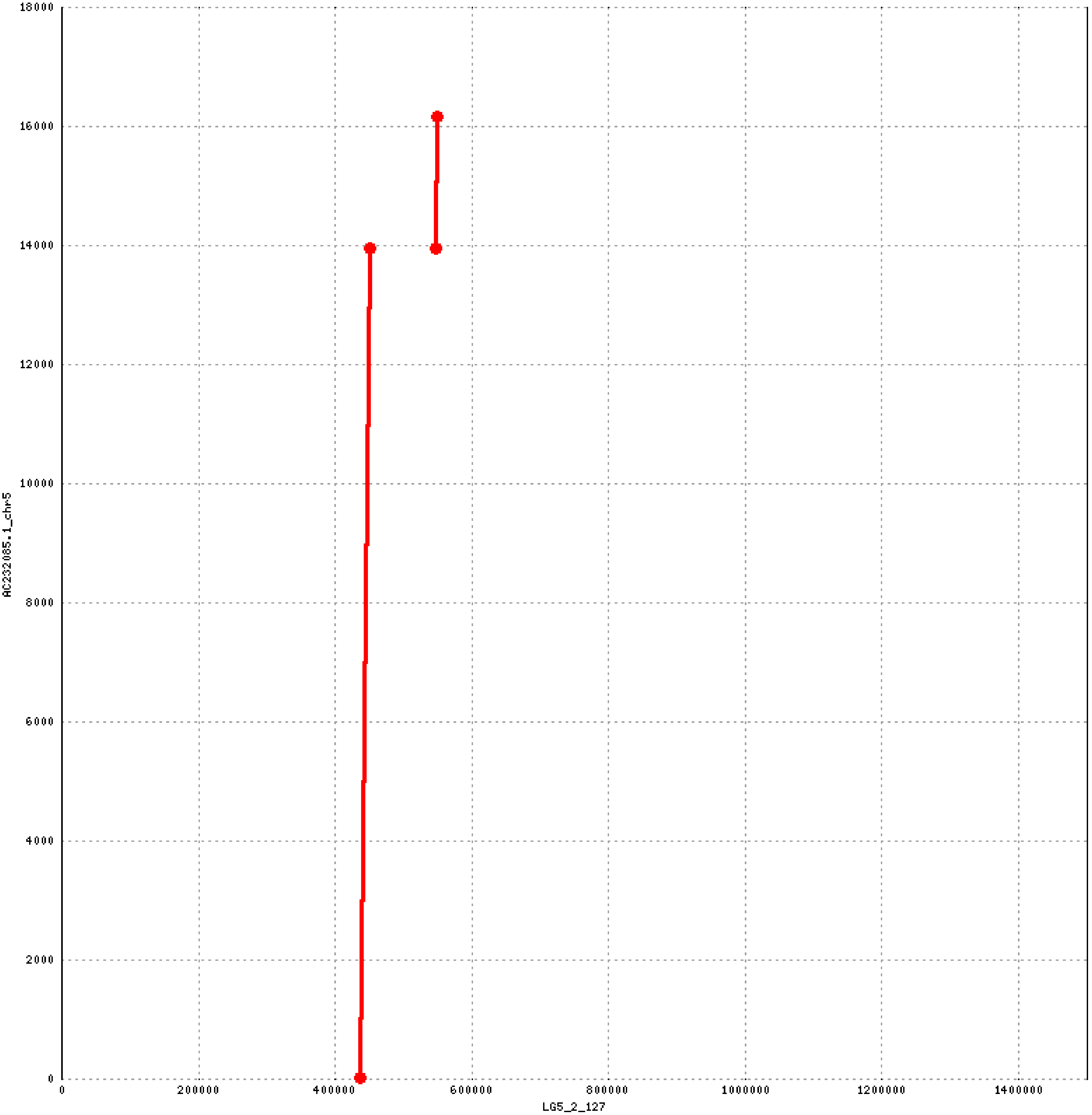
18000
16000
14000
12000
10000
8000
6000
4000
2000
0
AC232085.1_chr5
0
200000
400000
600000
800000
1000000
1200000
1400000
LG5_2_127

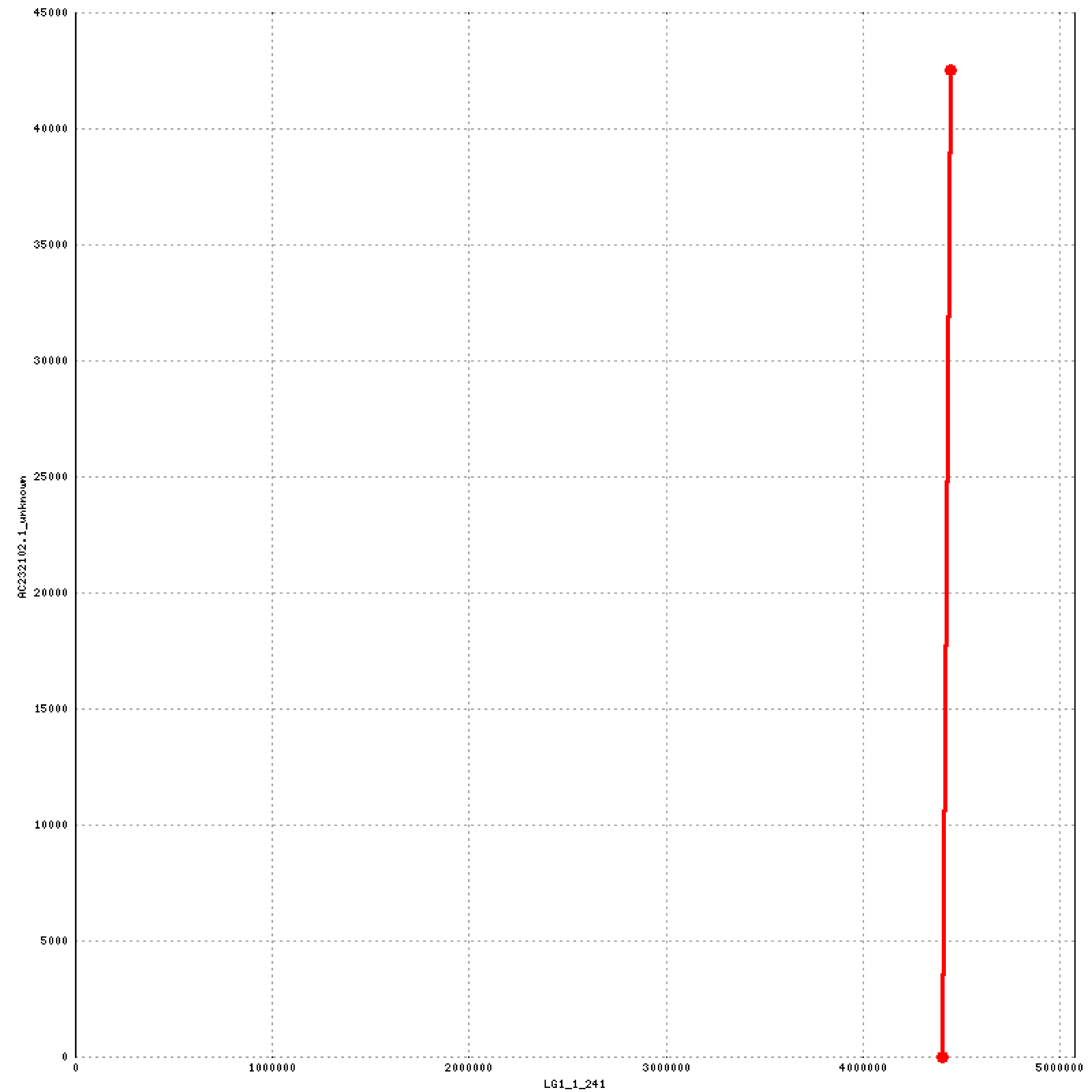

45000
40000
35000
30000
25000
20000
15000
10000
5000
0
AC232102.1_unknown
0
1000000
2000000
3000000
4000000
5000000
LG1_1_241

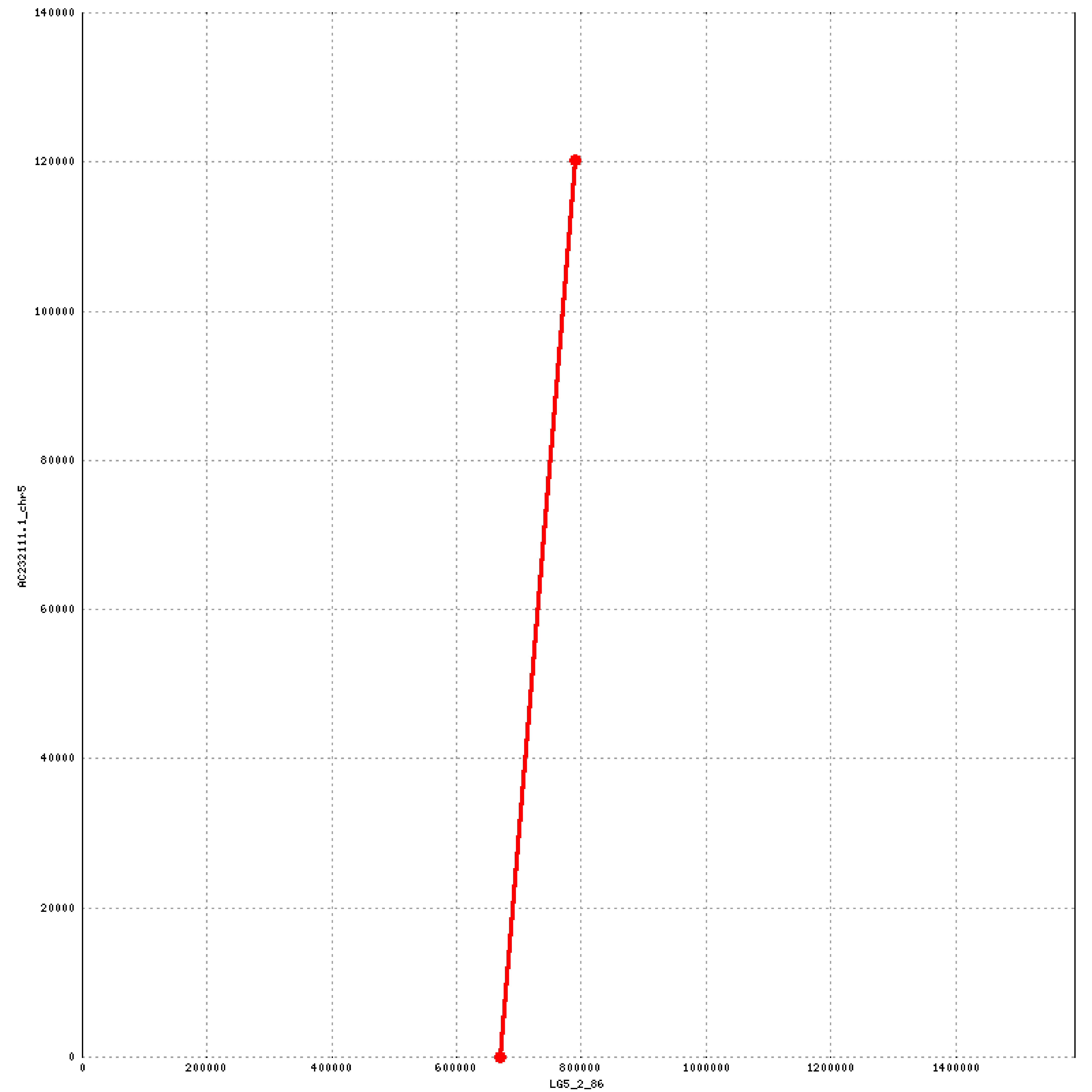

140000
120000
100000
80000
60000
40000
20000
0
AC232111.1_chr5
0
200000
400000
600000
800000
1000000
1200000
1400000
LG5_2_86

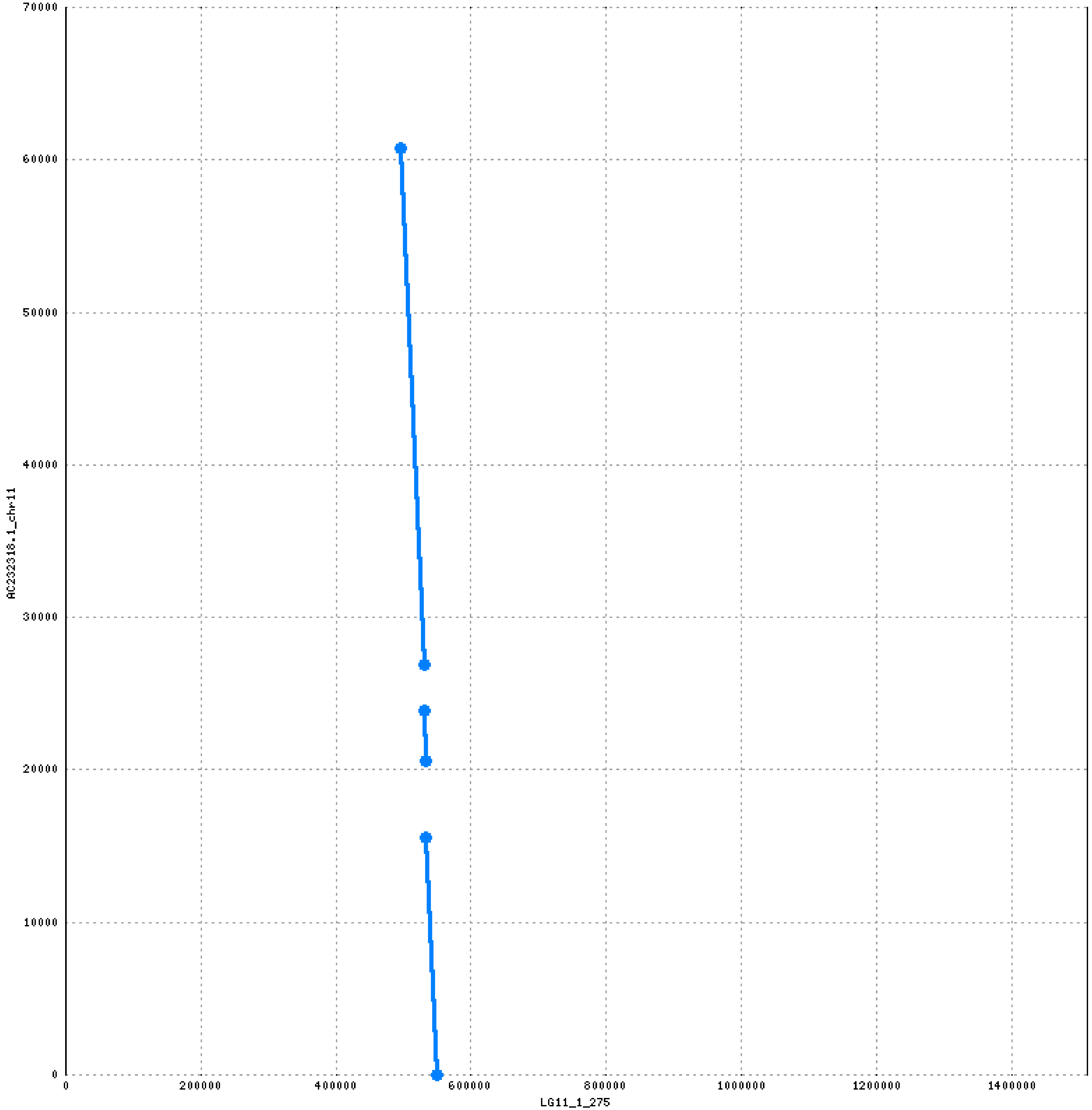

70000
60000
50000
40000
30000
20000
10000
0
AC232318.1_chr11
0
200000
400000
600000
800000
1000000
1200000
1400000
LG11_1_275

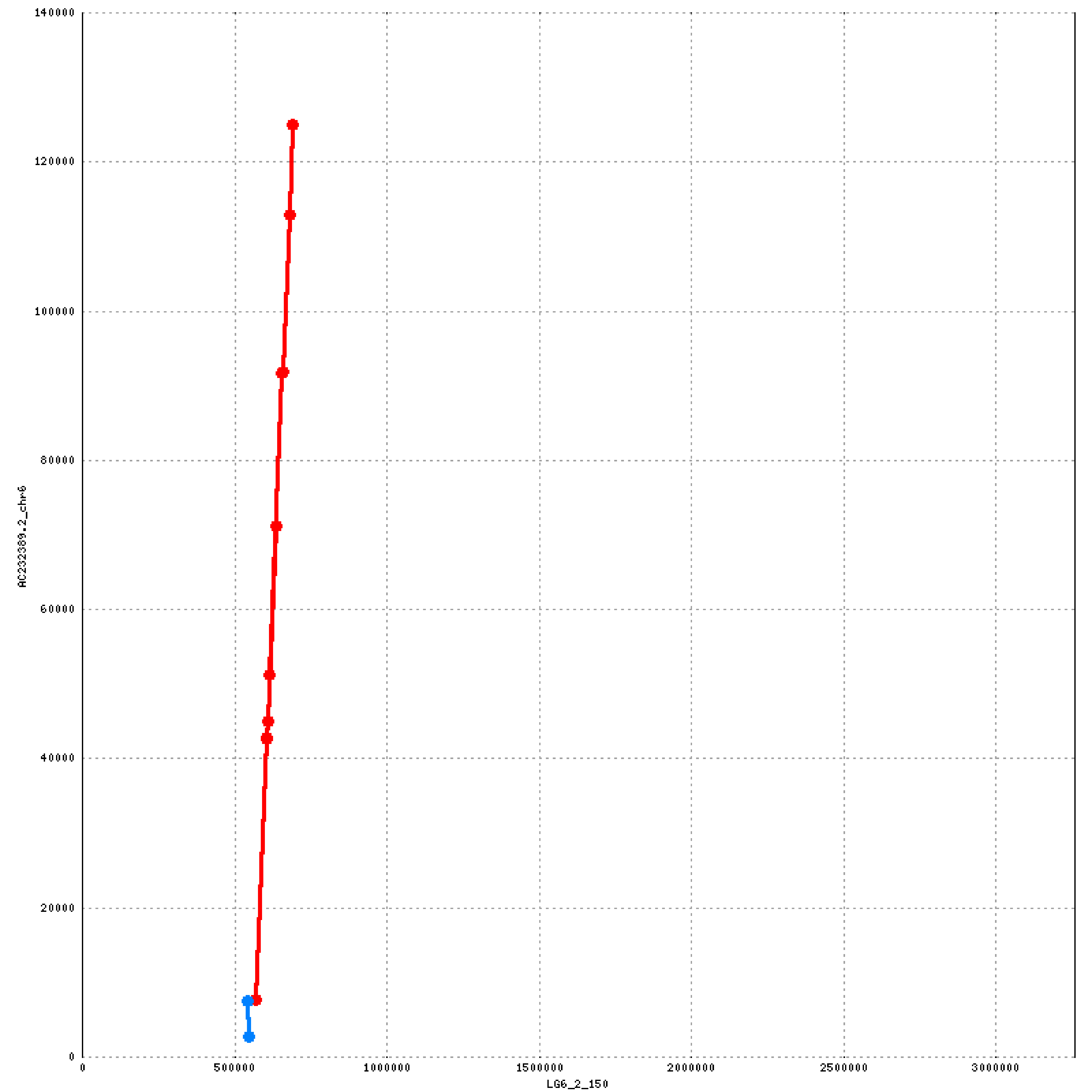
140000
120000
100000
80000
60000
40000
20000
0
AC232389.2_chr6
0
500000
1000000
1500000
2000000
2500000
3000000
LG6_2_150

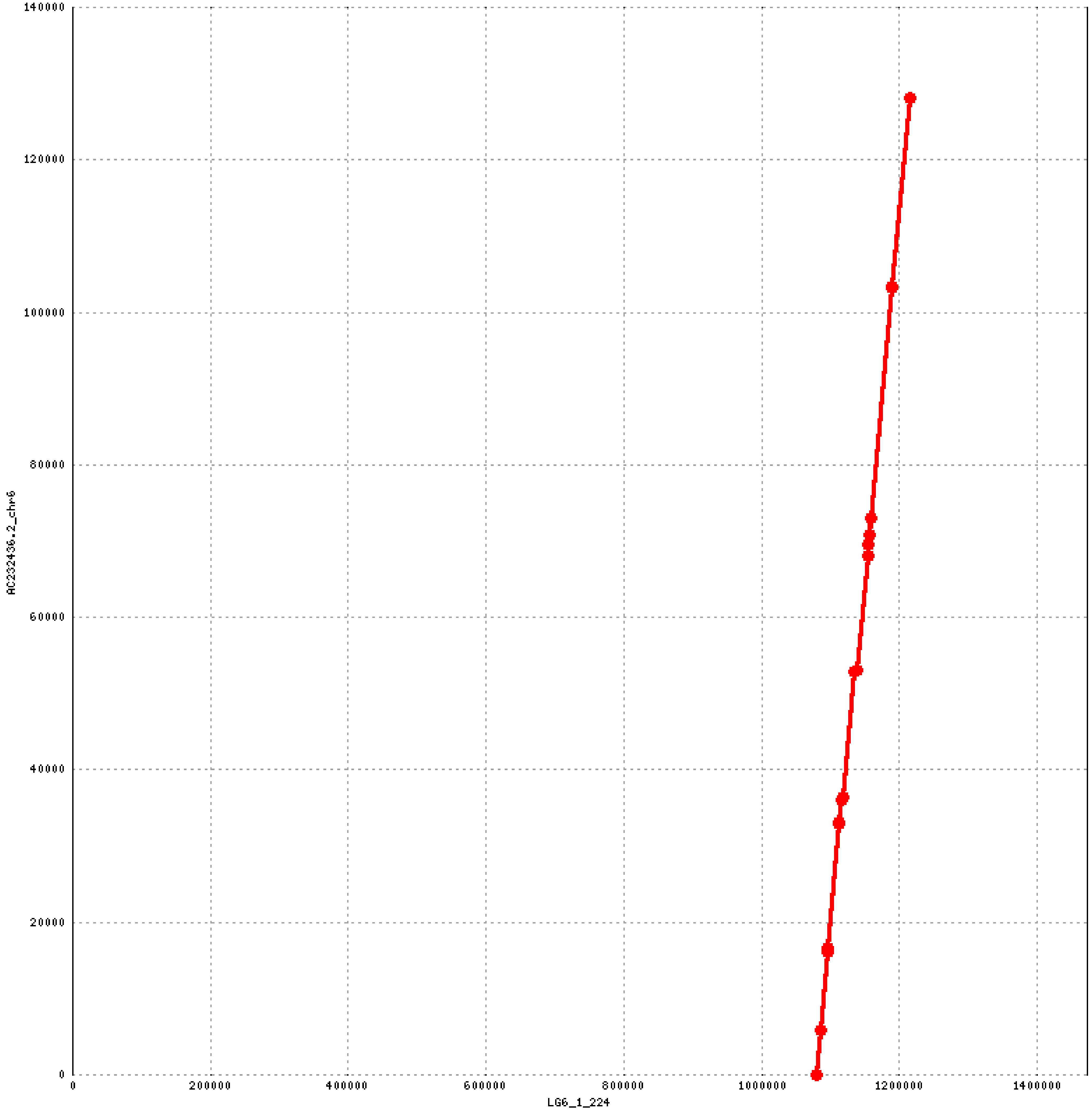

140000
120000
100000
80000
60000
40000
20000
0
AC232436.2_chr6
0
200000
400000
600000
800000
1000000
1200000
1400000
LG6_1_224

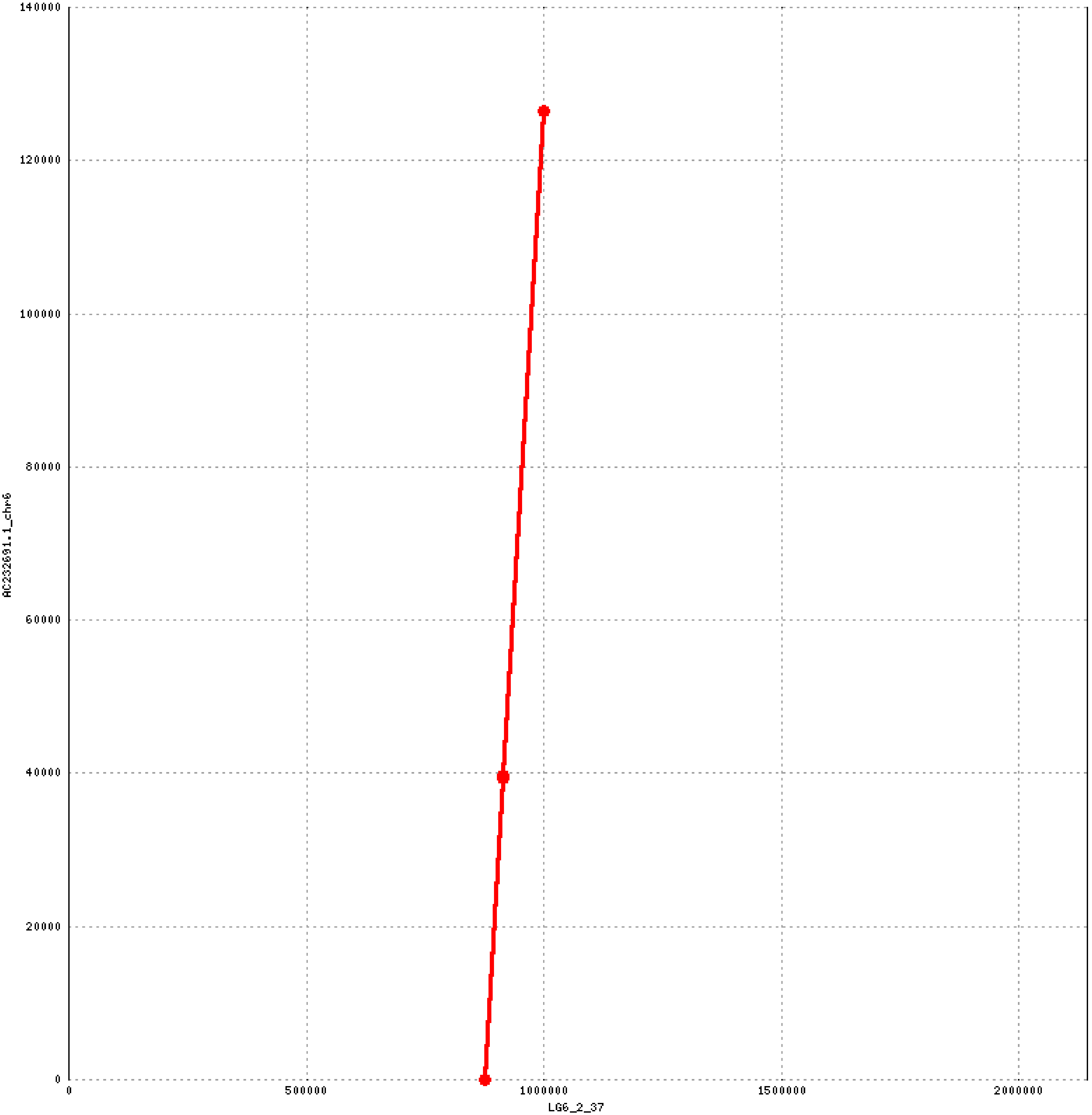
140000
120000
100000
80000
60000
40000
20000
0
AC232691.1_chr6
0
500000
1000000
1500000
2000000
LG6_2_37

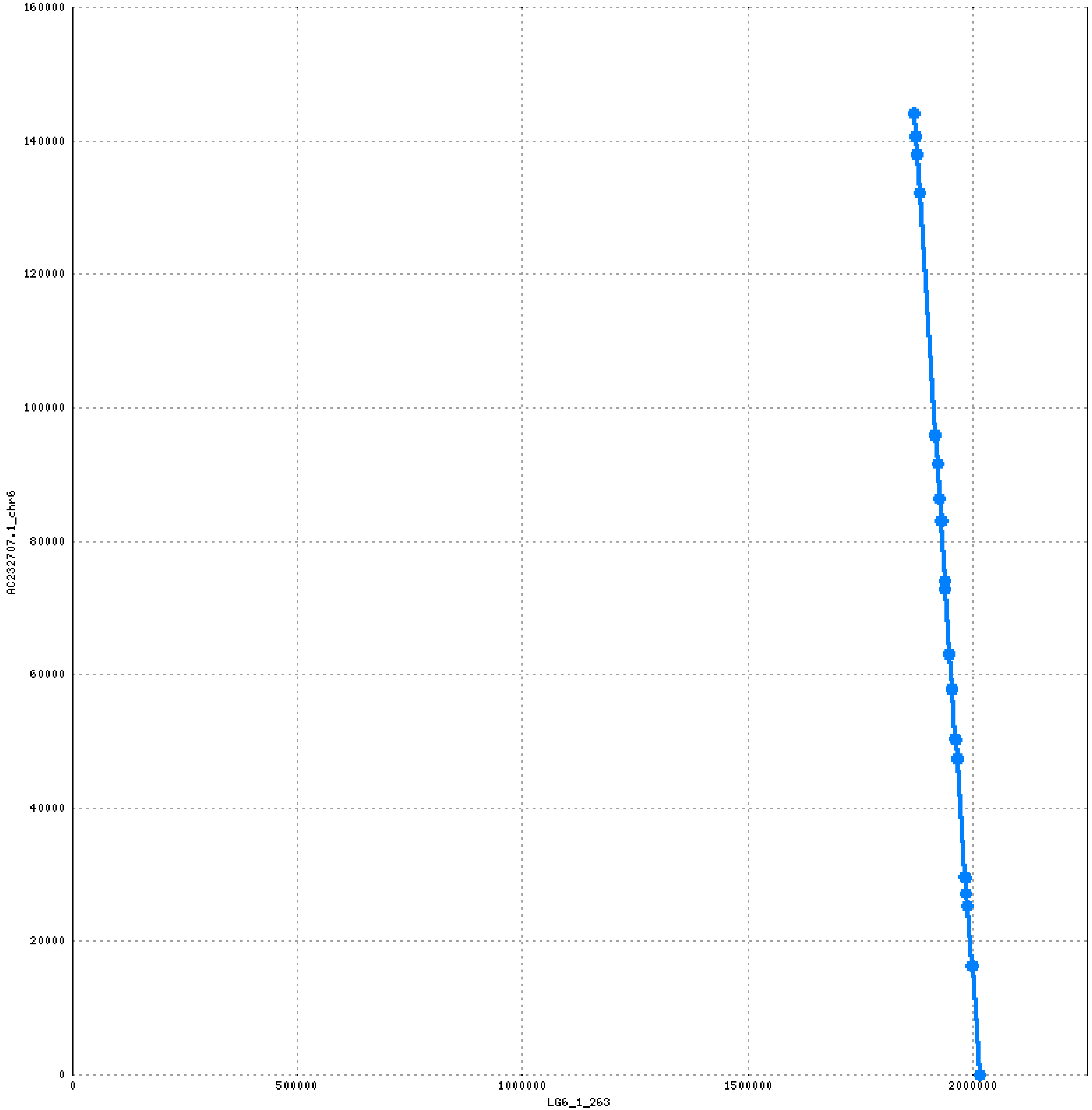

160000
140000
120000
100000
80000
60000
40000
20000
0
AC232707.1_chr6
0
500000
1000000
1500000
2000000
LG6_1_263

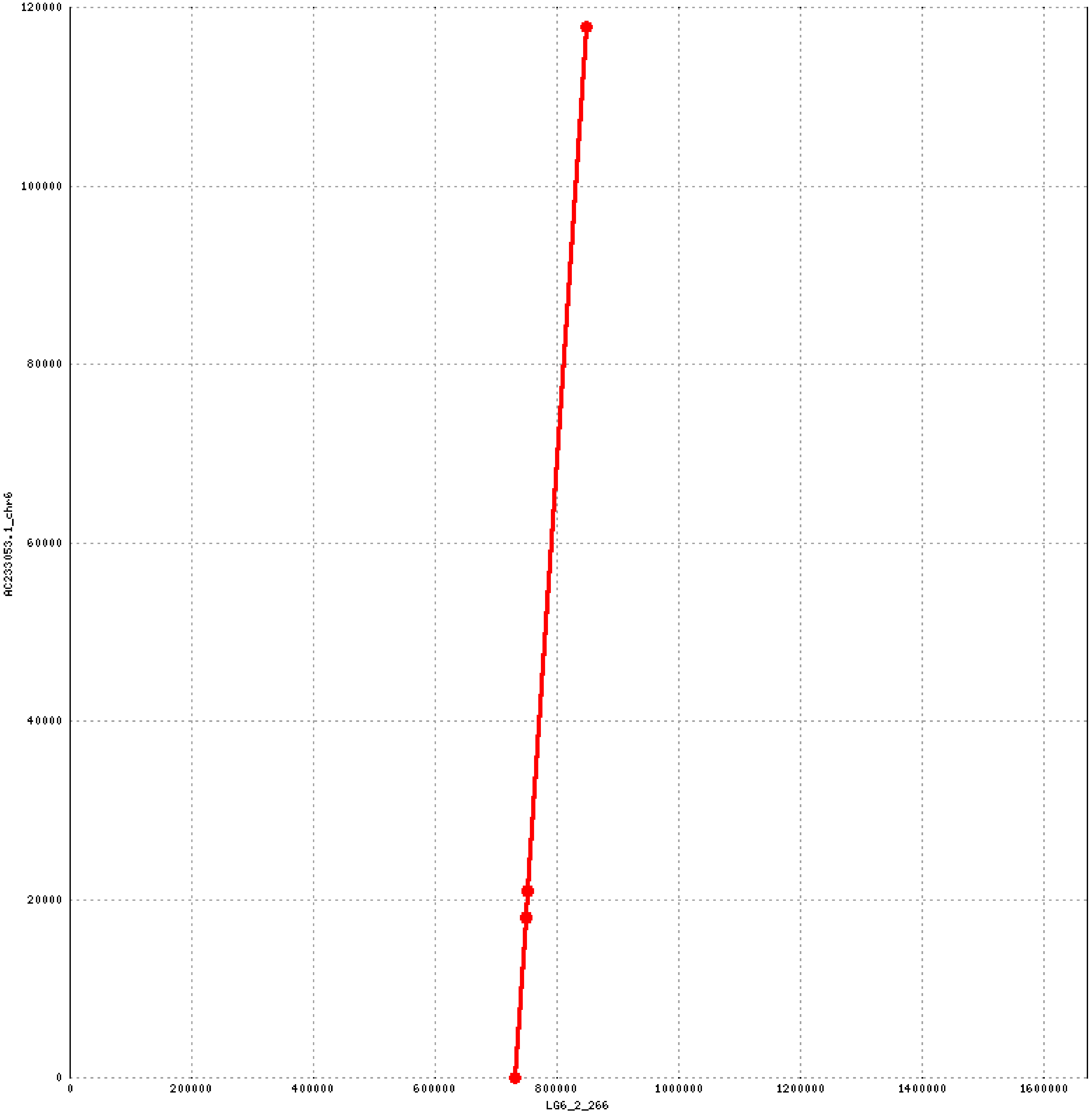

120000
100000
80000
60000
40000
20000
0
AC233053.1_chr6
0
200000
400000
600000
800000
1000000
1200000
1400000
1600000
LG6_2_266

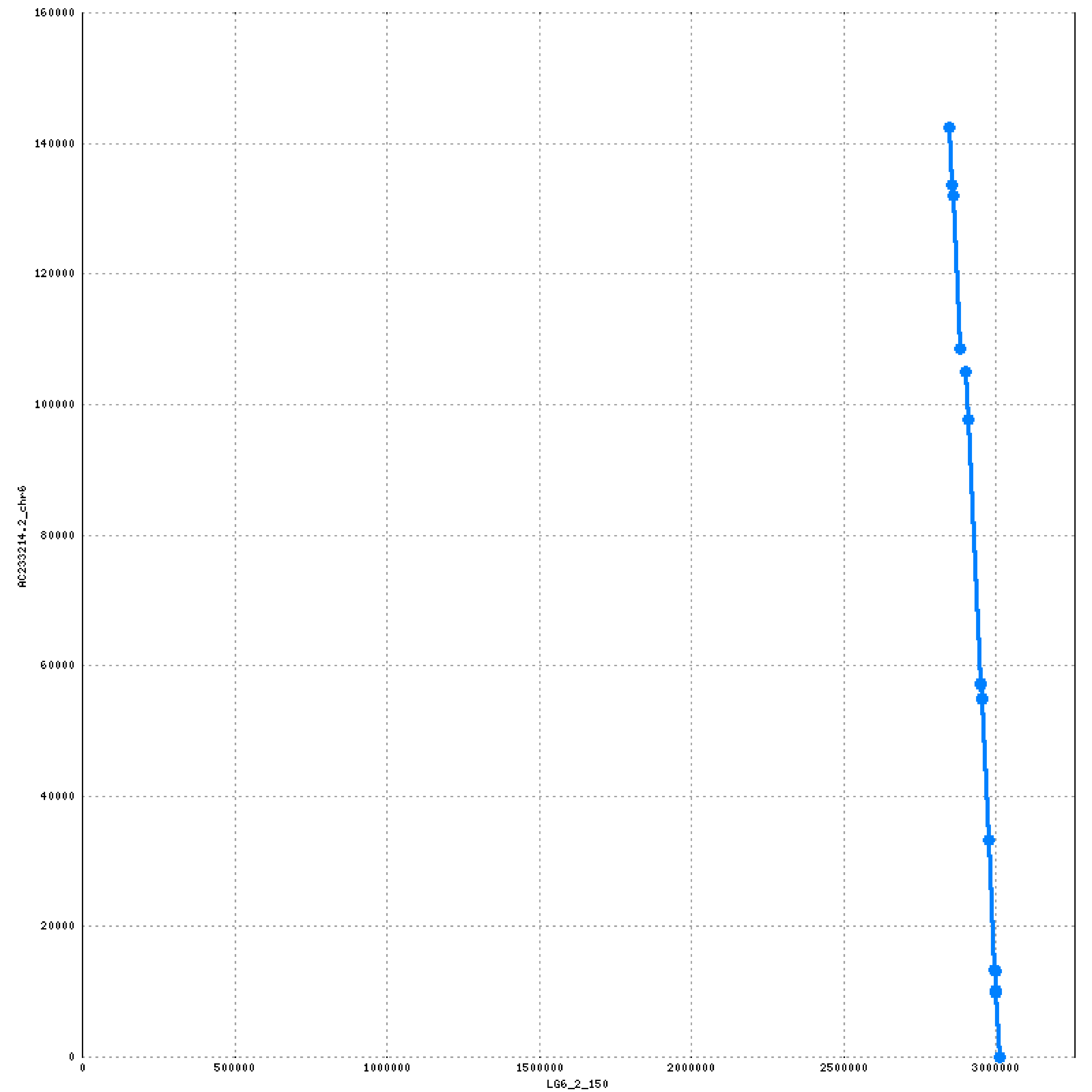

160000
140000
120000
100000
80000
60000
40000
20000
0
AC233214.2_chr6
0
500000
1000000
1500000
2000000
2500000
3000000
LG6_2_150

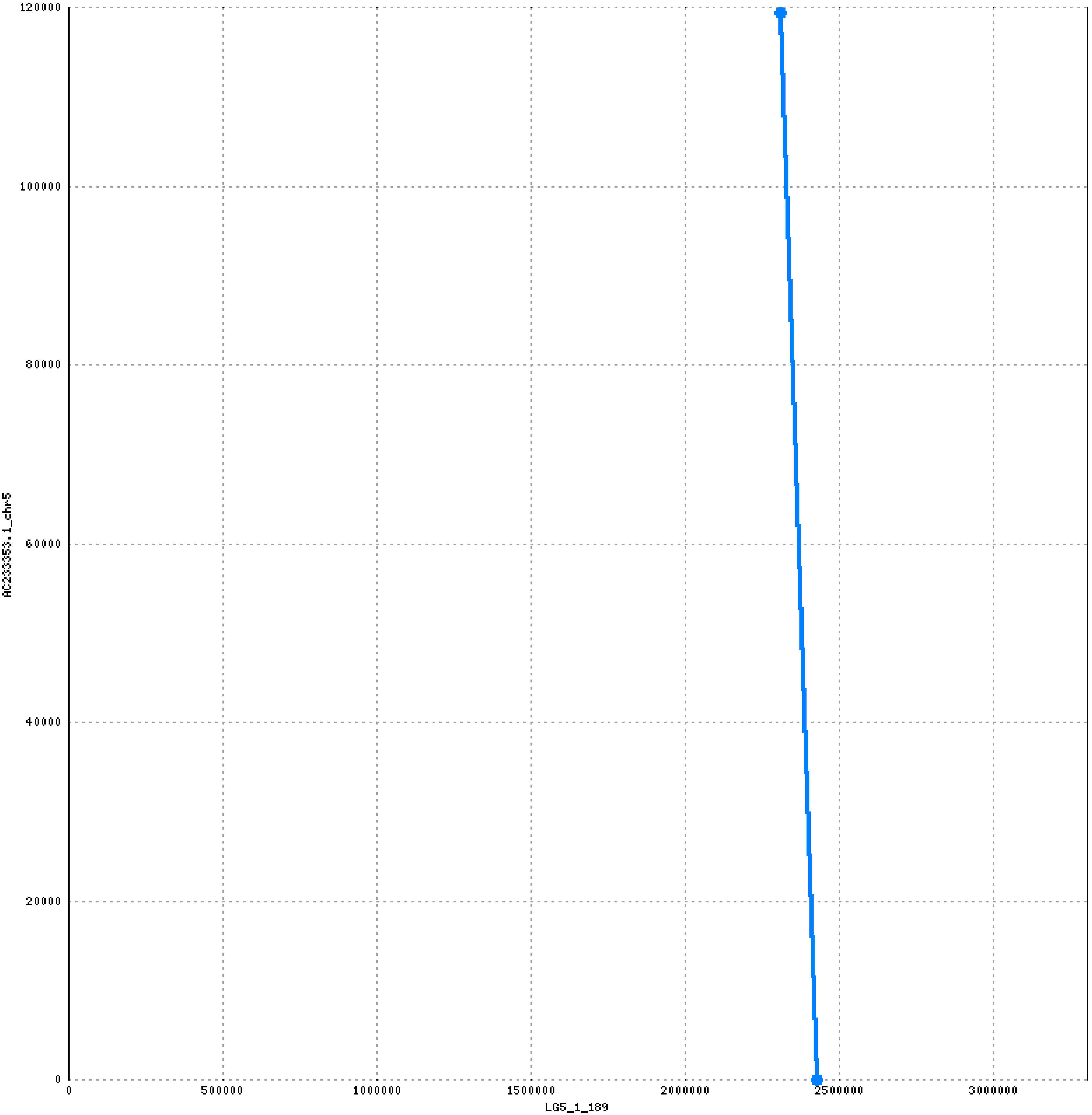

120000
100000
80000
60000
40000
20000
0
AC233353.1_chr5
0
500000
1000000
1500000
2000000
2500000
3000000
LG5_1_189

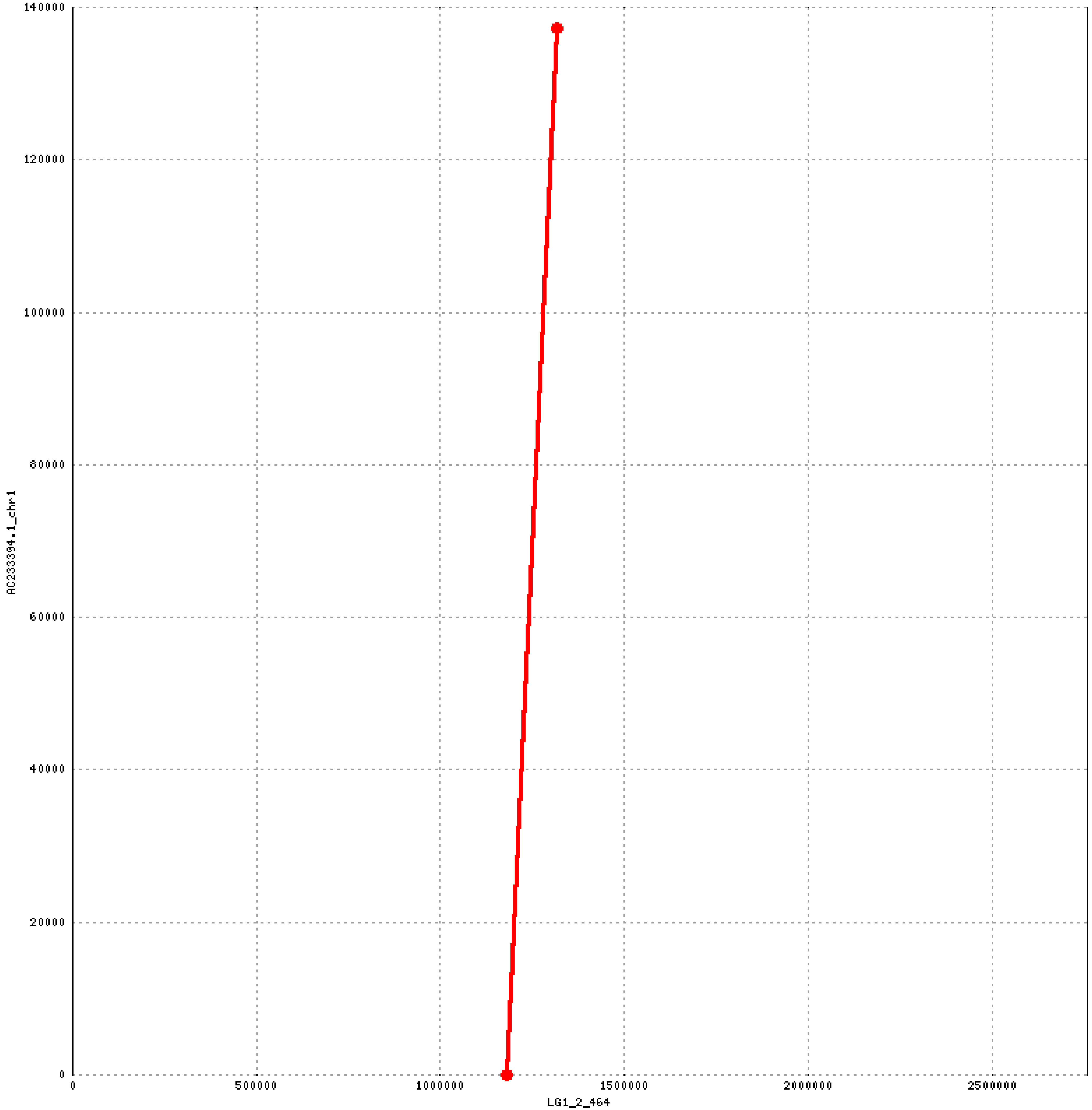
140000
120000
100000
80000
60000
40000
20000
0
AC233394.1_chr1
0
500000
1000000
1500000
2000000
2500000
LG1_2_464

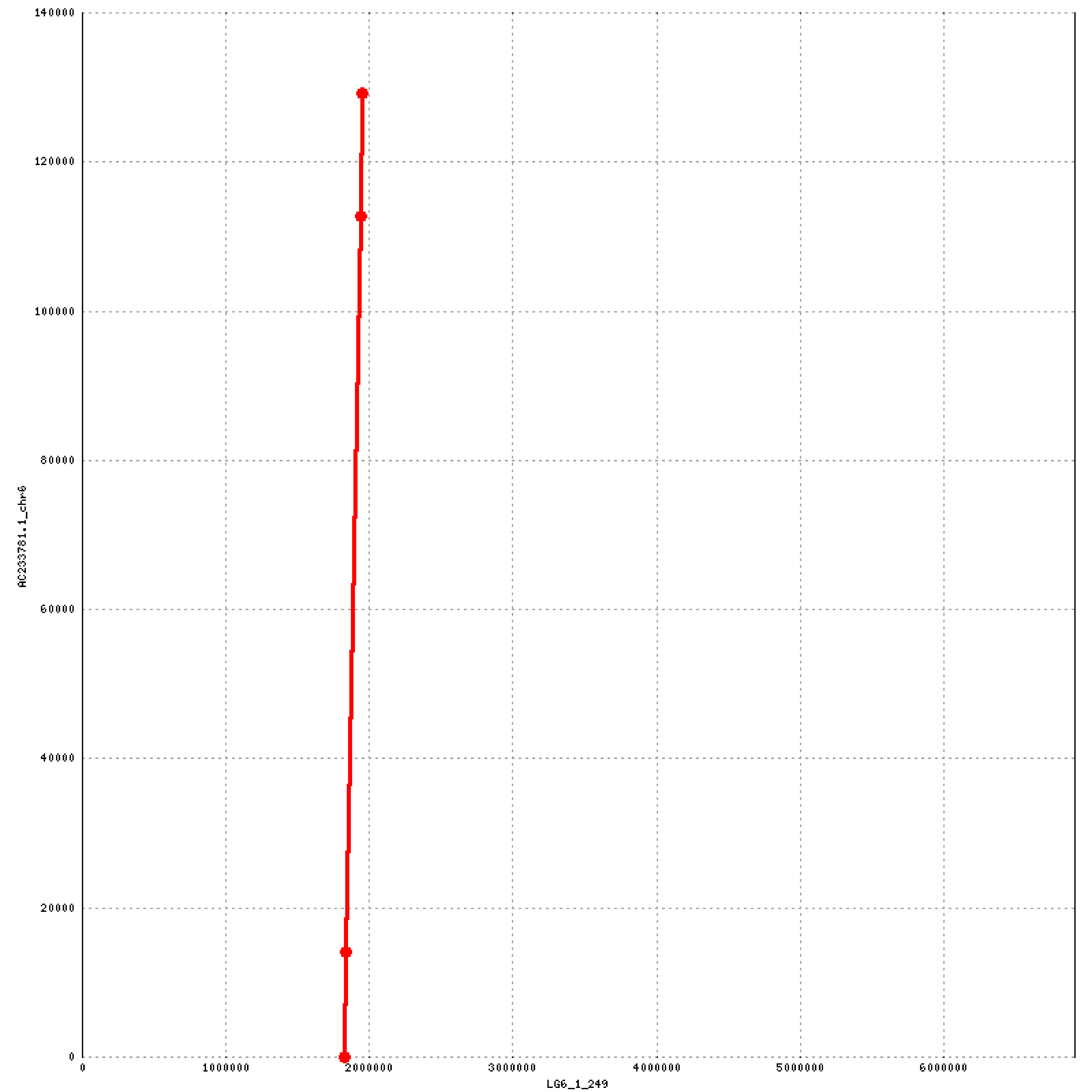
140000
120000
100000
80000
60000
40000
20000
0
AC233781.1_chr6
0
1000000
2000000
3000000
4000000
5000000
6000000
LG6_1_249

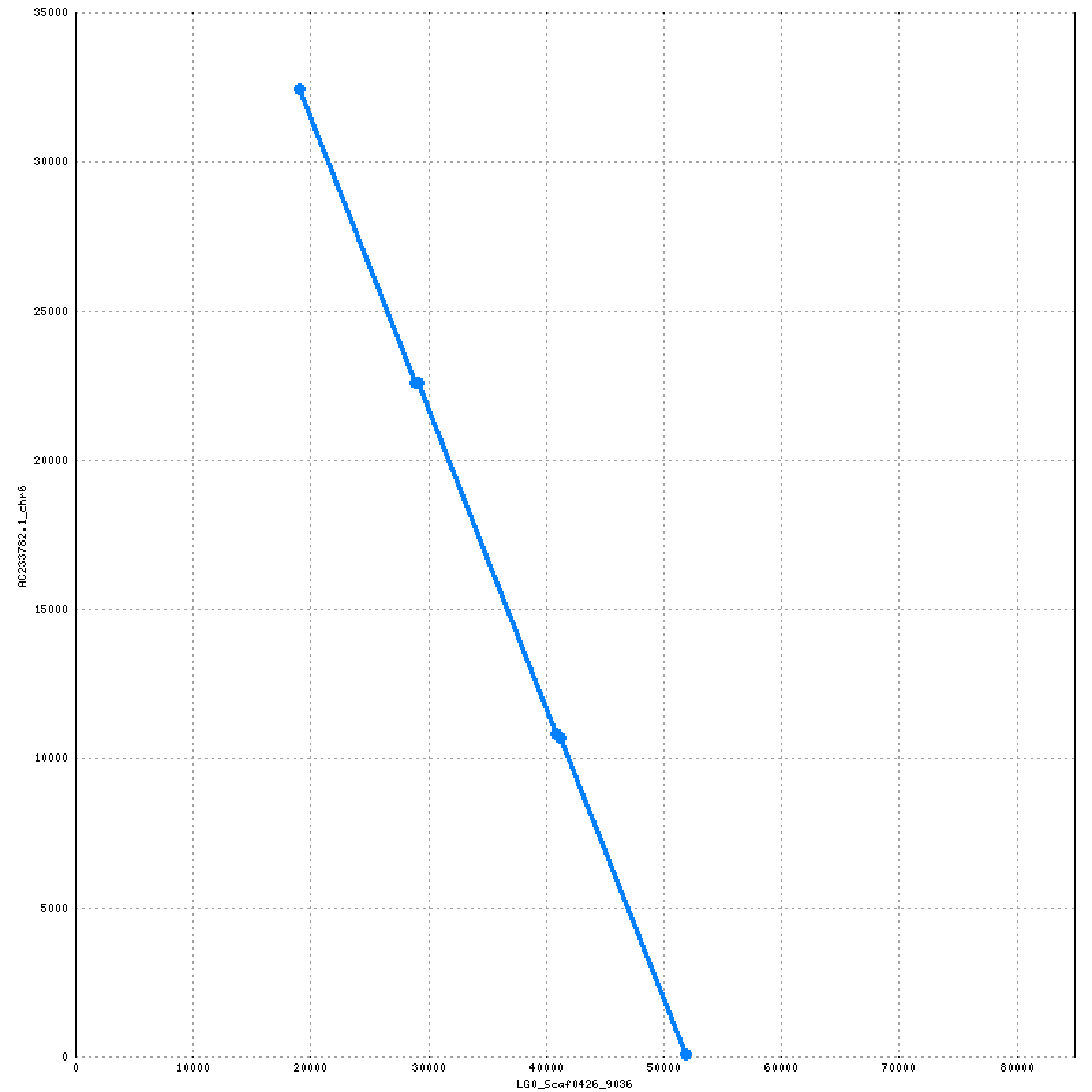

35000
30000
25000
20000
15000
10000
5000
0
AC233782.1_chr6
0
10000
20000
30000
40000
50000
60000
70000
80000
LG0_Scaf0426_9036

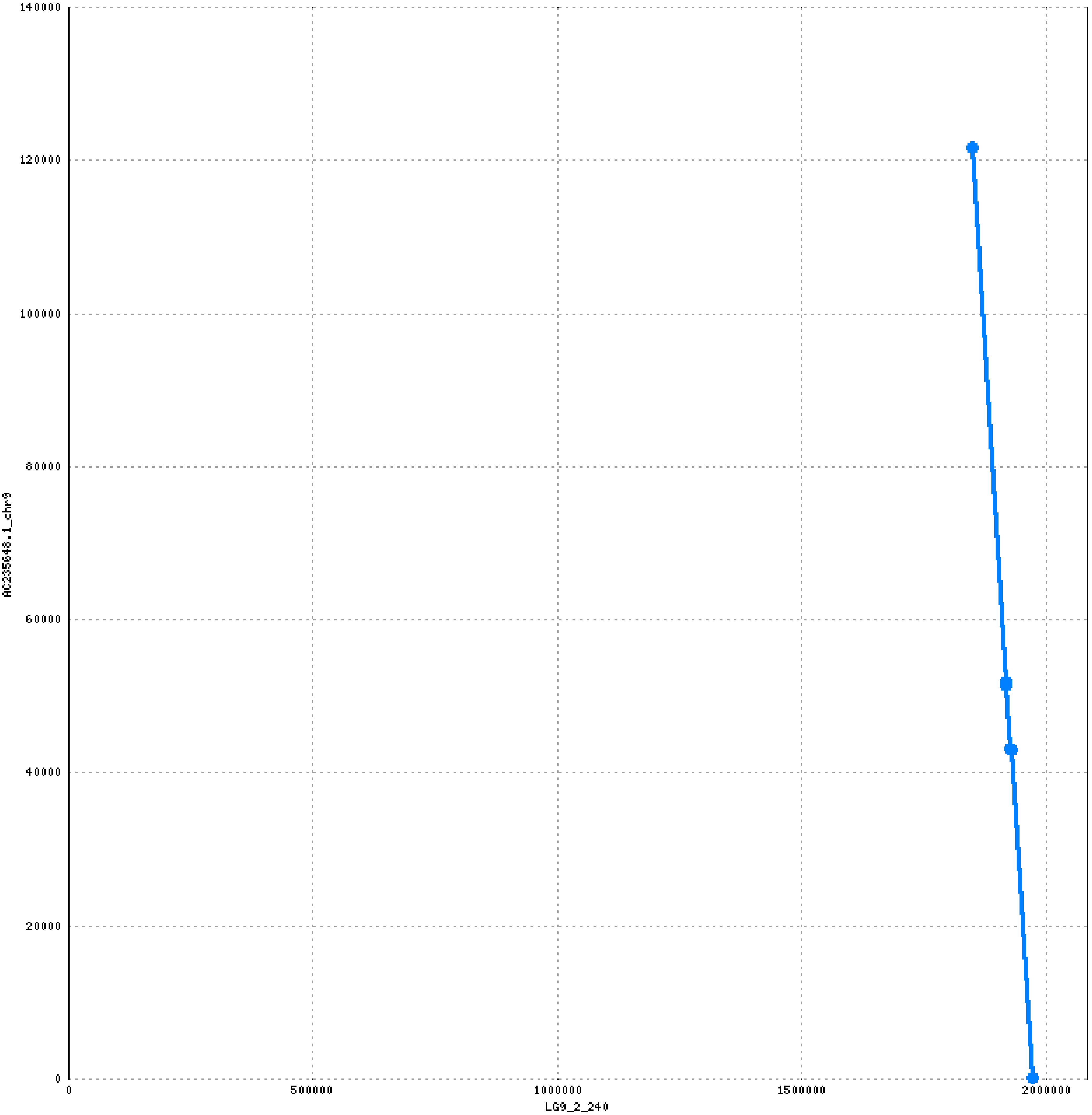
140000
120000
100000
80000
60000
40000
20000
0
AC235648.1_chr9
0
500000
1000000
1500000
2000000
LG9_2_240

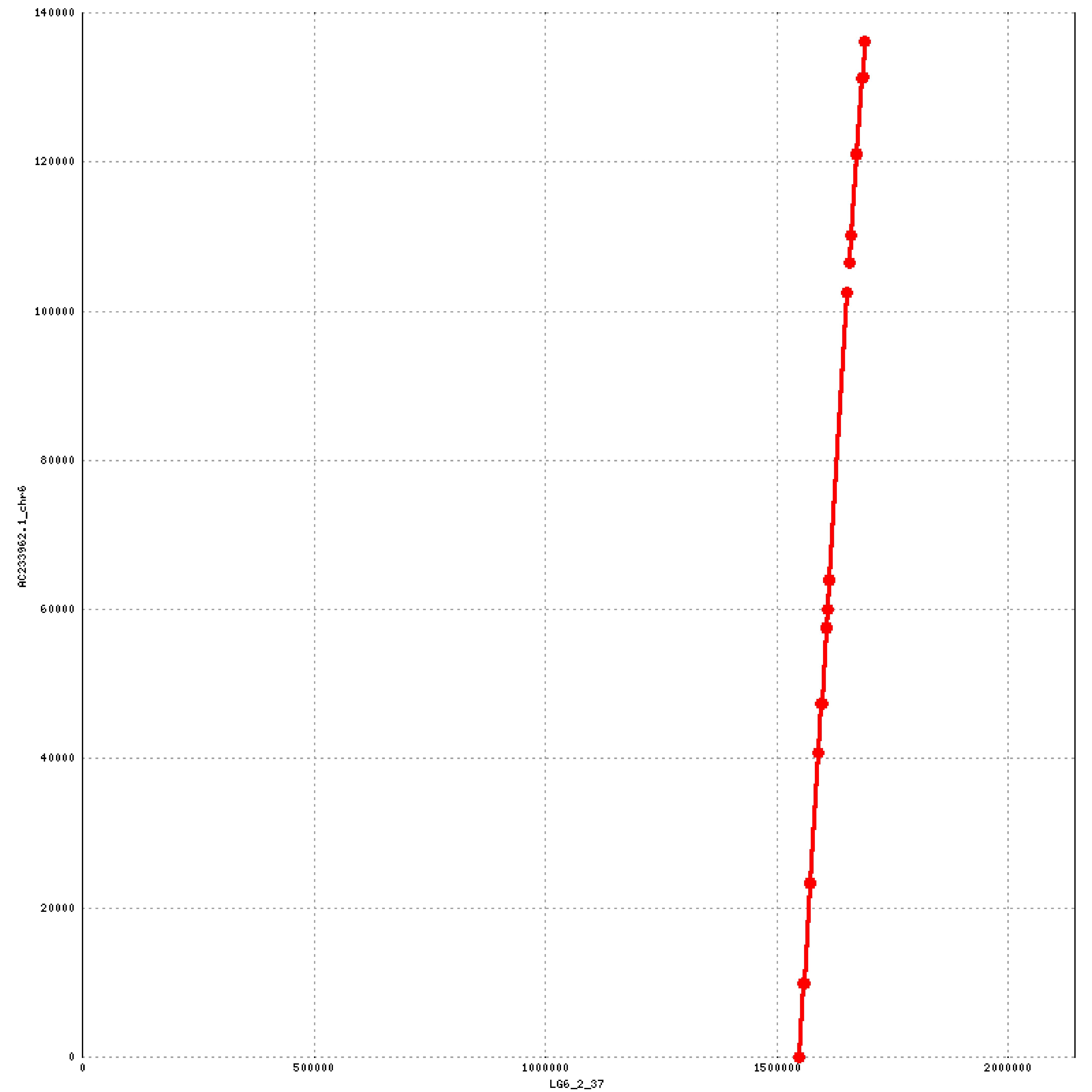

140000
120000
100000
80000
60000
40000
20000
0
AC233962.1_chr6
0
500000
1000000
1500000
2000000
LG6_2_37

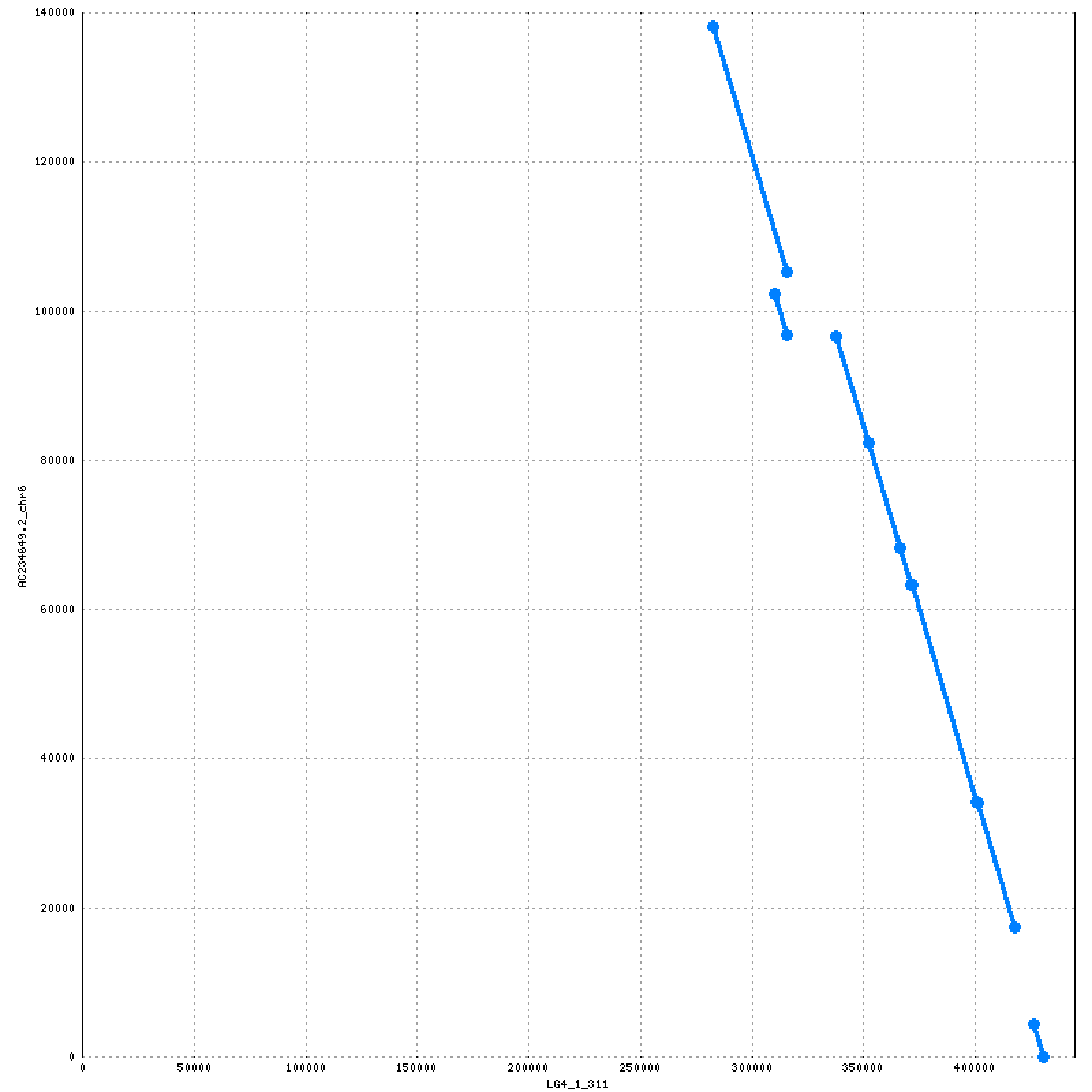

140000
120000
100000
80000
60000
40000
20000
0
AC234649.2_chr6
0
50000
100000
150000
200000
250000
300000
350000
400000
LG4_1_311

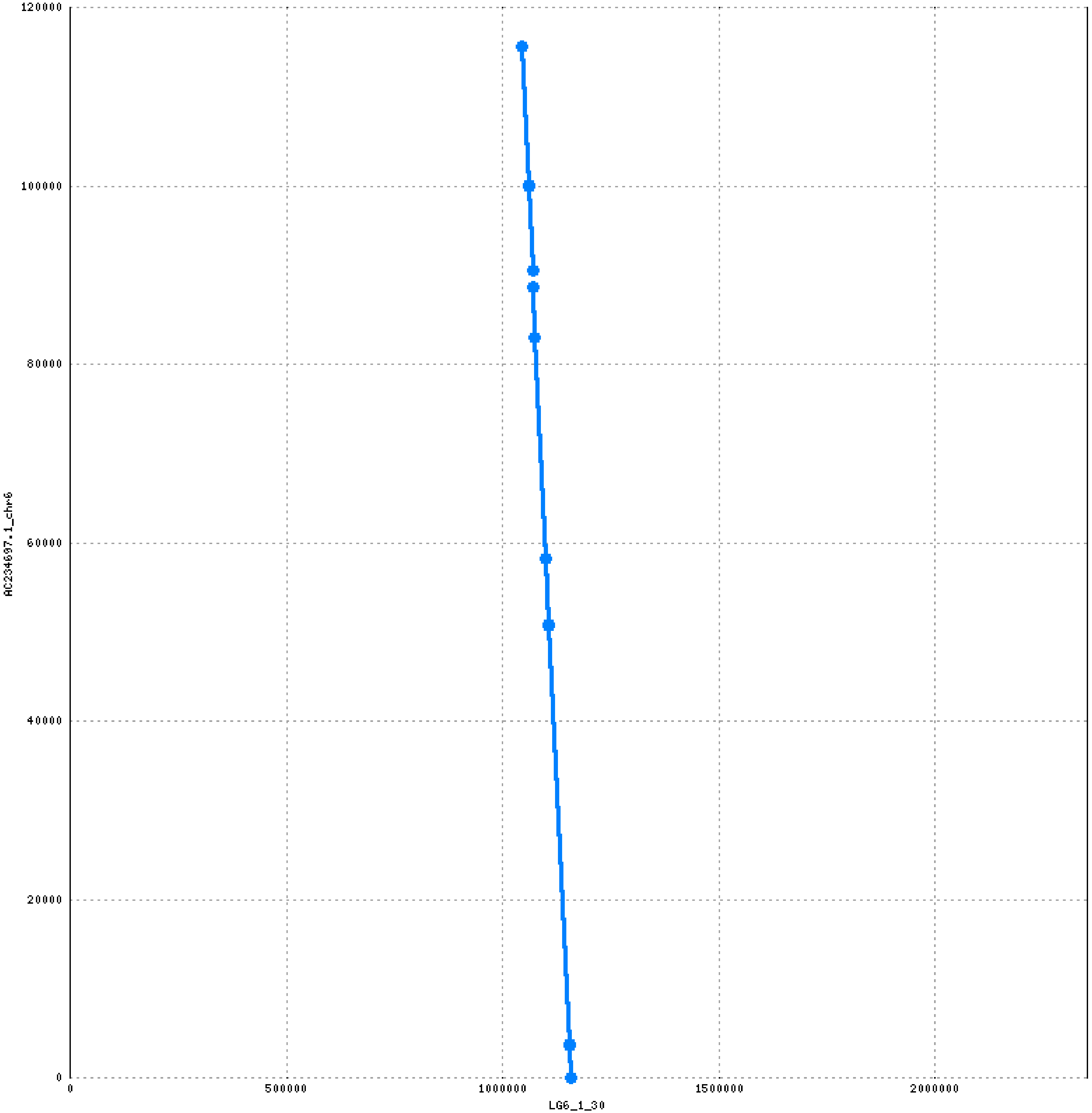

120000
100000
80000
60000
40000
20000
0
AC234697.1_chr6
0
500000
1000000
1500000
2000000
LG6_1_30

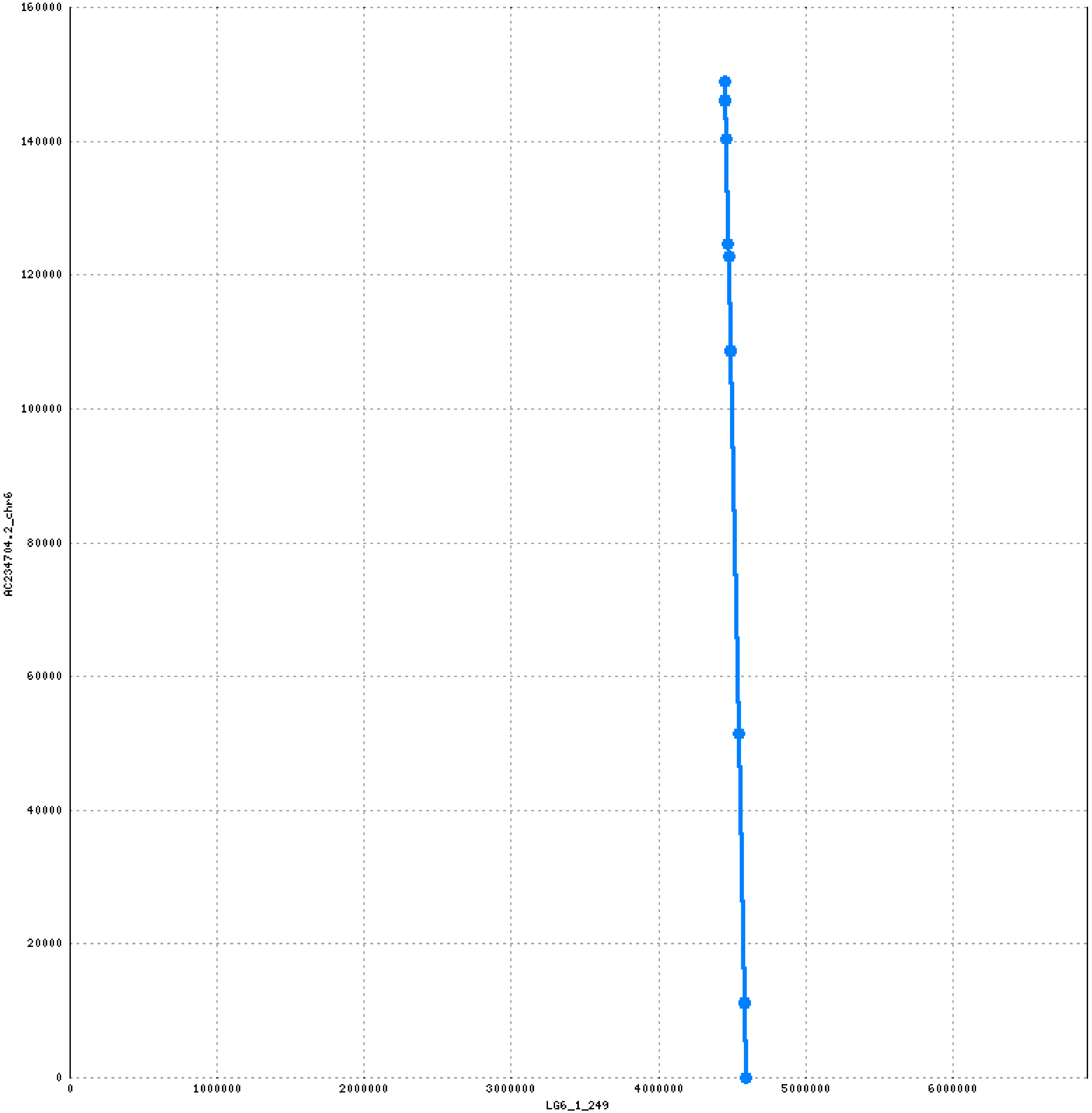

160000
140000
120000
100000
80000
60000
40000
20000
0
AC234704.2_chr6
0
1000000
2000000
3000000
4000000
5000000
6000000
LG6_1_249

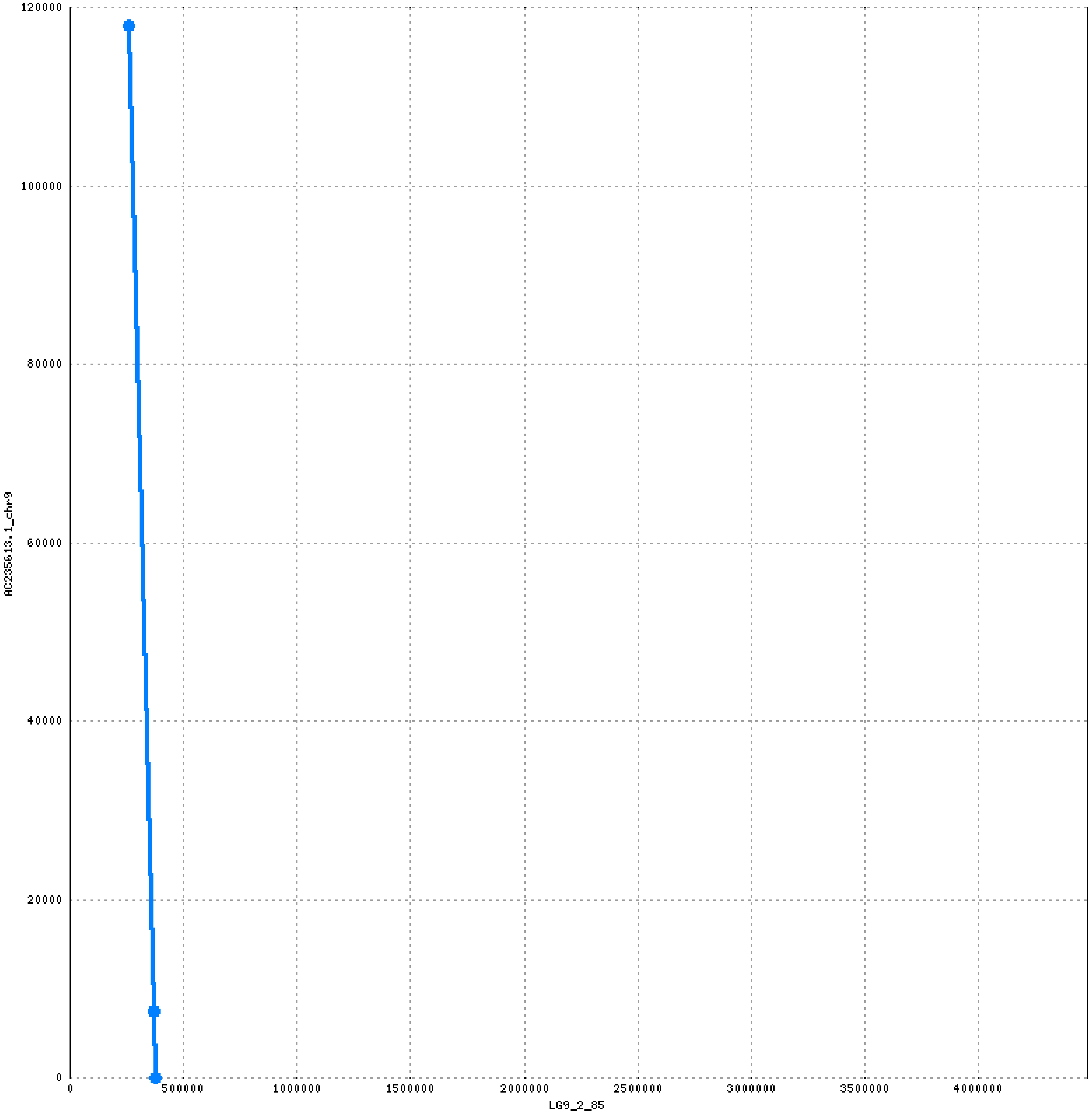

120000
100000
80000
60000
40000
20000
0
AC235613.1_chr9
0
500000
1000000
1500000
2000000
2500000
3000000
3500000
4000000
LG9_2_85

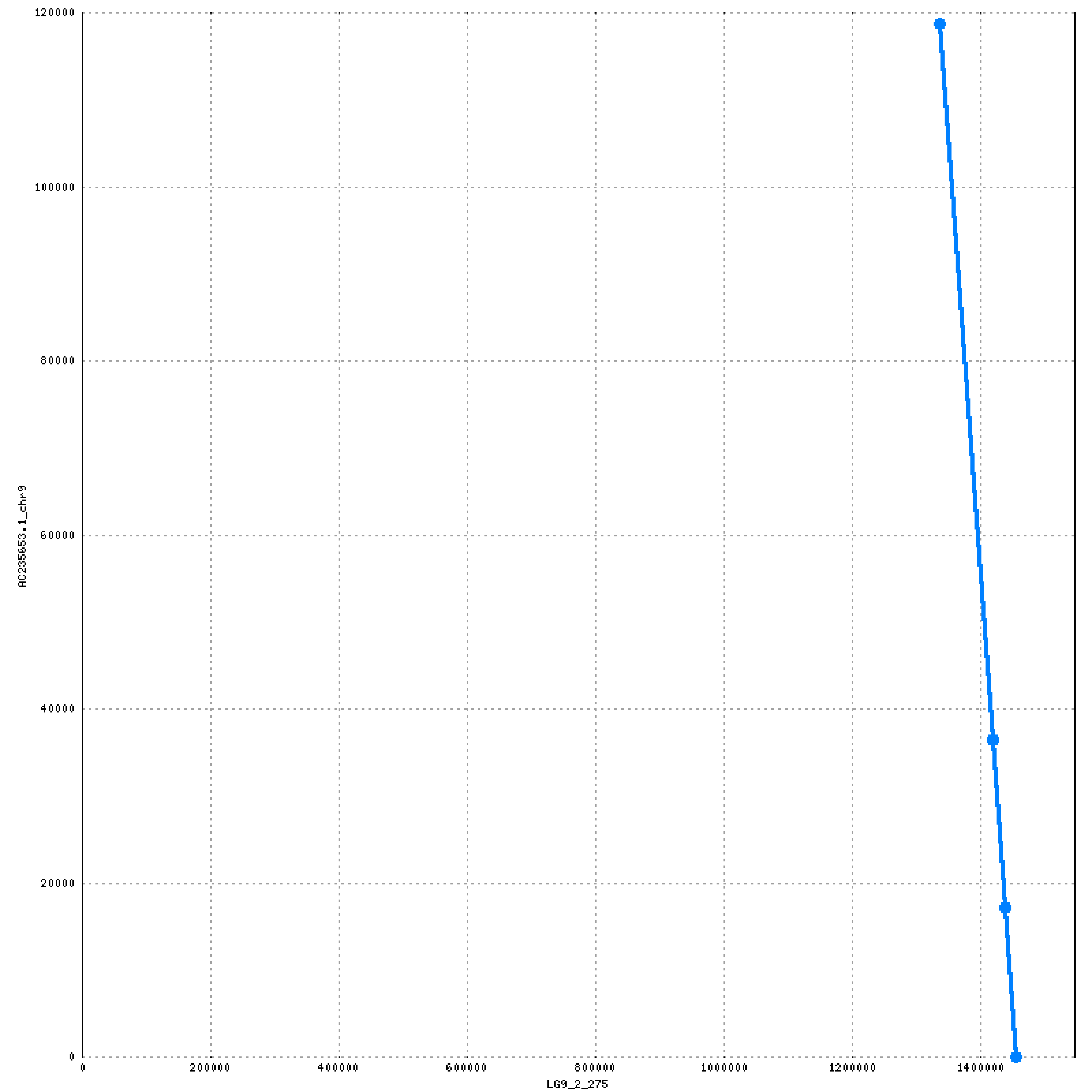

120000
100000
80000
60000
40000
20000
0
AC235653.1_chr9
0
200000
400000
600000
800000
1000000
1200000
1400000
LG9_2_275

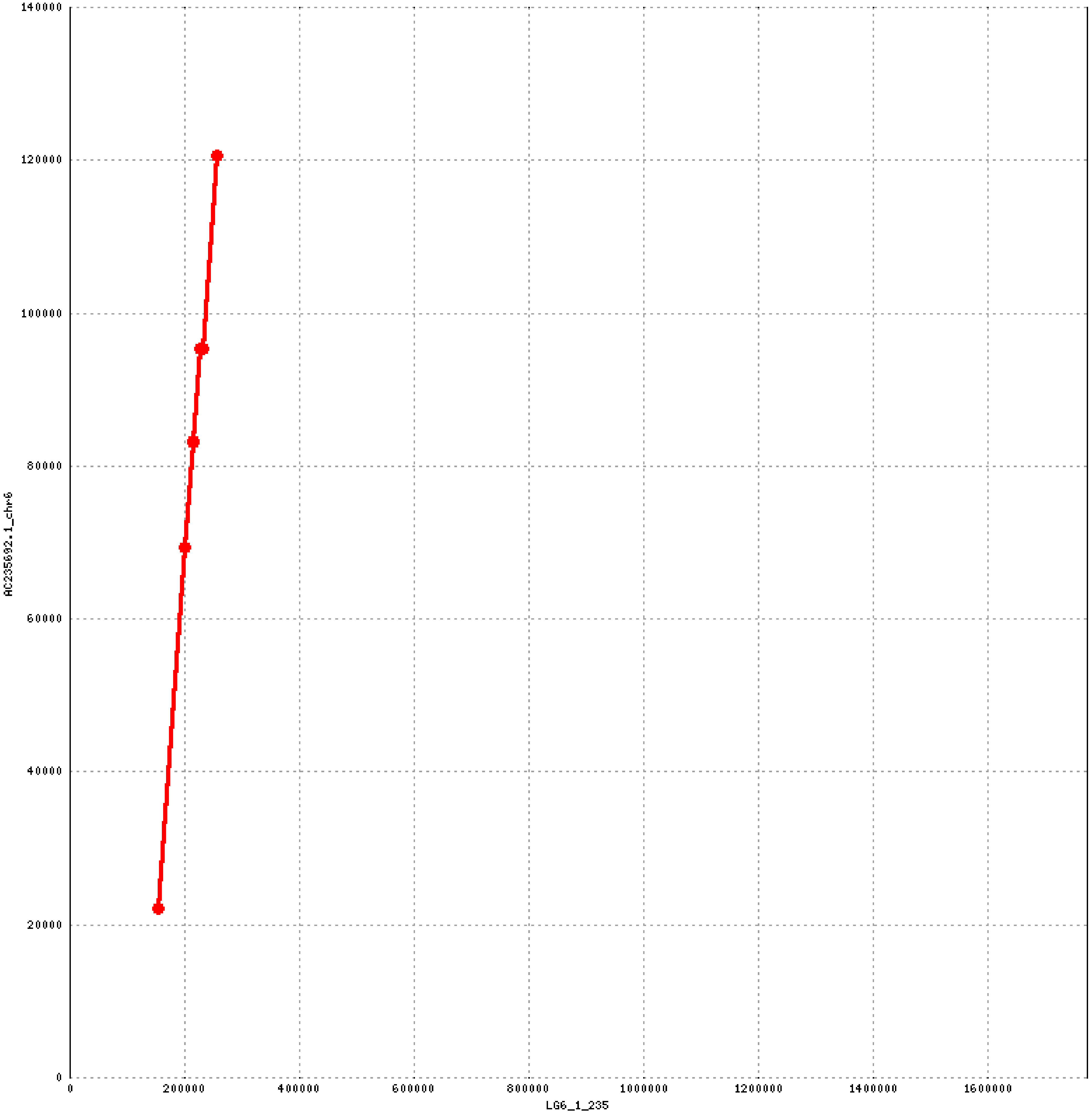
140000
120000
100000
80000
60000
40000
20000
0
AC235692.1_chr6
0
200000
400000
600000
800000
1000000
1200000
1400000
1600000
LG6_1_235

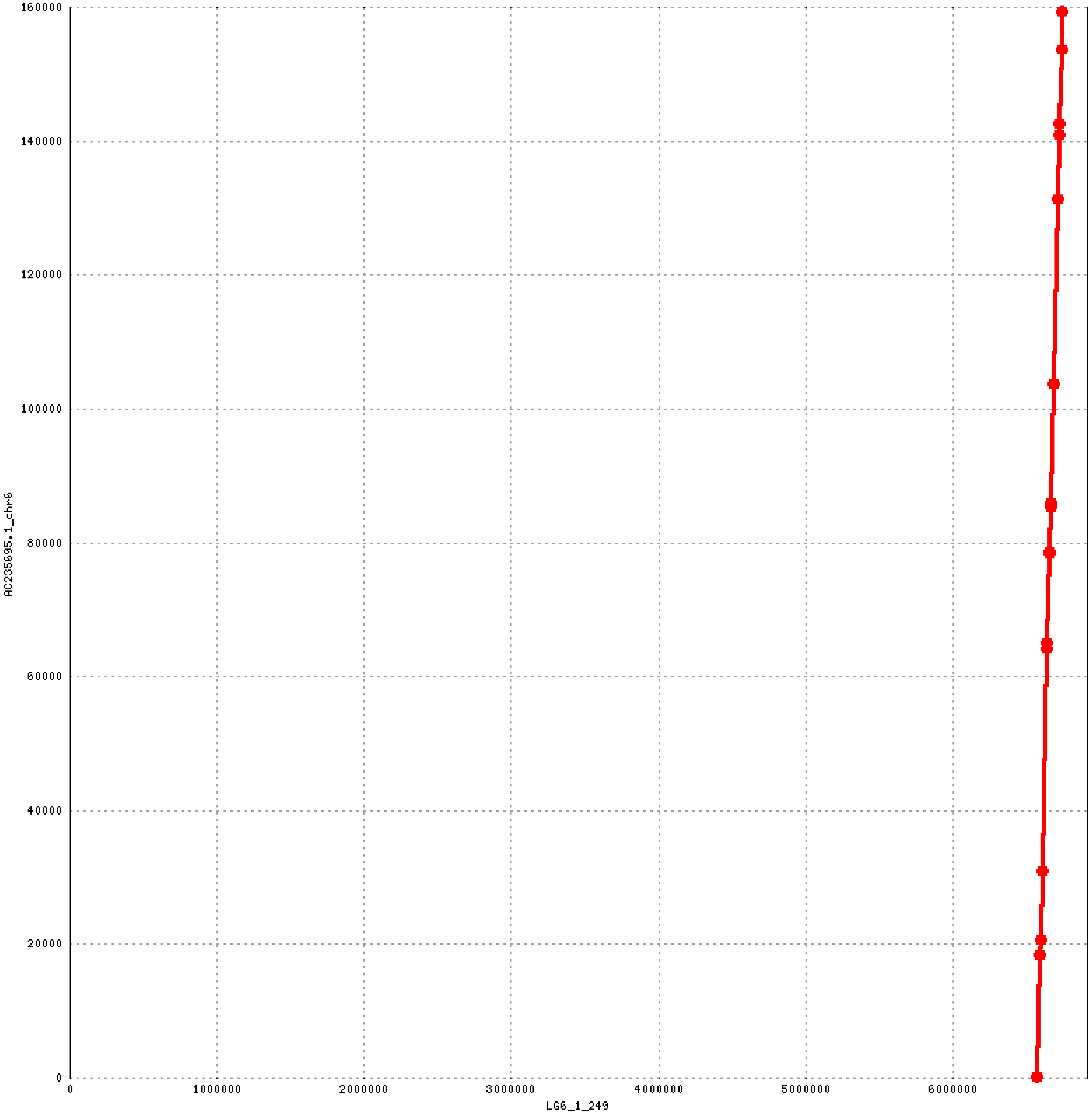

160000
140000
120000
100000
80000
60000
40000
20000
0
AC235695.1_chr6
0
1000000
2000000
3000000
4000000
5000000
6000000
LG6_1_249

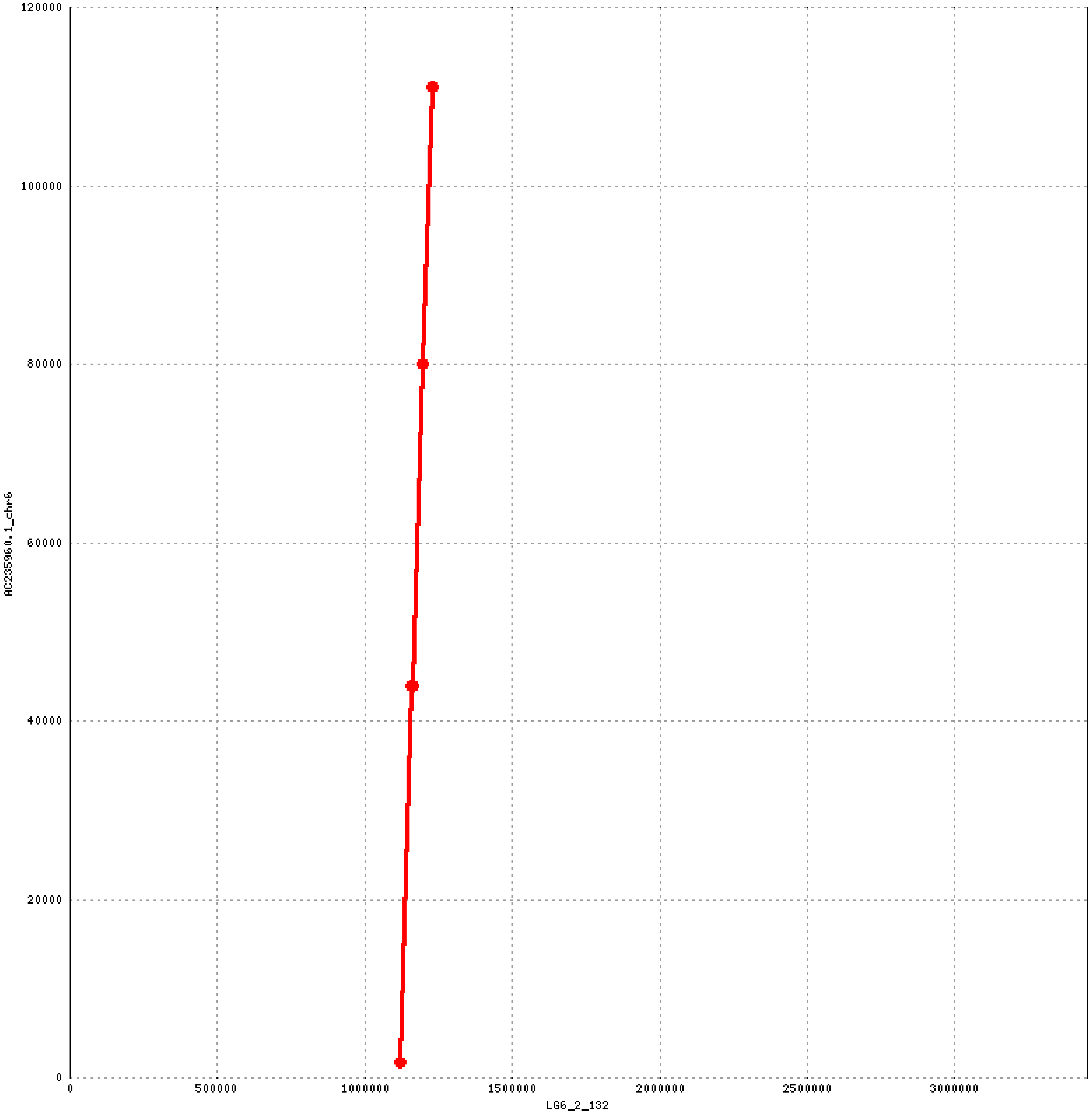

120000
100000
80000
60000
40000
20000
0
AC235960.1_chr6
0
500000
1000000
1500000
2000000
2500000
3000000
LG6_2_132

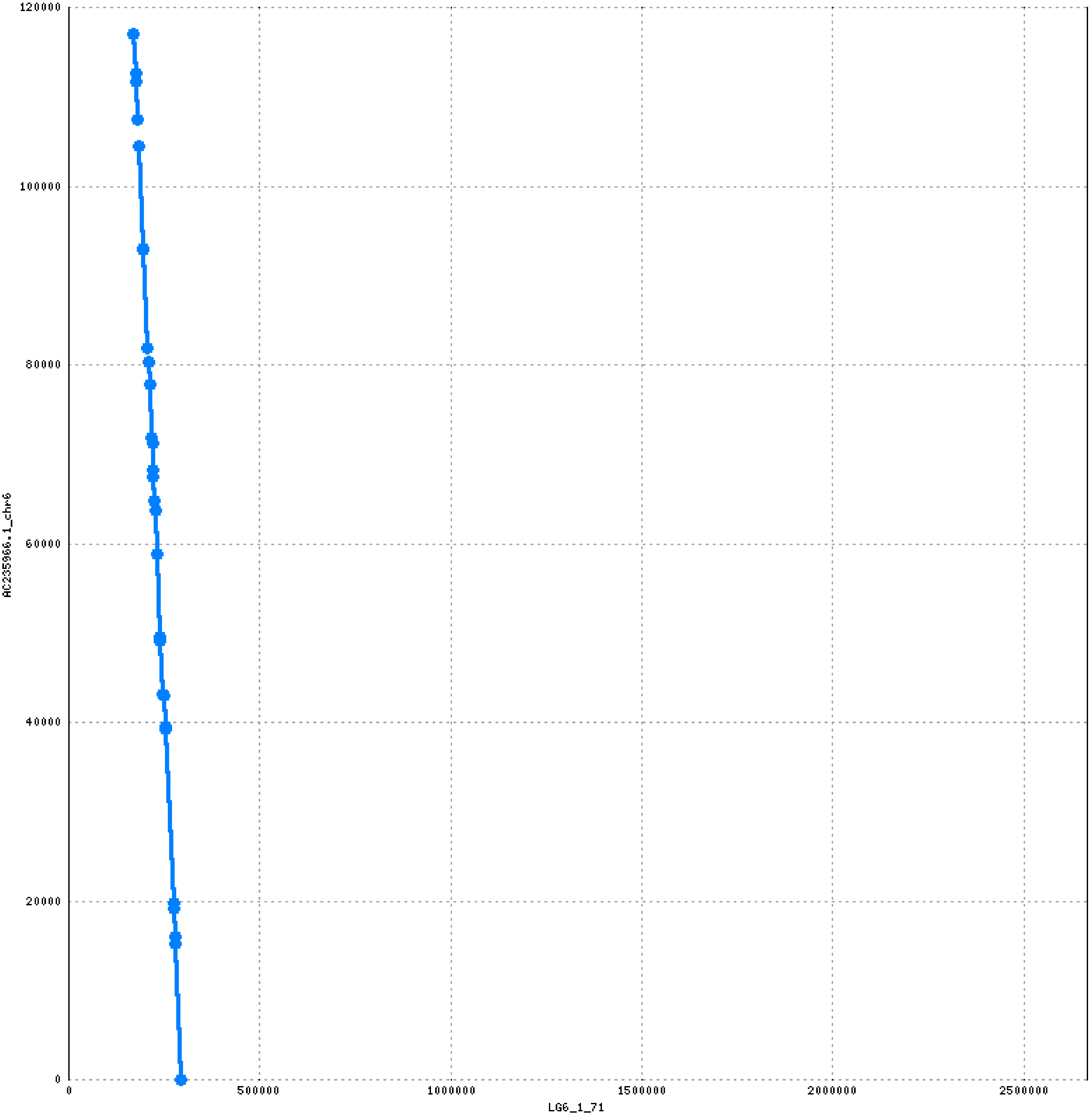
120000
100000
80000
60000
40000
20000
0
AC235966.1_chr6
0
500000
1000000
1500000
2000000
2500000
LG6_1_71

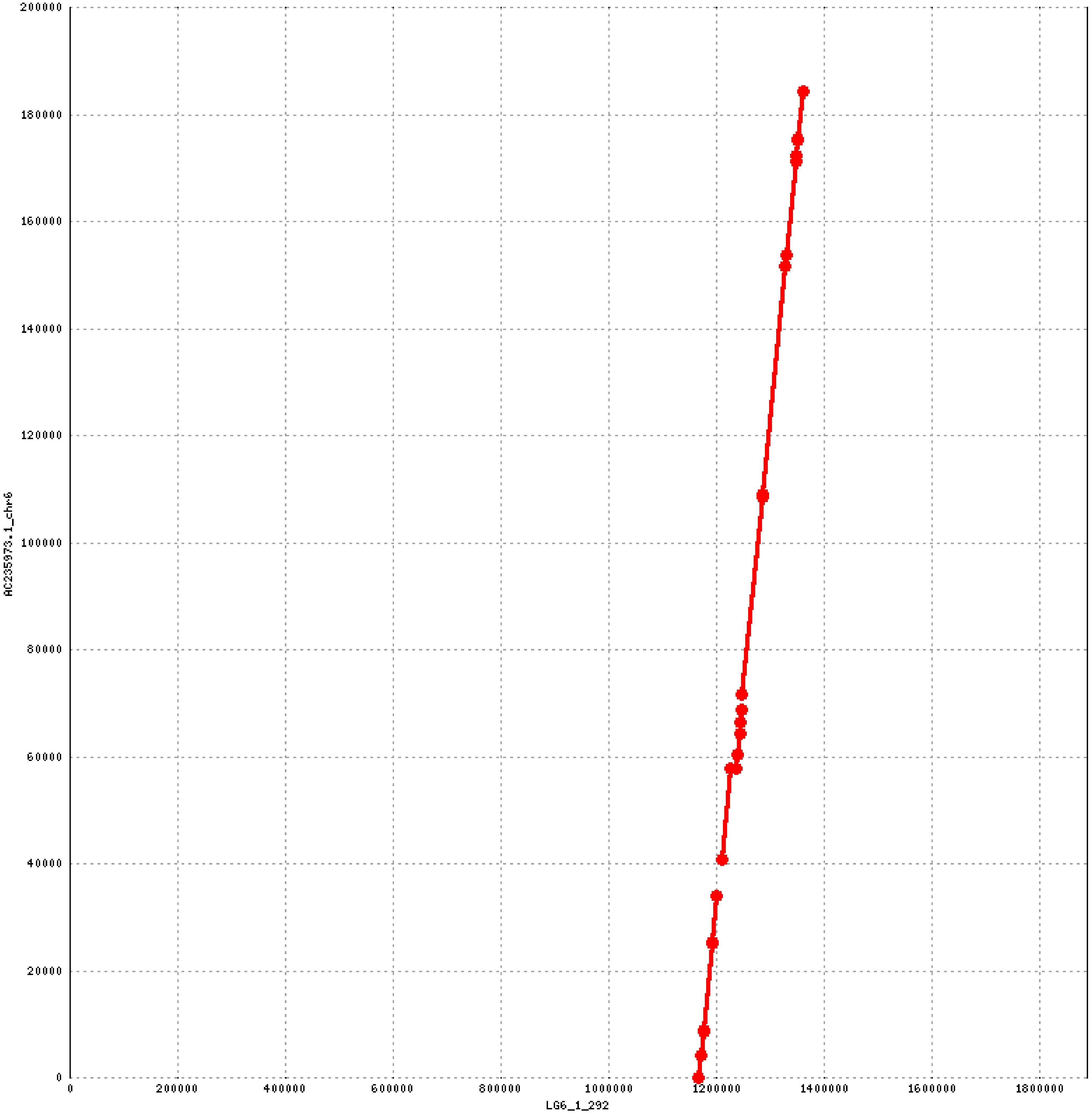

200000
180000
160000
140000
120000
100000
80000
60000
40000
20000
0
AC235973.1_chr6
0
200000
400000
600000
800000
1000000
1200000
1400000
1600000
1800000
LG6_1_292

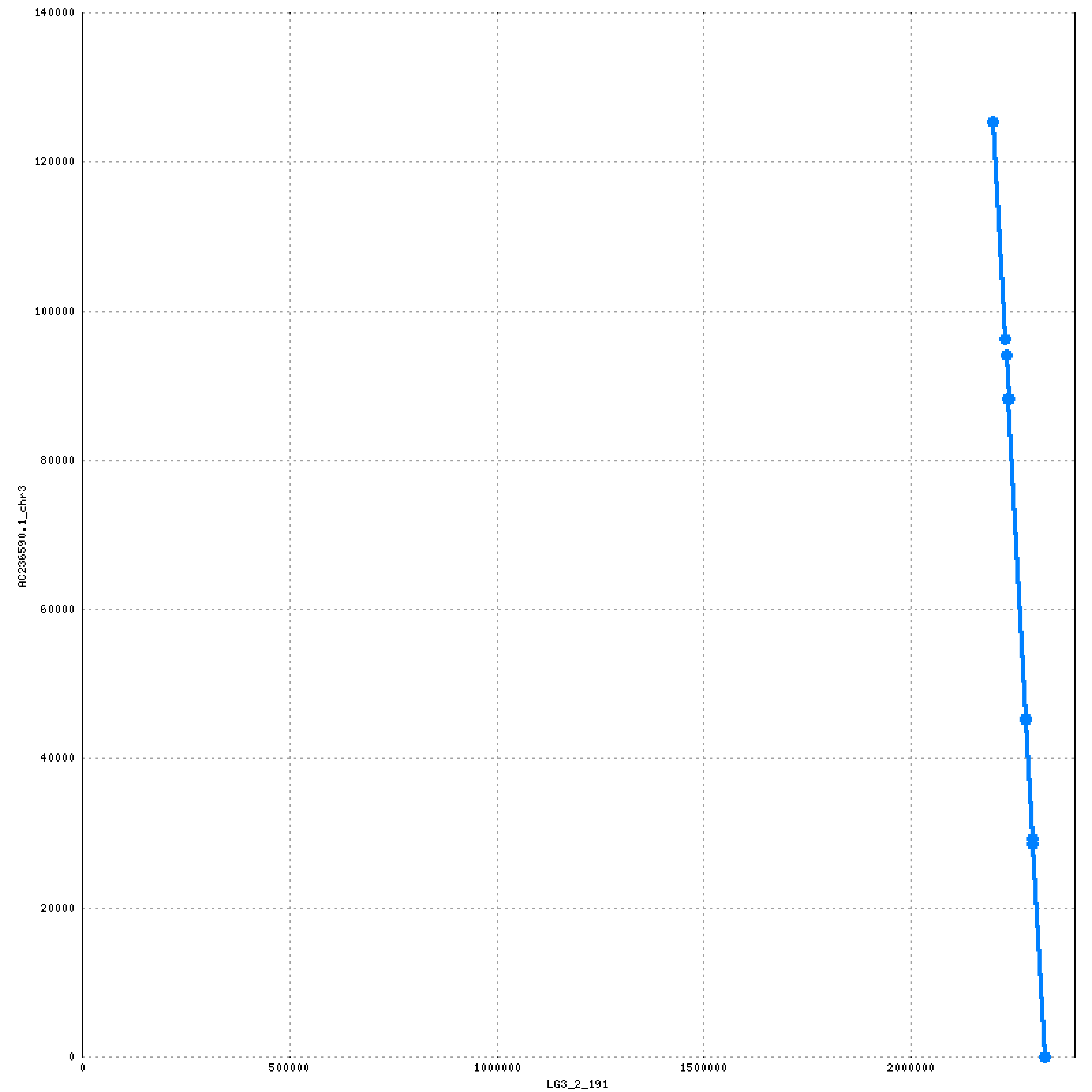

140000
120000
100000
80000
60000
40000
20000
0
AC236590.1_chr3
0
500000
1000000
1500000
2000000
LG3_2_191

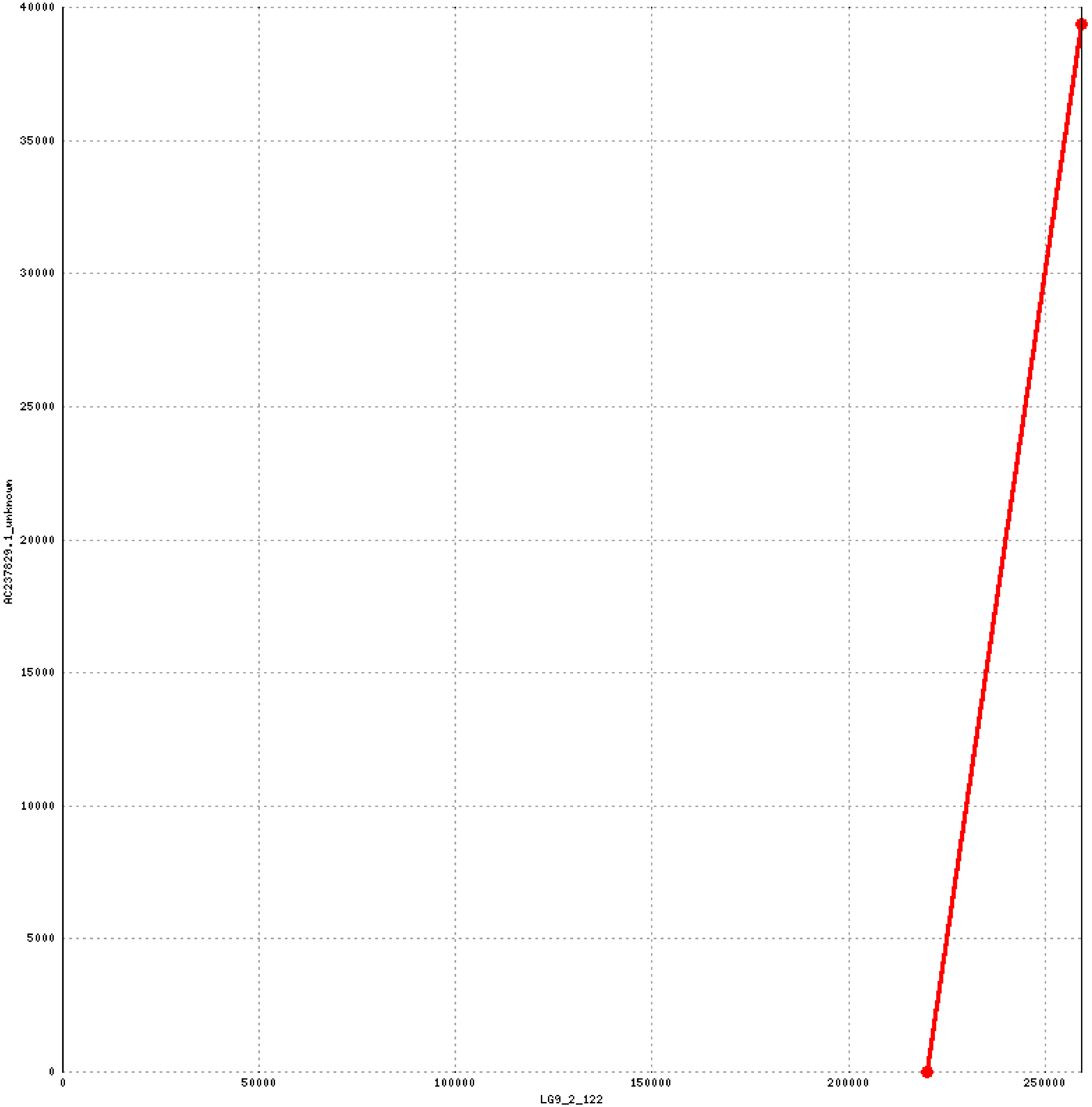

40000
35000
30000
25000
20000
15000
10000
5000
0
AC237829.1_unknown
0
50000
100000
150000
200000
250000
LG9_2_122

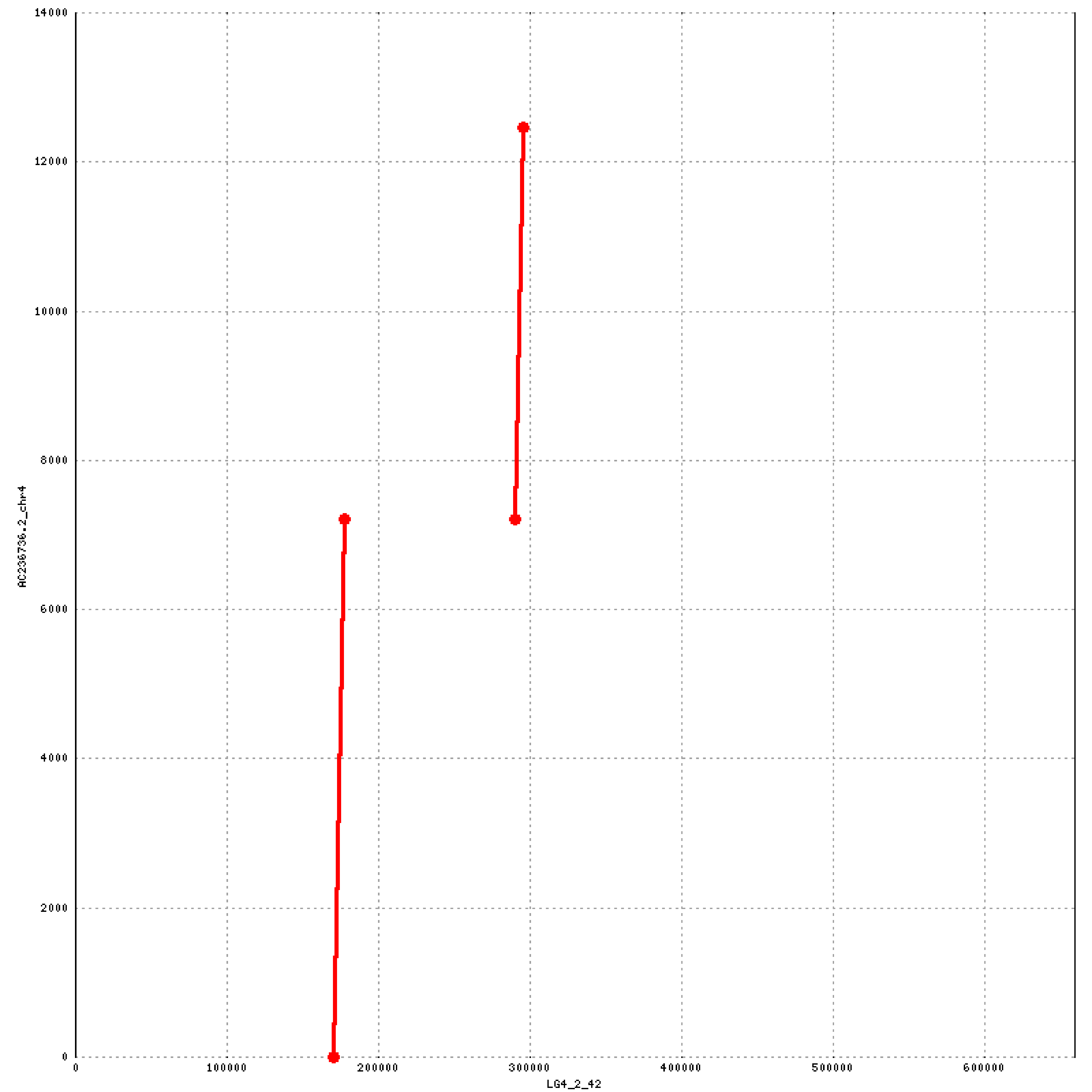

14000
12000
10000
8000
6000
4000
2000
0
0
100000
200000
300000
400000
500000
600000
AC236736.2_chr4
LG4_2_42

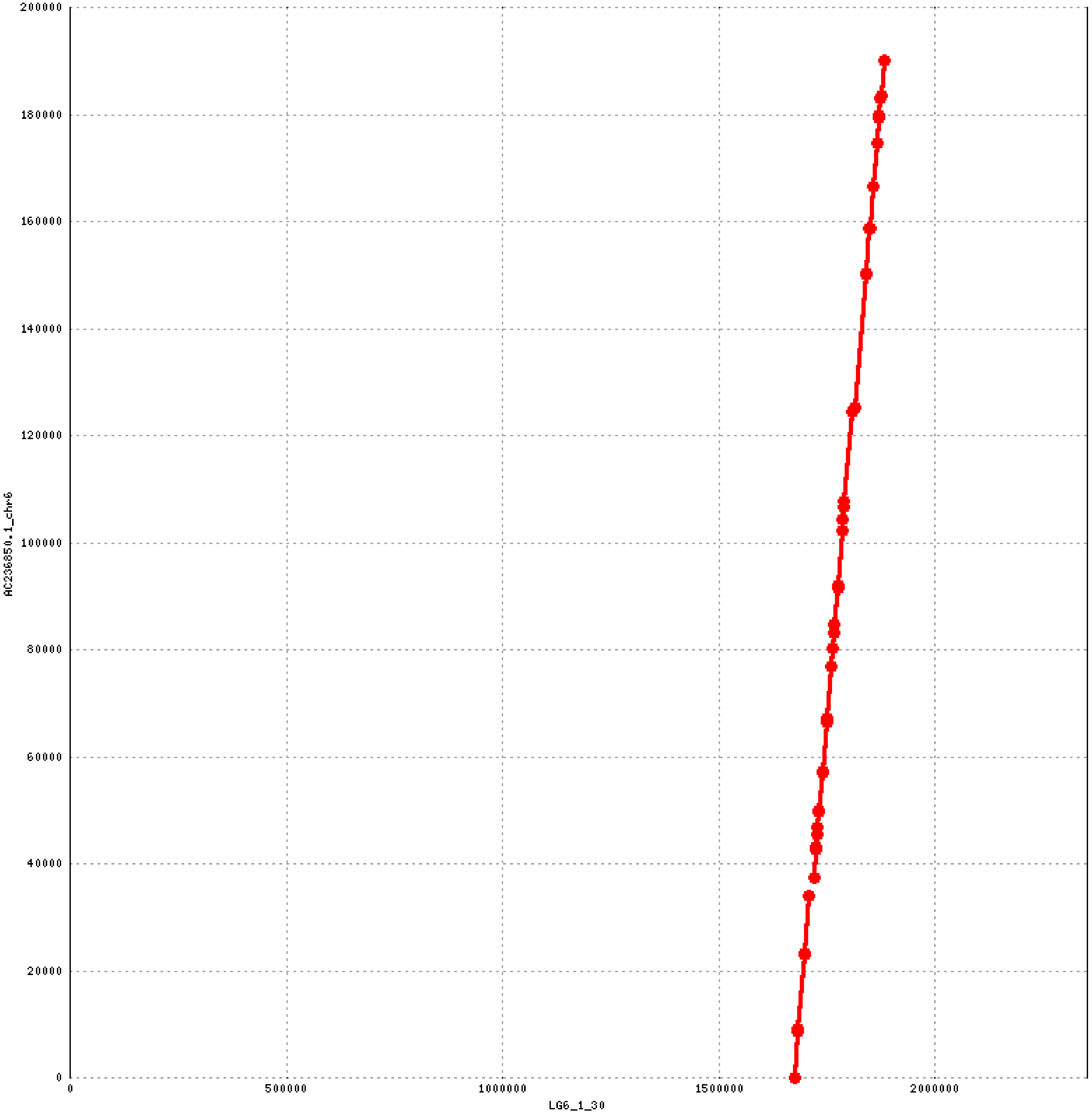
AC236850.1_chr6
LG6_1_30
0
20000
40000
60000
80000
100000
120000
140000
160000
180000
200000
500000
1000000
1500000
2000000

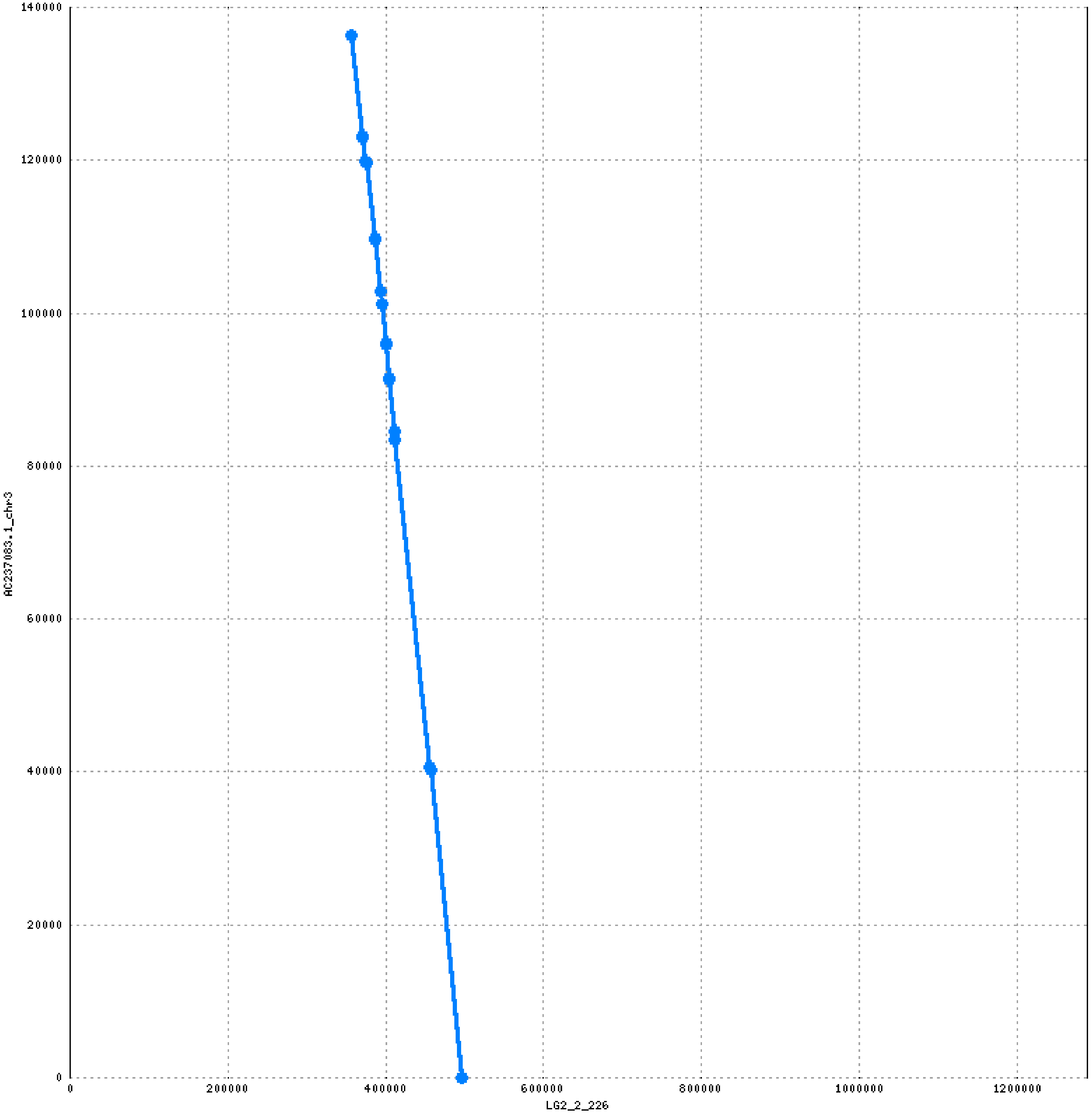

140000
120000
100000
80000
60000
40000
20000
0
AC237083.1_chr3
0
200000
400000
600000
800000
1000000
1200000
LG2_2_226

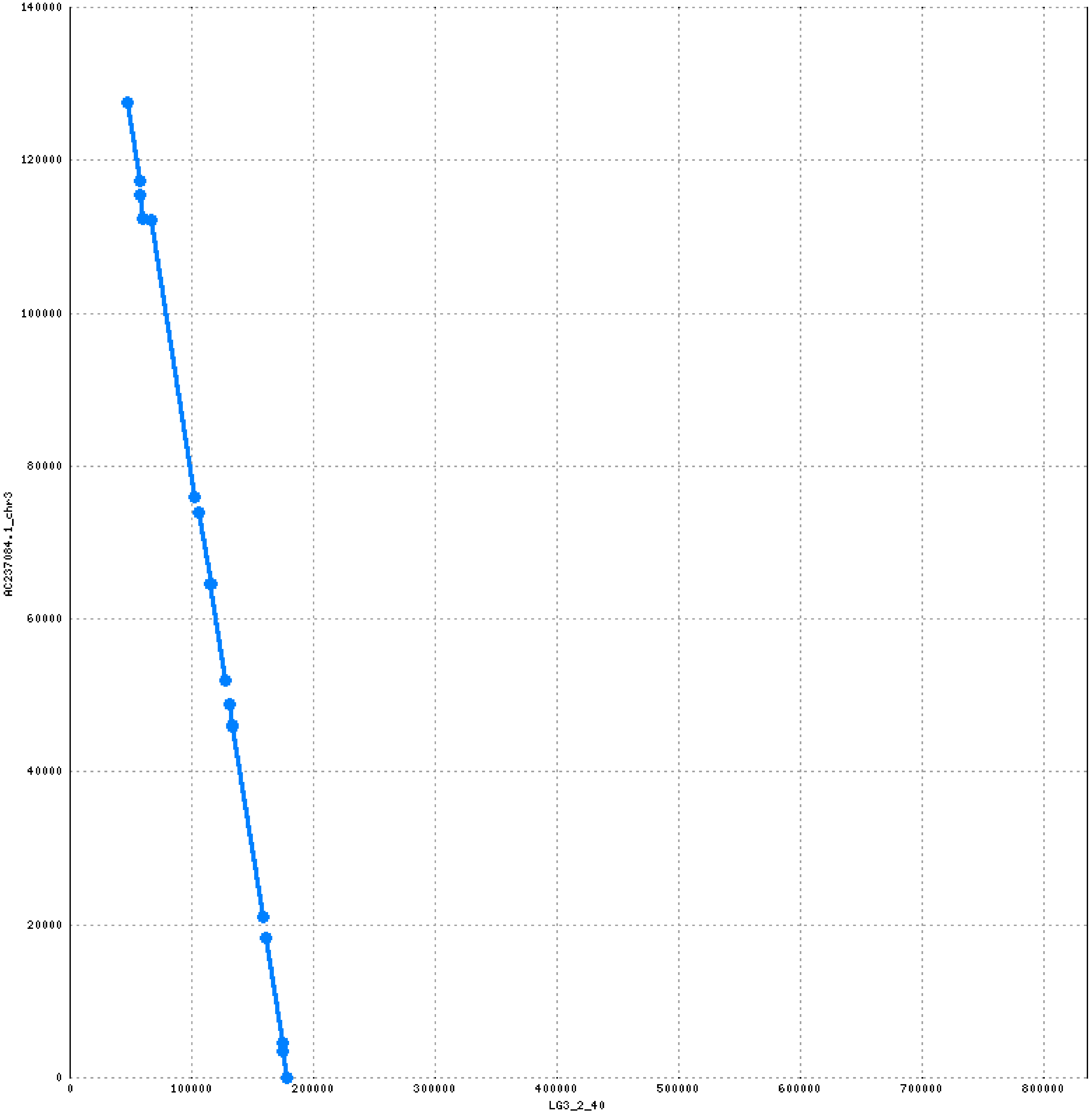
140000
120000
100000
80000
60000
40000
20000
0
AC237084.1_chr3
0
100000
200000
300000
400000
500000
600000
700000
800000
LG3_2_40

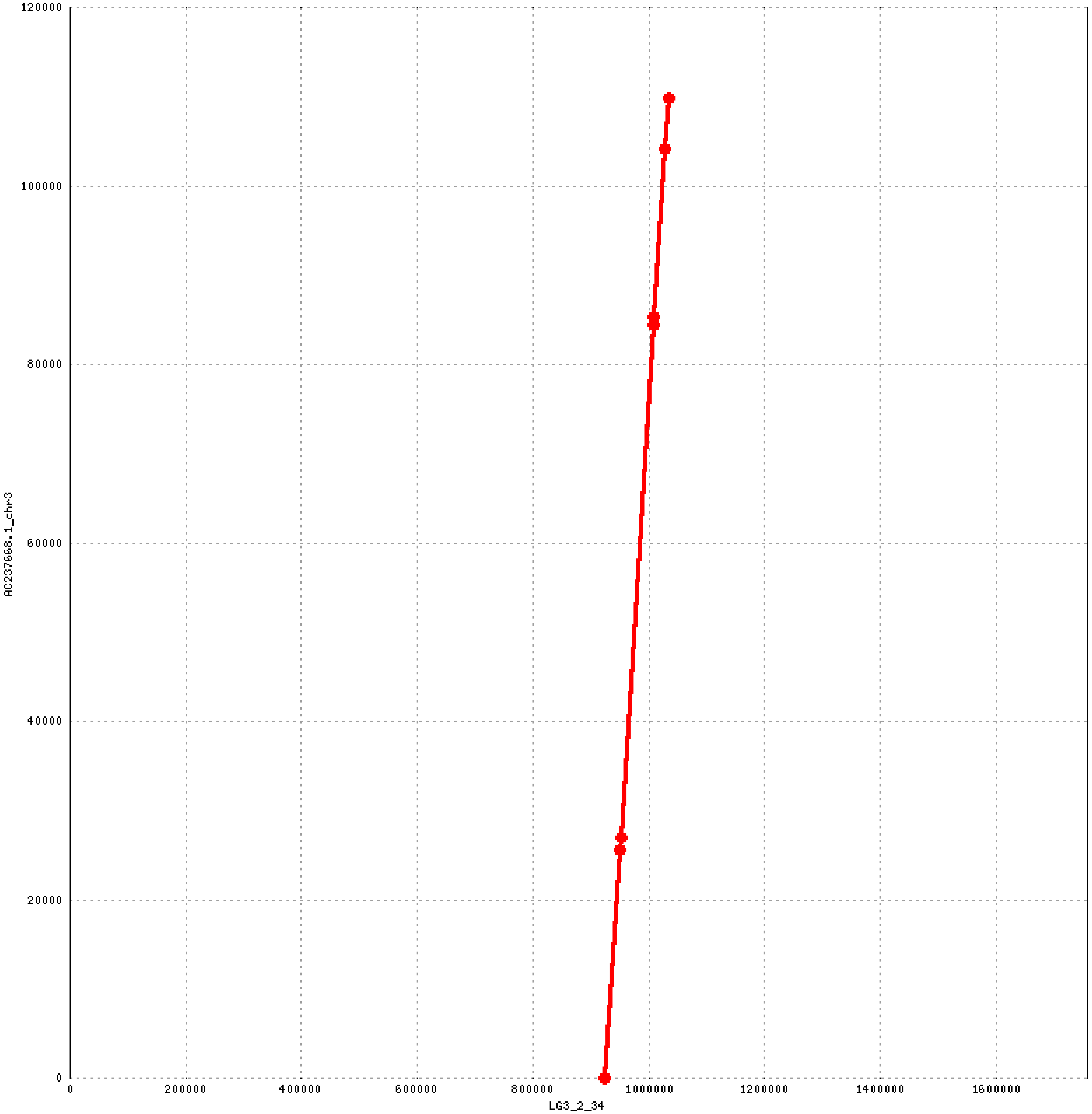

120000
100000
80000
60000
40000
20000
0
AC237668.1_chr3
0
200000
400000
600000
800000
1000000
1200000
1400000
1600000
LG3_2_34

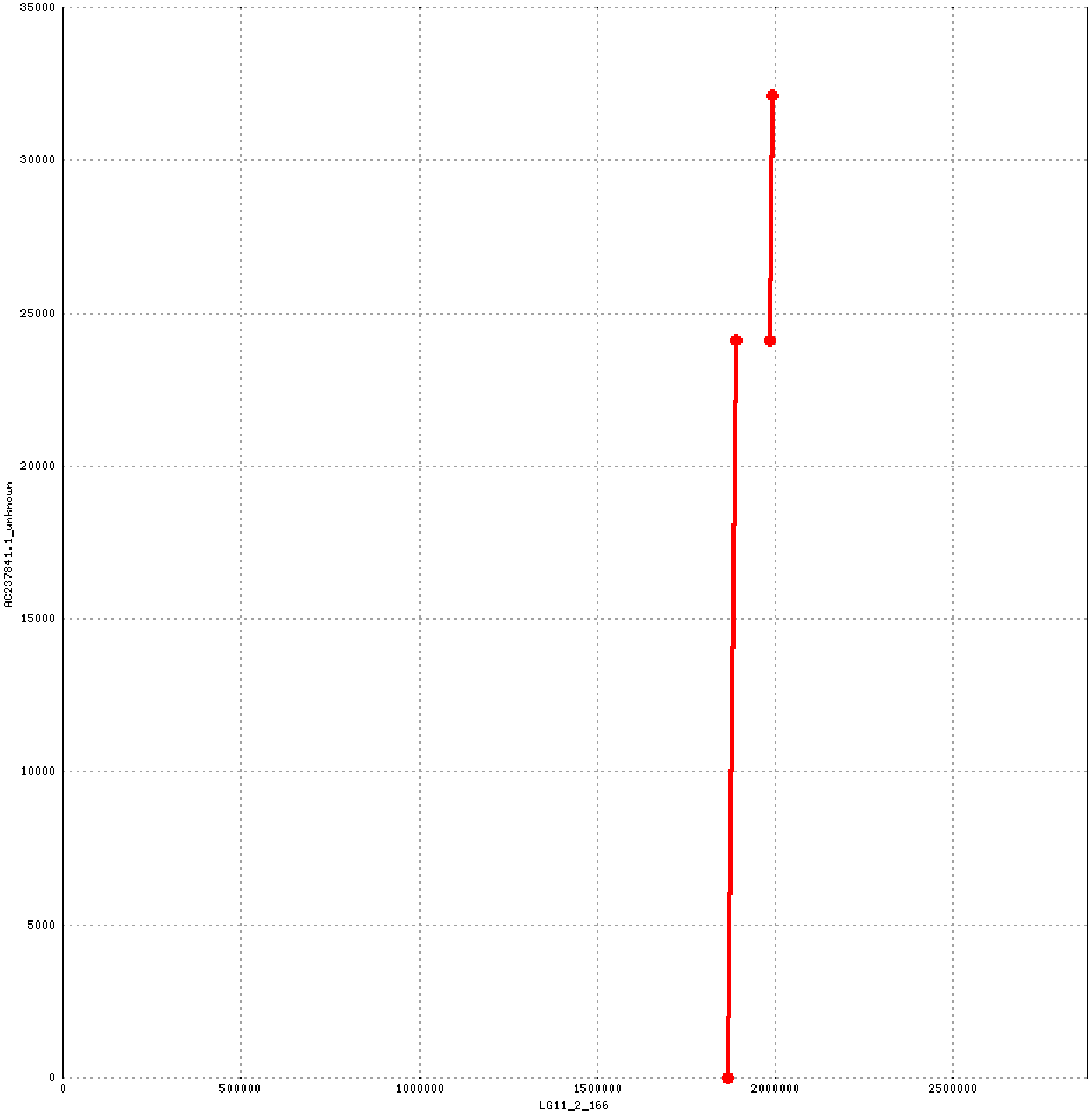
35000
30000
25000
20000
15000
10000
5000
0
AC237841.1_unknown
0
500000
1000000
1500000
2000000
2500000
LG11_2_166

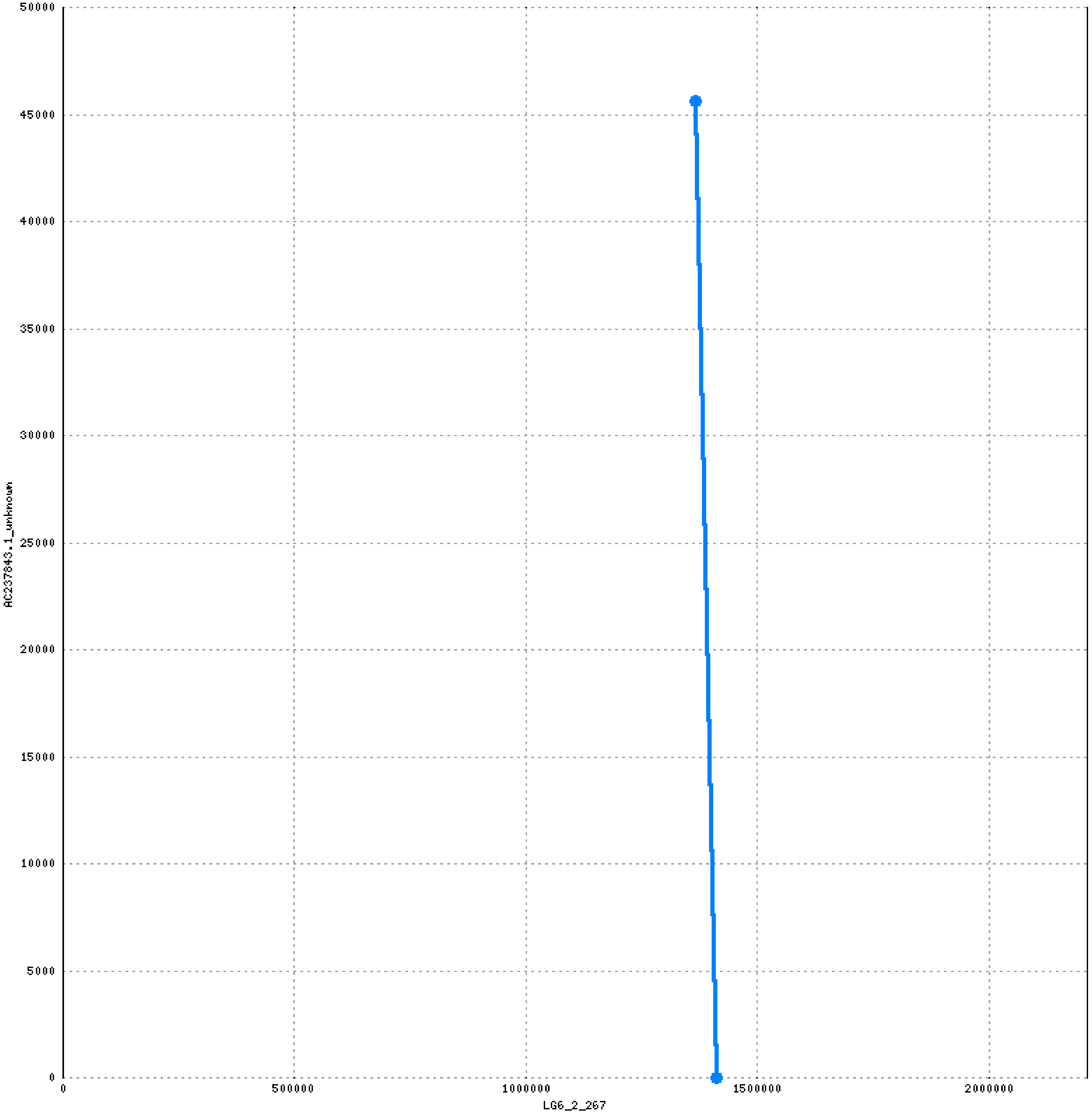

50000
45000
40000
35000
30000
25000
20000
15000
10000
5000
0
AC237843.1_unknown
0
500000
1000000
1500000
2000000
LG6_2_267

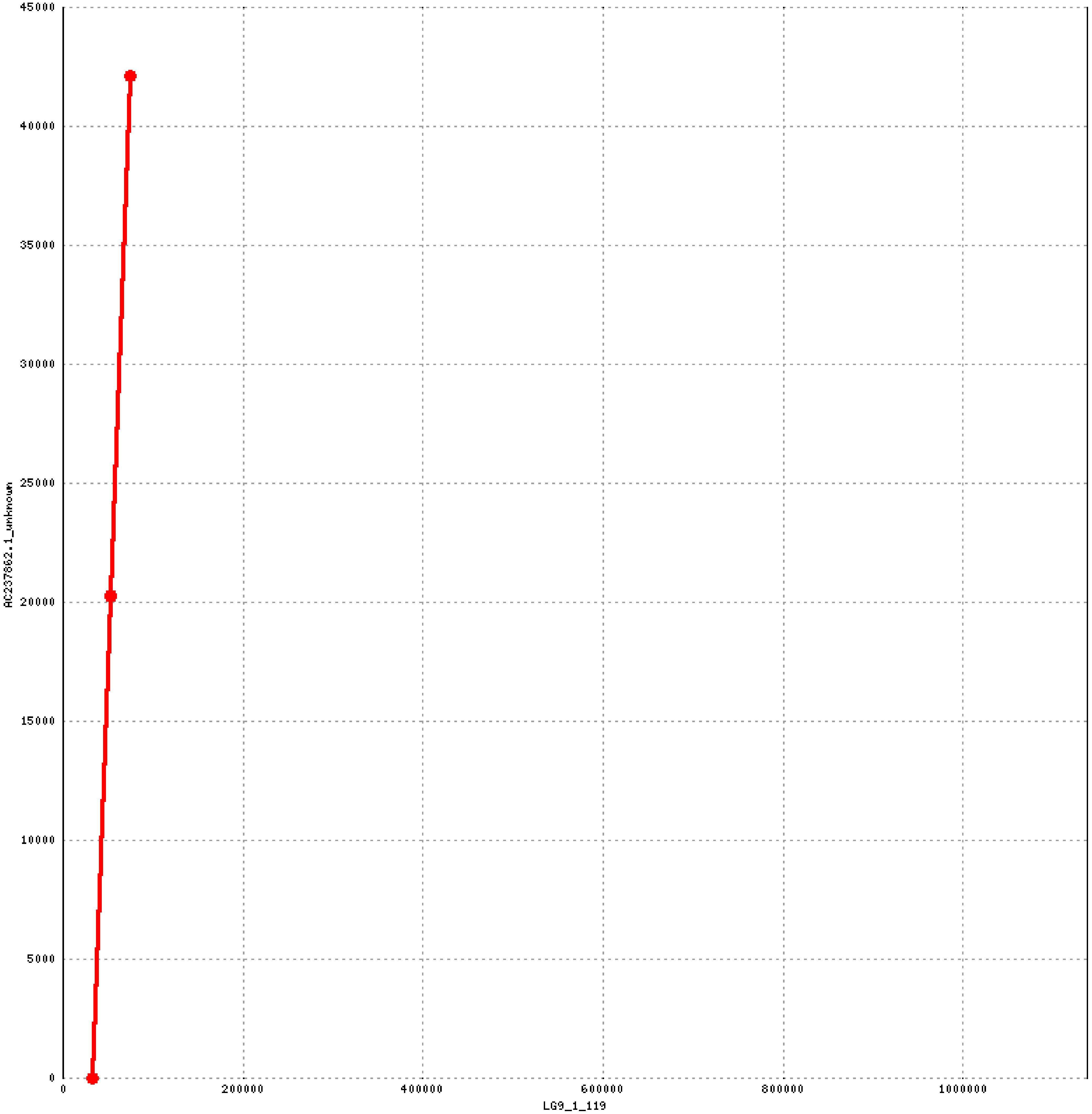
45000
40000
35000
30000
25000
20000
15000
10000
5000
0
AC237862.1_unknown
0
200000
400000
600000
800000
1000000
LG9_1_119

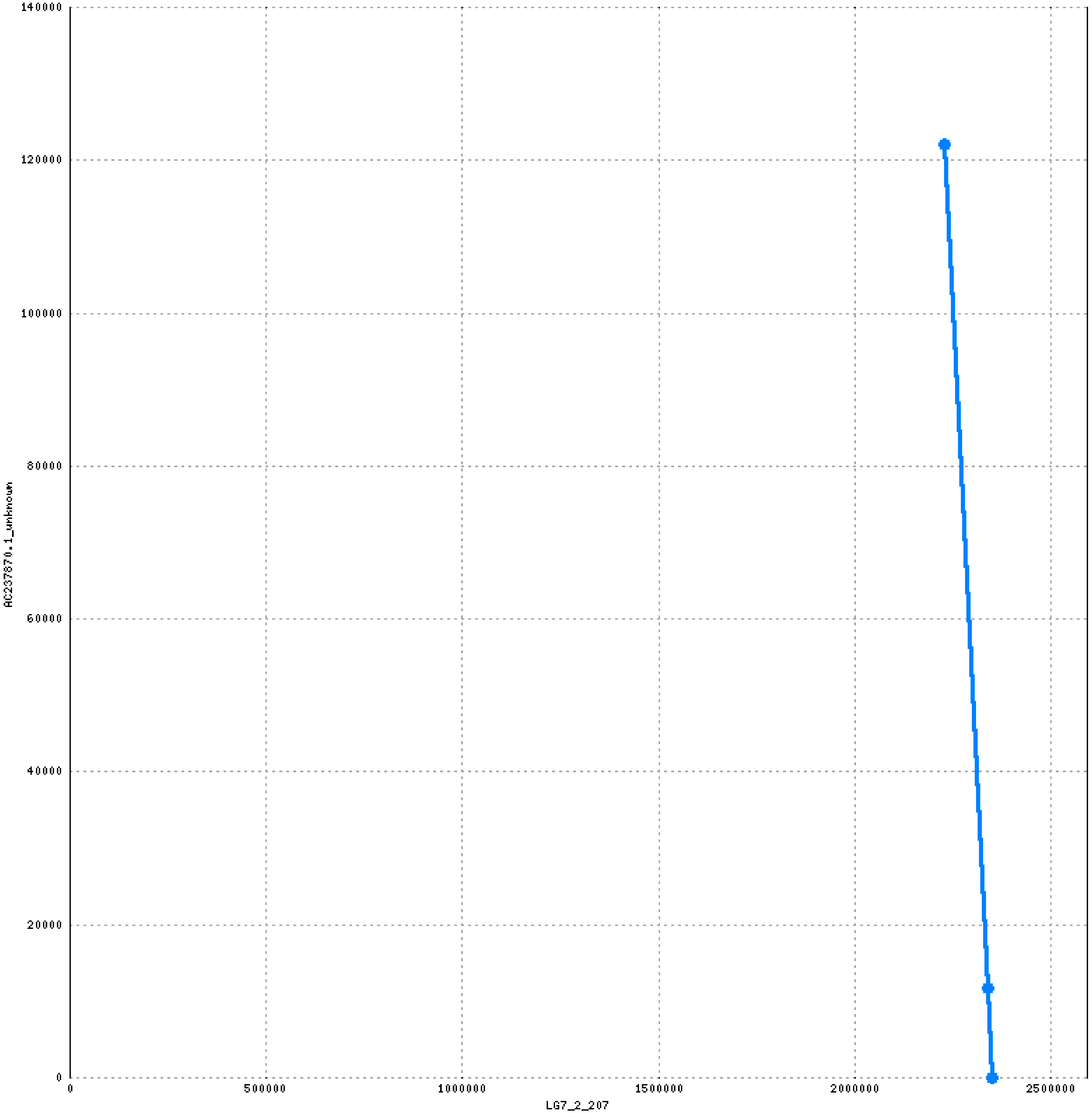
140000
120000
100000
80000
60000
40000
20000
0
AC237870.1_unknown
0
500000
1000000
1500000
2000000
2500000
LG7_2_207

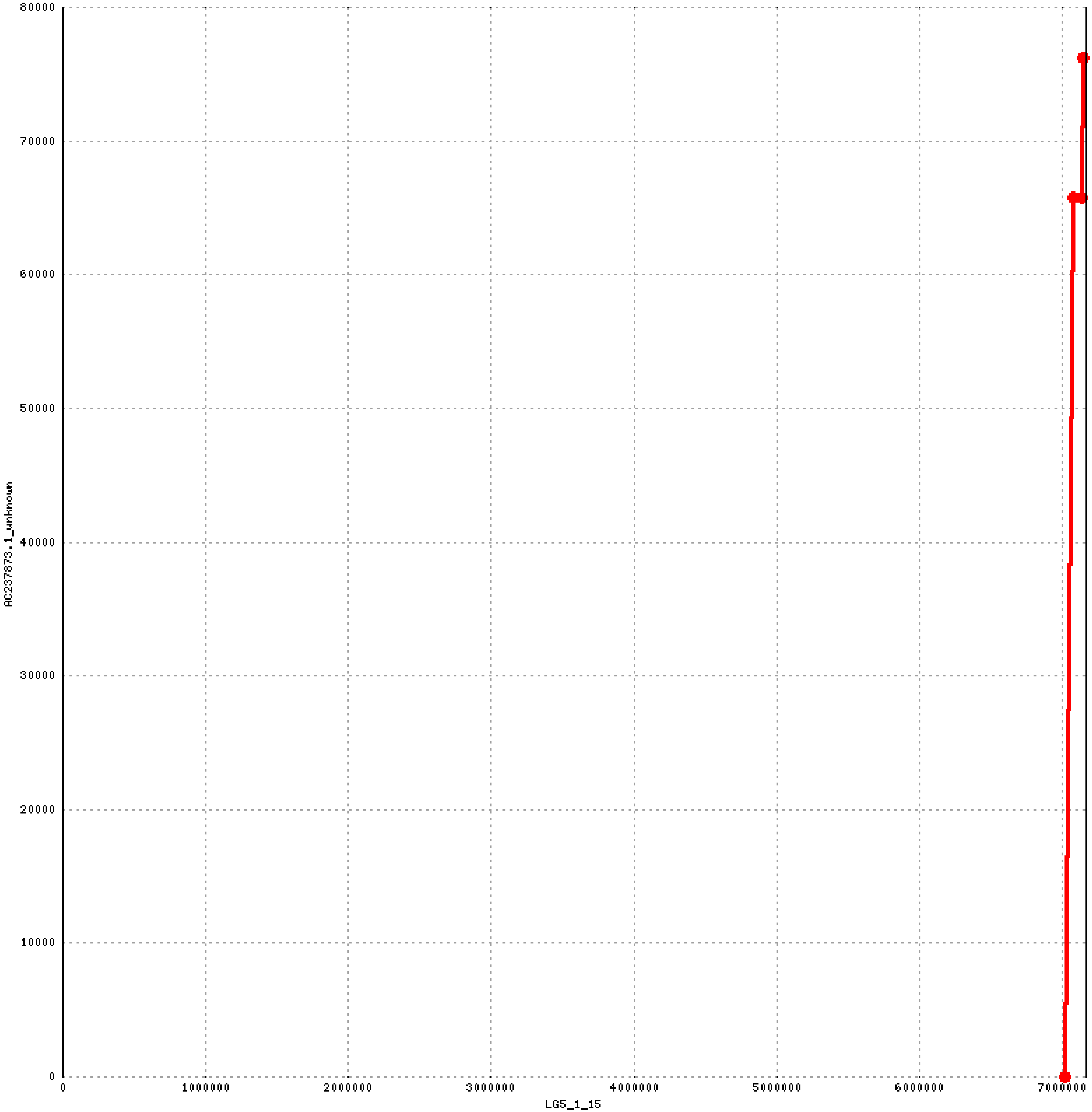
AC237873.1_unknown
LG5_1_15
0
10000
20000
30000
40000
50000
60000
70000
80000
1000000
2000000
3000000
4000000
5000000
6000000
7000000

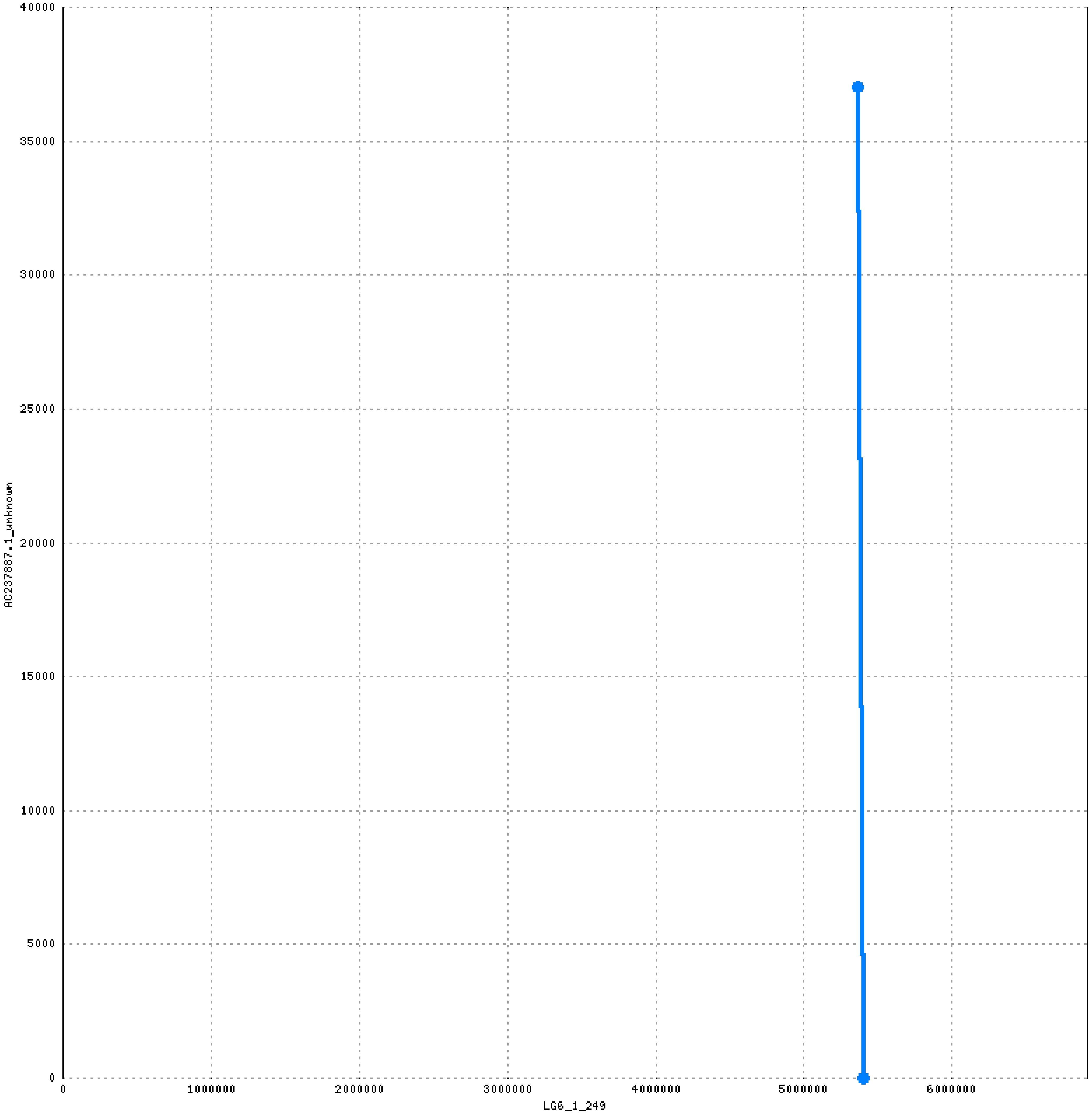
40000
35000
30000
25000
20000
15000
10000
5000
0
AC237887.1_unknown
0
1000000
2000000
3000000
4000000
5000000
6000000
LG6_1_249

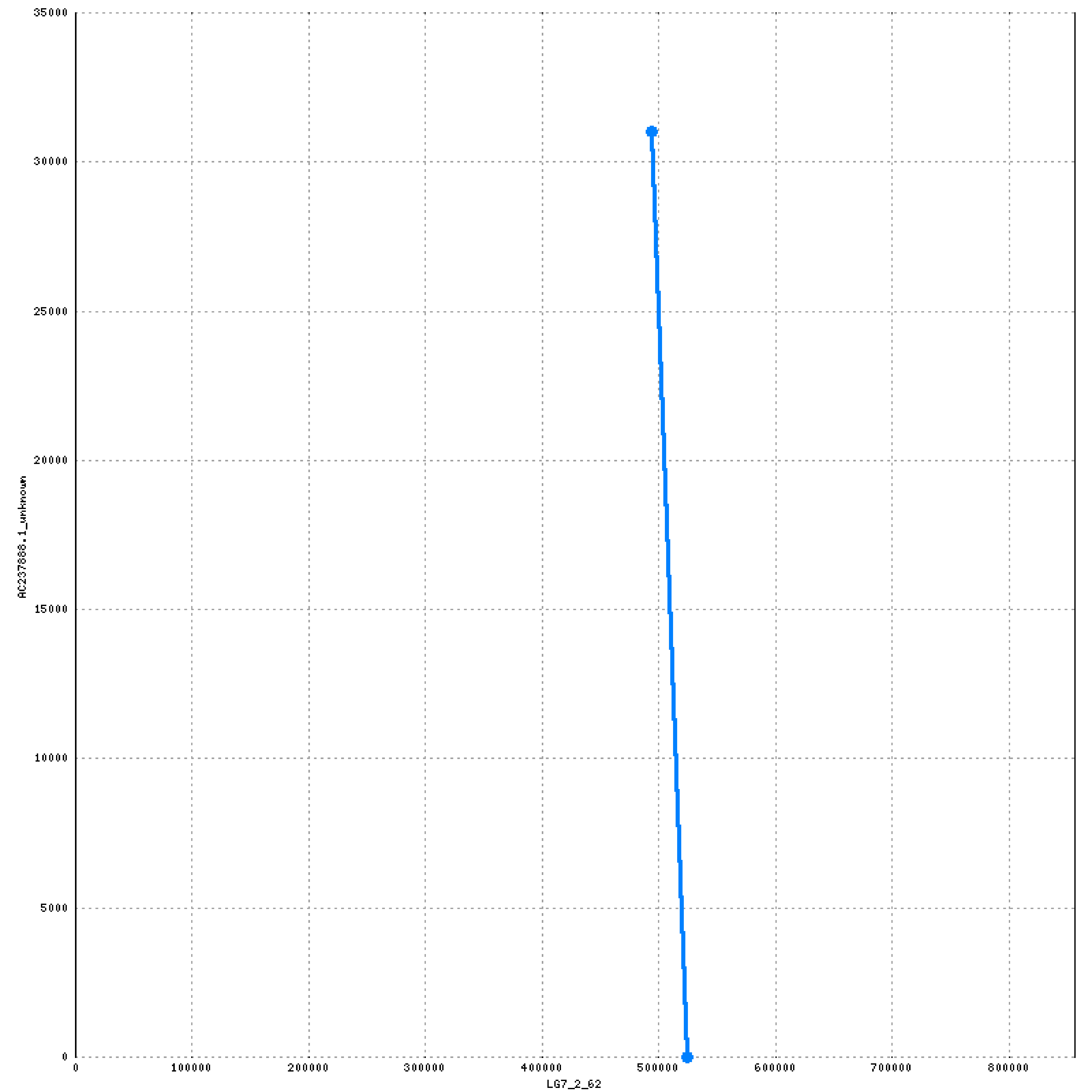

35000
30000
25000
20000
15000
10000
5000
0
AC237888.1_unknown
0
100000
200000
300000
400000
500000
600000
700000
800000
LG7_2_62

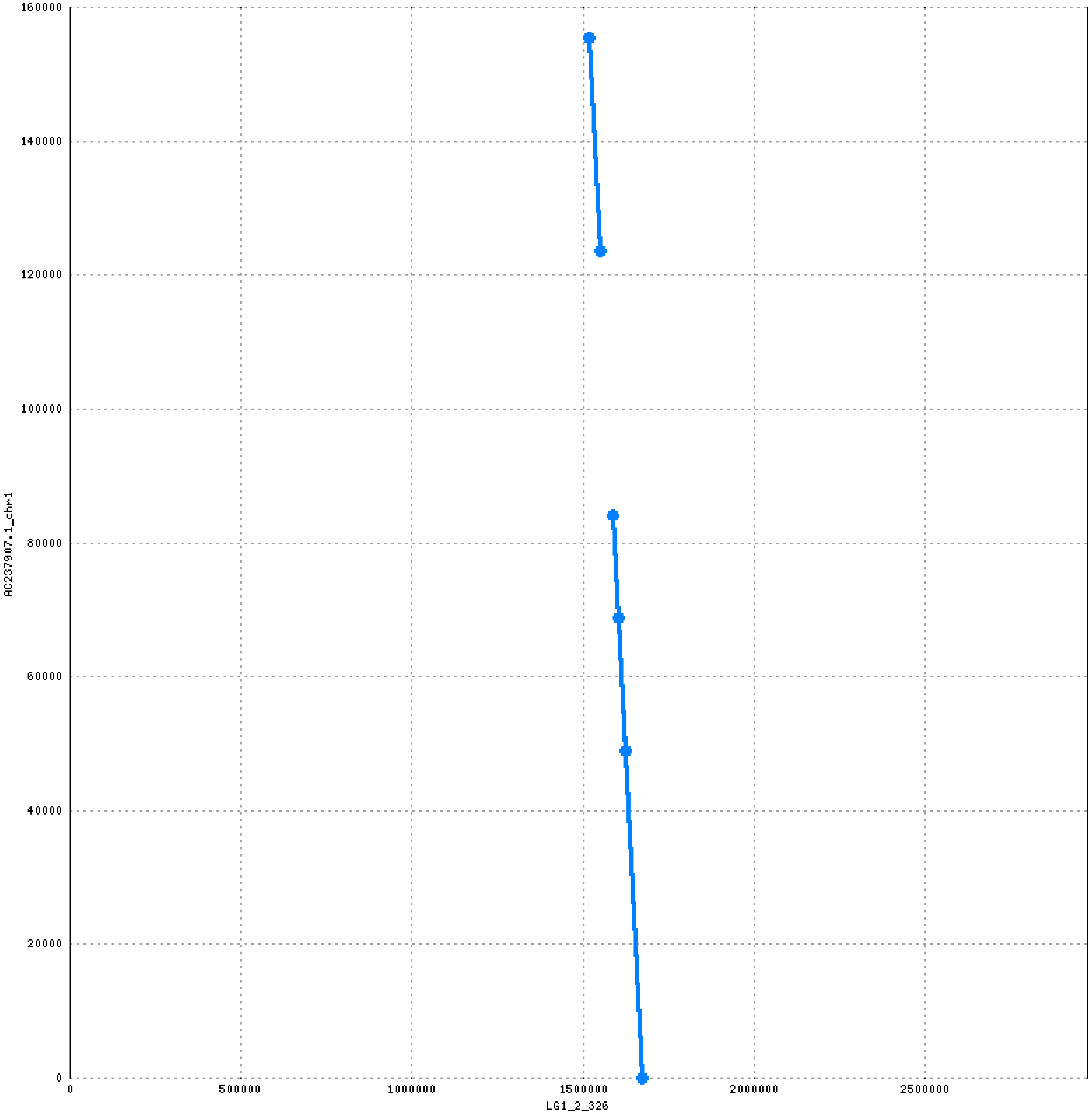

160000
140000
120000
100000
80000
60000
40000
20000
0
AC237907.1_chr1
0
500000
1000000
1500000
2000000
2500000
LG1_2_326

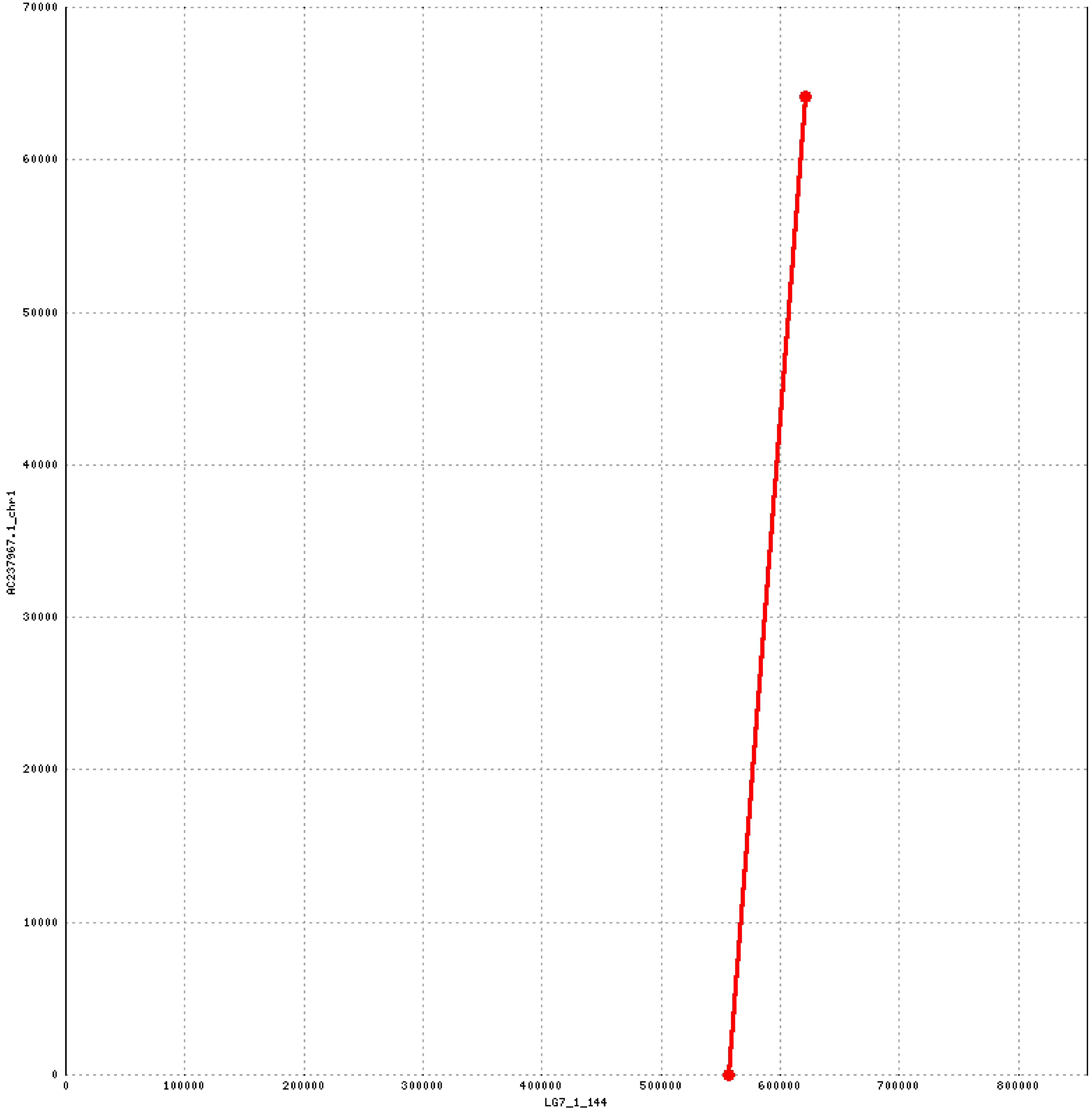

70000
60000
50000
40000
30000
20000
10000
0
AC237967.1_chr1
0
100000
200000
300000
400000
500000
600000
700000
800000
LG7_1_144

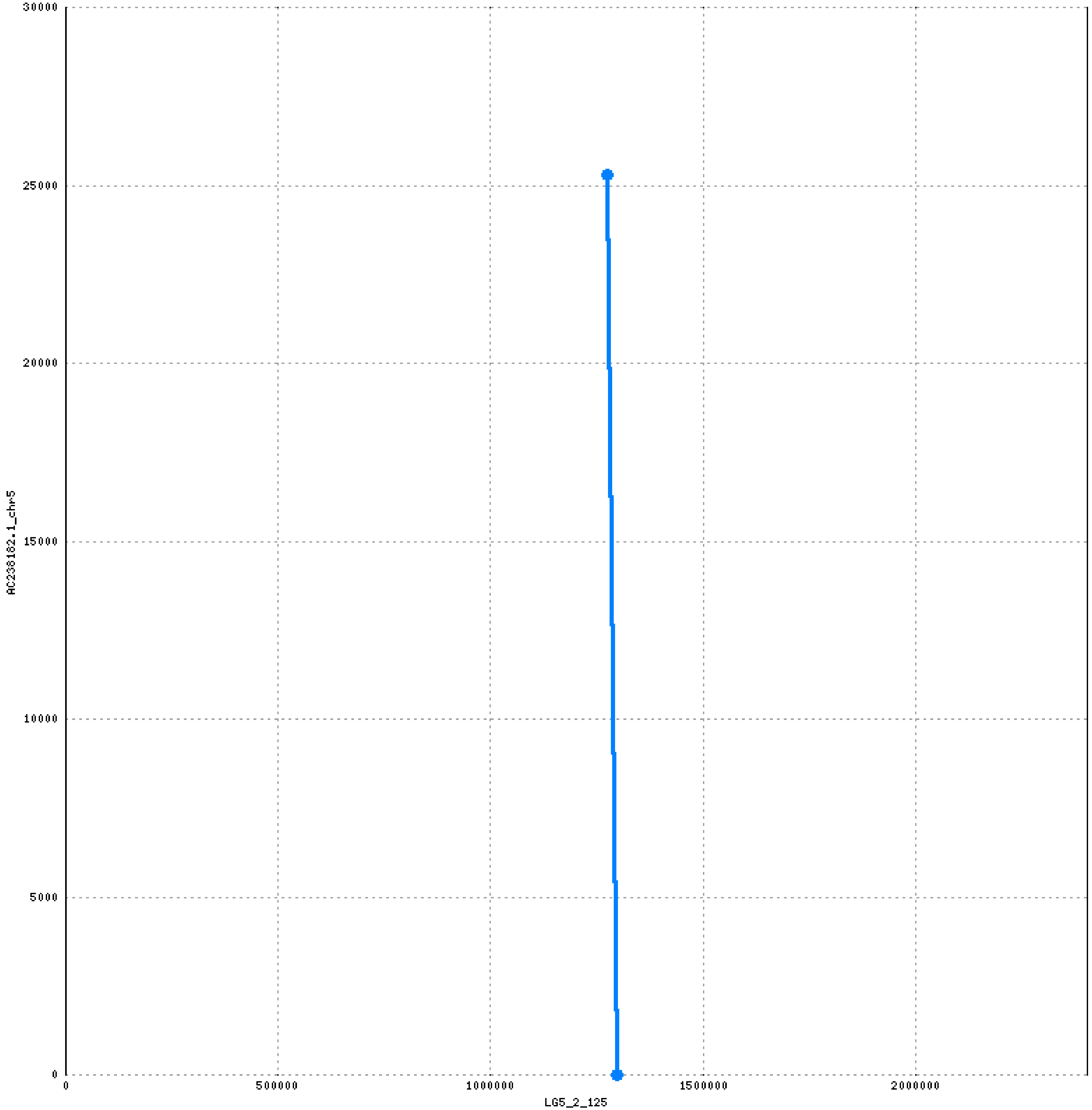

30000
25000
20000
15000
10000
5000
0
AC238182.1_chr5
0
500000
1000000
1500000
2000000
LG5_2_125

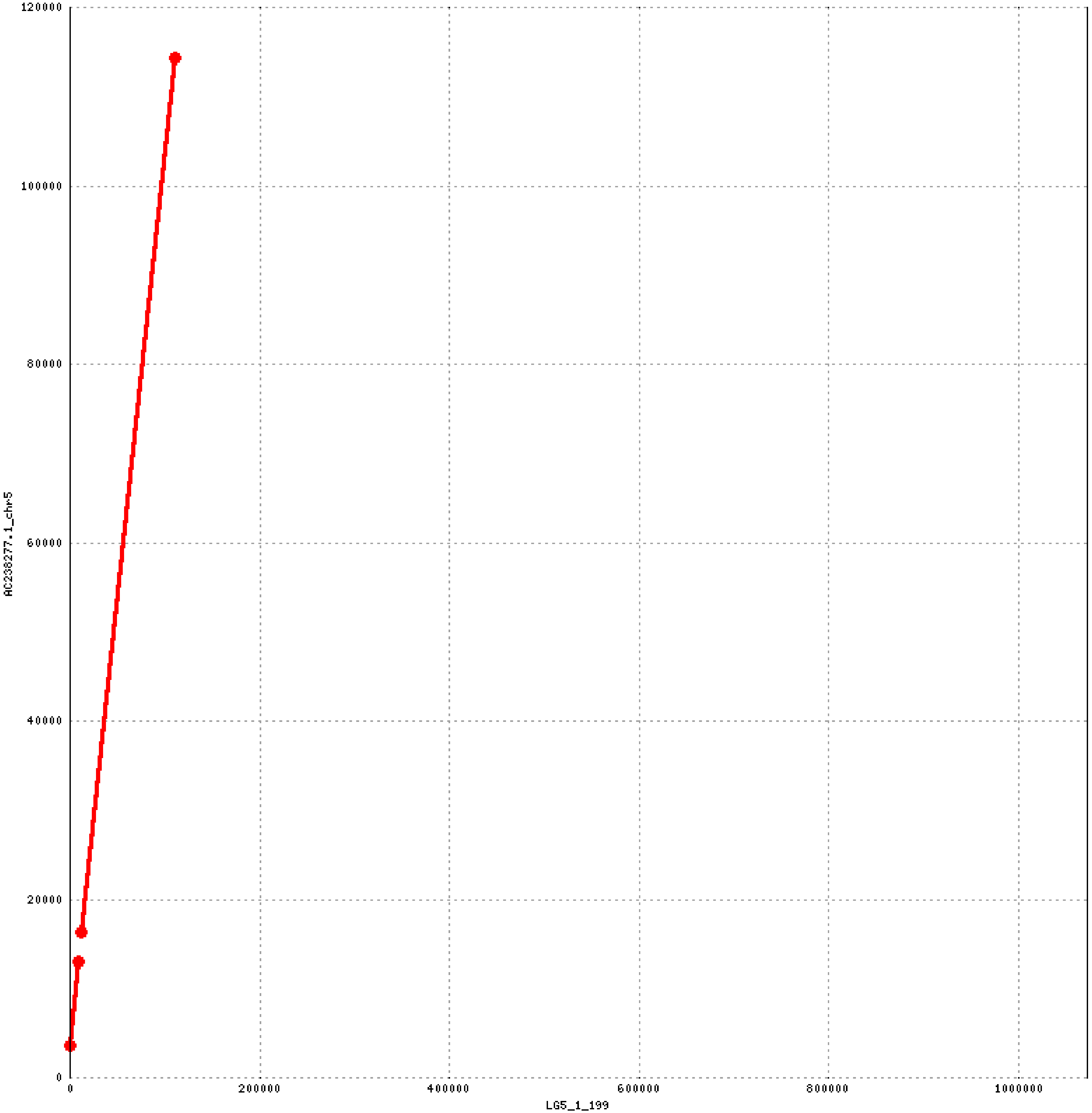
120000
100000
80000
60000
40000
20000
0
AC238277.1_chr5
0
200000
400000
600000
800000
1000000
LG5_1_199

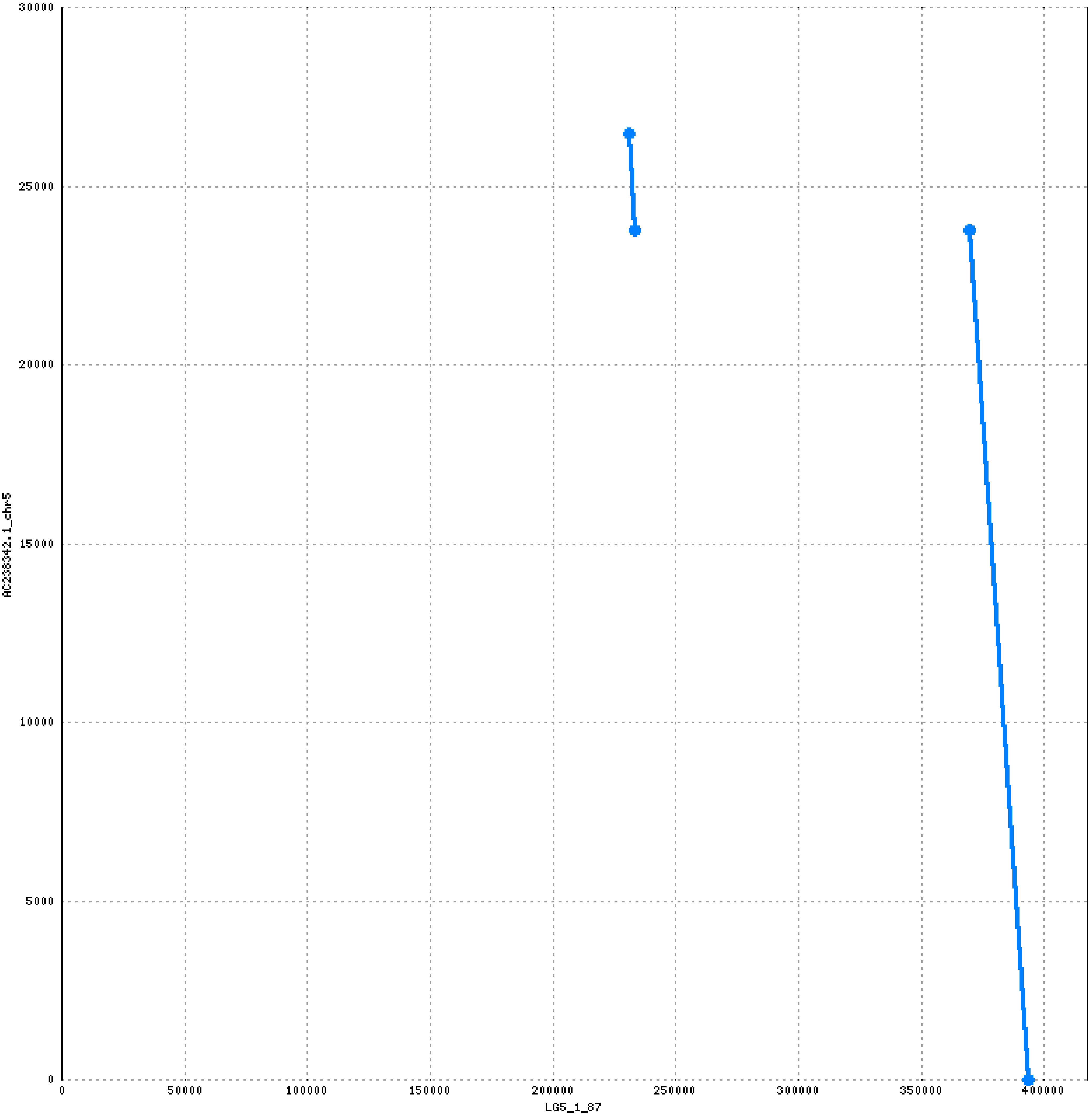

30000
25000
20000
15000
10000
5000
0
0
50000
100000
150000
200000
250000
300000
350000
400000
AC238342.1_chr5
LG5_1_87

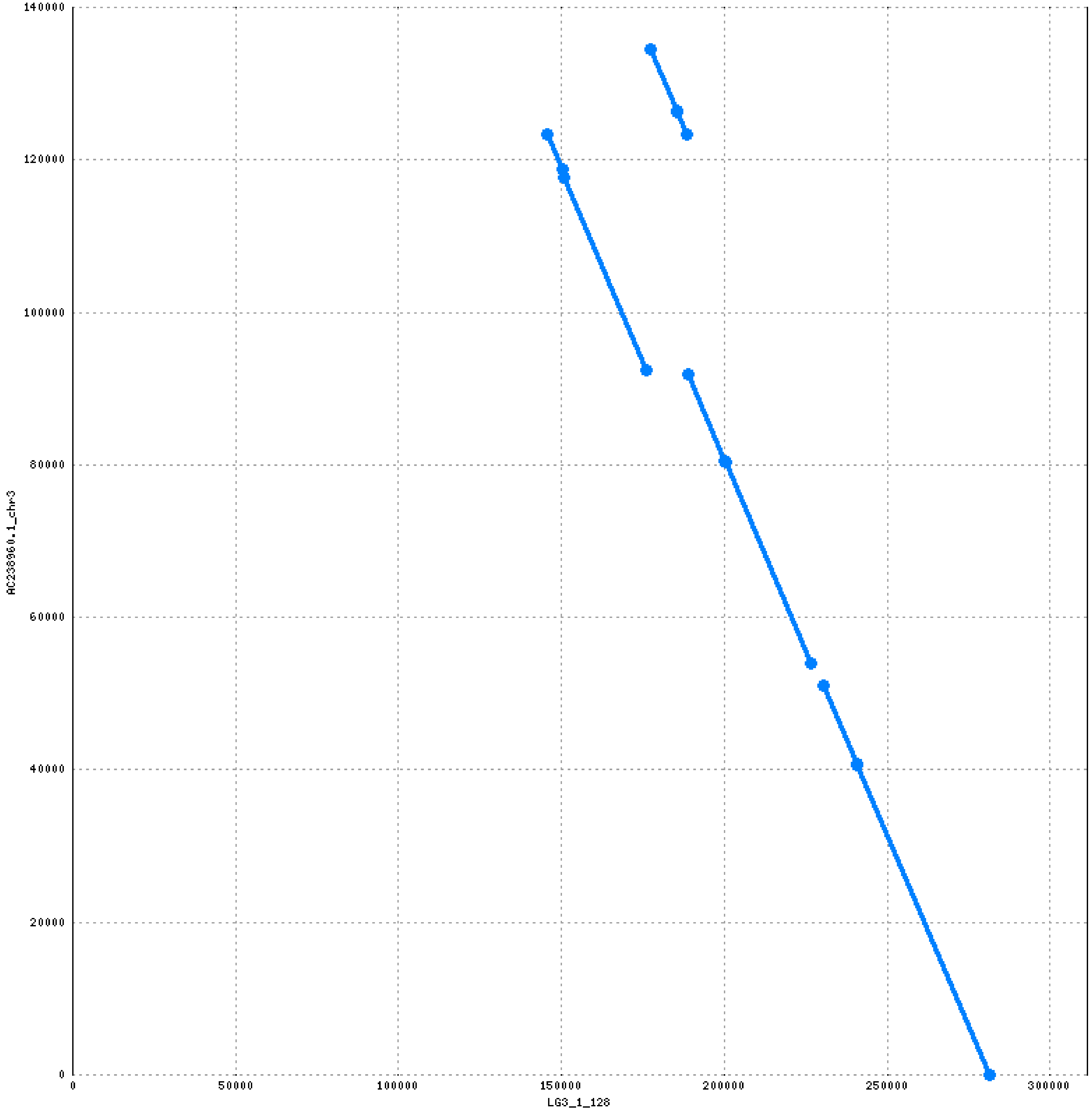

140000
120000
100000
80000
60000
40000
20000
0
AC238960.1_chr3
0
50000
100000
150000
200000
250000
300000
LG3_1_128

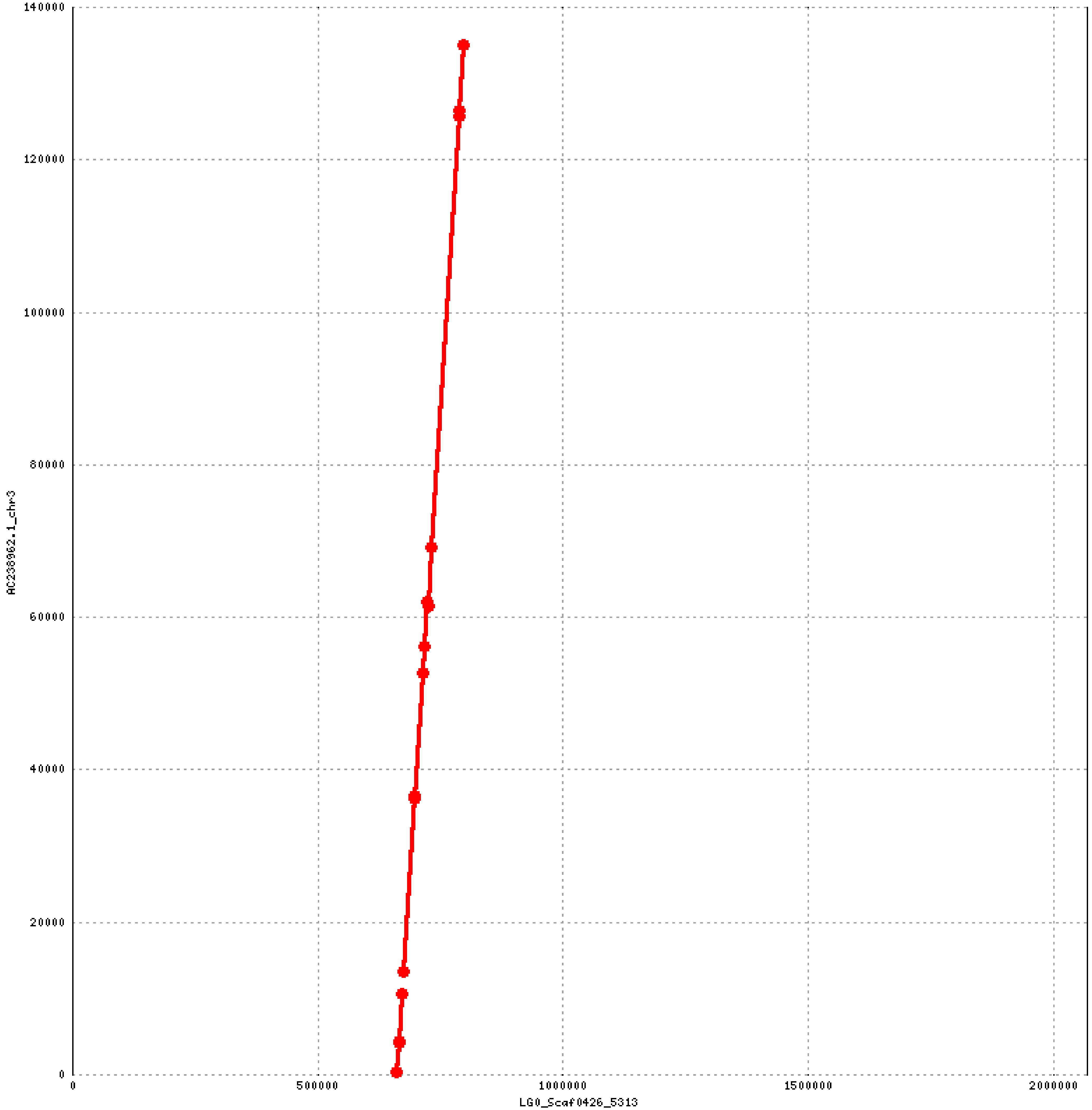

140000
120000
100000
80000
60000
40000
20000
0
AC238962.1_chr3
0
500000
1000000
1500000
2000000
LG0_Scaf0426_5313

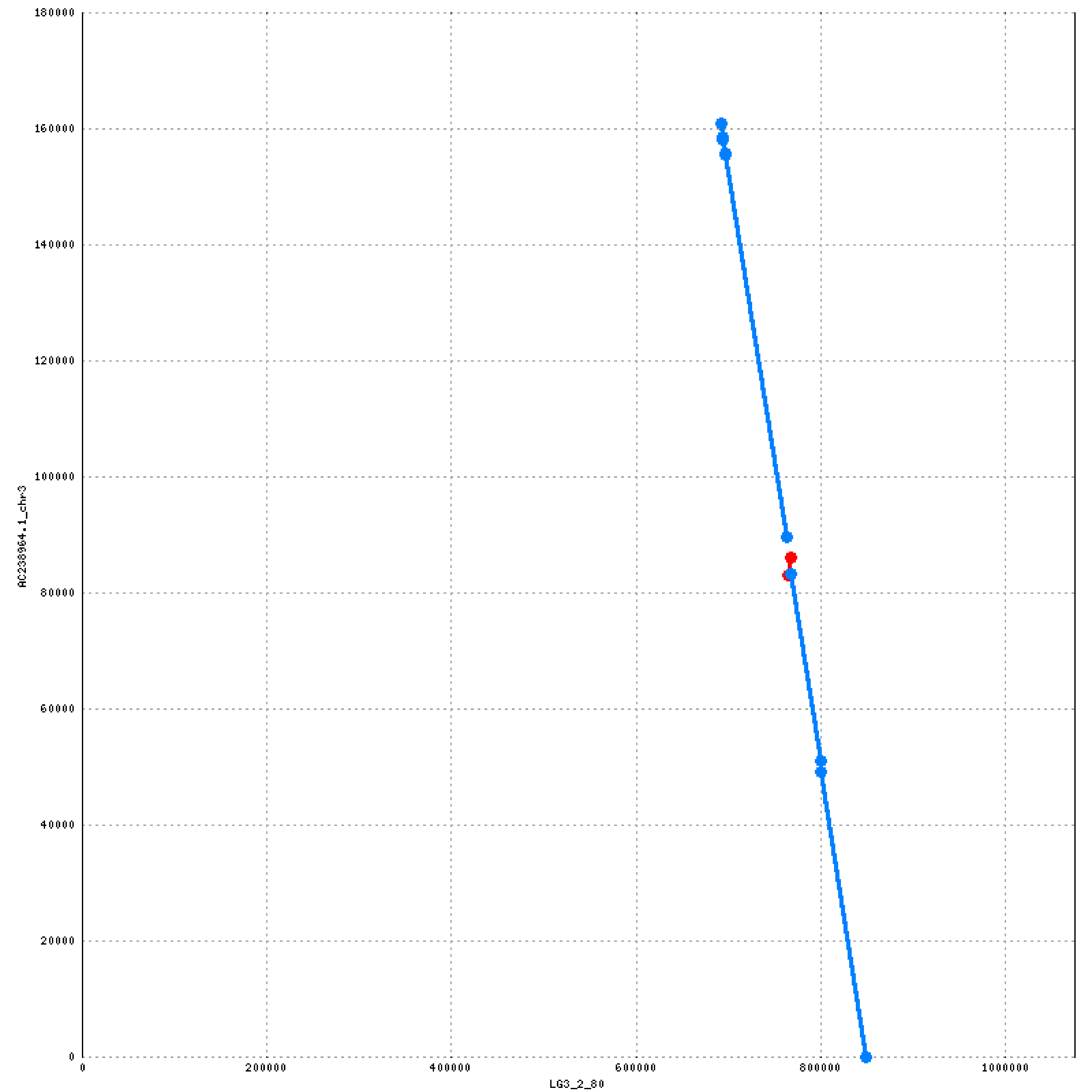
180000
160000
140000
120000
100000
80000
60000
40000
20000
0
AC238964.1_chr3
0
200000
400000
600000
800000
1000000
LG3_2_80

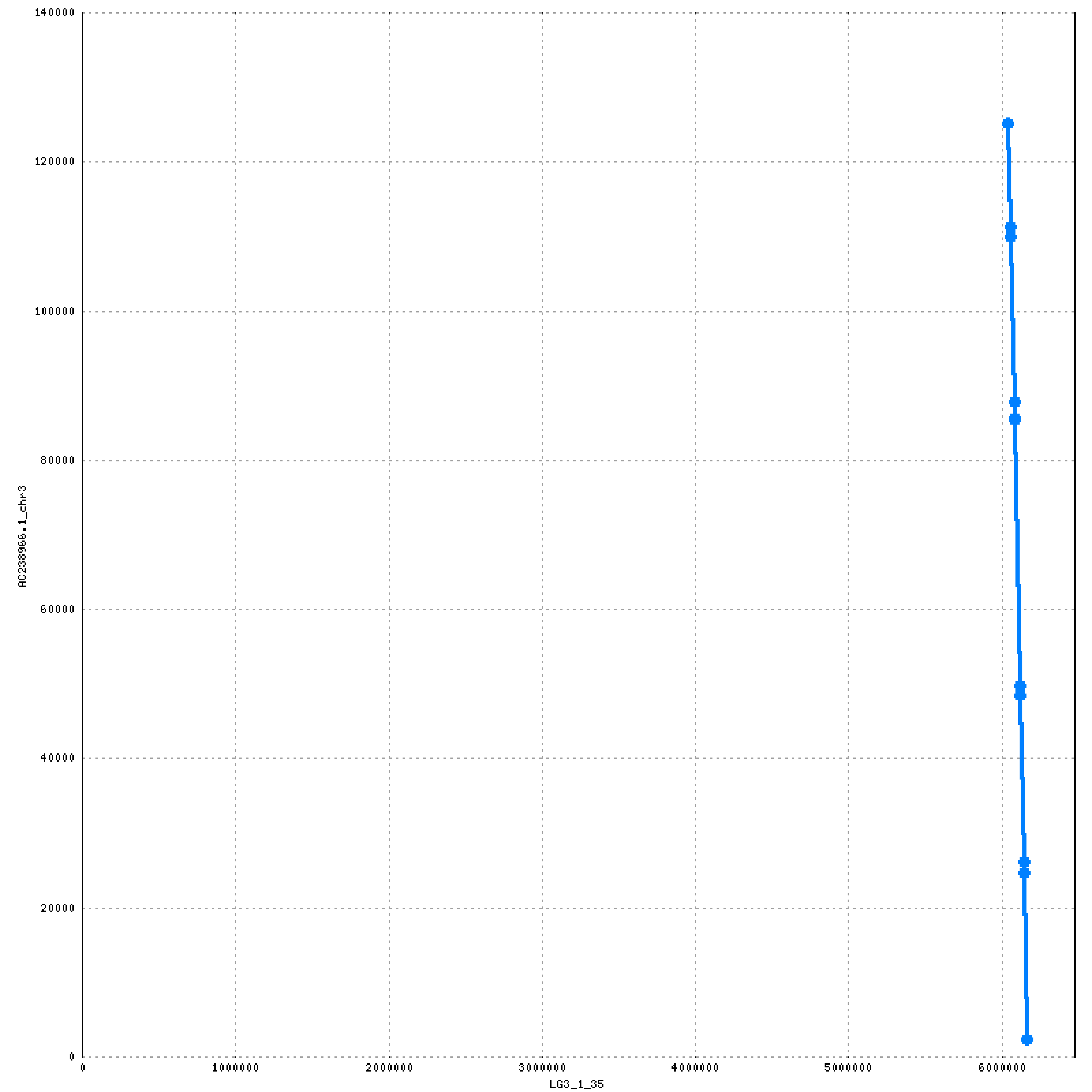

140000
120000
100000
80000
60000
40000
20000
0
AC238966.1_chr3
0
1000000
2000000
3000000
4000000
5000000
6000000
LG3_1_35

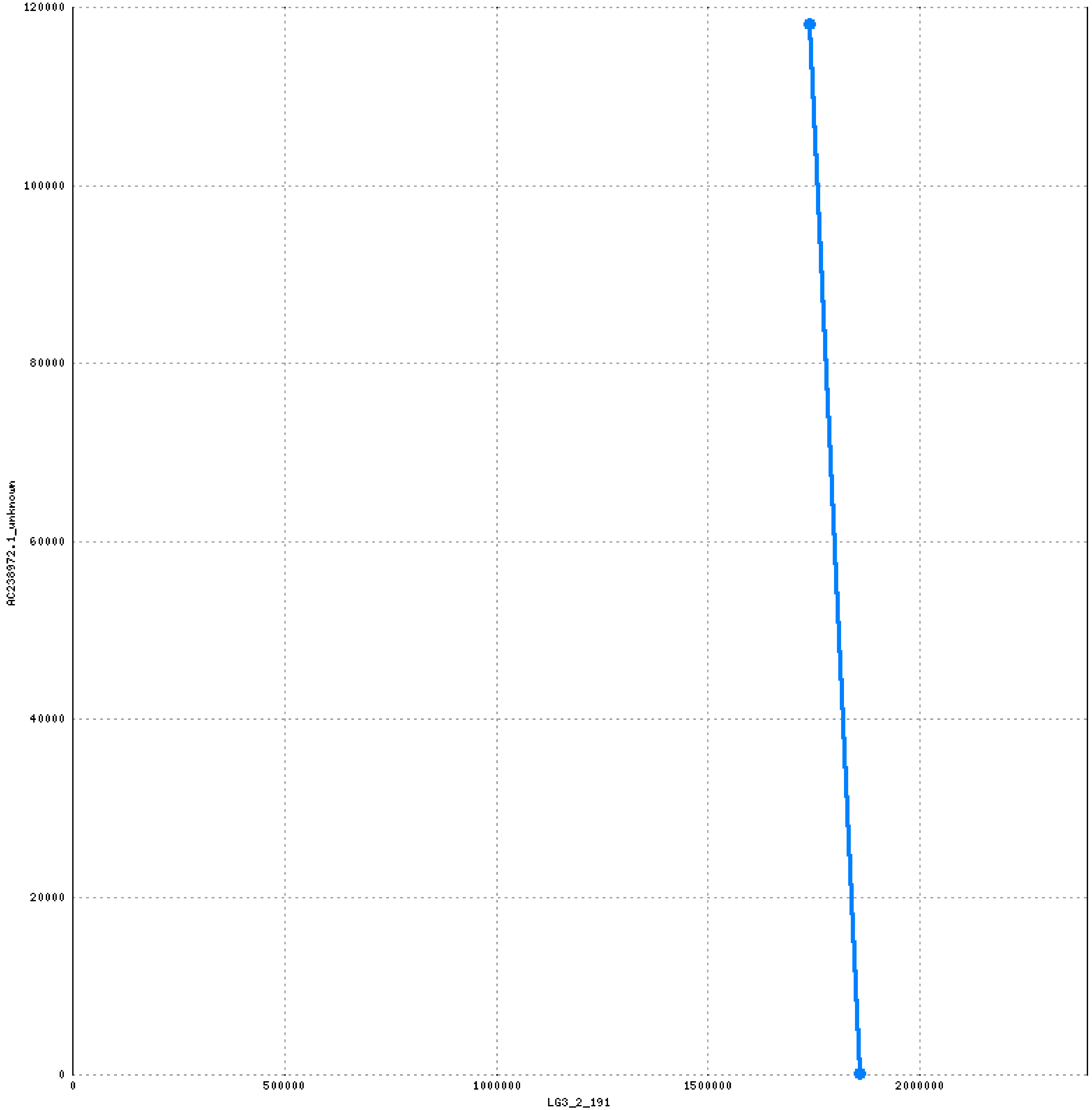

120000
100000
80000
60000
40000
20000
0
AC238972.1_unknown
0
500000
1000000
1500000
2000000
LG3_2_191

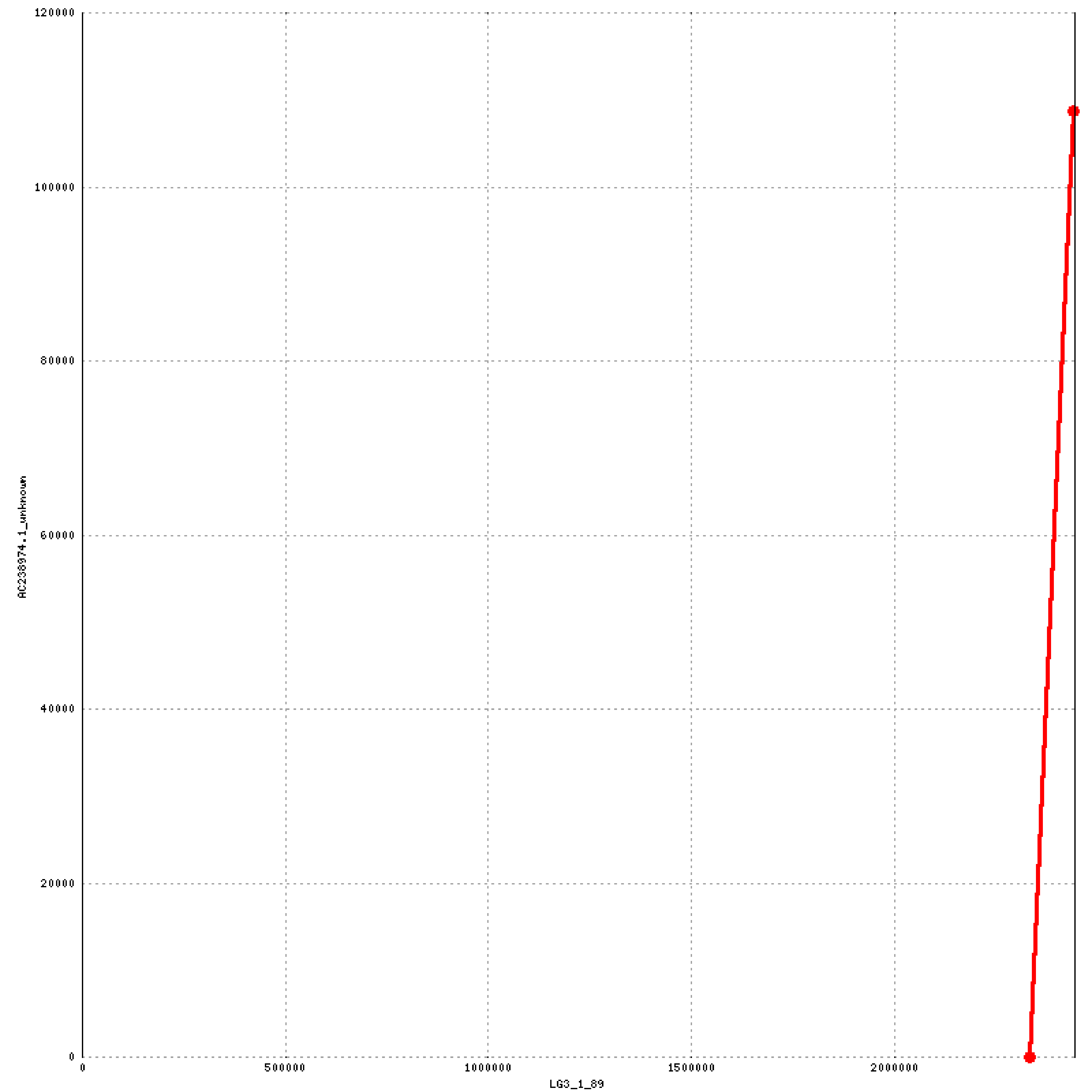
120000
100000
80000
60000
40000
20000
0
AC238974.1_unknown
0
500000
1000000
1500000
2000000
LG3_1_89

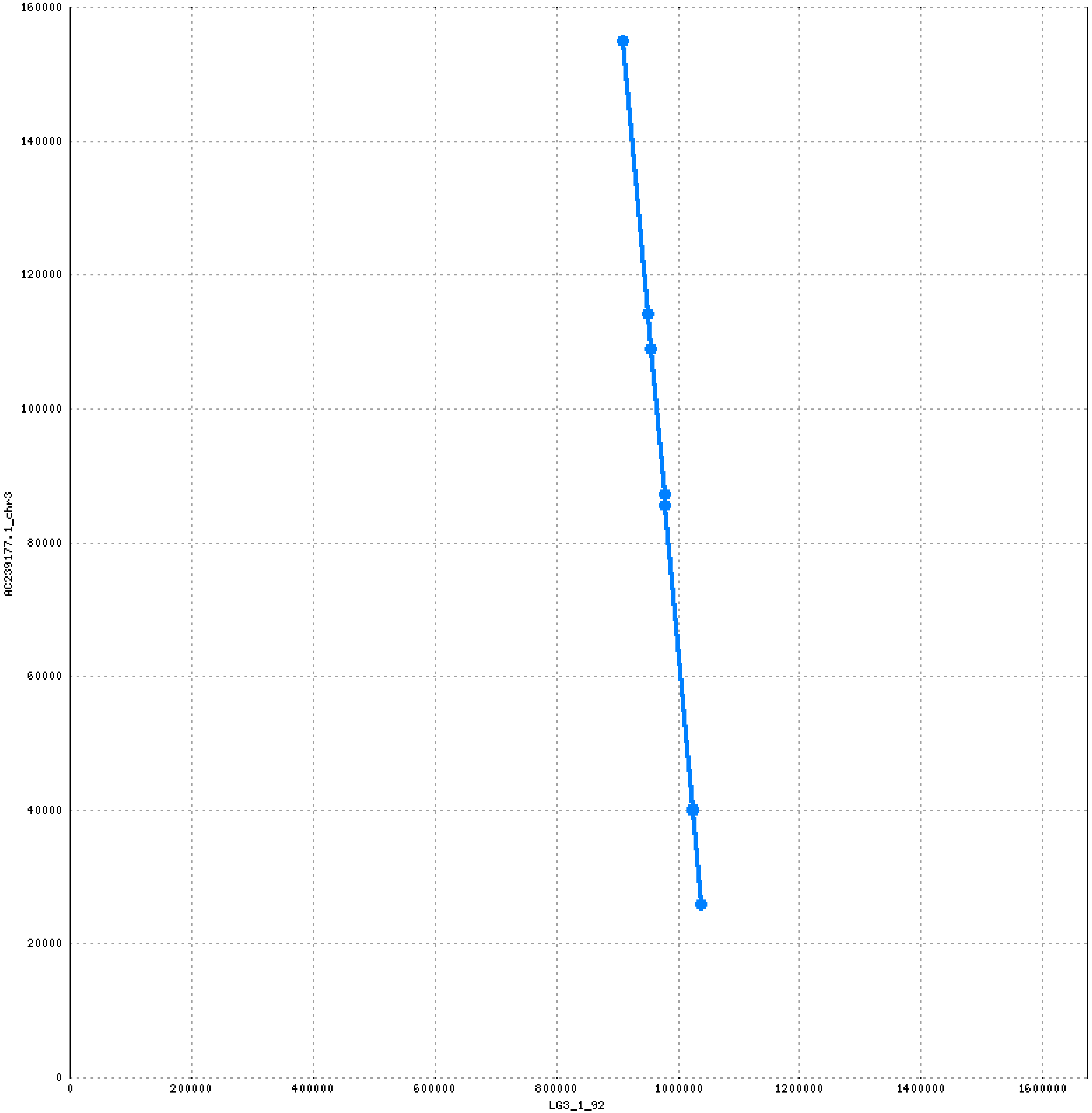

160000
140000
120000
100000
80000
60000
40000
20000
0
AC239177.1_chr3
0
200000
400000
600000
800000
1000000
1200000
1400000
1600000
LG3_1_92

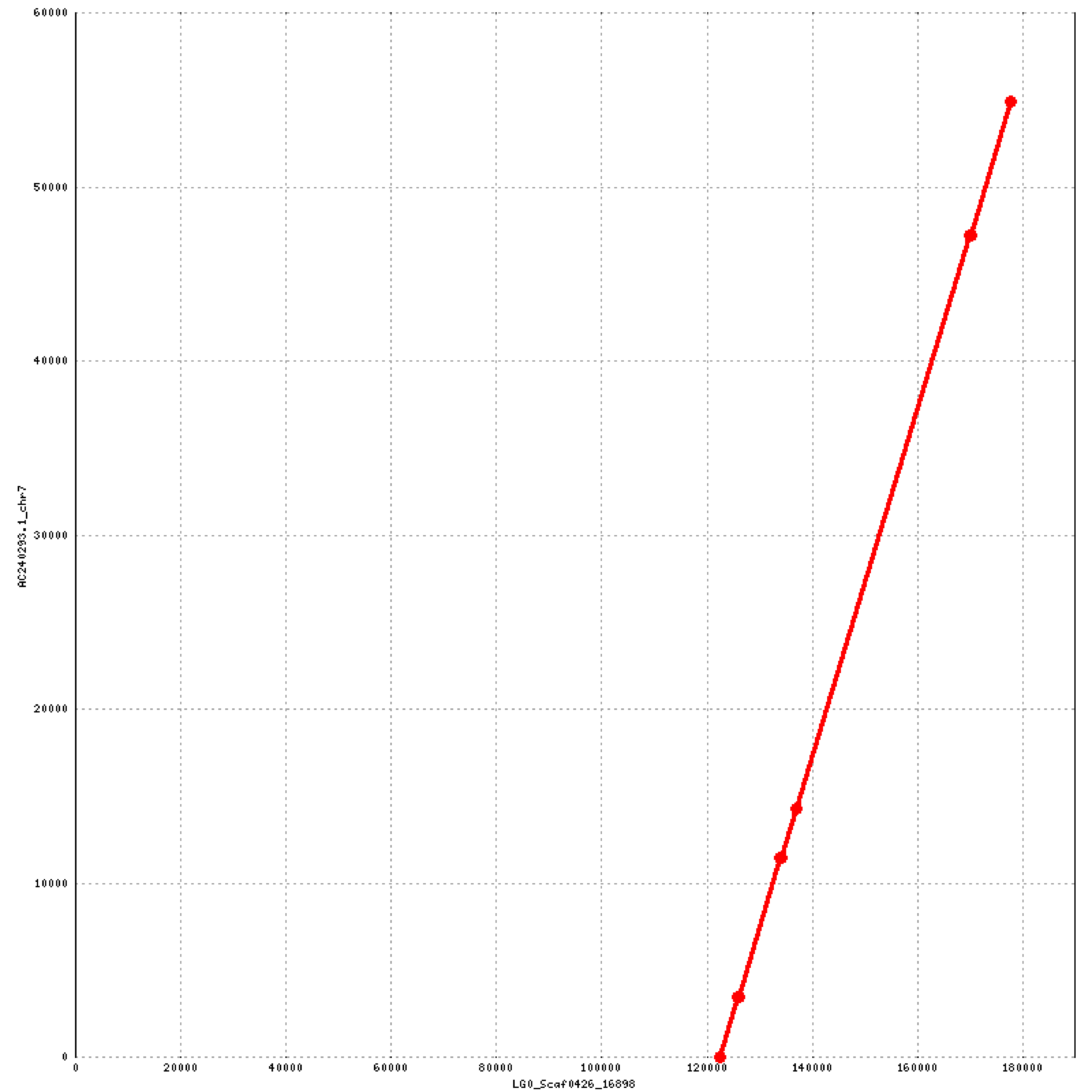

60000
50000
40000
30000
20000
10000
0
AC240293.1_chr7
0
20000
40000
60000
80000
100000
120000
140000
160000
180000
LG0_Scaf0426_16898

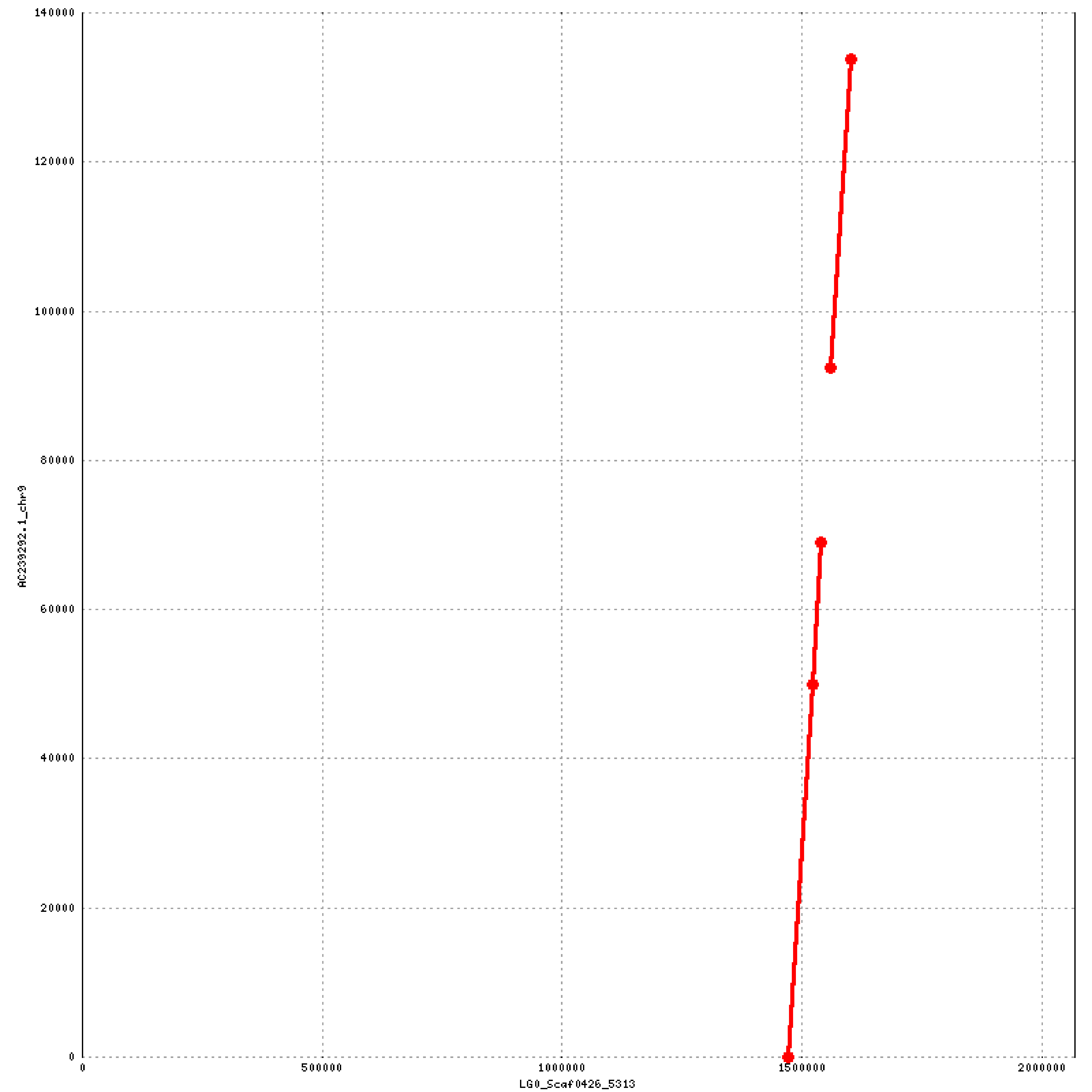
140000
120000
100000
80000
60000
40000
20000
0
AC239292.1_chr9
0
500000
1000000
1500000
2000000
LG0_Scaf0426_5313

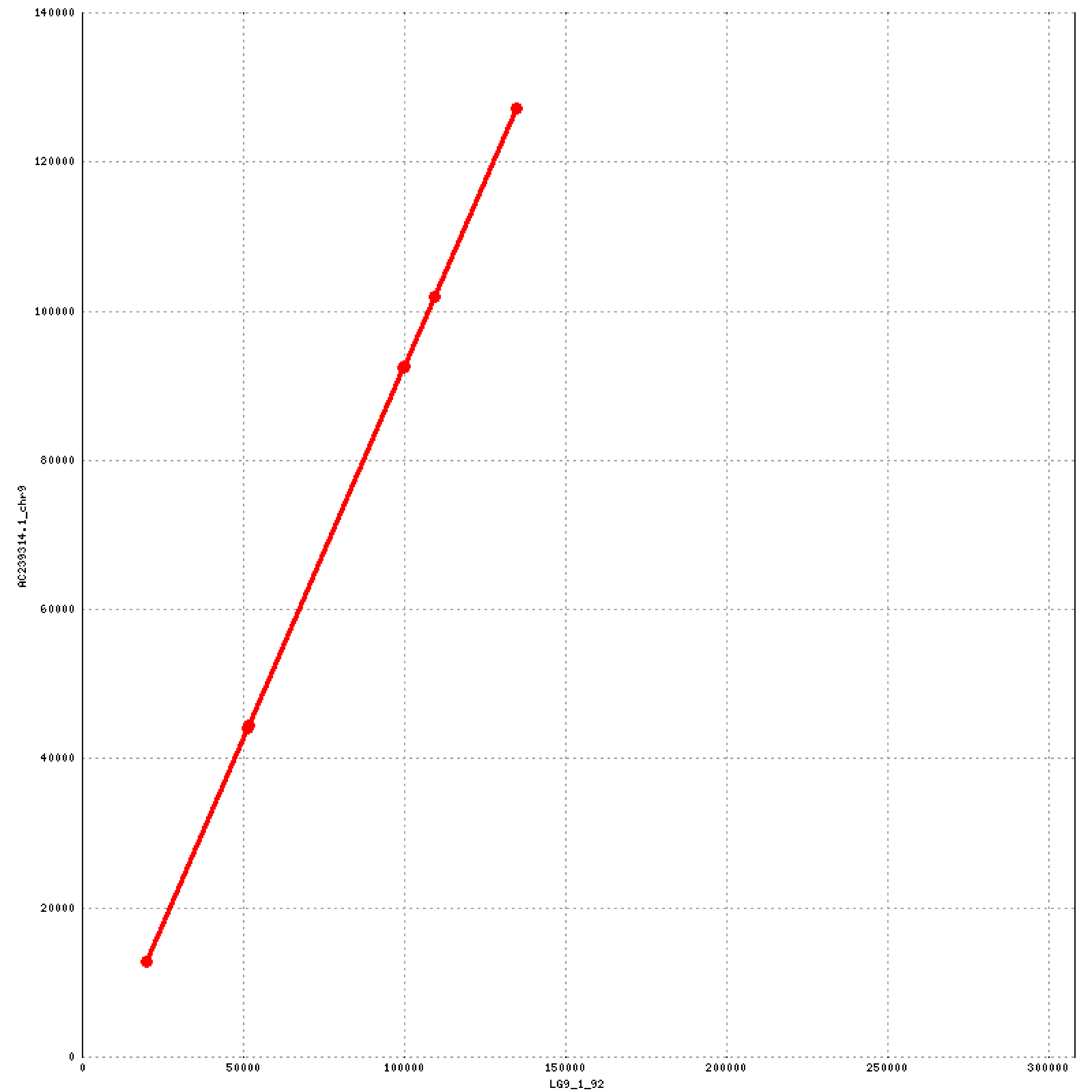

140000
120000
100000
80000
60000
40000
20000
0
AC239314.1_chr9
0
50000
100000
150000
200000
250000
300000
LG9_1_92

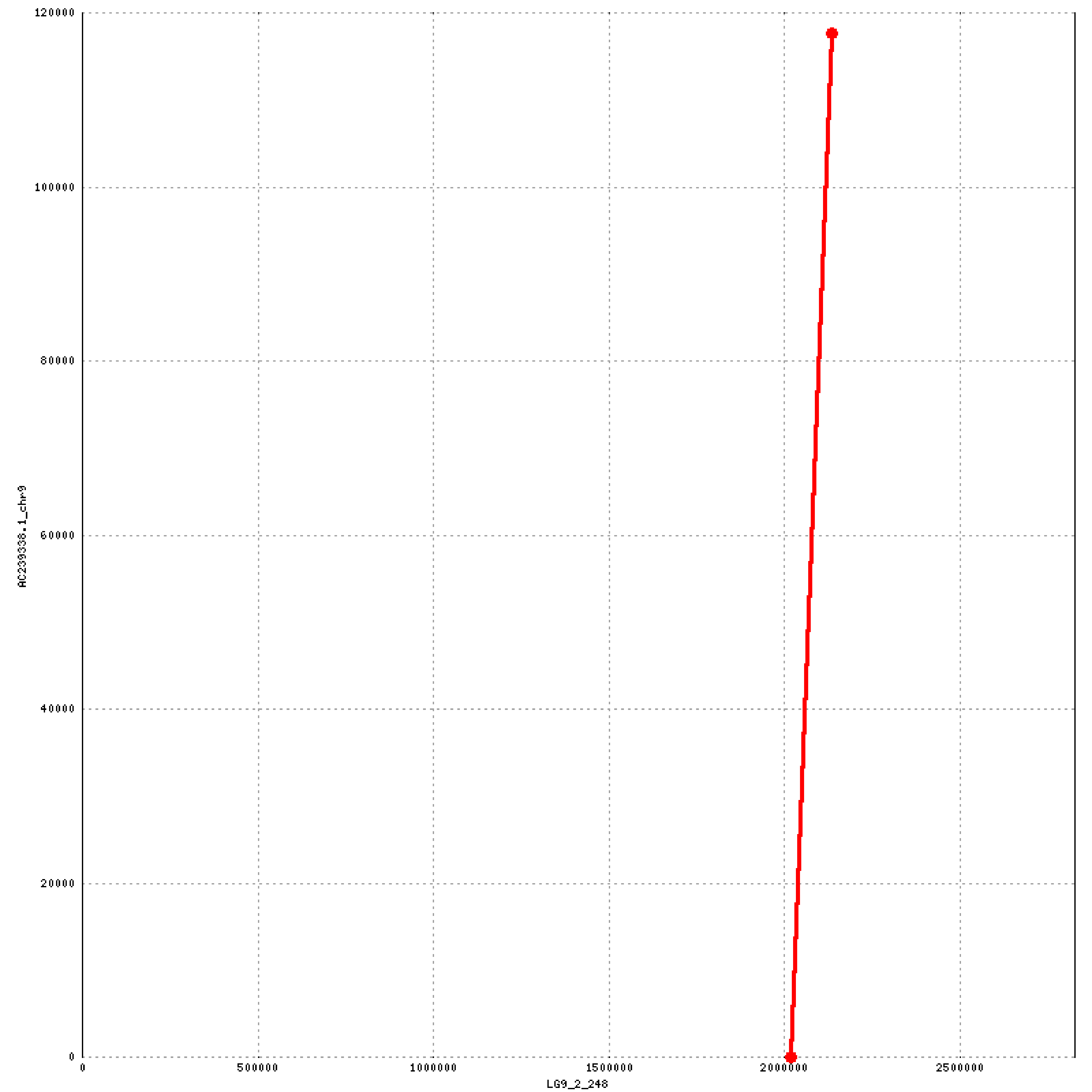

120000
100000
80000
60000
40000
20000
0
AC239338.1_chr9
0
500000
1000000
1500000
2000000
2500000
LG9_2_248

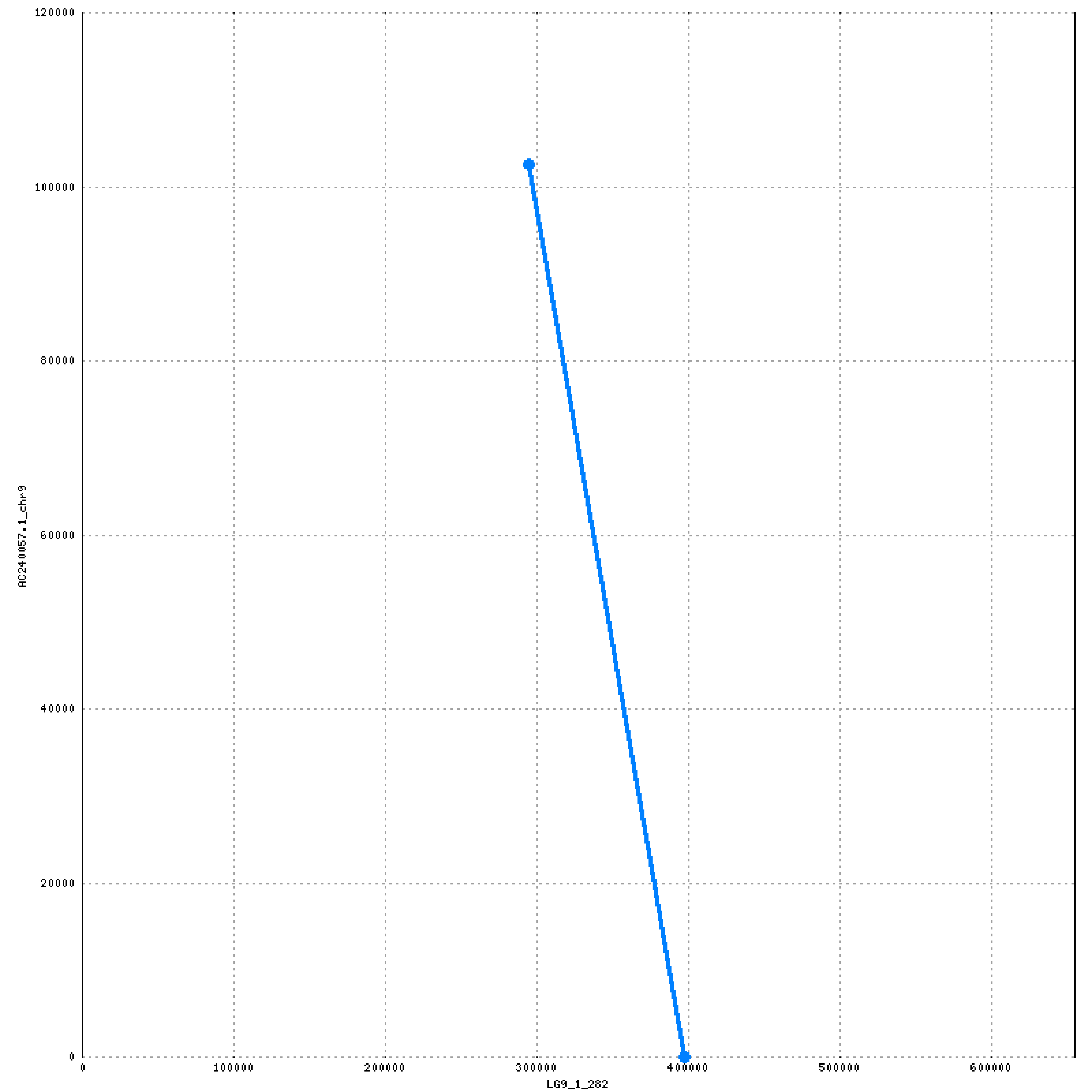

120000
100000
80000
60000
40000
20000
0
AC240057.1_chr9
0
100000
200000
300000
400000
500000
600000
LG9_1_282

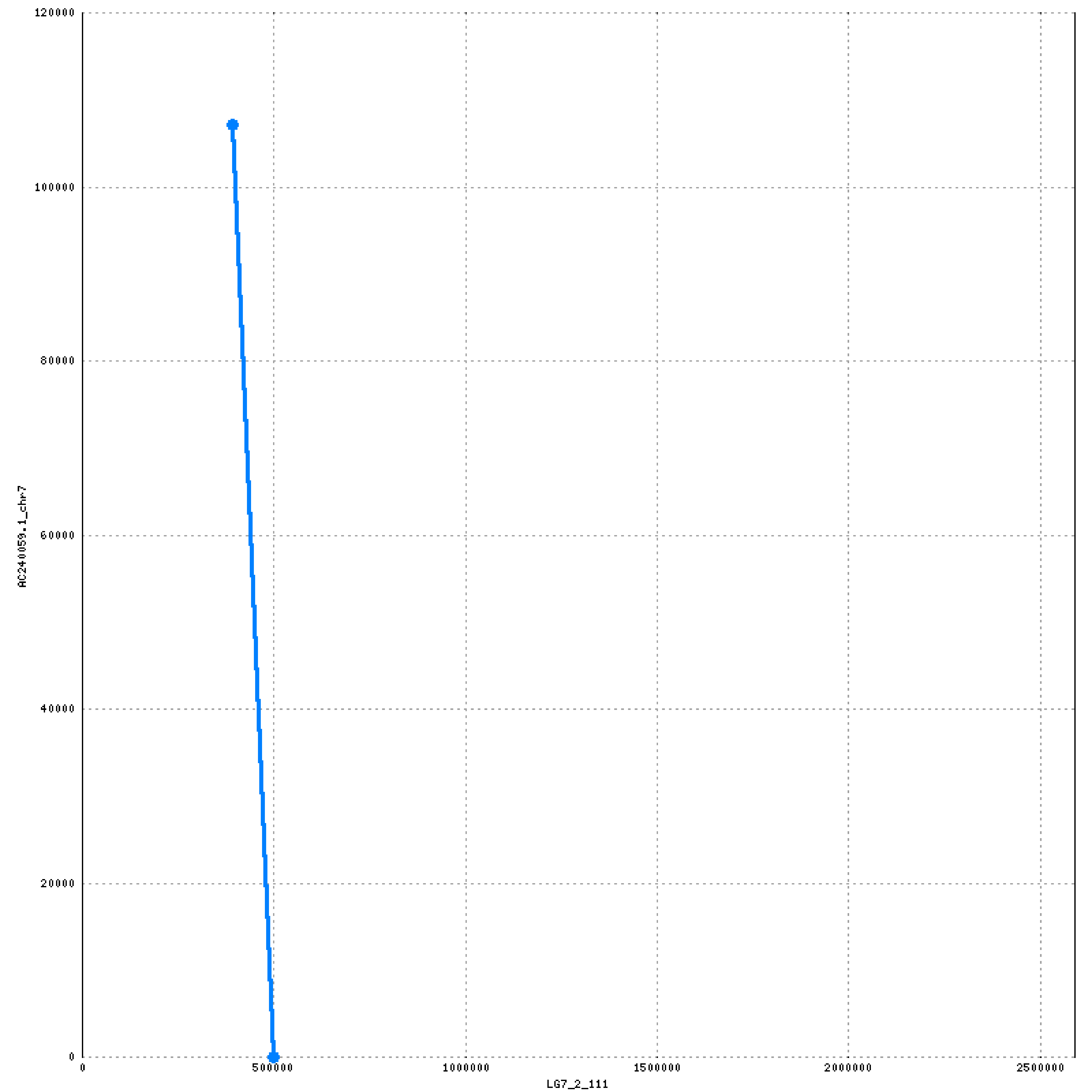

120000
100000
80000
60000
40000
20000
0
AC240059.1_chr7
0
500000
1000000
1500000
2000000
2500000
LG7_2_111

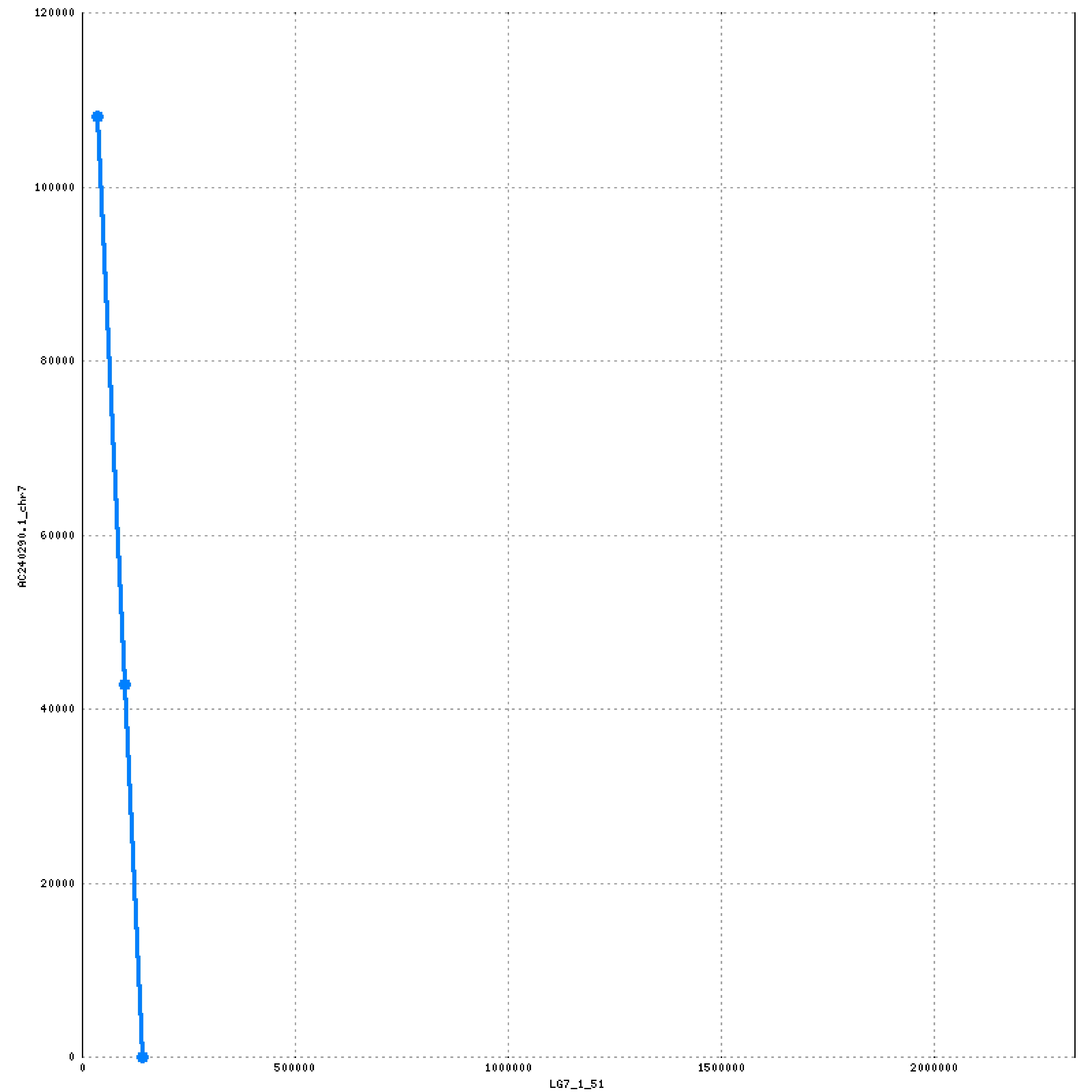

120000
100000
80000
60000
40000
20000
0
AC240290.1_chr7
0
500000
1000000
1500000
2000000
LG7_1_51

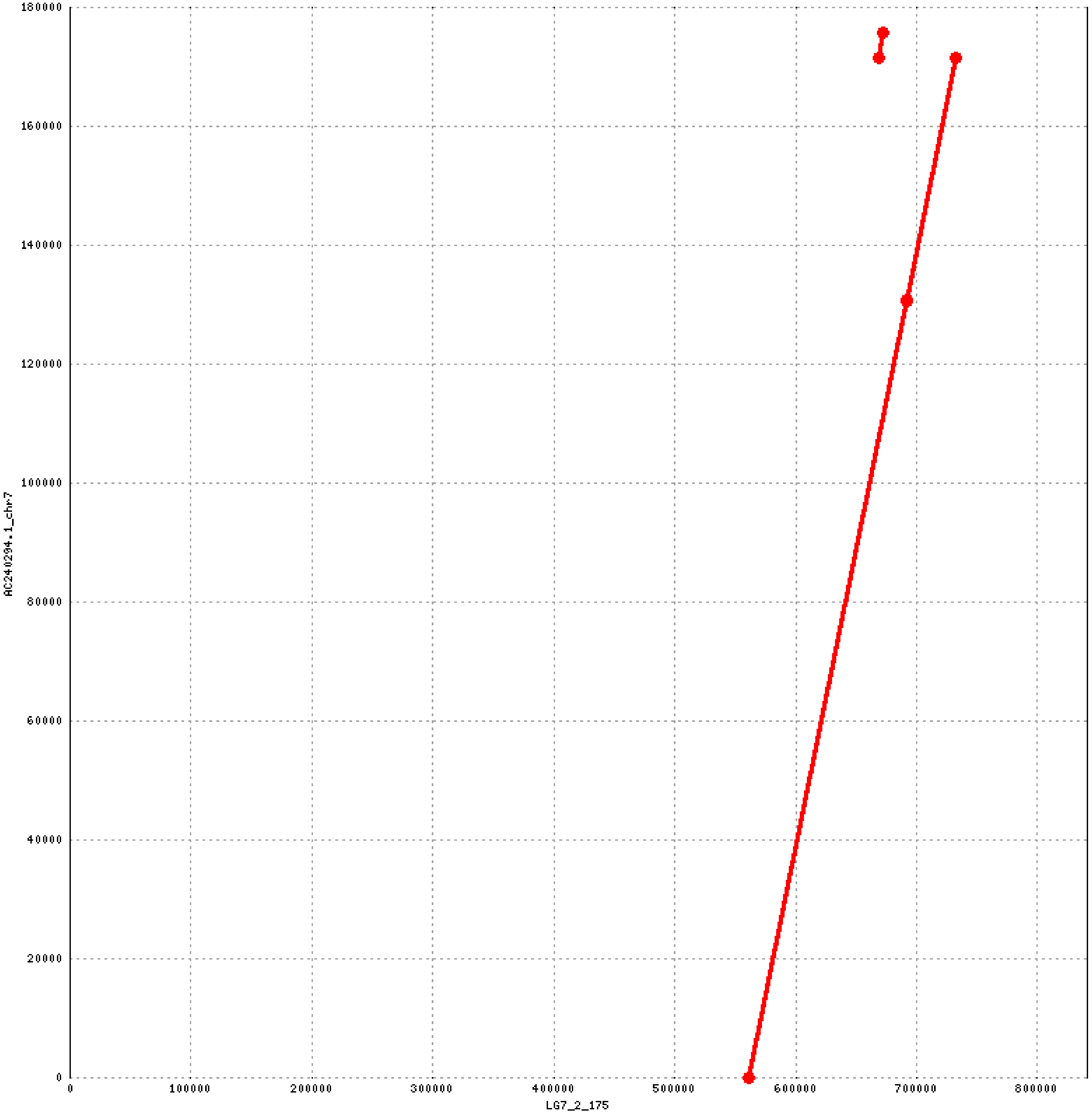

180000
160000
140000
120000
100000
80000
60000
40000
20000
0
0
100000
200000
300000
400000
500000
600000
700000
800000
AC240294.1_chr7
LG7_2_175

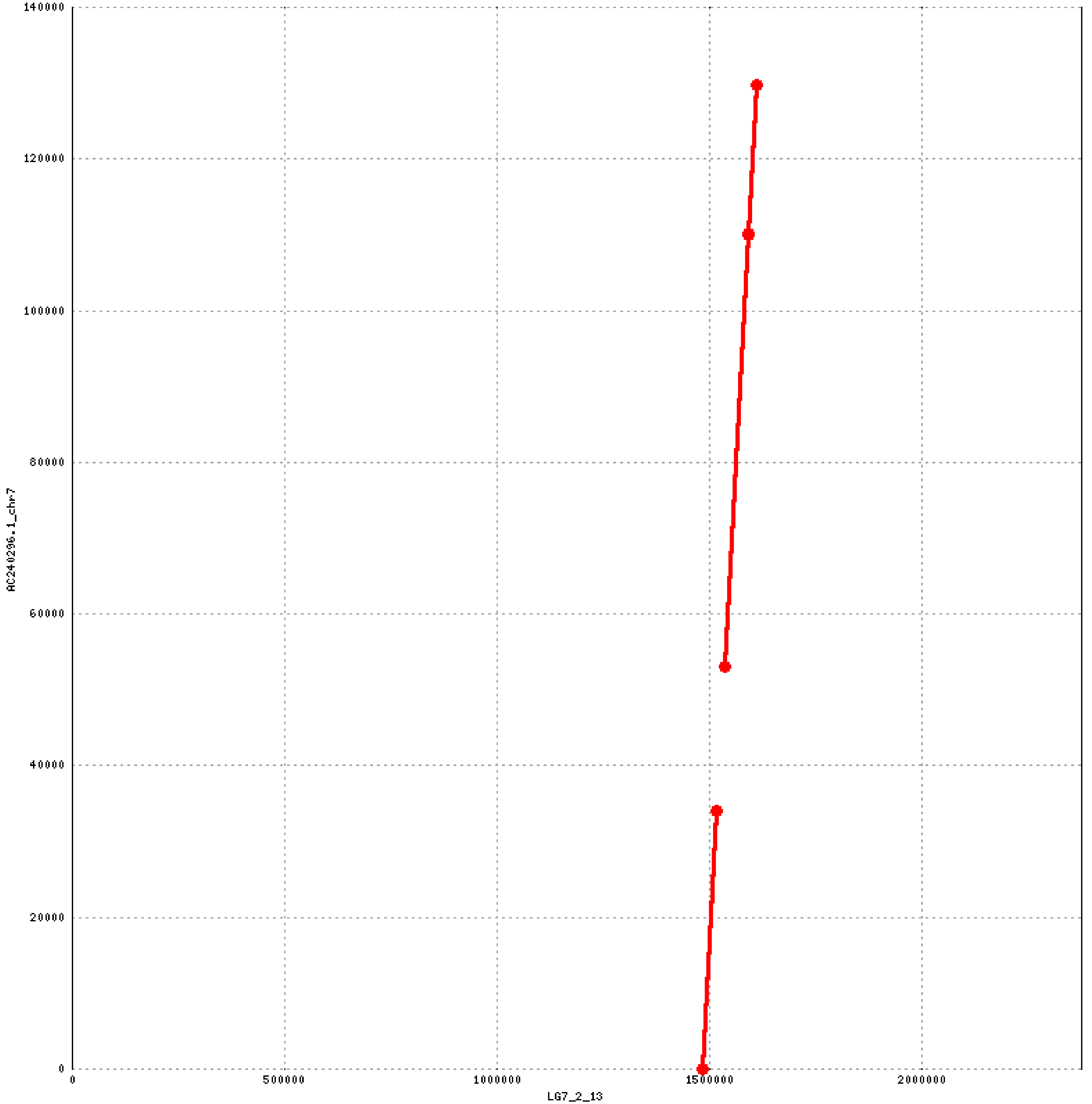

140000
120000
100000
80000
60000
40000
20000
0
AC240296.1_chr7
0
500000
1000000
1500000
2000000
LG7_2_13

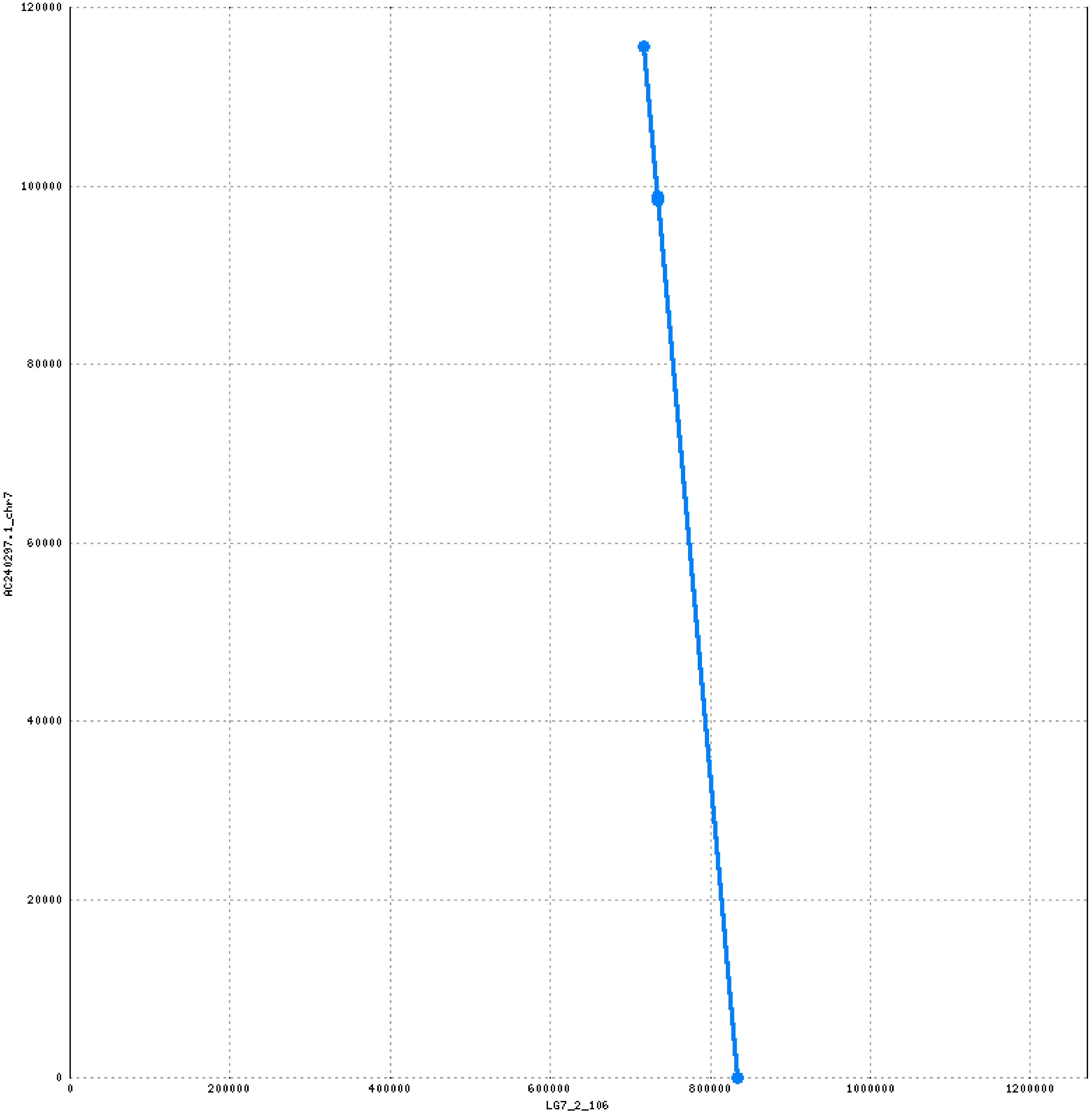

120000
100000
80000
60000
40000
20000
0
AC240297.1_chr7
0
200000
400000
600000
800000
1000000
1200000
LG7_2_106

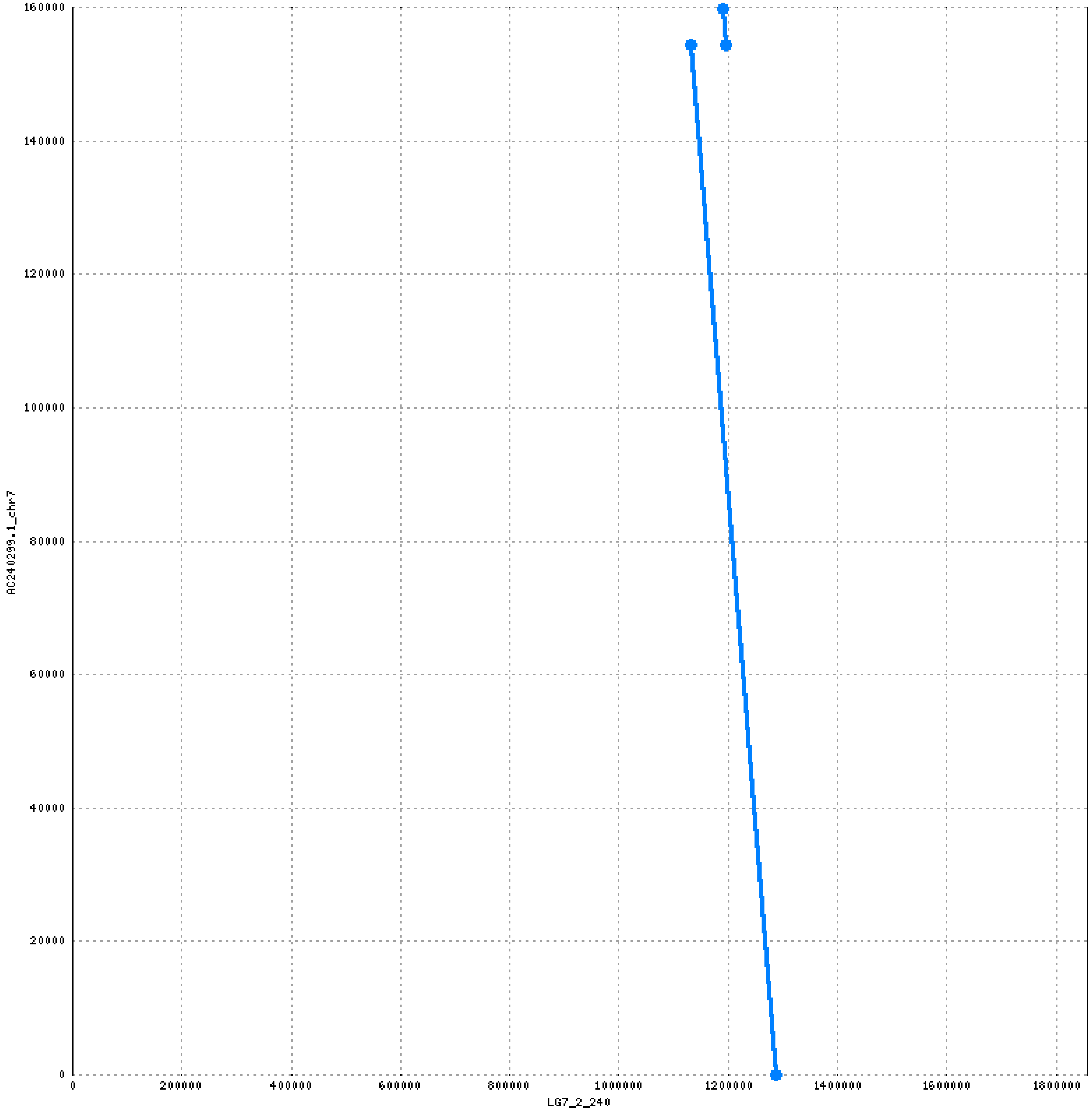

160000
140000
120000
100000
80000
60000
40000
20000
0
AC240299.1_chr7
0
200000
400000
600000
800000
1000000
1200000
1400000
1600000
1800000
LG7_2_240

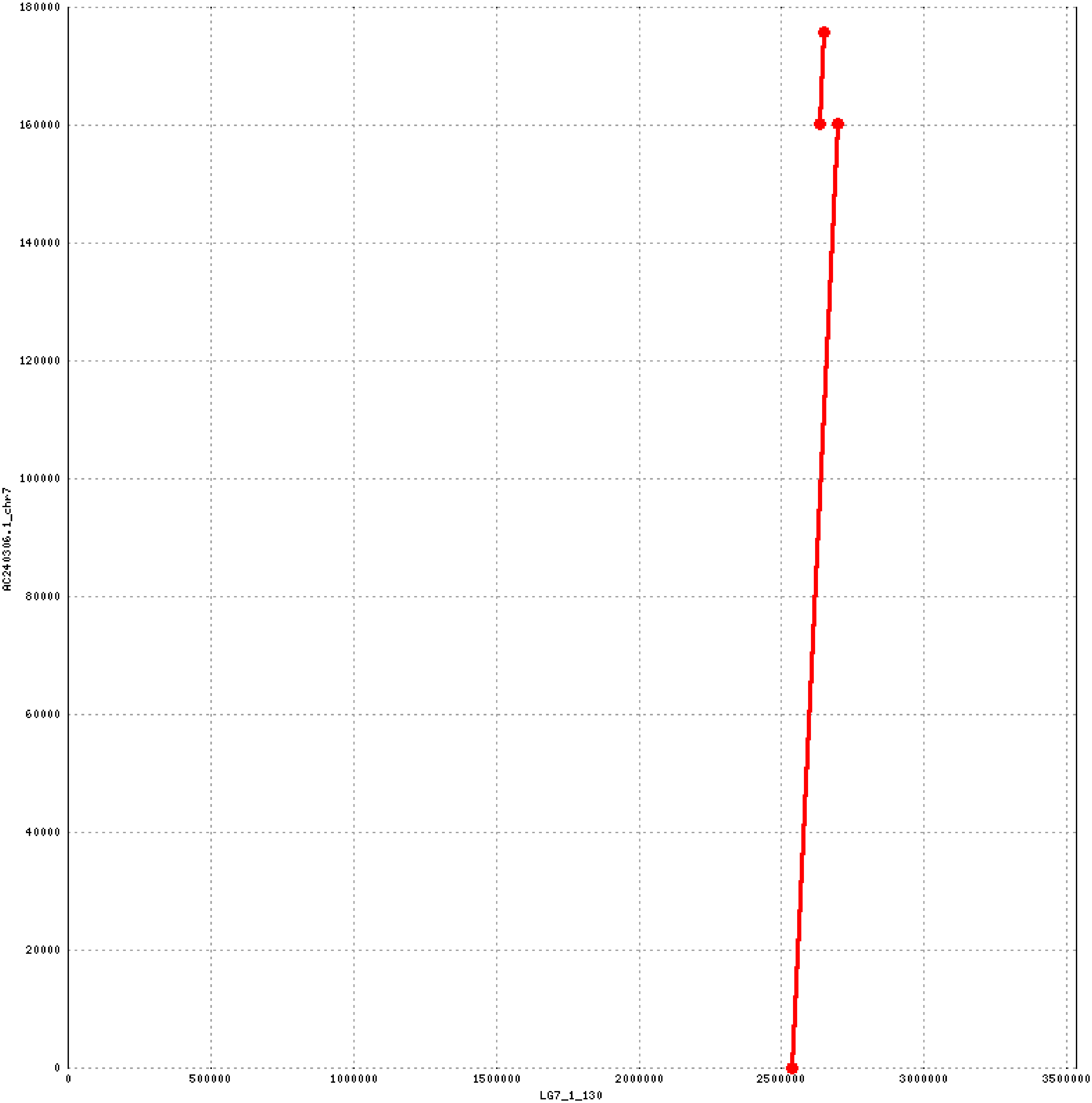
180000
160000
140000
120000
100000
80000
60000
40000
20000
0
AC240306.1_chr7
0
500000
1000000
1500000
2000000
2500000
3000000
3500000
LG7_1_130

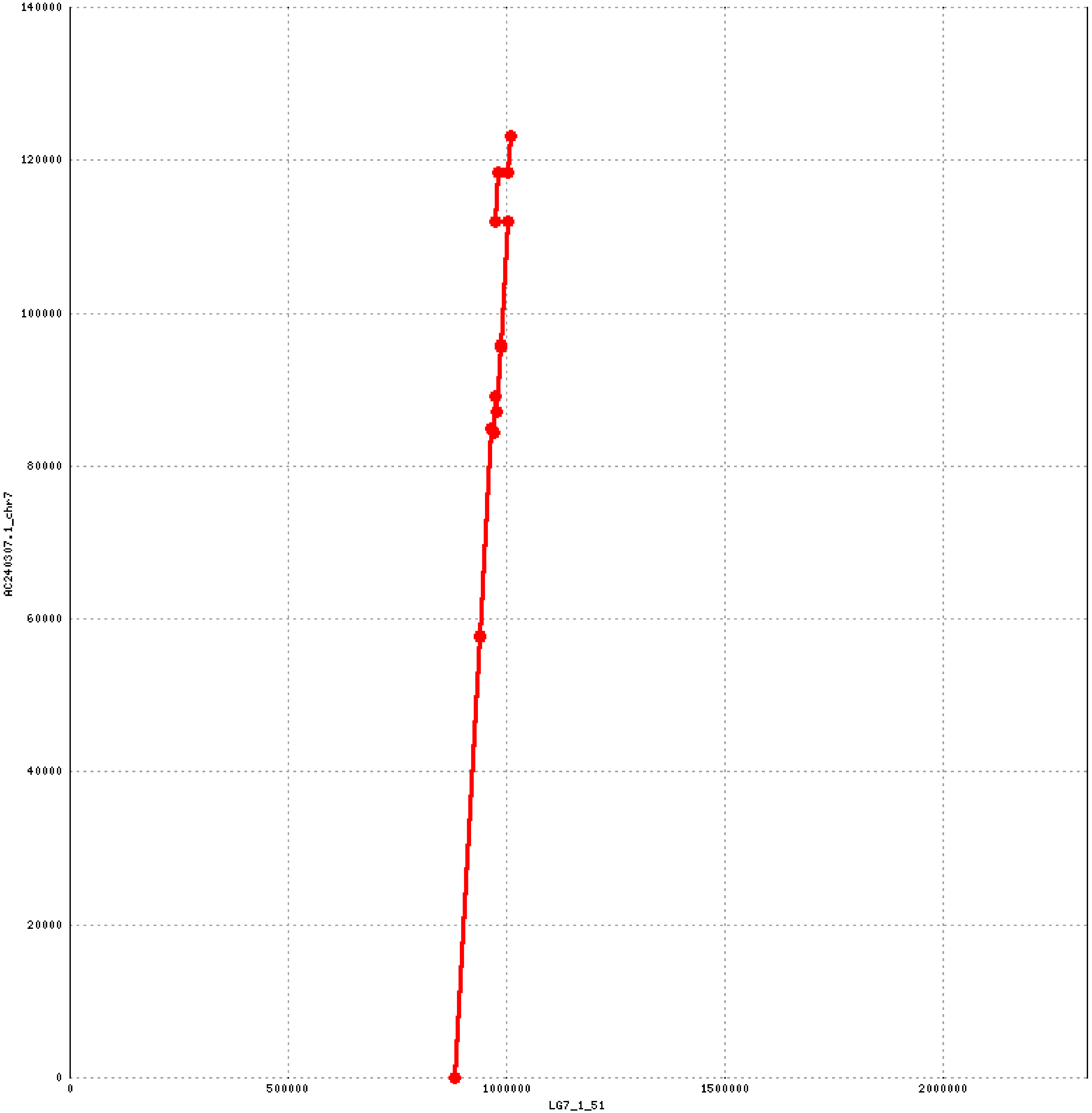
140000
120000
100000
80000
60000
40000
20000
0
AC240307.1_chr7
0
500000
1000000
1500000
2000000
LG7_1_51

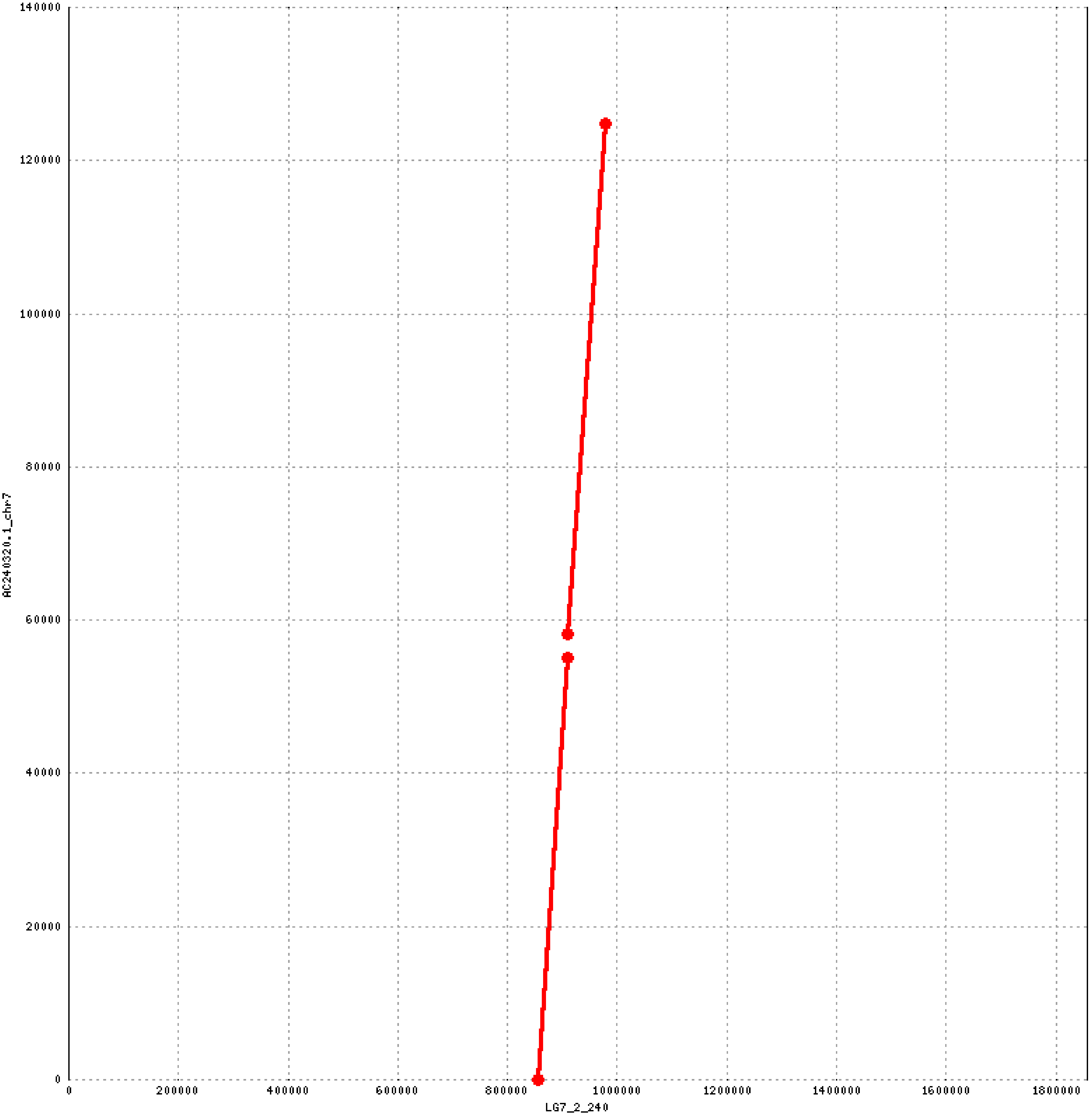

140000
120000
100000
80000
60000
40000
20000
0
AC240320.1_chr7
0
200000
400000
600000
800000
1000000
1200000
1400000
1600000
1800000
LG7_2_240

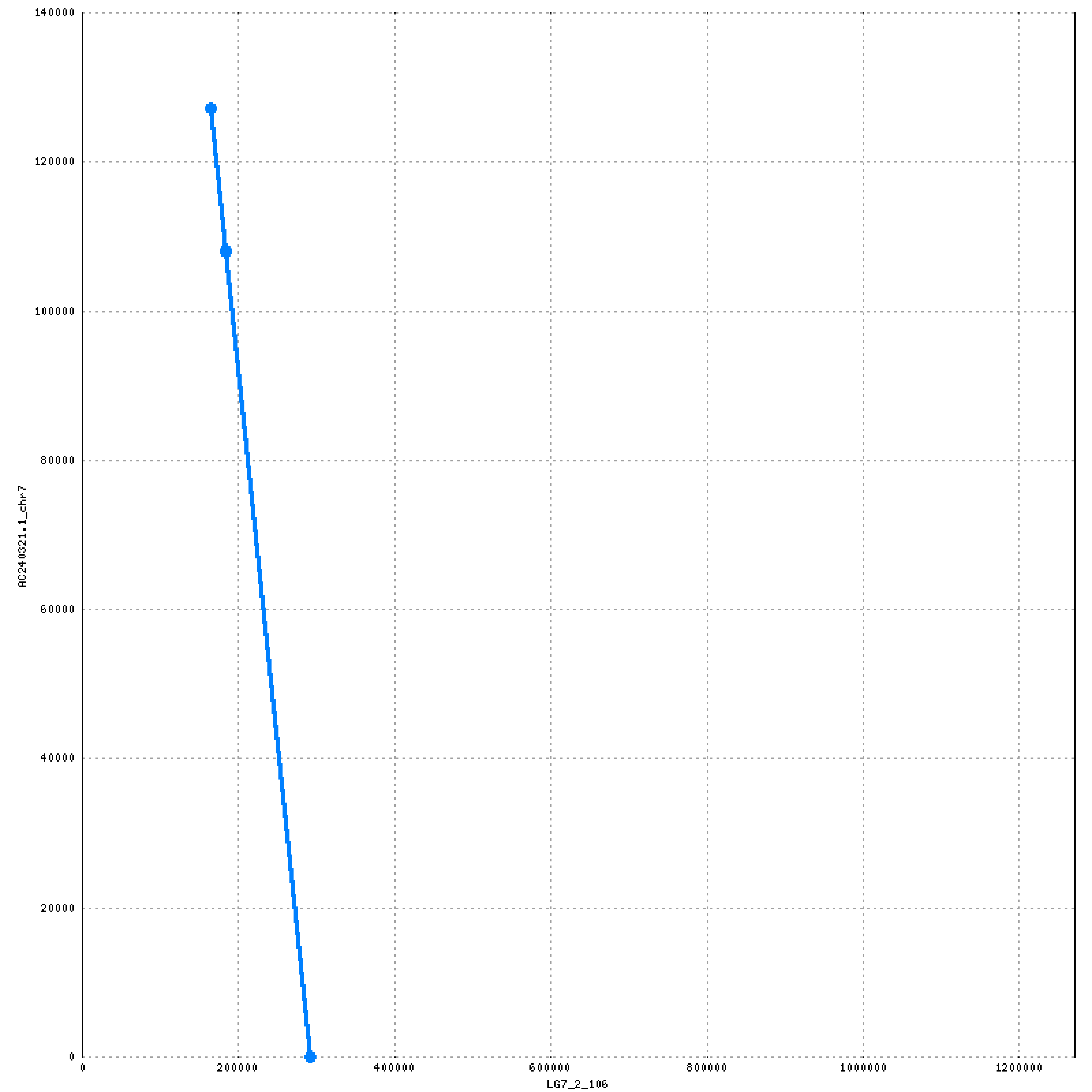

140000
120000
100000
80000
60000
40000
20000
0
AC240321.1_chr7
0
200000
400000
600000
800000
1000000
1200000
LG7_2_106

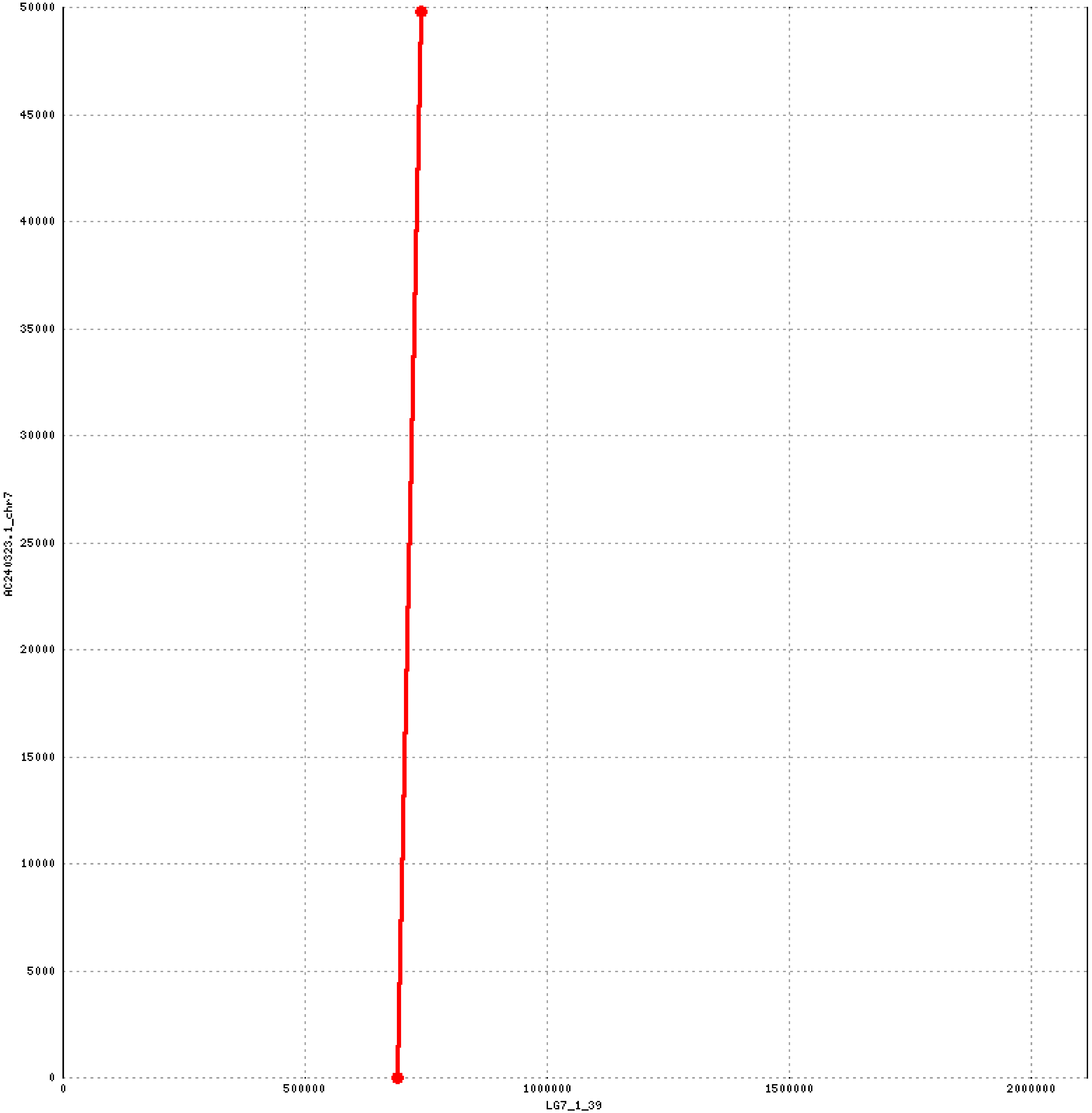

50000
45000
40000
35000
30000
25000
20000
15000
10000
5000
0
AC240323.1_chr7
0
500000
1000000
1500000
2000000
LG7_1_39

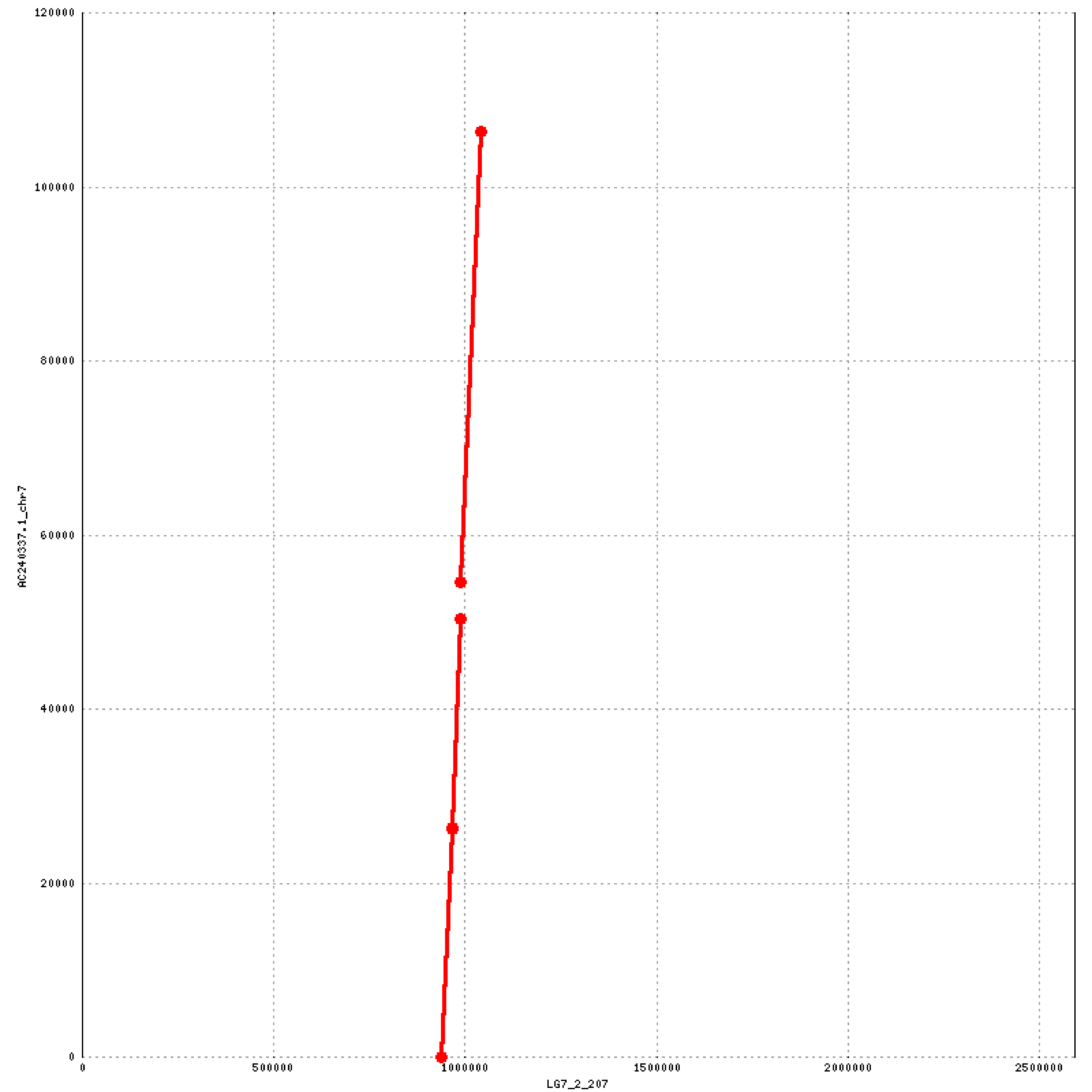

120000
100000
80000
60000
40000
20000
0
AC240337.1_chr7
0
500000
1000000
1500000
2000000
2500000
LG7_2_207

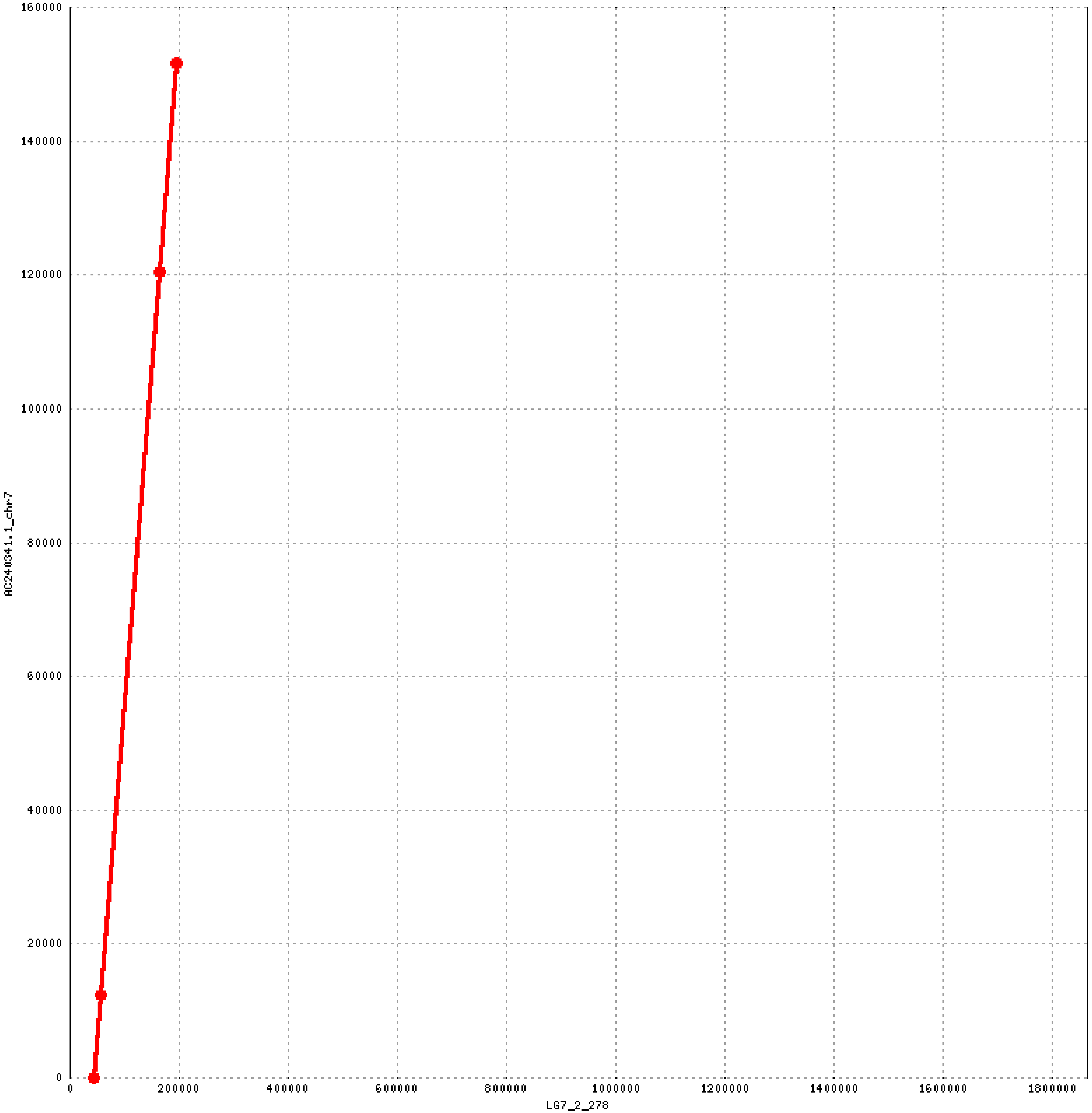

160000
140000
120000
100000
80000
60000
40000
20000
0
AC240341.1_chr7
0
200000
400000
600000
800000
1000000
1200000
1400000
1600000
1800000
LG7_2_278

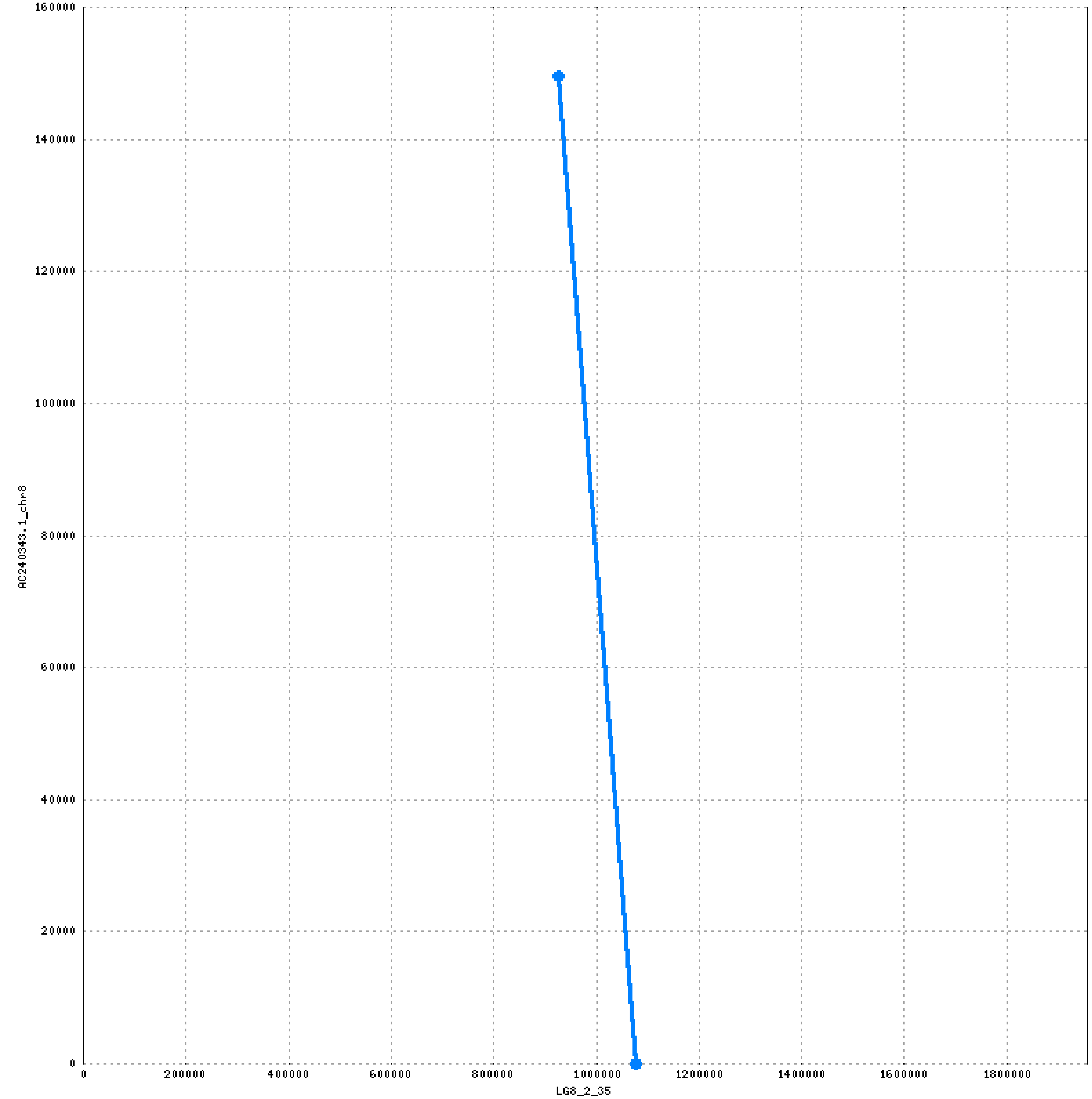

160000
140000
120000
100000
80000
60000
40000
20000
0
AC240343.1_chr8
0
200000
400000
600000
800000
1000000
1200000
1400000
1600000
1800000
LG8_2_35

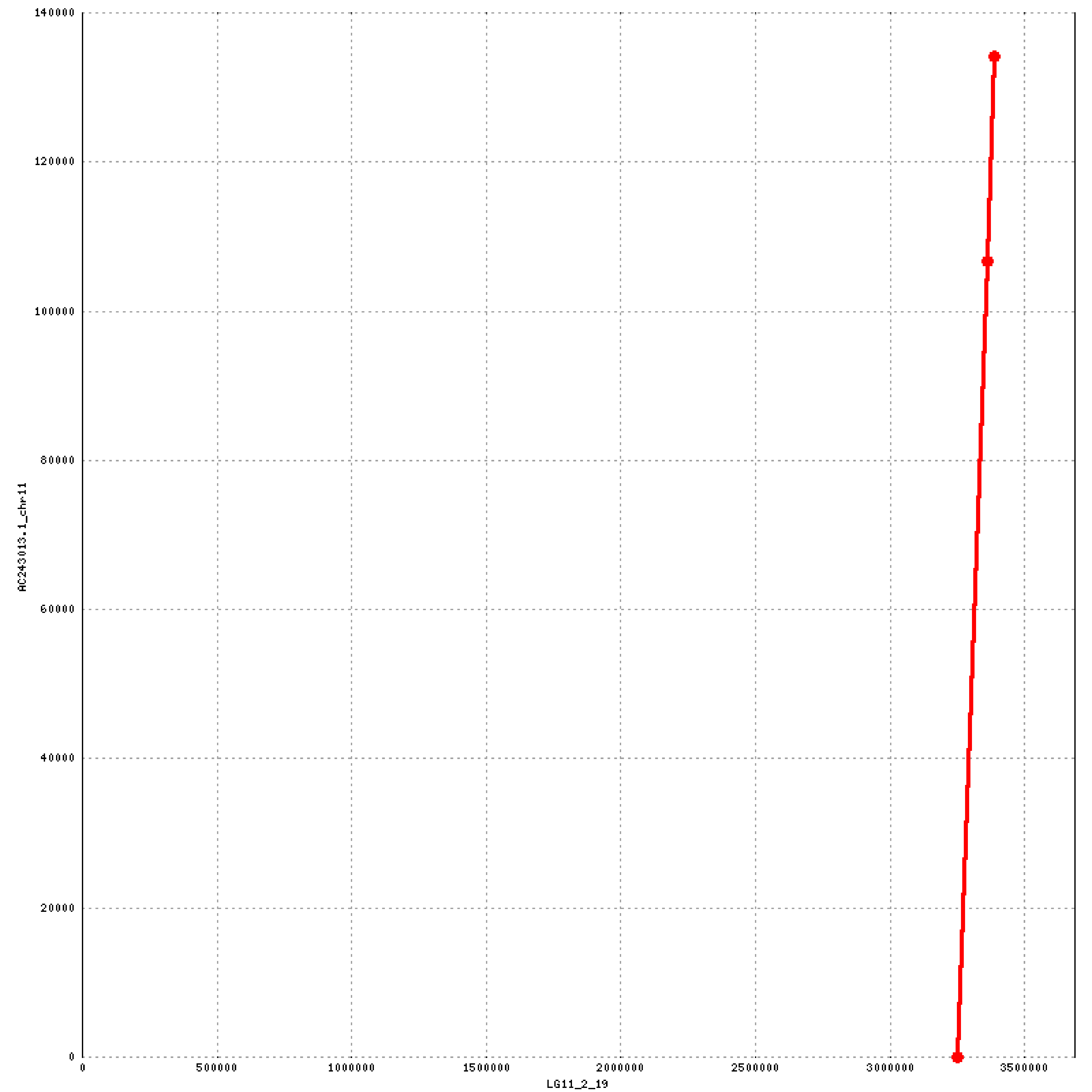
140000
120000
100000
80000
60000
40000
20000
0
AC243013.1_chr11
0
500000
1000000
1500000
2000000
2500000
3000000
3500000
LG11_2_19

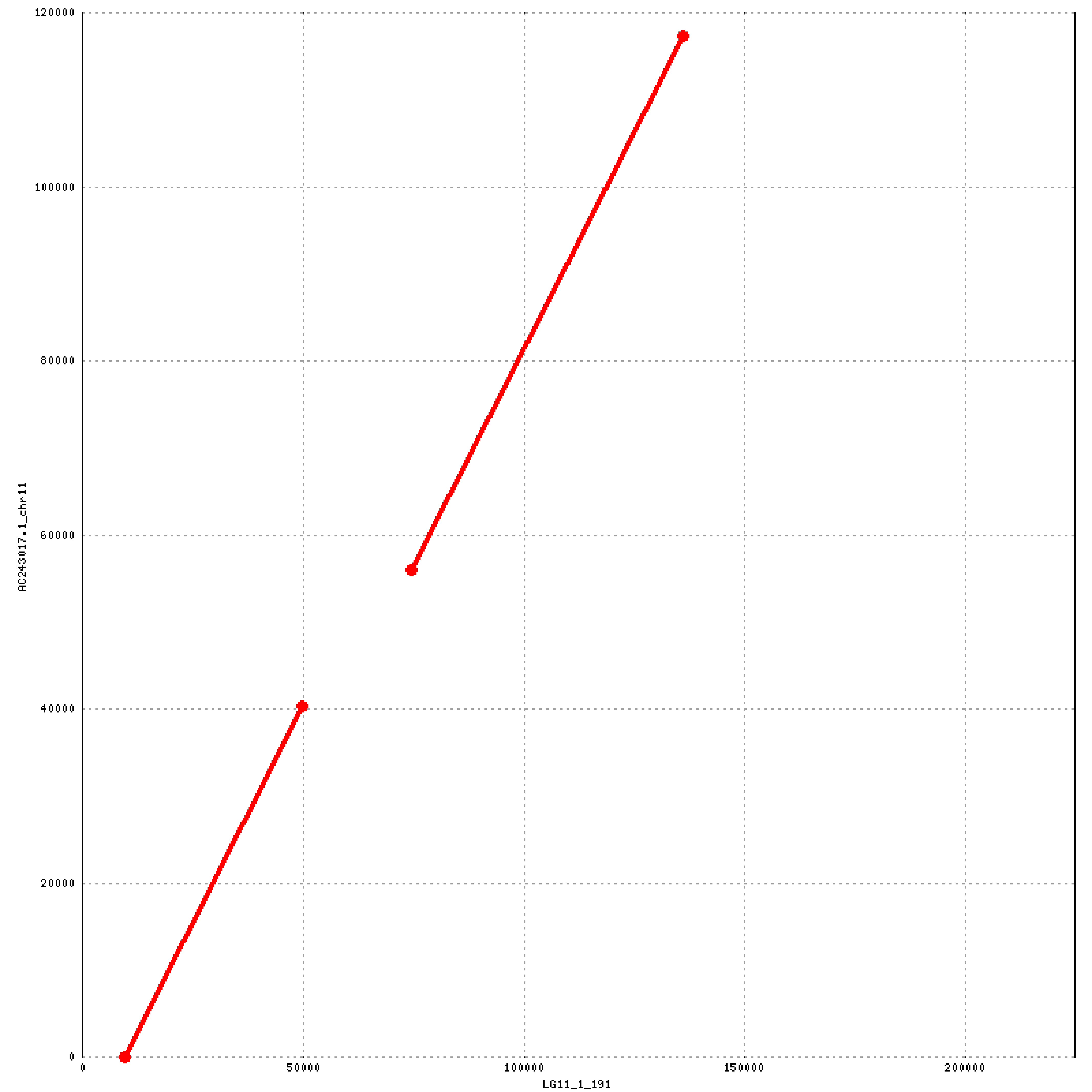
120000
100000
80000
60000
40000
20000
0
AC243017.1_chr11
0
50000
100000
150000
200000
LG11_1_191

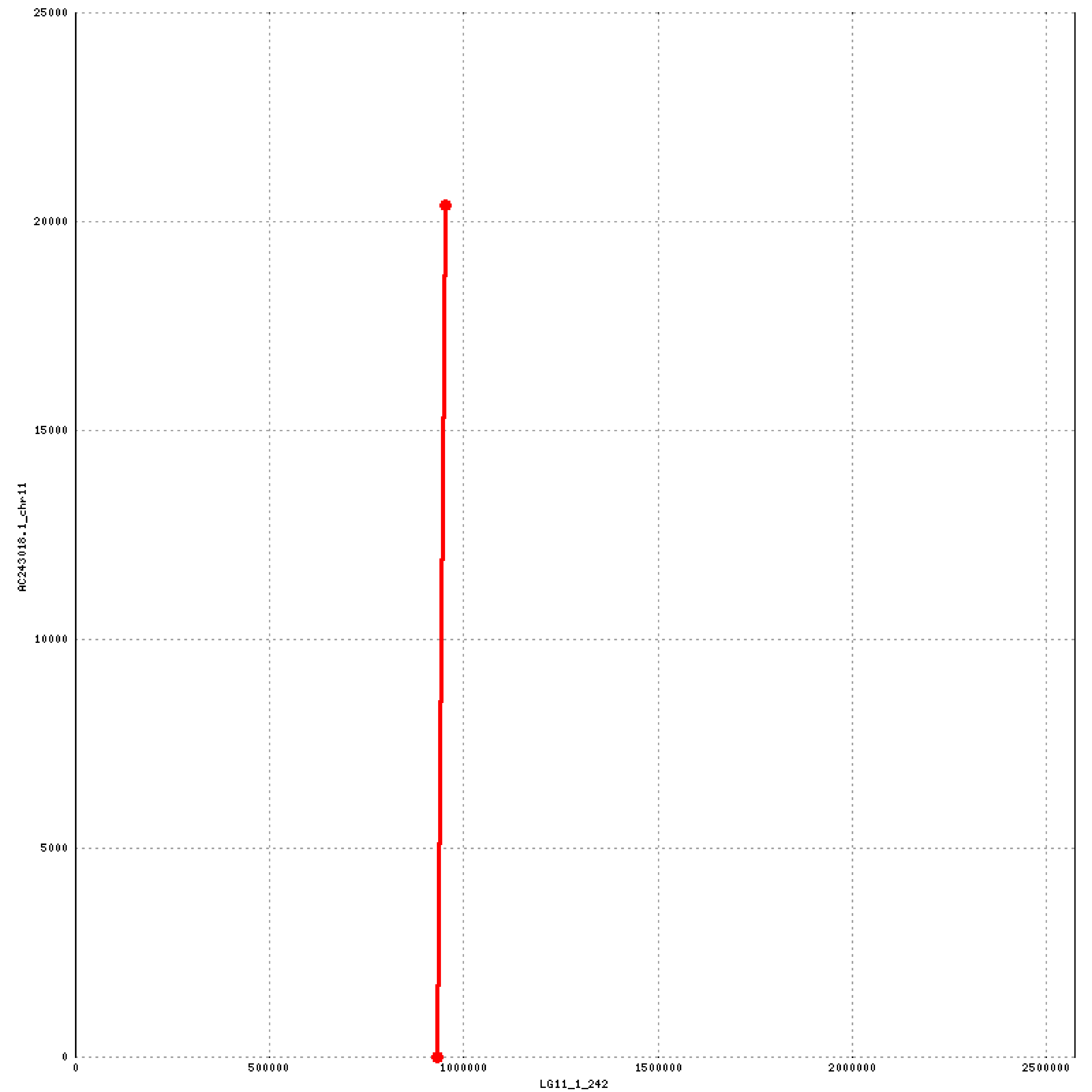
25000
20000
15000
10000
5000
0
AC243018.1_chr11
0
500000
1000000
1500000
2000000
2500000
LG11_1_242

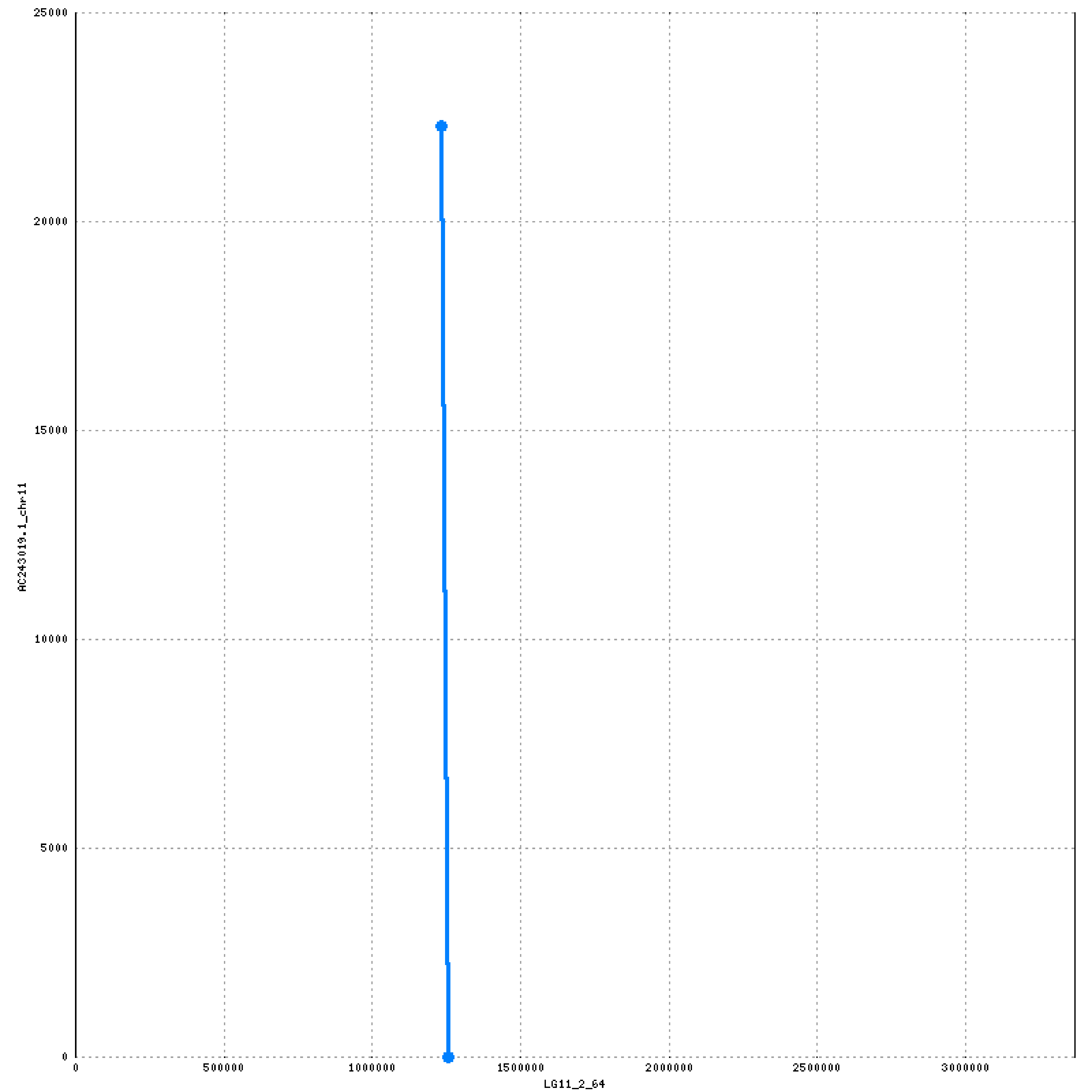

25000
20000
15000
10000
5000
0
AC243019.1_chr11
0
500000
1000000
1500000
2000000
2500000
3000000
LG11_2_64

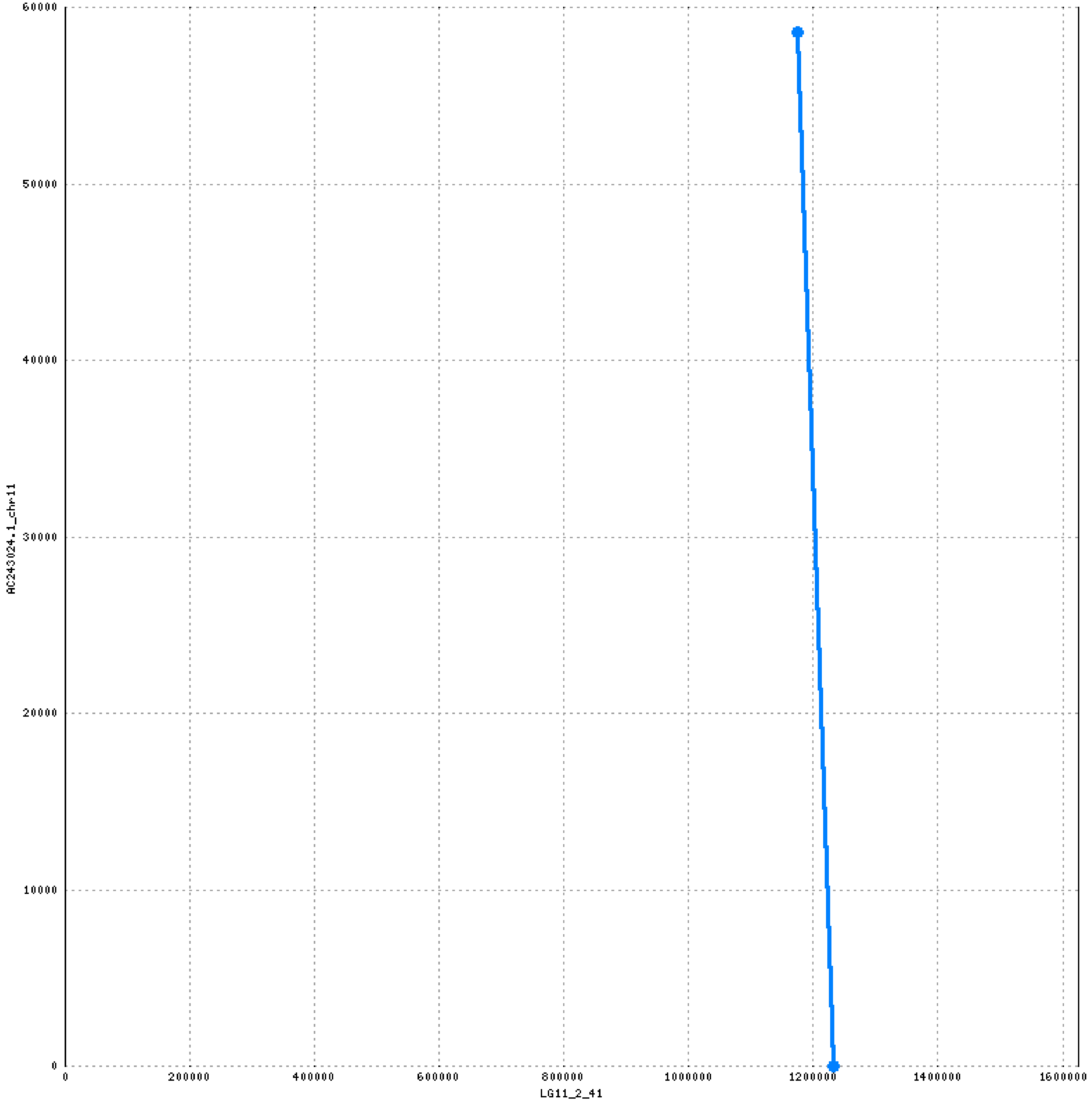

60000
50000
40000
30000
20000
10000
0
AC243024.1_chr11
0
200000
400000
600000
800000
1000000
1200000
1400000
1600000
LG11_2_41

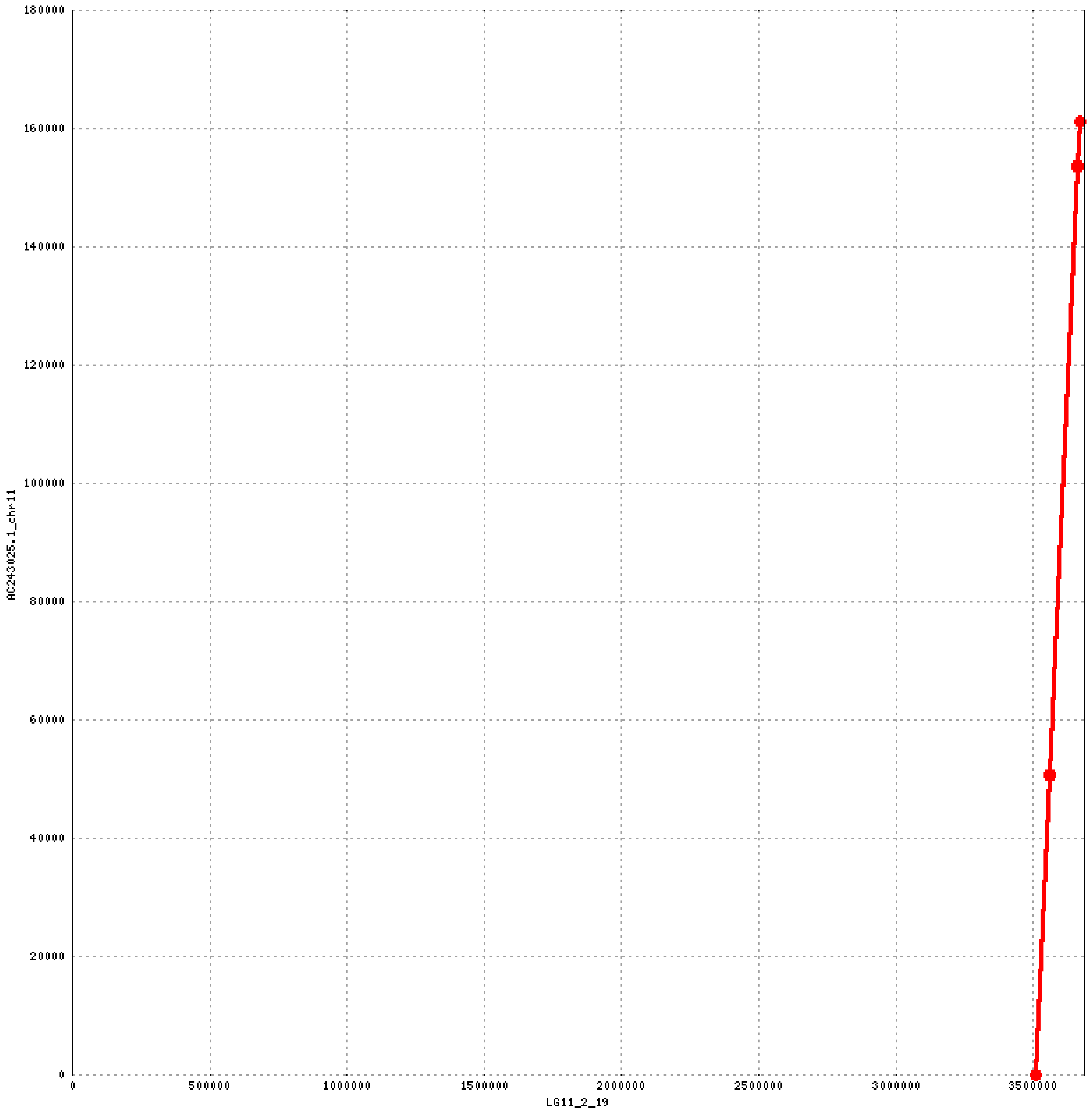

180000
160000
140000
120000
100000
80000
60000
40000
20000
0
AC243025.1_chr11
0
500000
1000000
1500000
2000000
2500000
3000000
3500000
LG11_2_19

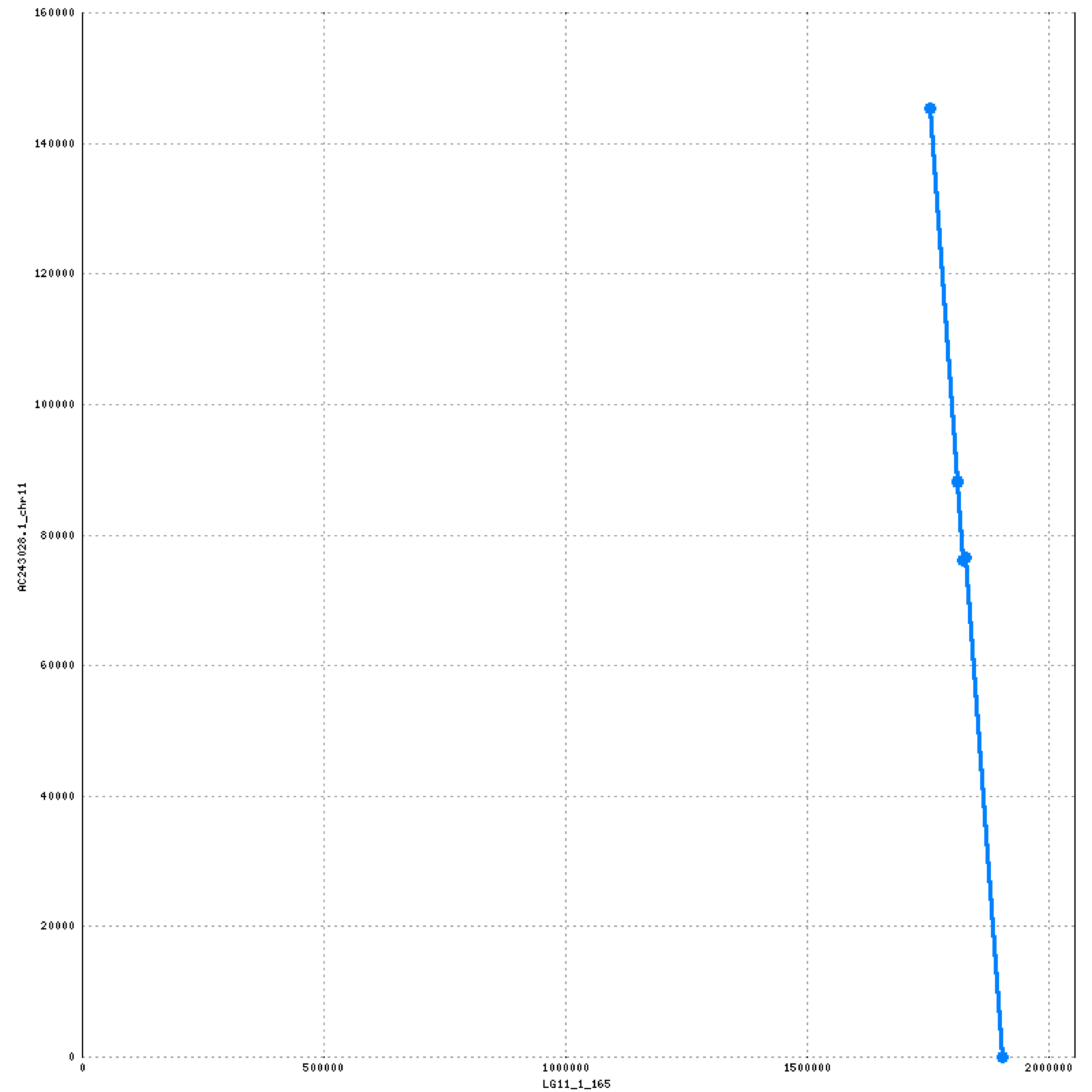

160000
140000
120000
100000
80000
60000
40000
20000
0
AC243028.1_chr11
0
500000
1000000
1500000
2000000
LG11_1_165

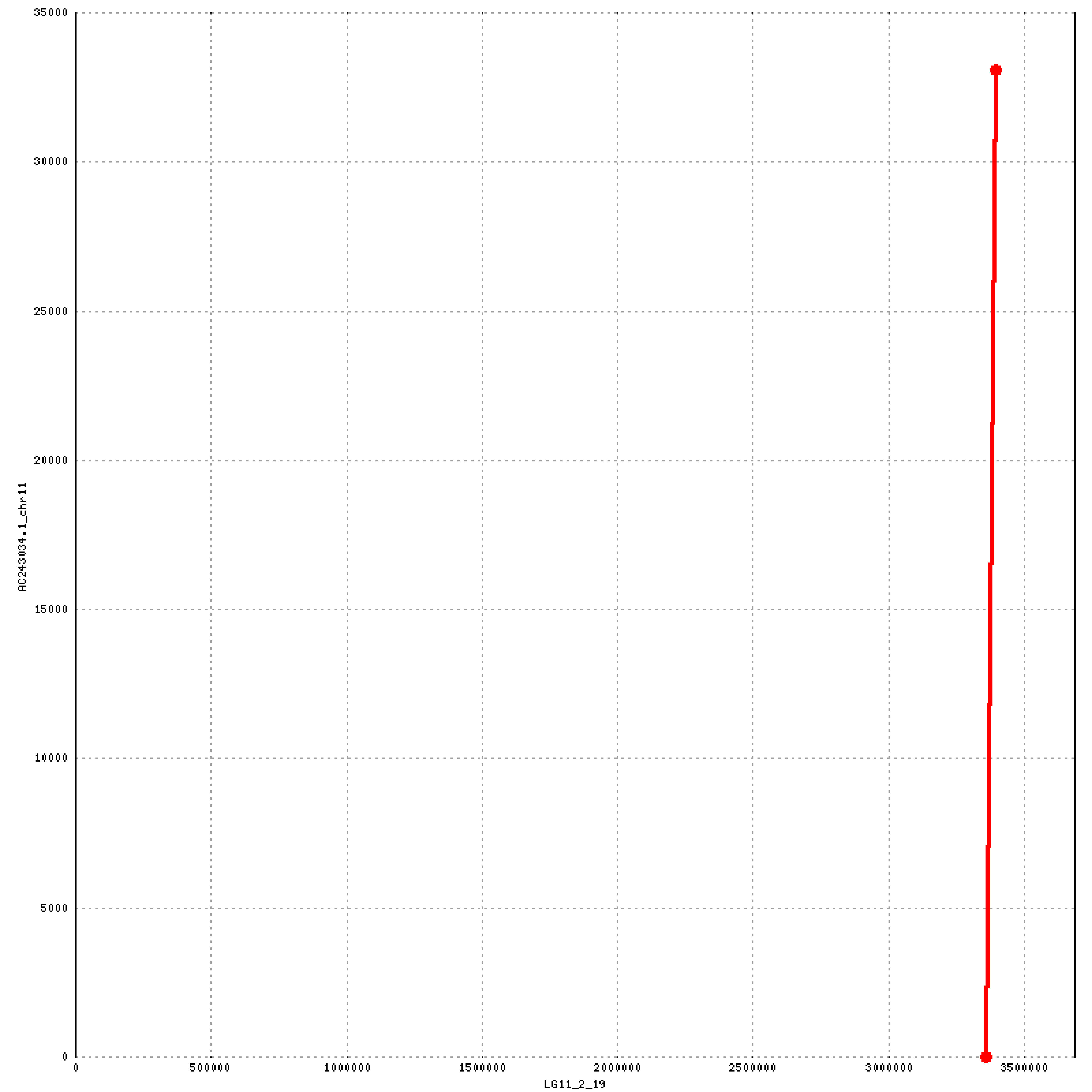

AC243034.1_chr11
LG11_2_19
0
5000
10000
15000
20000
25000
30000
35000
0
500000
1000000
1500000
2000000
2500000
3000000
3500000

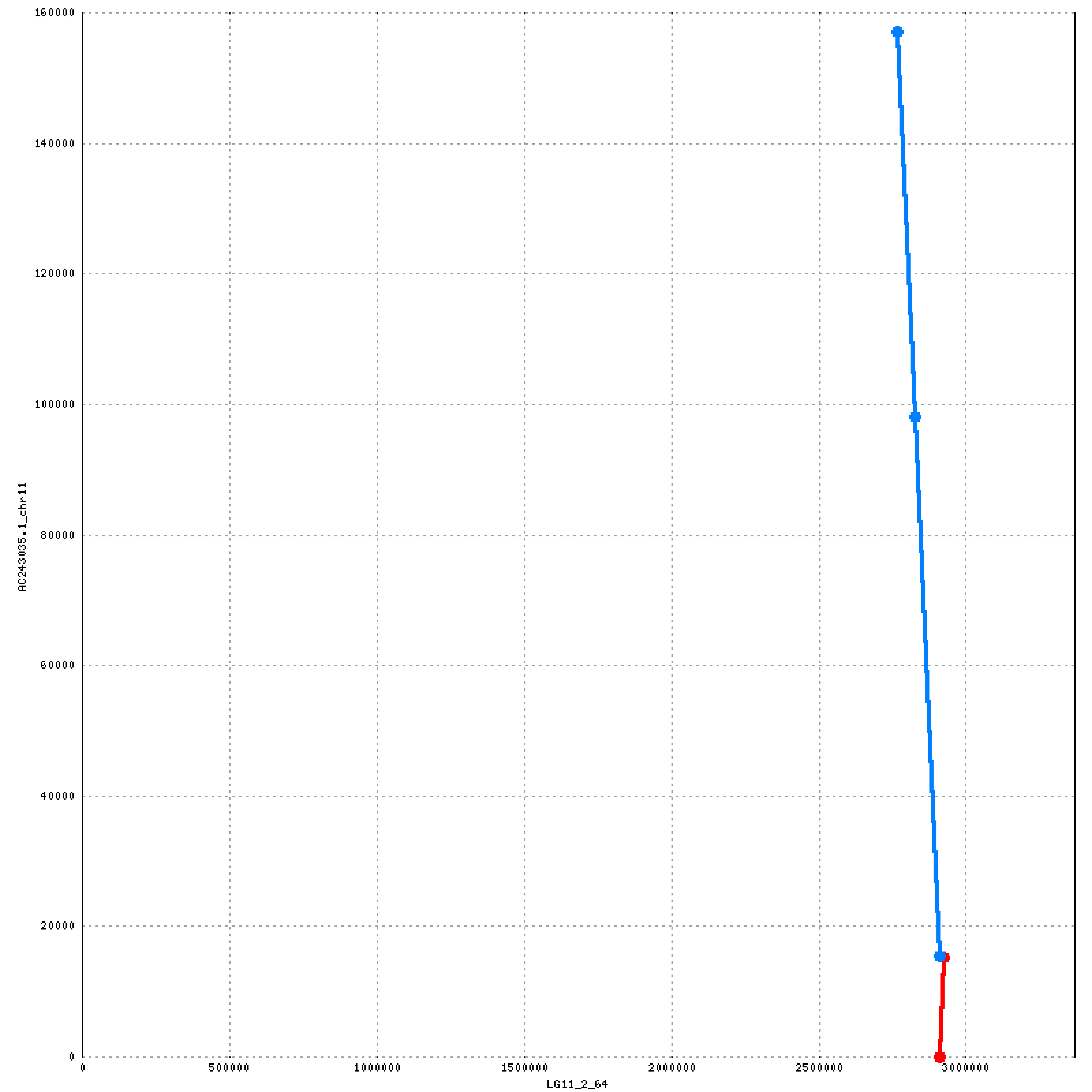

160000
140000
120000
100000
80000
60000
40000
20000
0
AC243035.1_chr11
0
500000
1000000
1500000
2000000
2500000
3000000
LG11_2_64

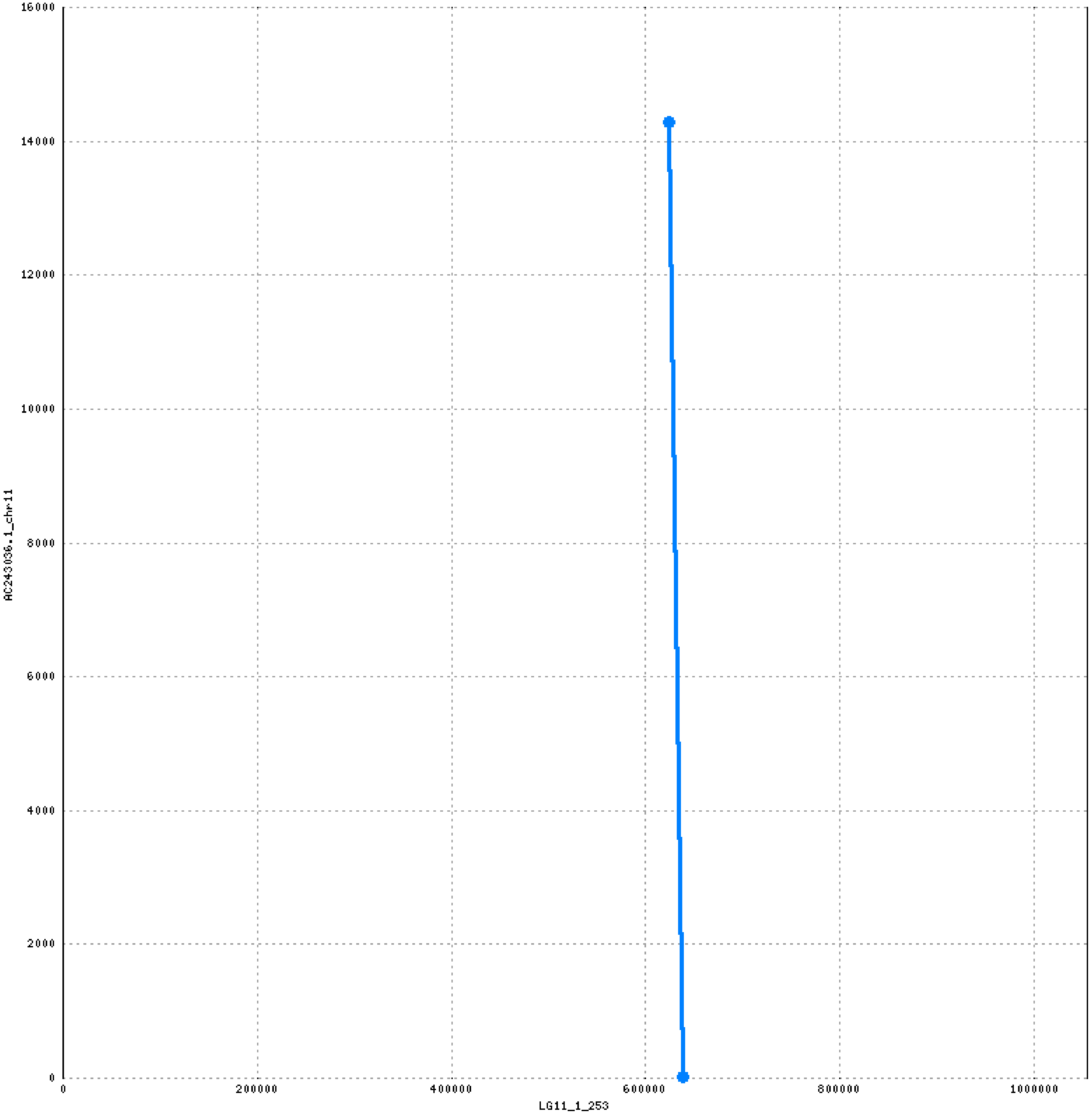

16000
14000
12000
10000
8000
6000
4000
2000
0
0
200000
400000
600000
800000
1000000
LG11_1_253
AC243036.1_chr11

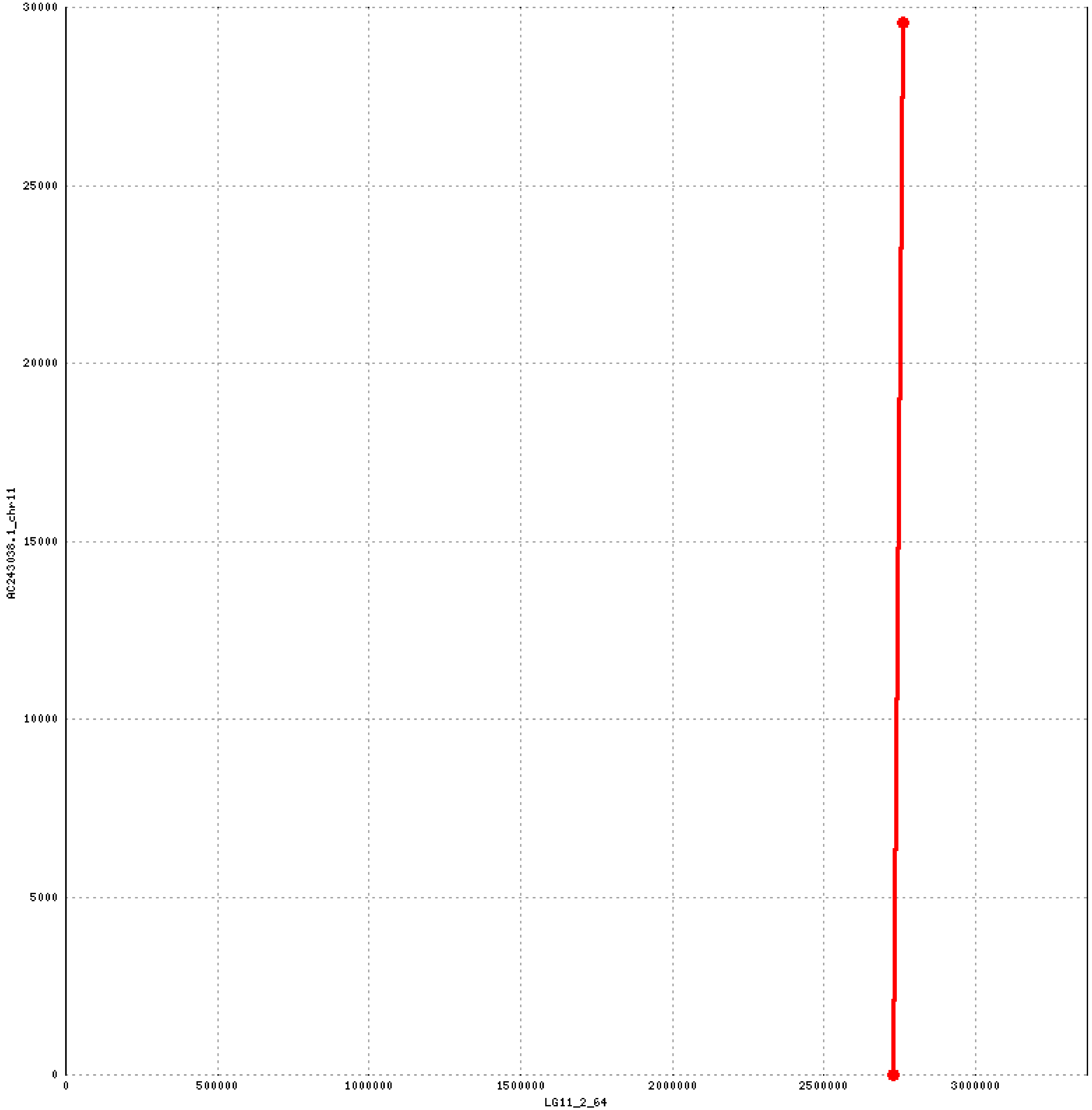

30000
25000
20000
15000
10000
5000
0
AC243038.1_chr11
0
500000
1000000
1500000
2000000
2500000
3000000
LG11_2_64

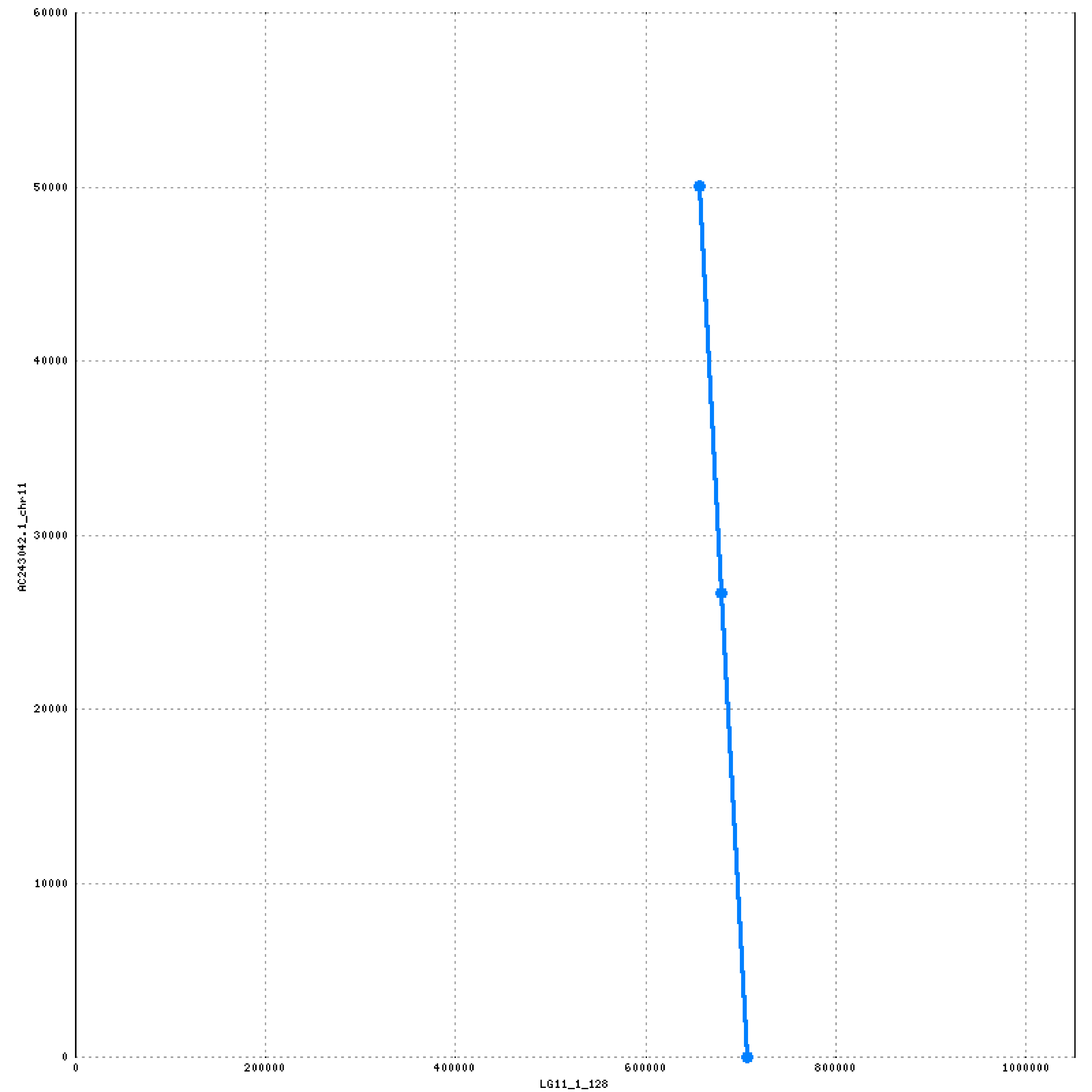
60000
50000
40000
30000
20000
10000
0
AC243042.1_chr11
0
200000
400000
600000
800000
1000000
LG11_1_128

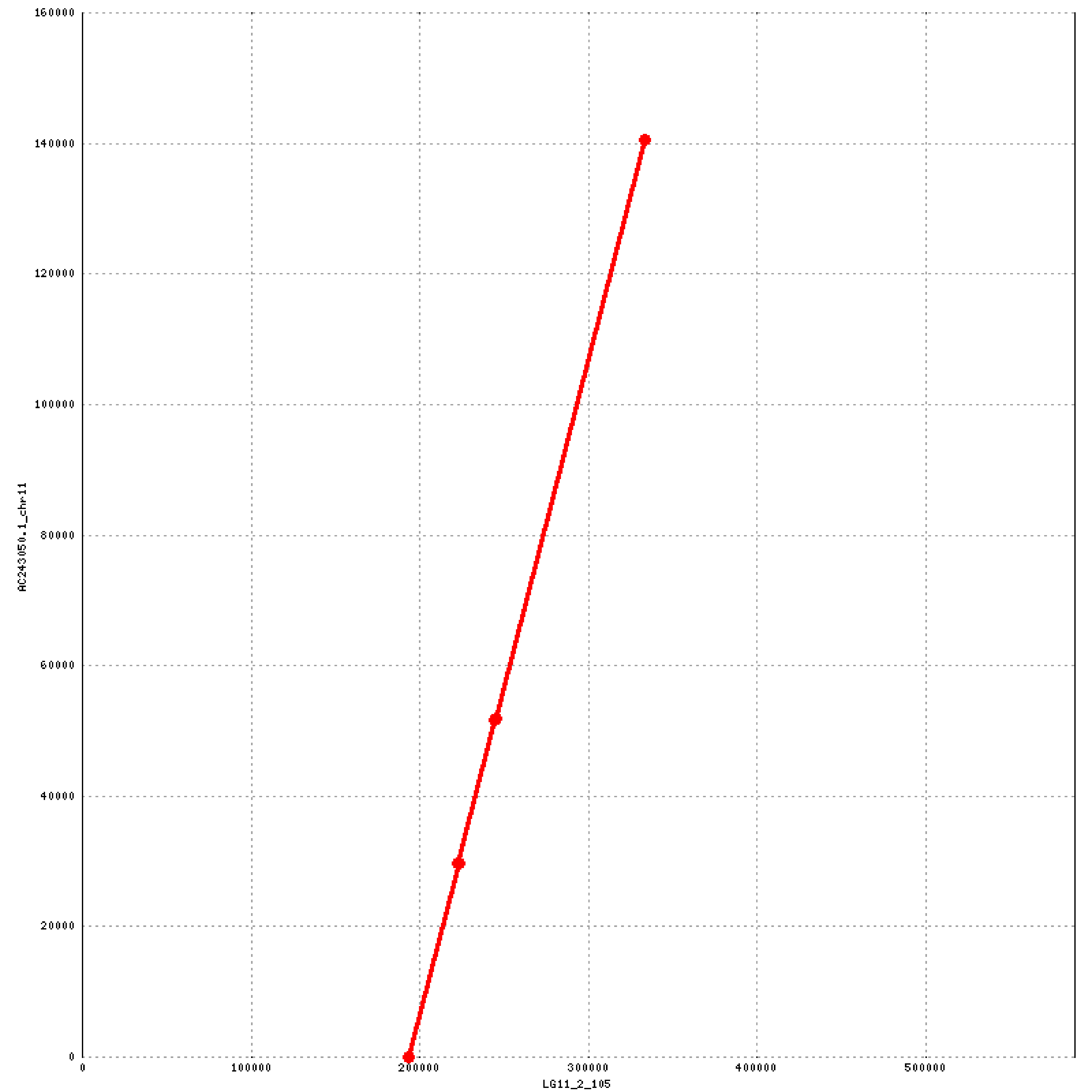
160000
140000
120000
100000
80000
60000
40000
20000
0
AC243050.1_chr11
0
100000
200000
300000
400000
500000
LG11_2_105

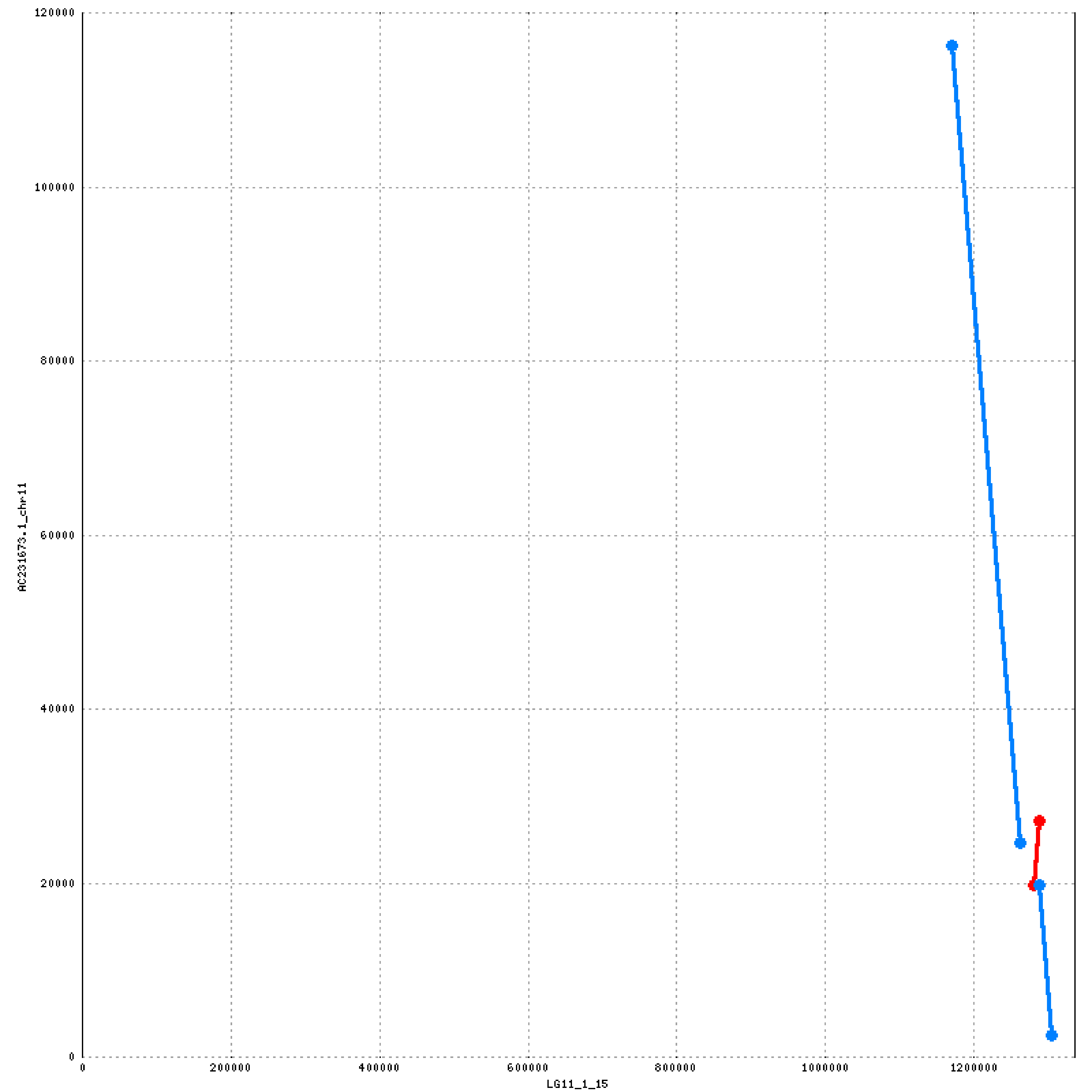
120000
100000
80000
60000
40000
20000
0
AC231673.1_chr11
0
200000
400000
600000
800000
1000000
1200000
LG11_1_15

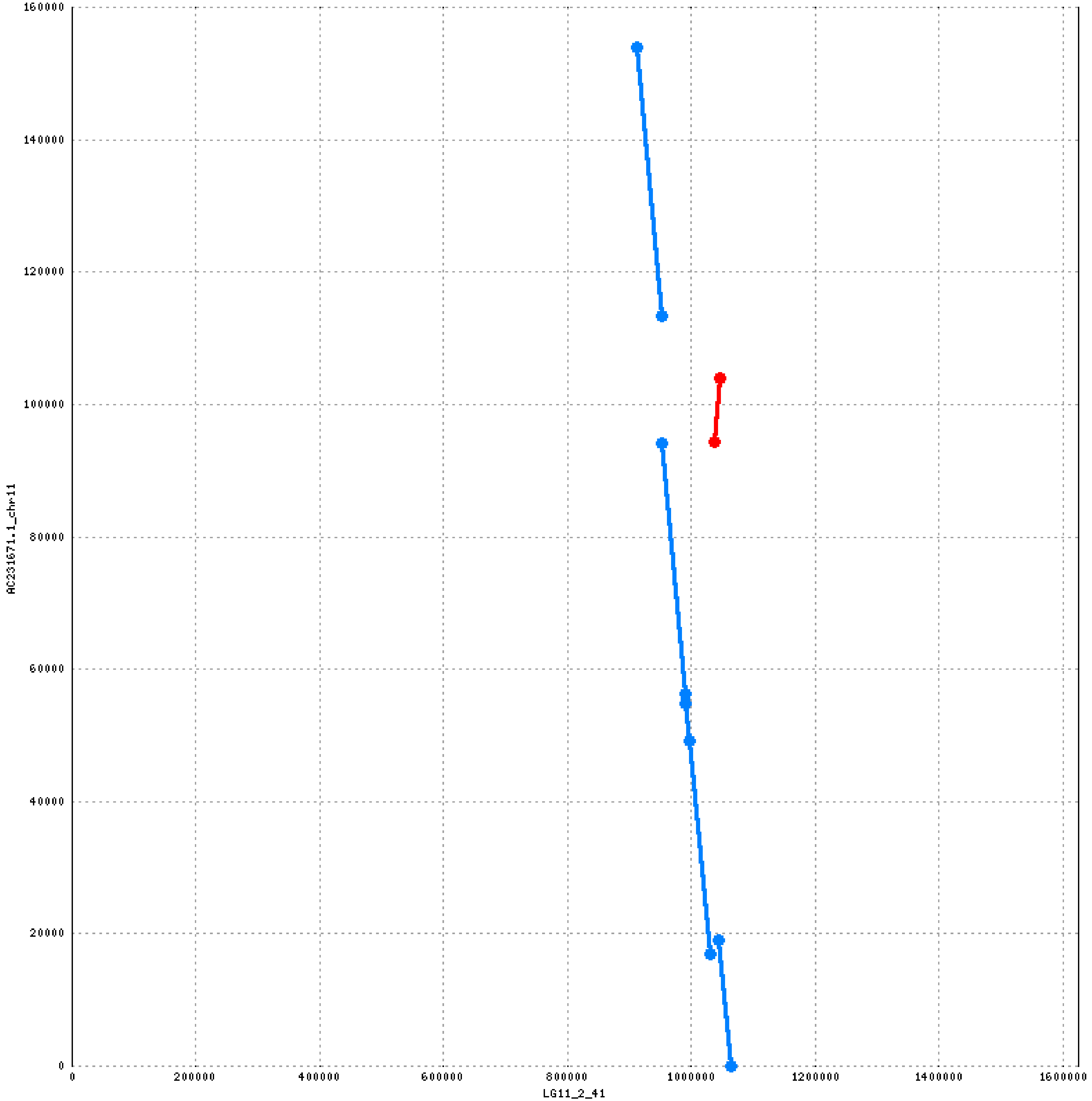

160000
140000
120000
100000
80000
60000
40000
20000
0
AC231671.1_chr11
0
200000
400000
600000
800000
1000000
1200000
1400000
1600000
LG11_2_41

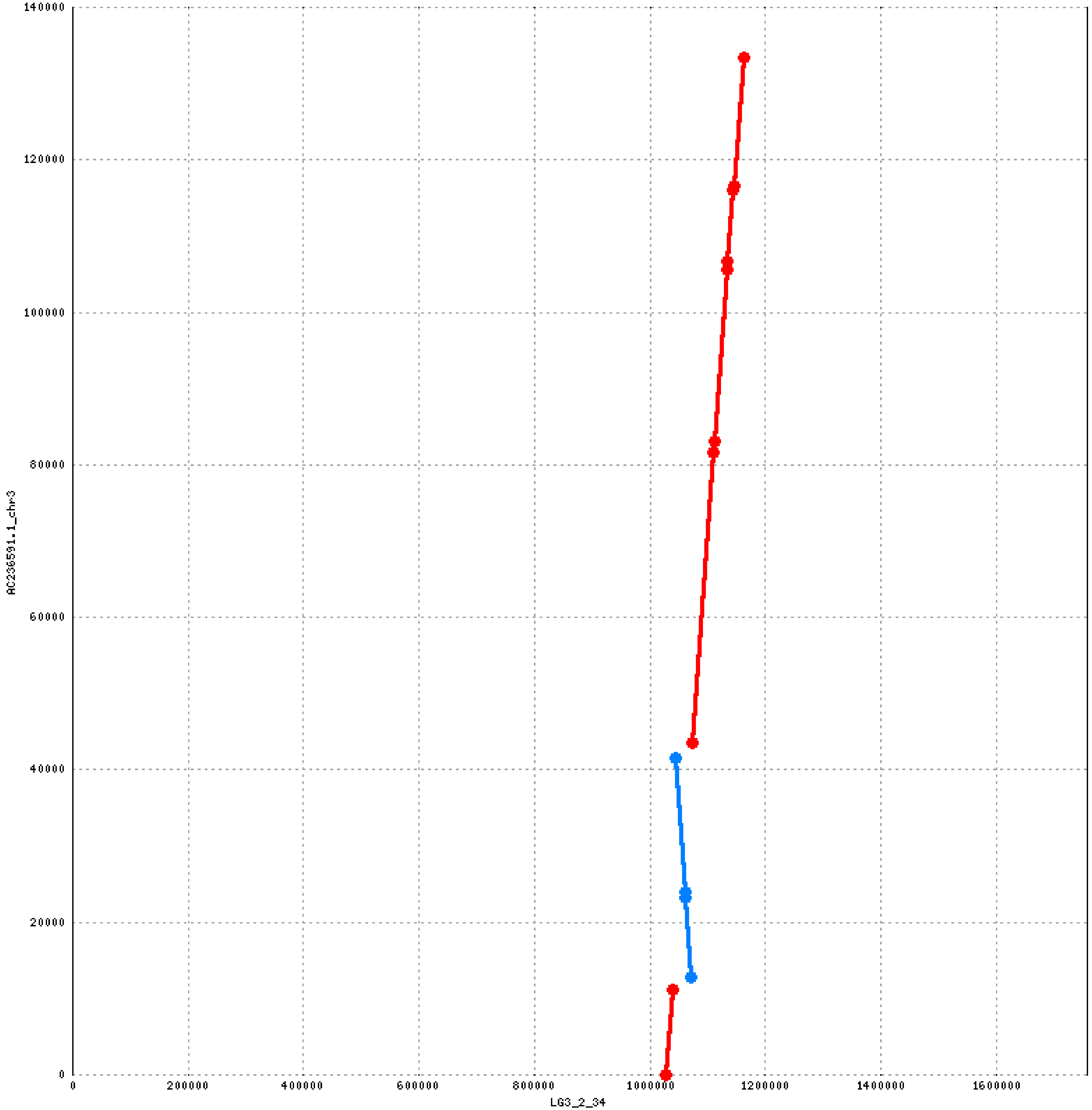

140000
120000
100000
80000
60000
40000
20000
0
AC236591.1_chr3
0
200000
400000
600000
800000
1000000
1200000
1400000
1600000
LG3_2_34

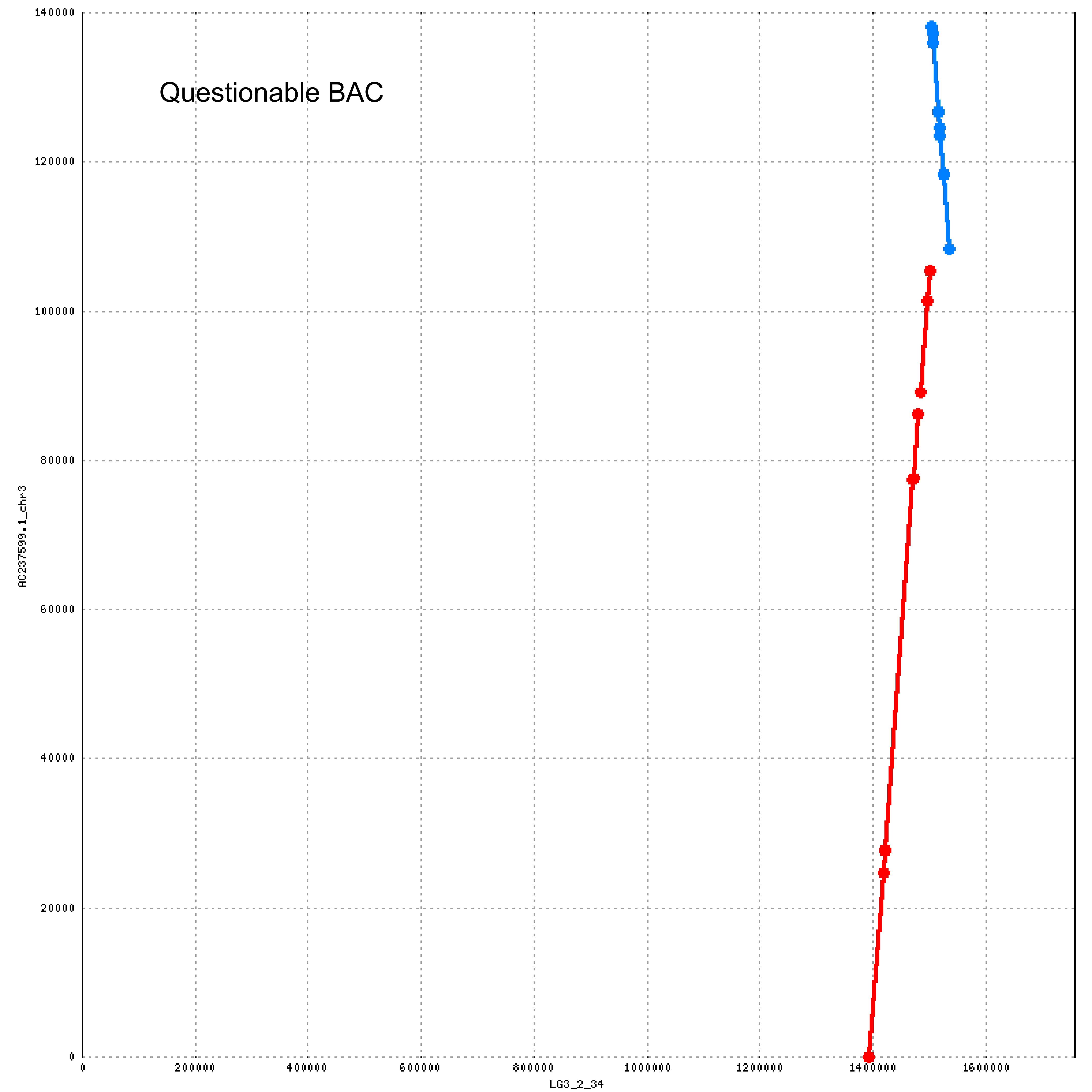

Questionable BAC
140000
120000
100000
80000
60000
40000
20000
0
AC237599.1_chr3
0
200000
400000
600000
800000
1000000
1200000
1400000
1600000
LG3_2_34

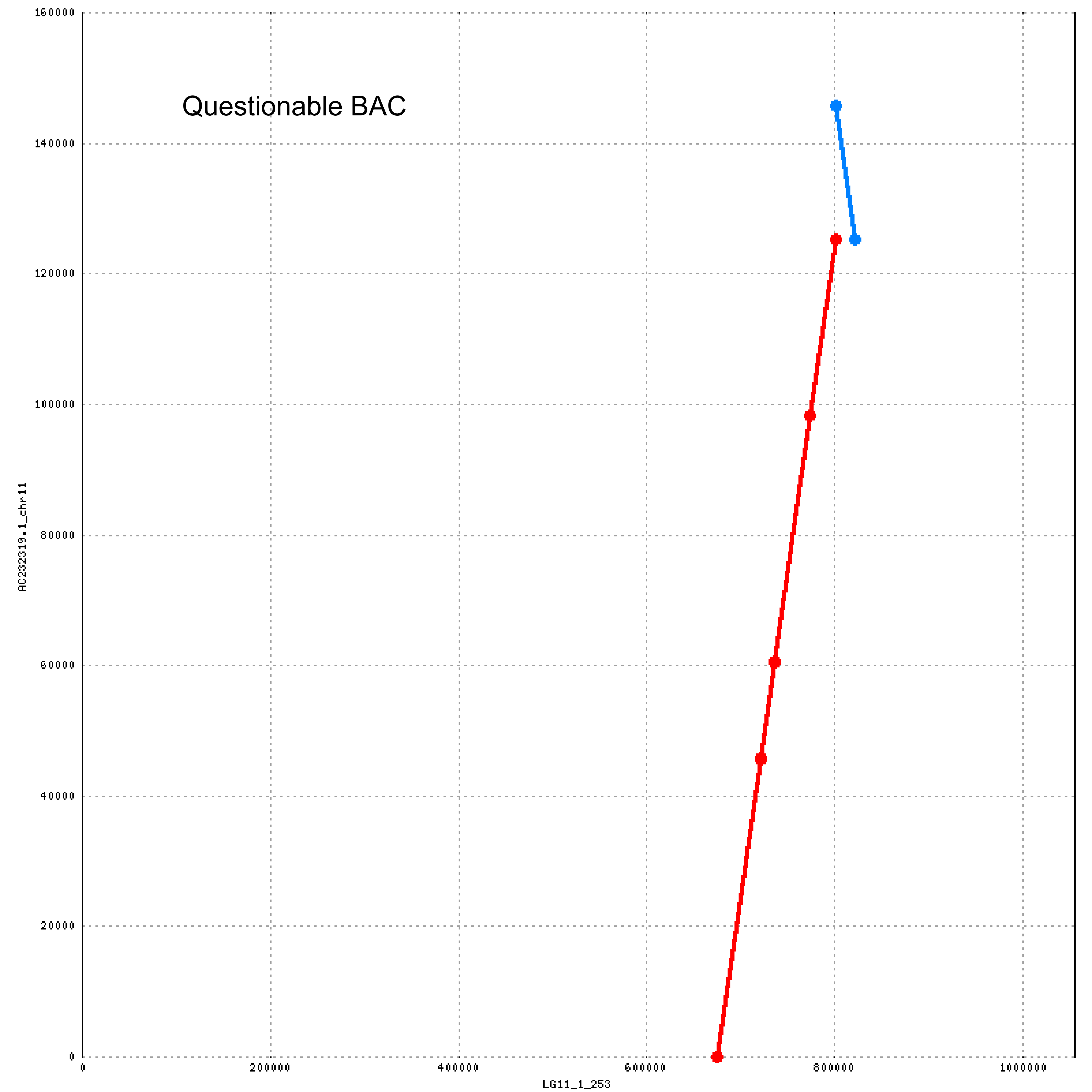

Questionable BAC
160000
140000
120000
100000
80000
60000
40000
20000
0
AC232319.1_chr11
0
200000
400000
600000
800000
1000000
LG11_1_253

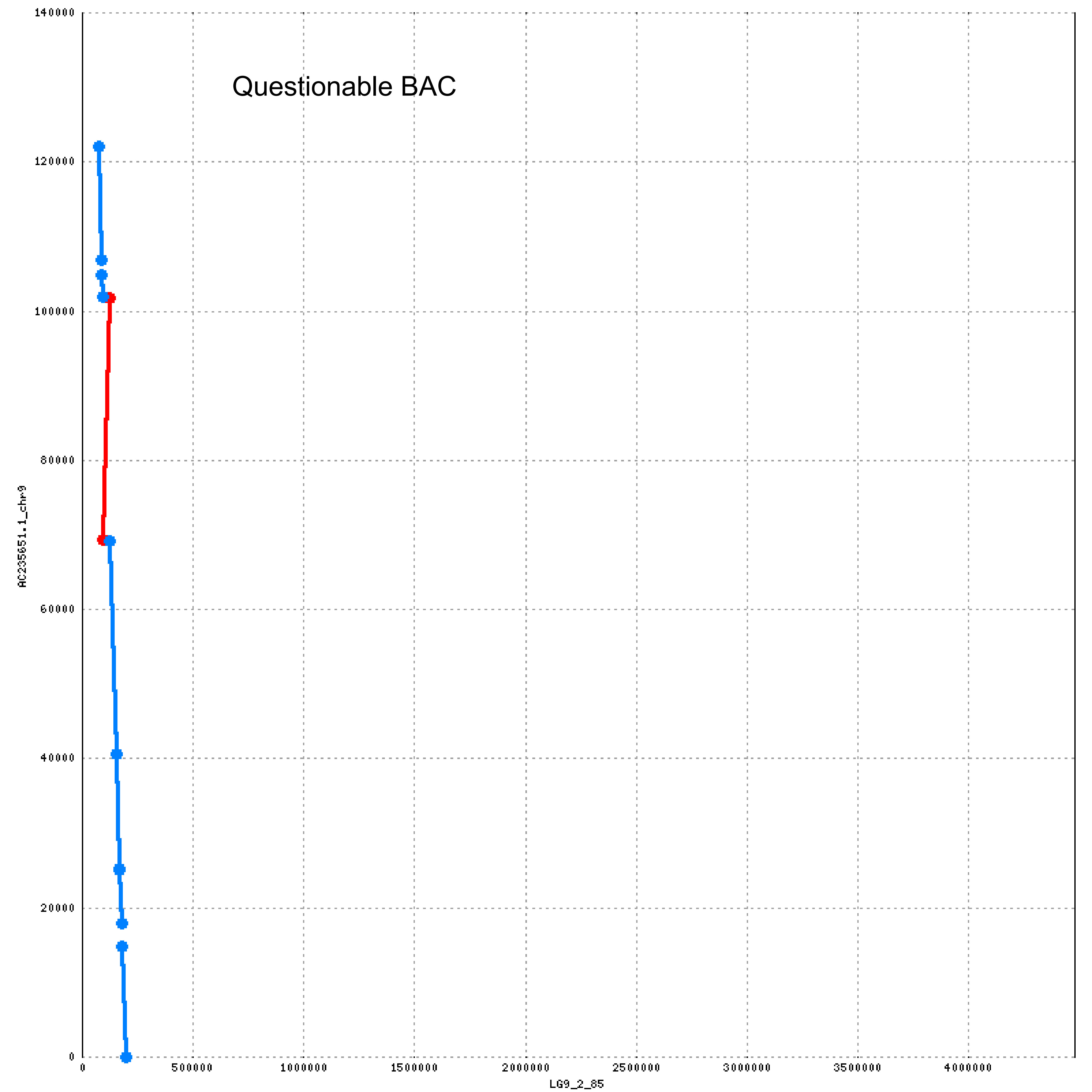

Questionable BAC
140000
120000
100000
80000
60000
40000
20000
0
AC235651.1_chr9
0
500000
1000000
1500000
2000000
2500000
3000000
3500000
4000000
LG9_2_85

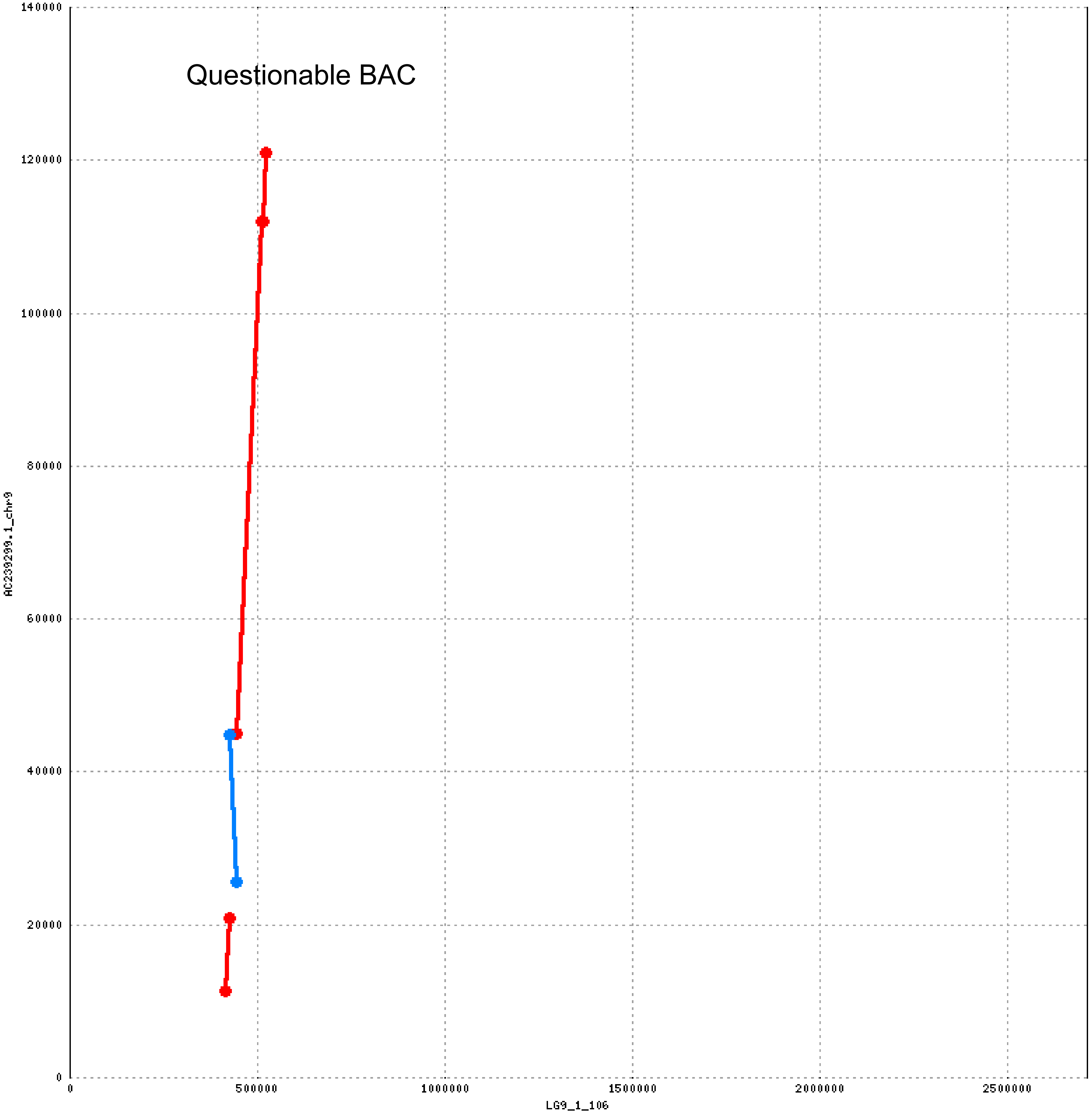

Questionable BAC
140000
120000
100000
80000
60000
40000
20000
0
AC239299.1_chr9
0
500000
1000000
1500000
2000000
2500000
LG9_1_106

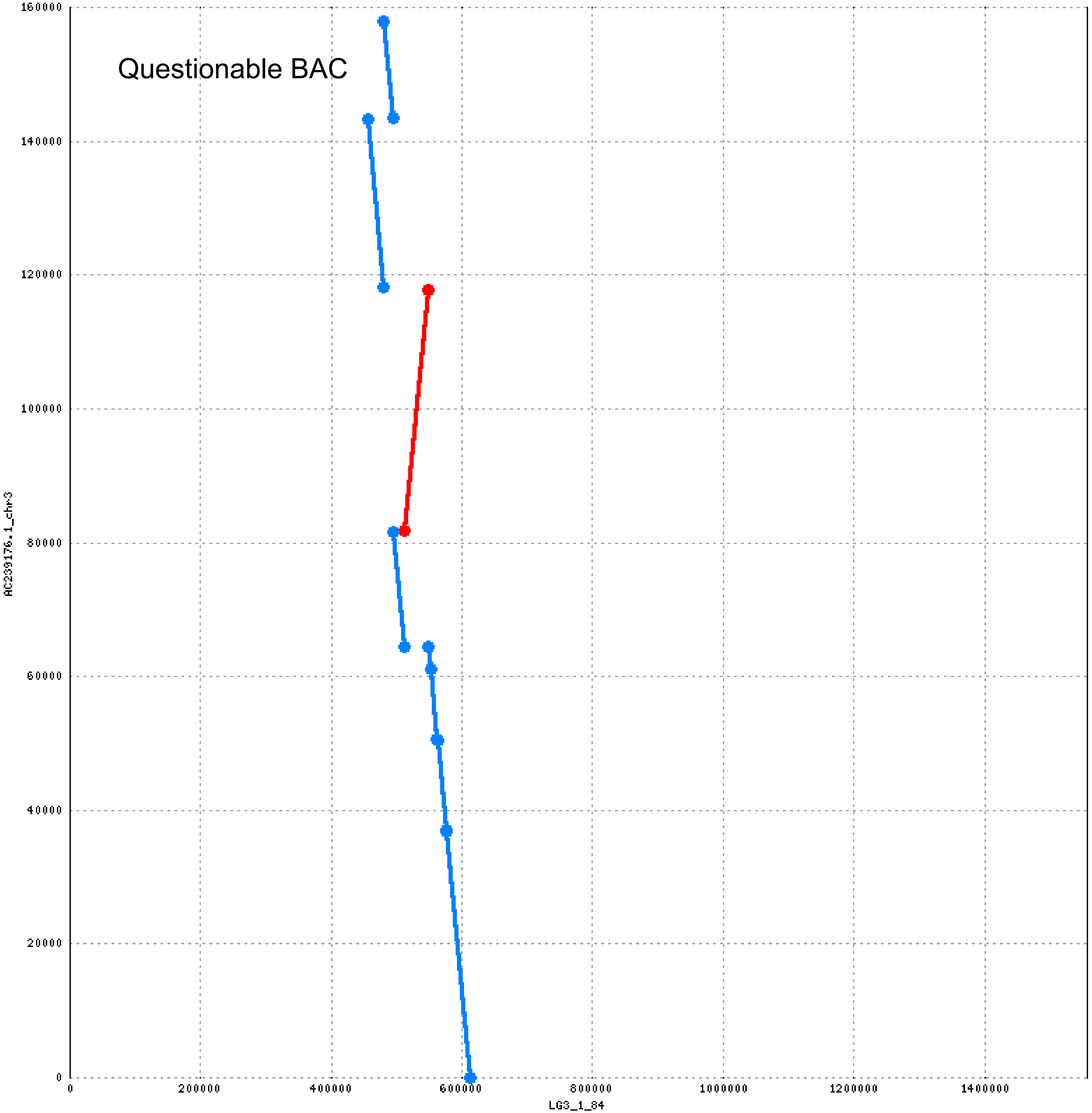

Questionable BAC
160000
140000
120000
100000
80000
60000
40000
20000
0
AC239176.1_chr3
0
200000
400000
600000
800000
1000000
1200000
1400000
LG3_1_84

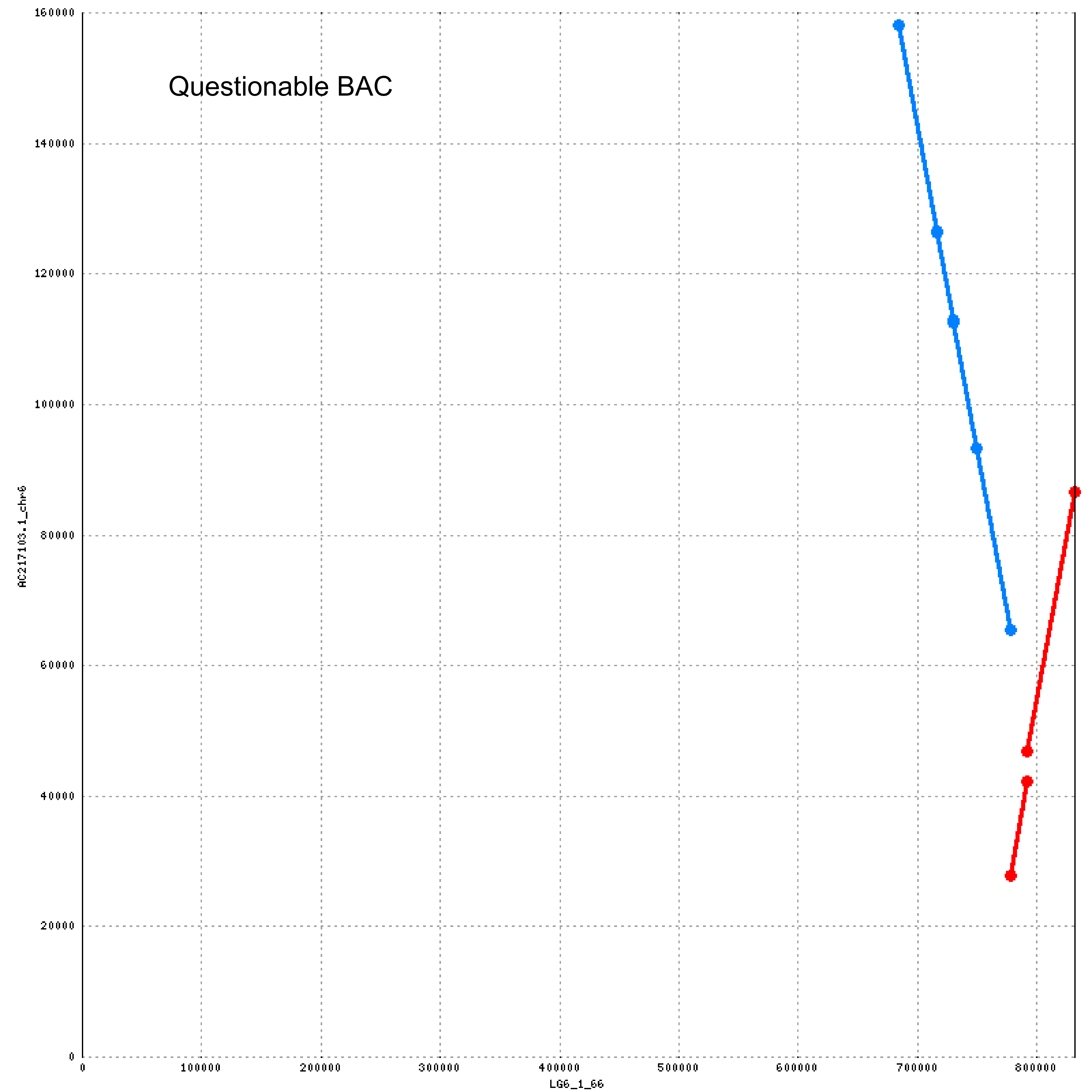

Questionable BAC
160000
140000
120000
100000
80000
60000
40000
20000
0
AC217103.1_chr6
0
100000
200000
300000
400000
500000
600000
700000
800000
LG6_1_66

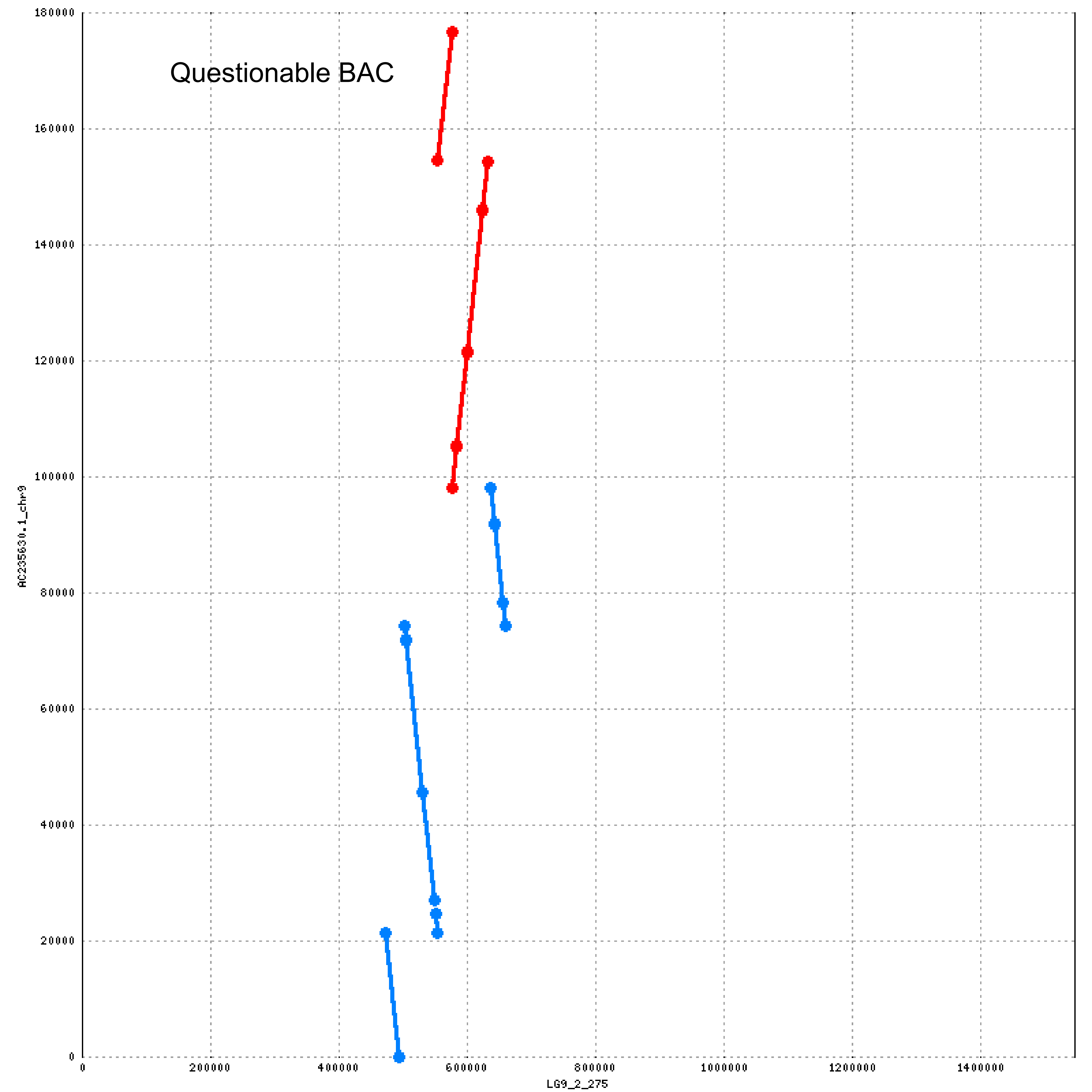
Questionable BAC
AC235630.1_chr9
LG9_2_275
0
20000
40000
60000
80000
100000
120000
140000
160000
180000
200000
400000
600000
800000
1000000
1200000
1400000

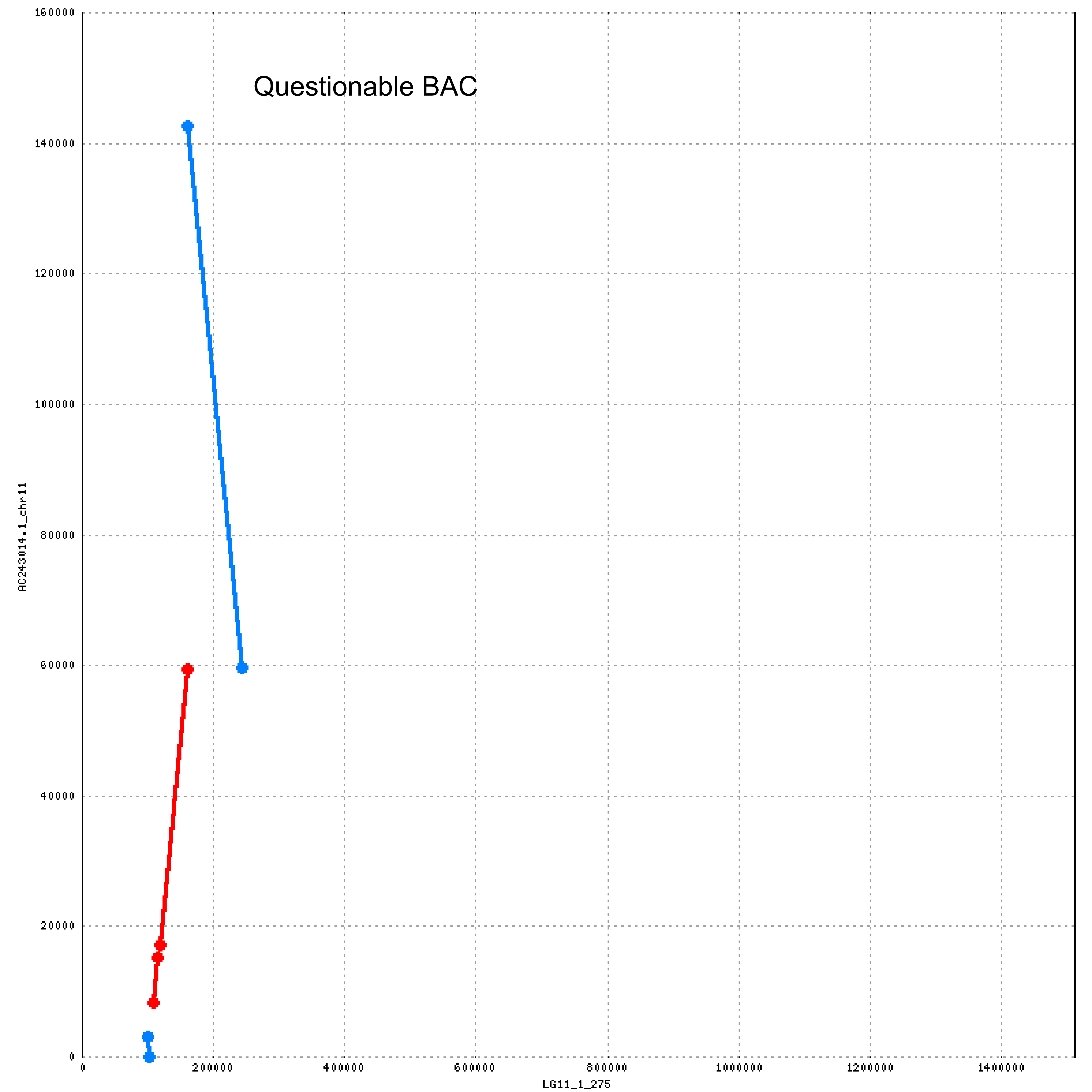
Questionable BAC
AC243014.1_chr11
LG11_1_275
0
20000
40000
60000
80000
100000
120000
140000
160000
0
200000
400000
600000
800000
1000000
1200000
1400000

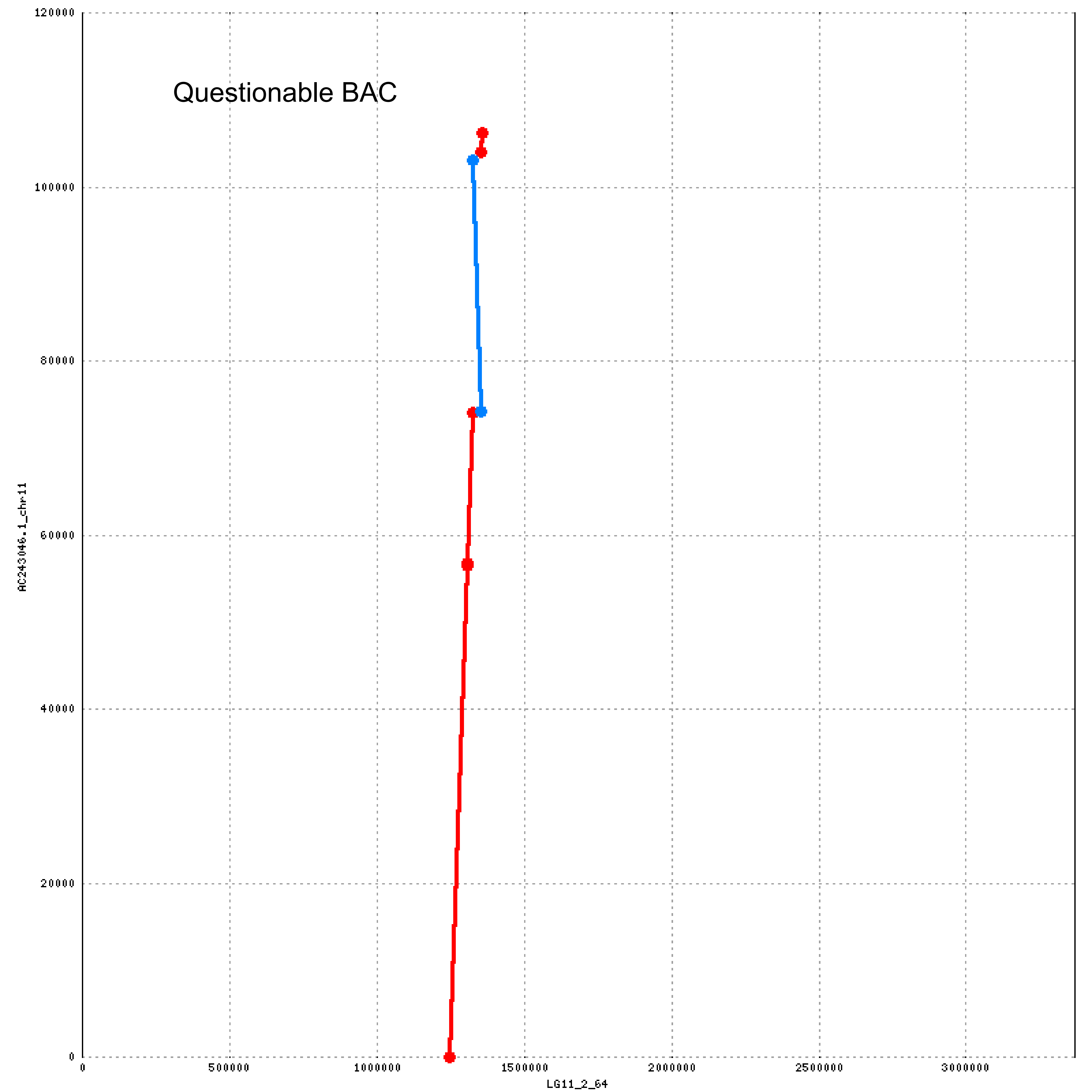
Questionable BAC
120000
100000
80000
60000
40000
20000
0
AC243046.1_chr11
0
500000
1000000
1500000
2000000
2500000
3000000
LG11_2_64

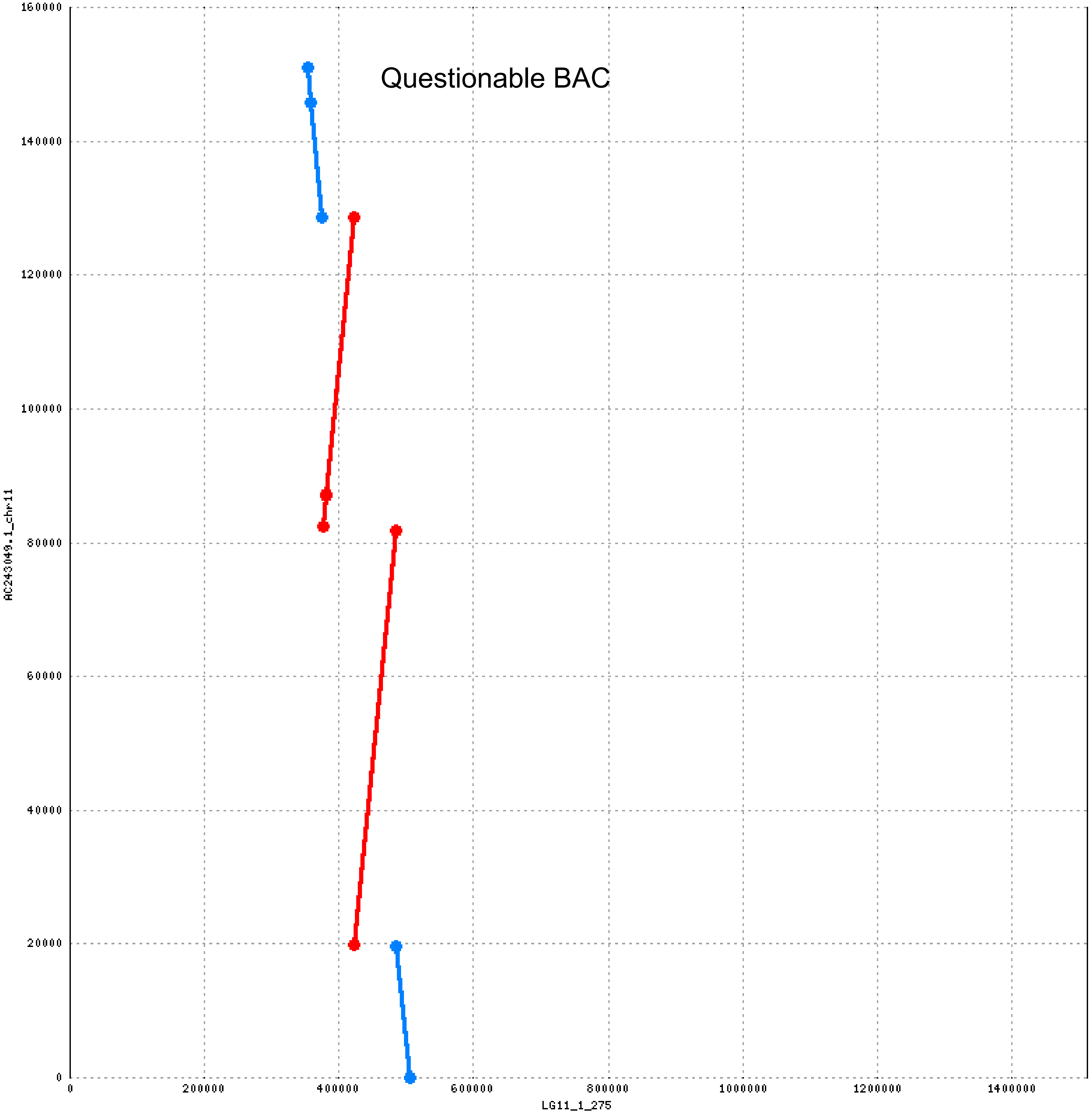
Questionable BAC
AC243049.1_chr11
LG11_1_275
0
20000
40000
60000
80000
100000
120000
140000
160000
200000
400000
600000
800000
1000000
1200000
1400000
