# Supplementary figures and images for: Haplotype-resolved genome analyses of a heterozygous diploid potato
Source: Nat Genet. 2020 Sep 28;52(10):1018–23. doi: 10.1038/s41588-020-0699-x (PMC7527274; doi:10.1038/s41588-020-0699-x)

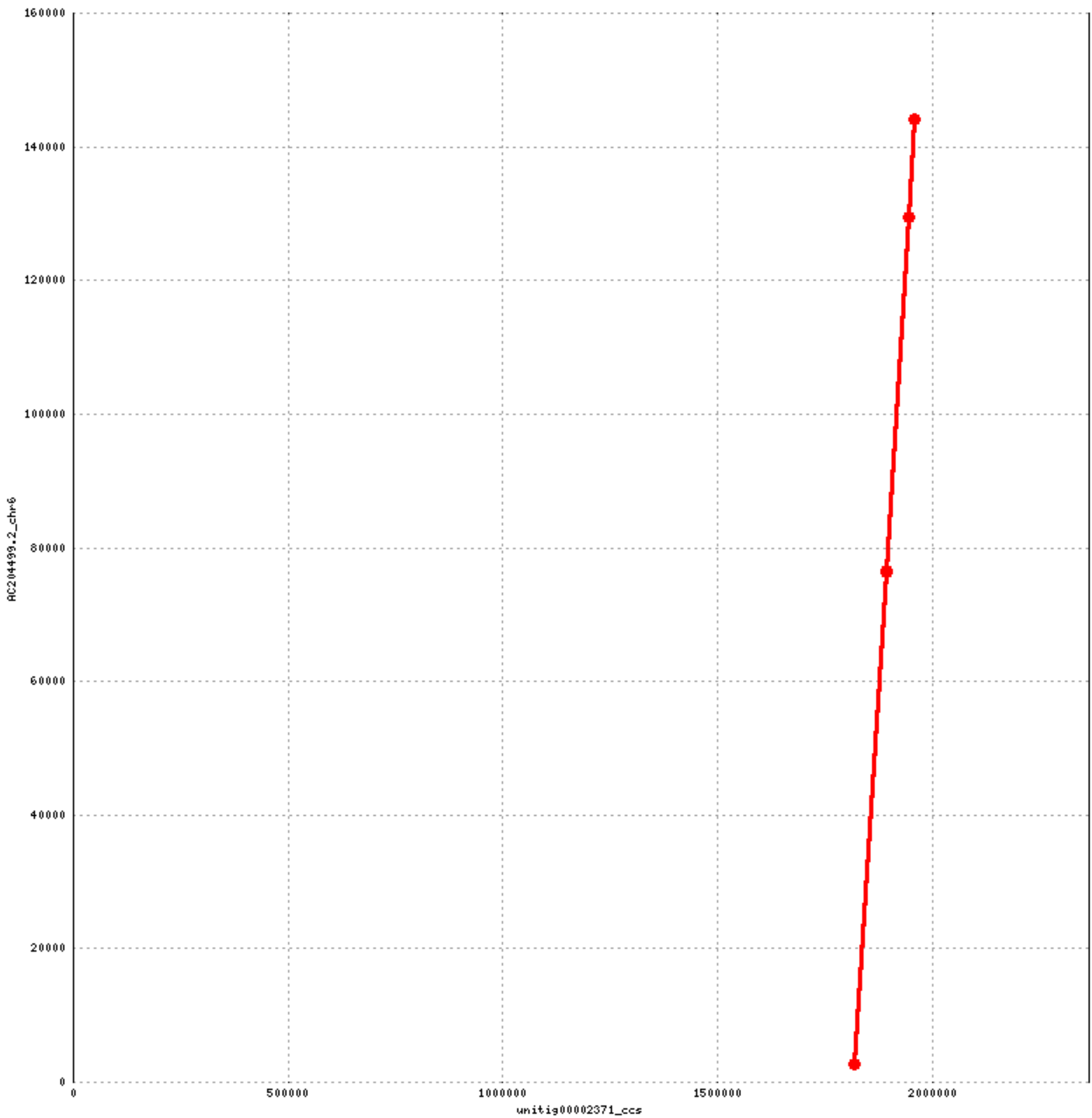

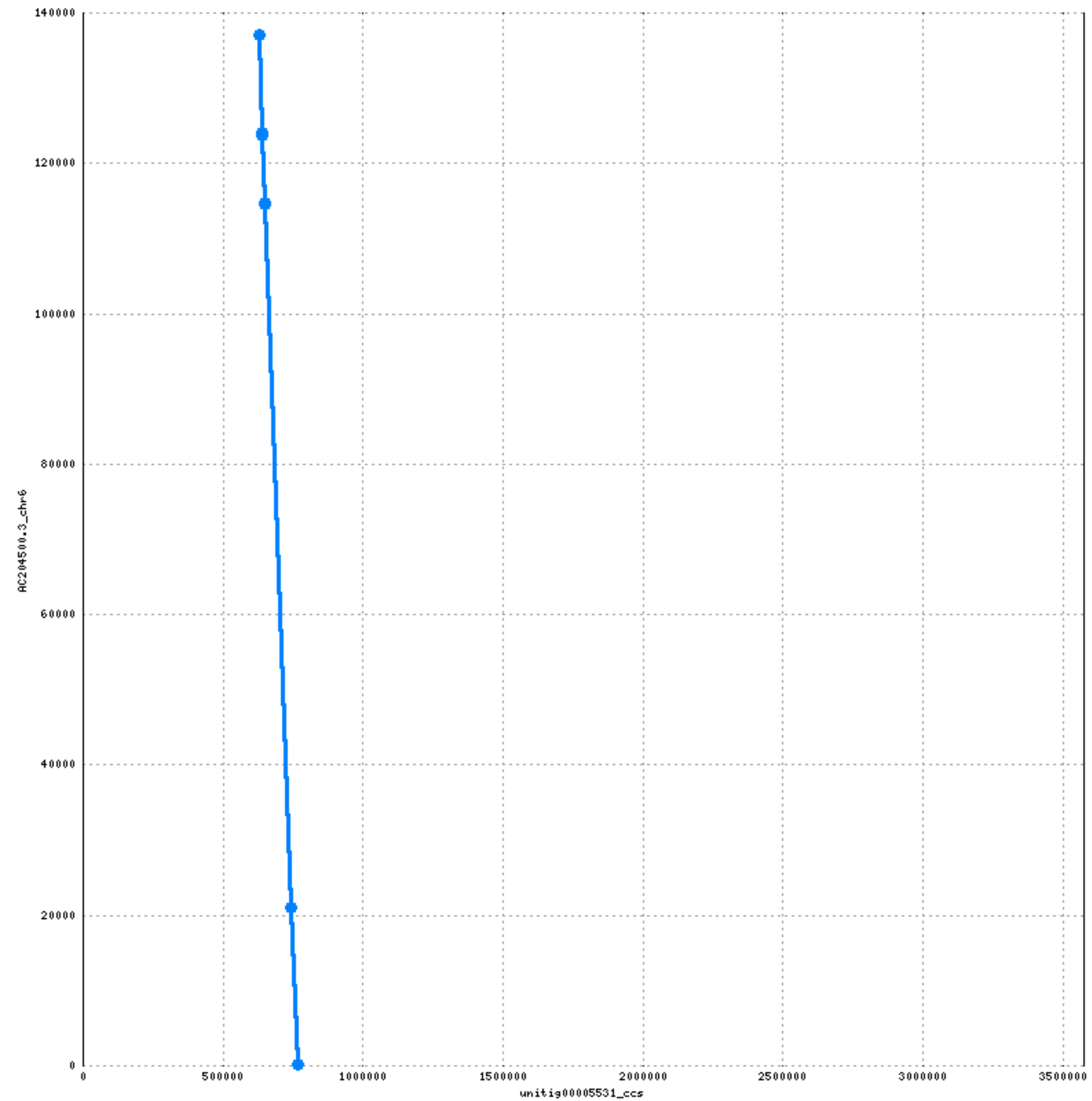

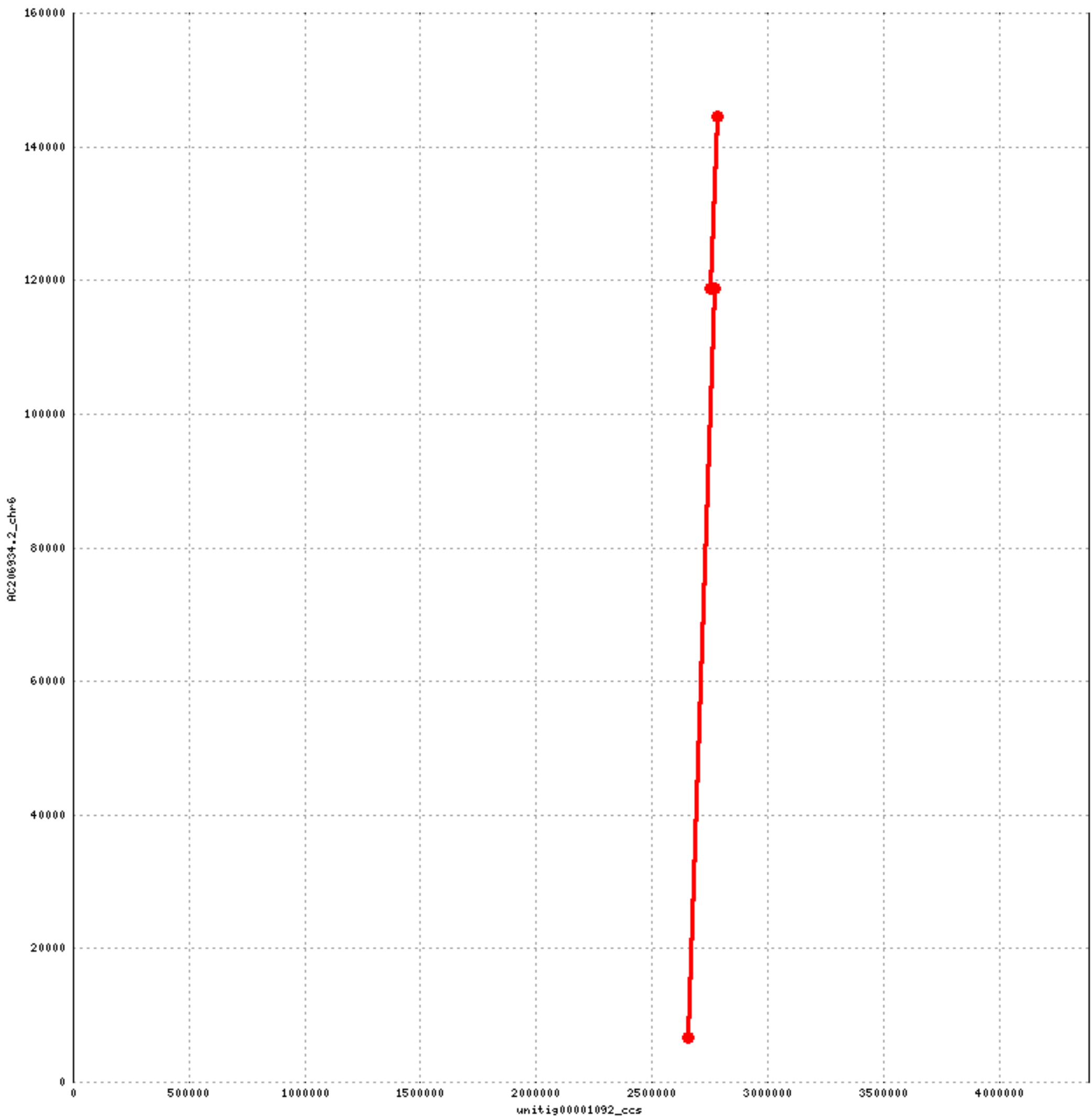

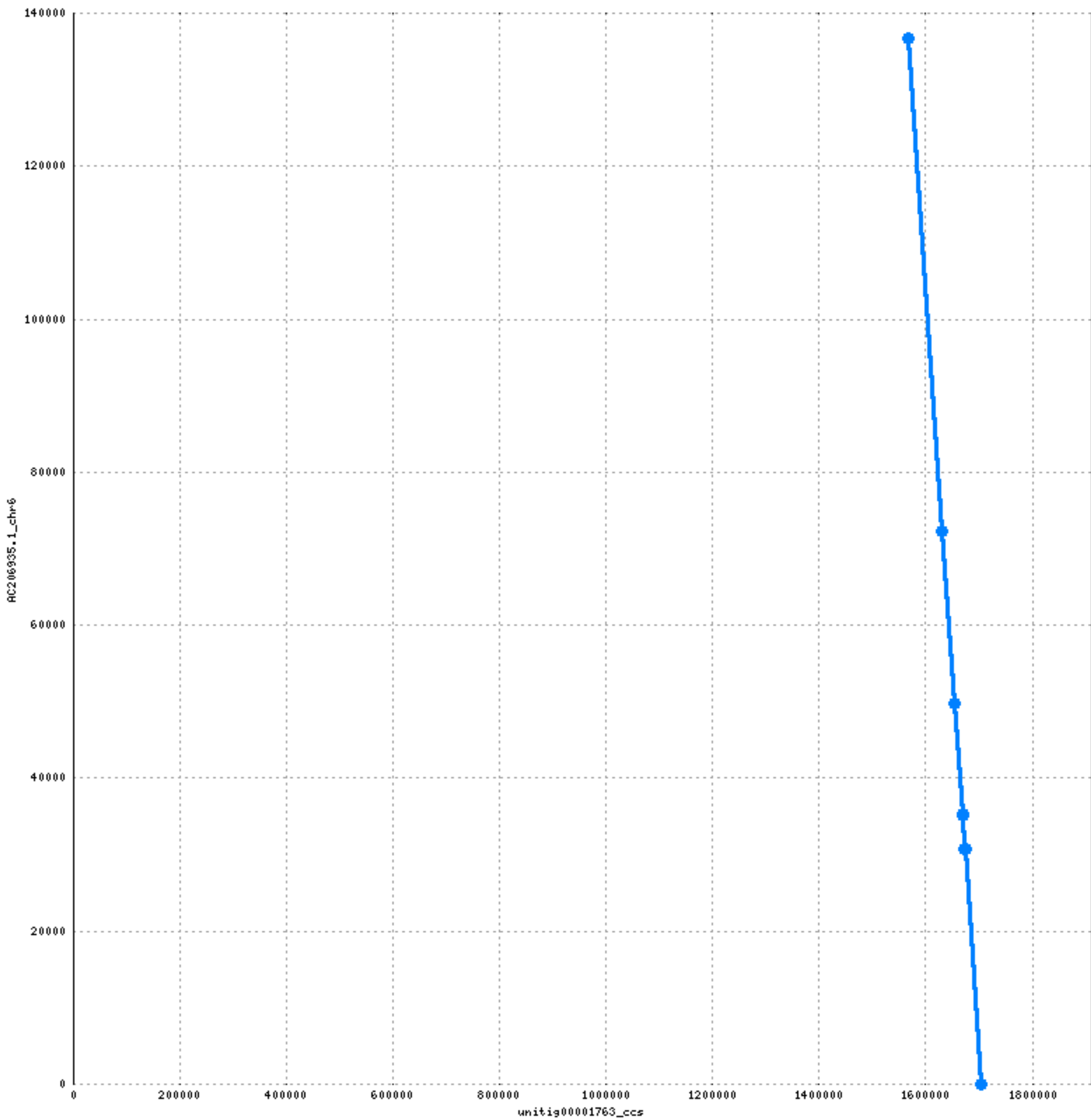

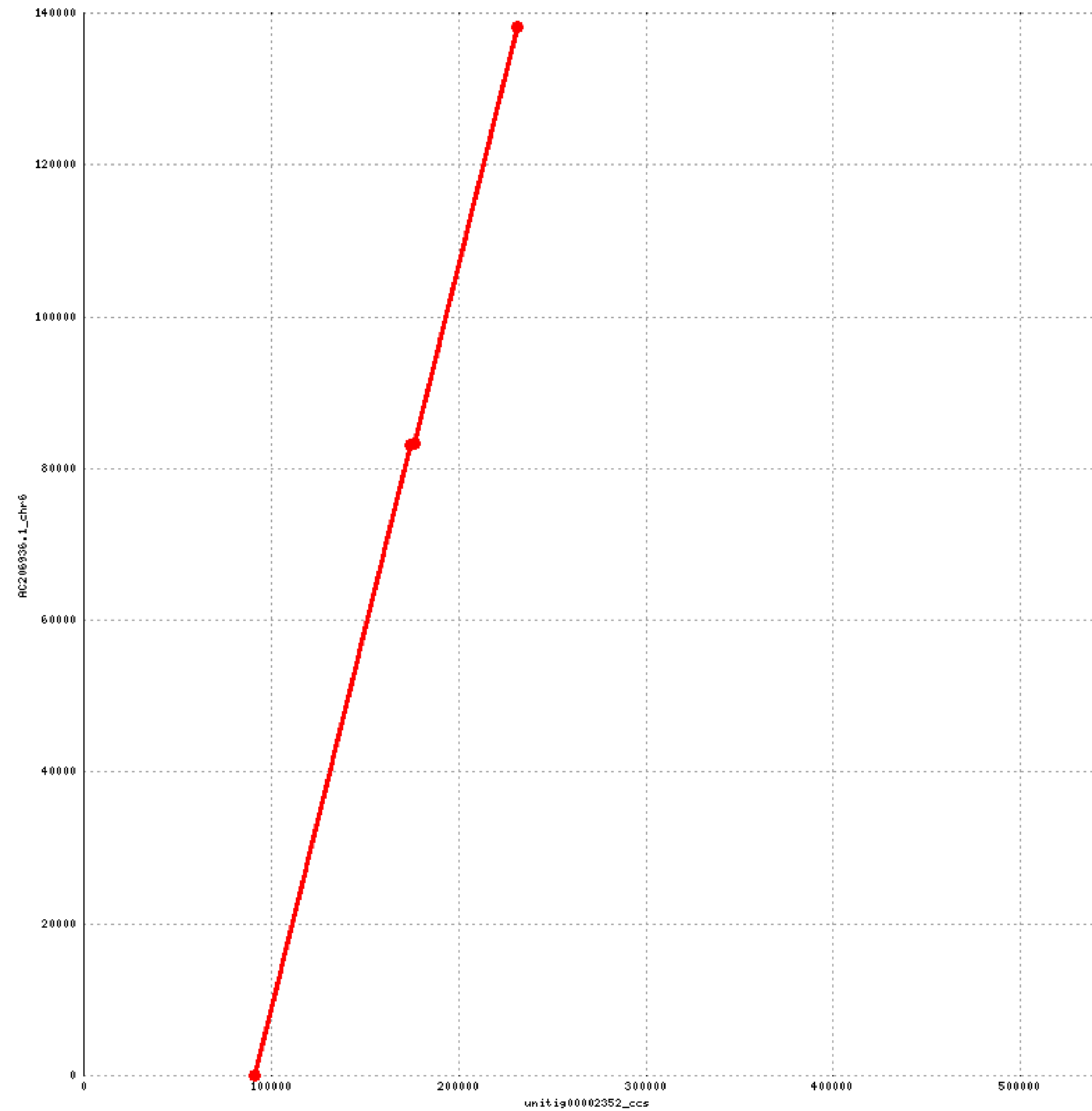

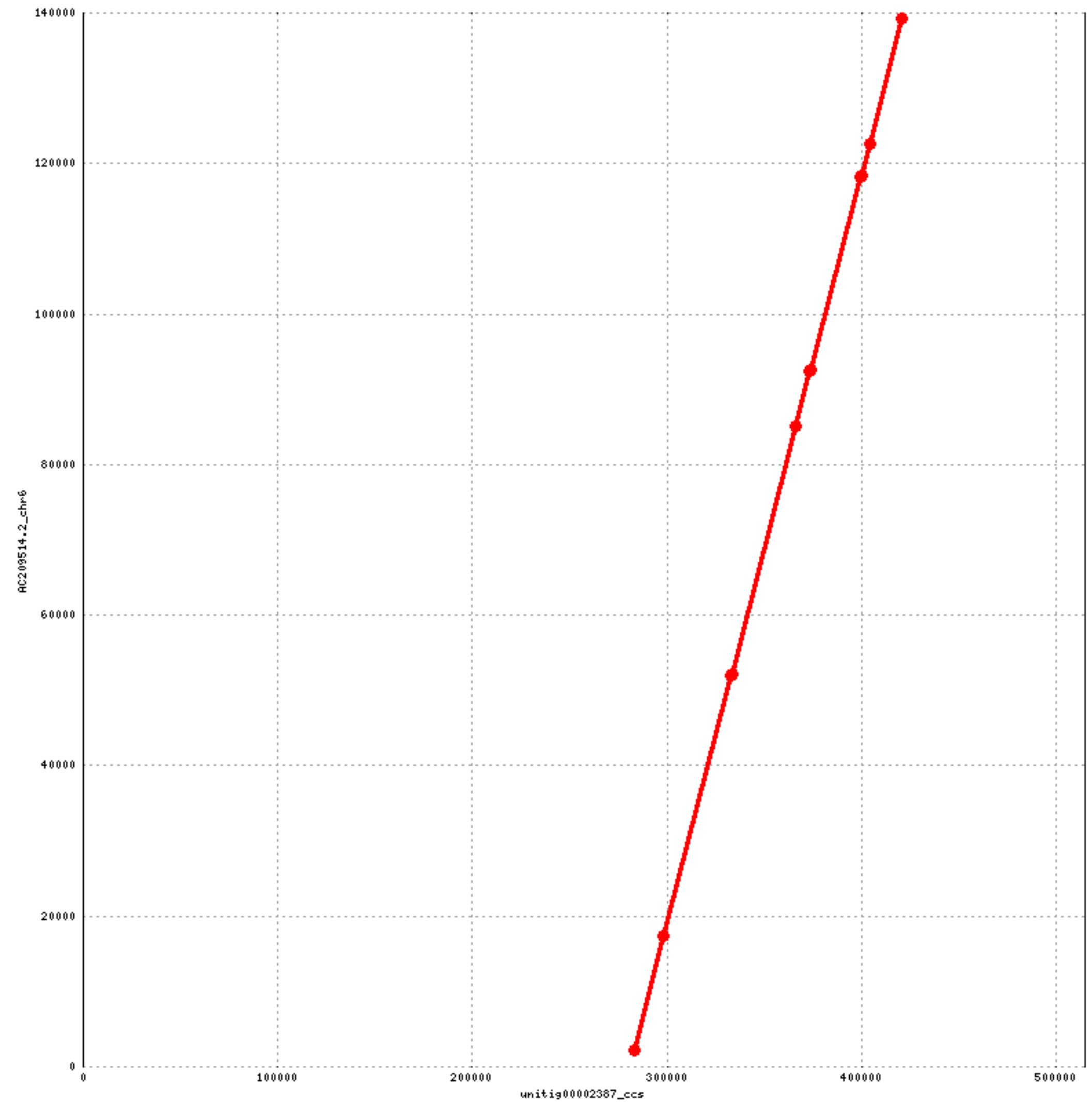

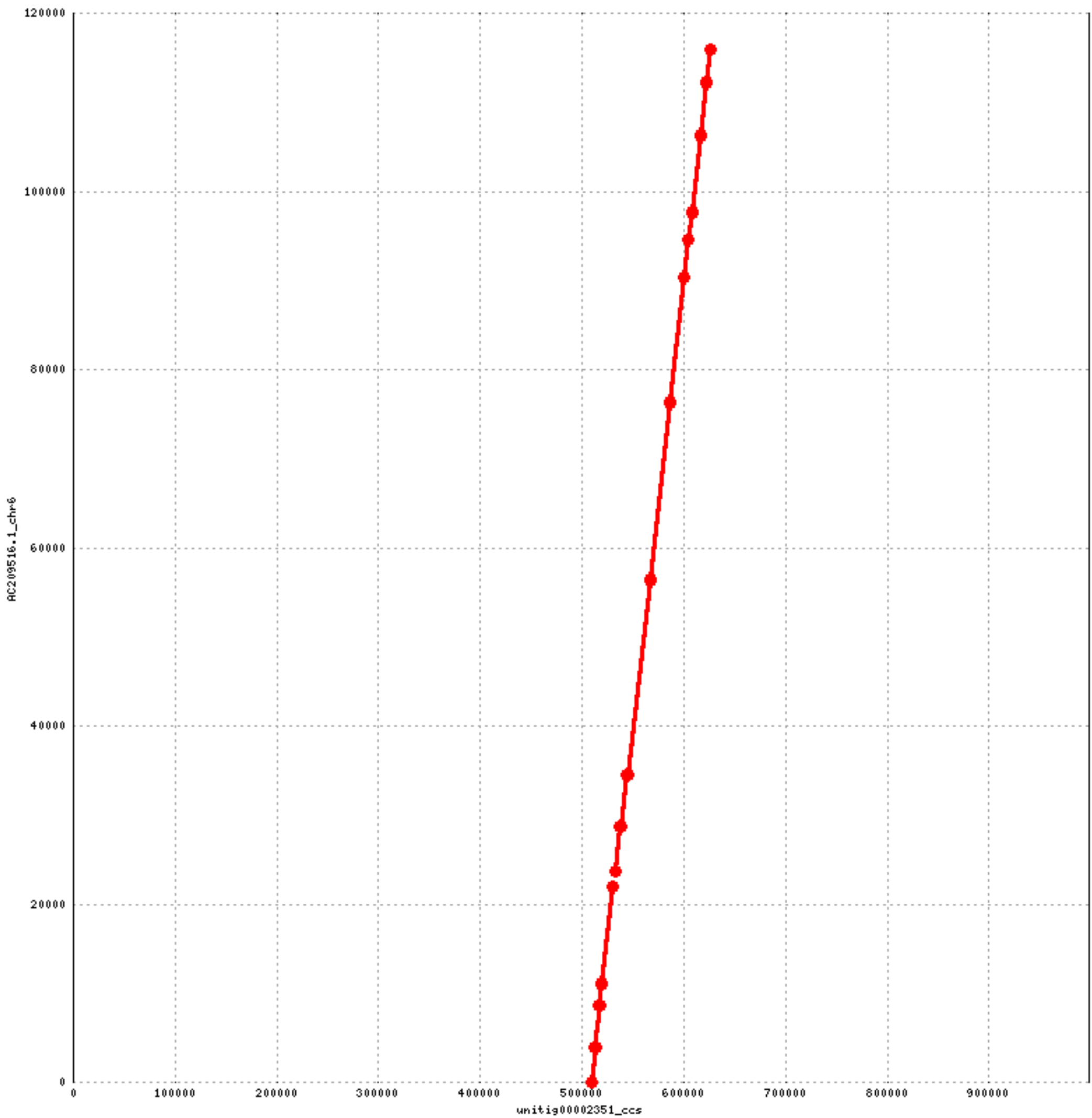

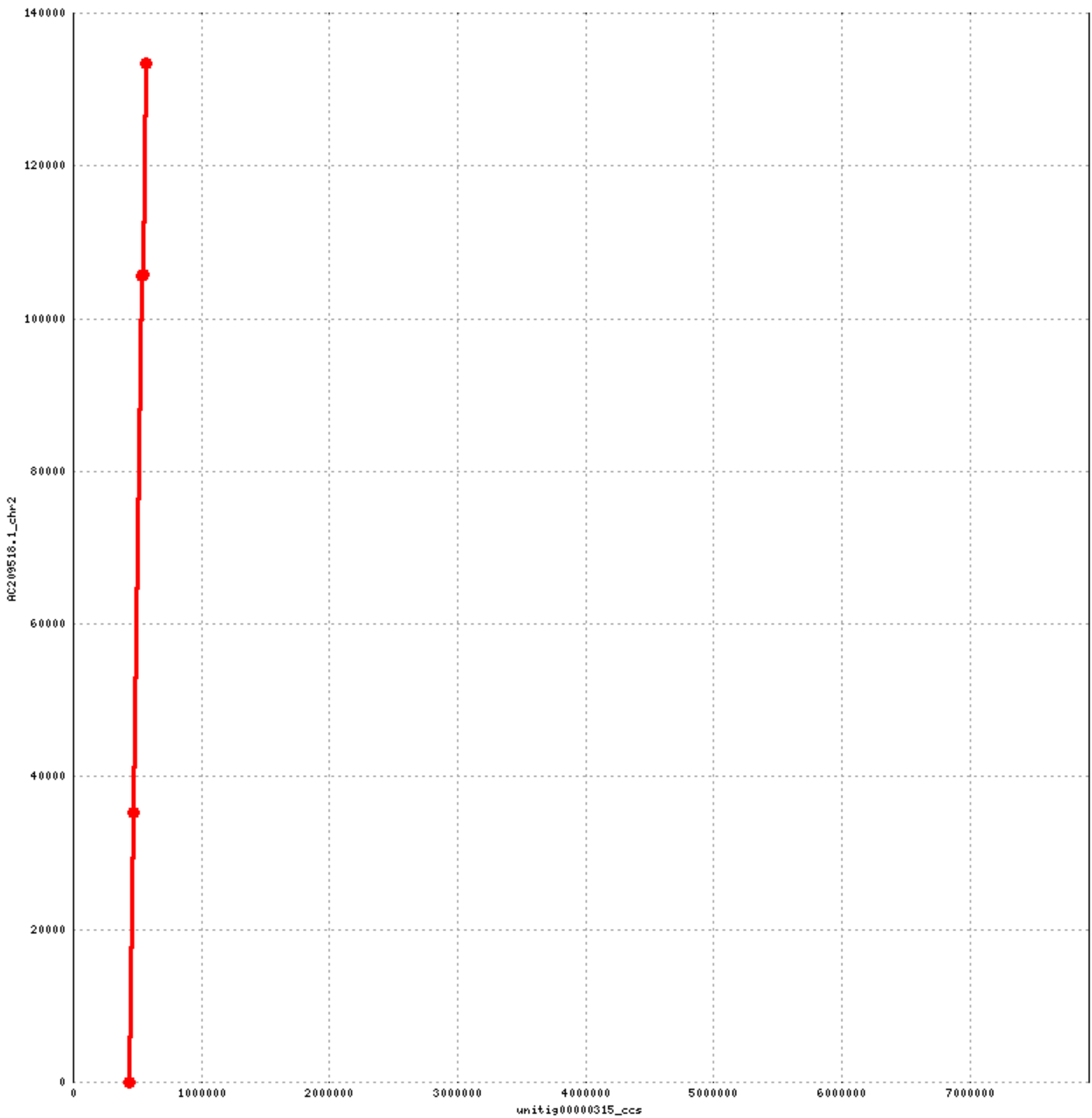

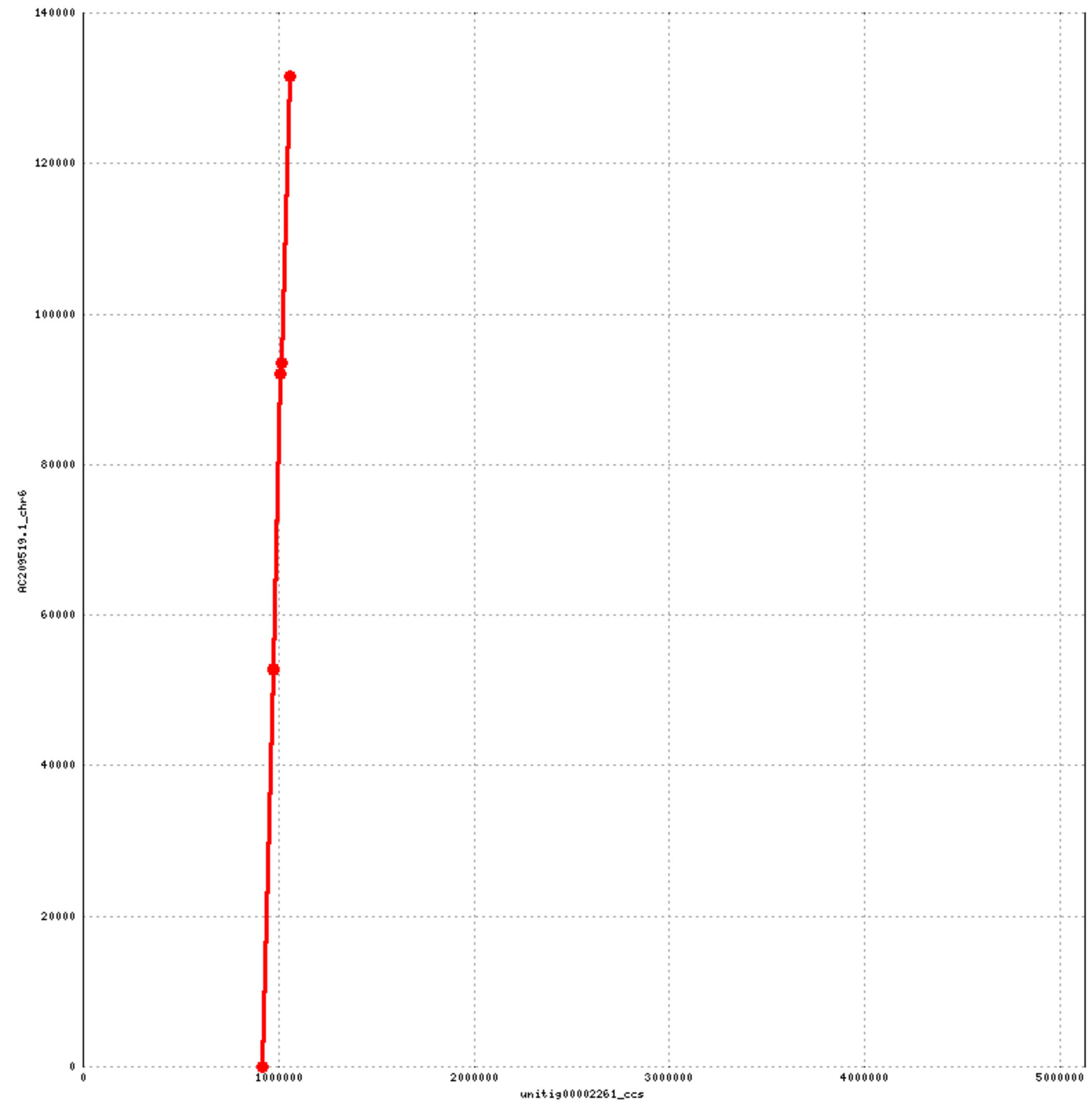

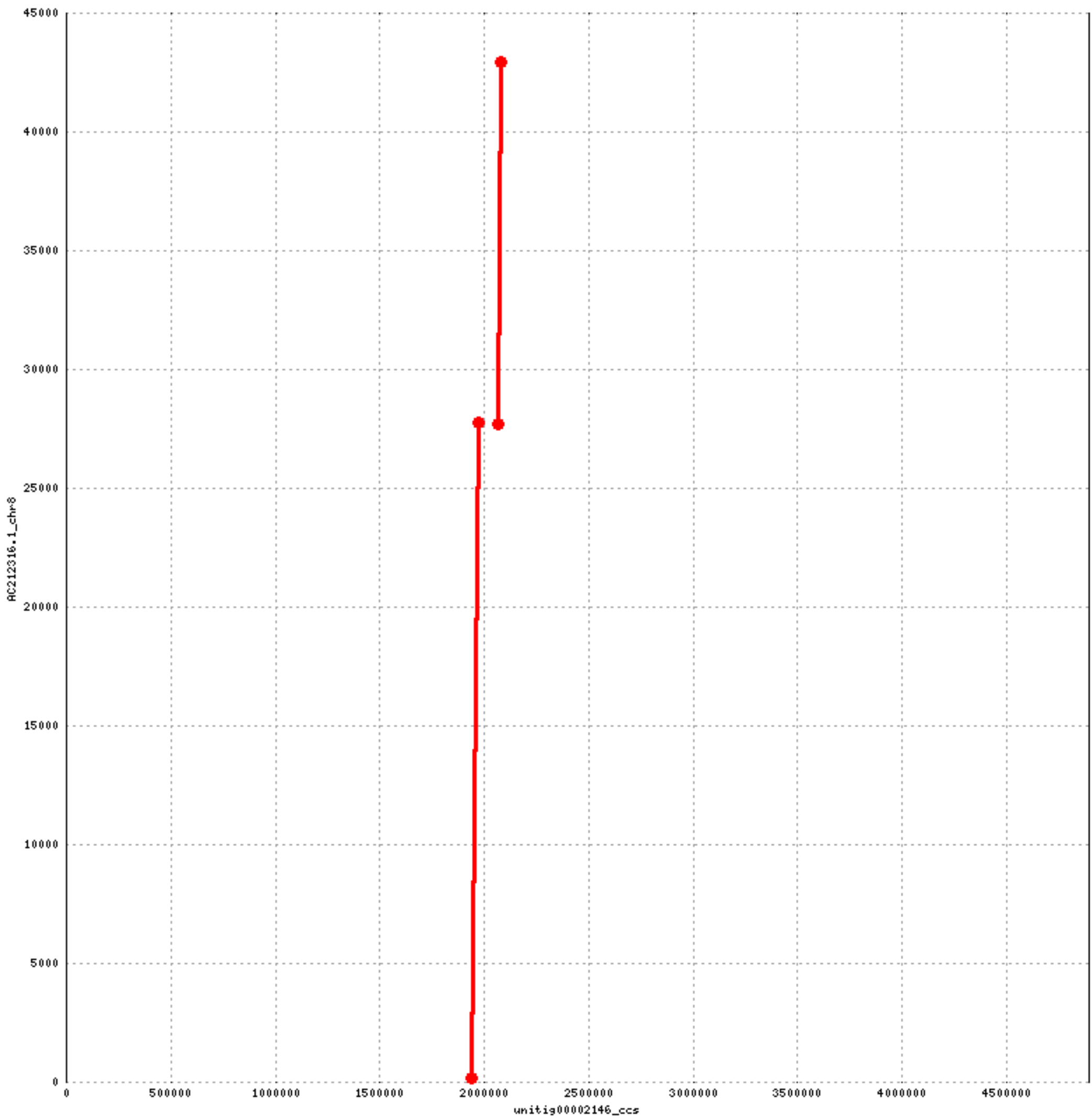

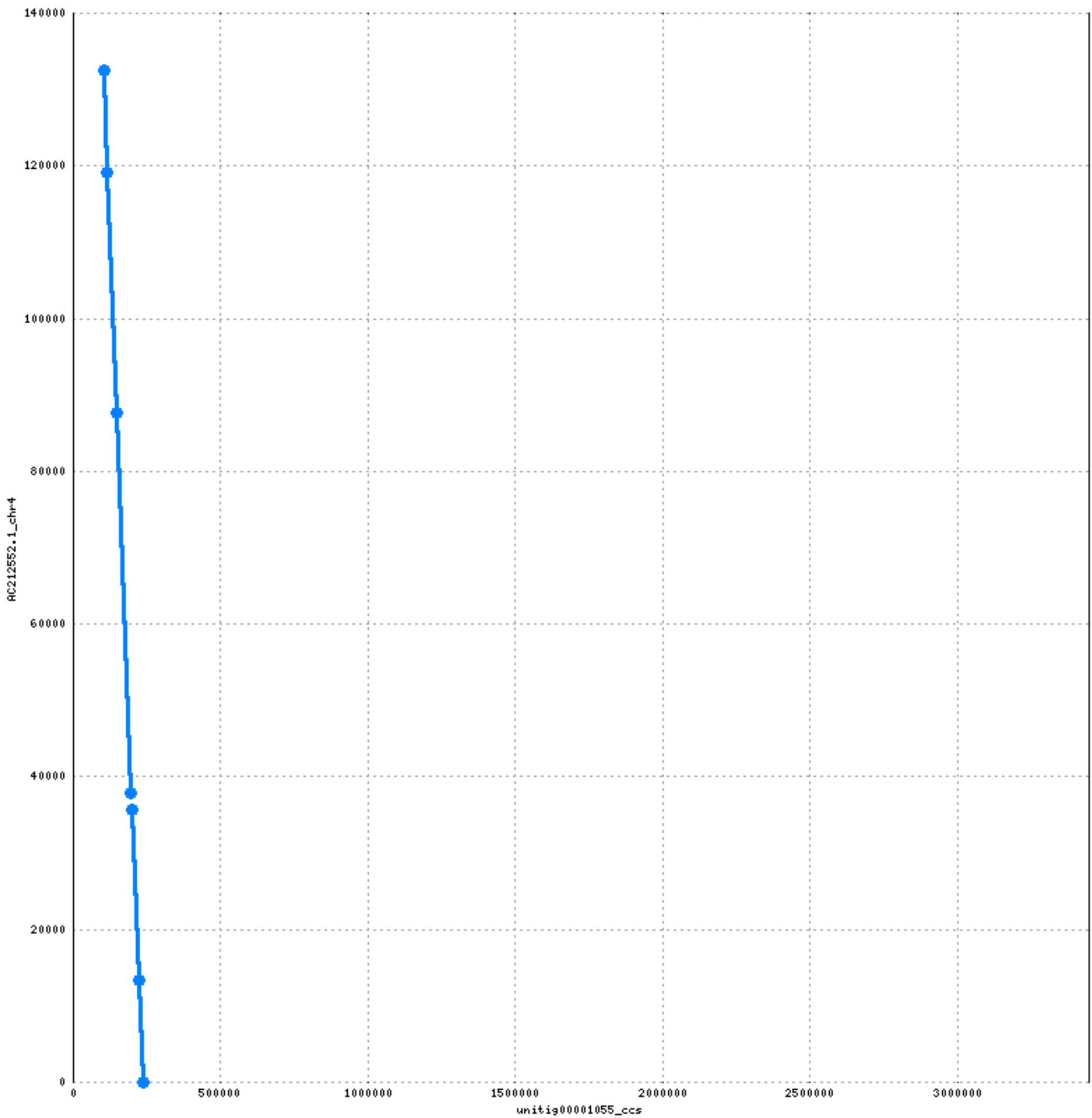

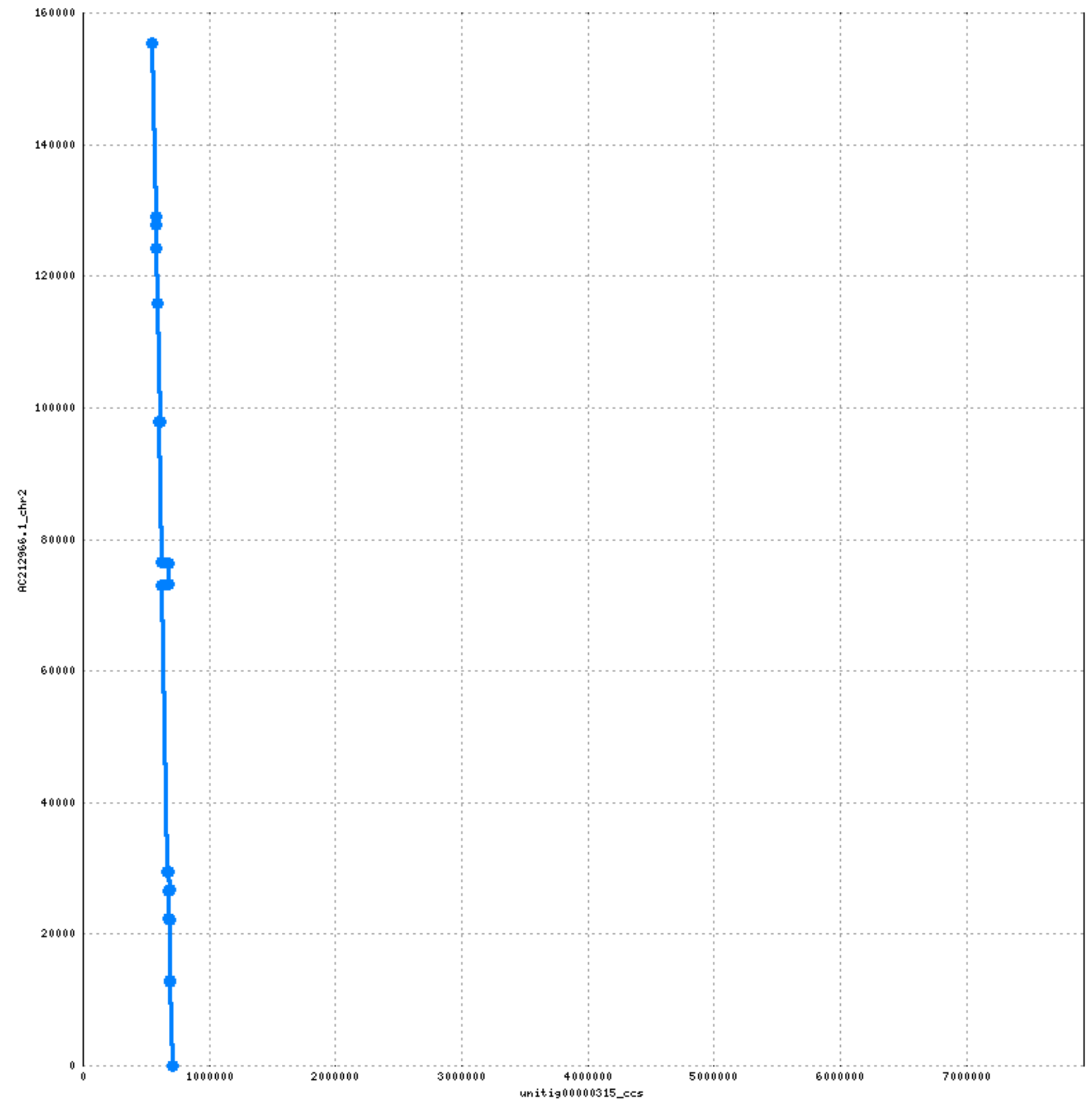

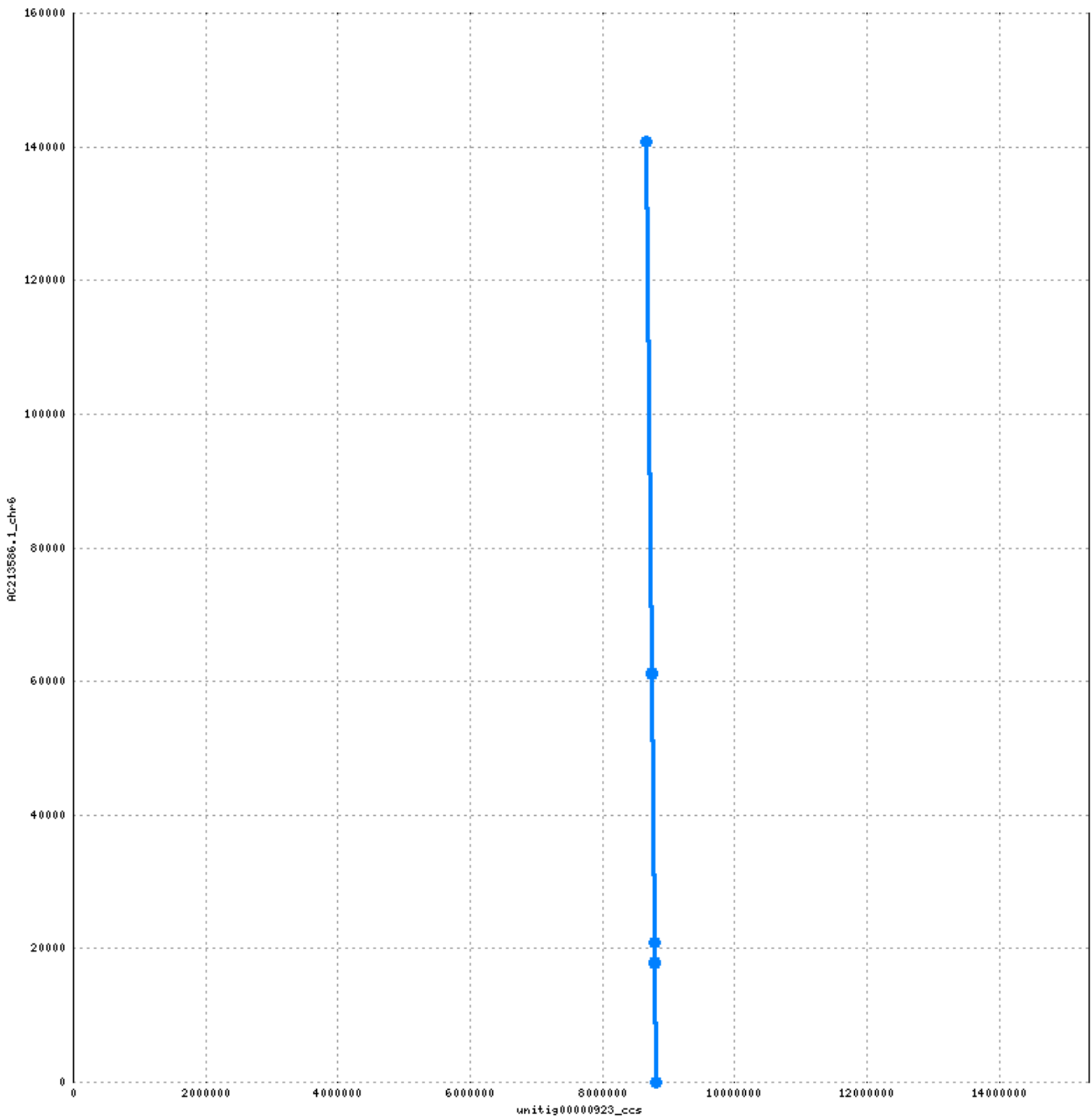

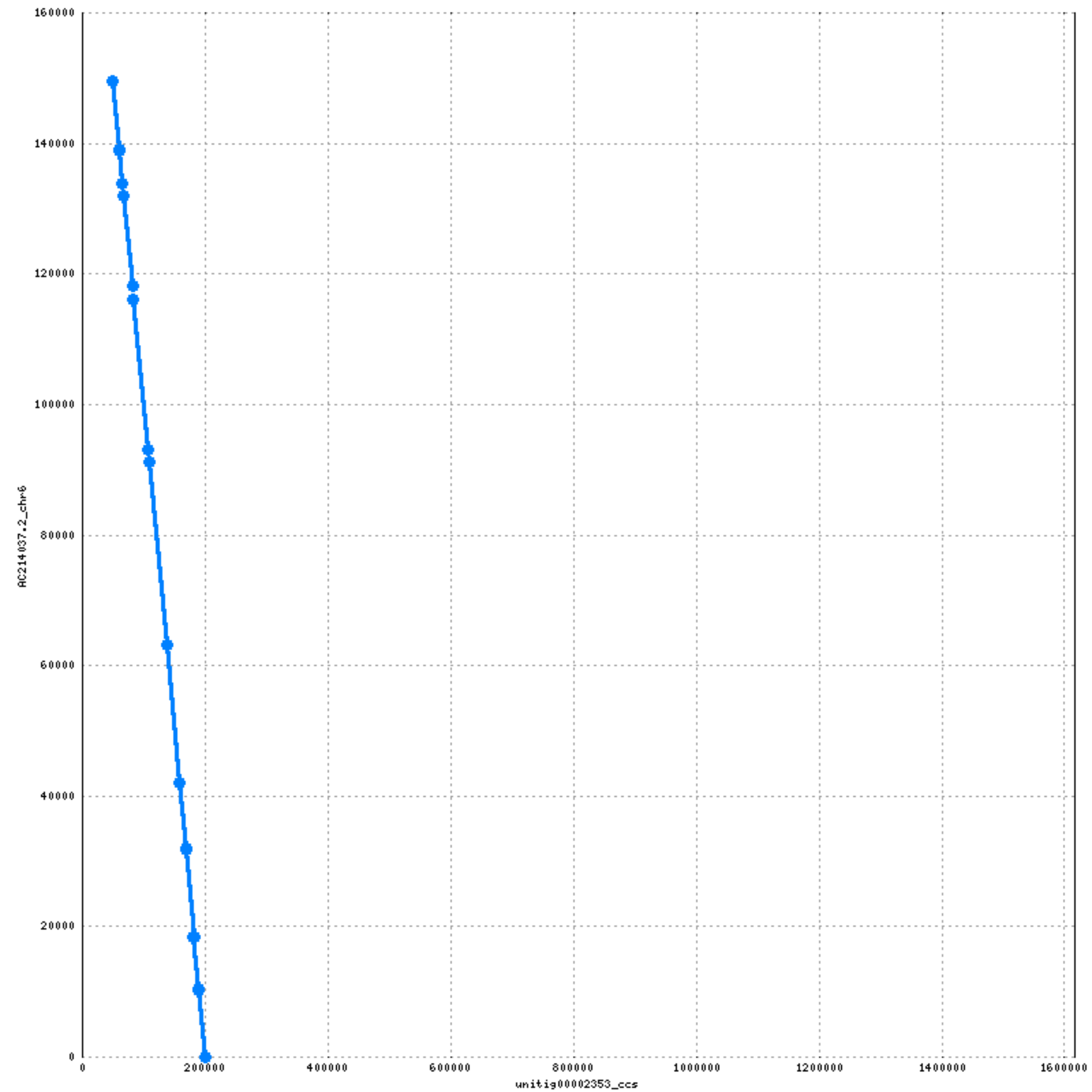

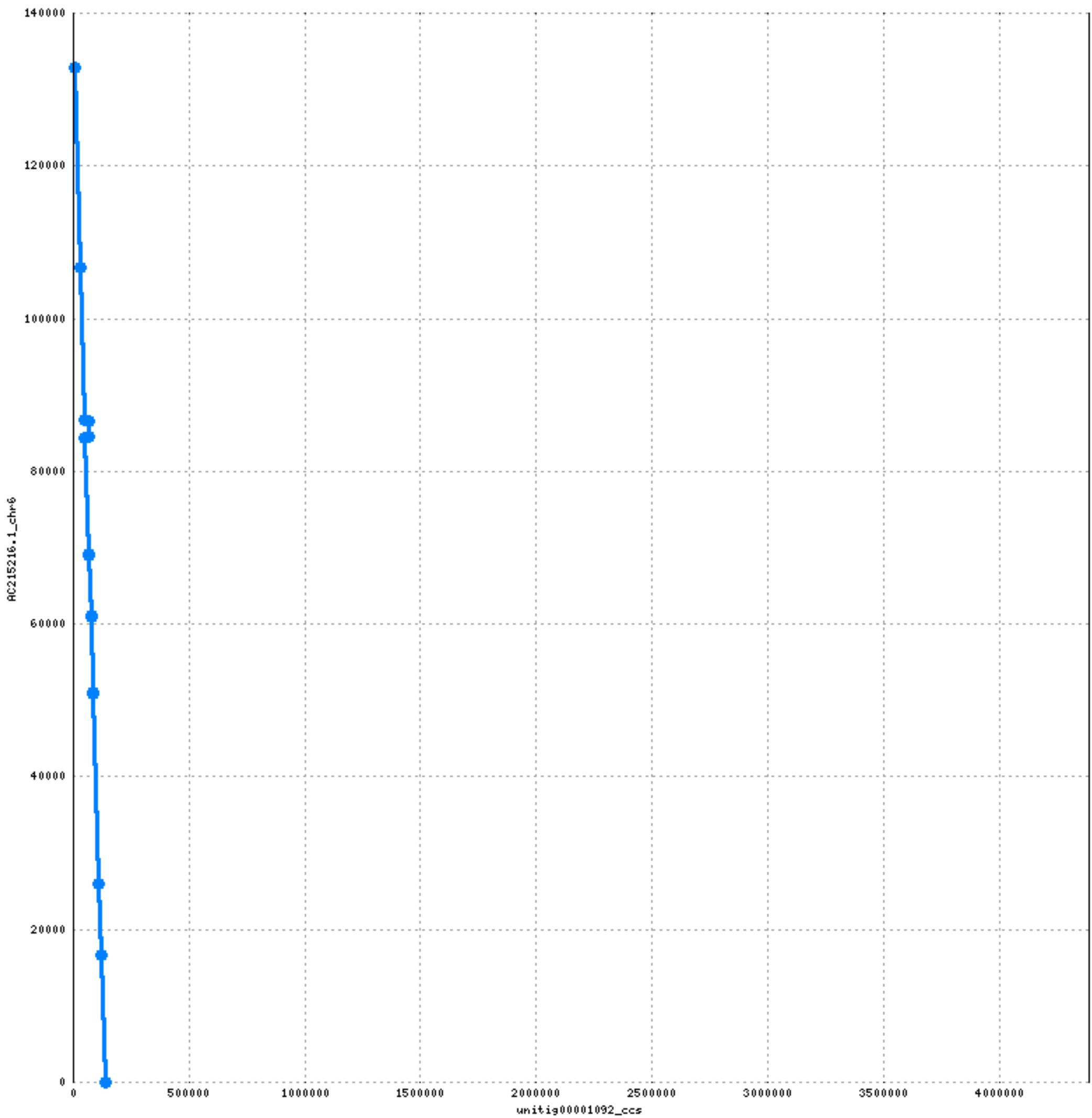

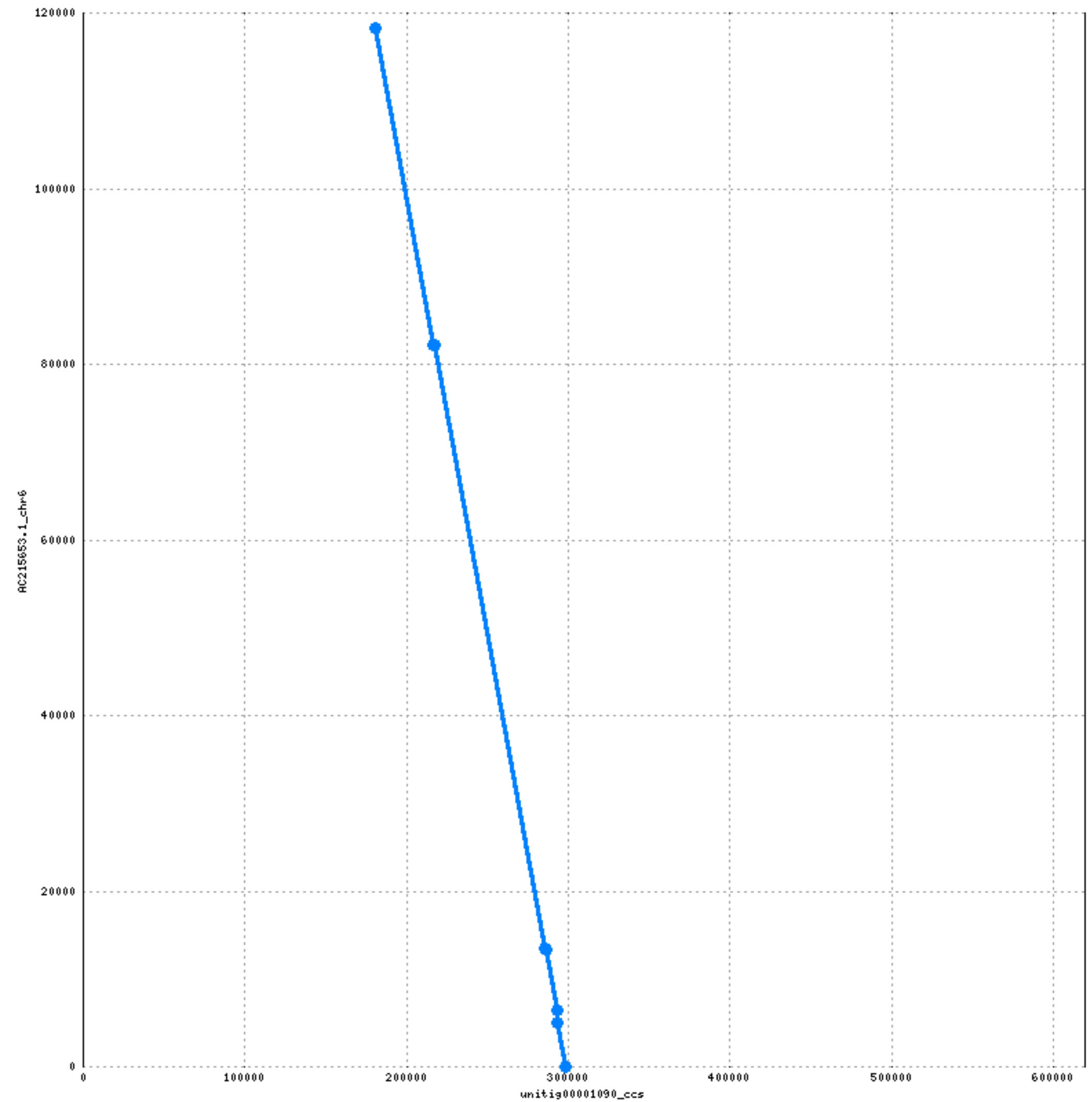

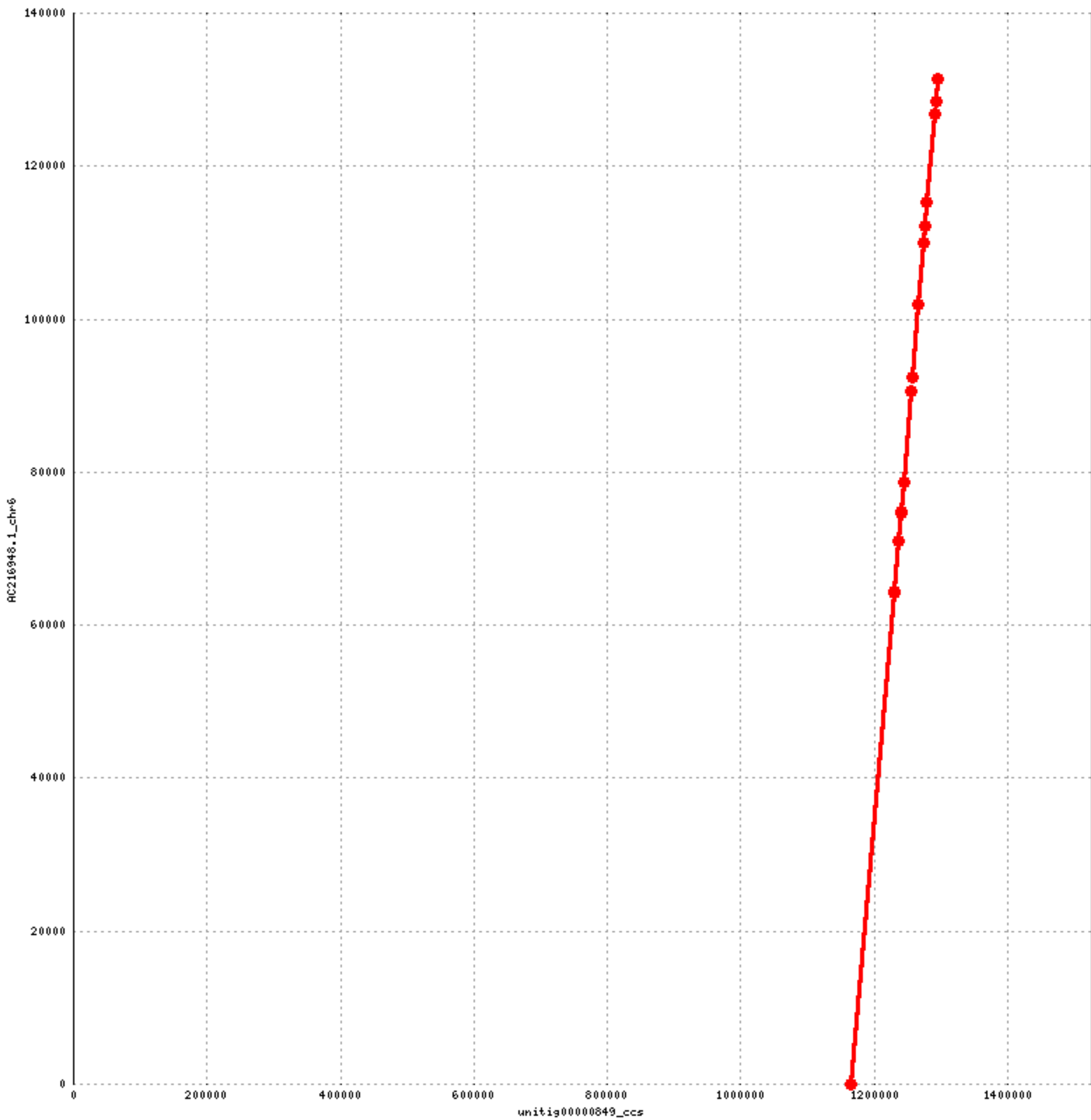

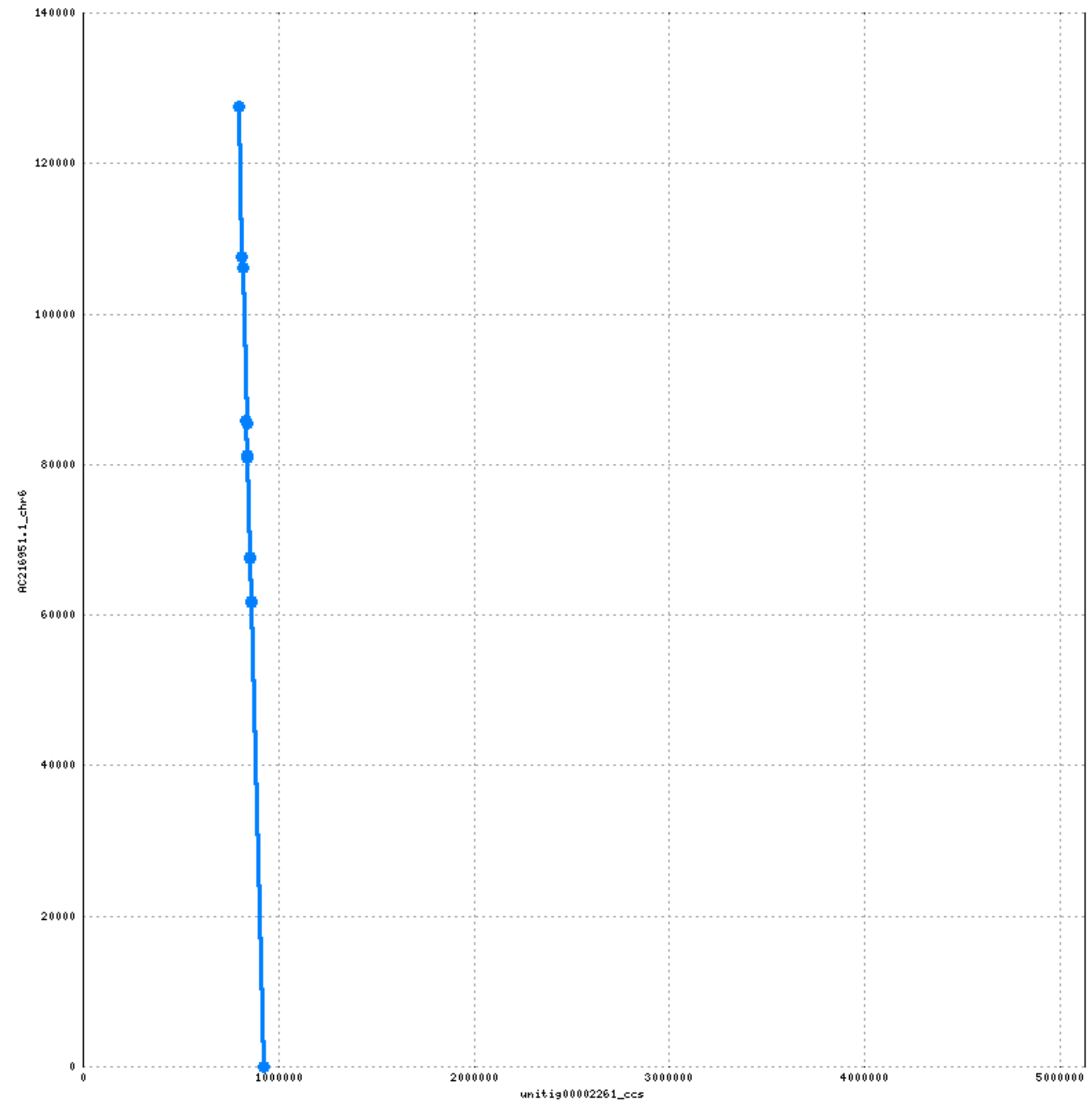

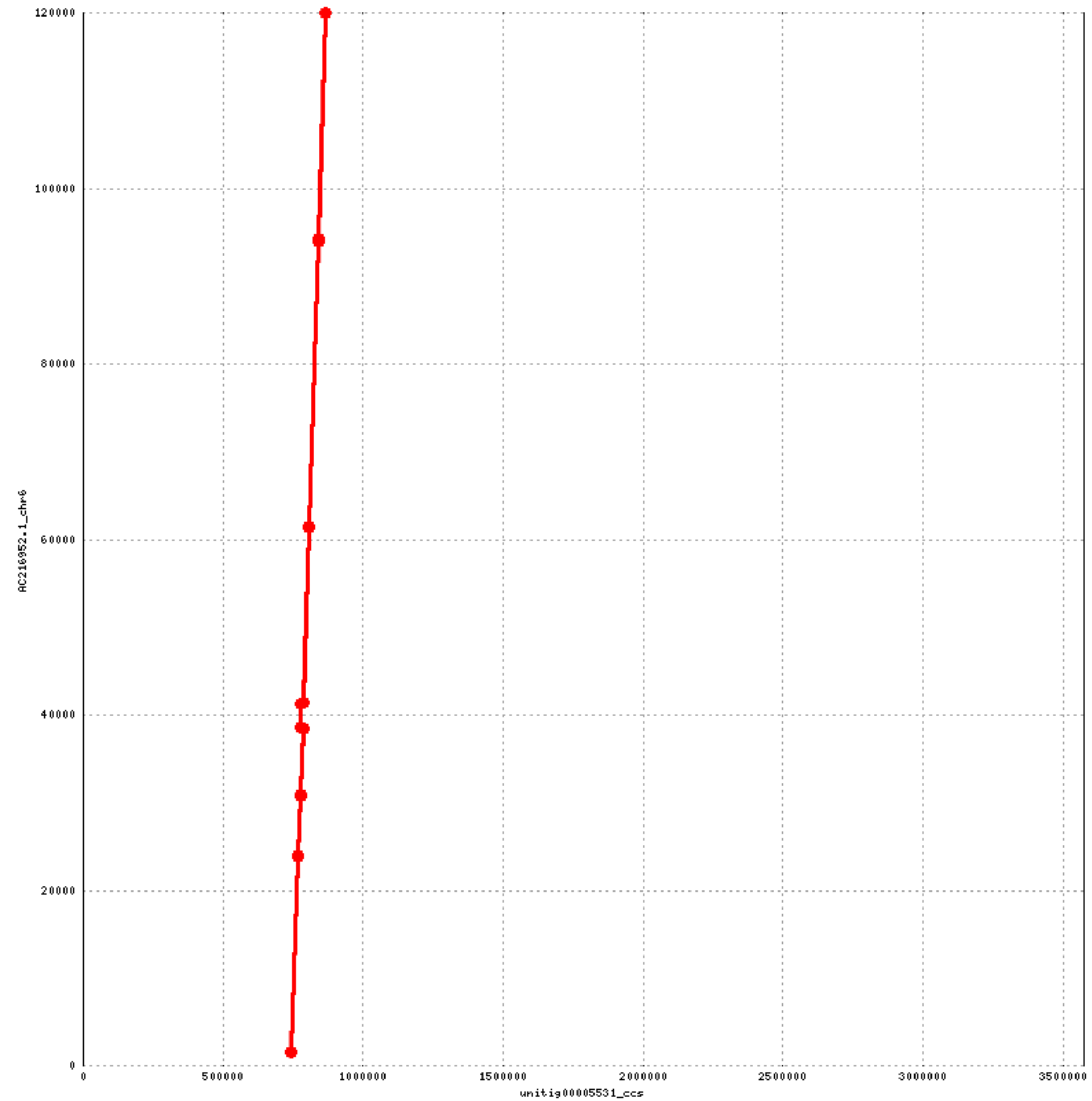

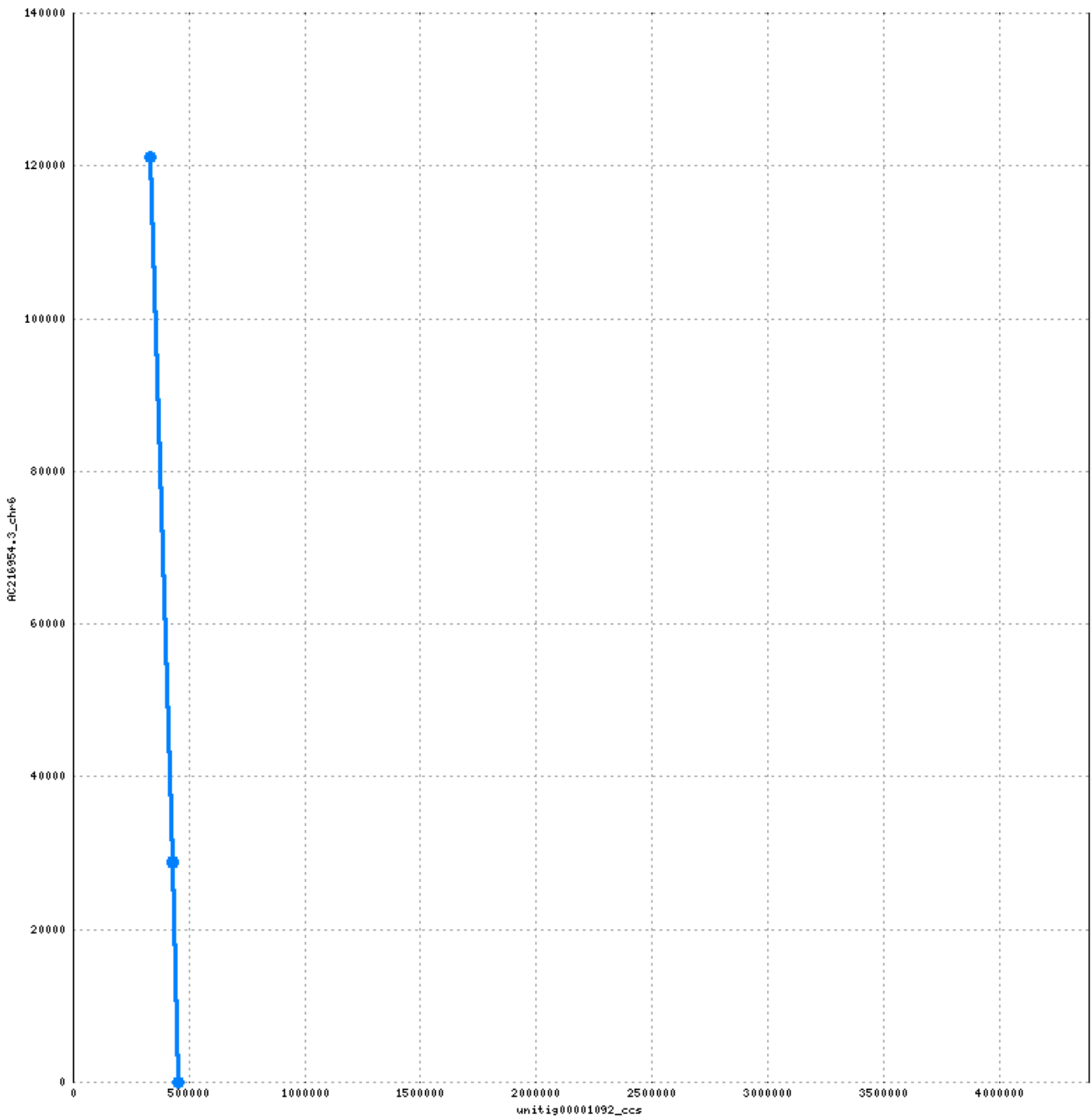

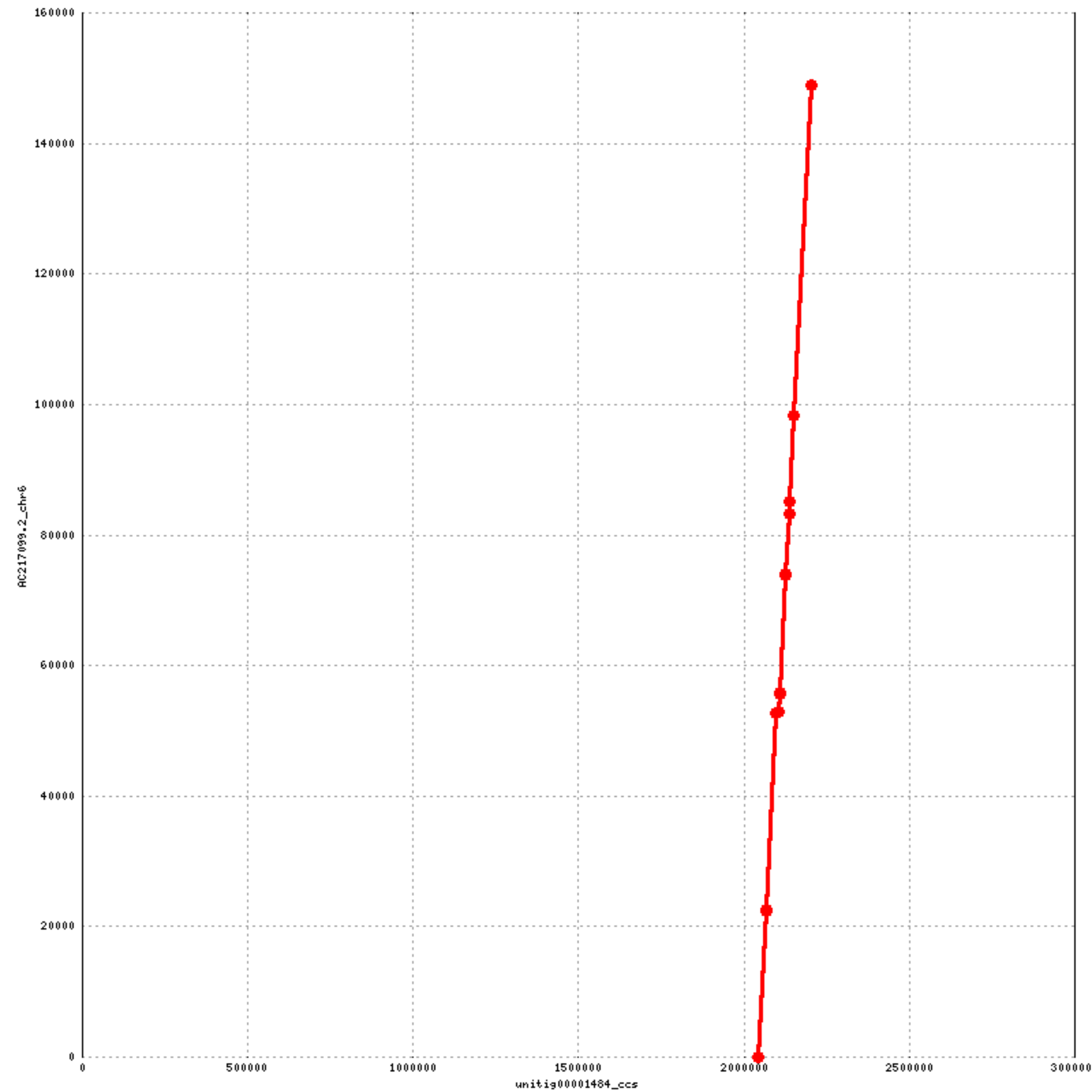

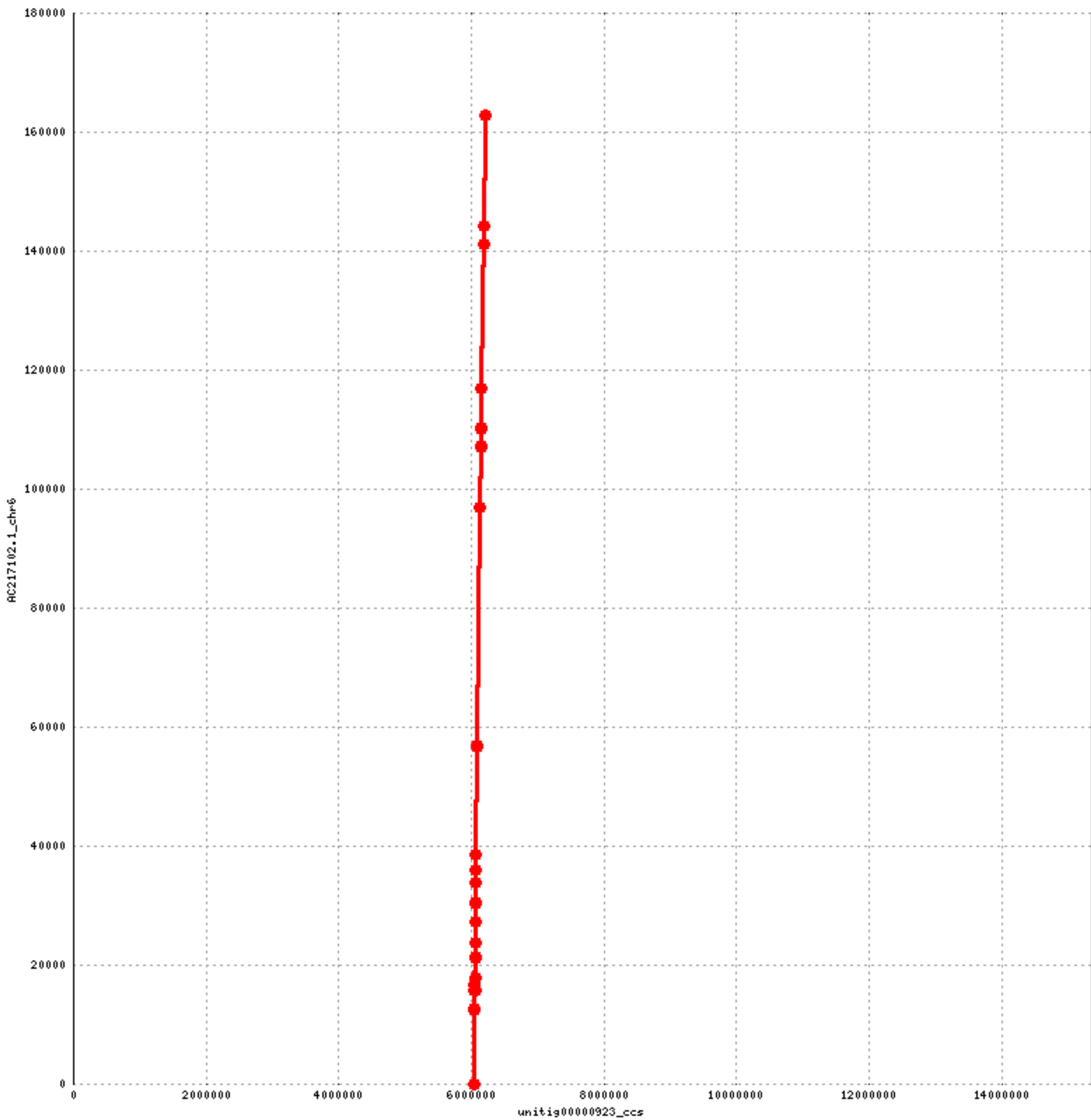

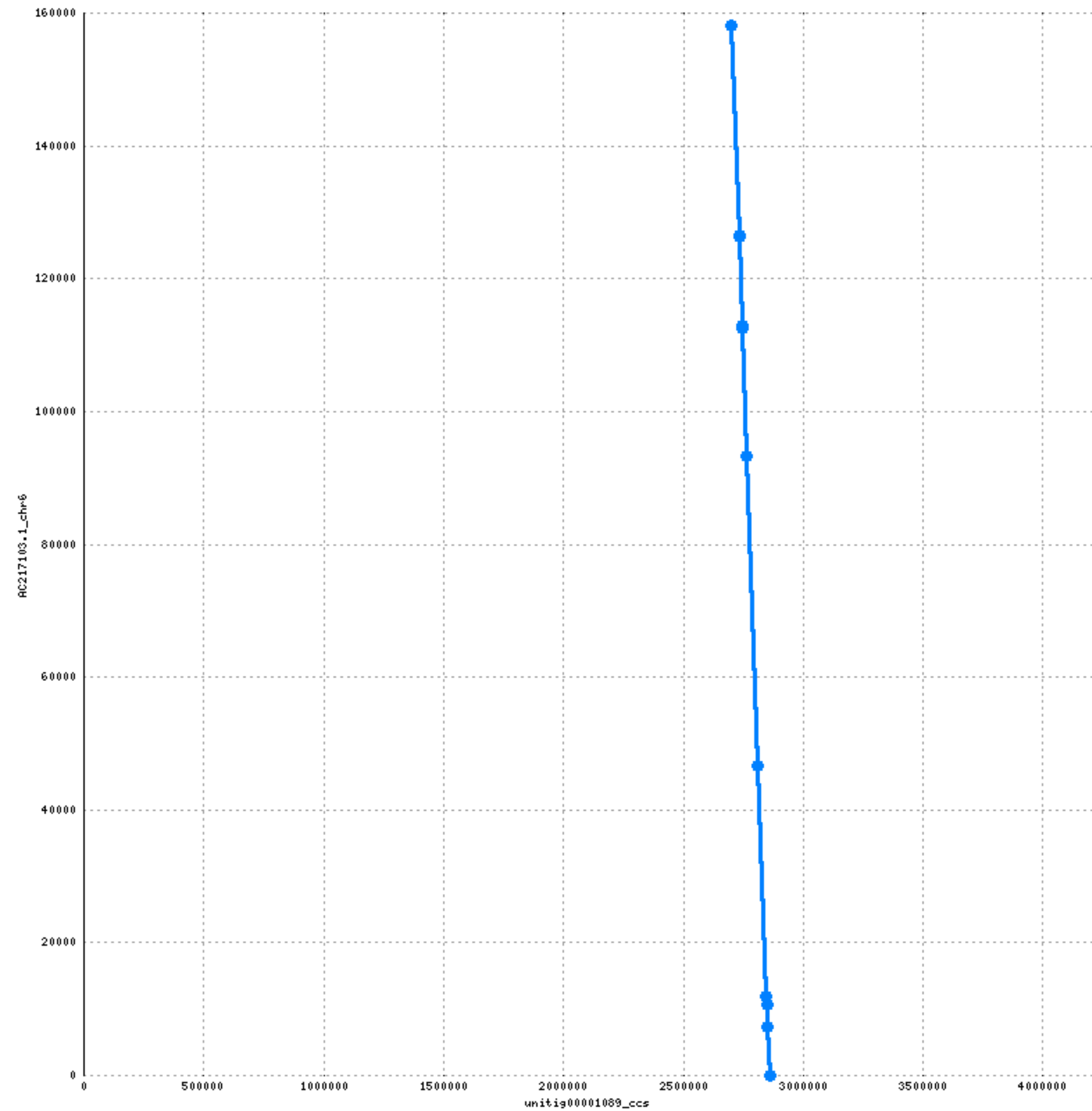

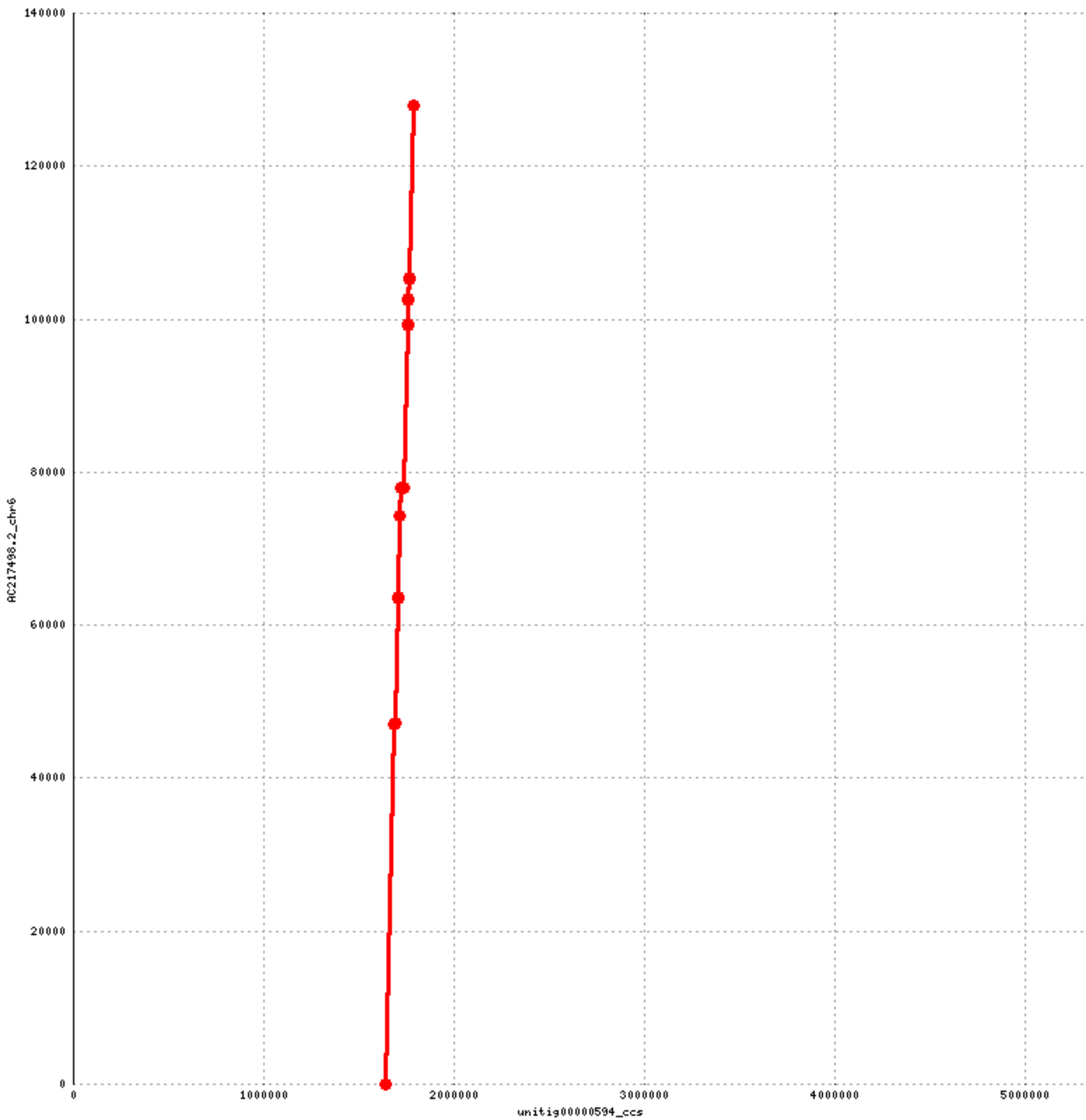

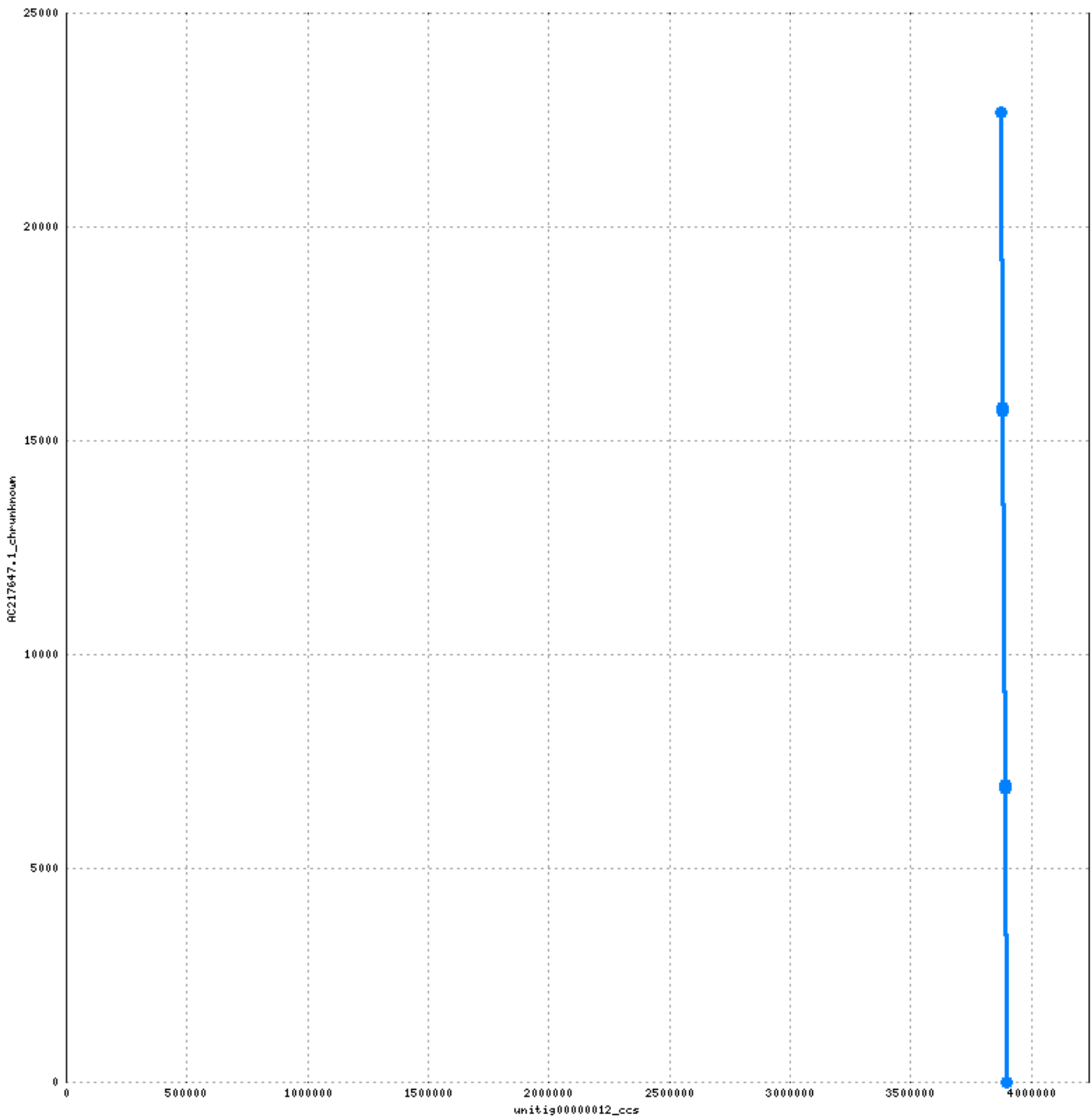

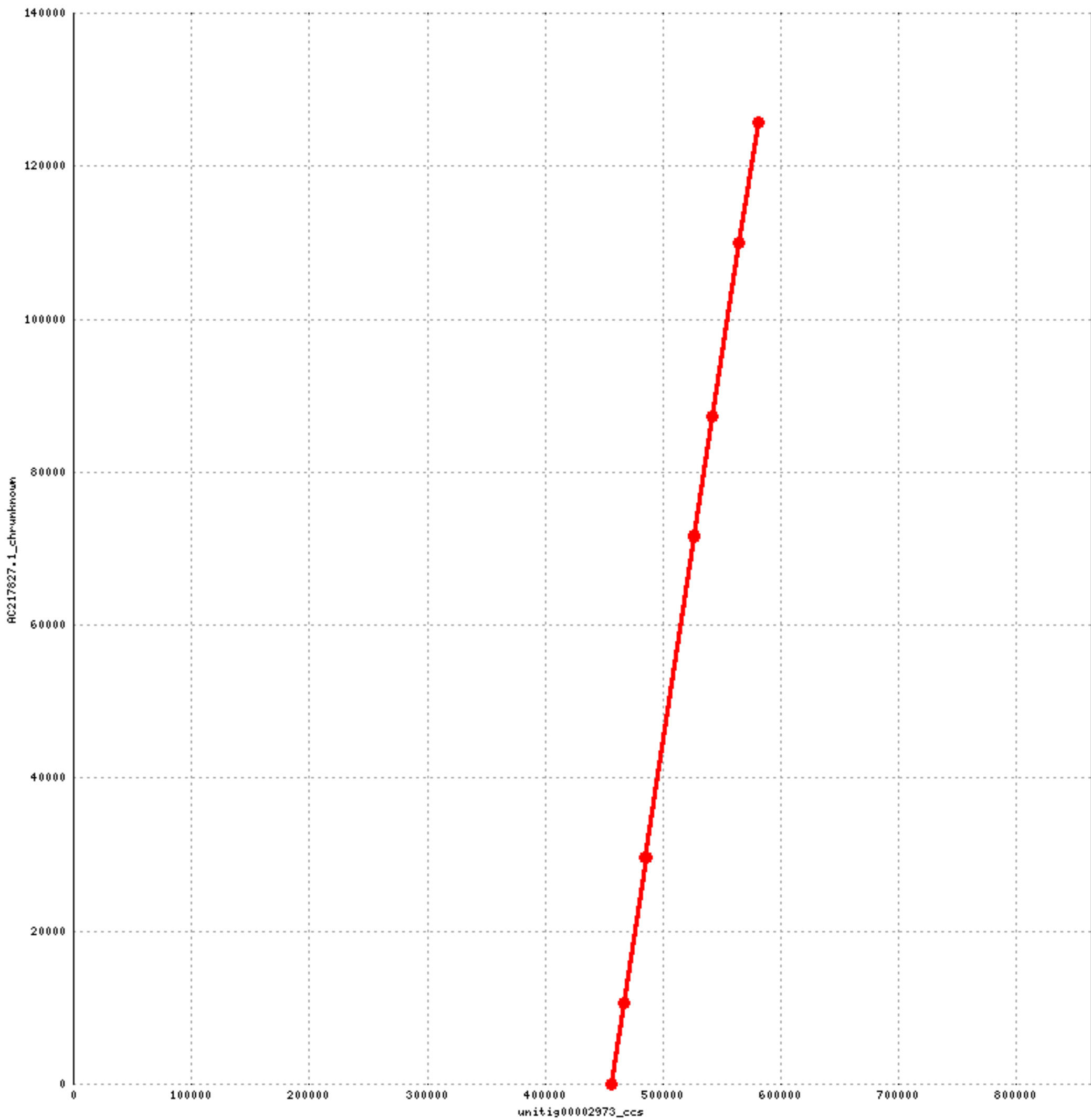

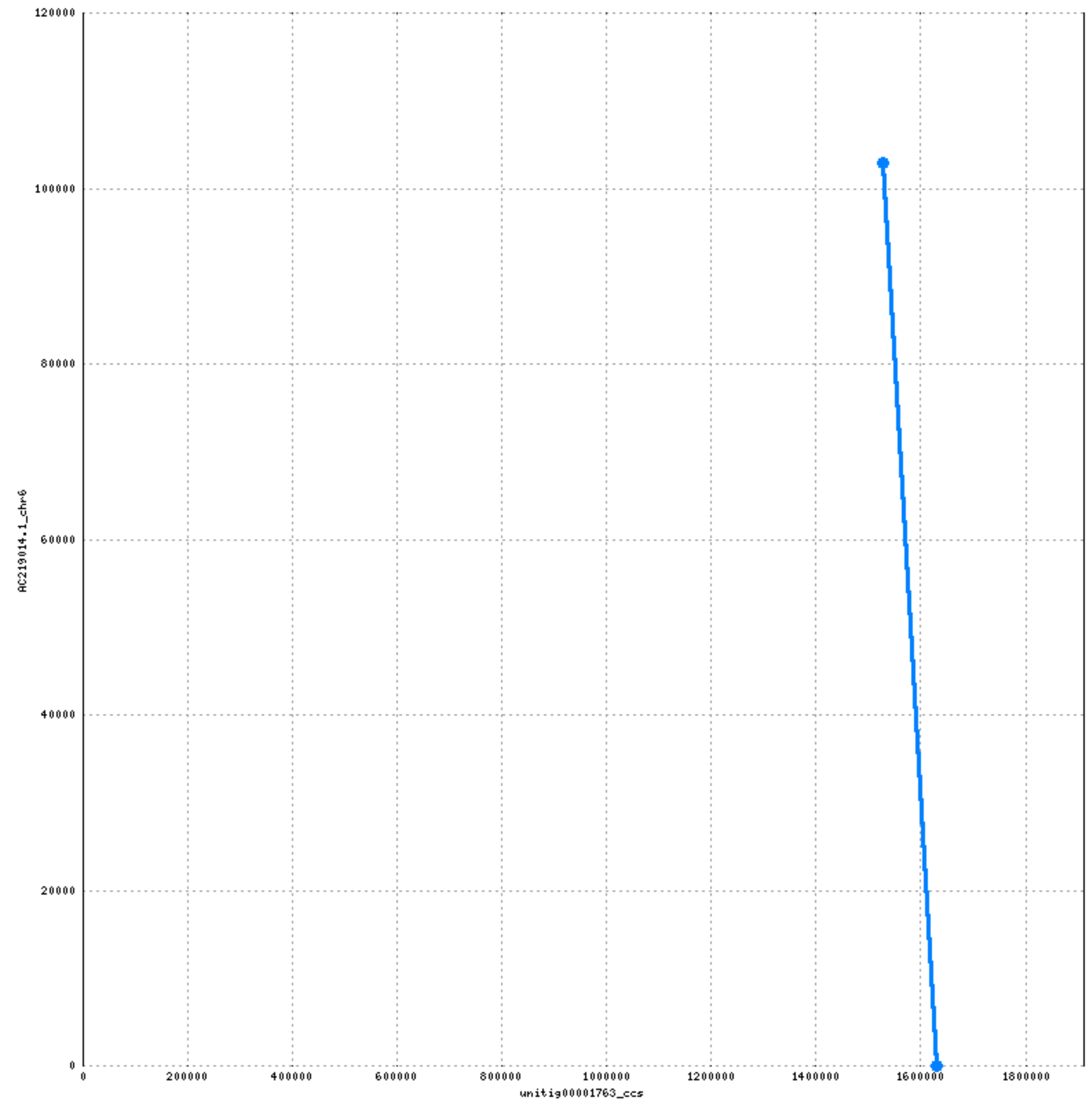

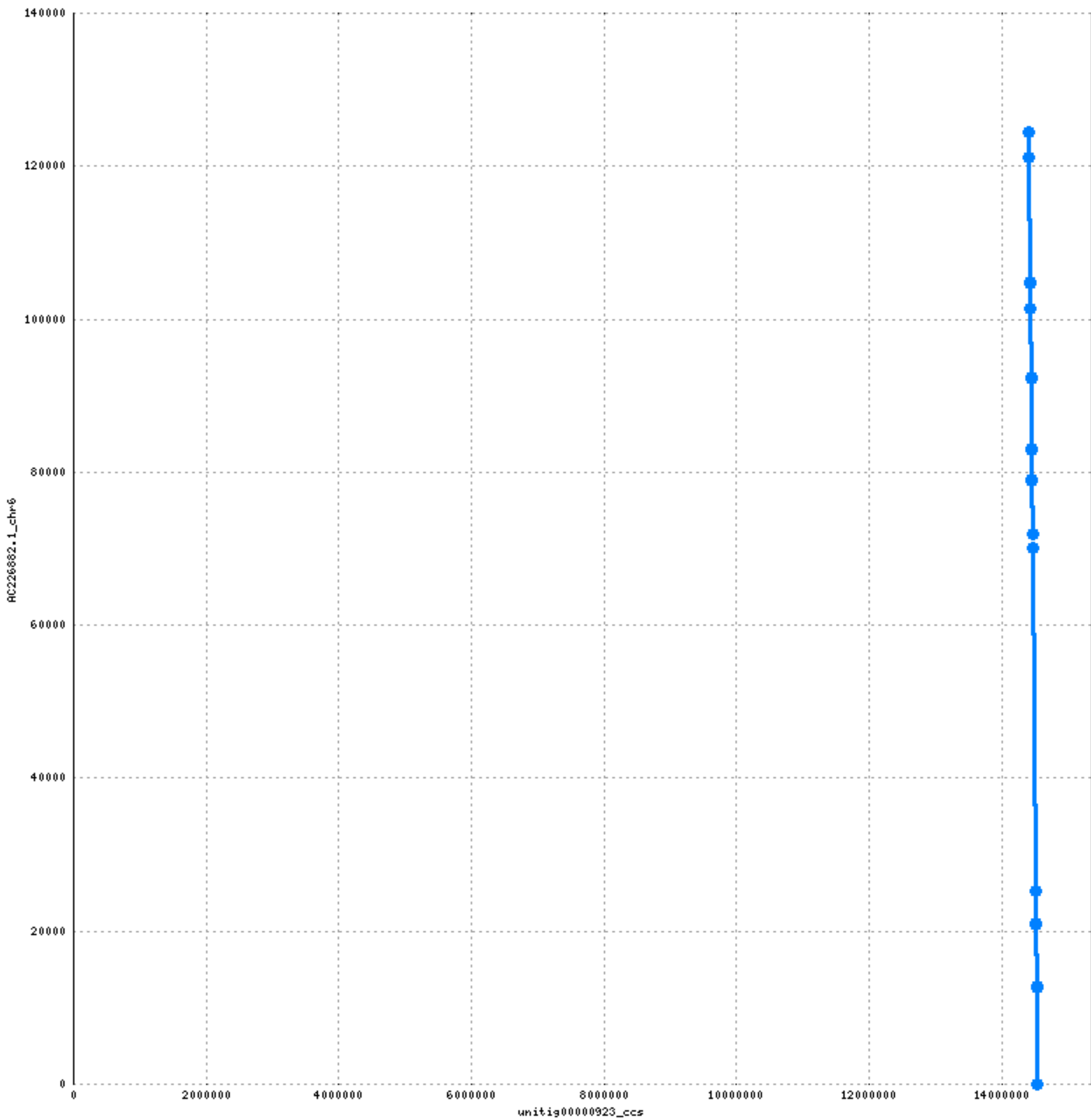

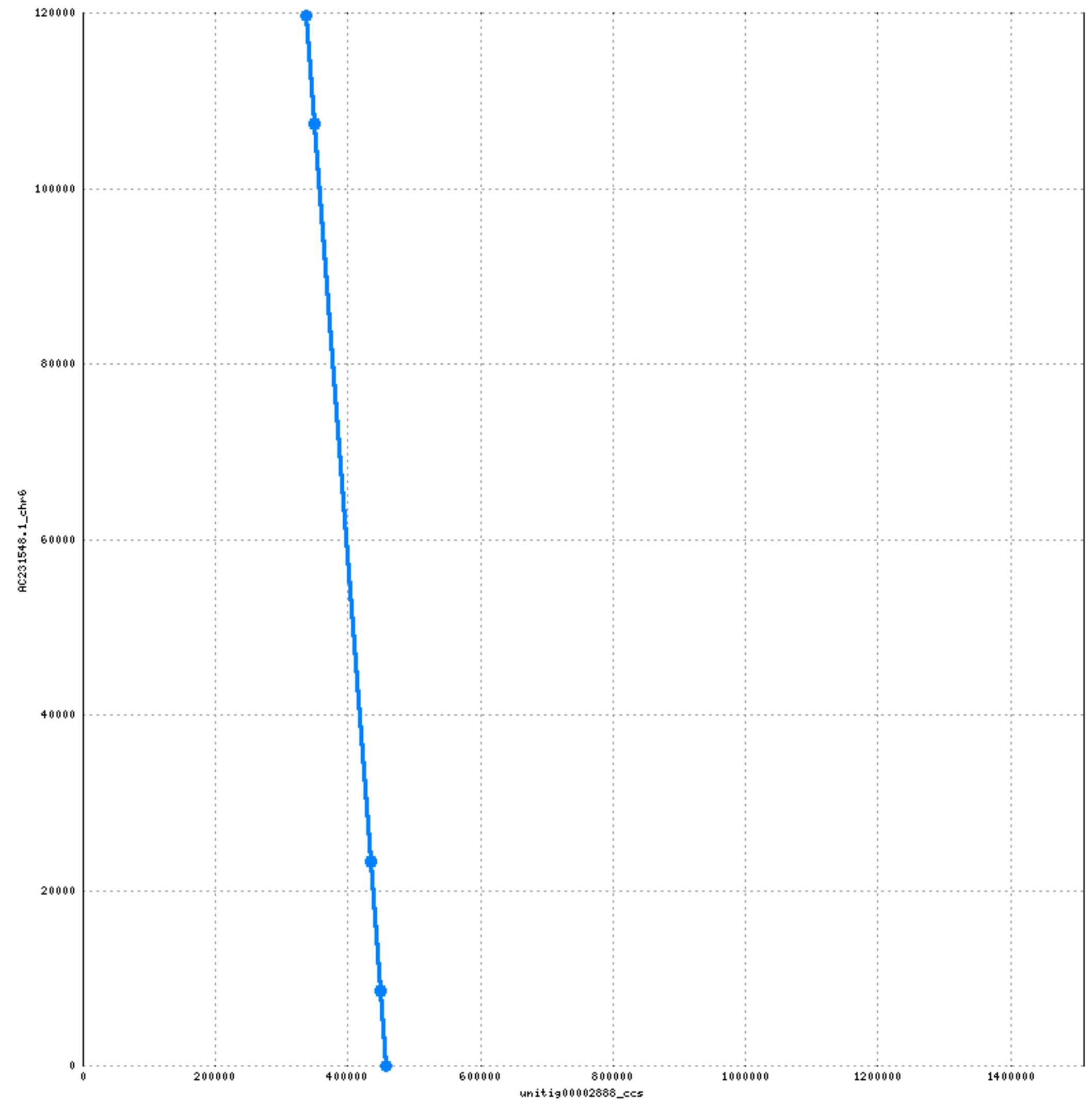

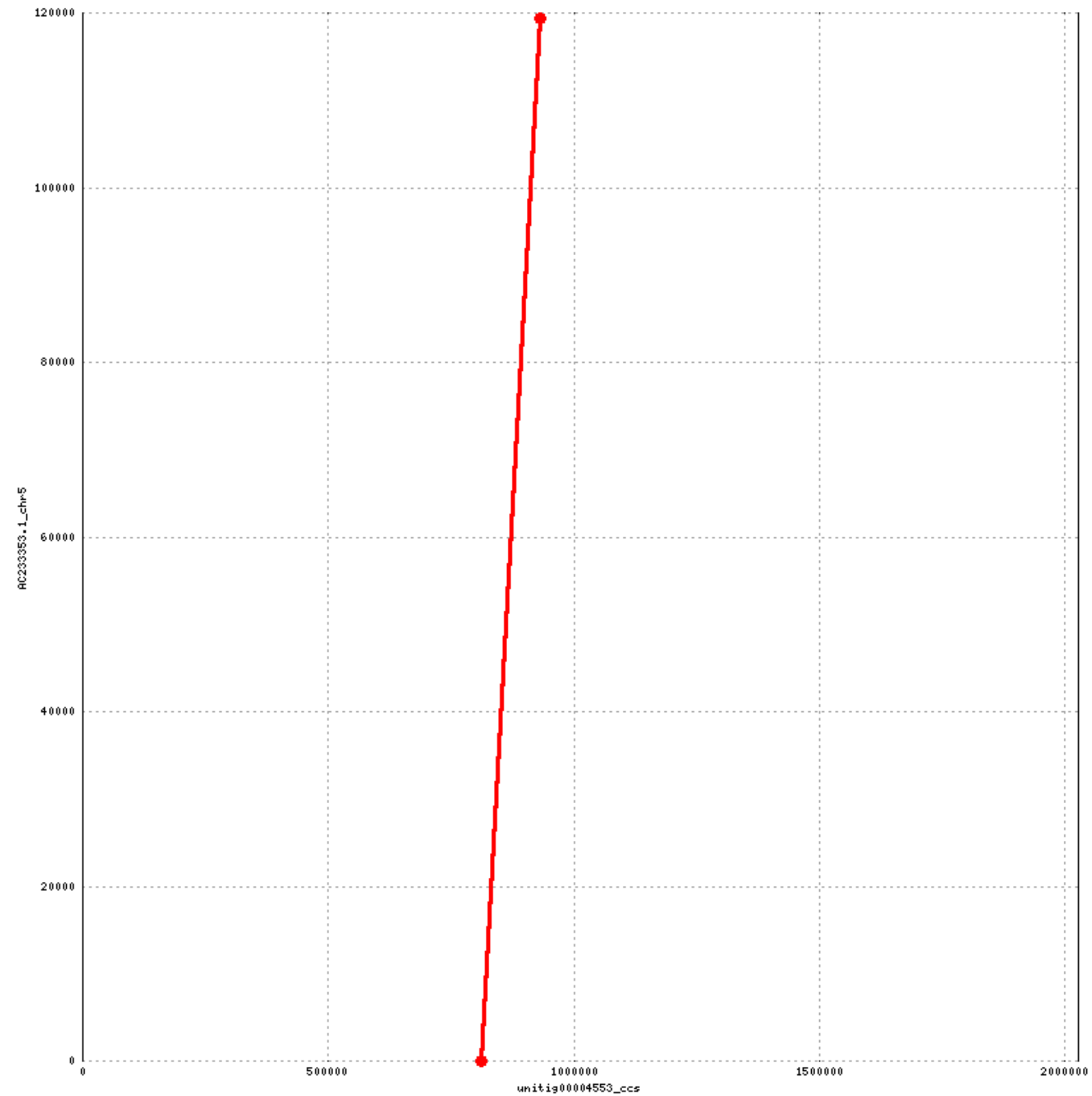

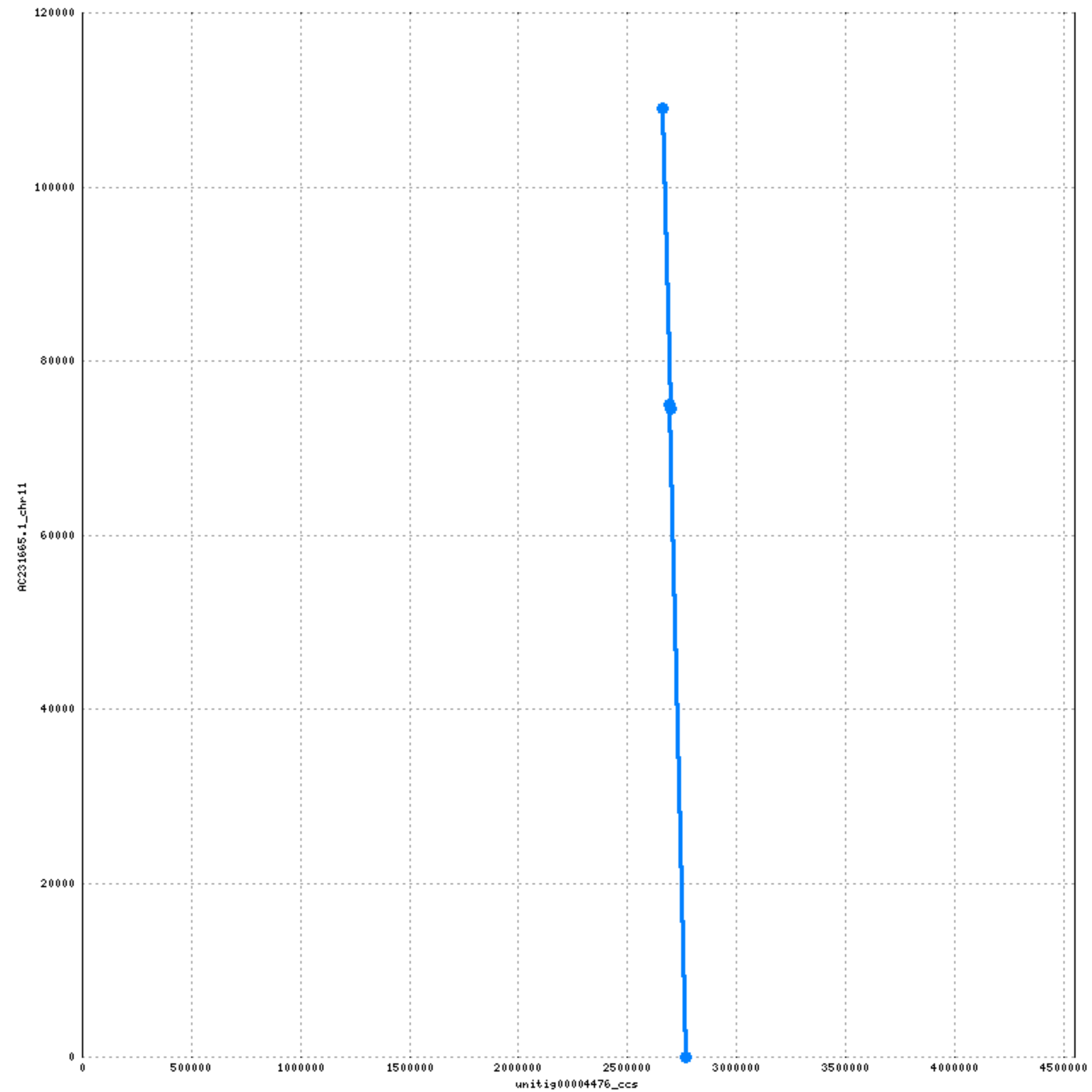

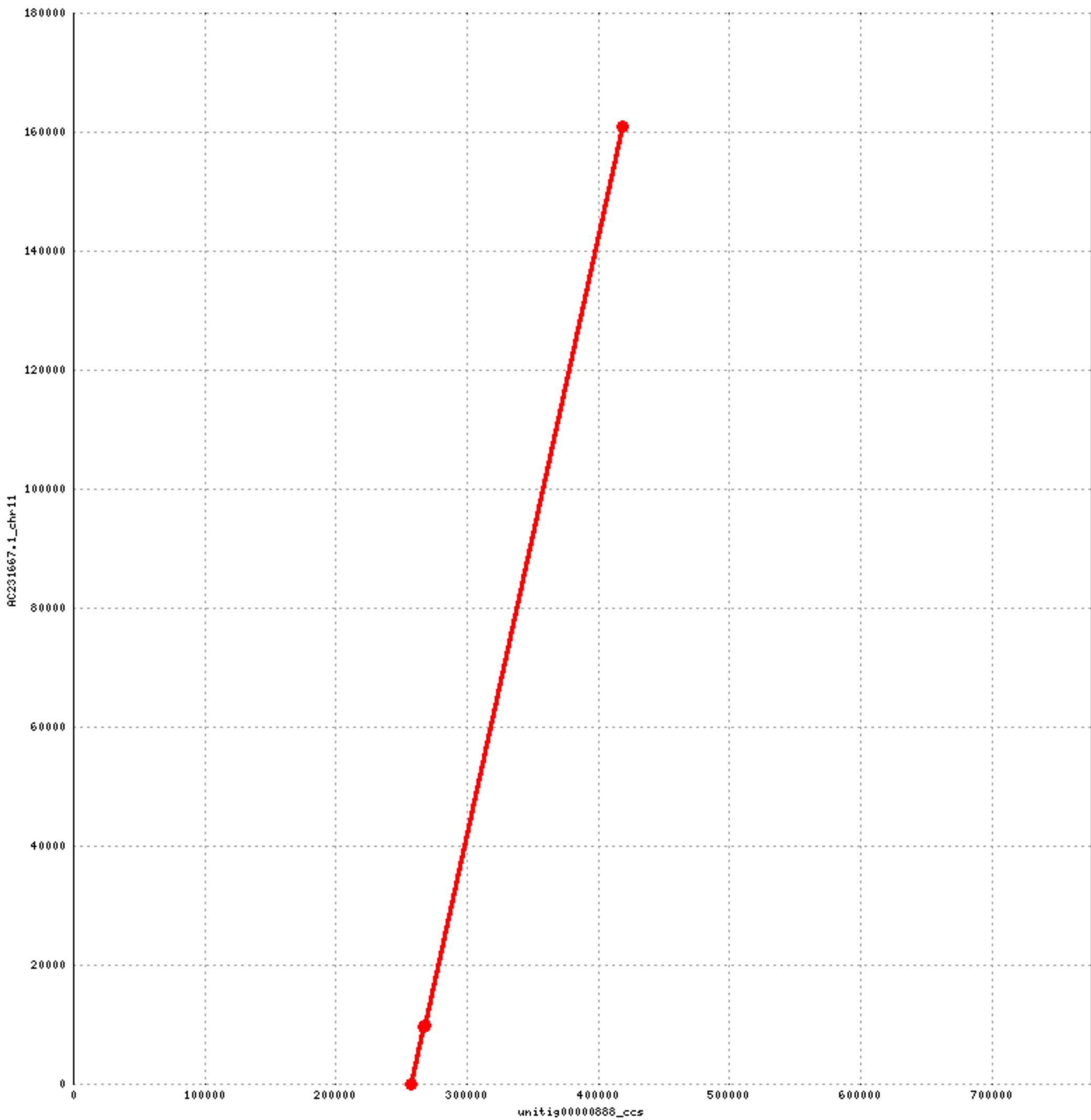

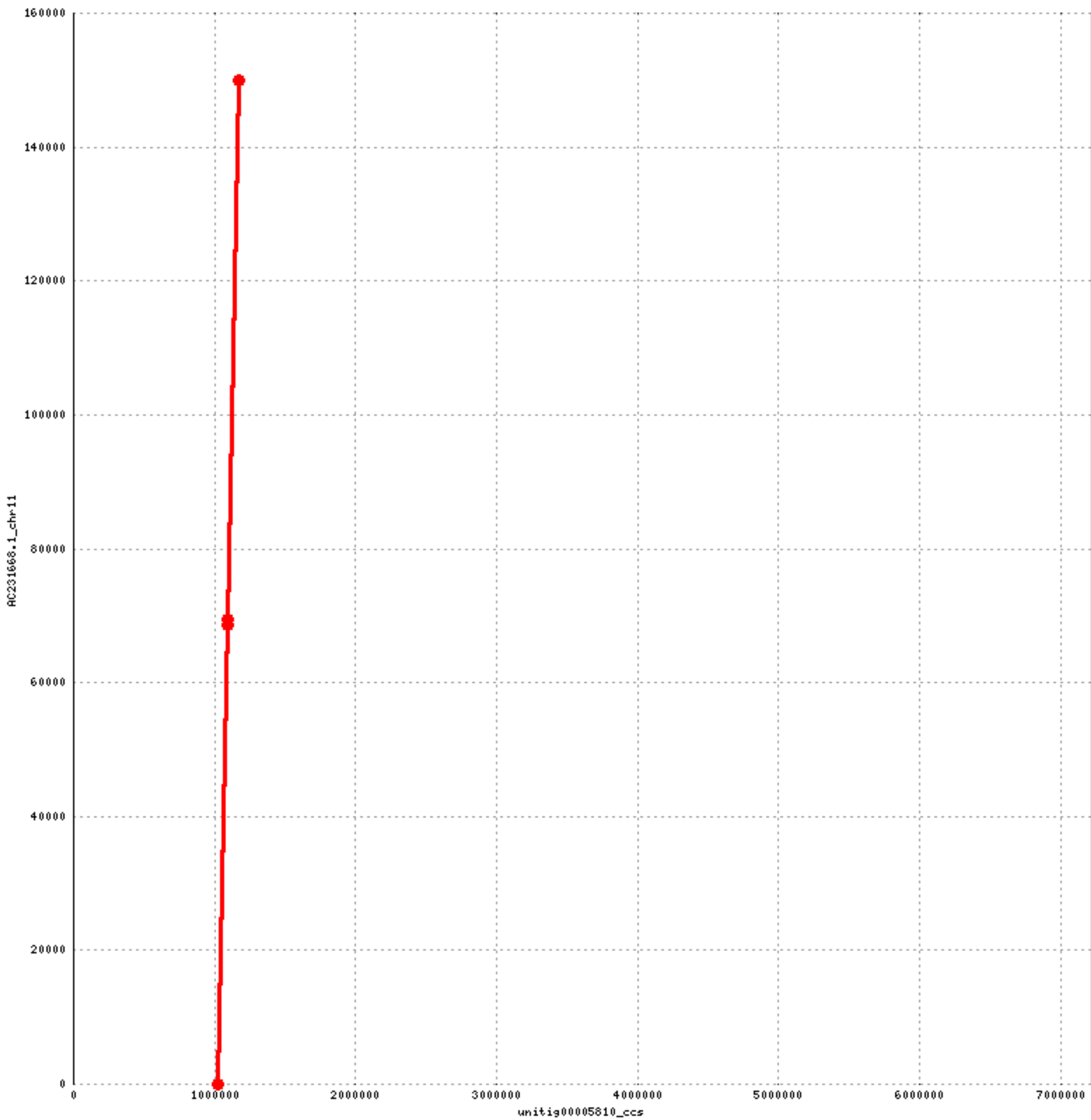

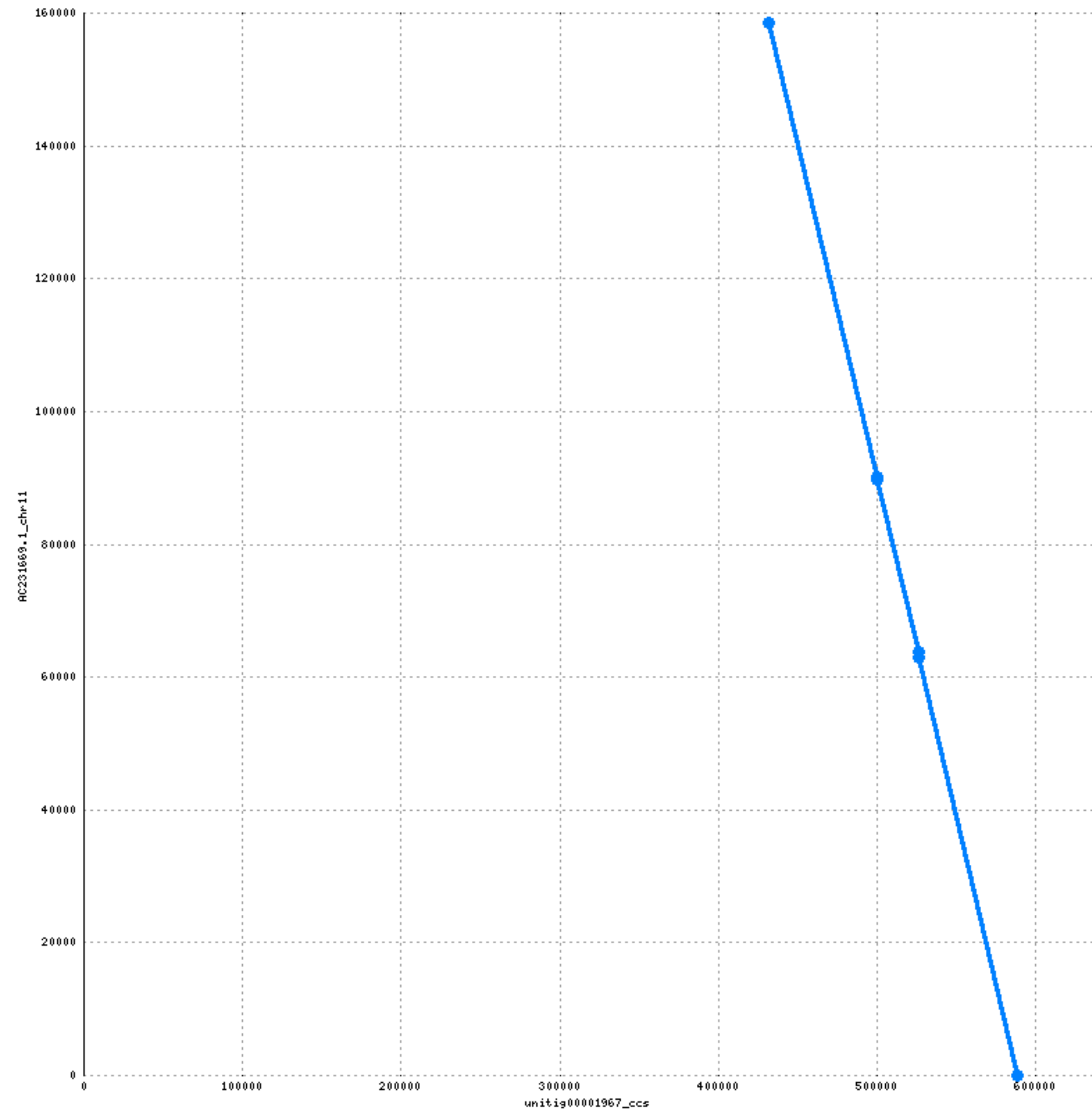

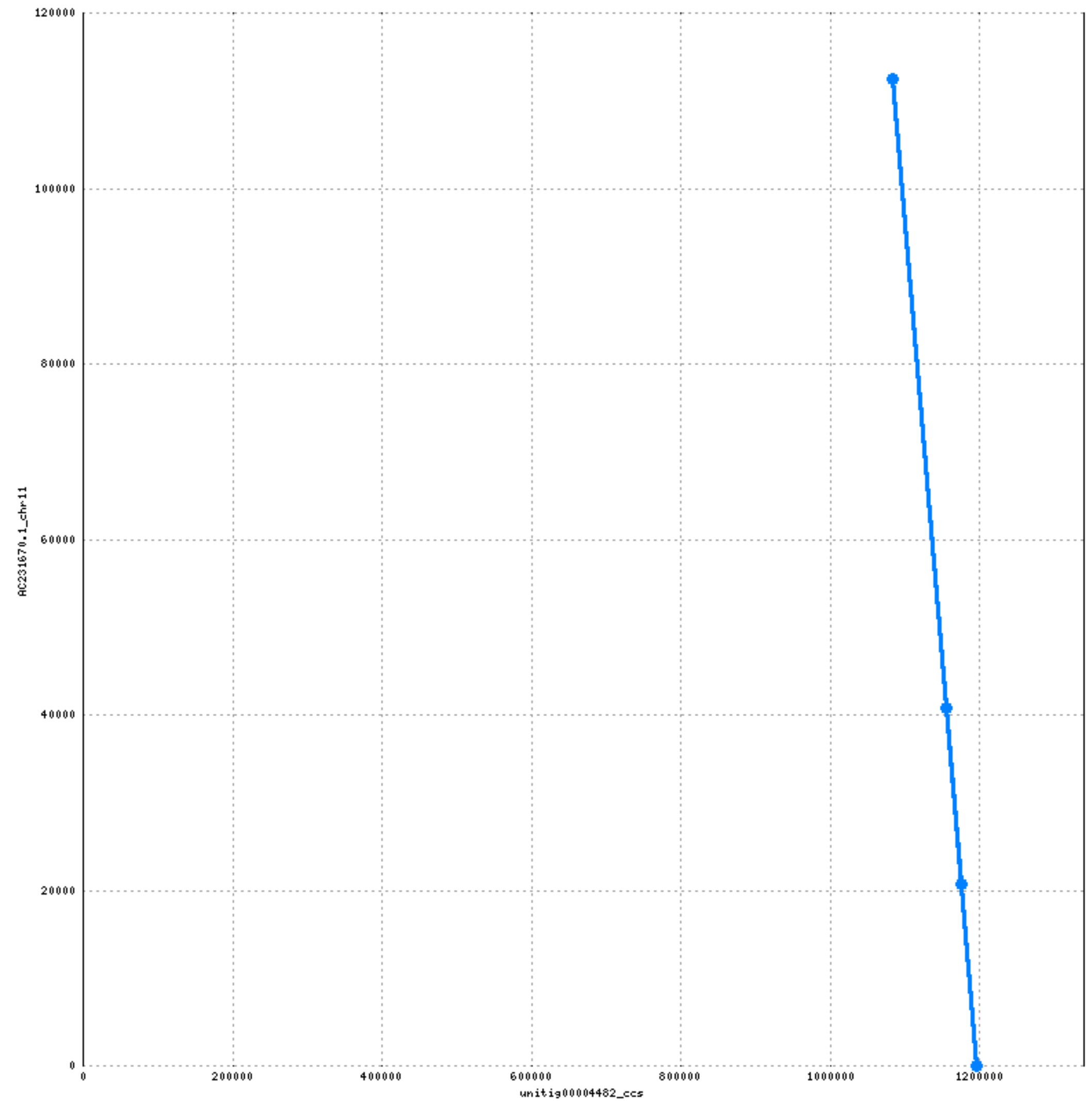

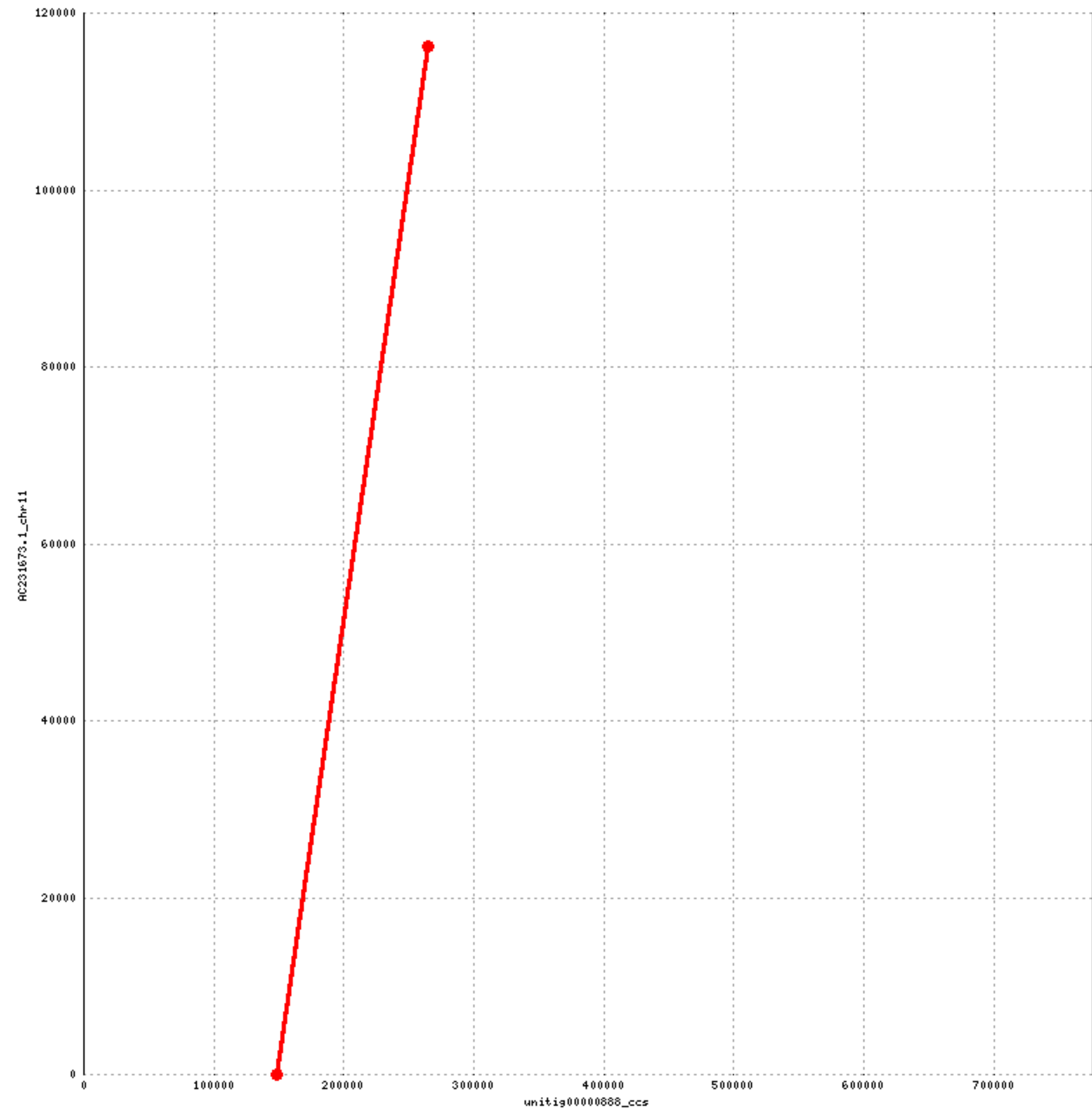

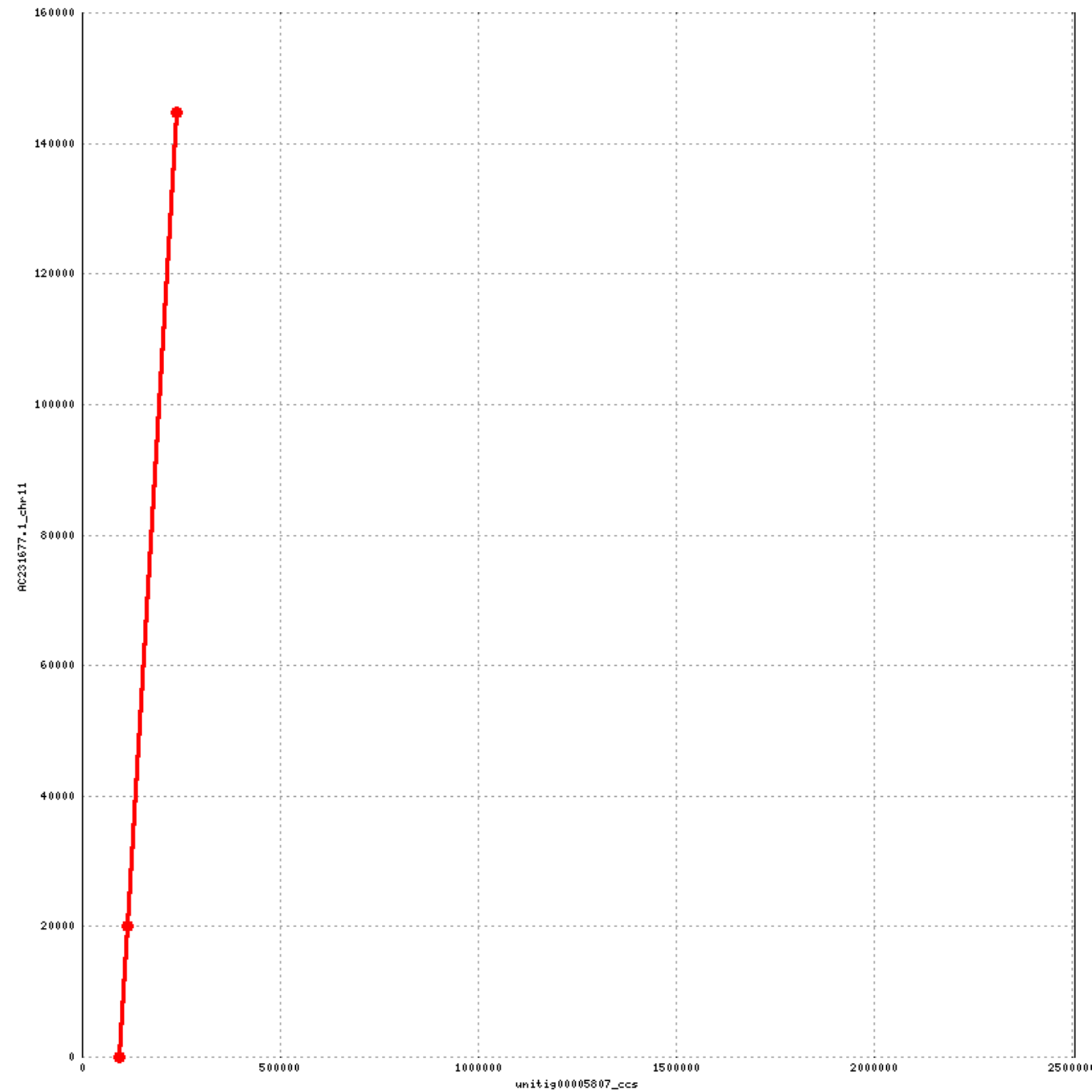

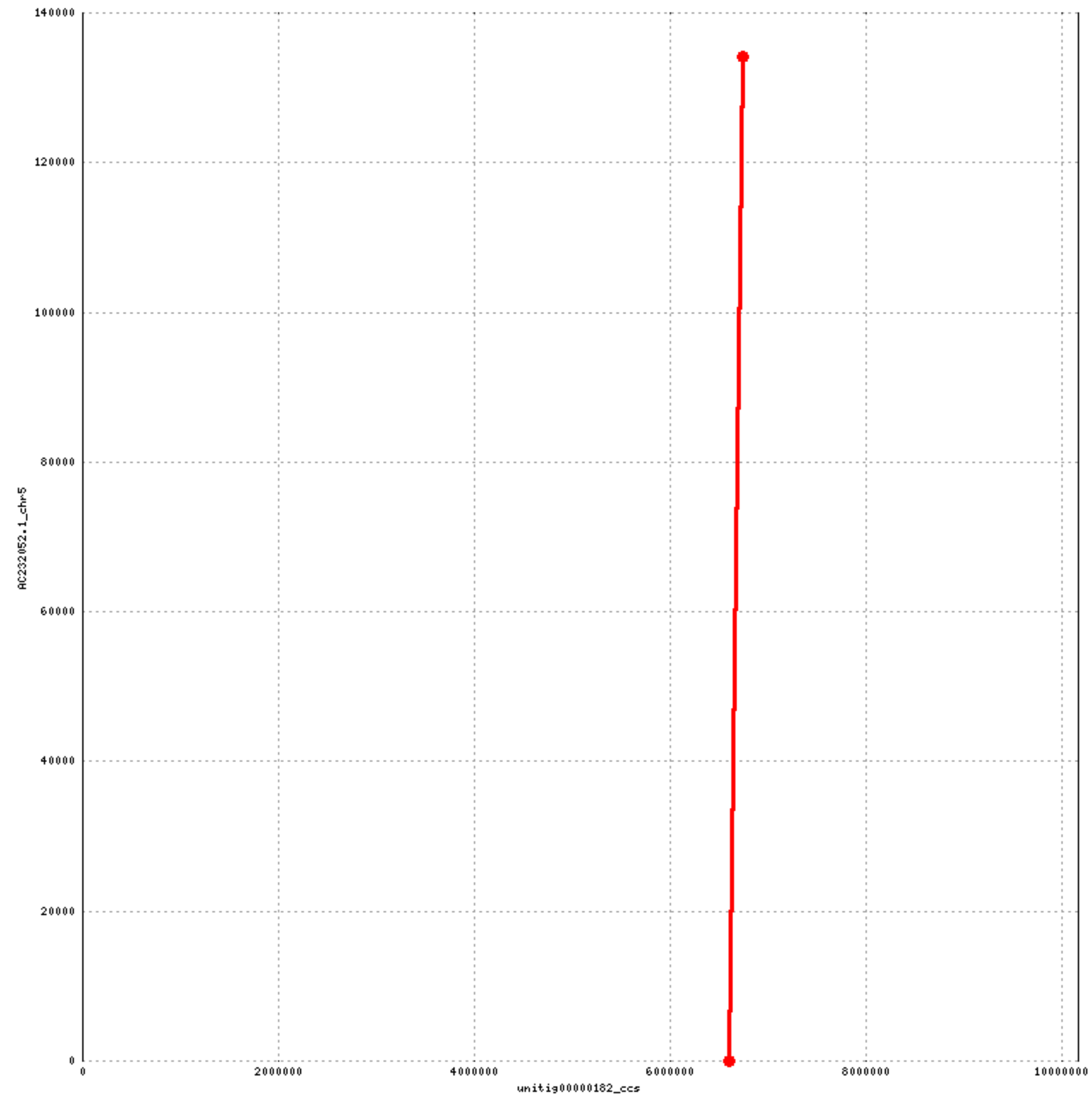

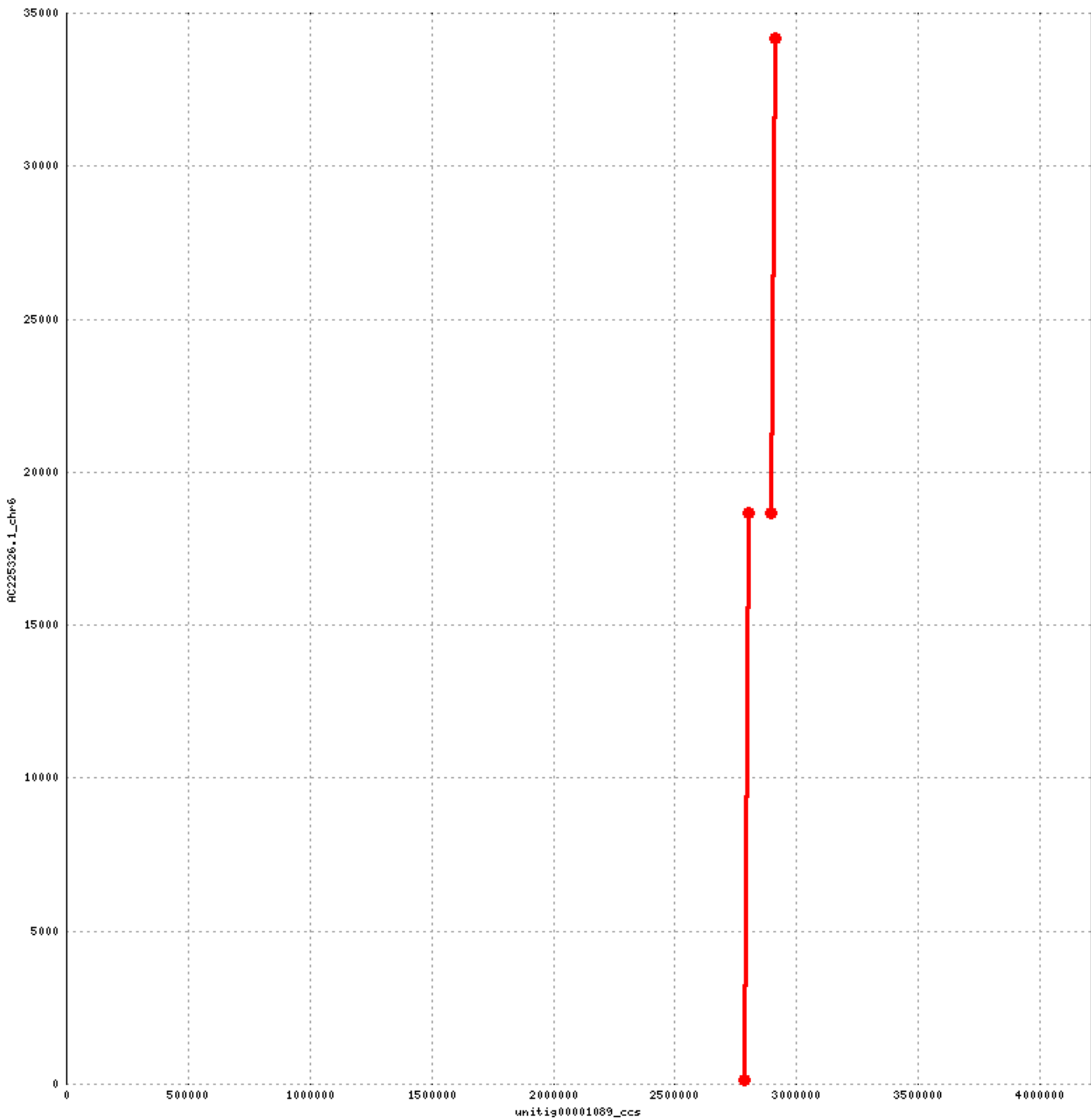

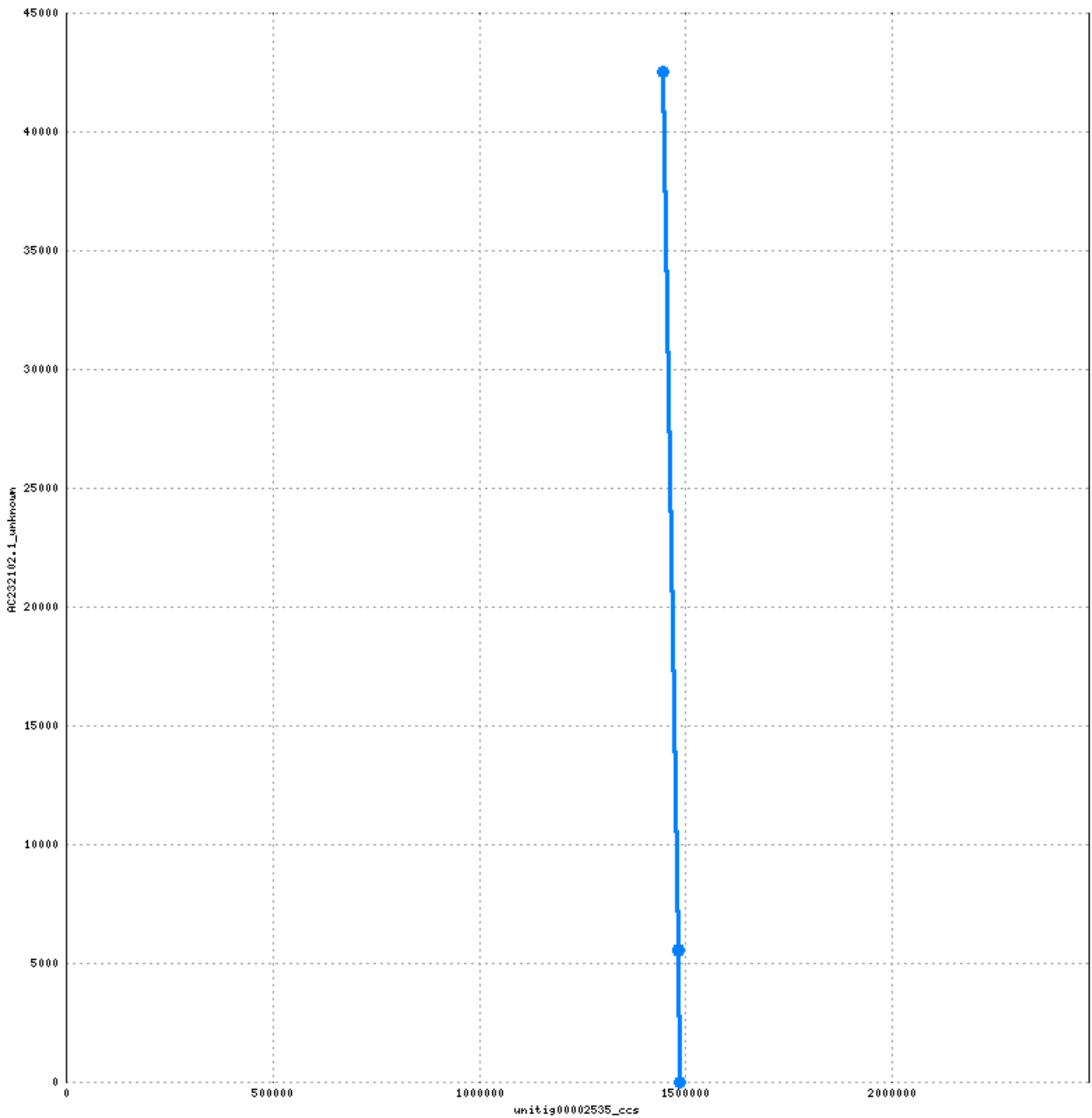

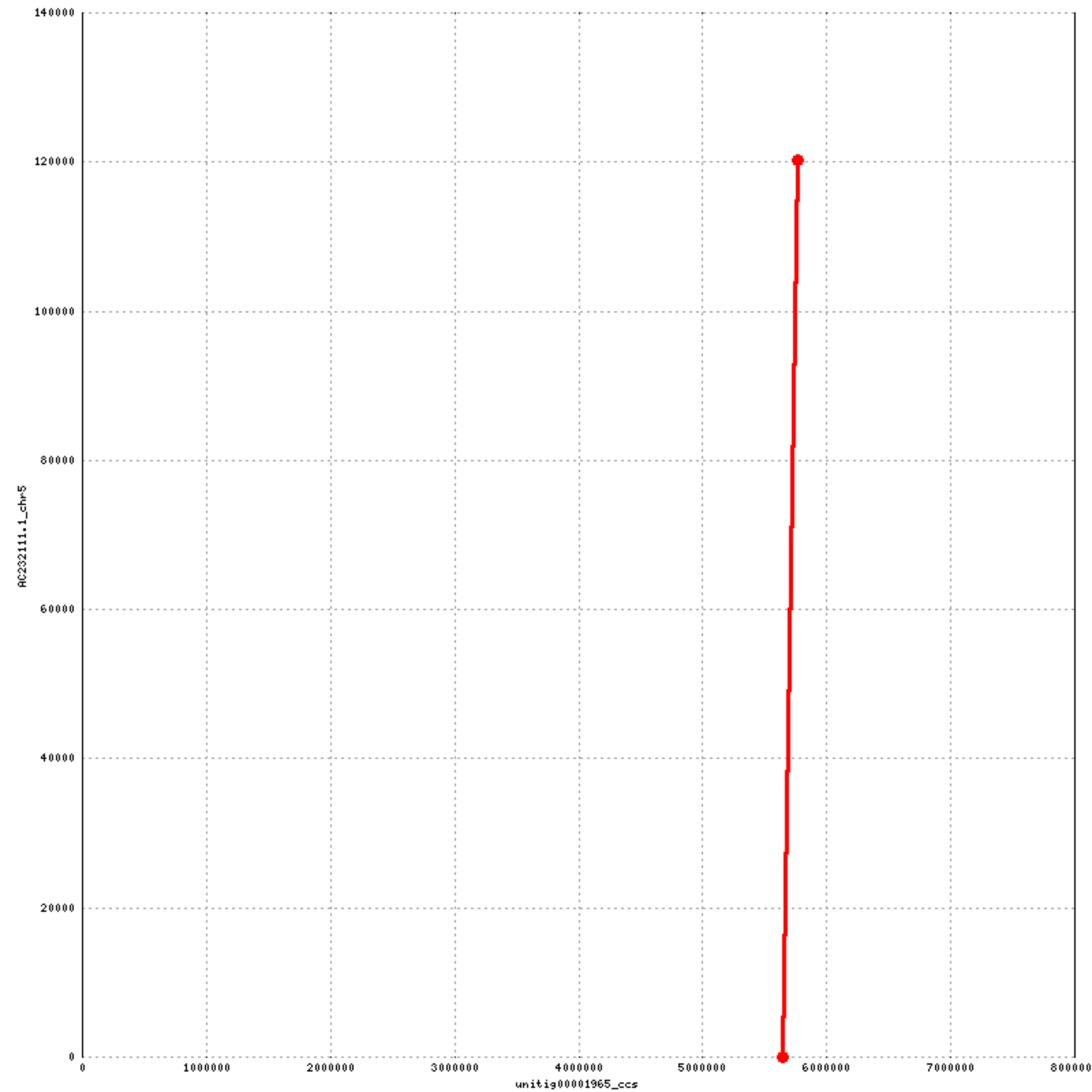

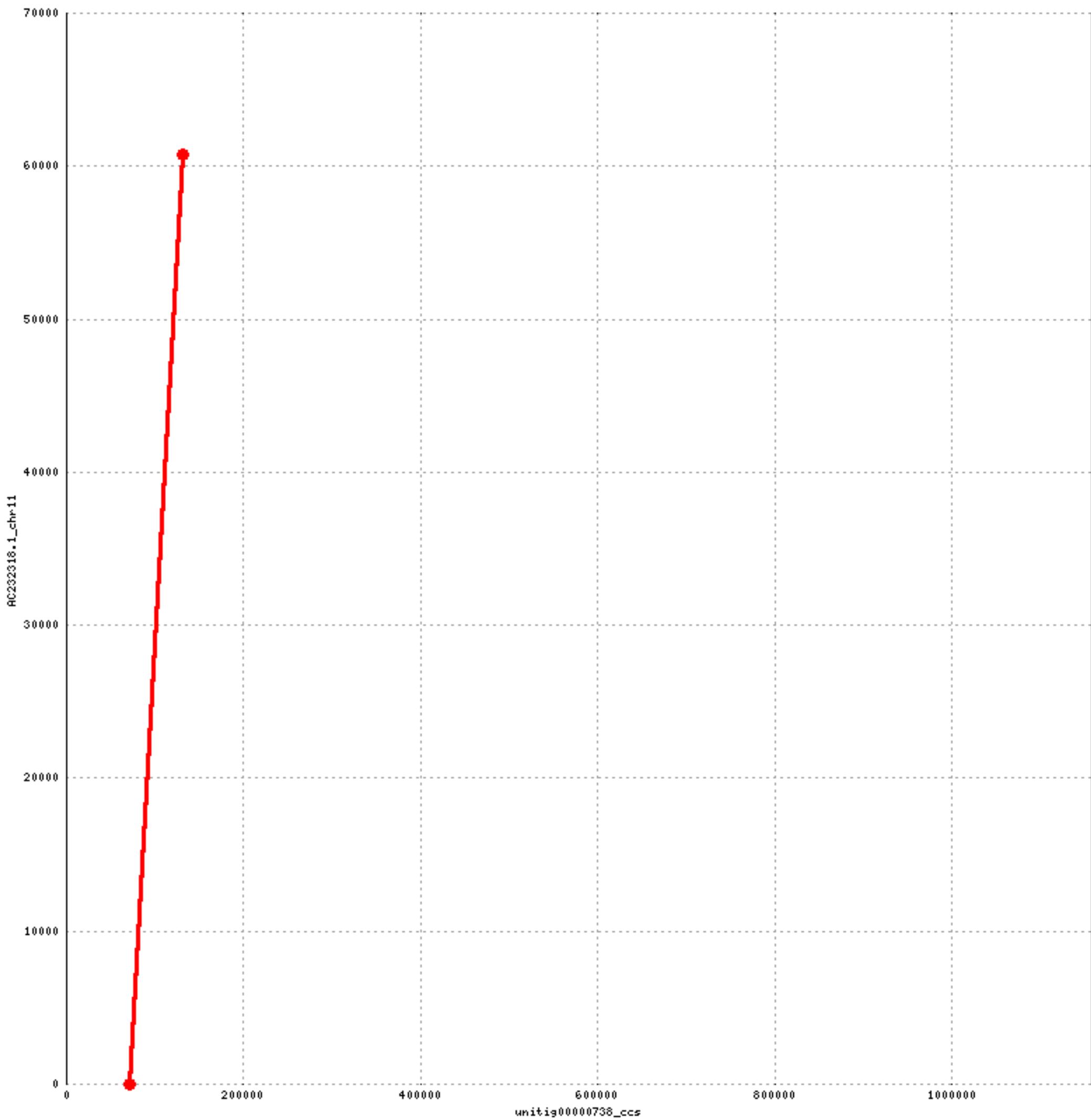

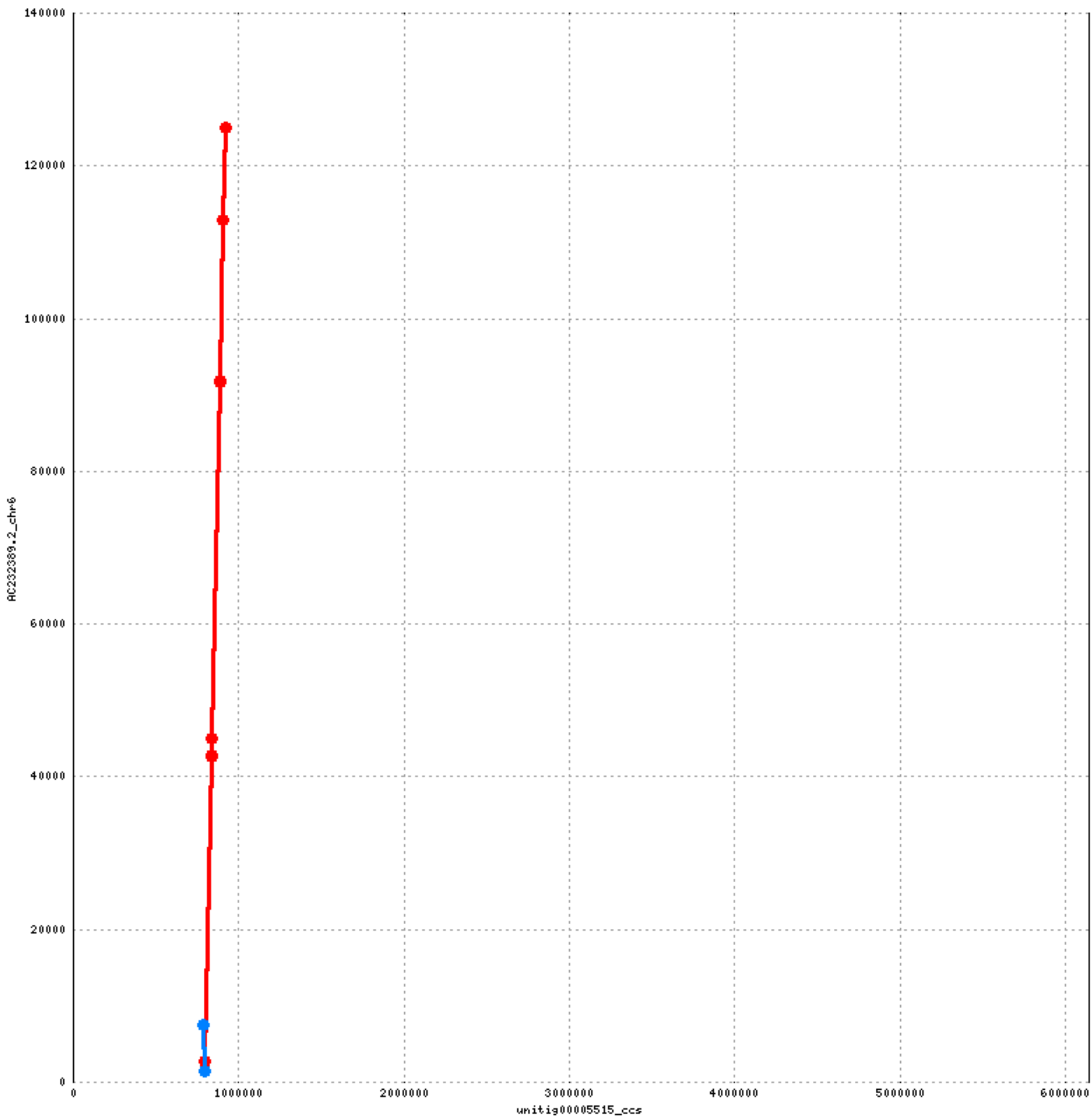

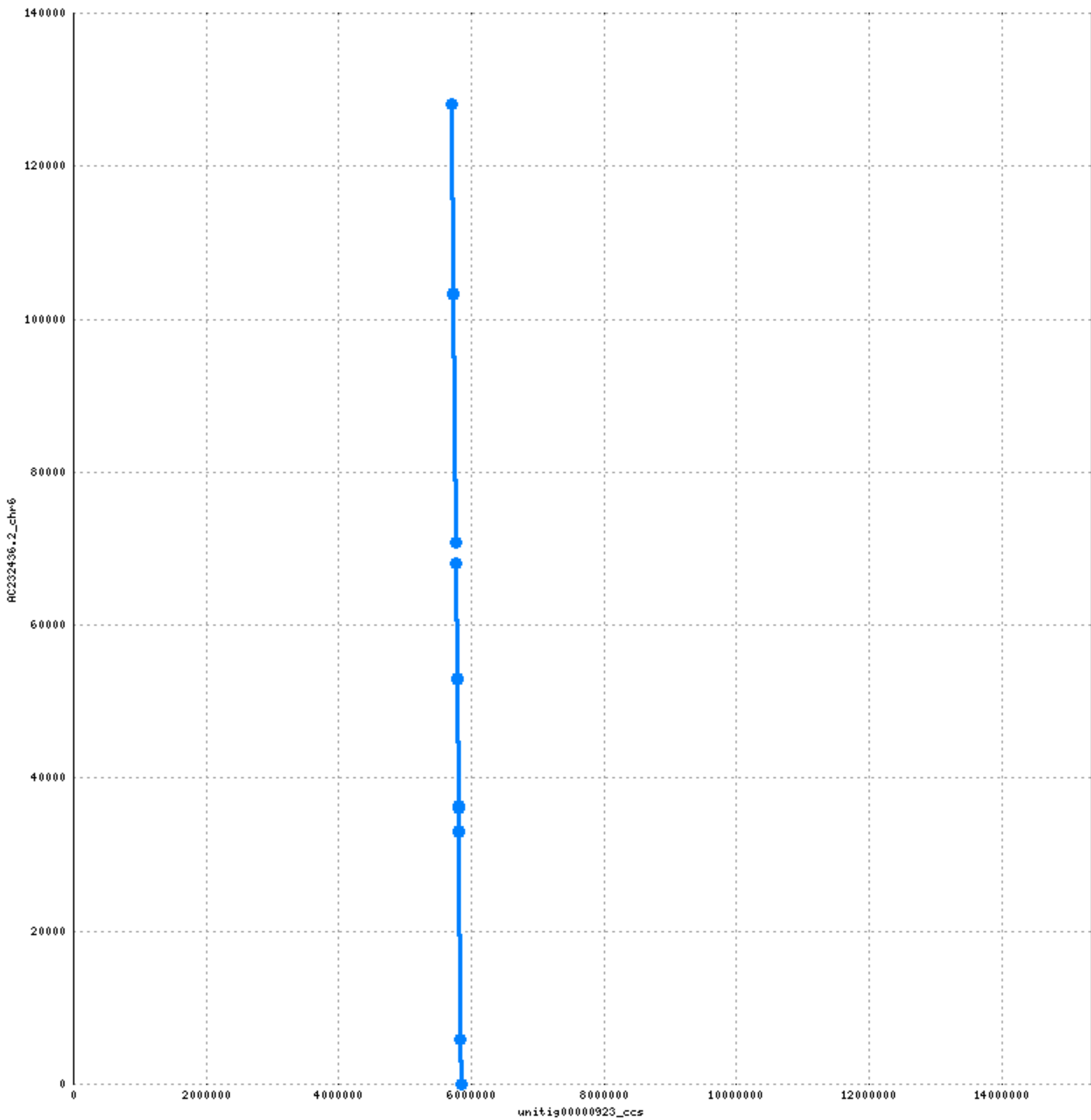

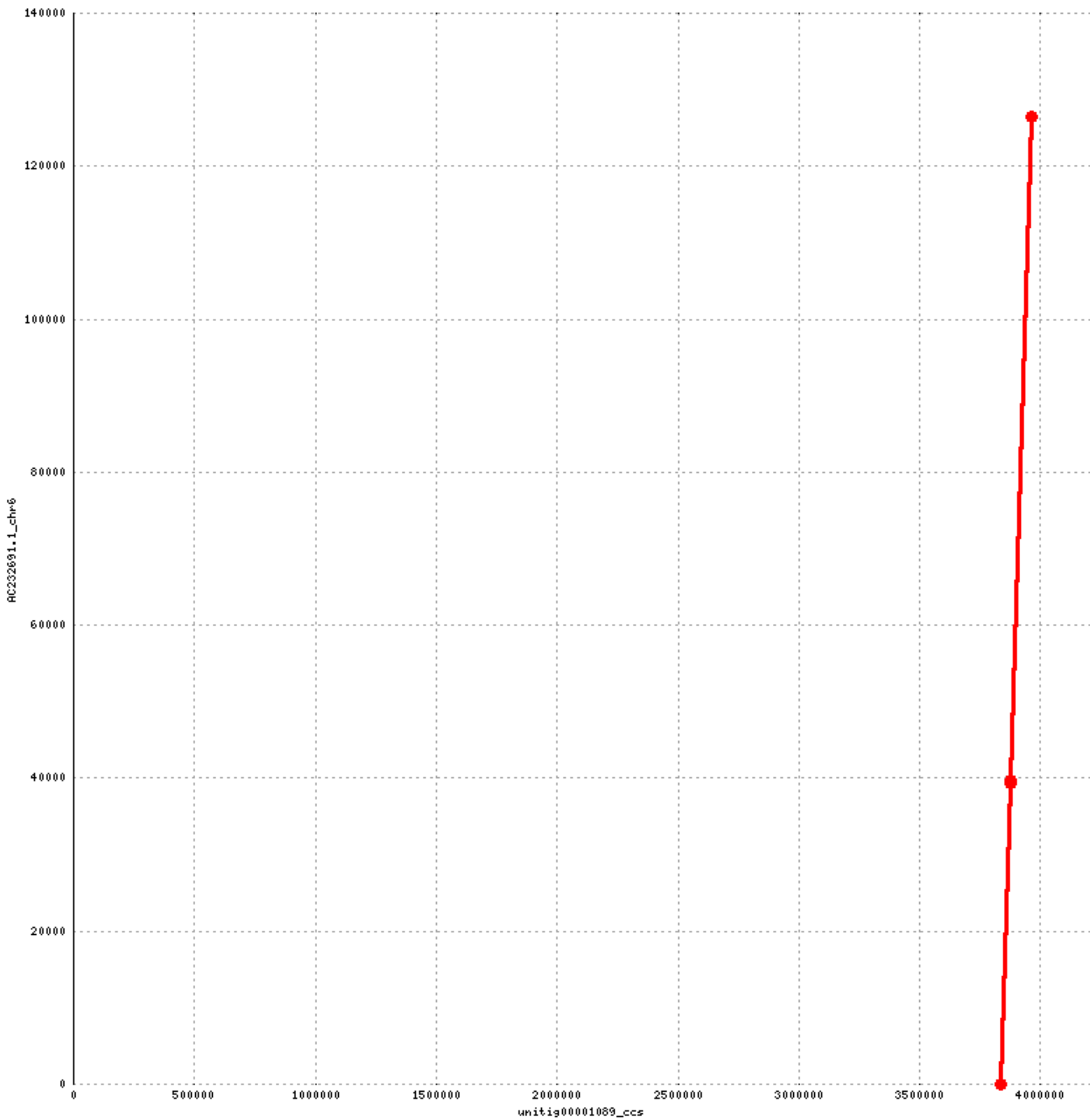

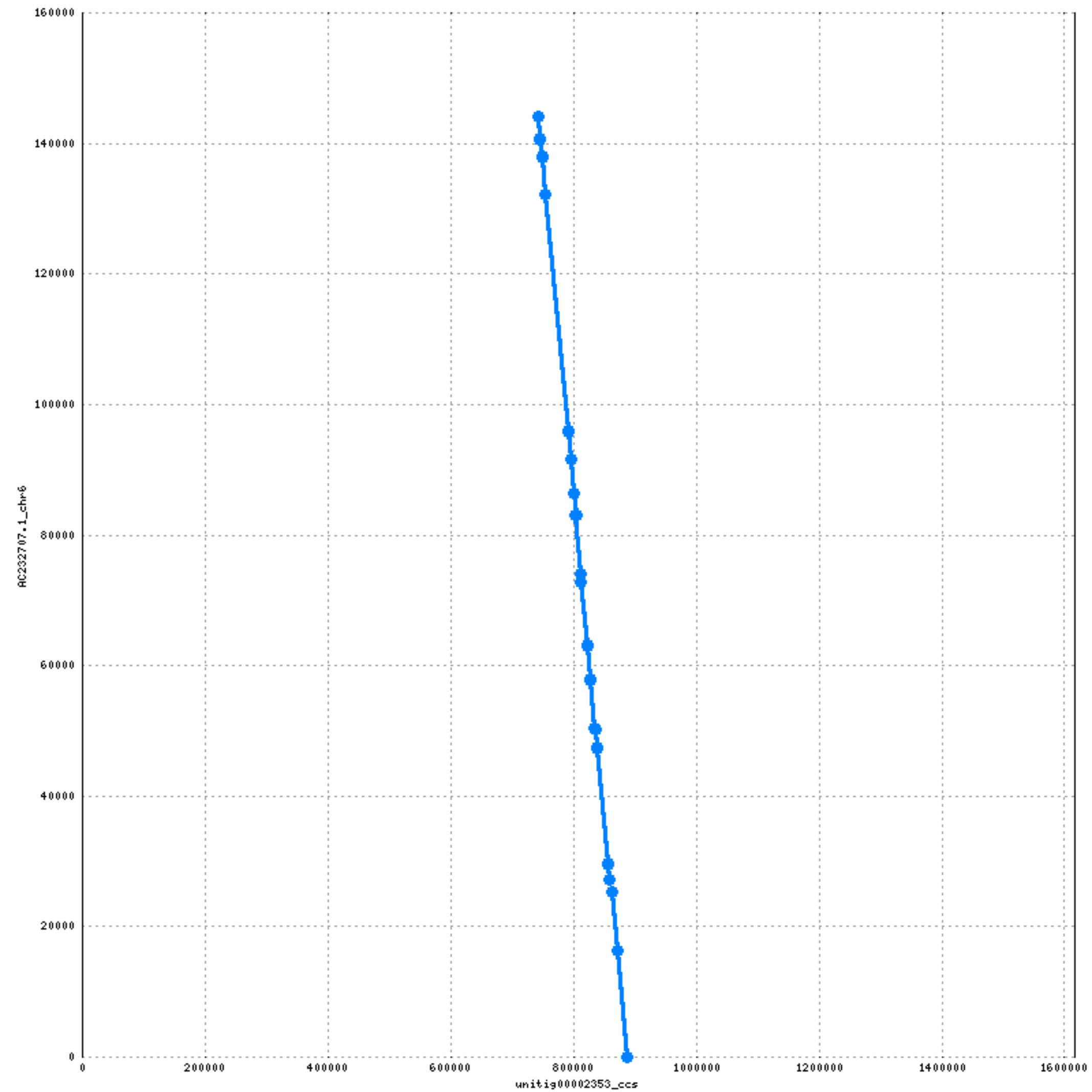

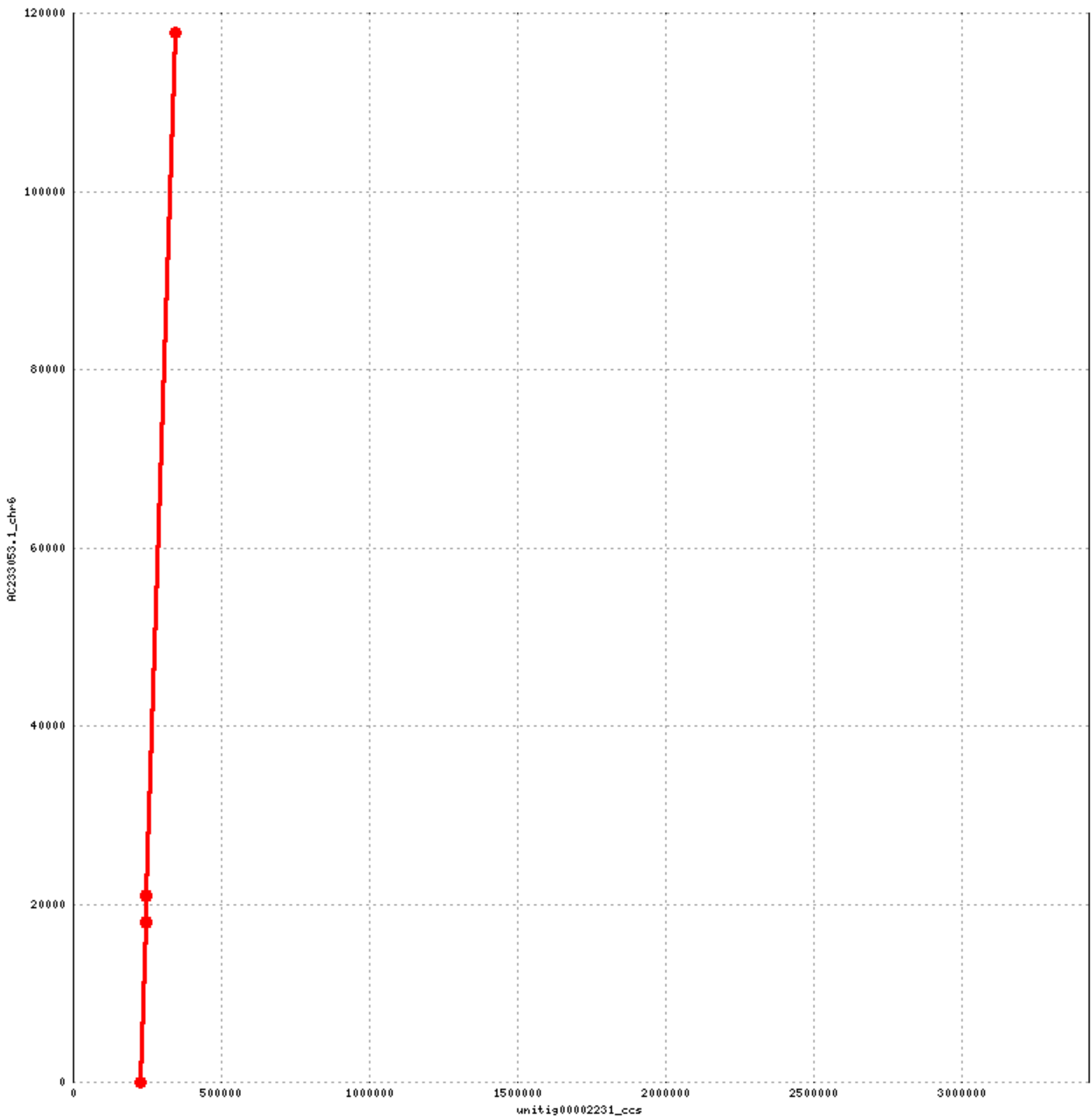

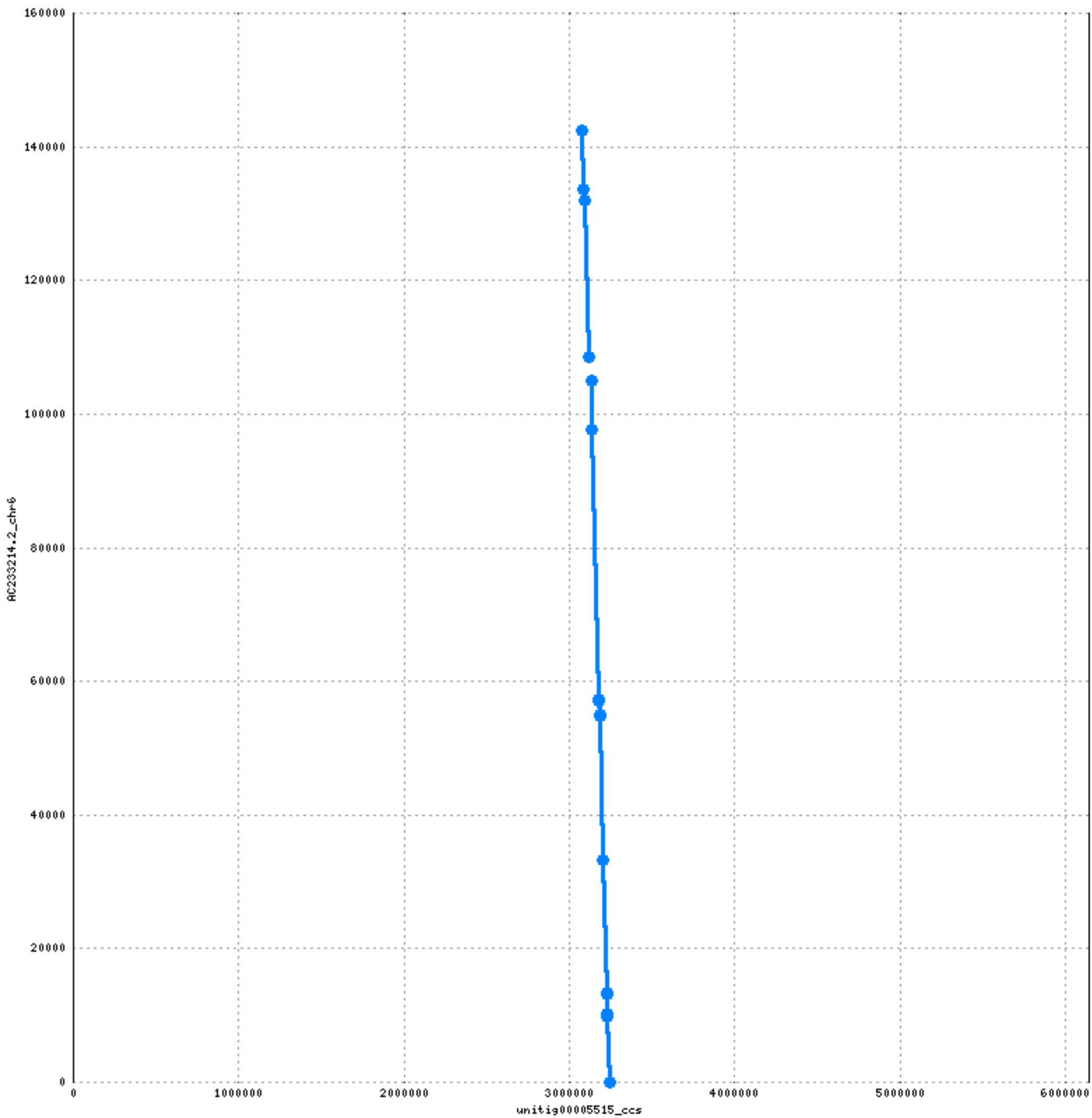

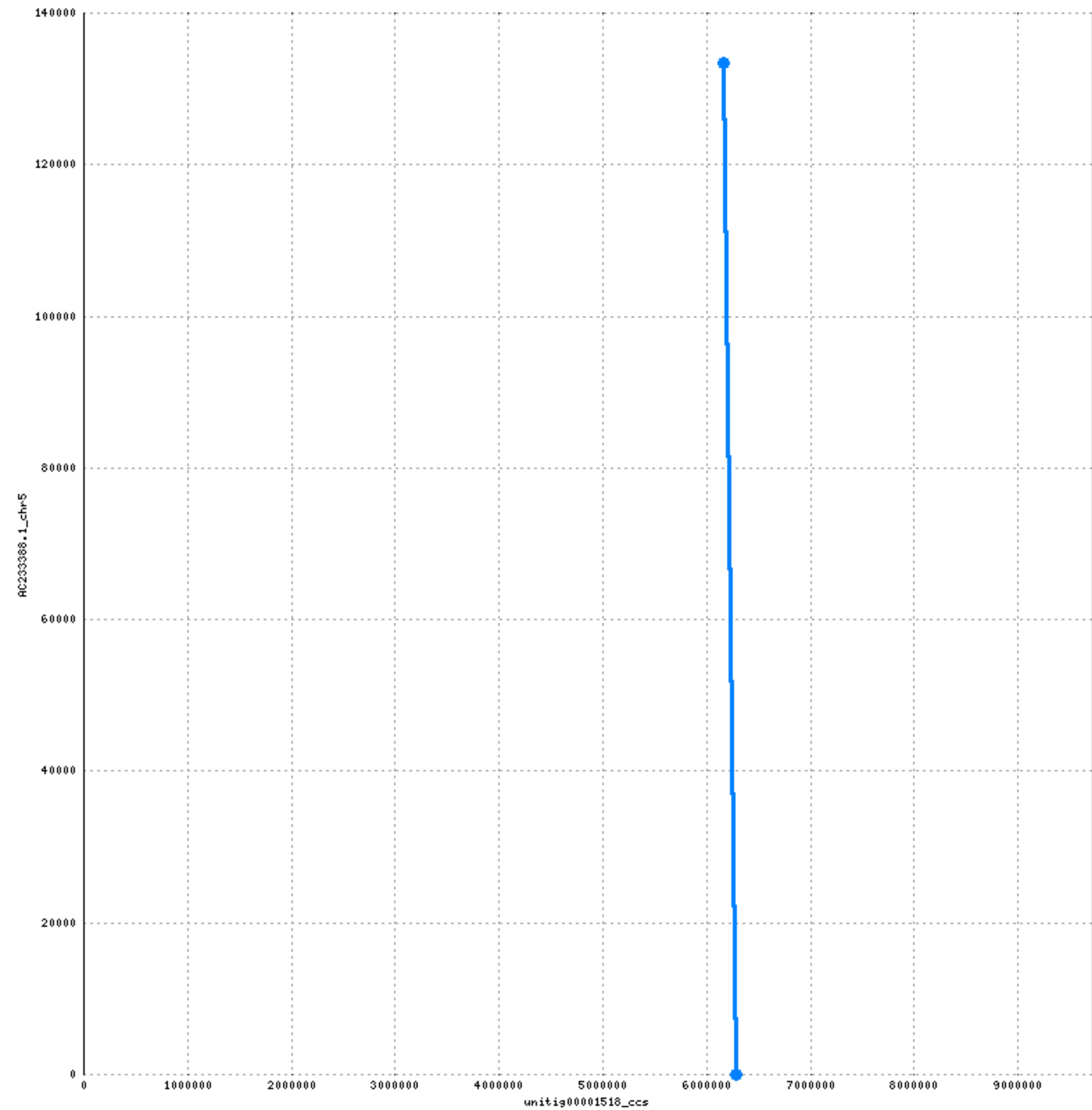

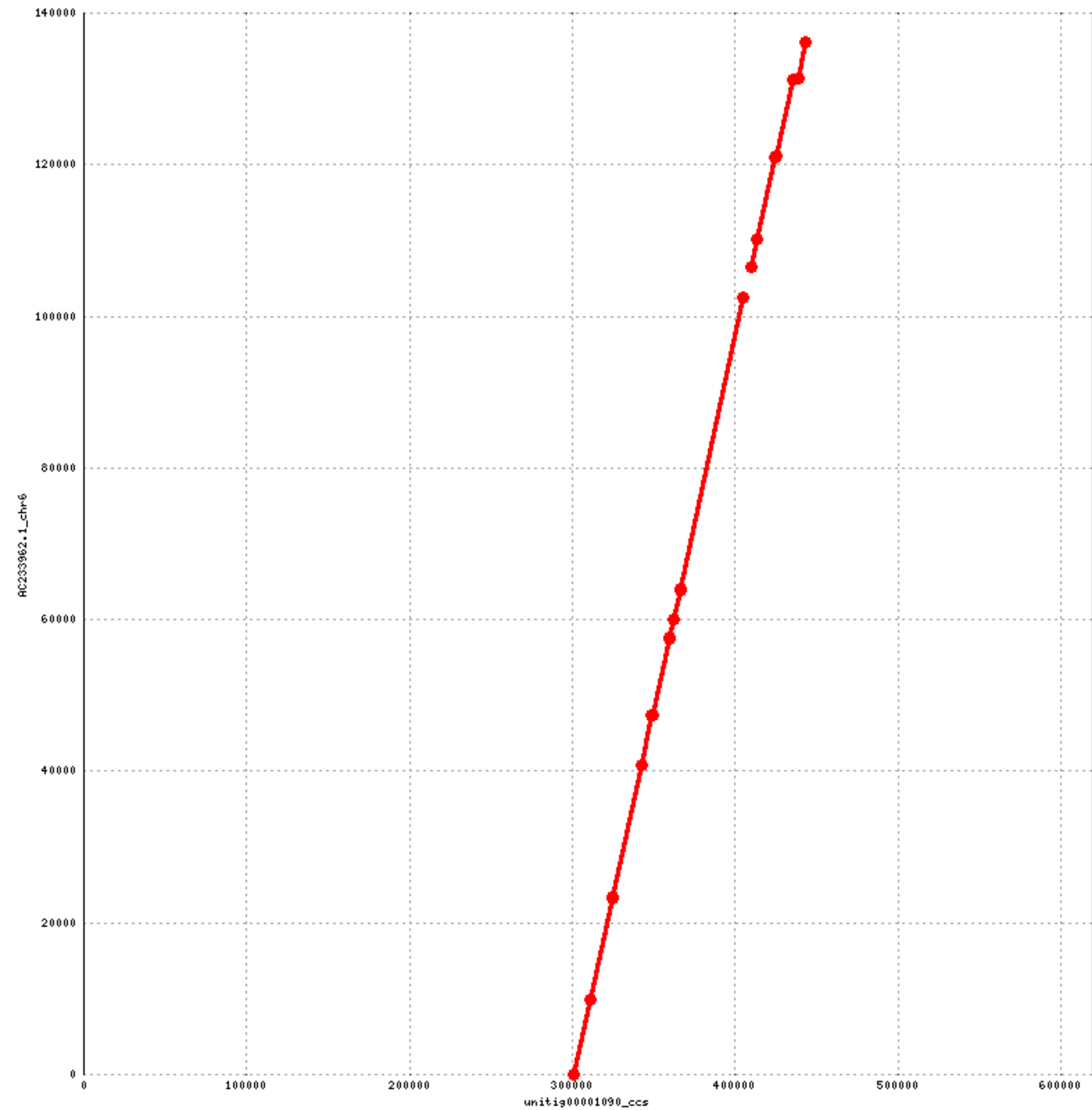

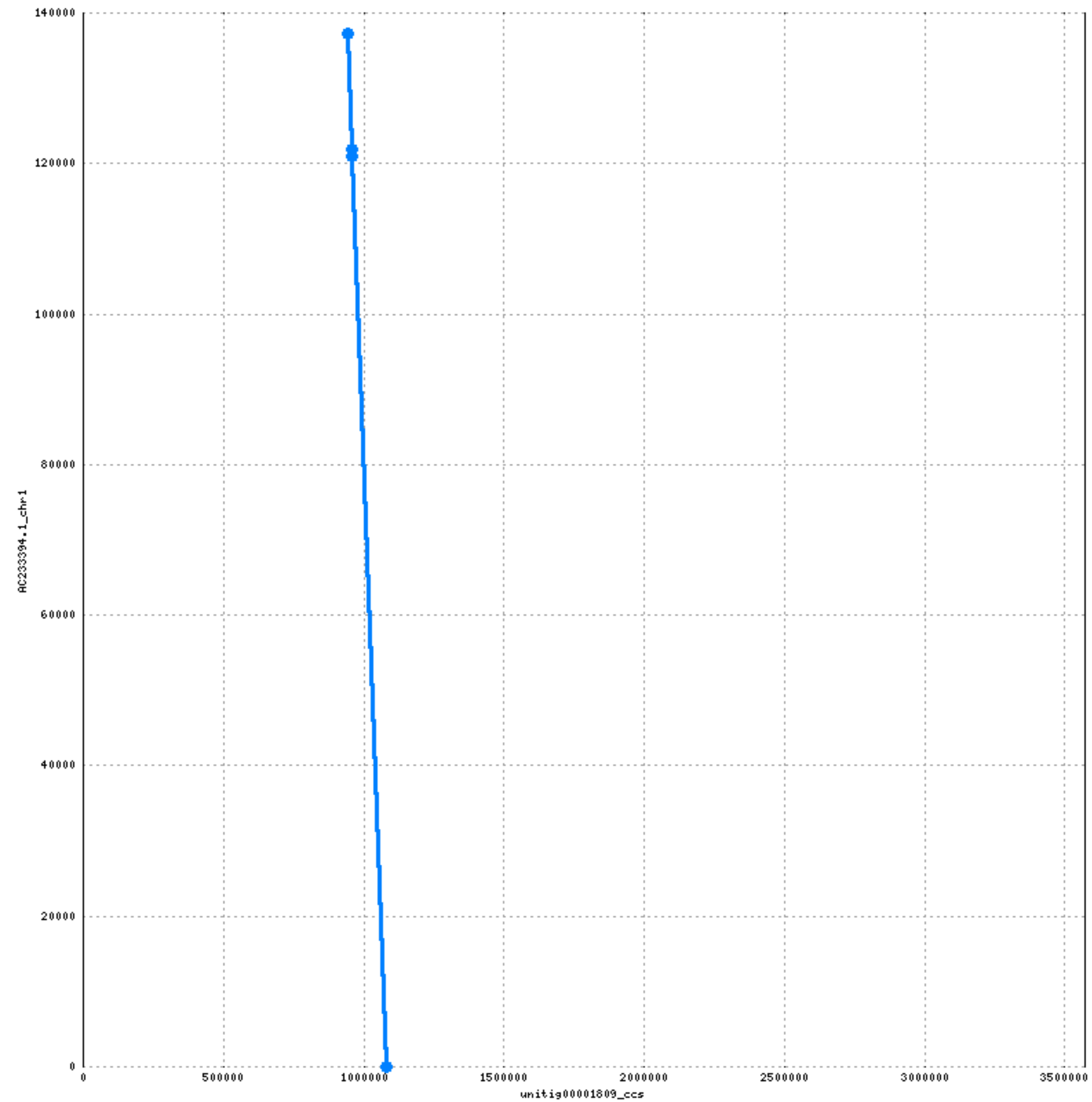

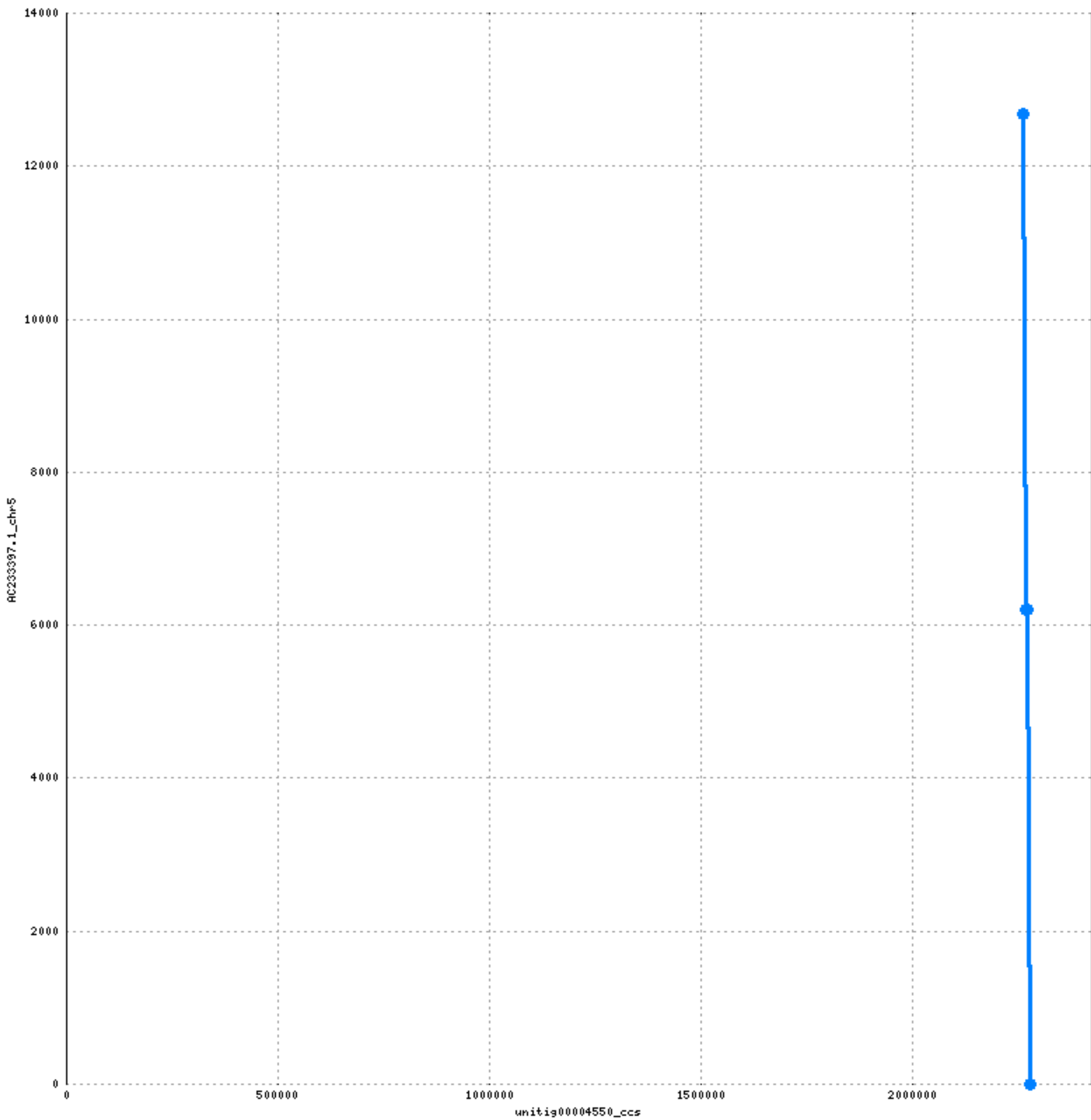

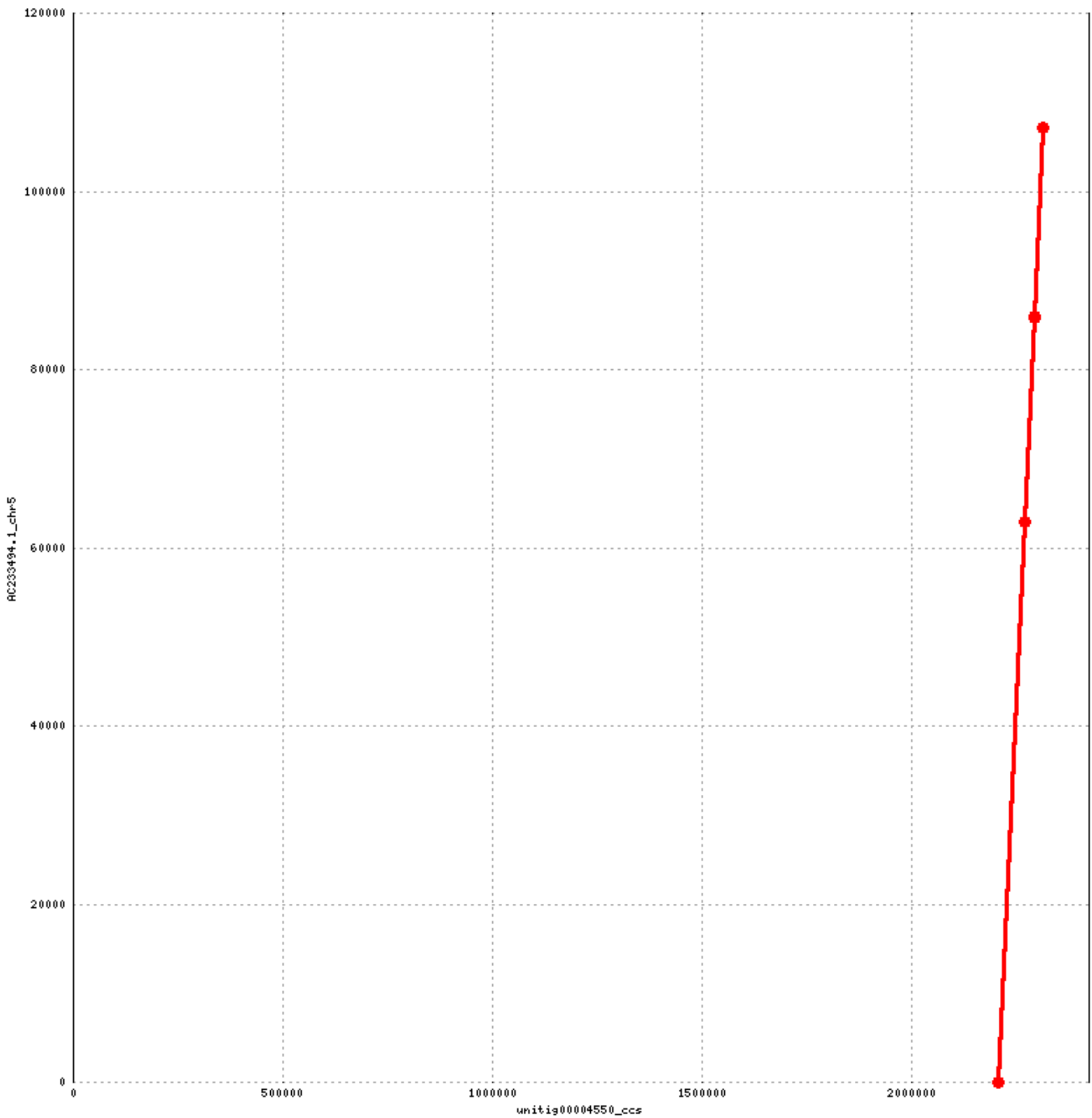

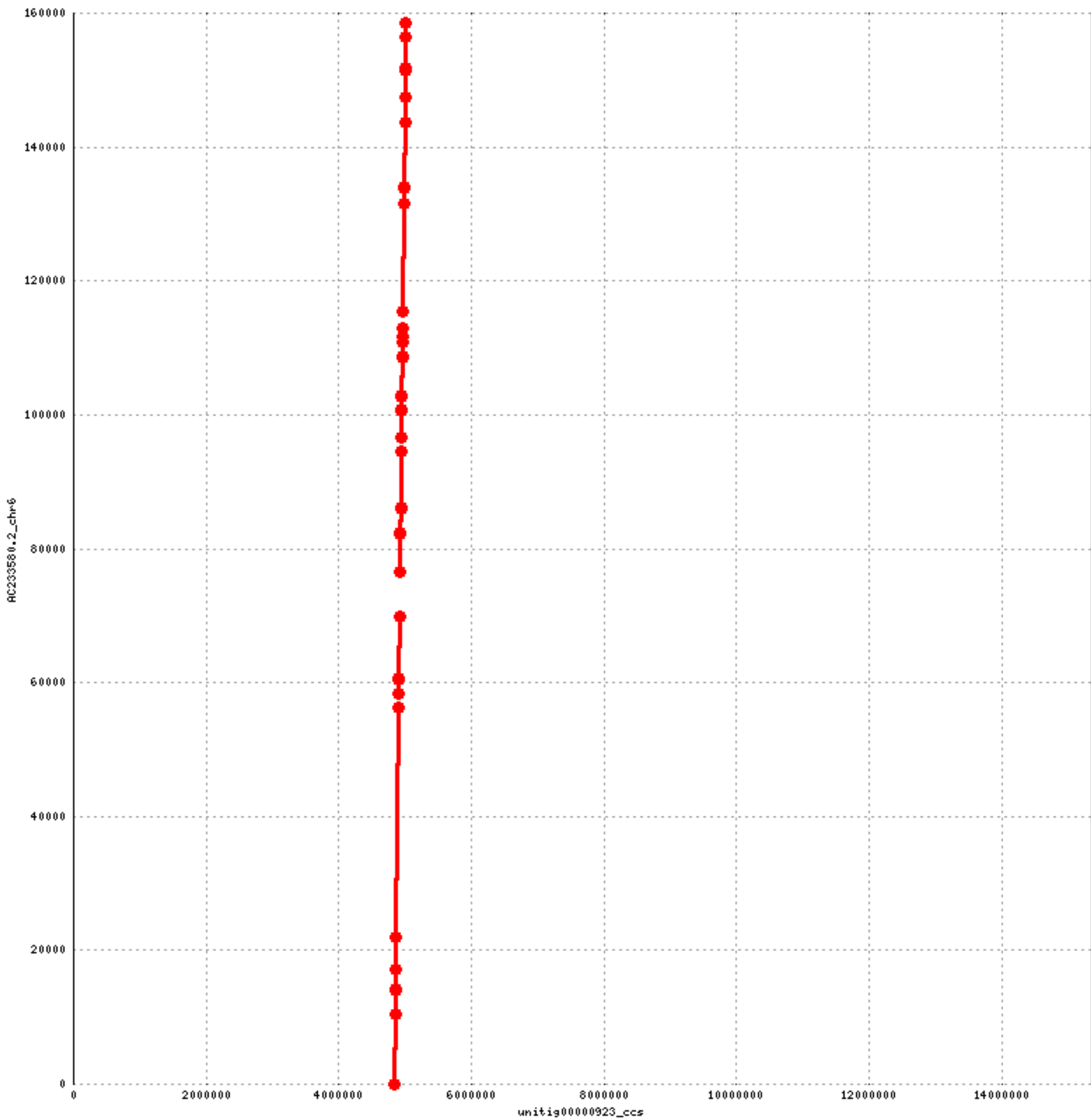

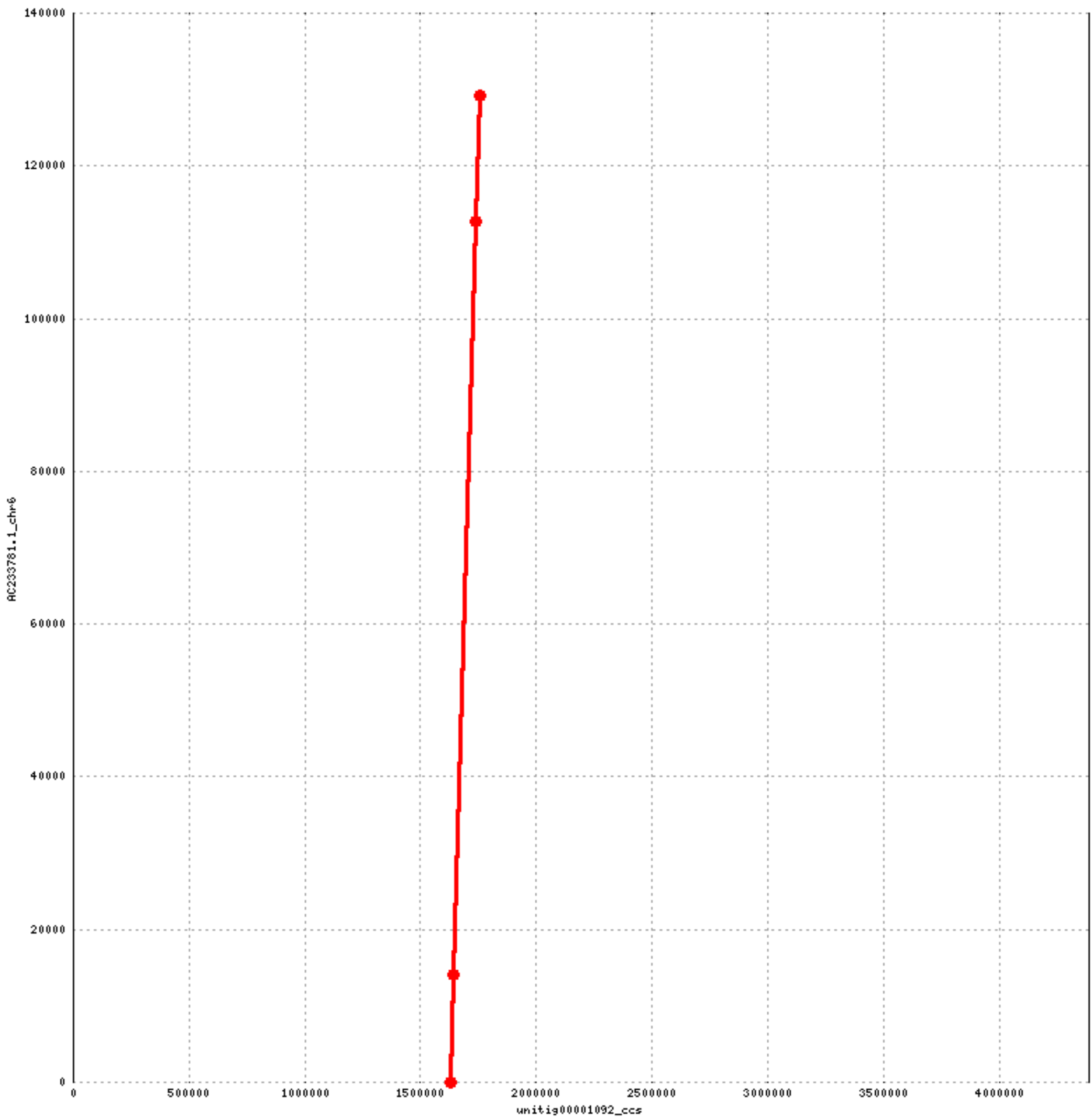

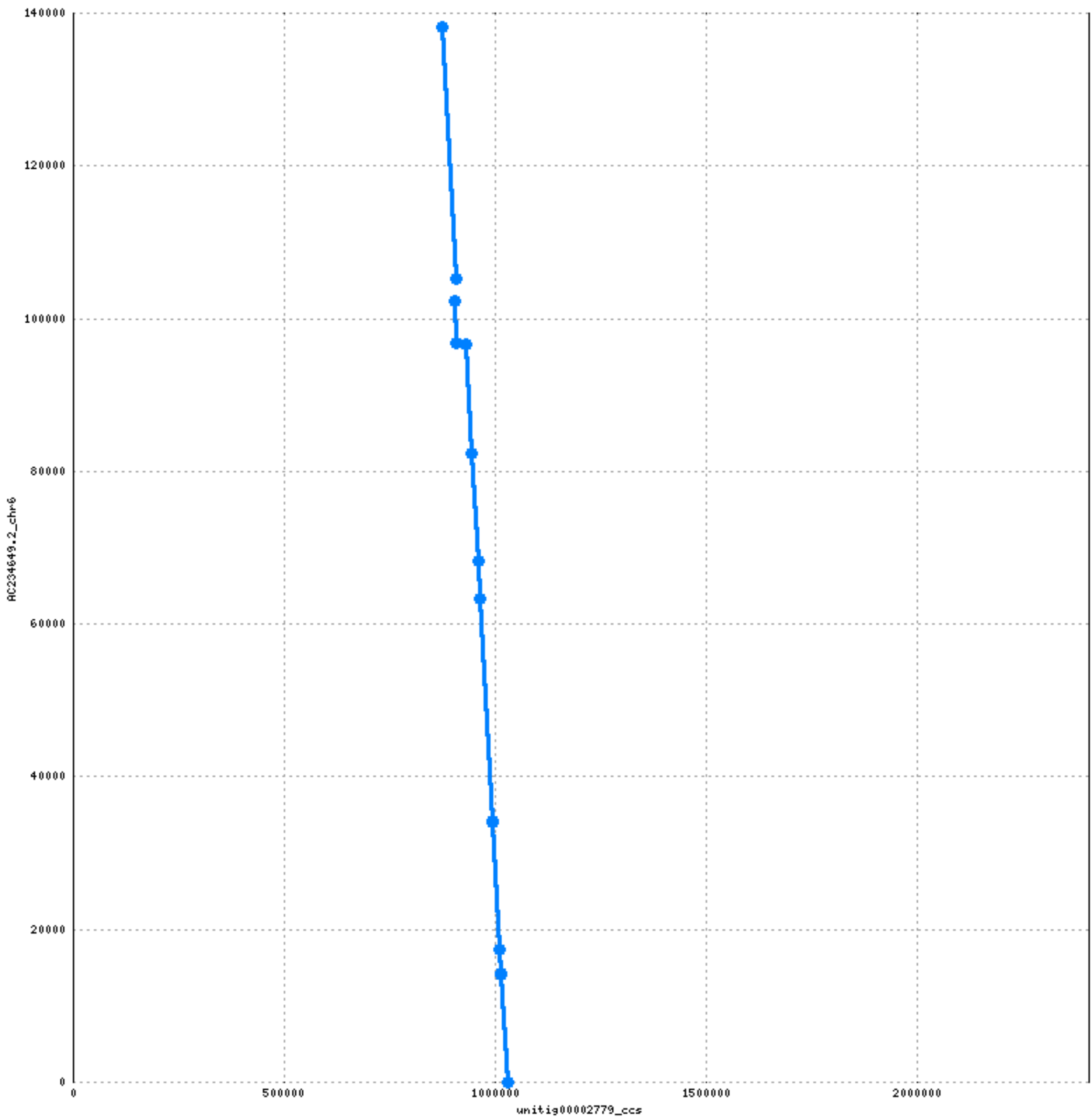

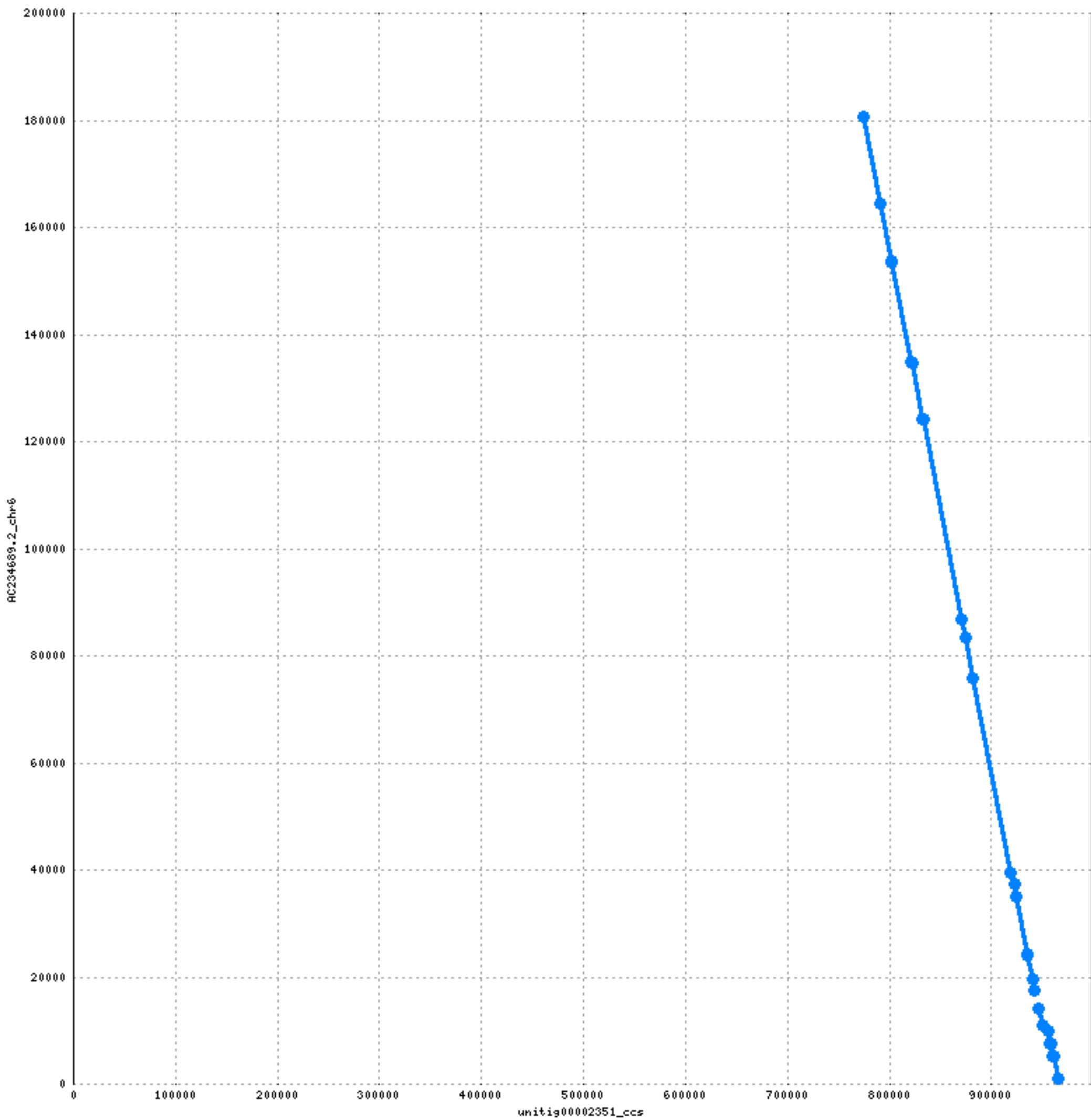

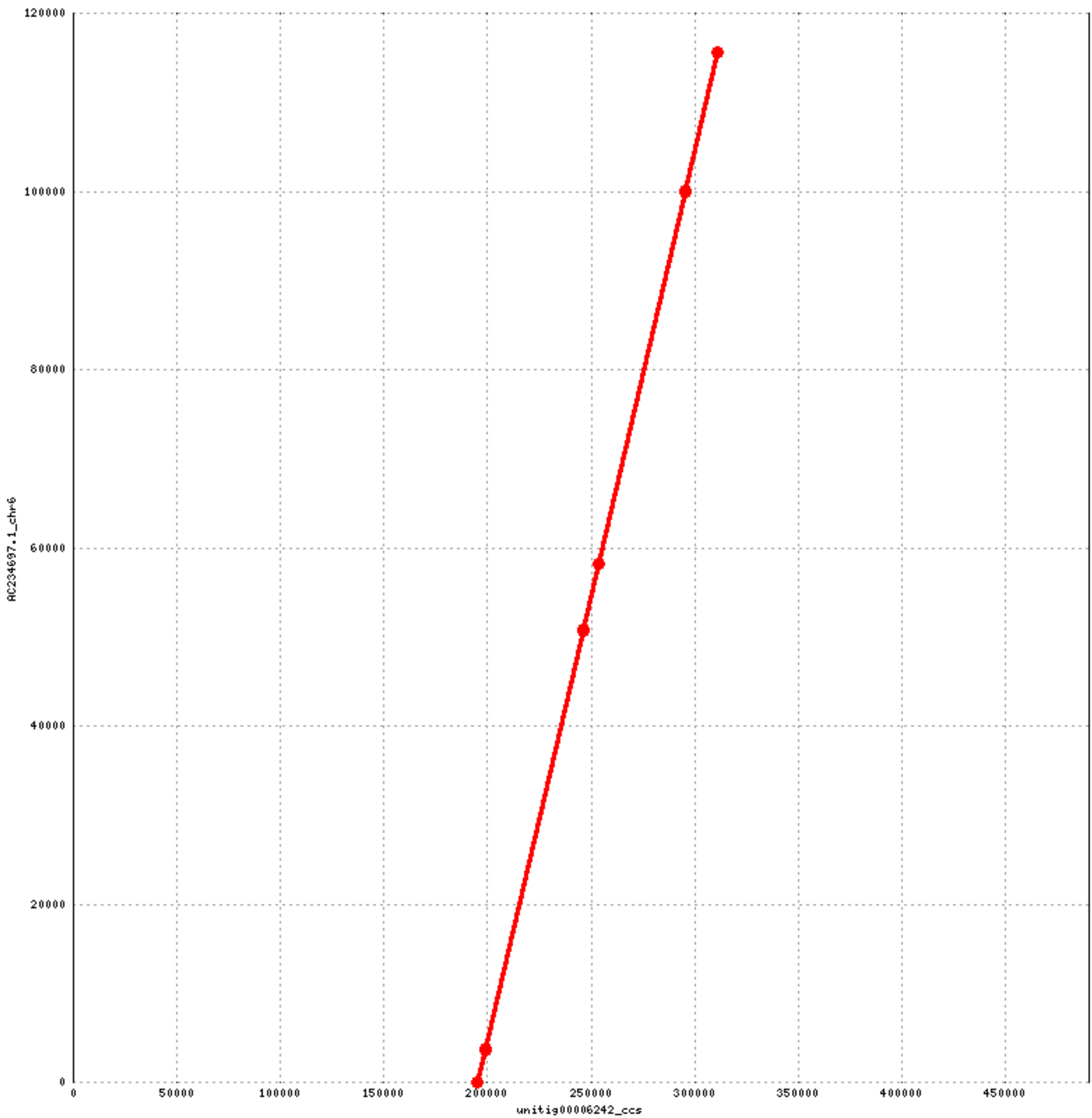

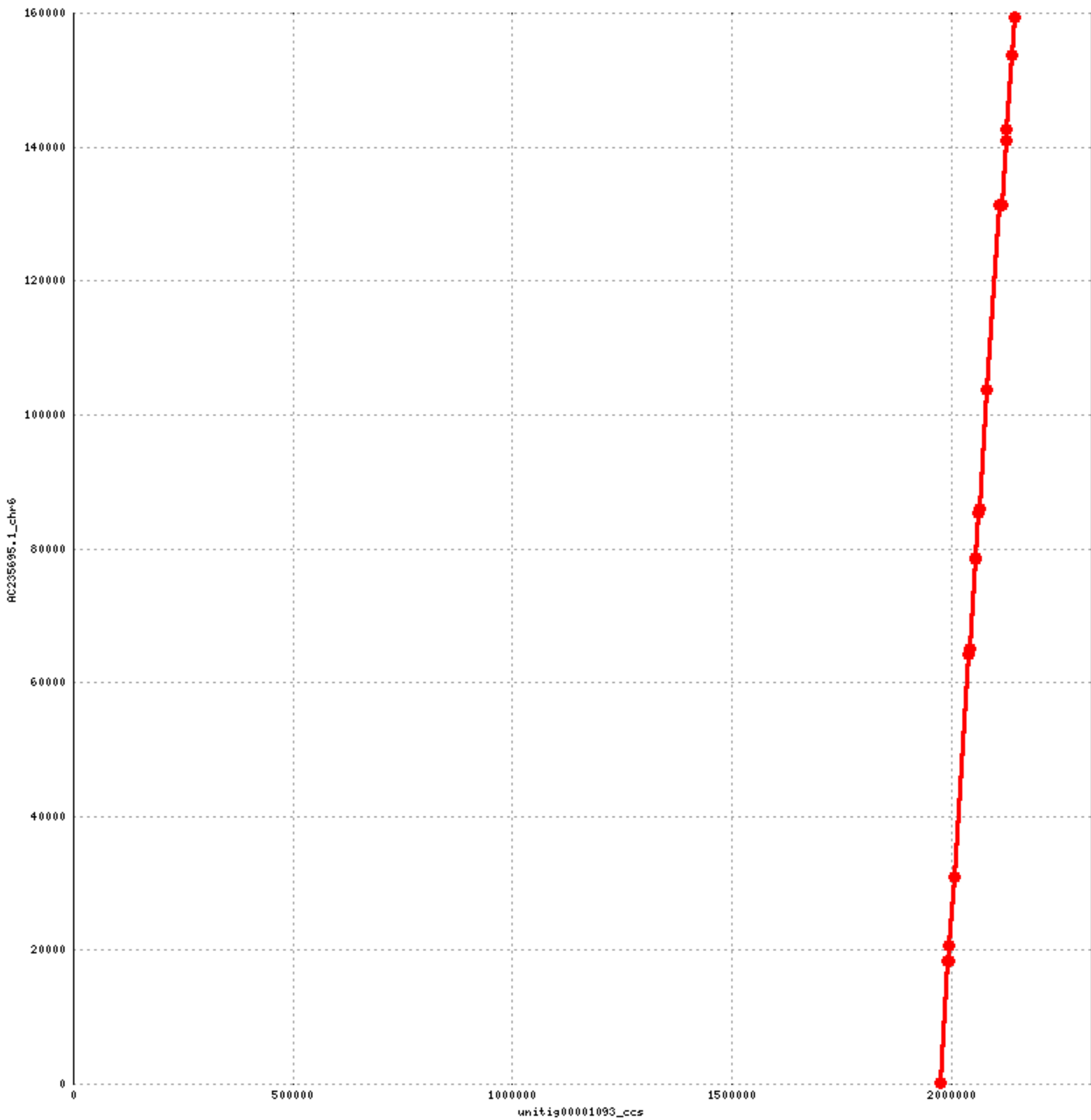

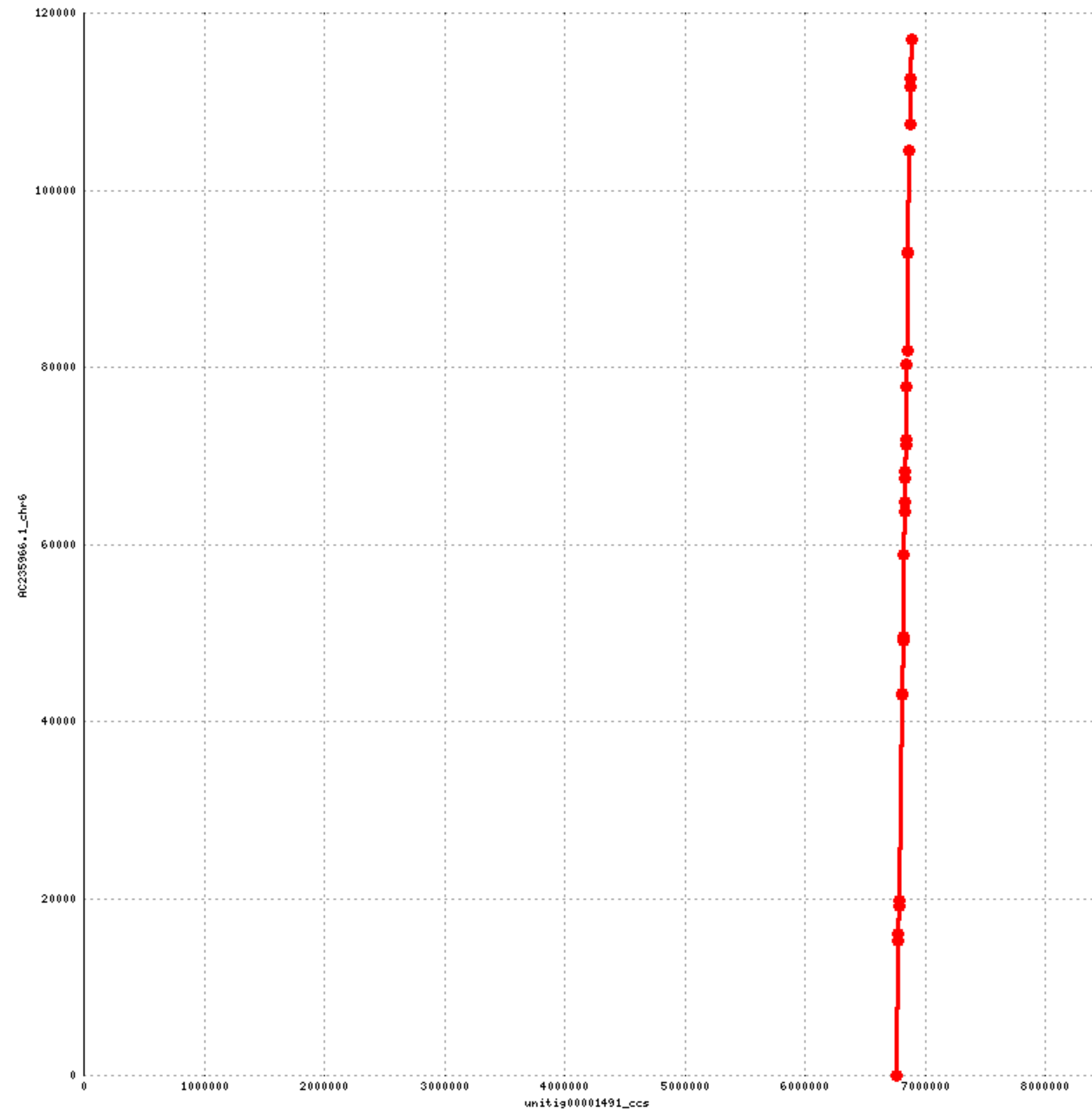

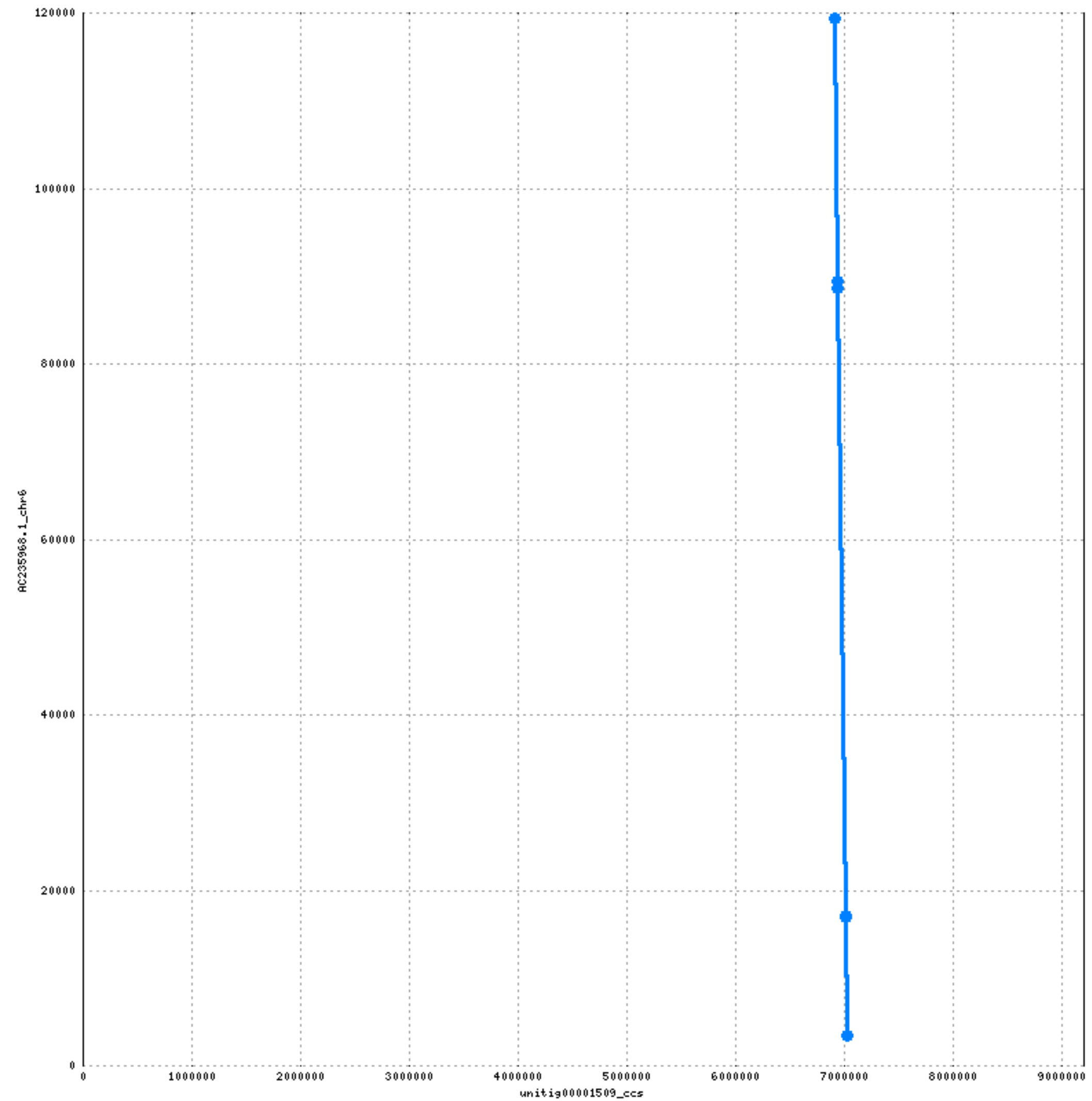

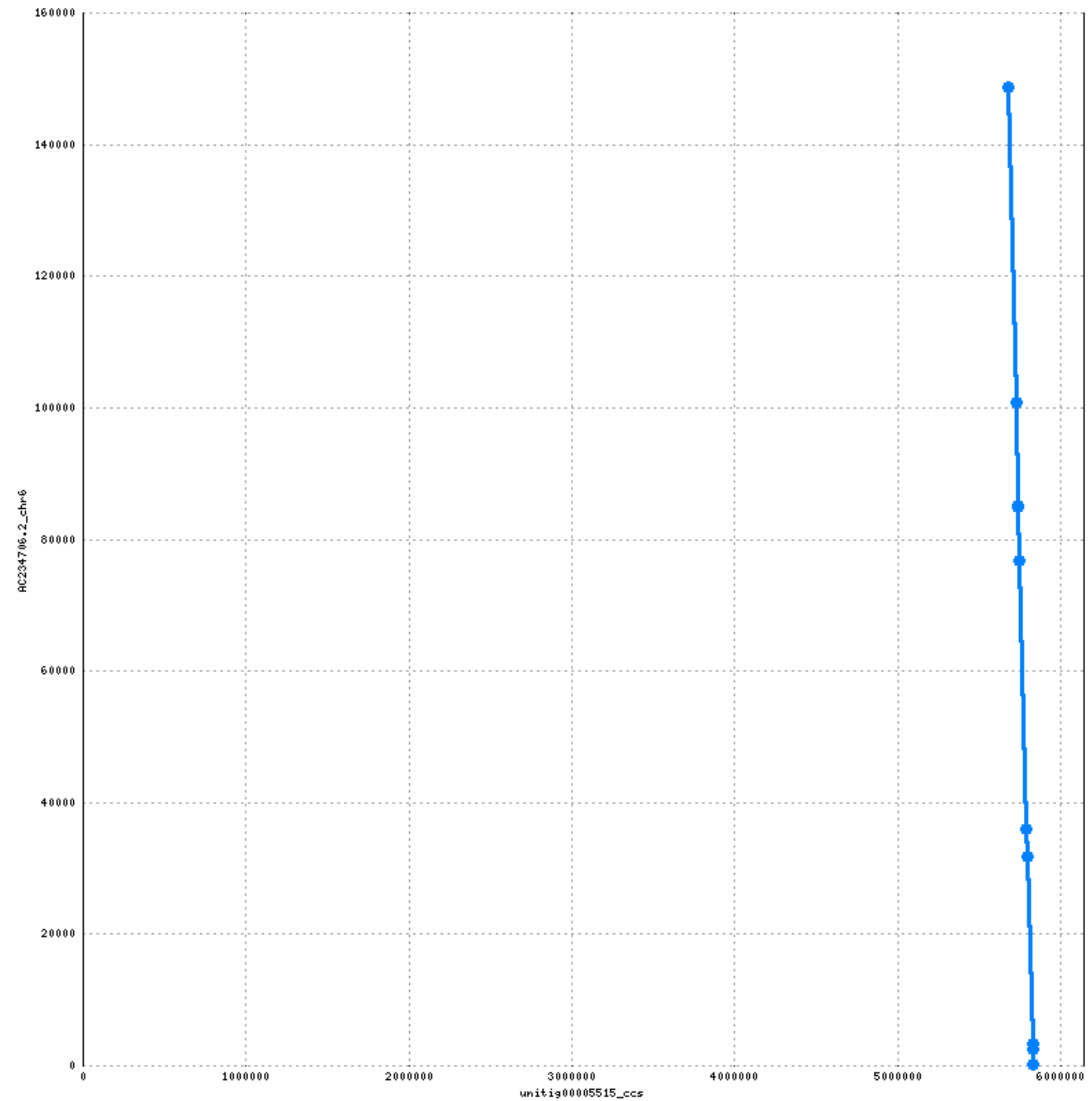

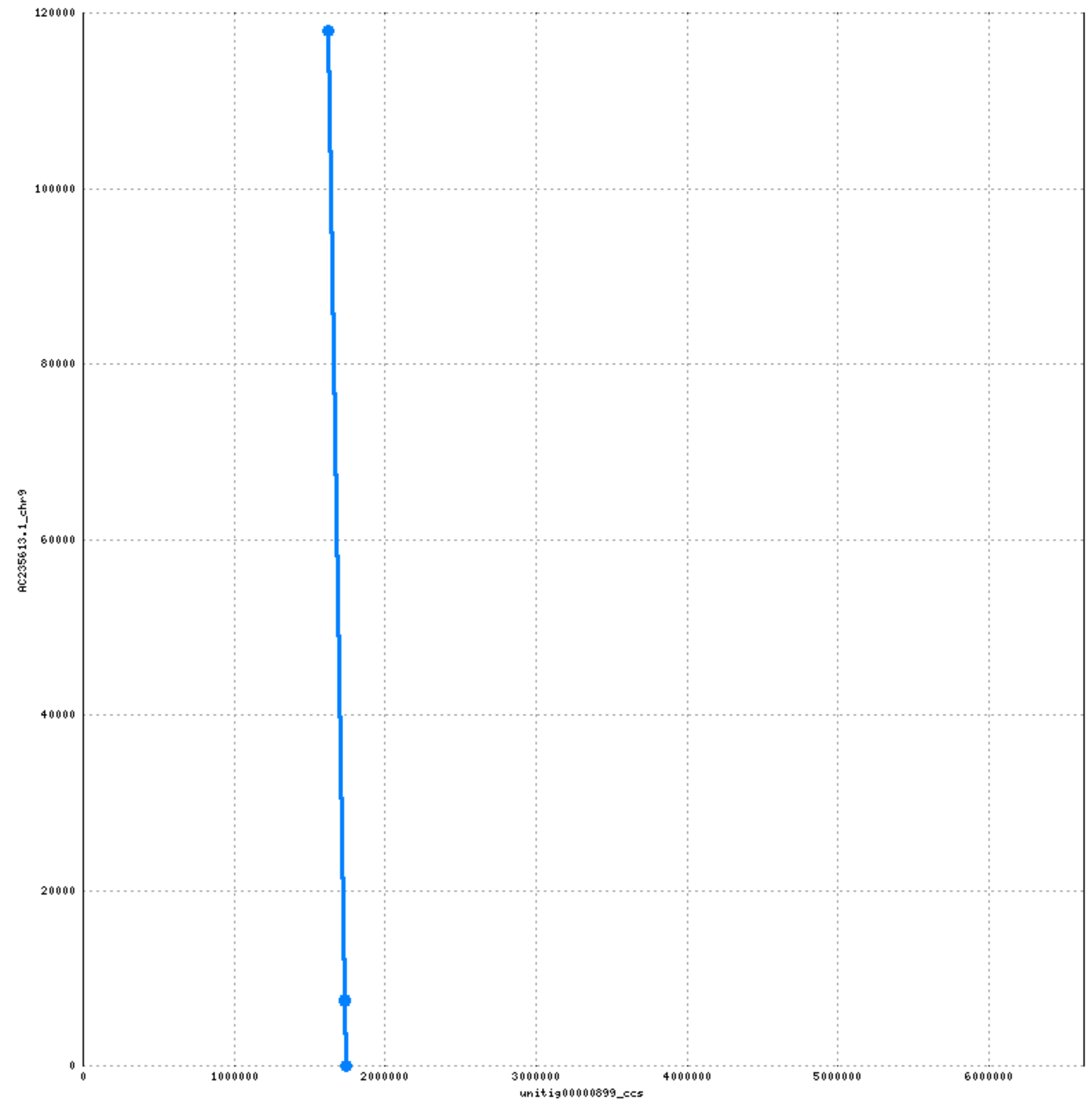

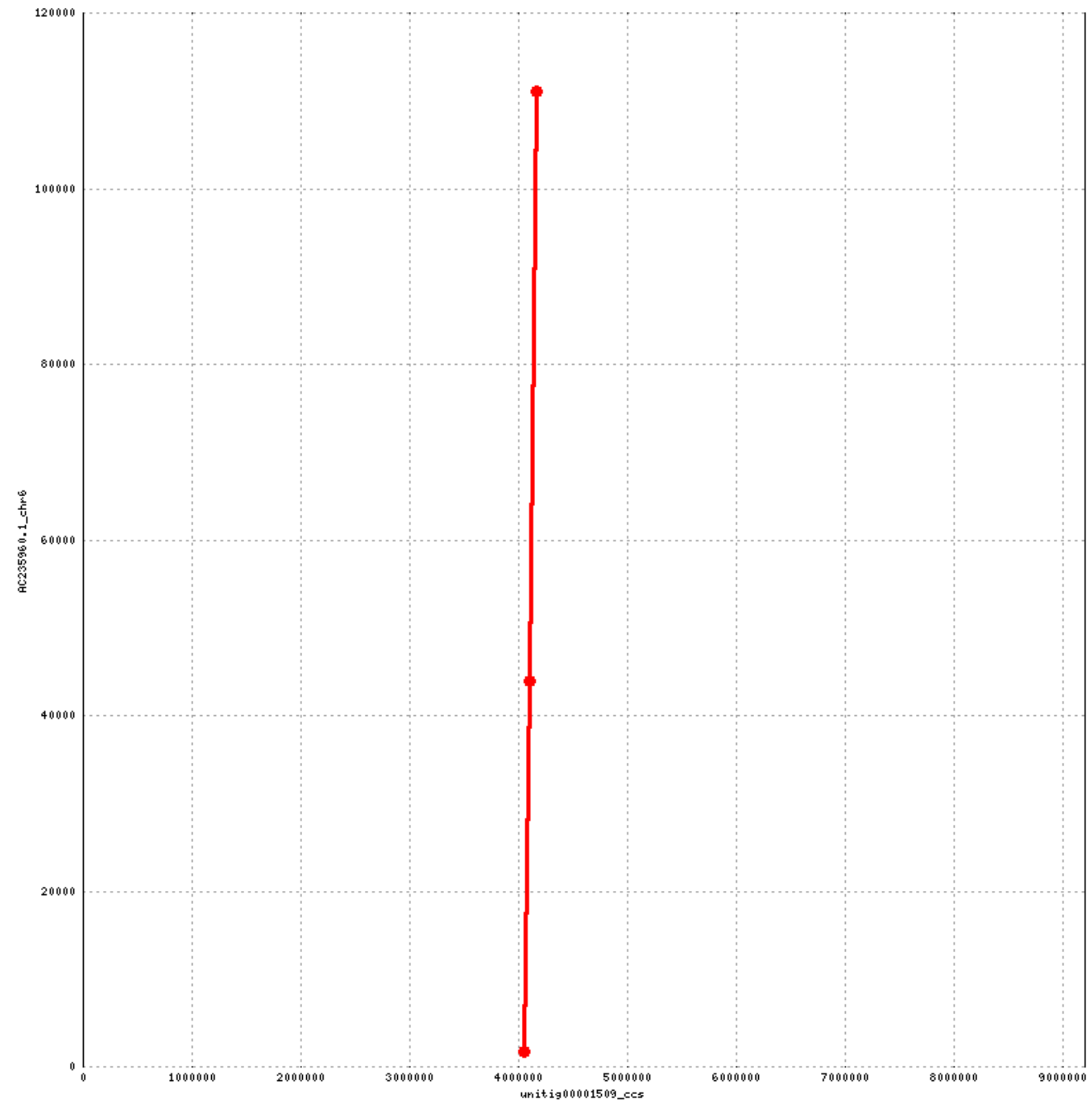

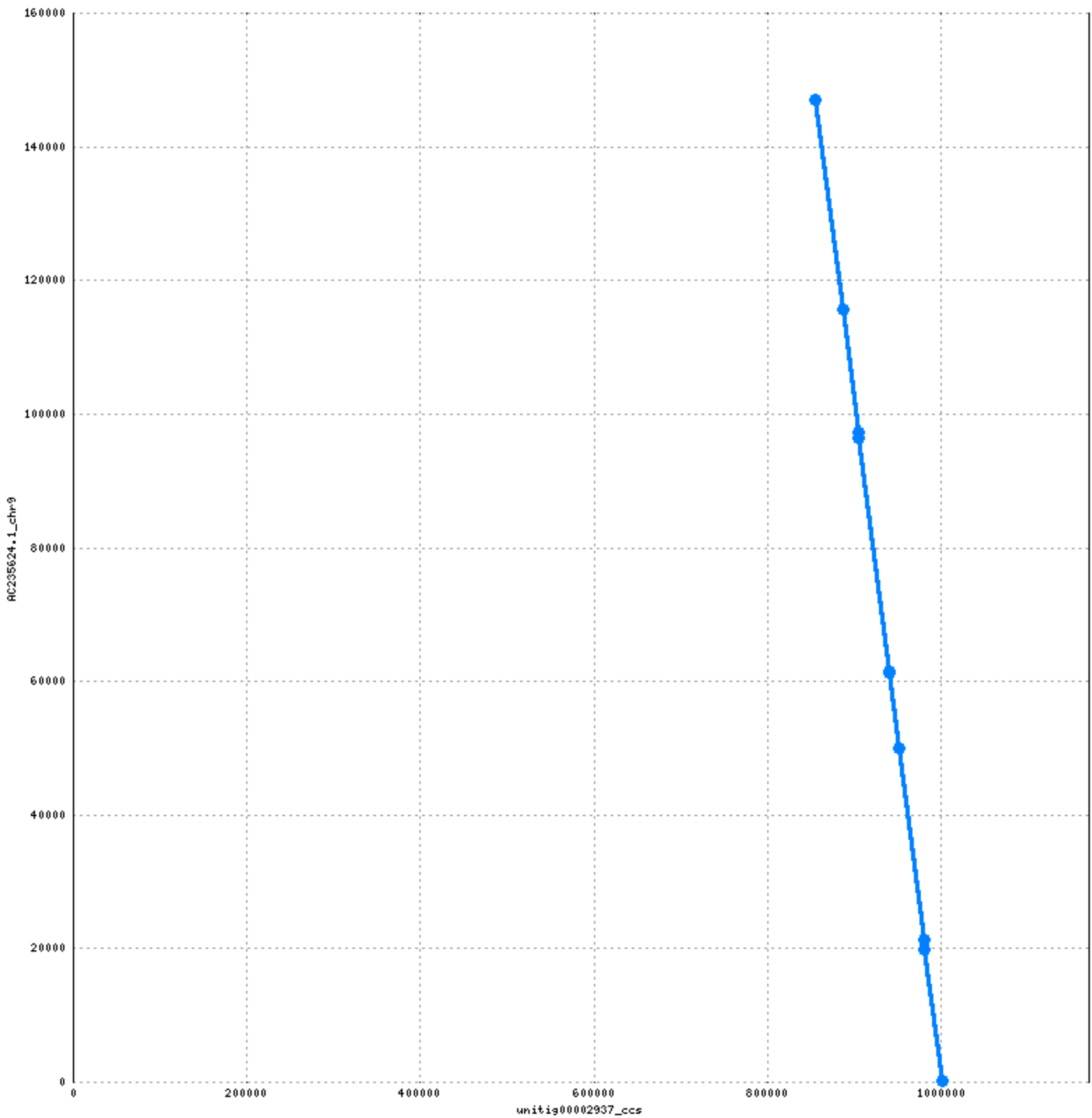

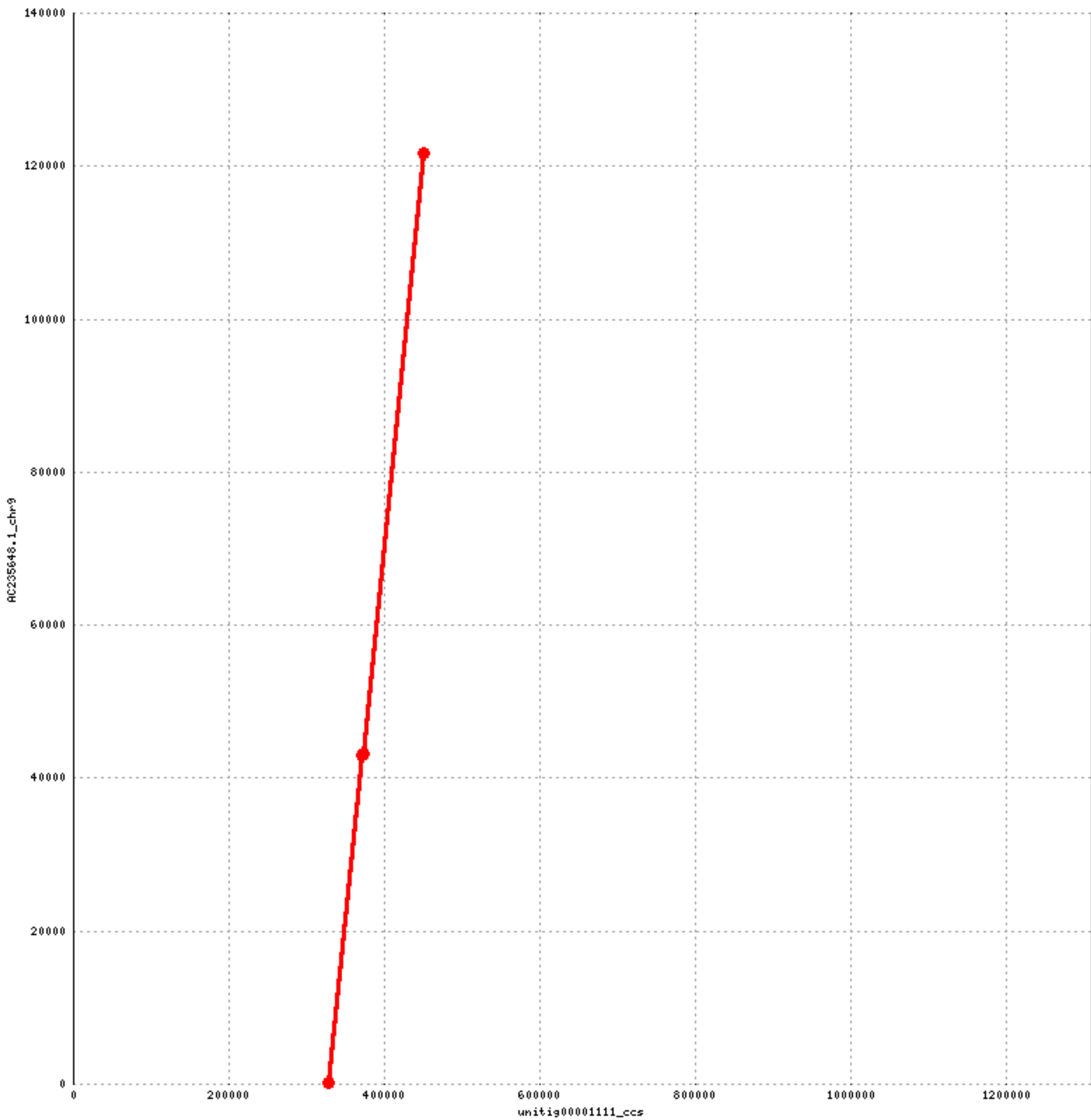

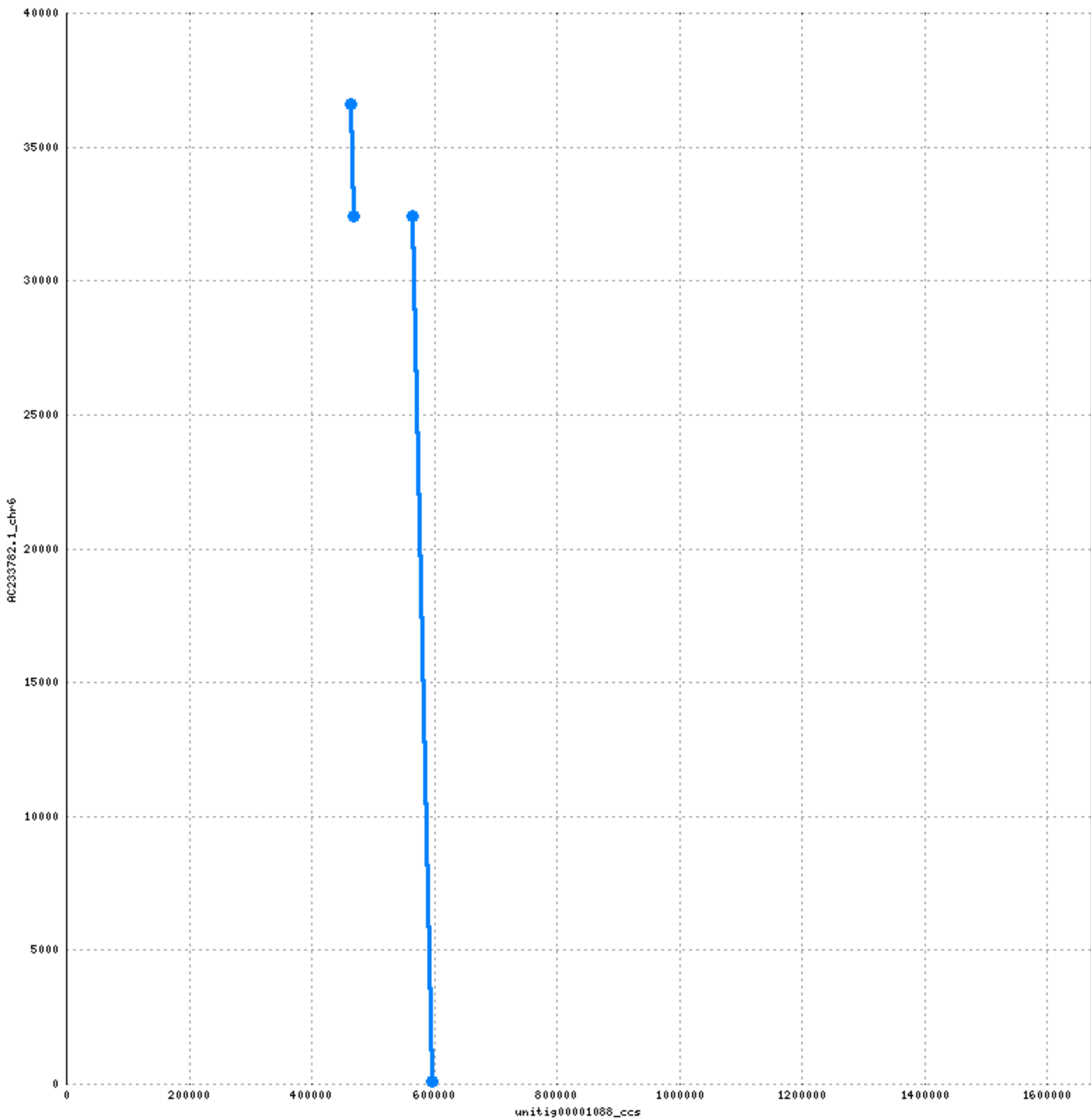

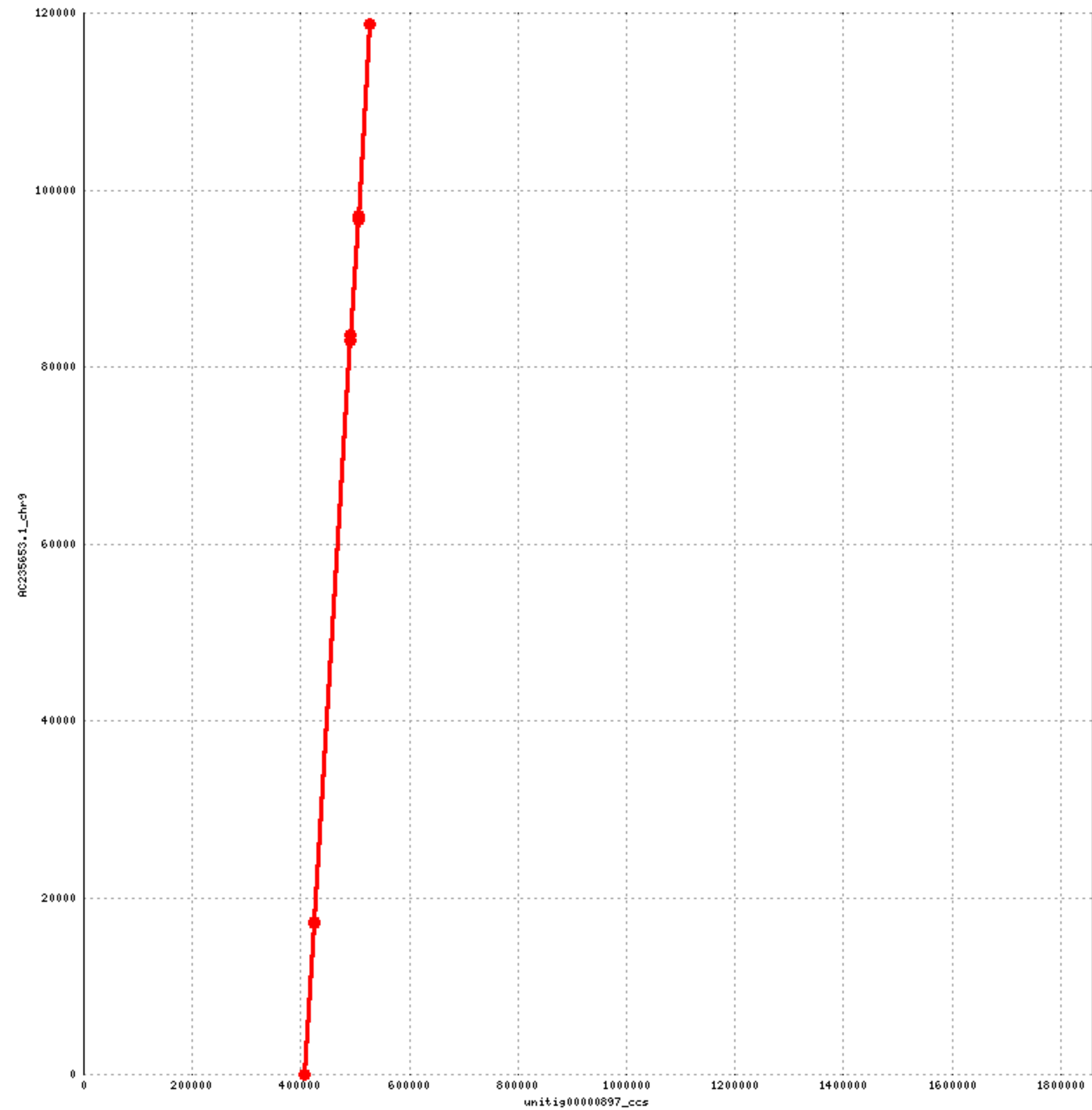

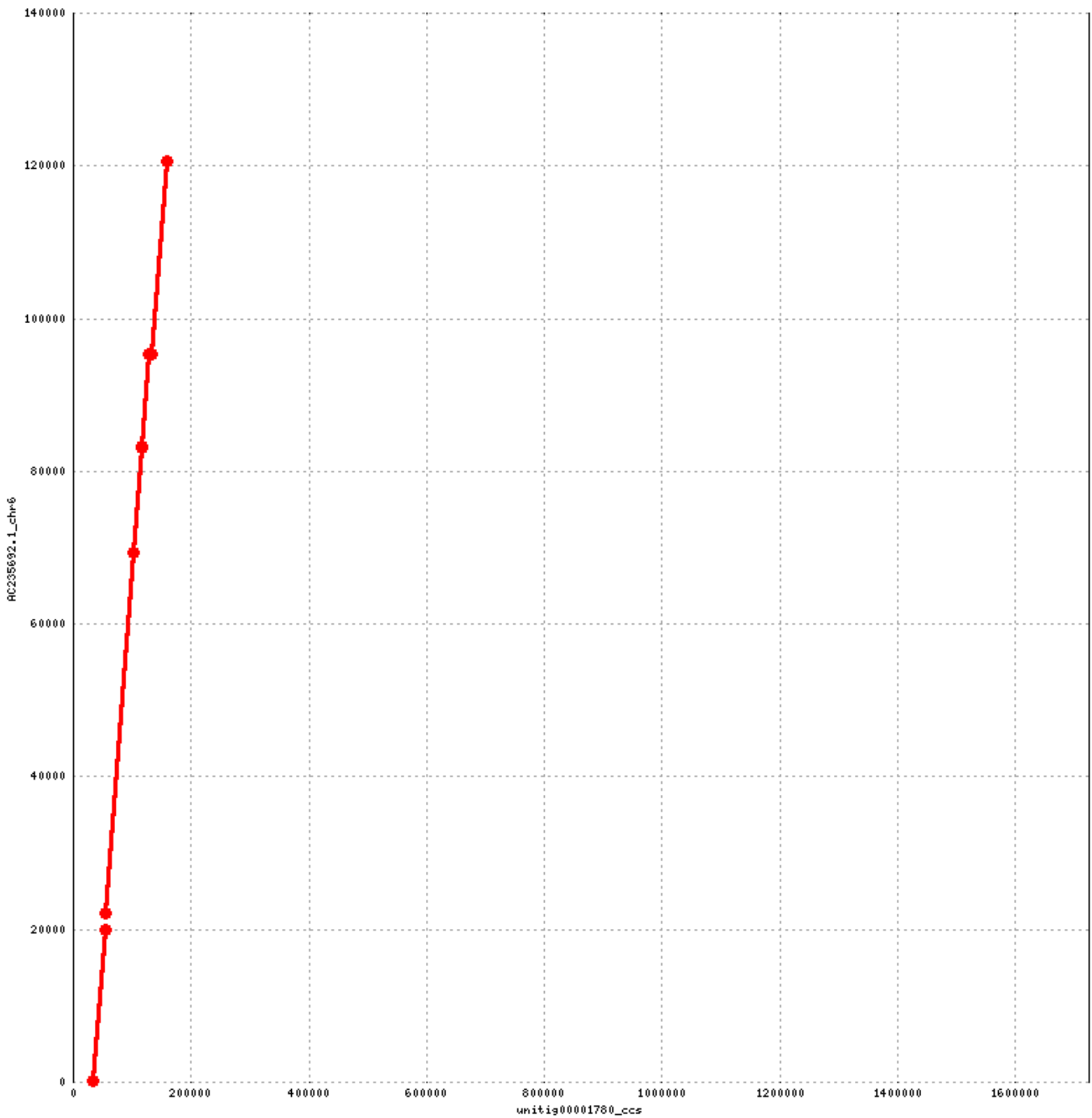

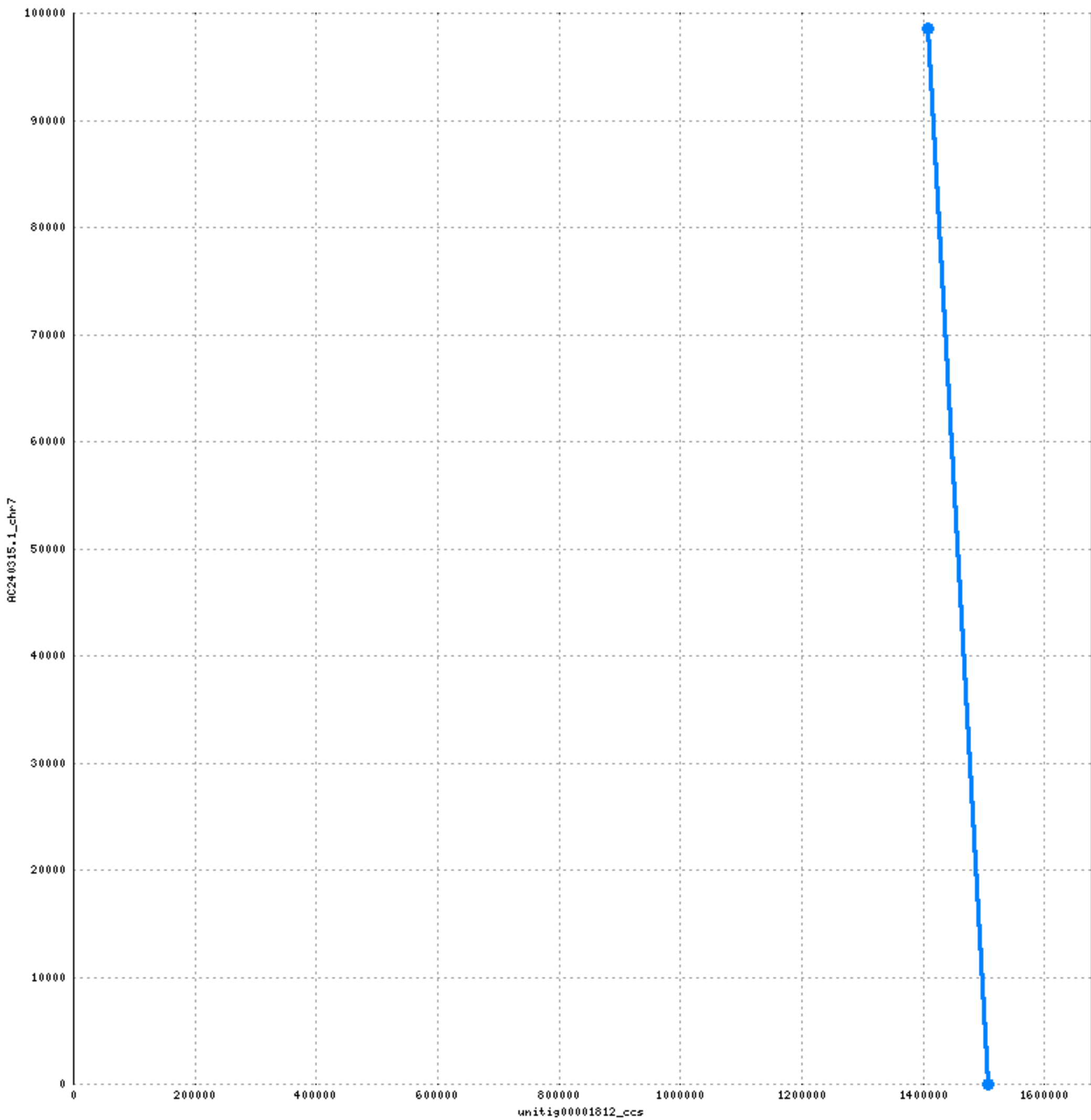

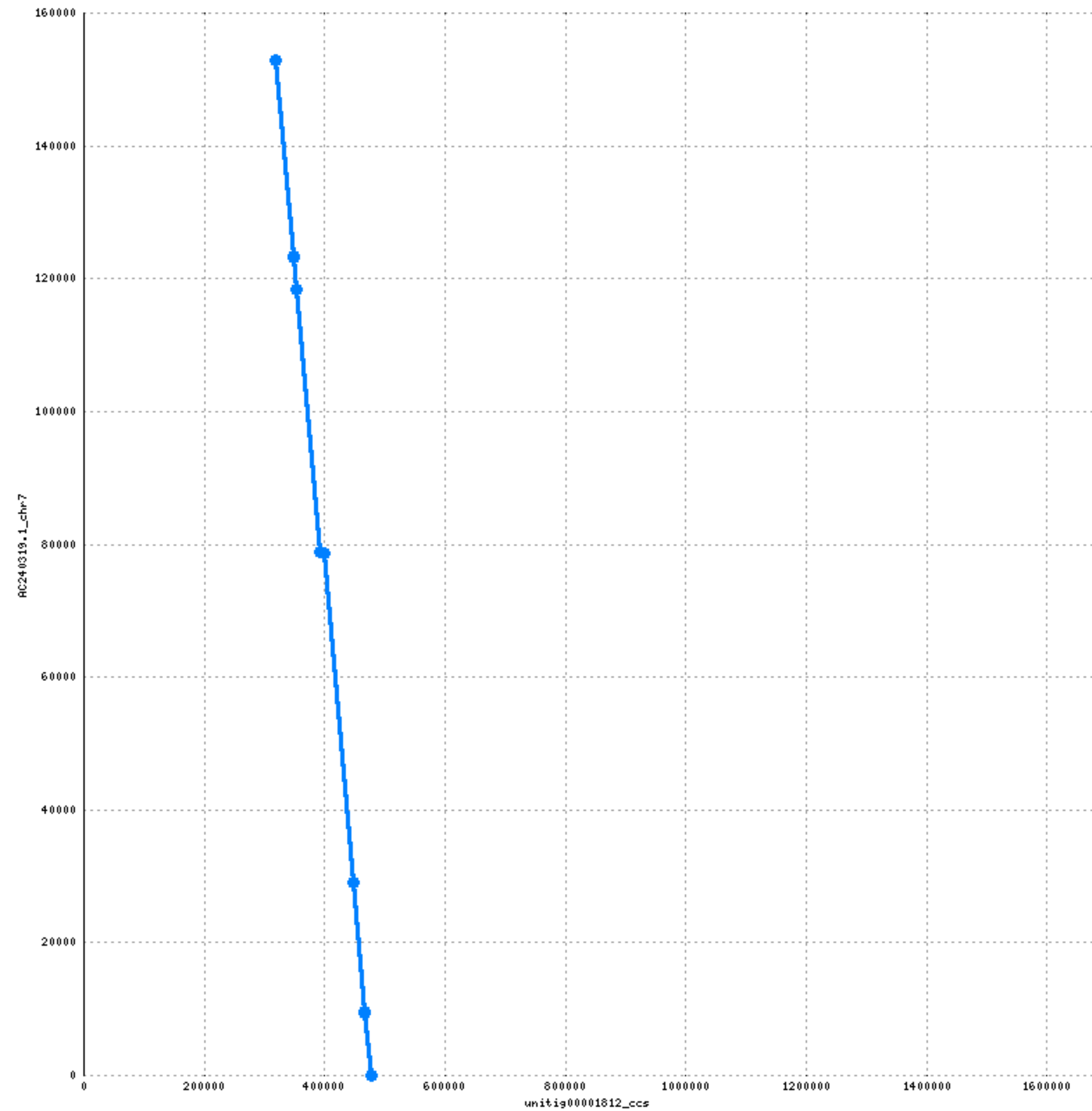

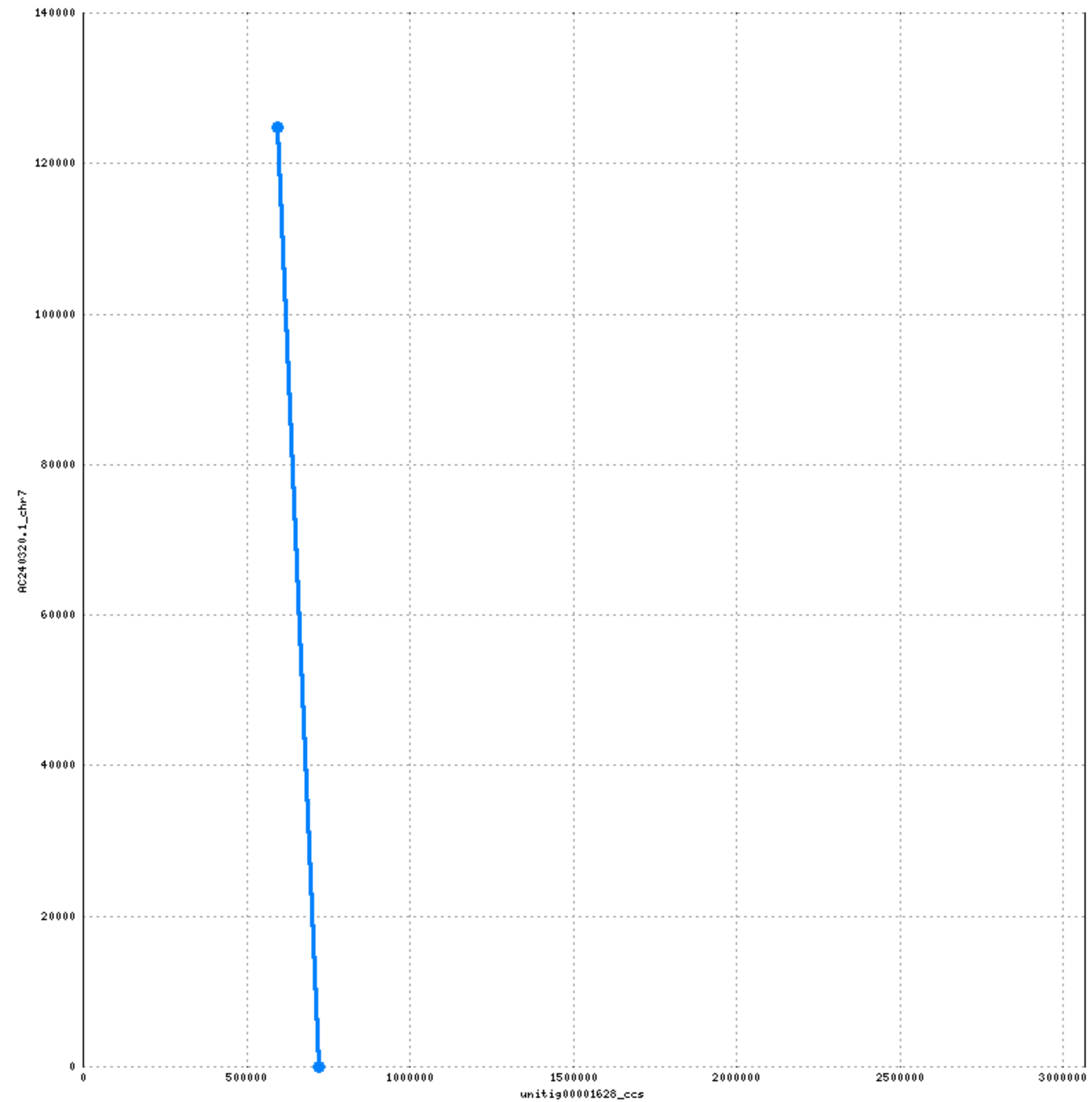

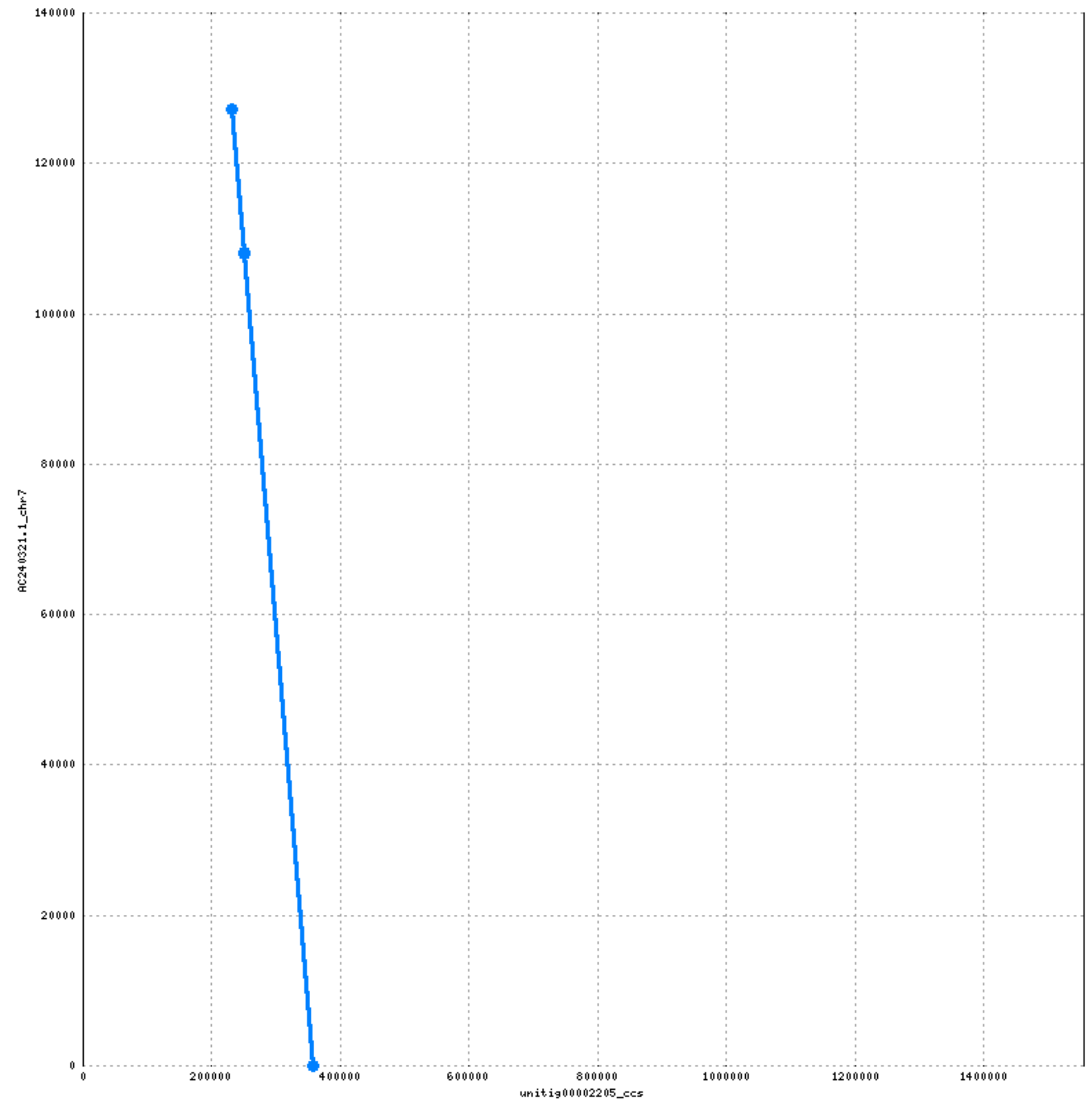

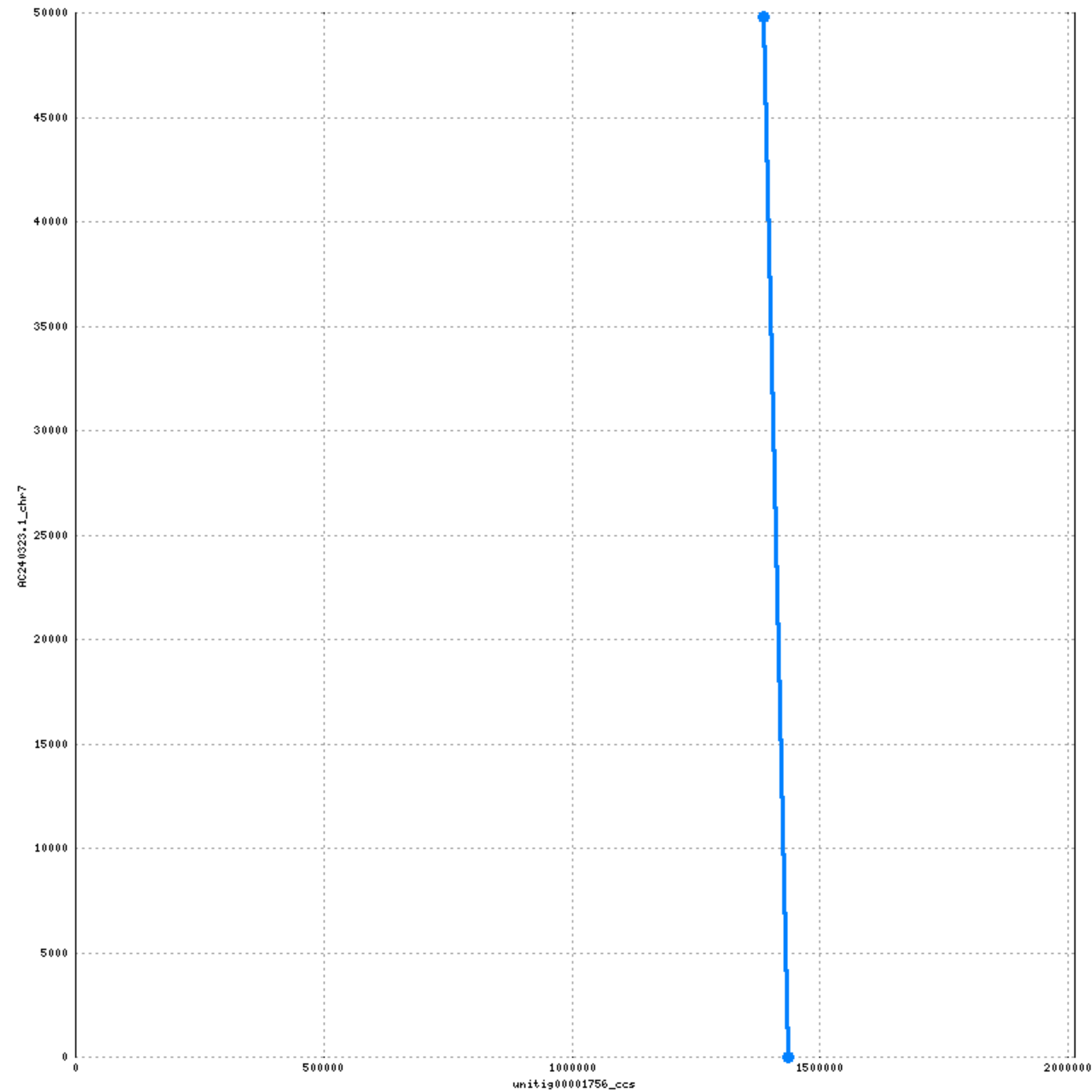

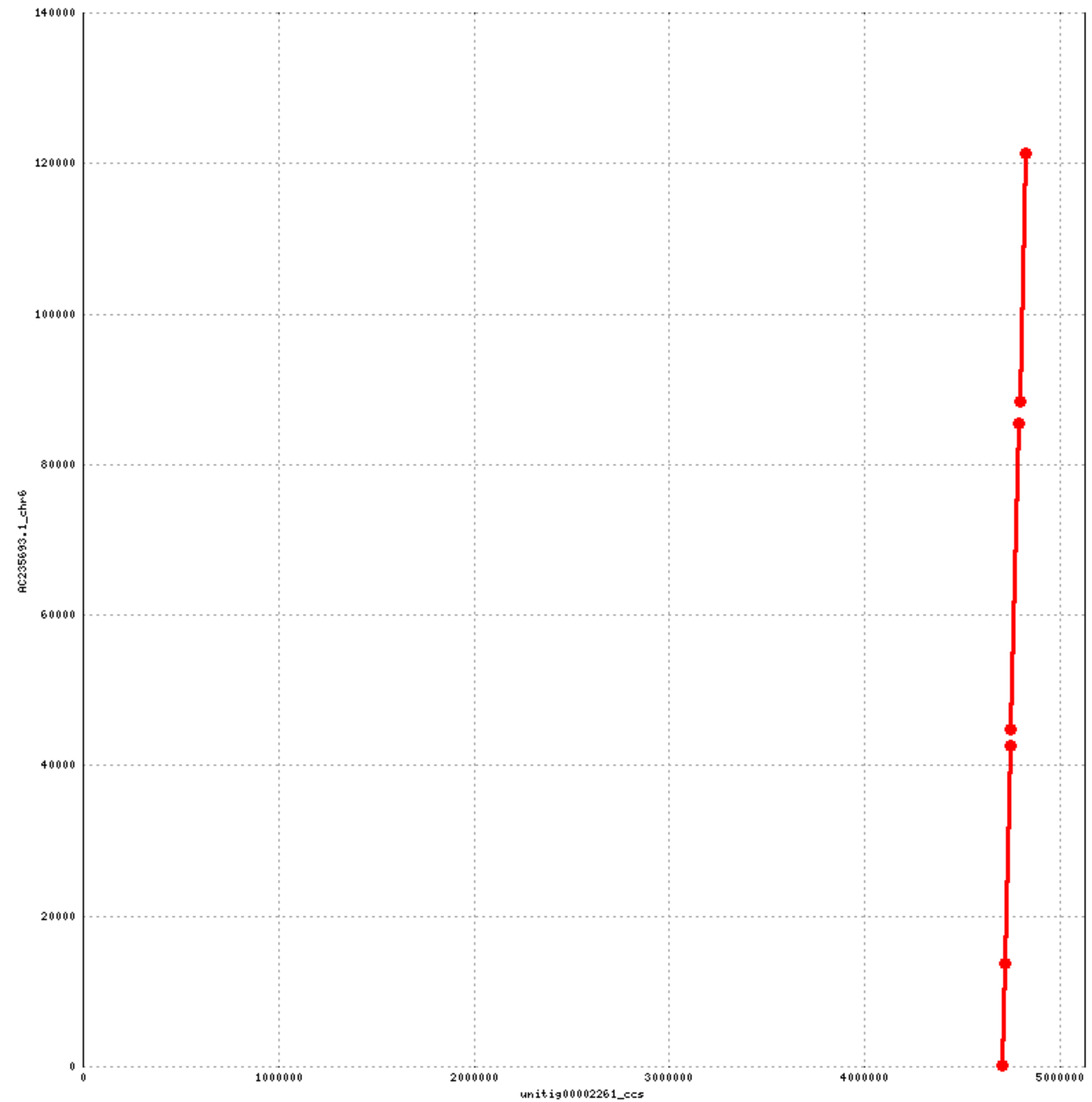

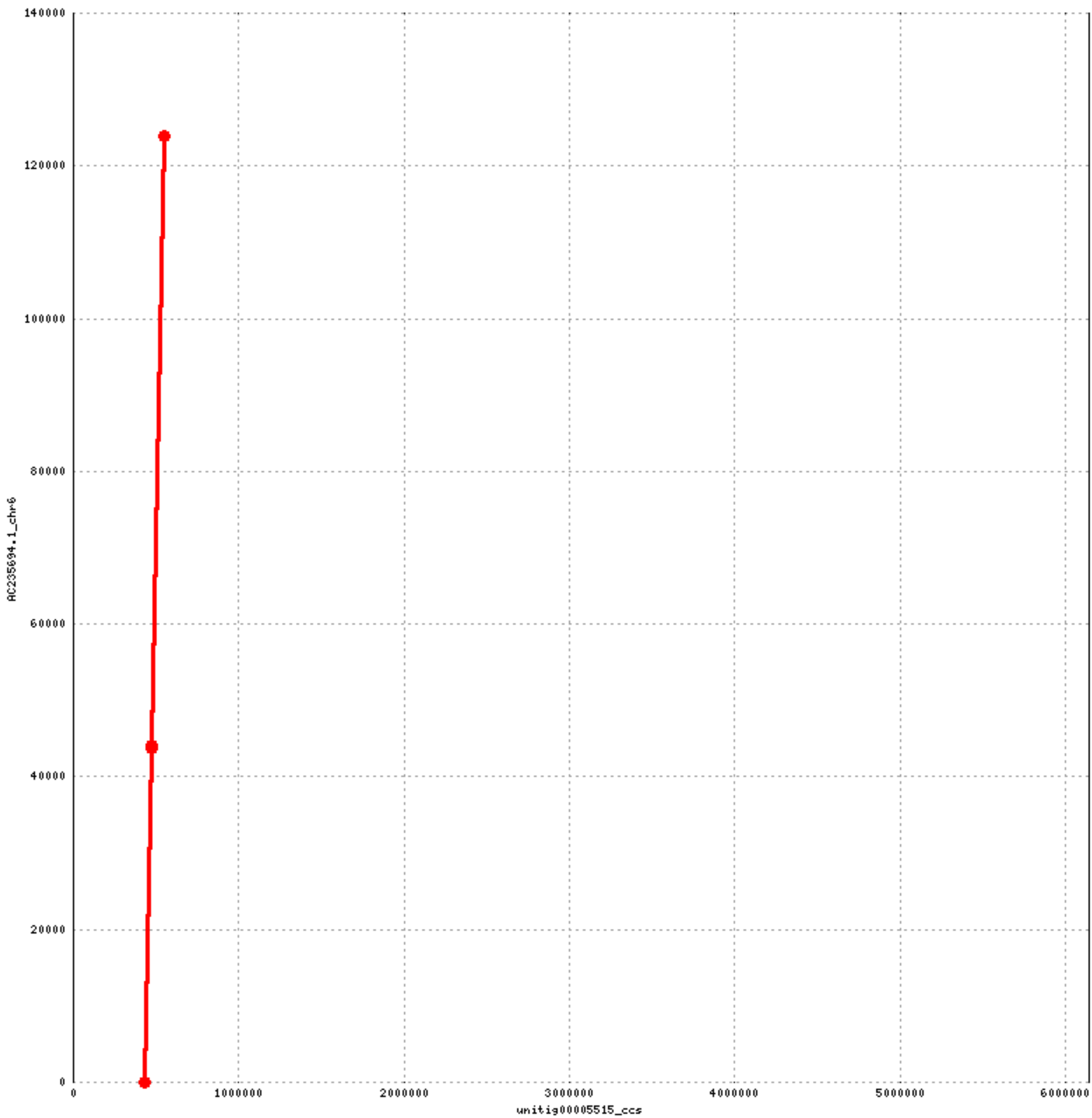

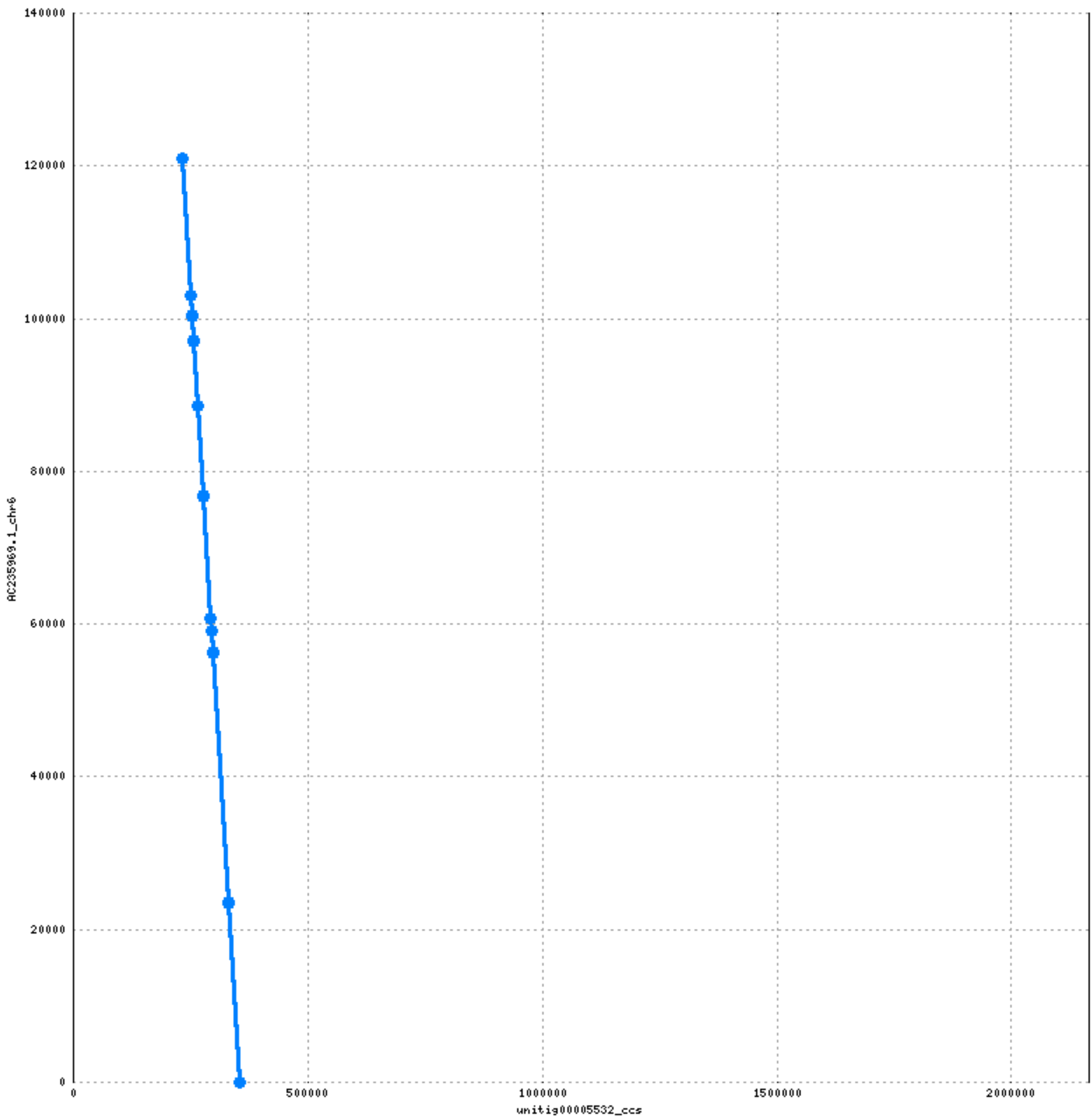

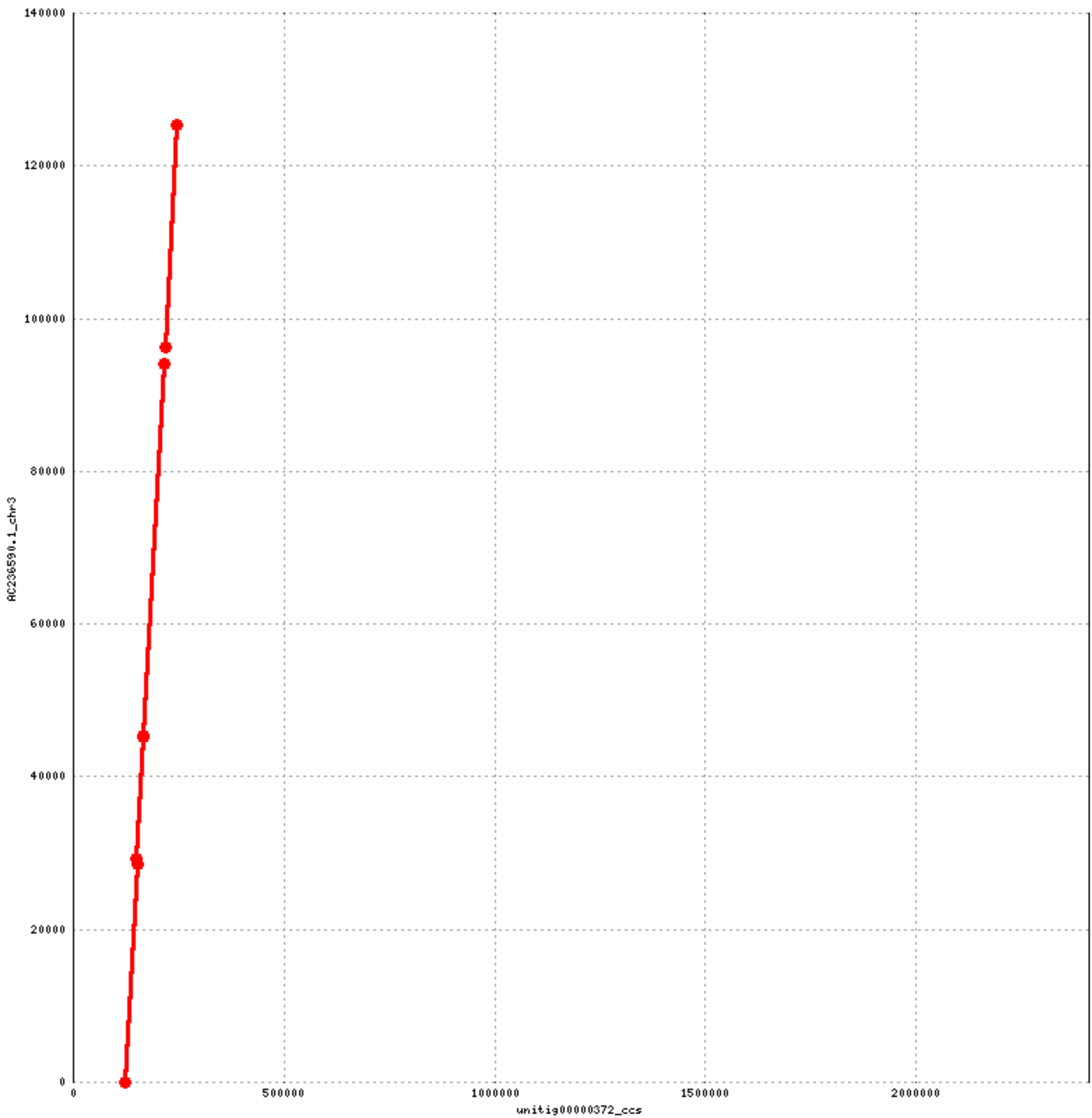

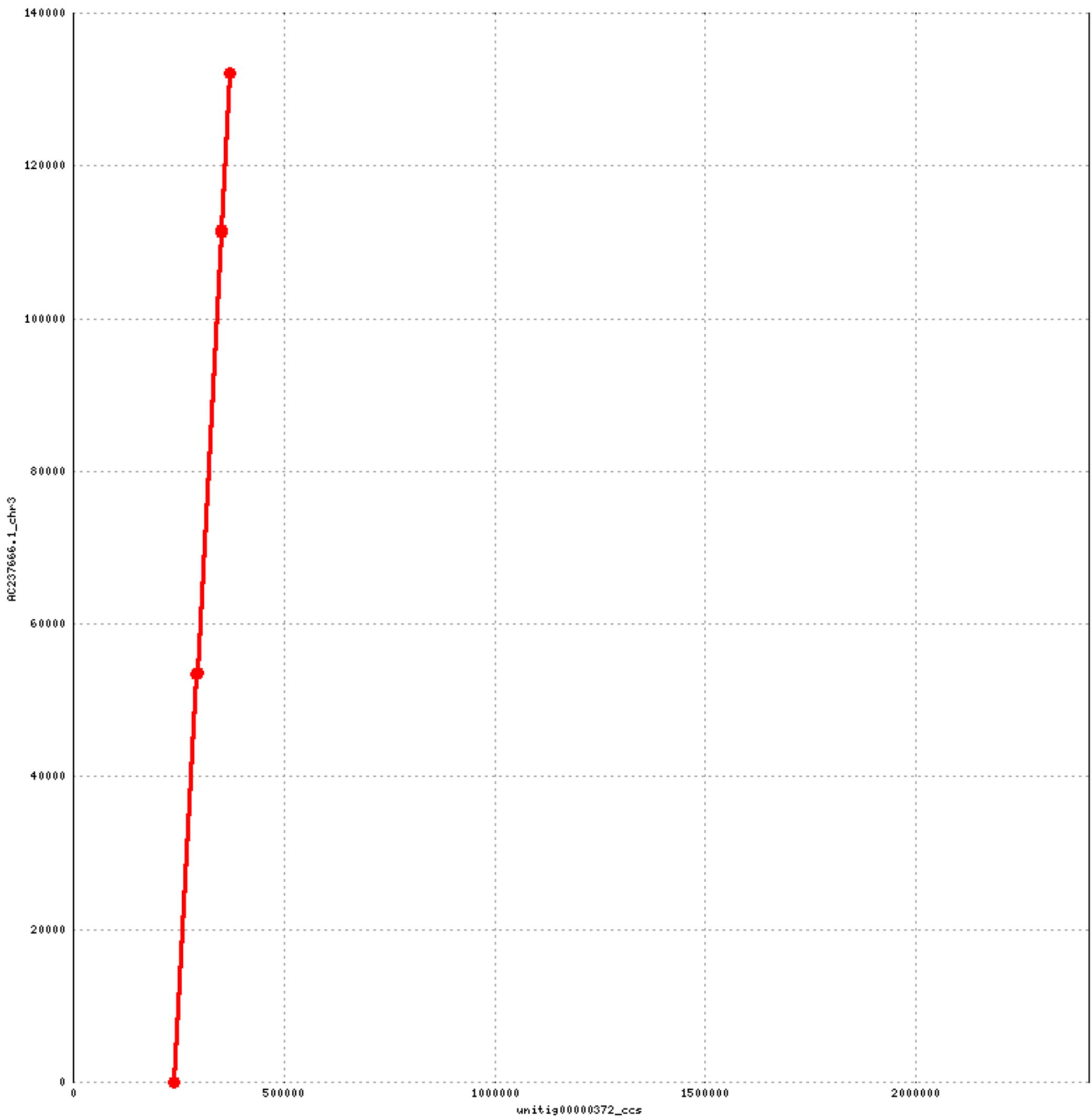

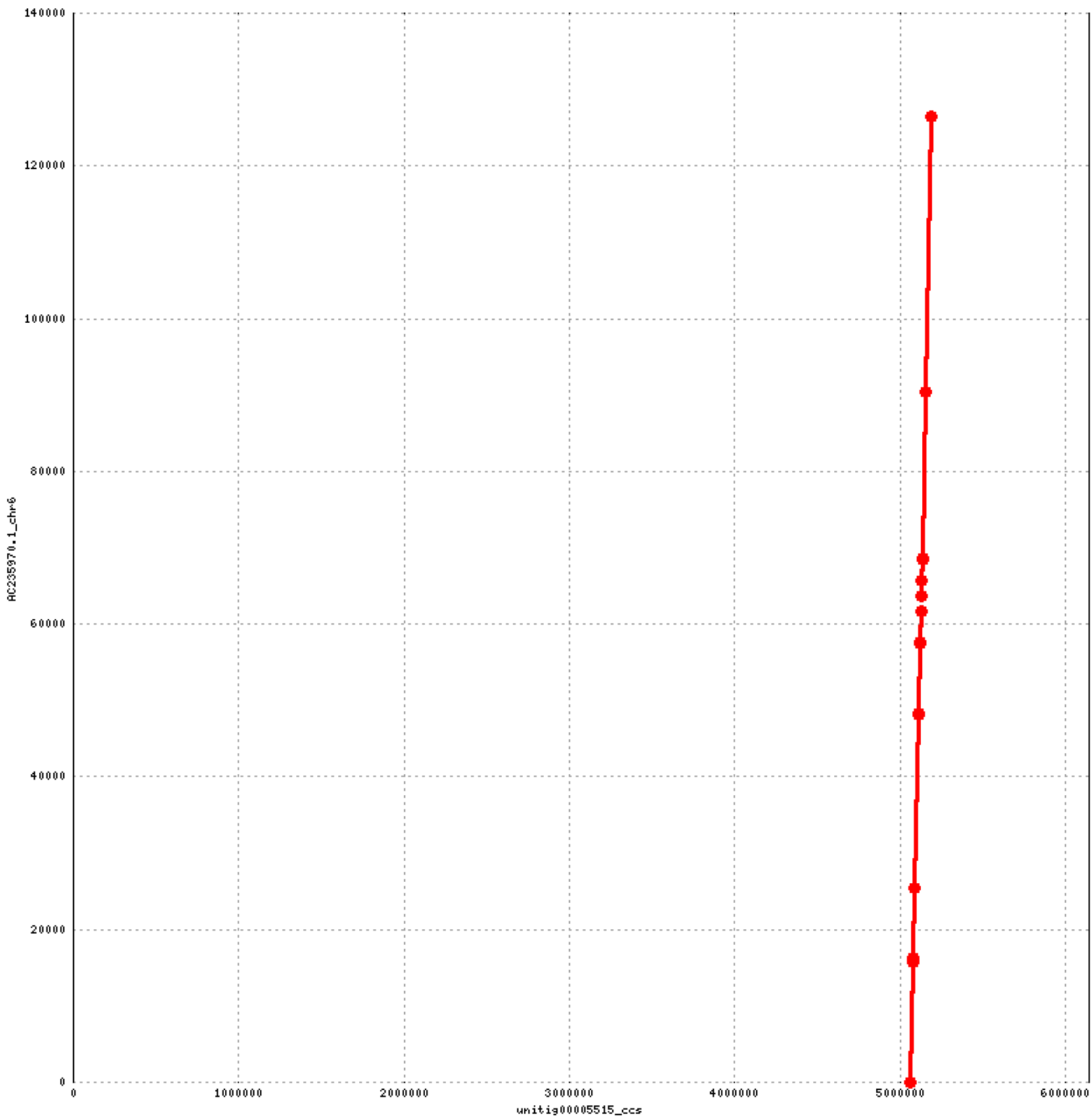

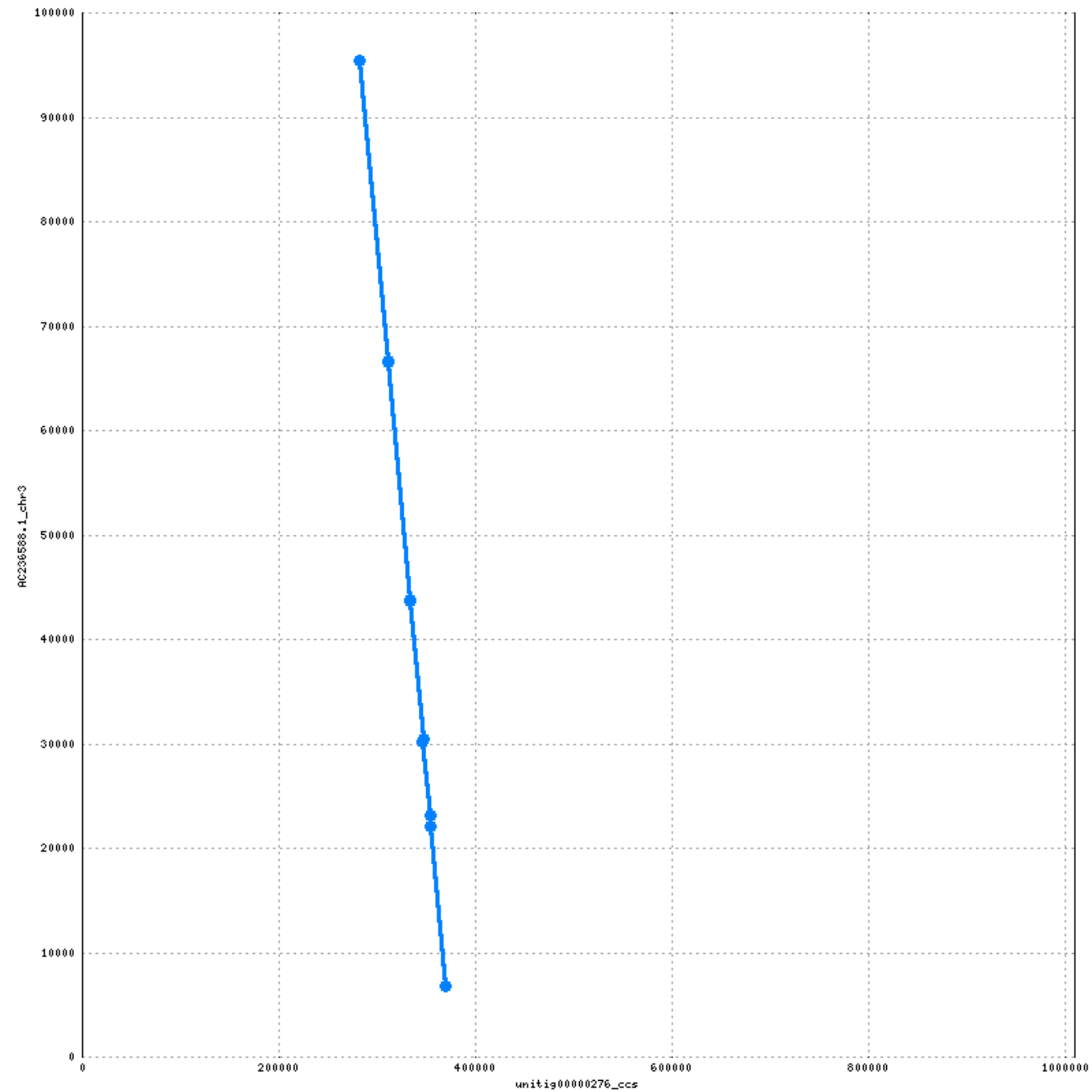

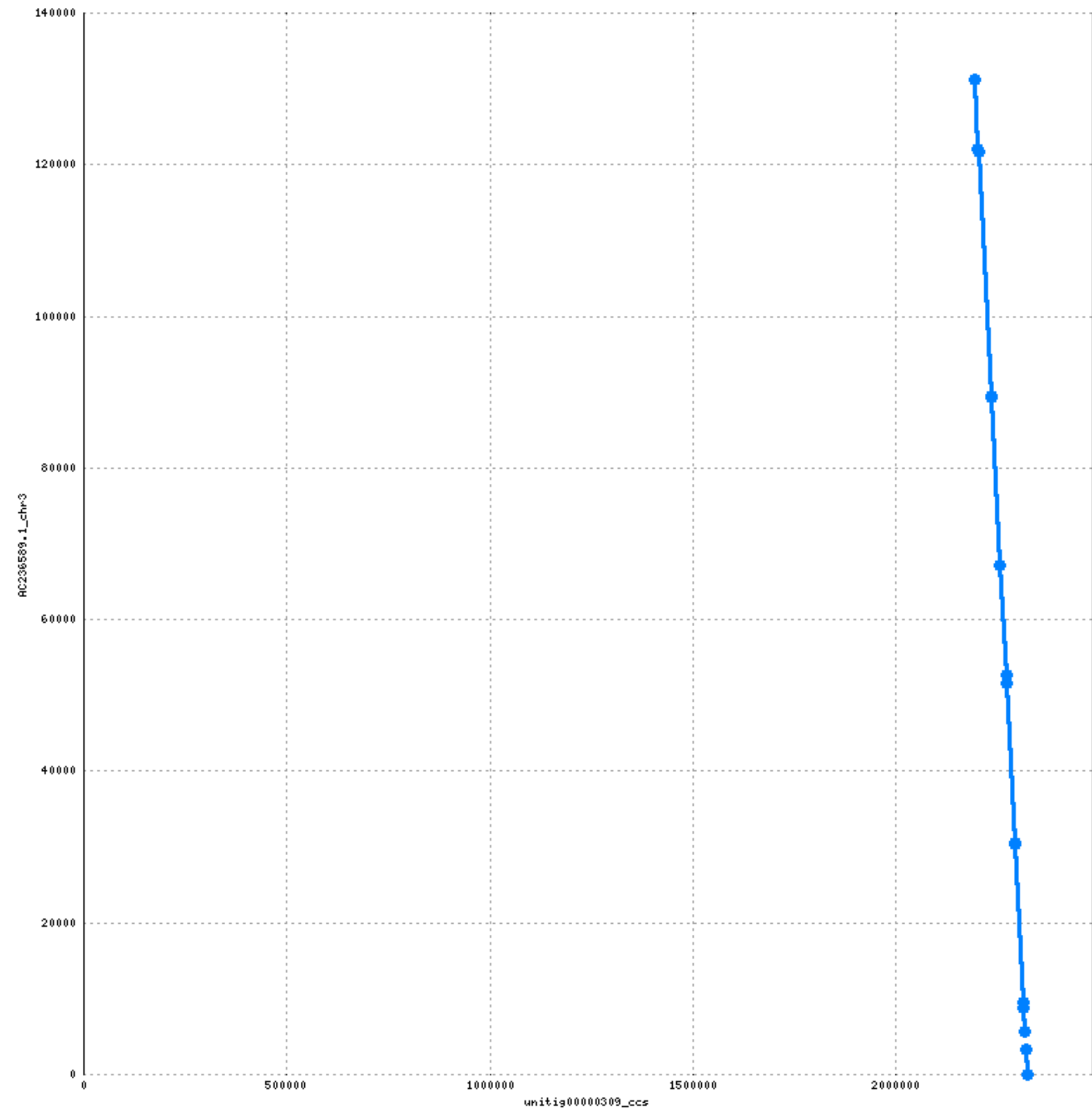

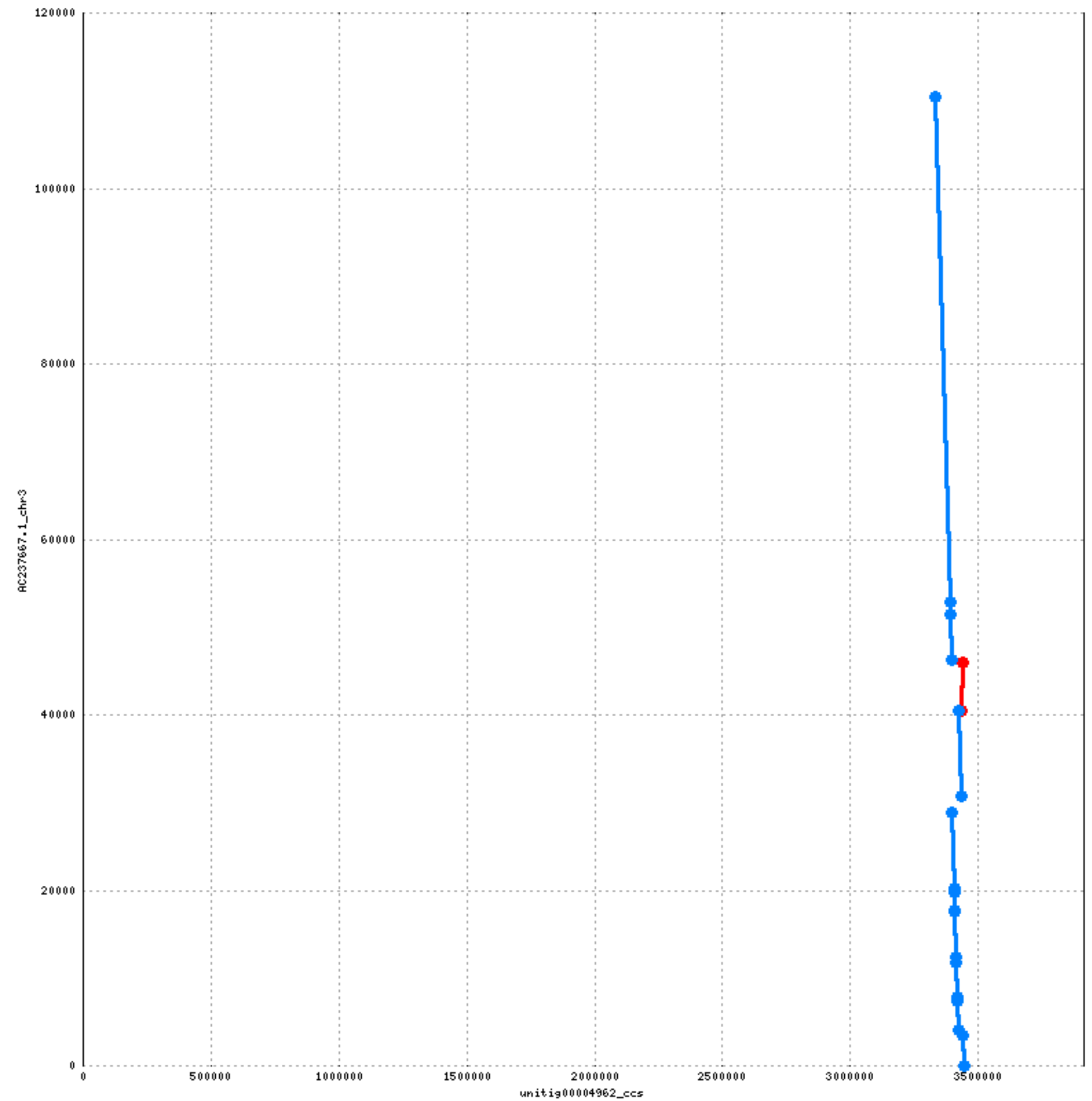

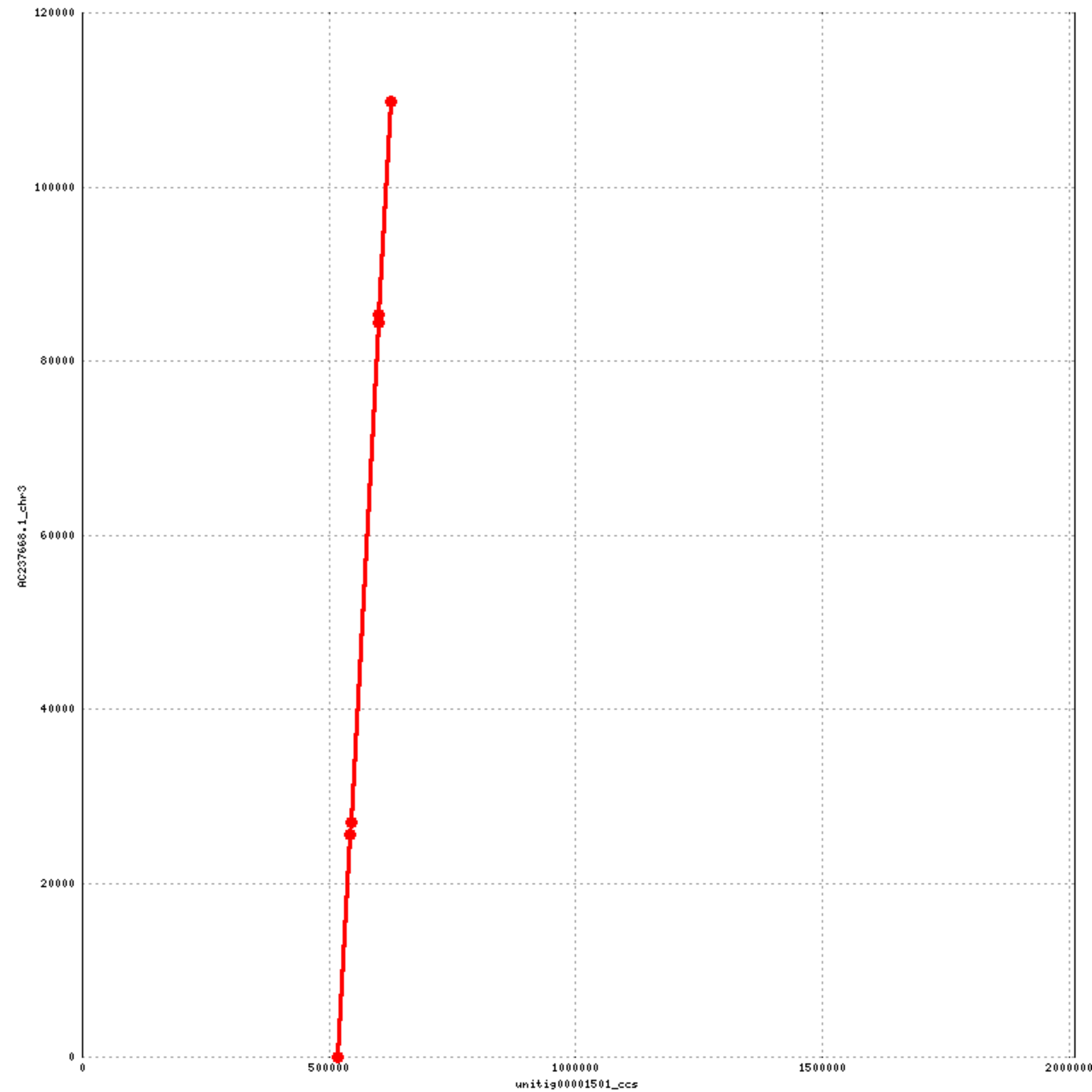

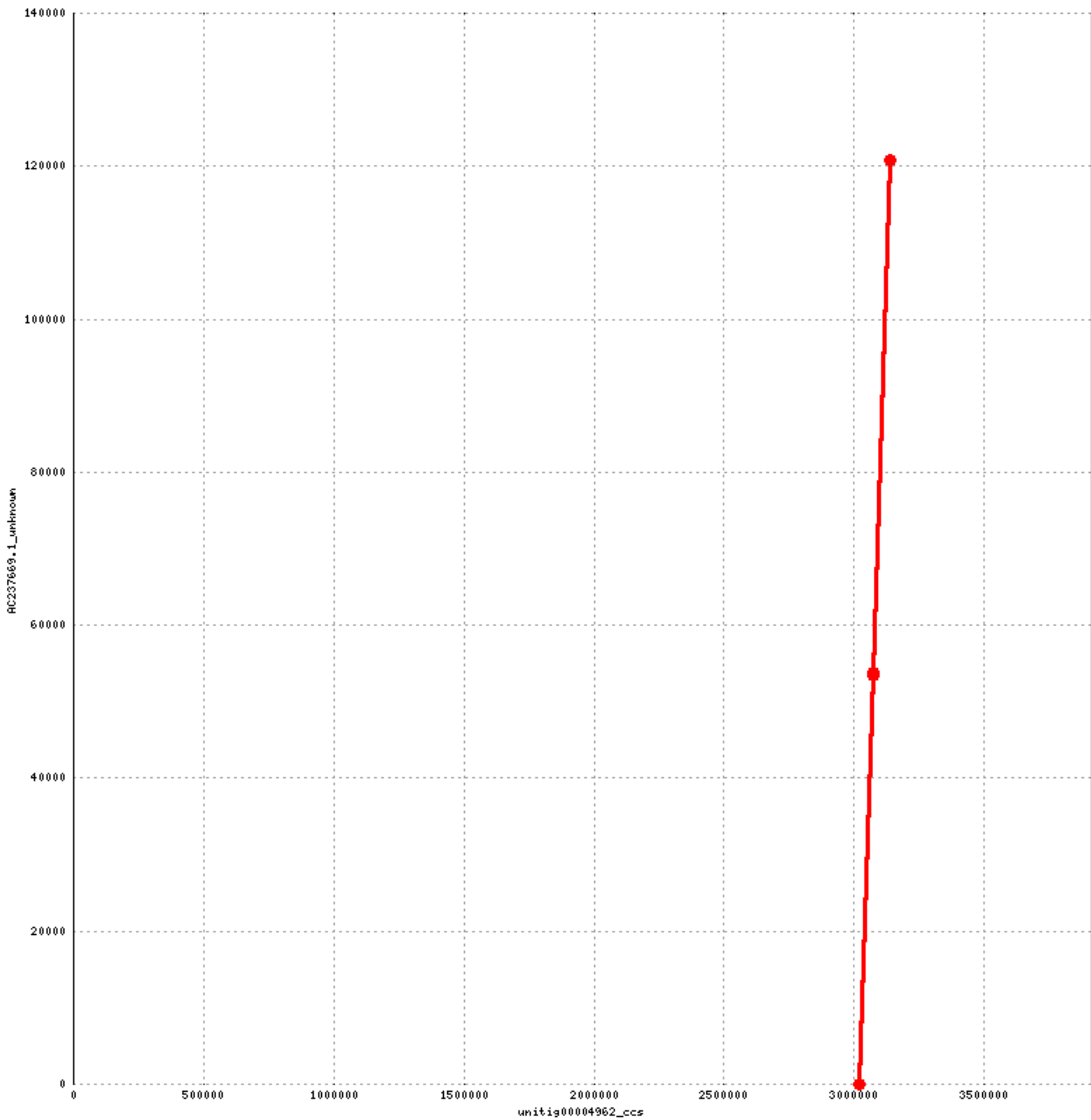

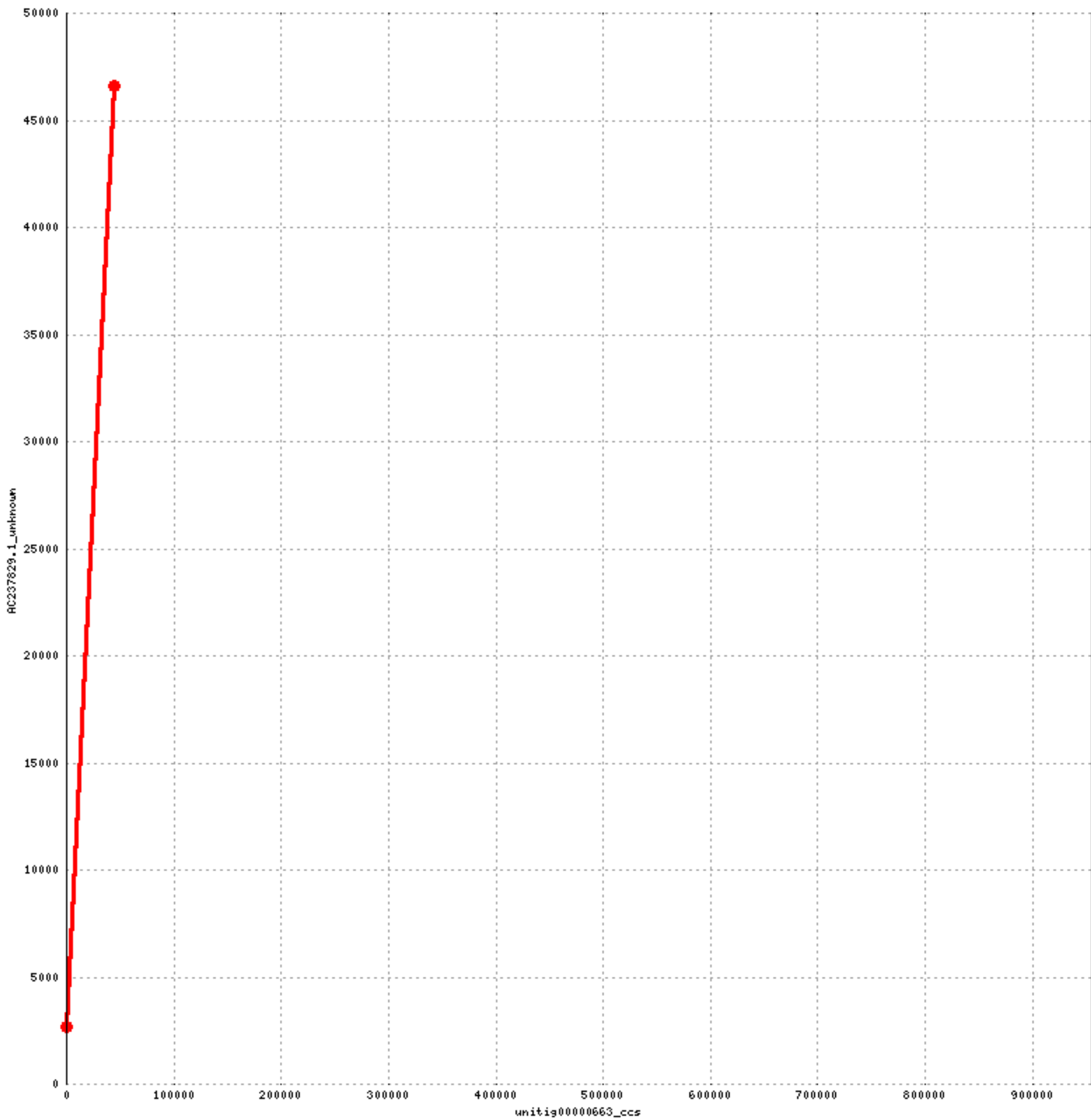

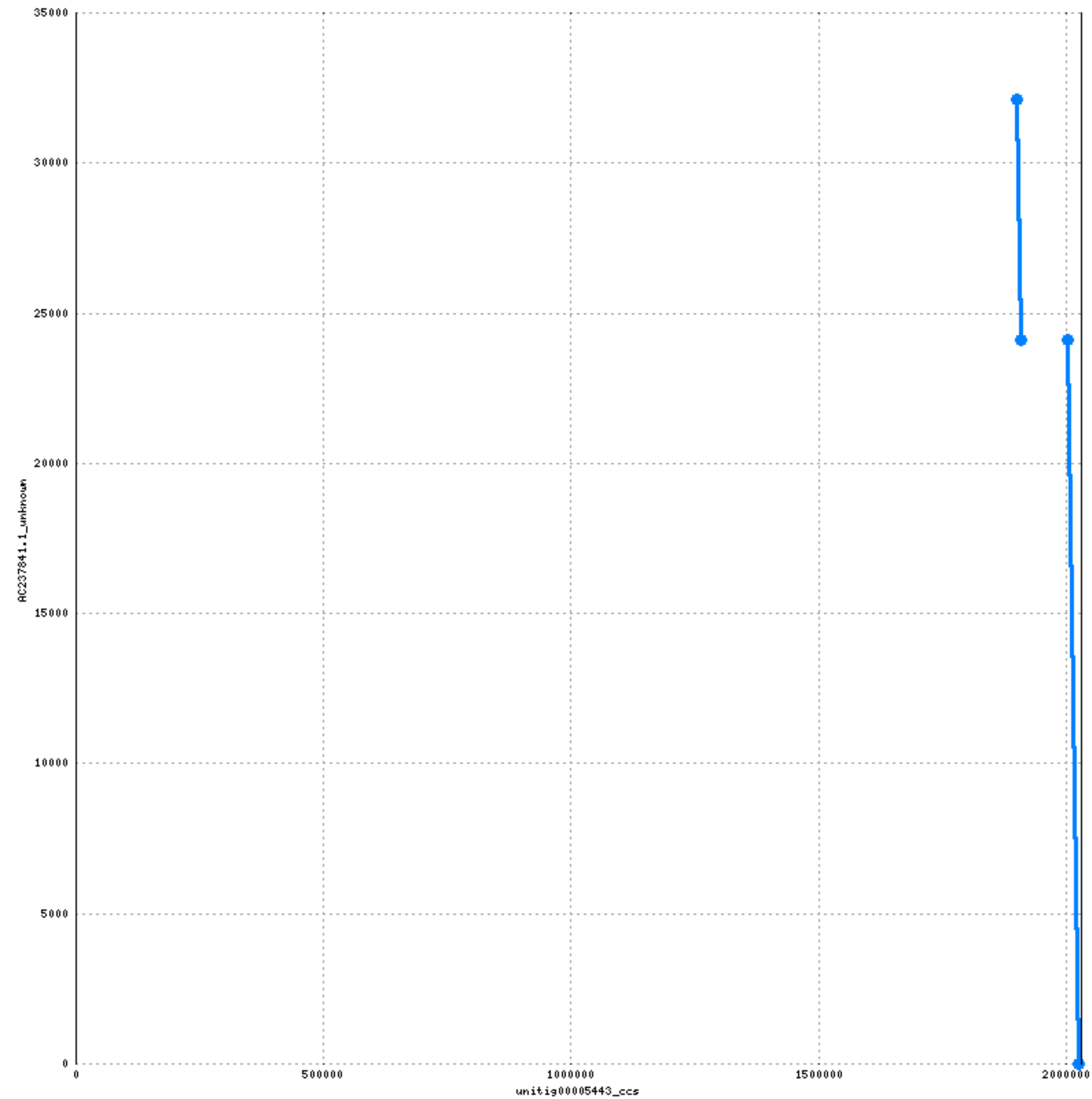

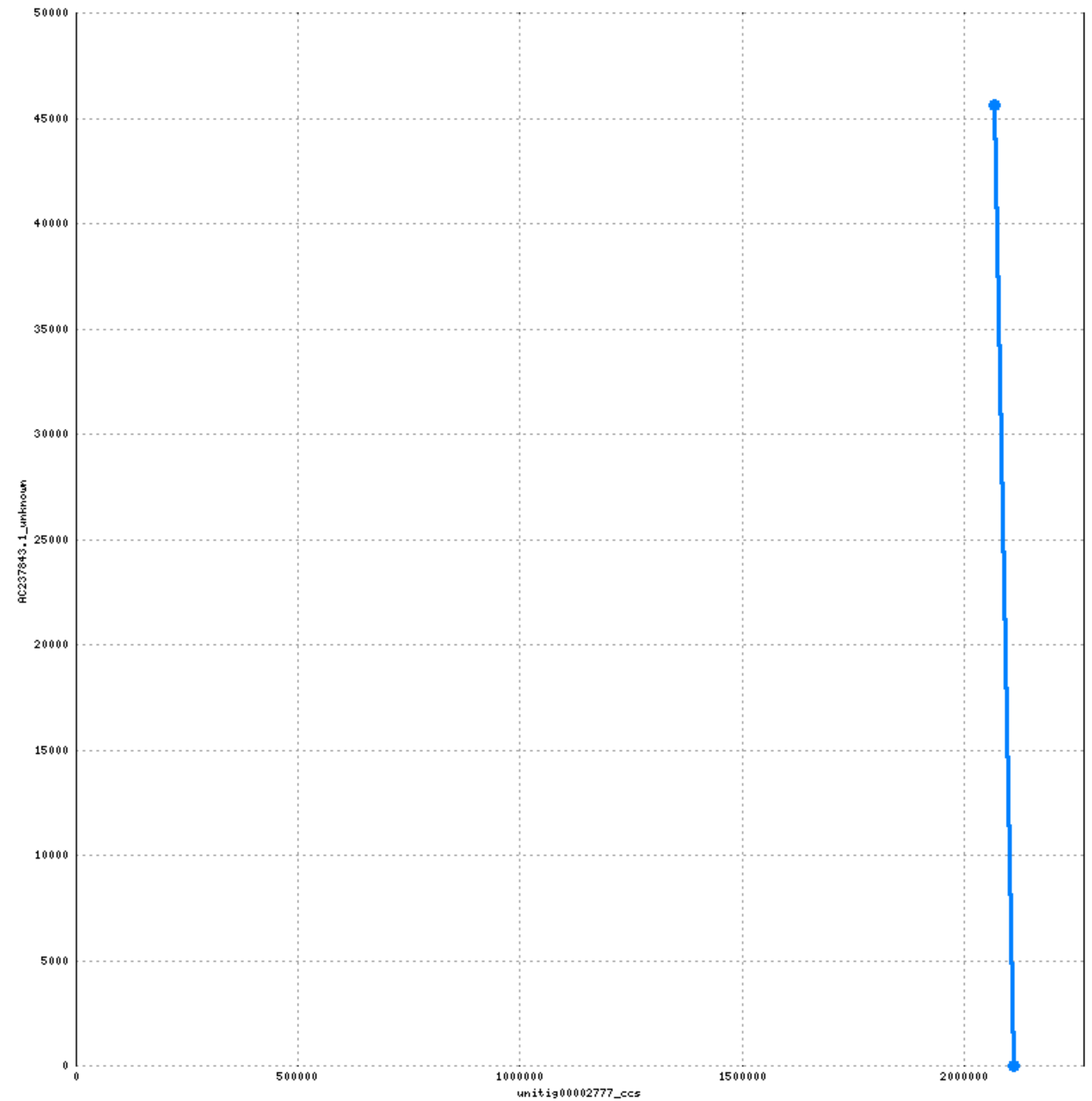

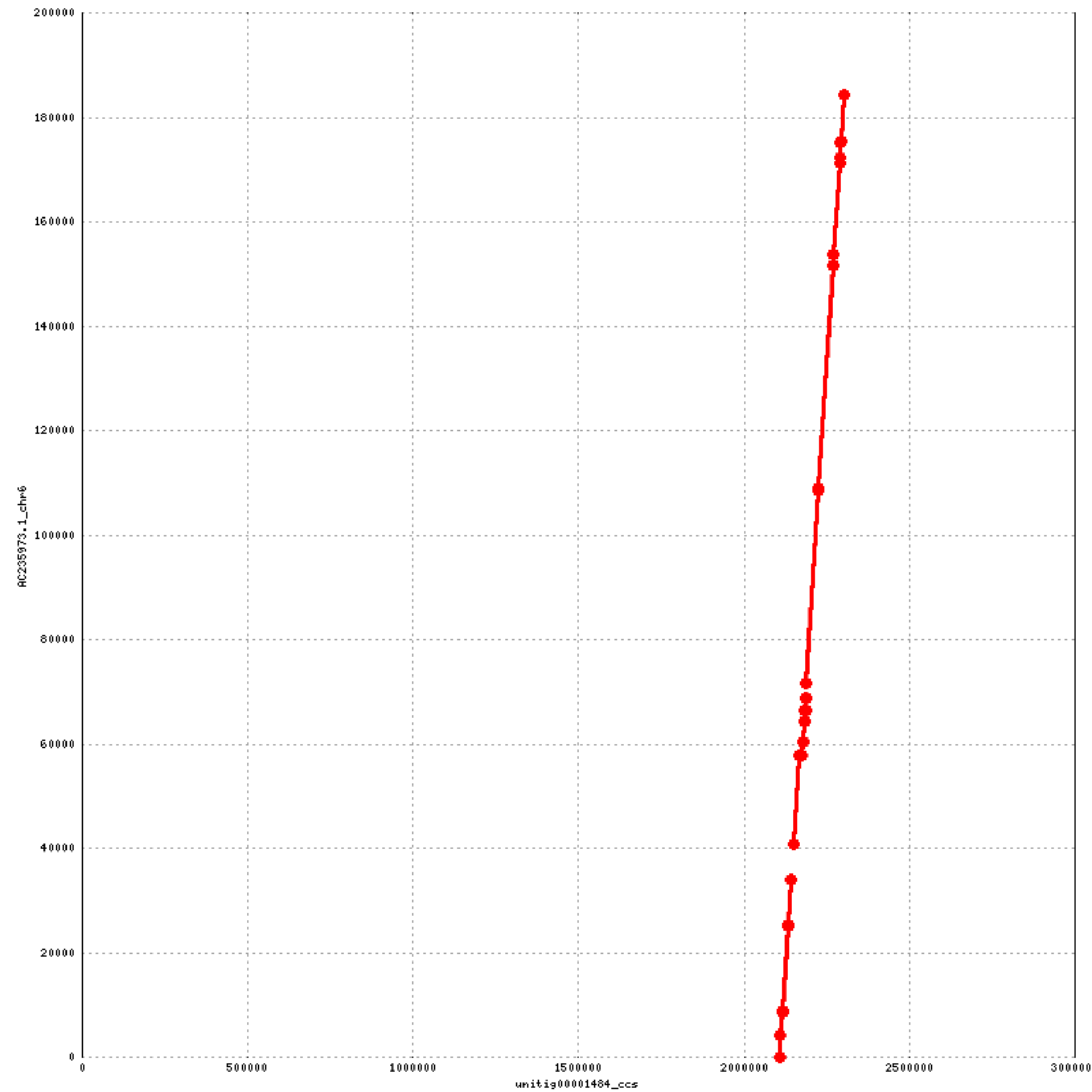

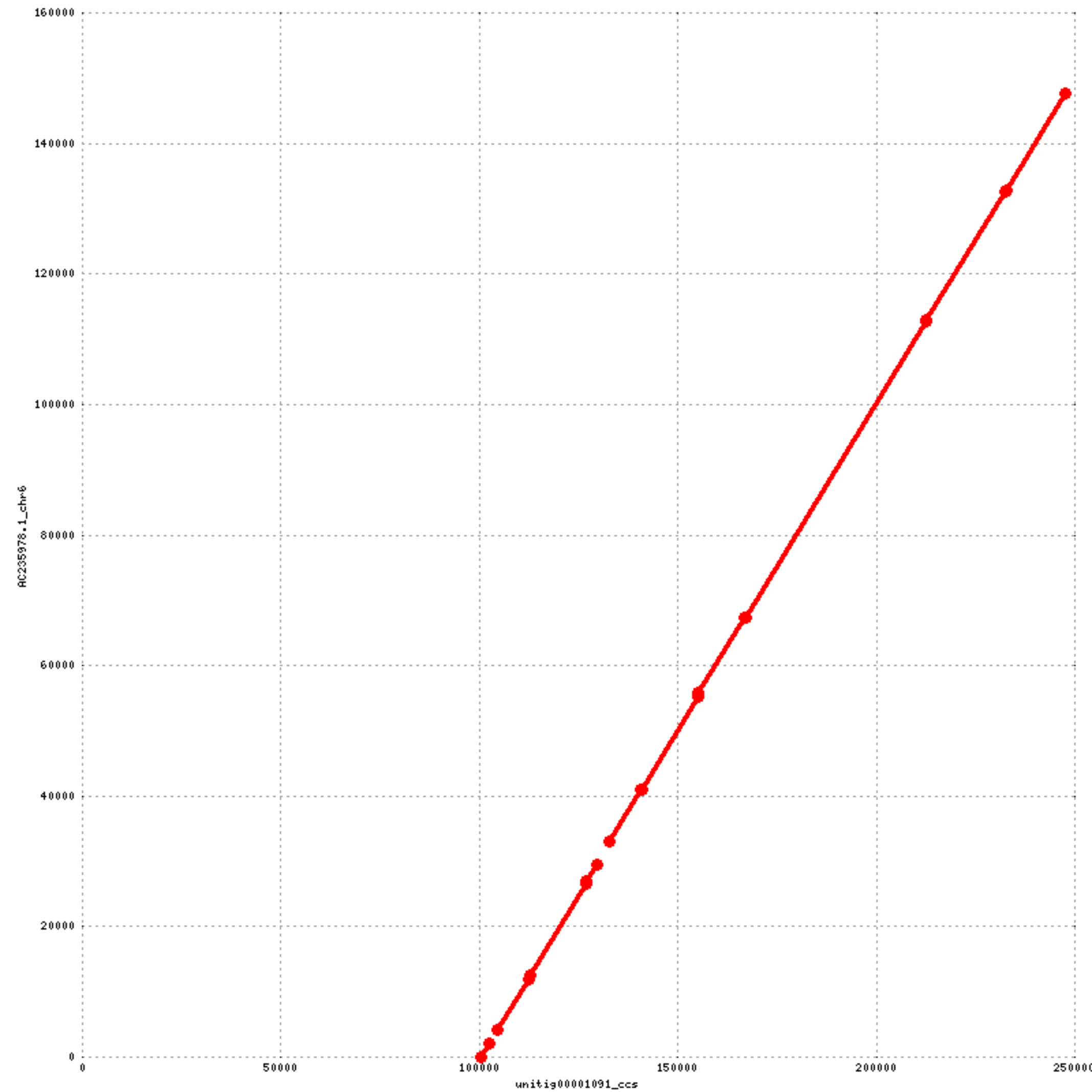

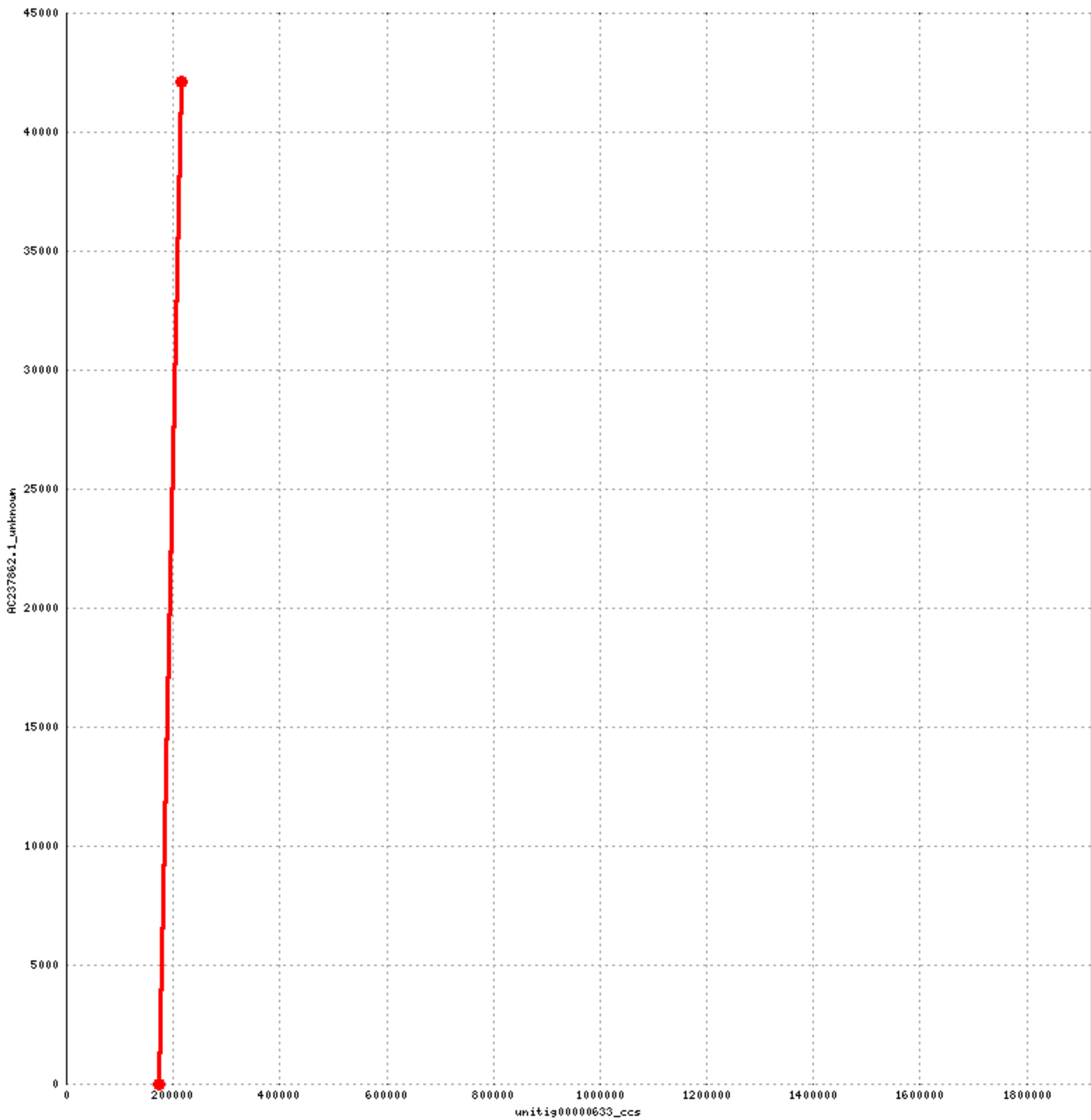

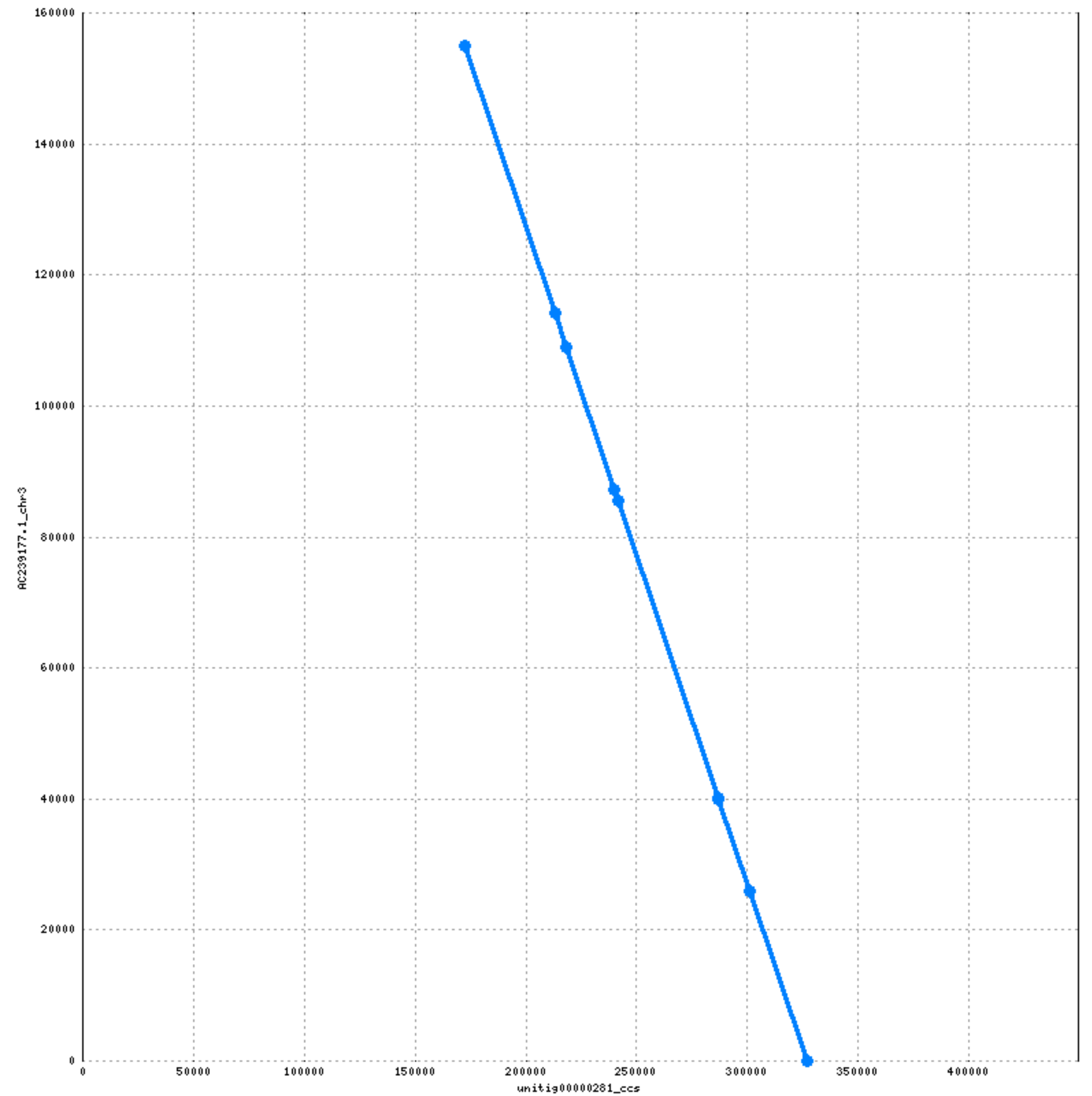

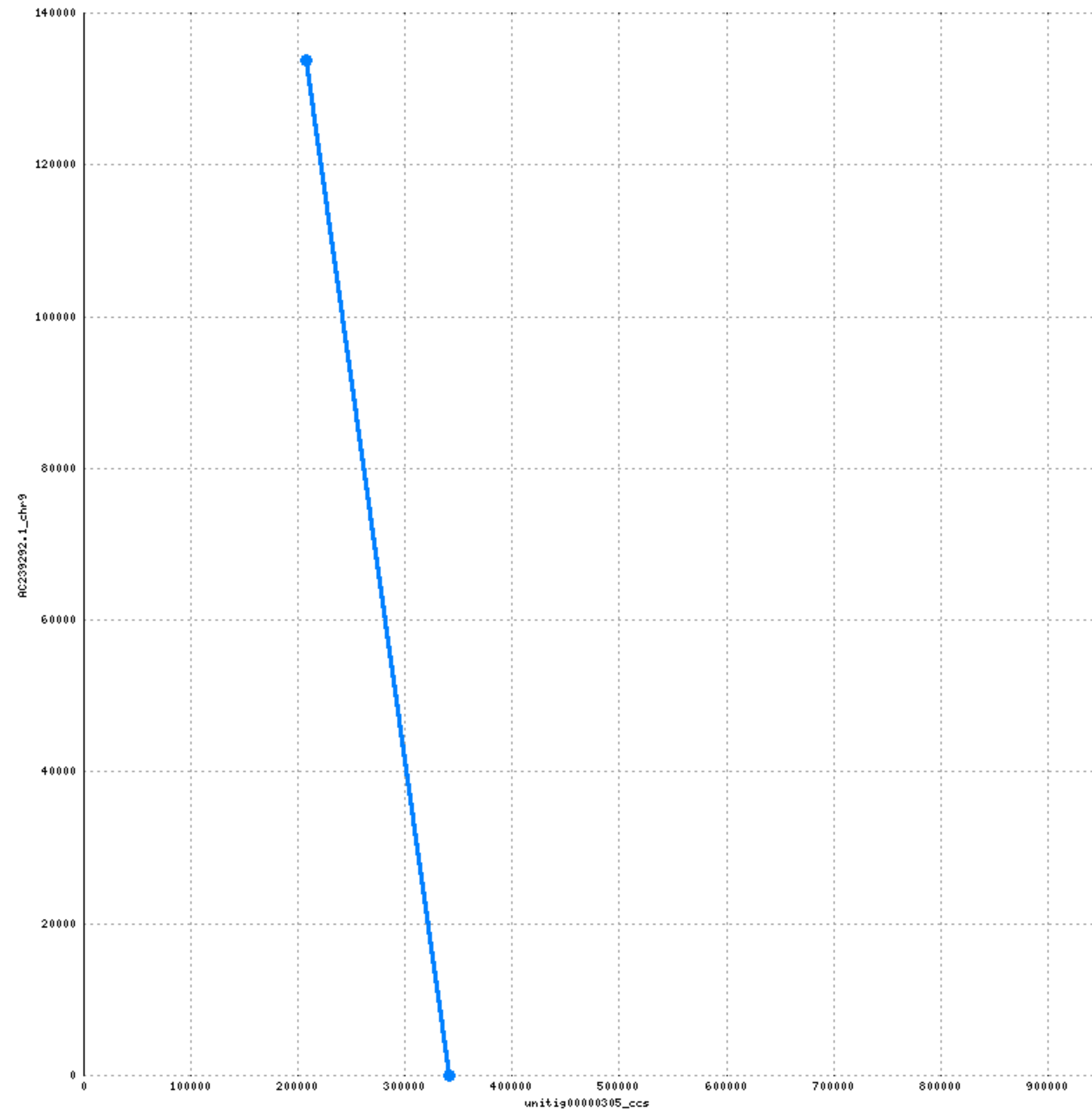

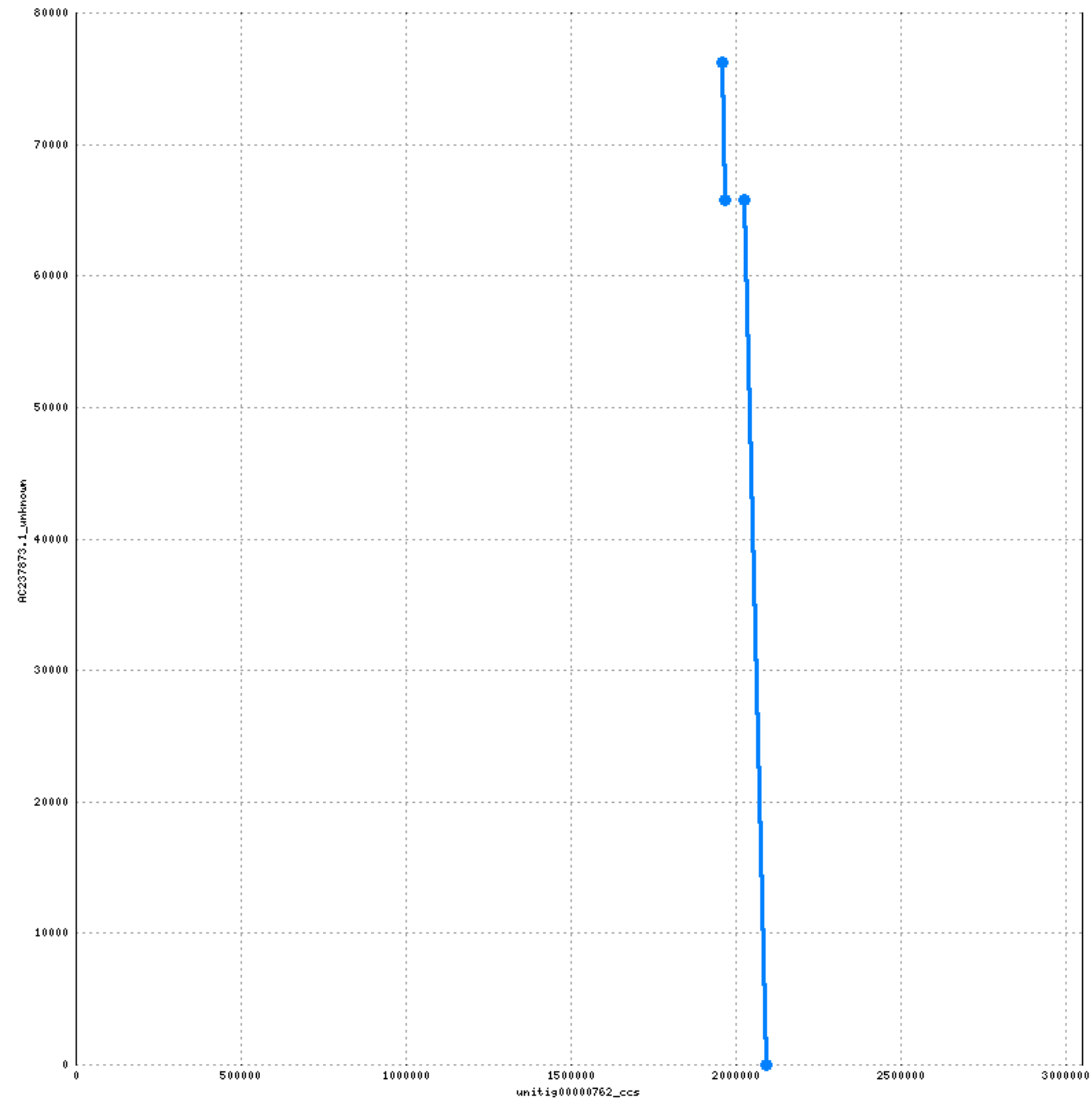

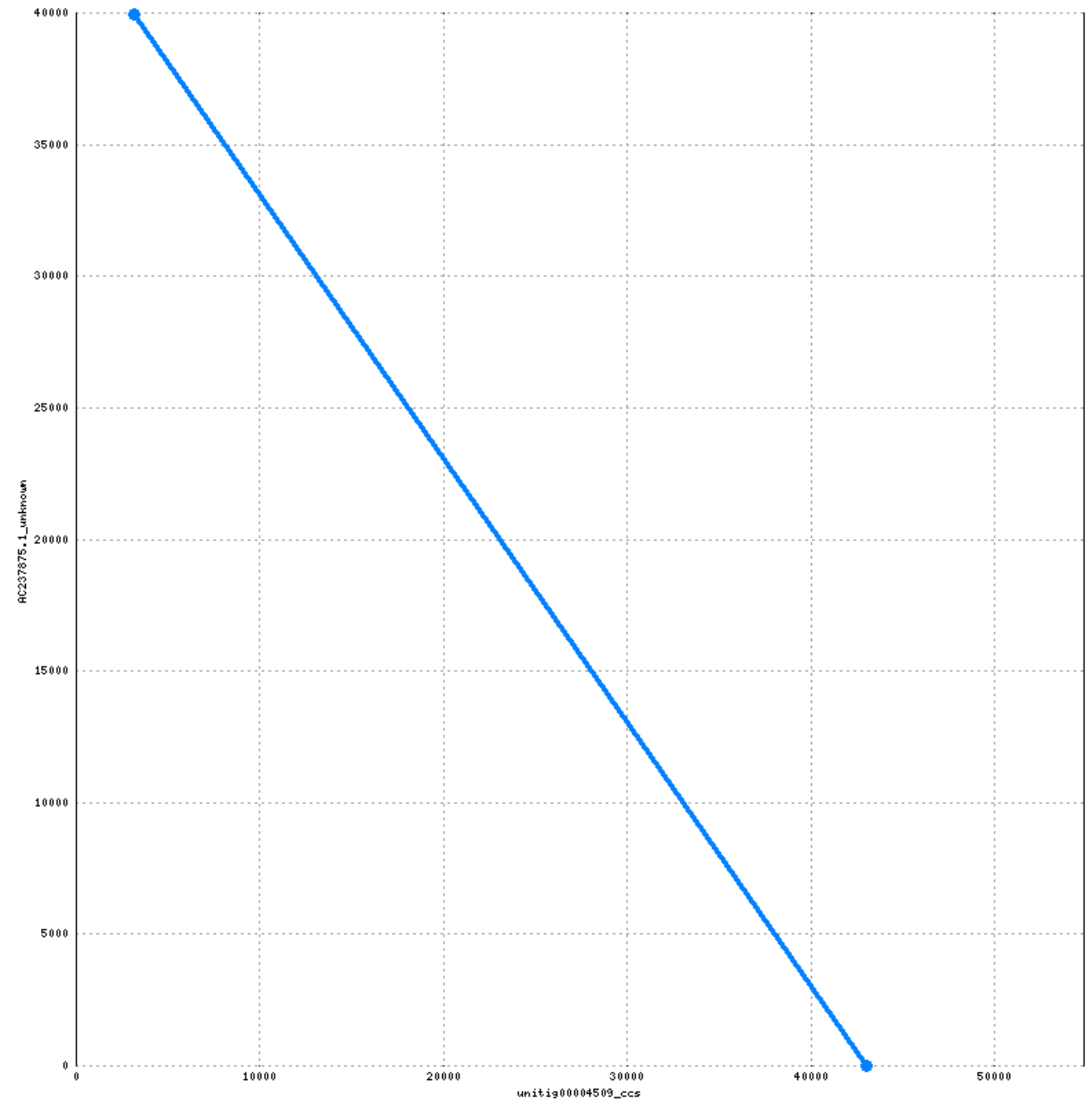

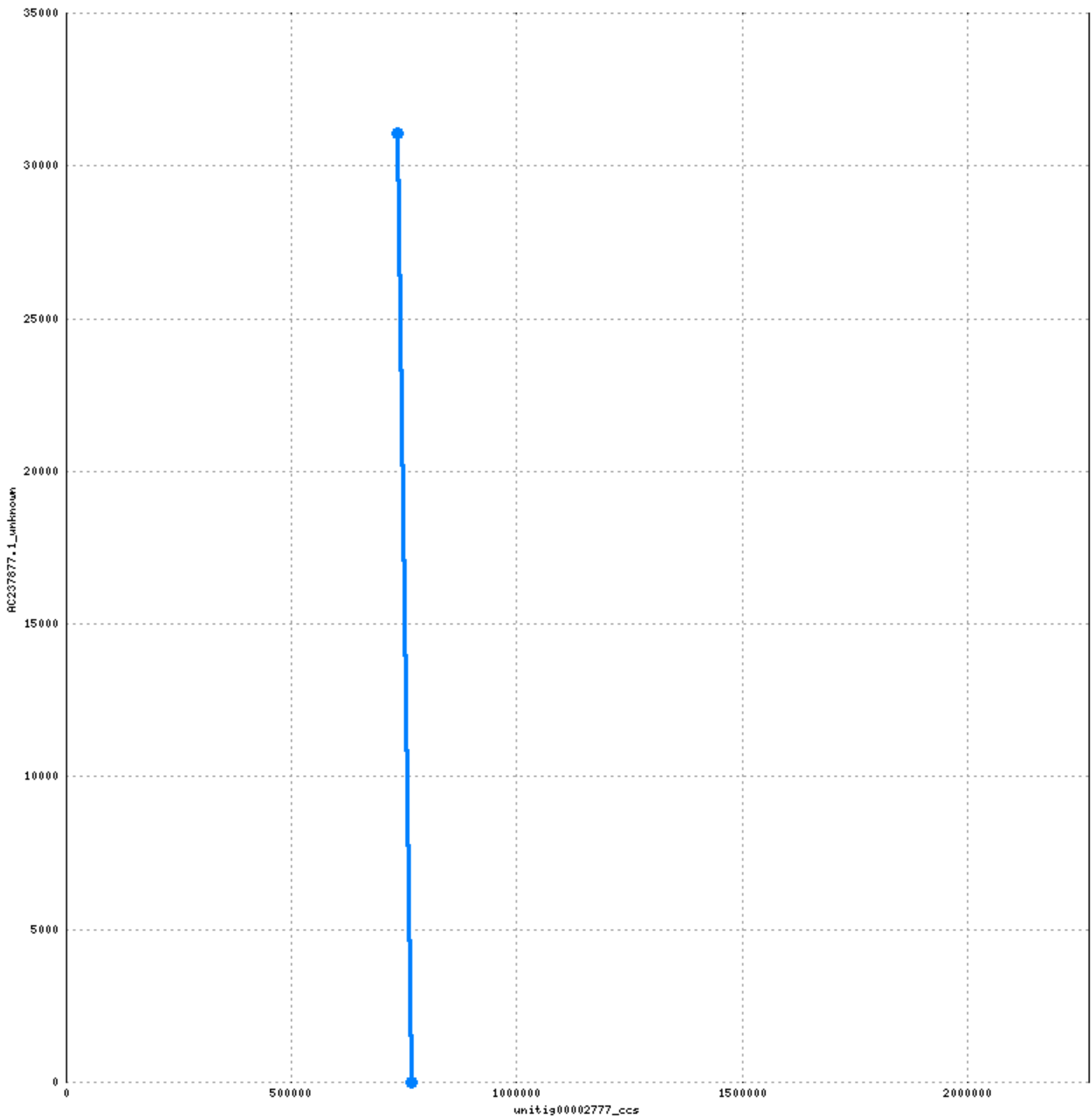

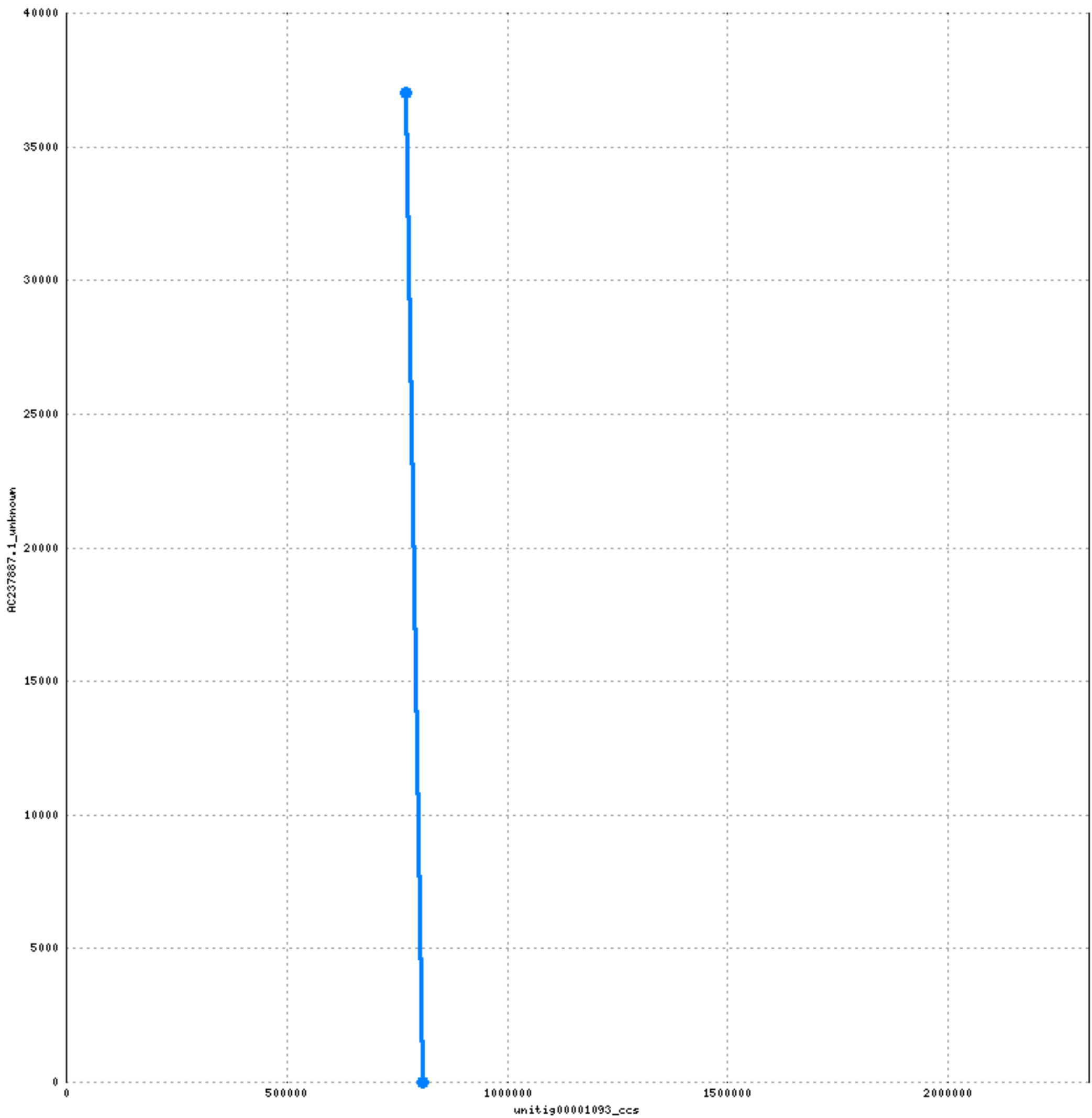

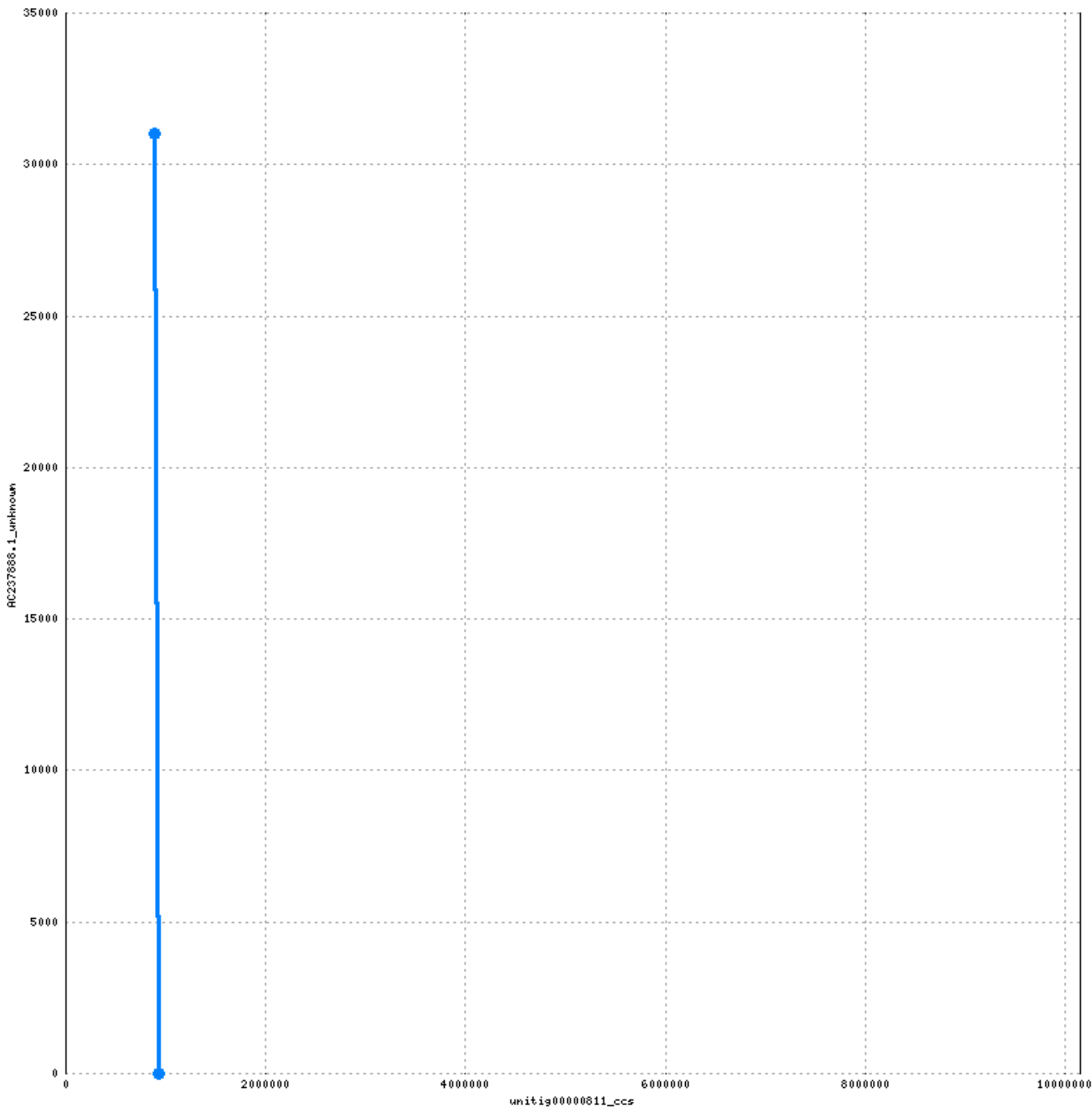

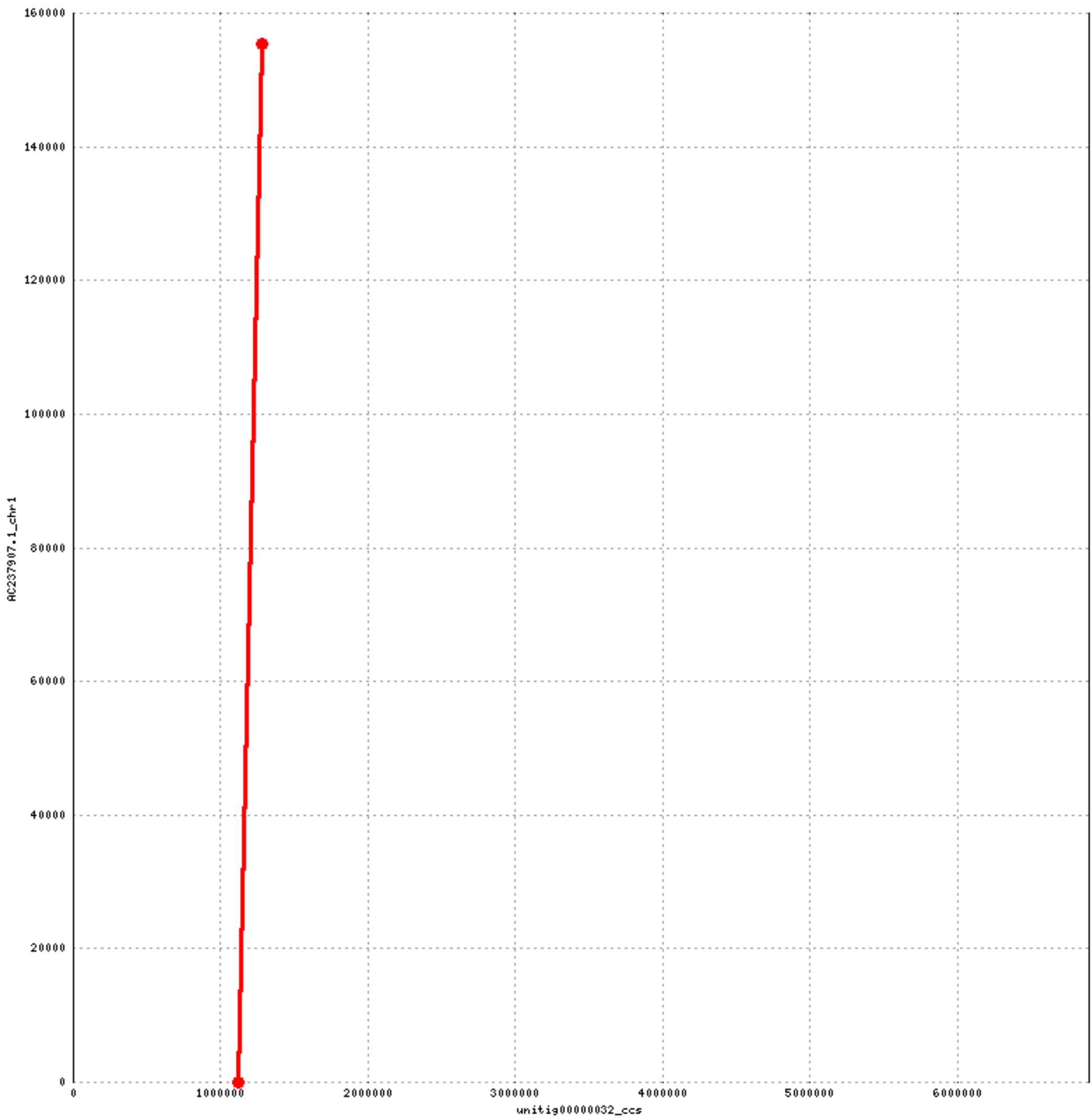

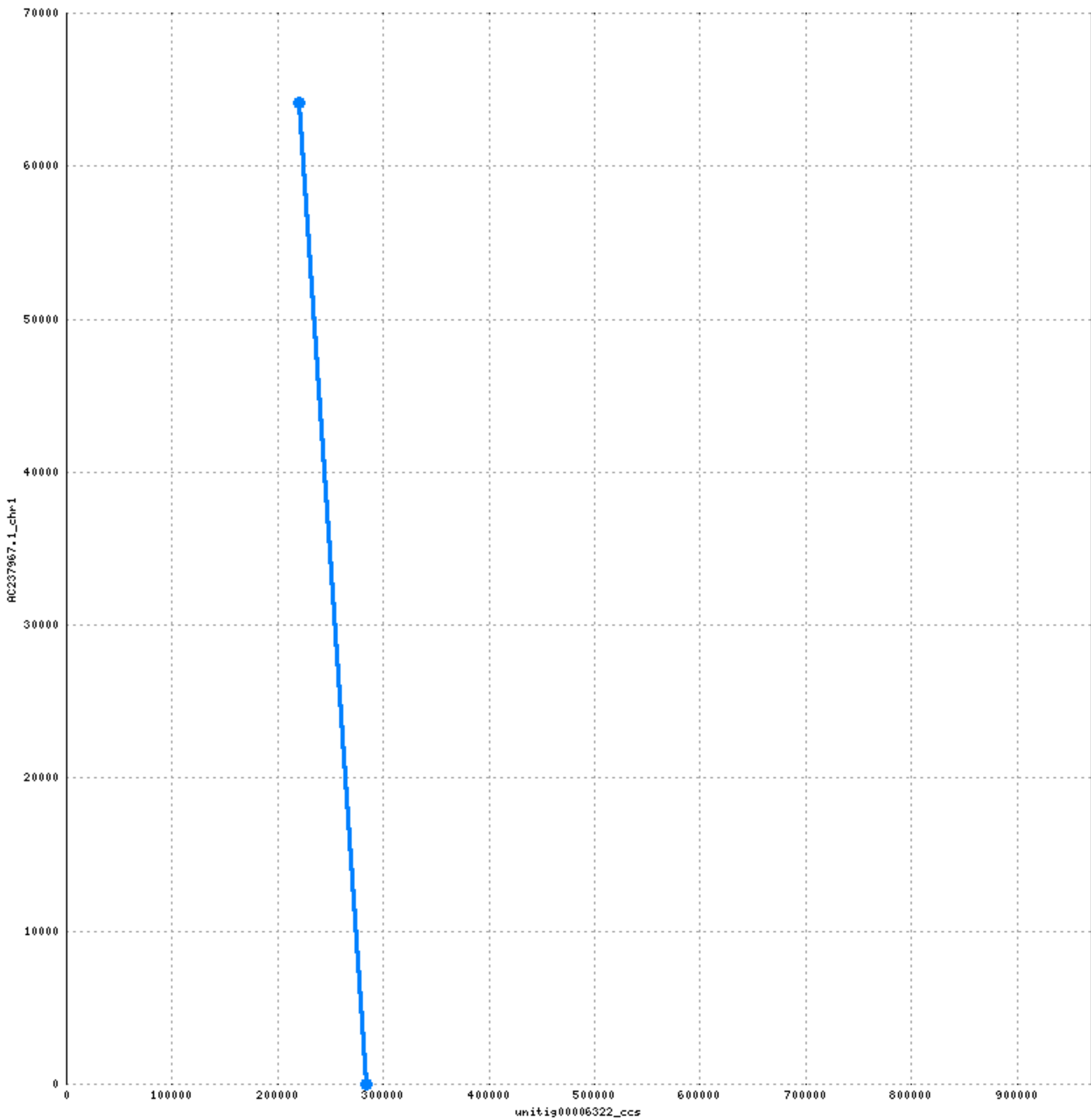

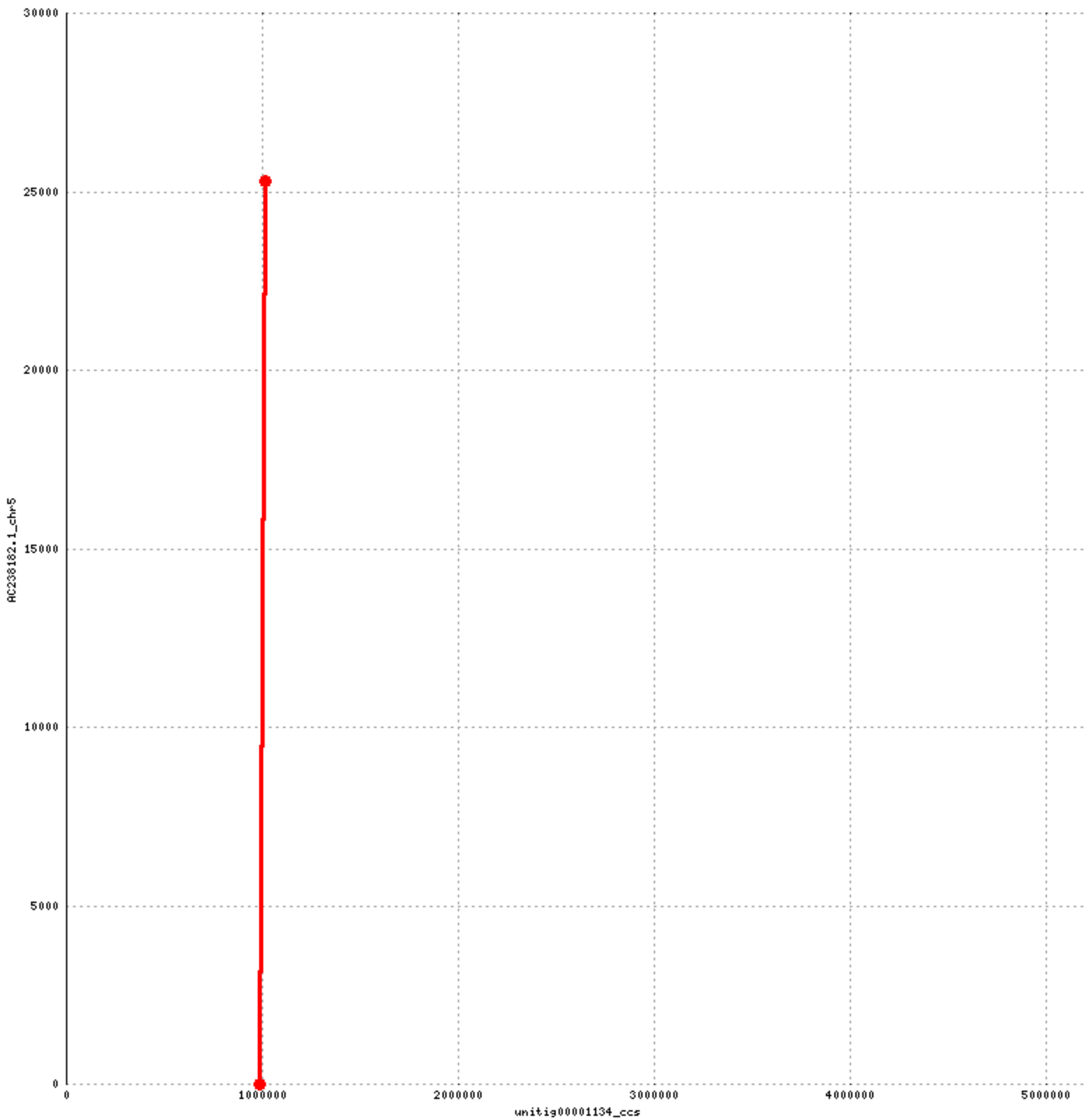

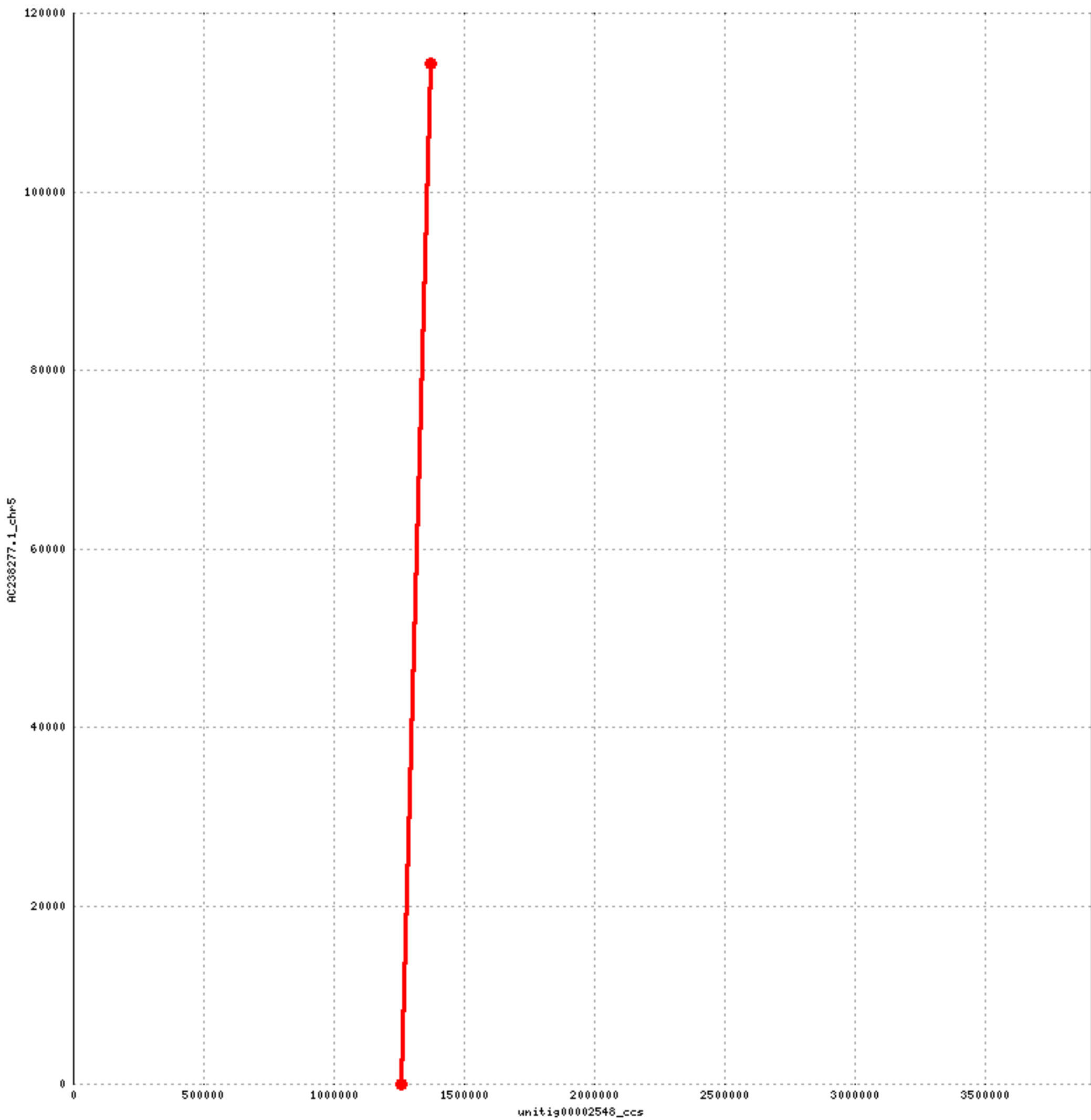

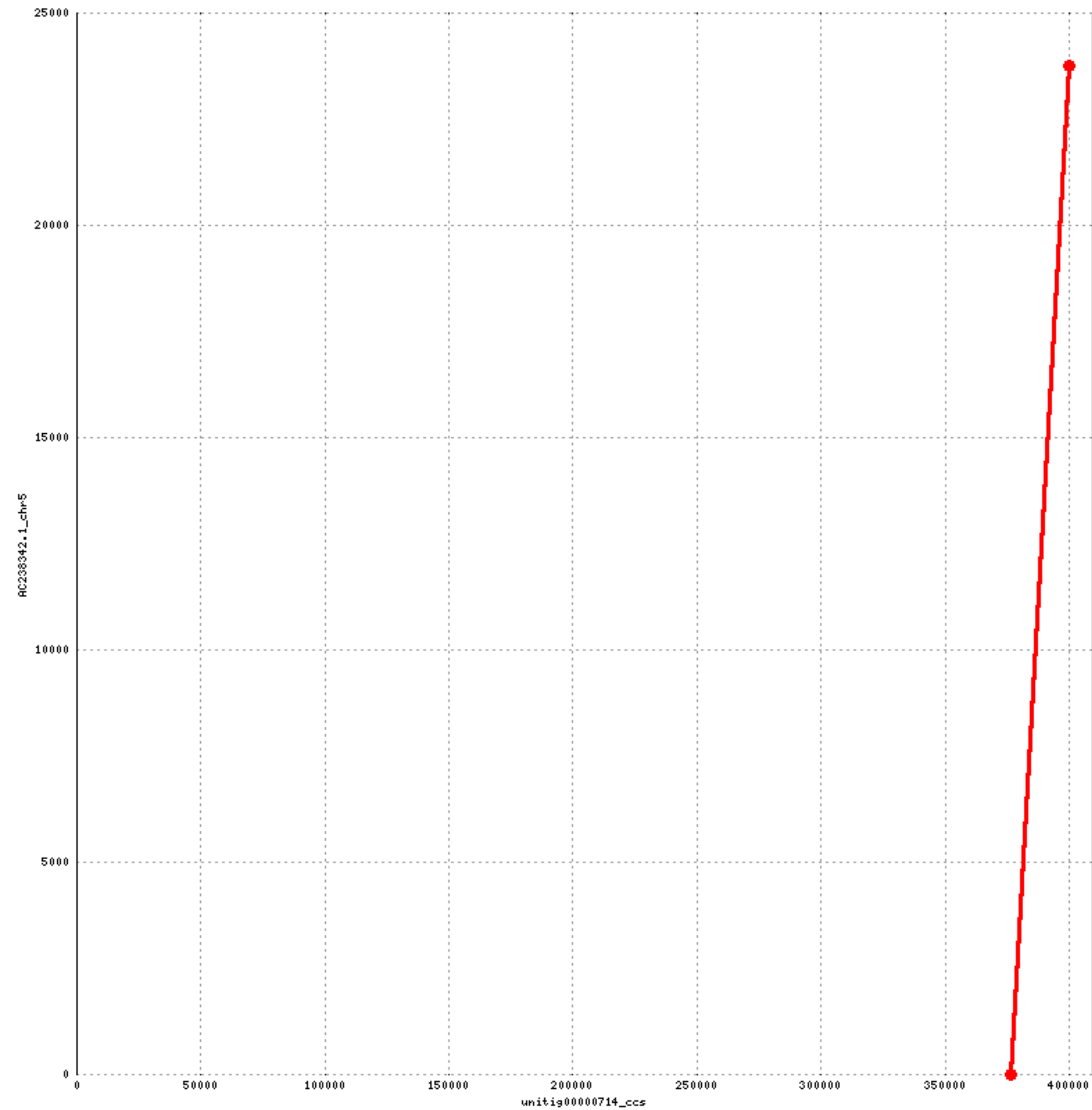

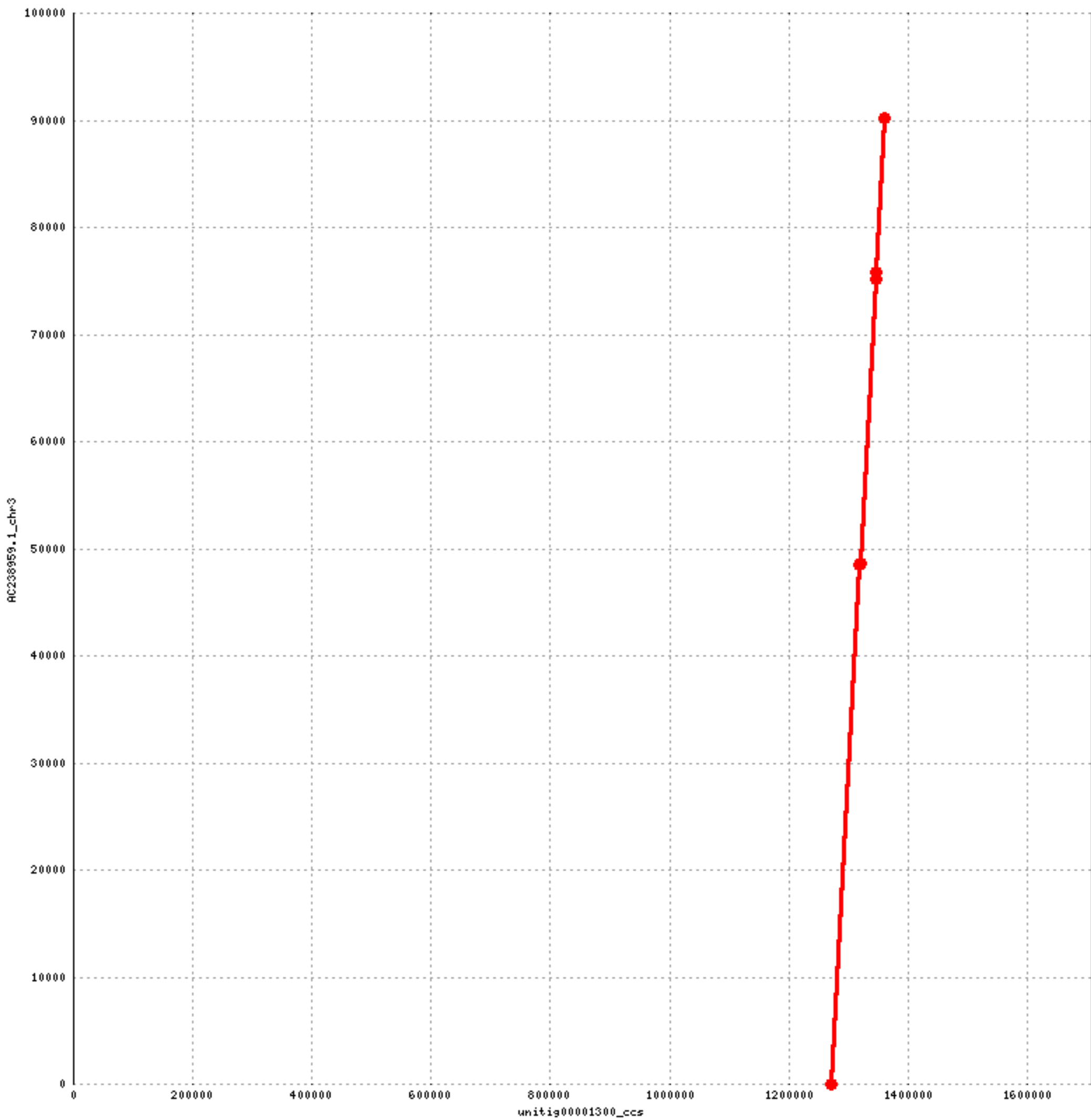

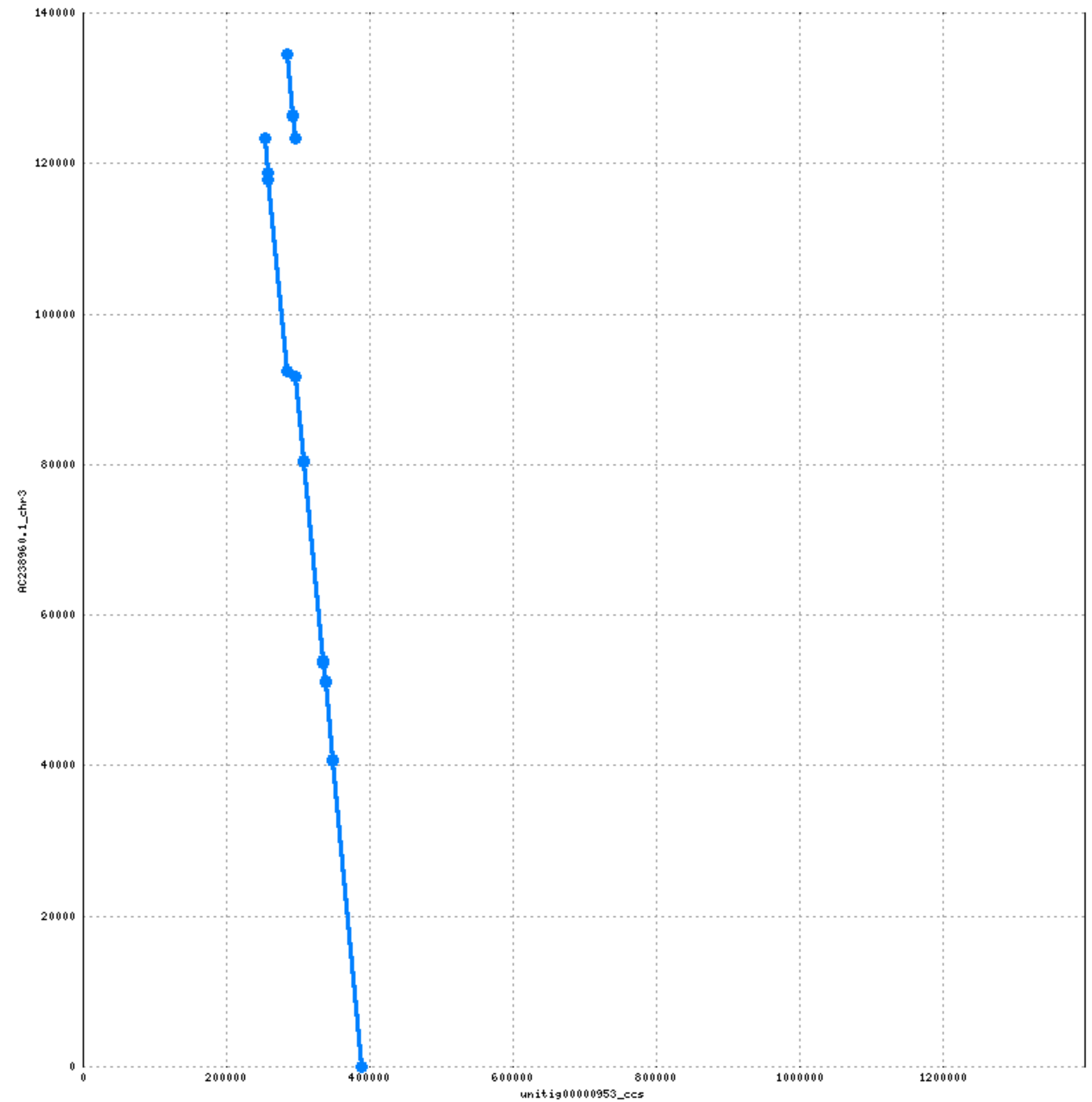

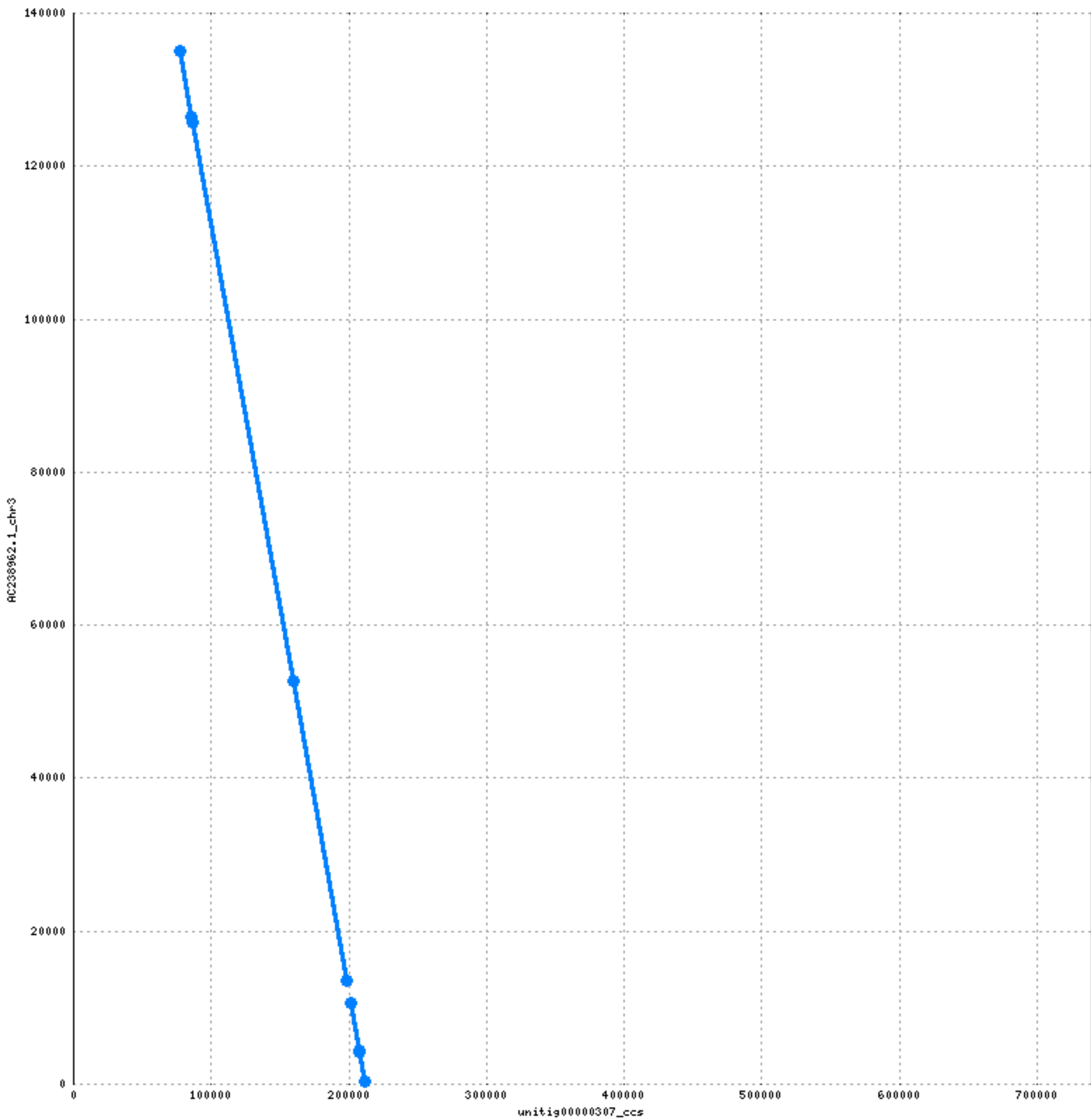

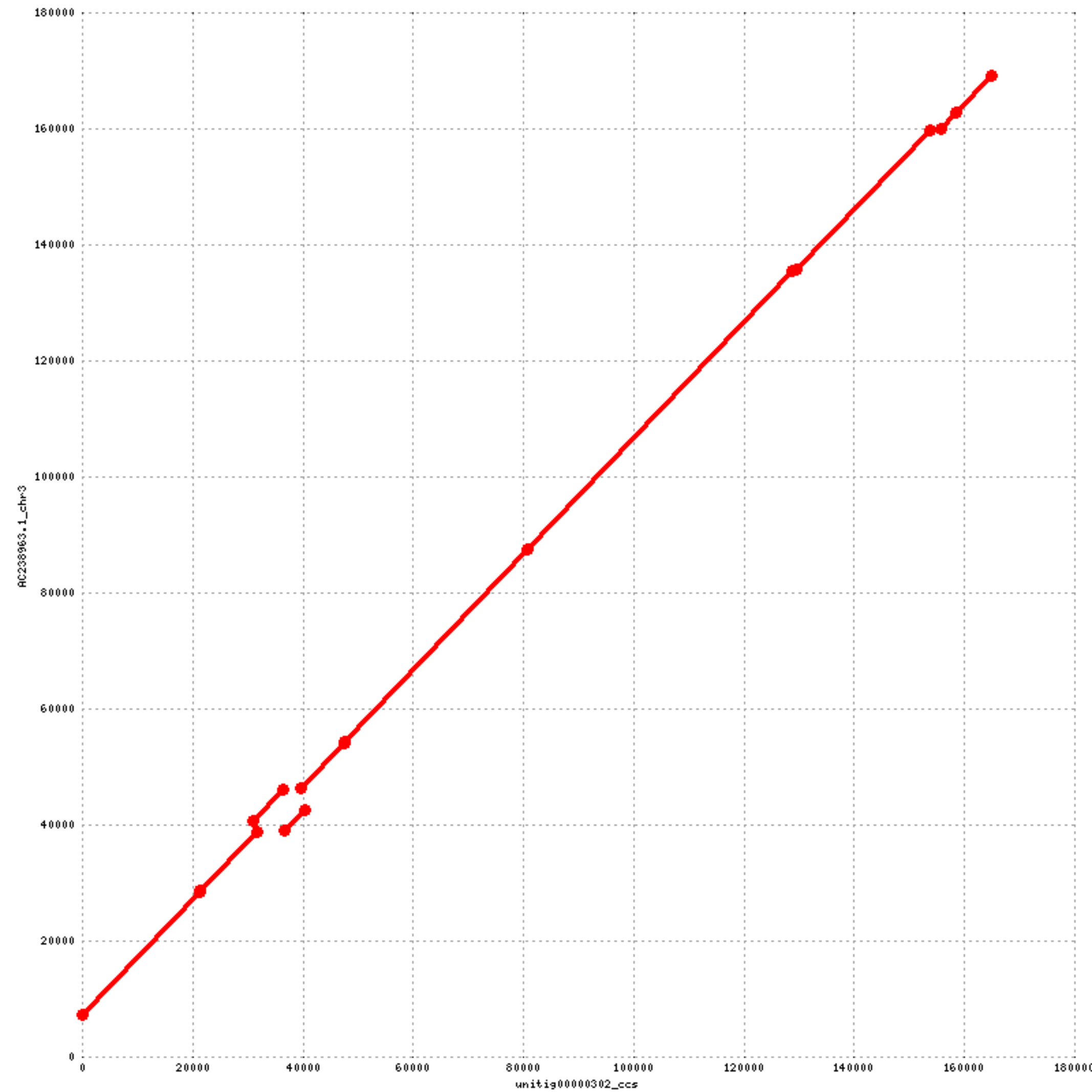

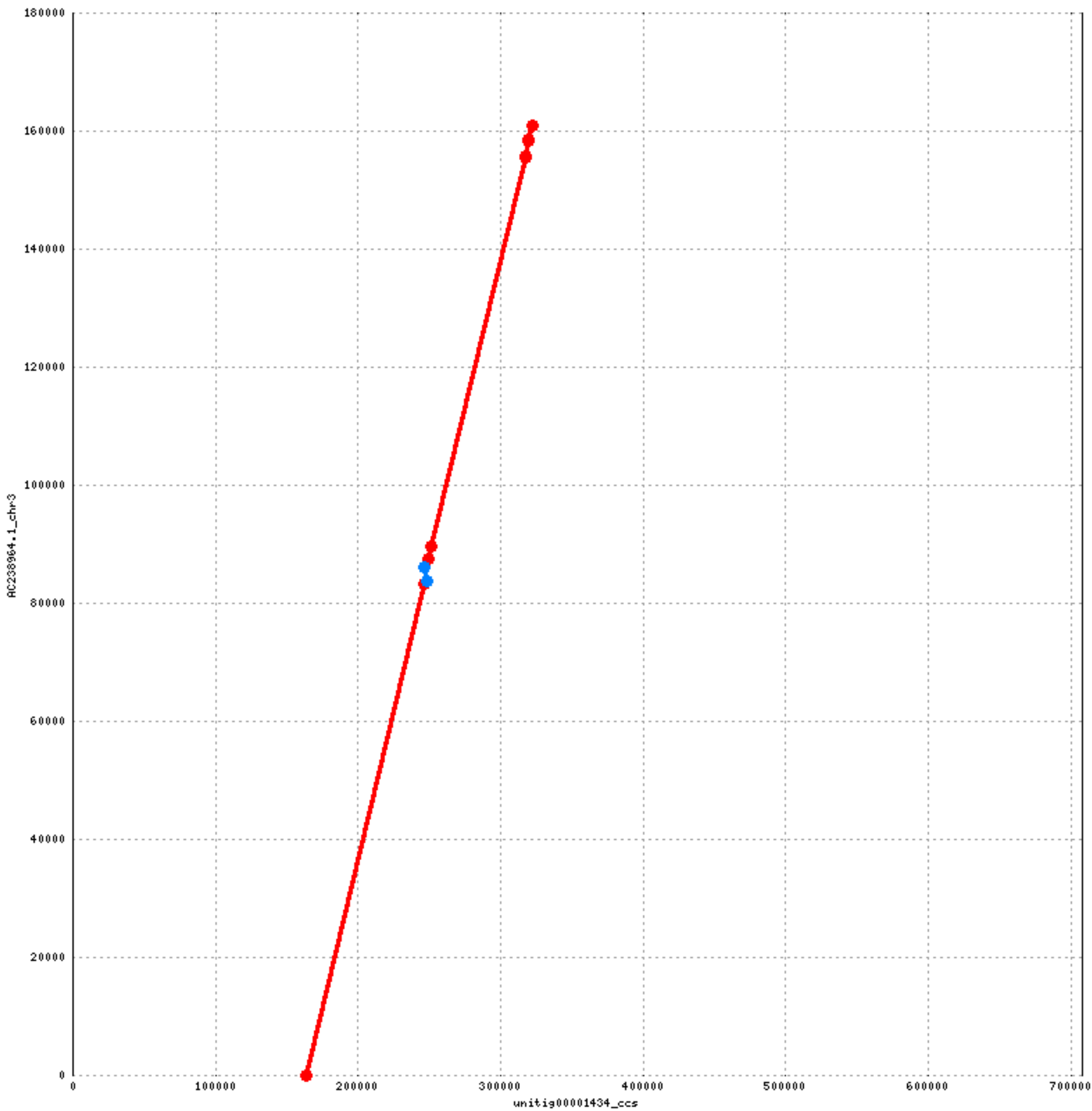

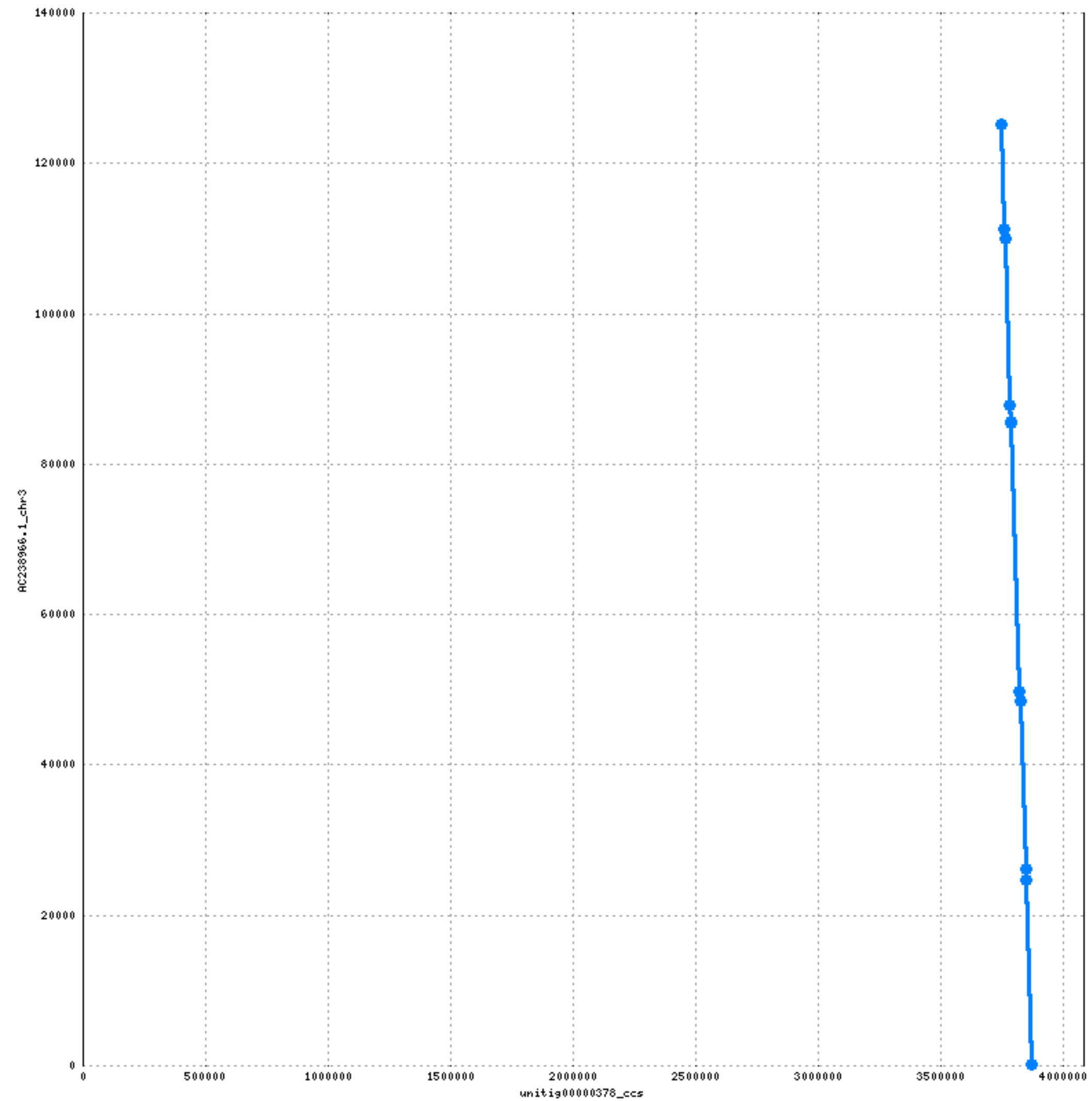

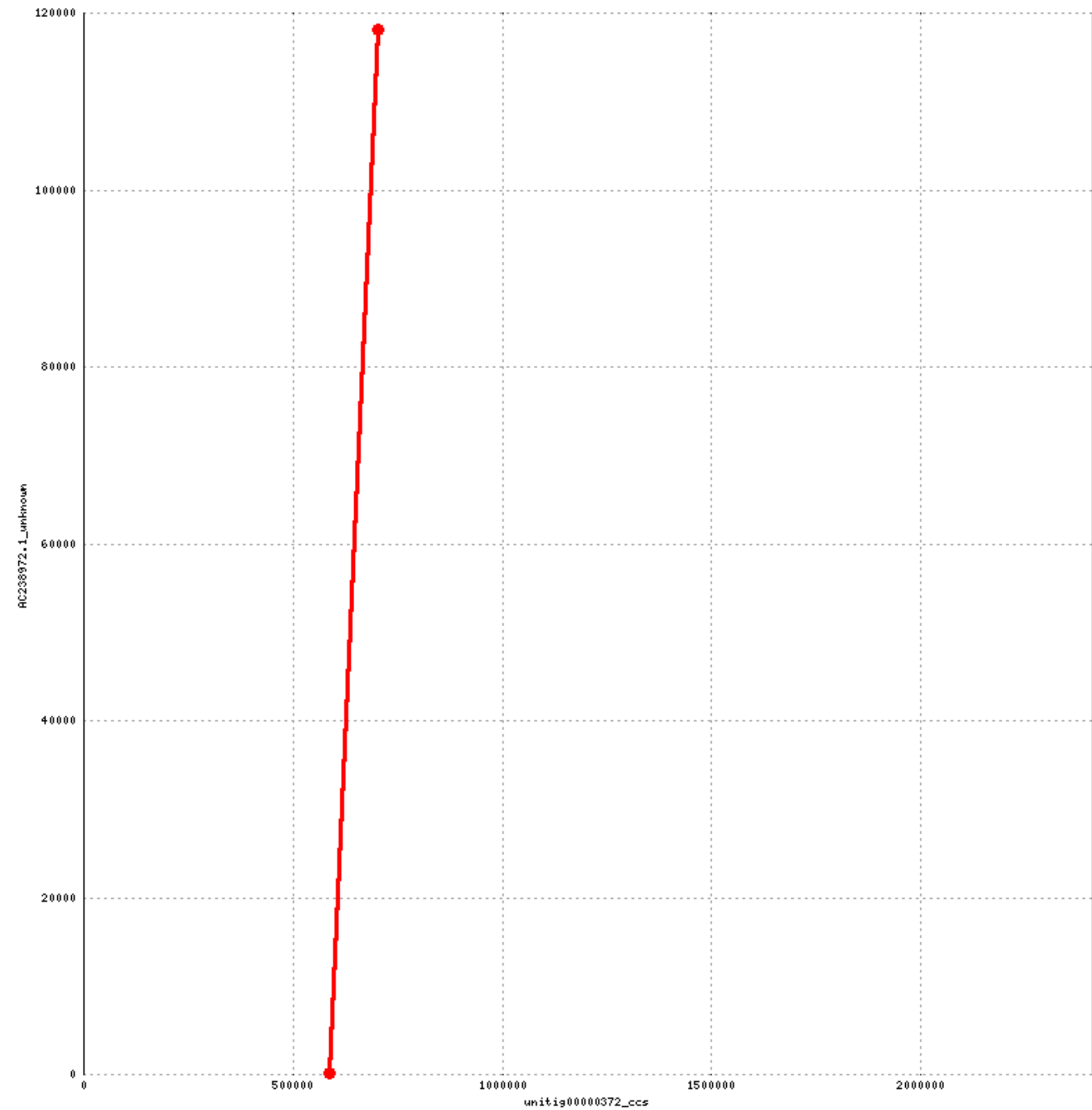

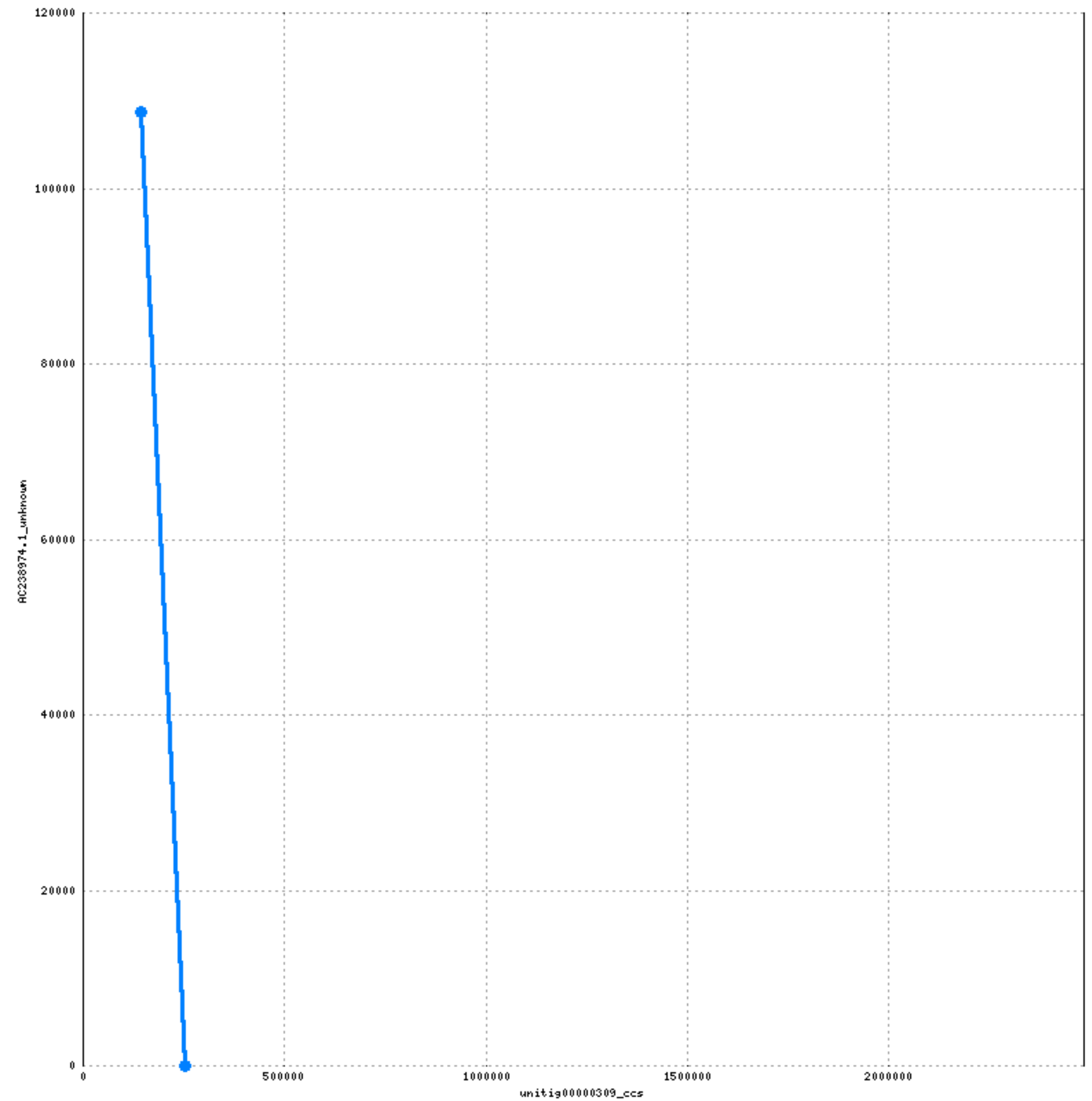

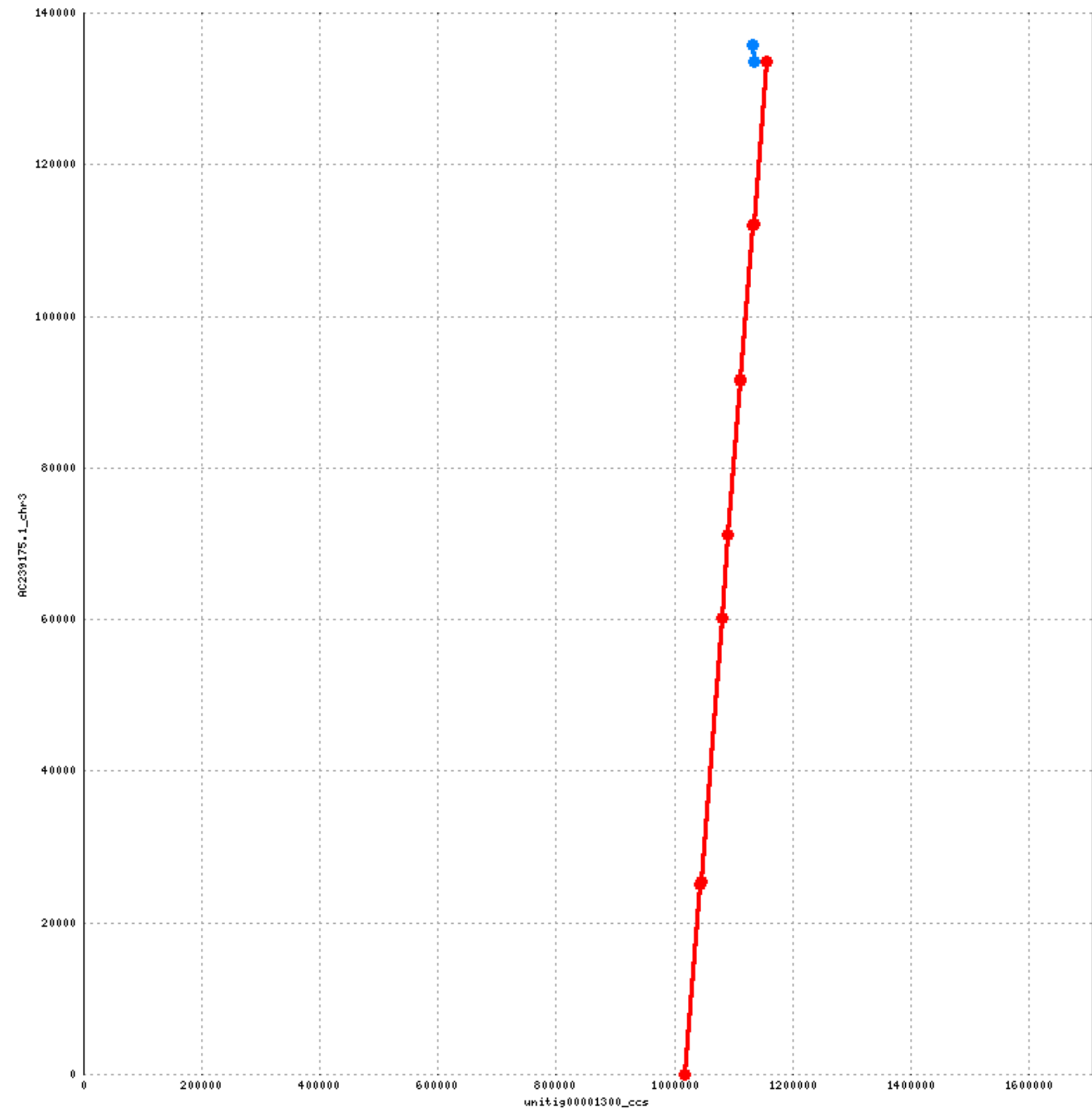

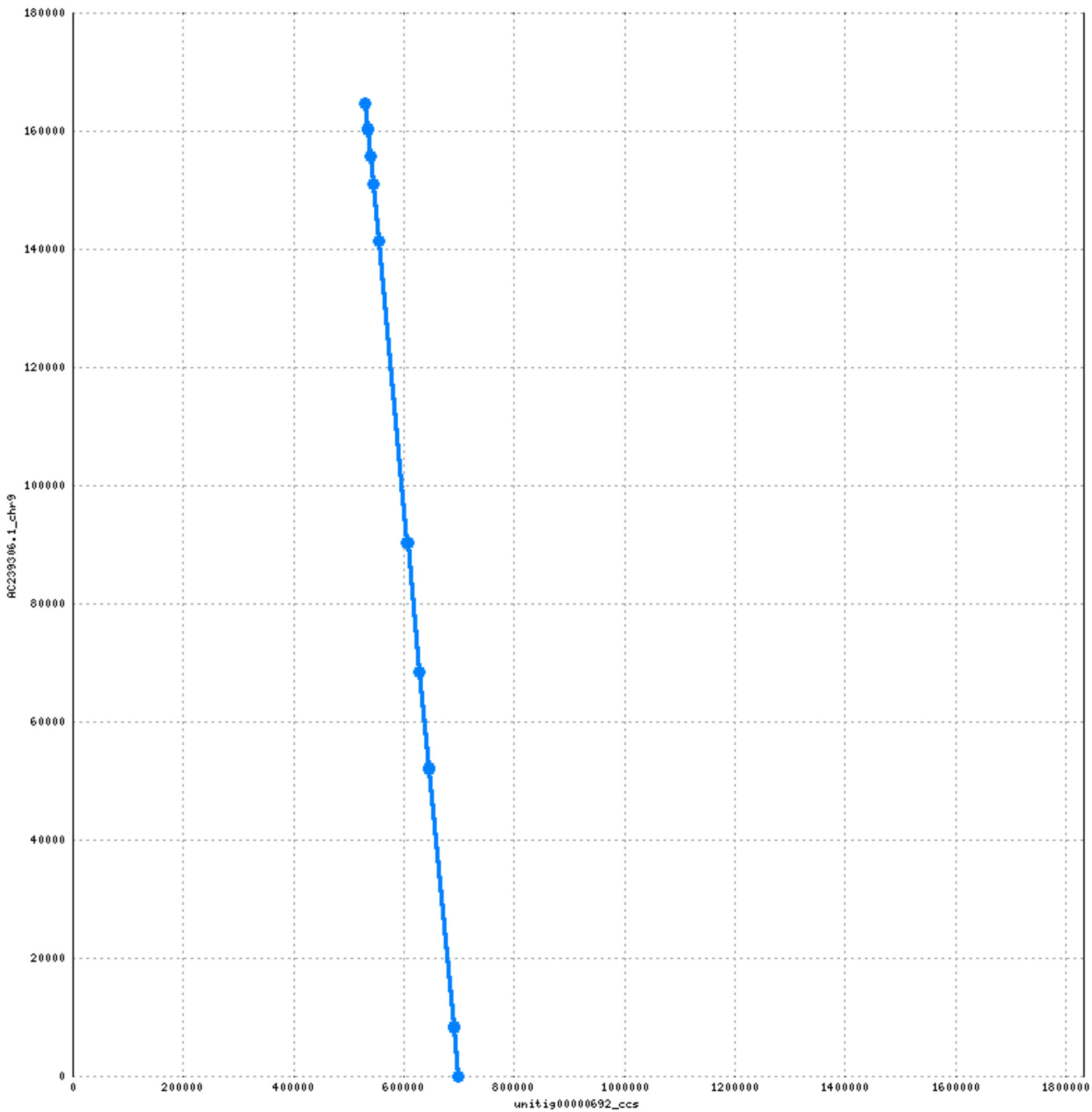

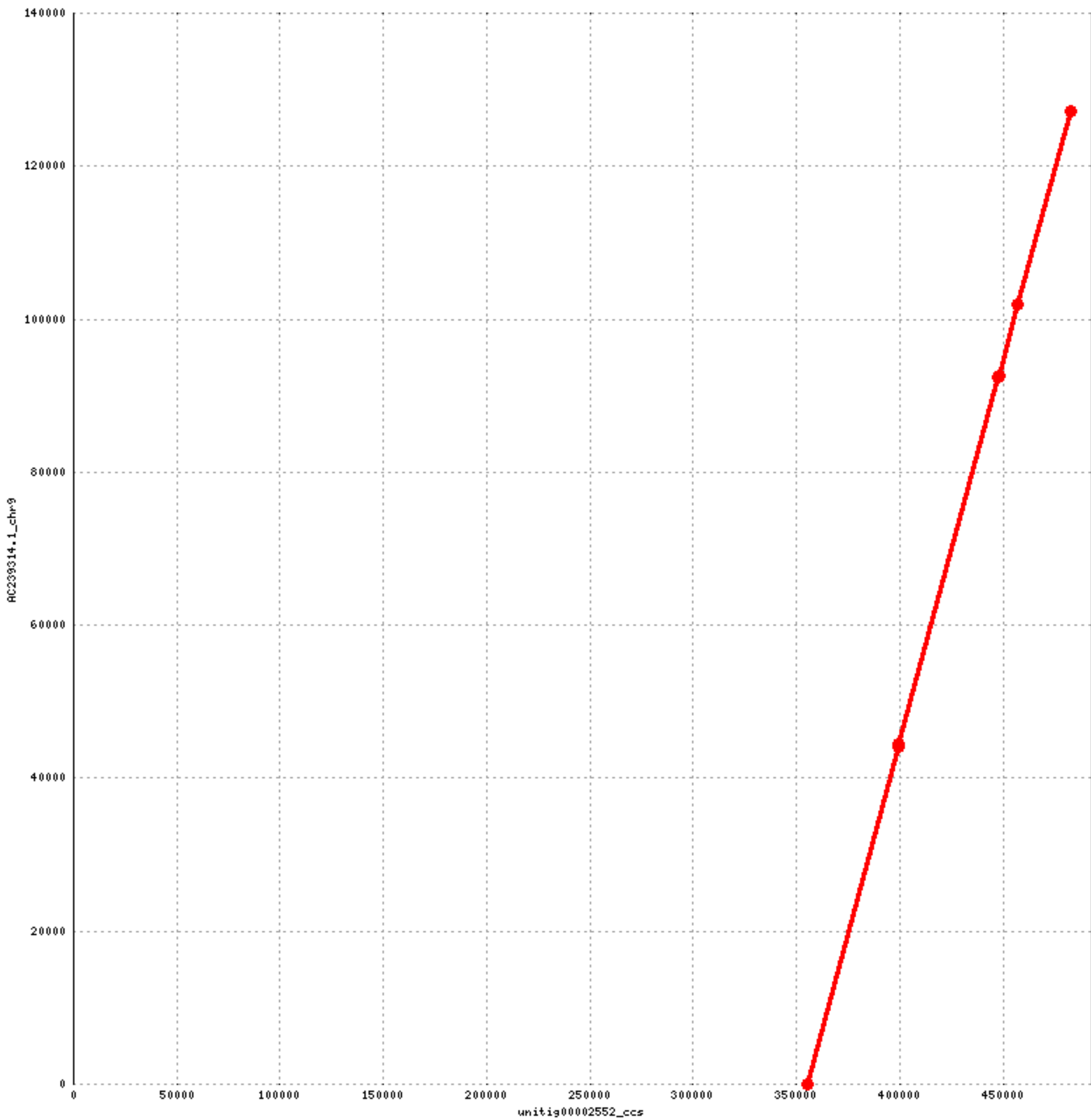

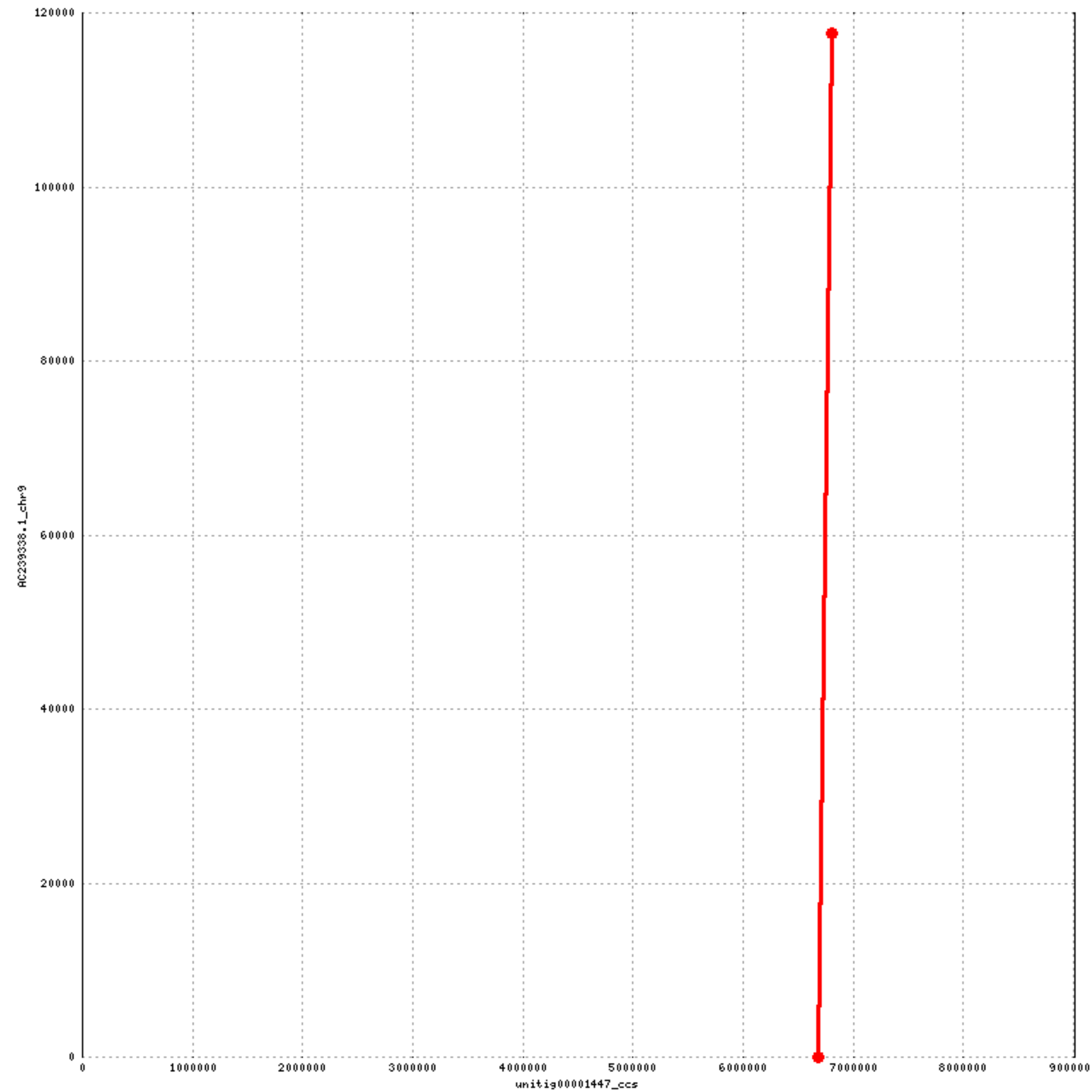

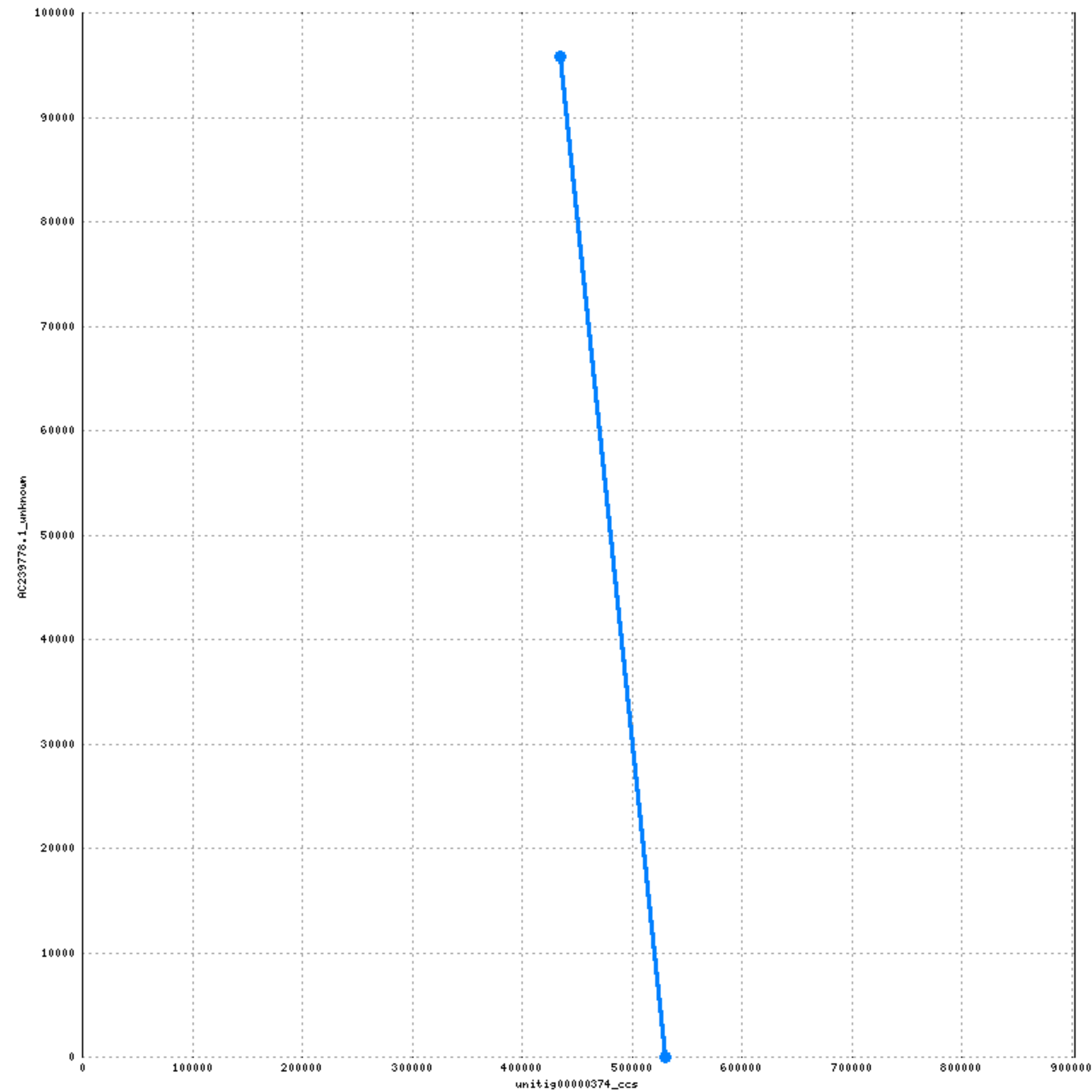

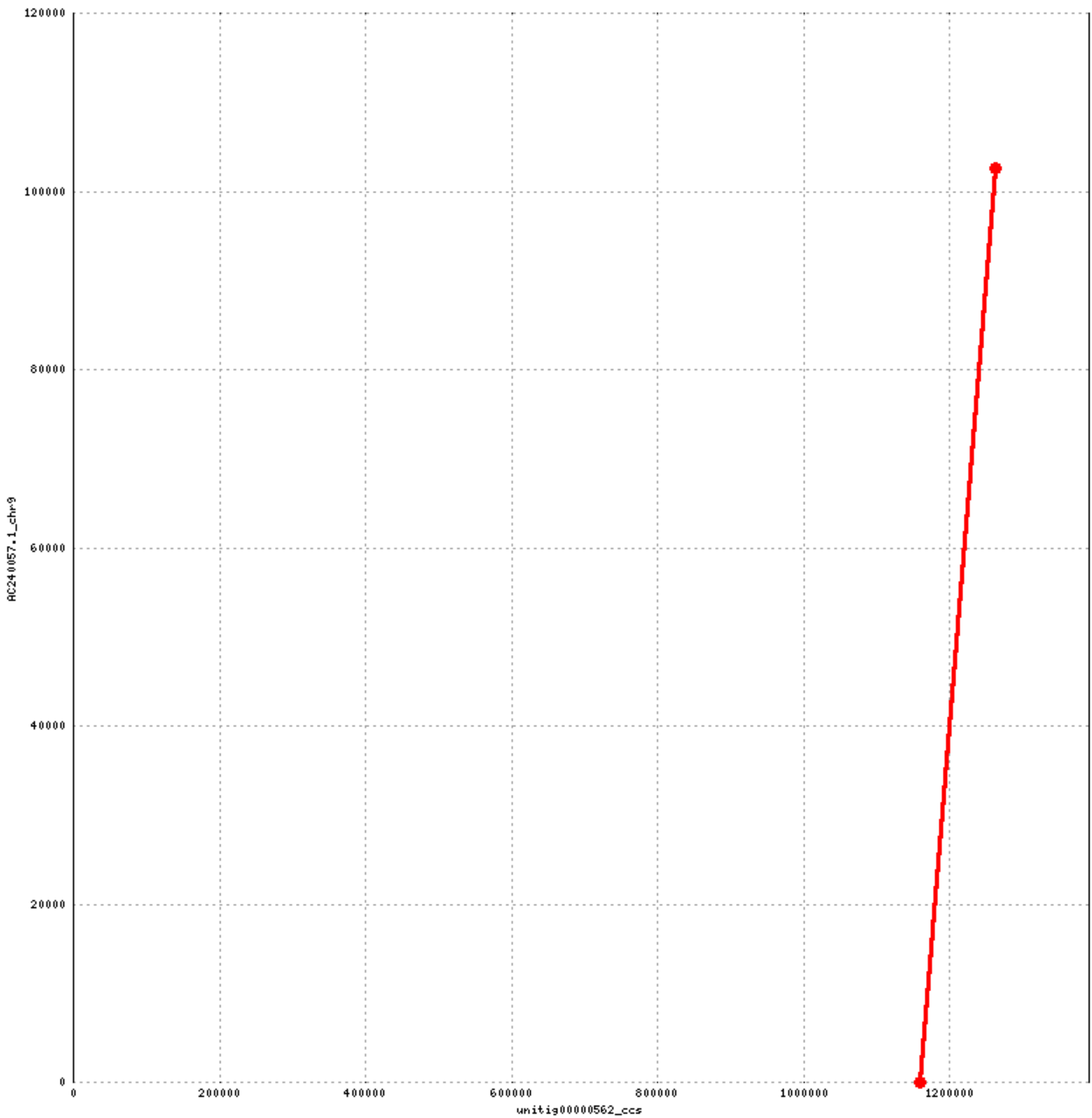

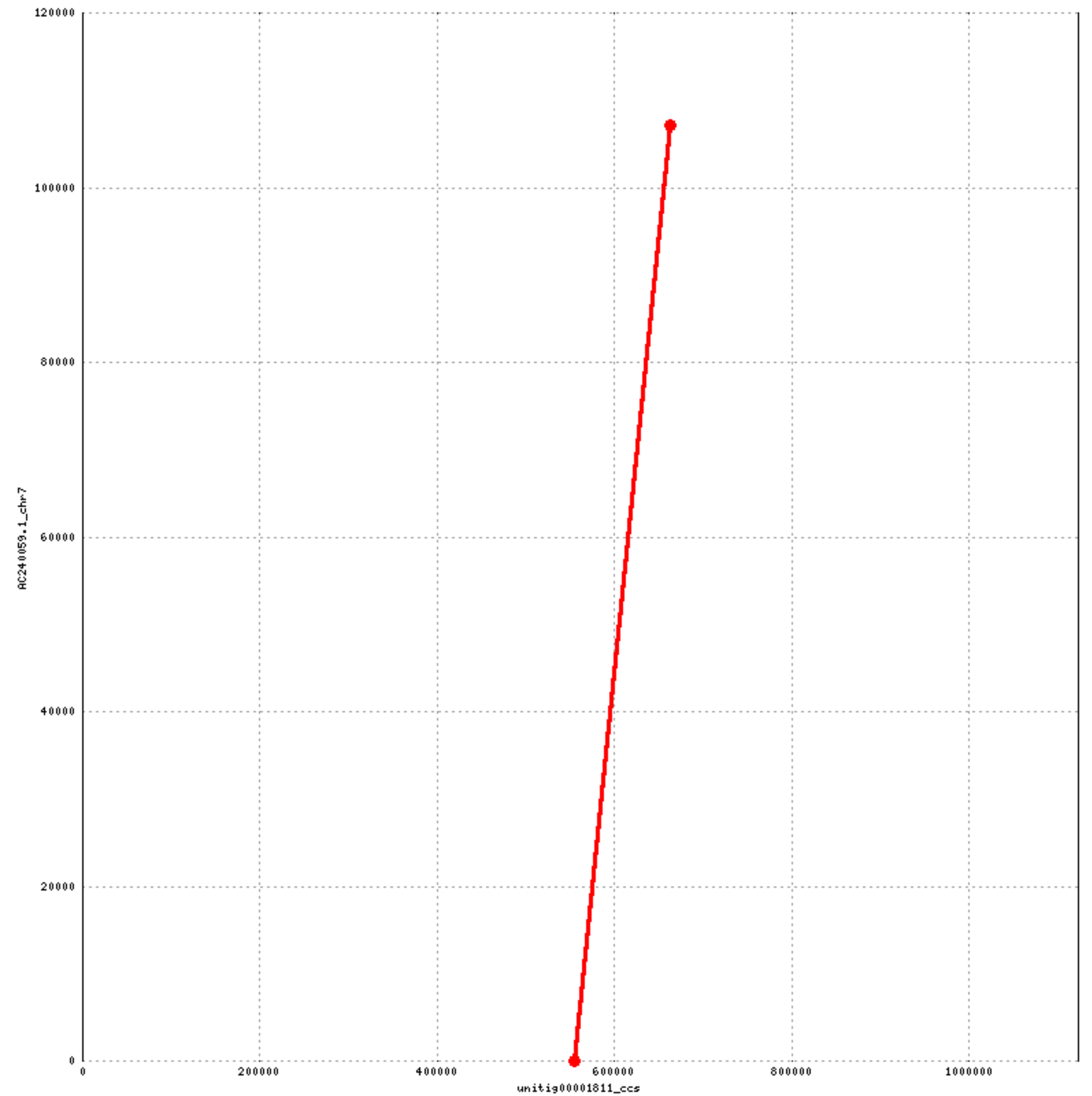

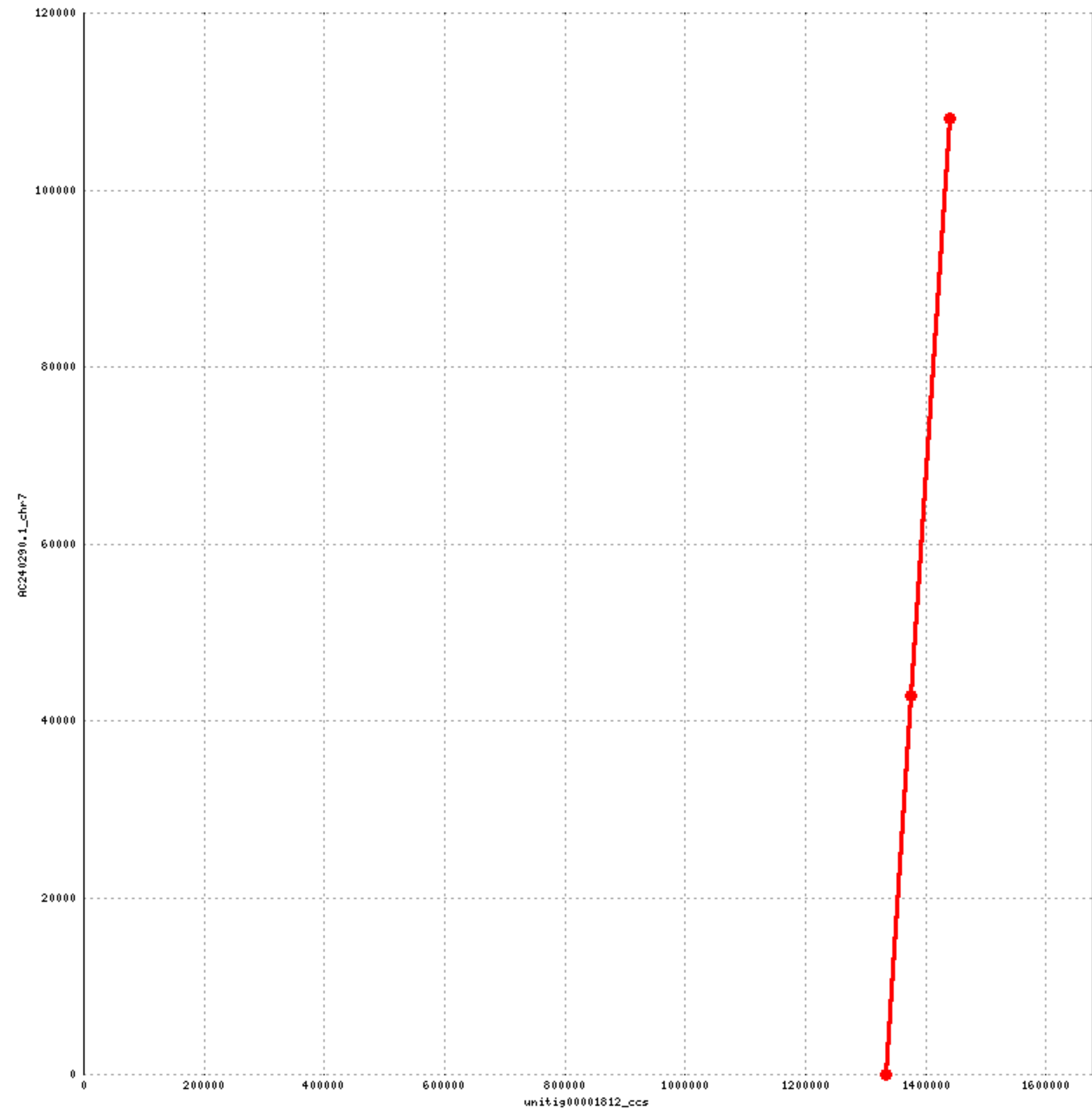

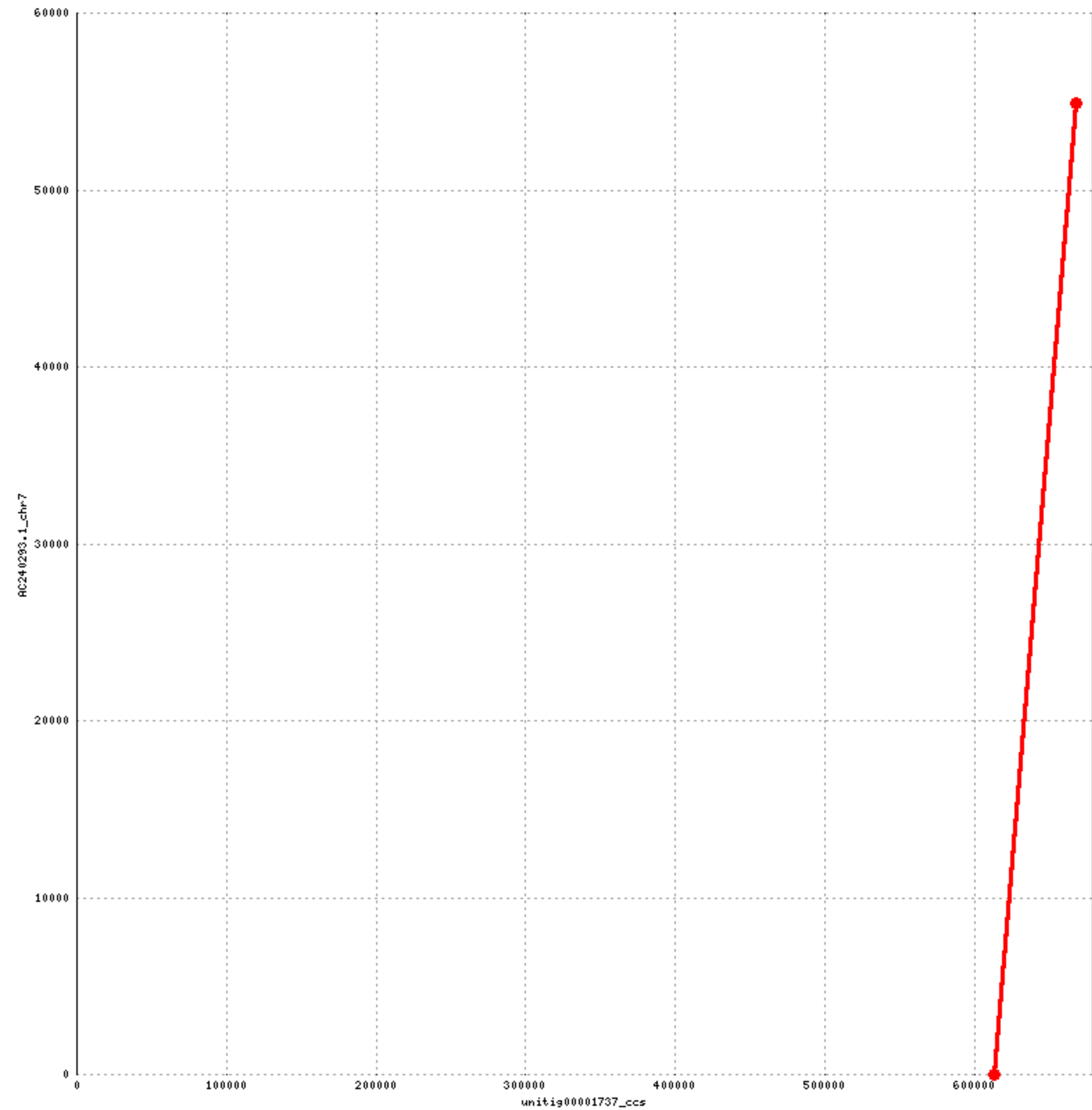

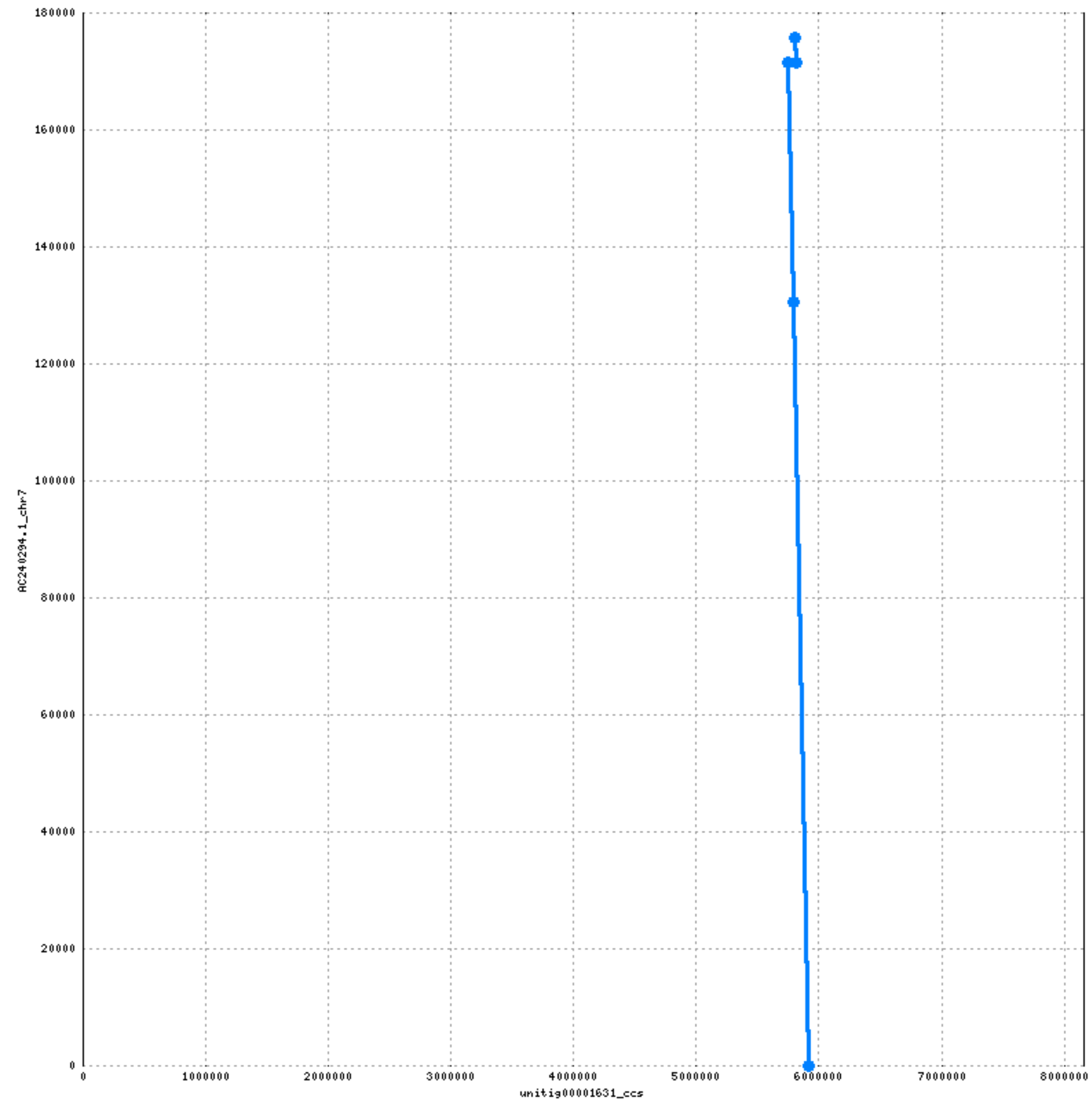

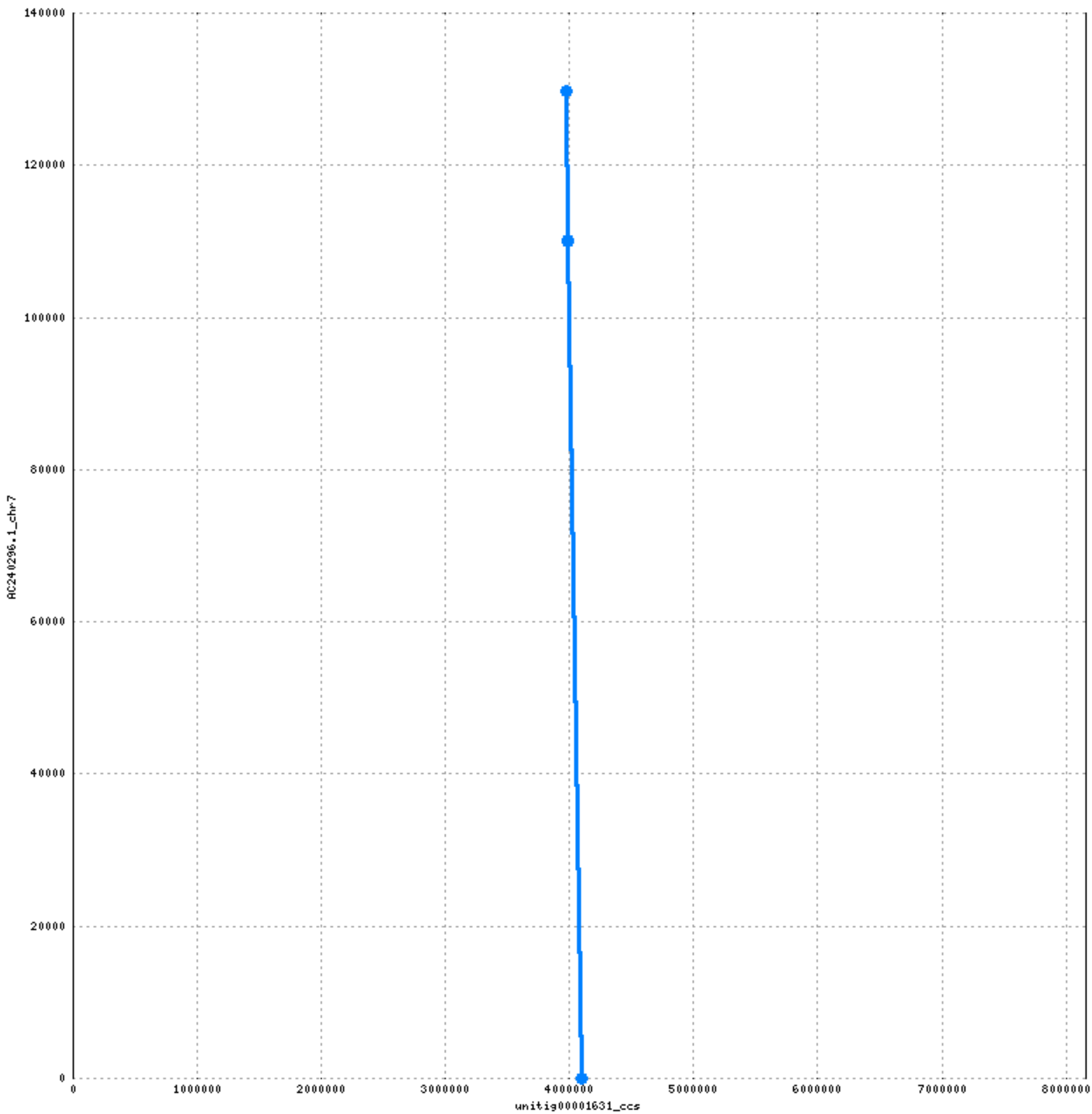

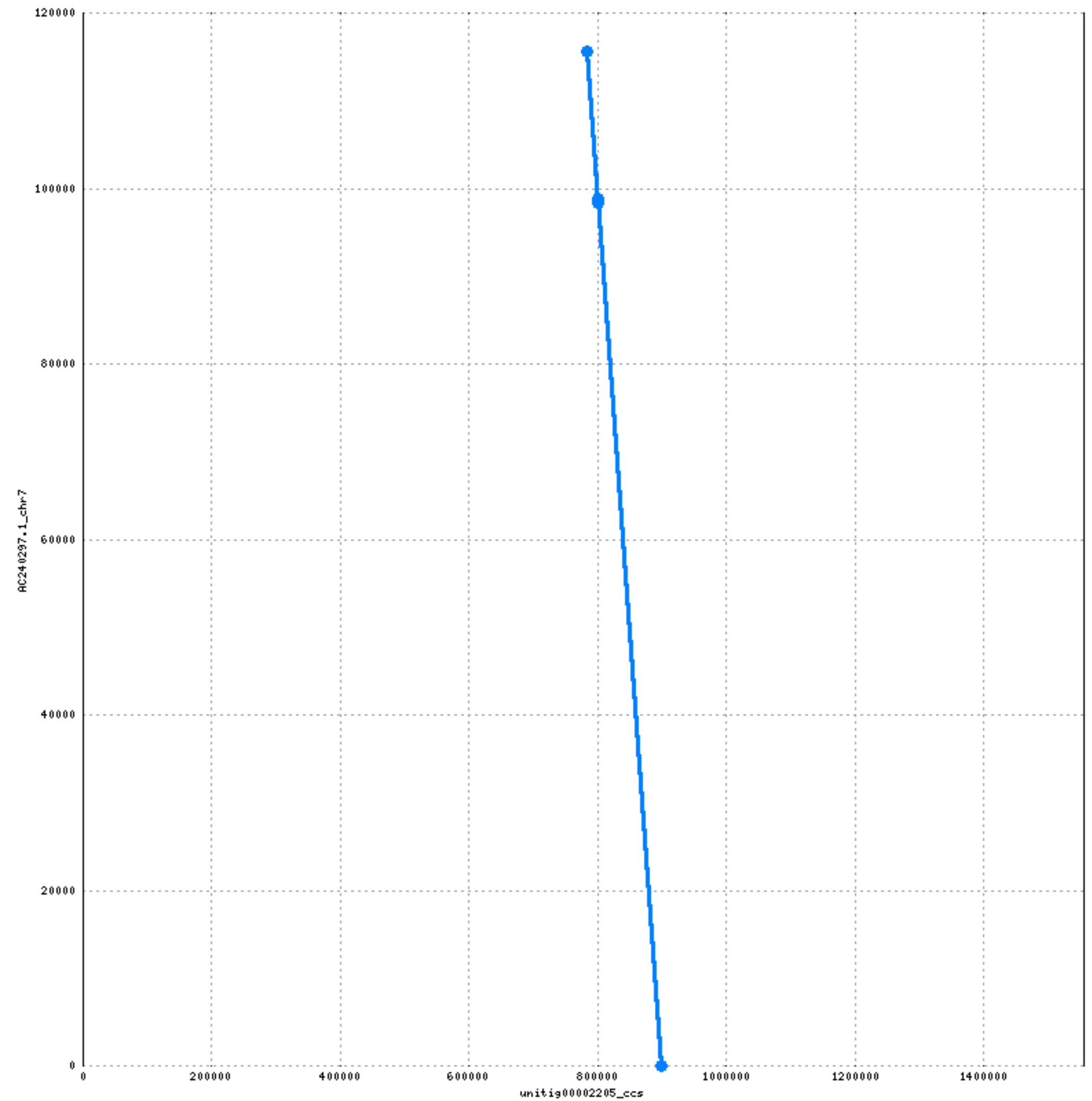

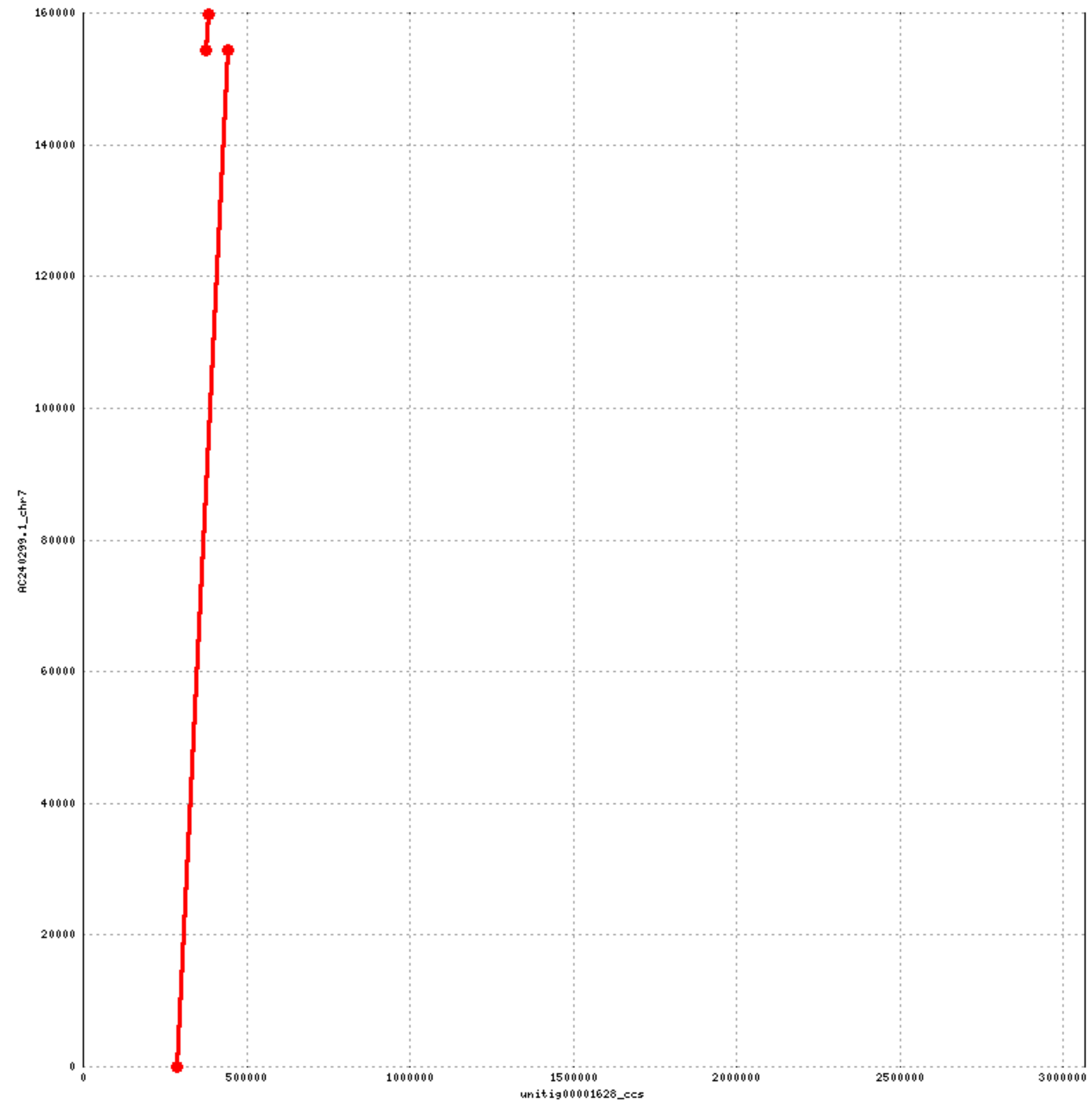

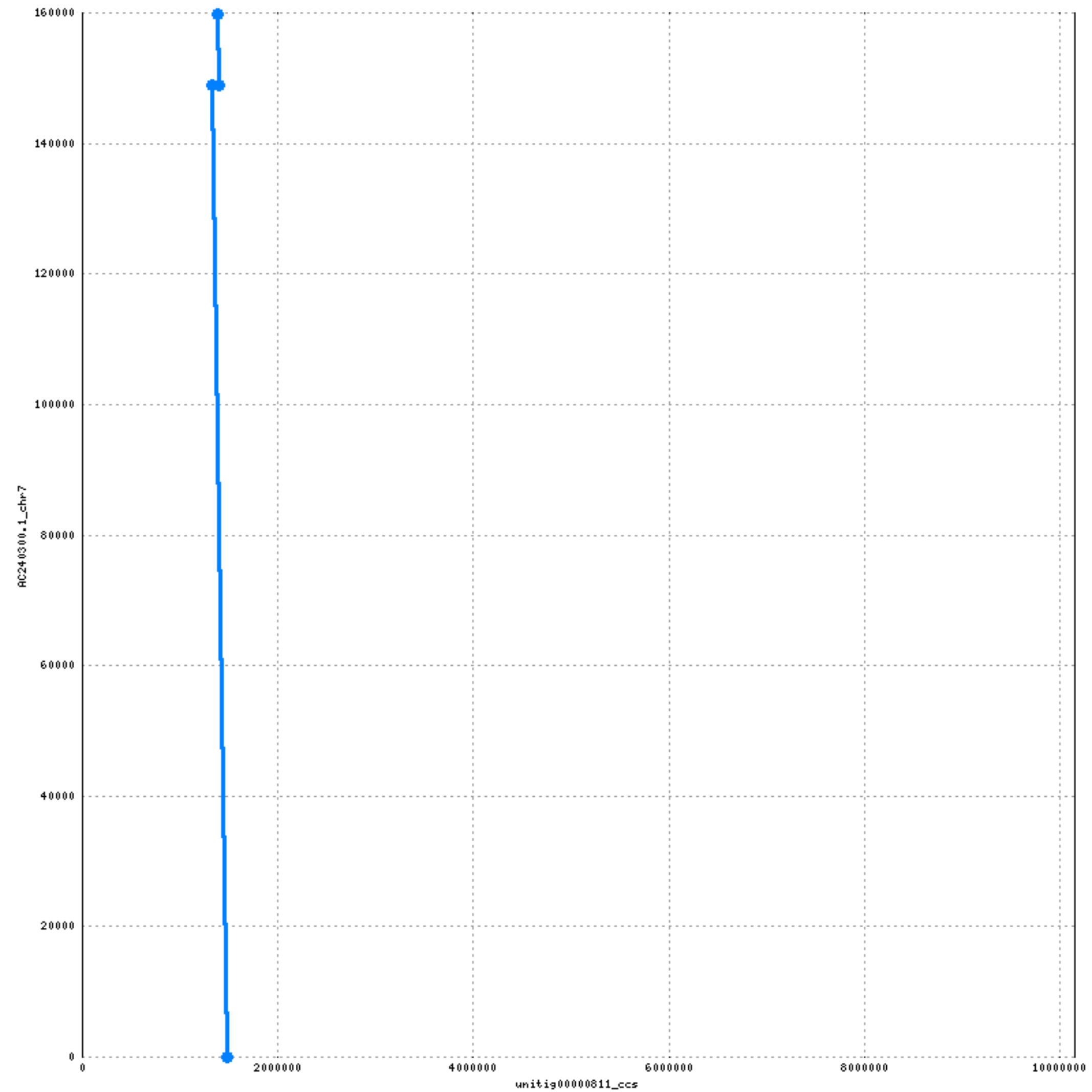

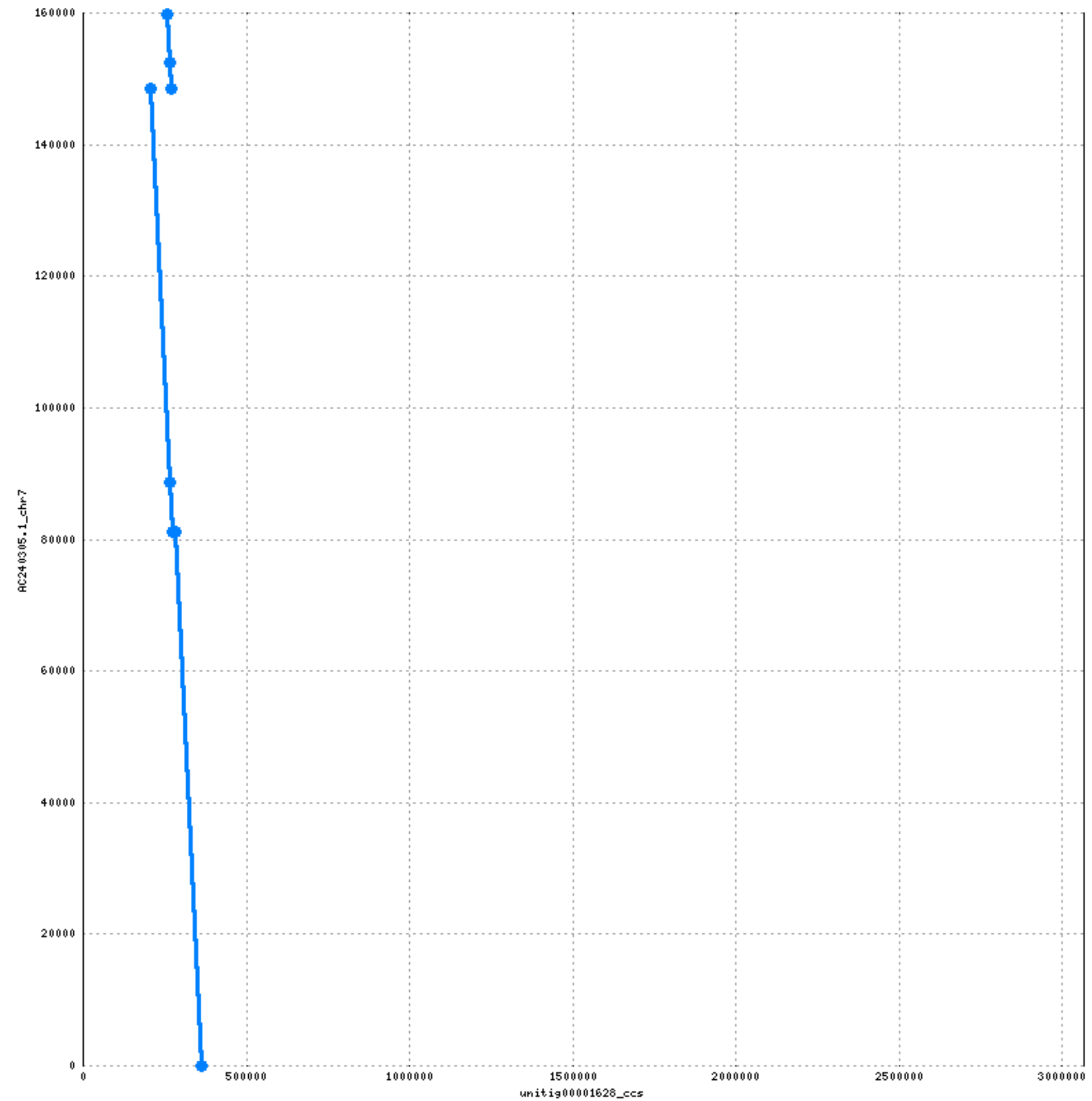

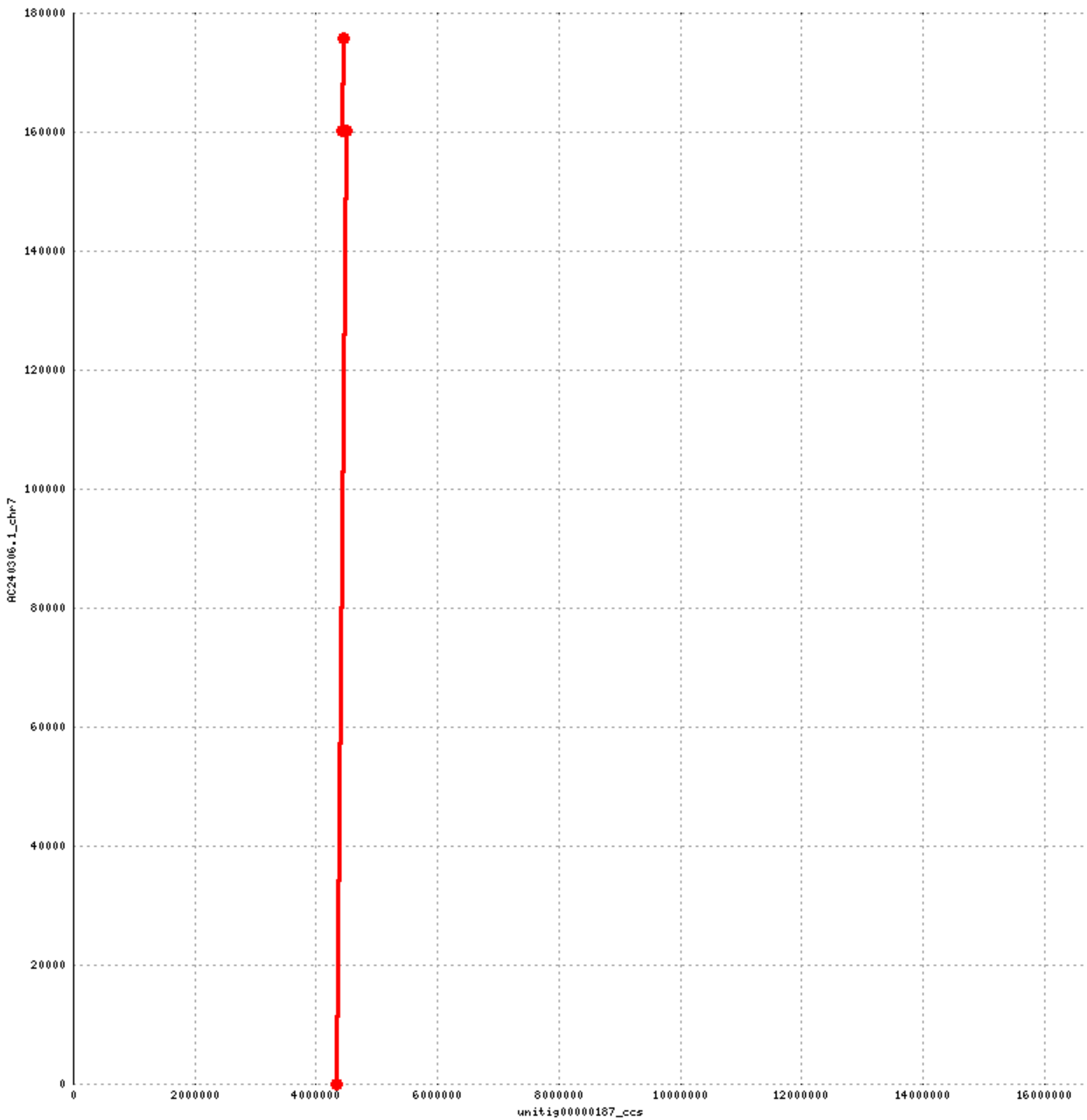

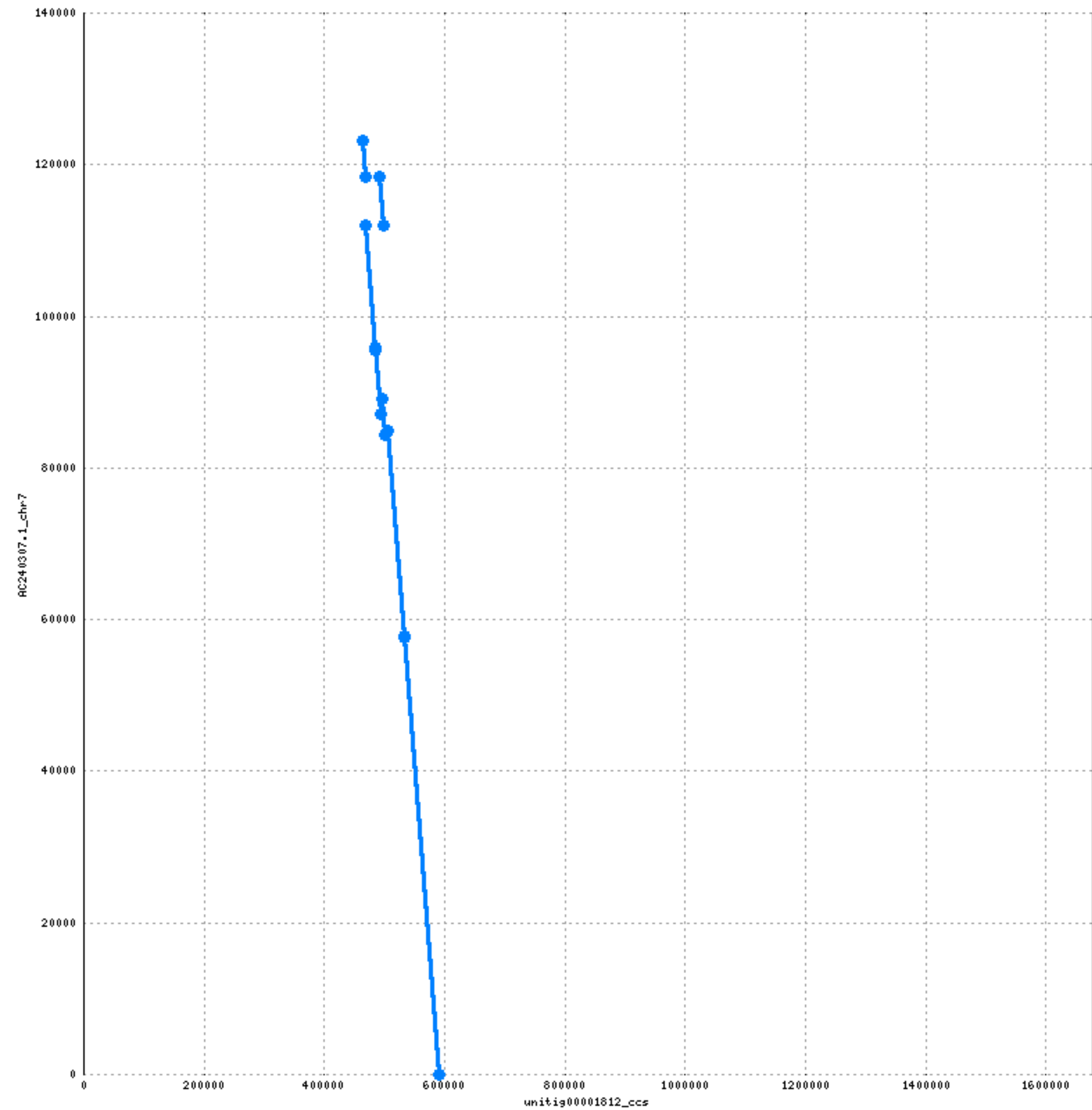

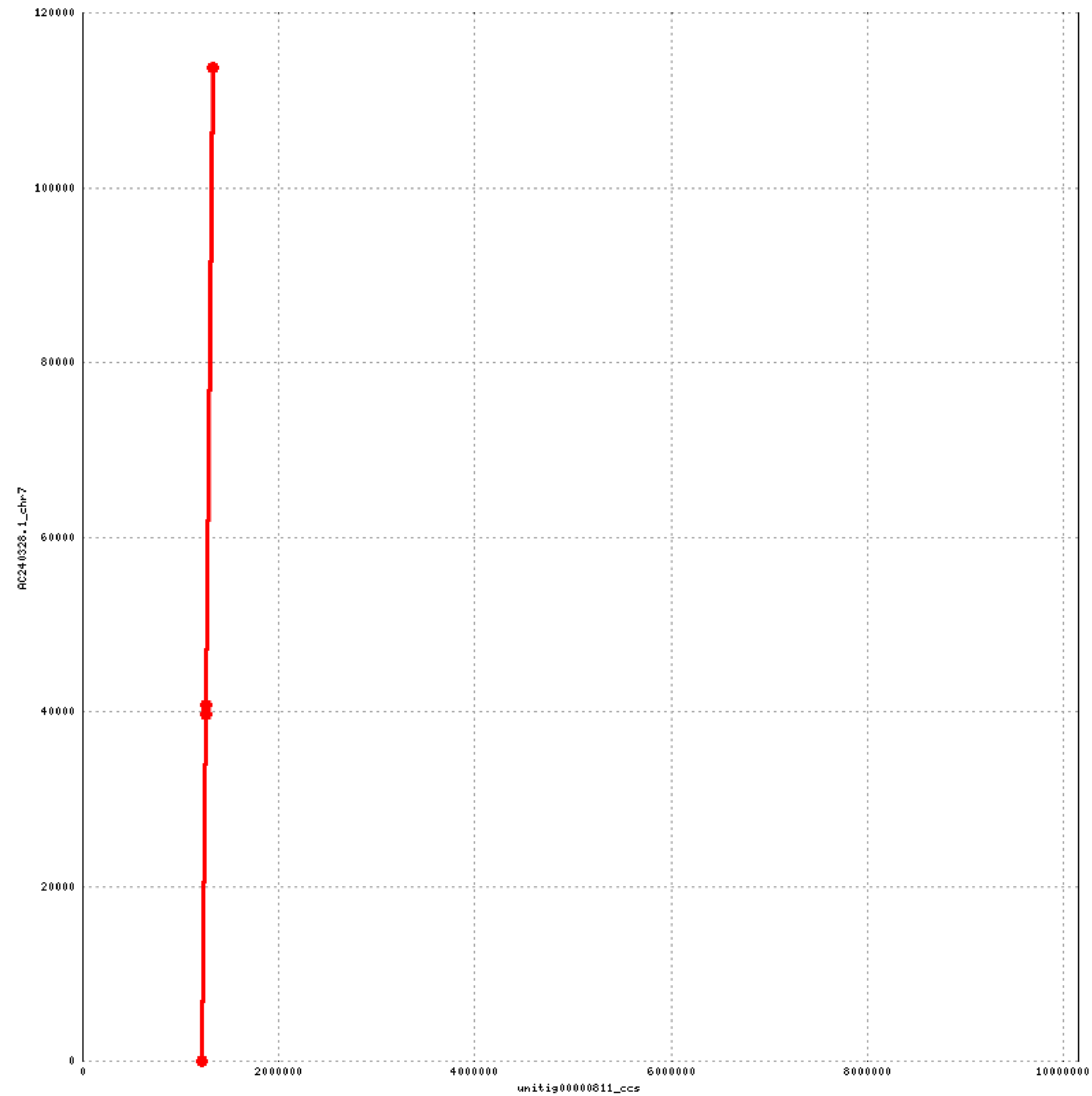

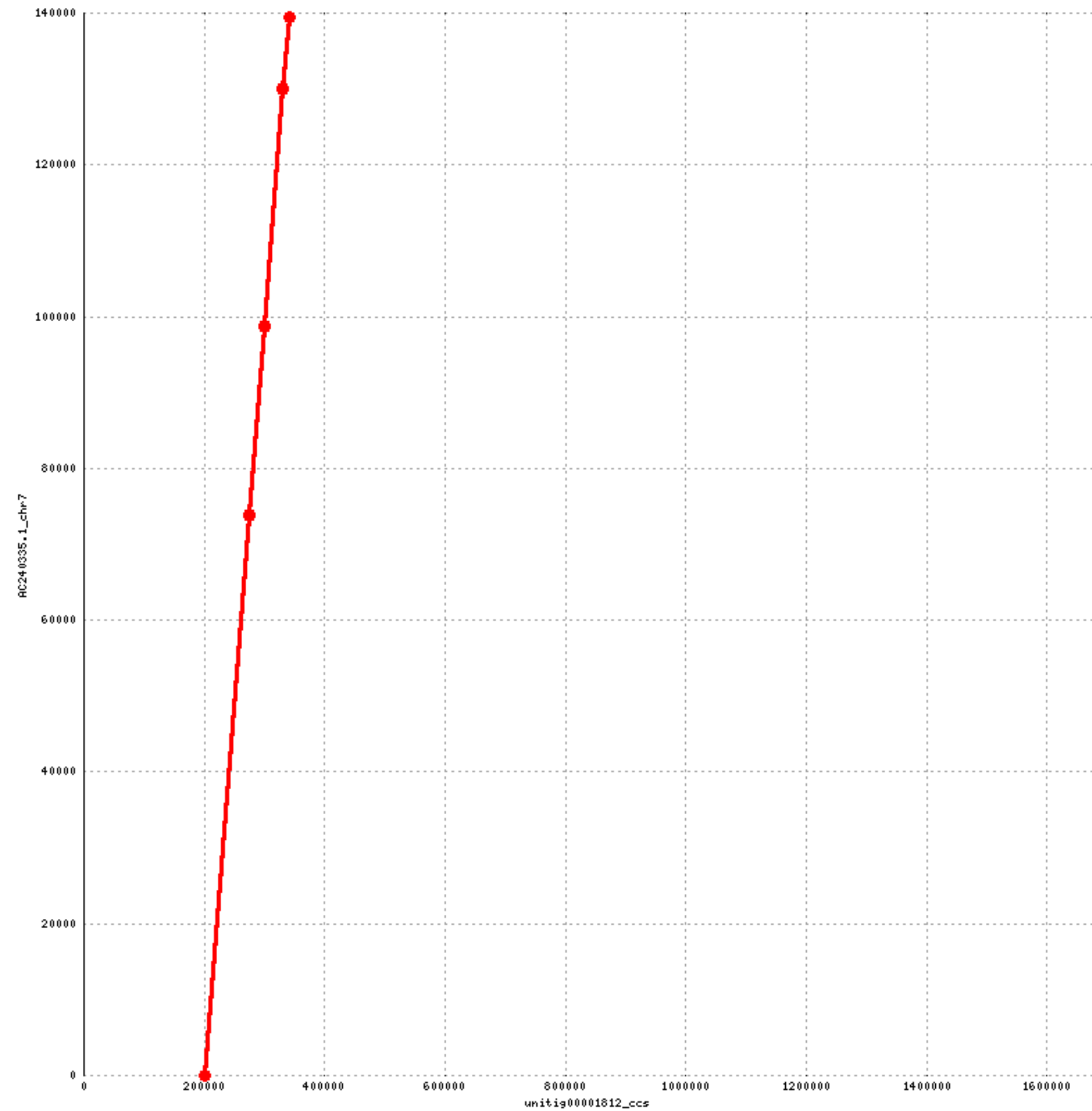

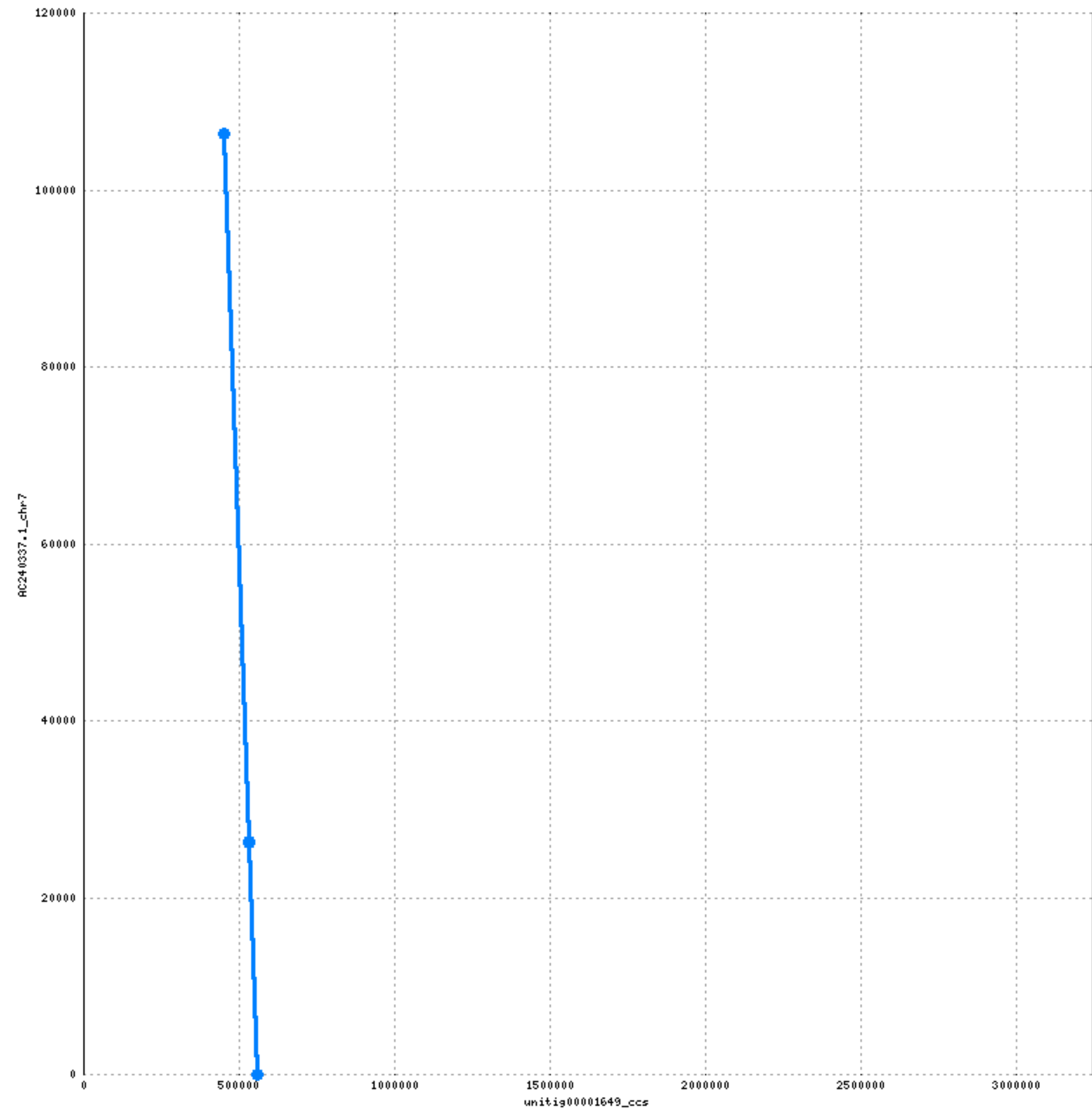

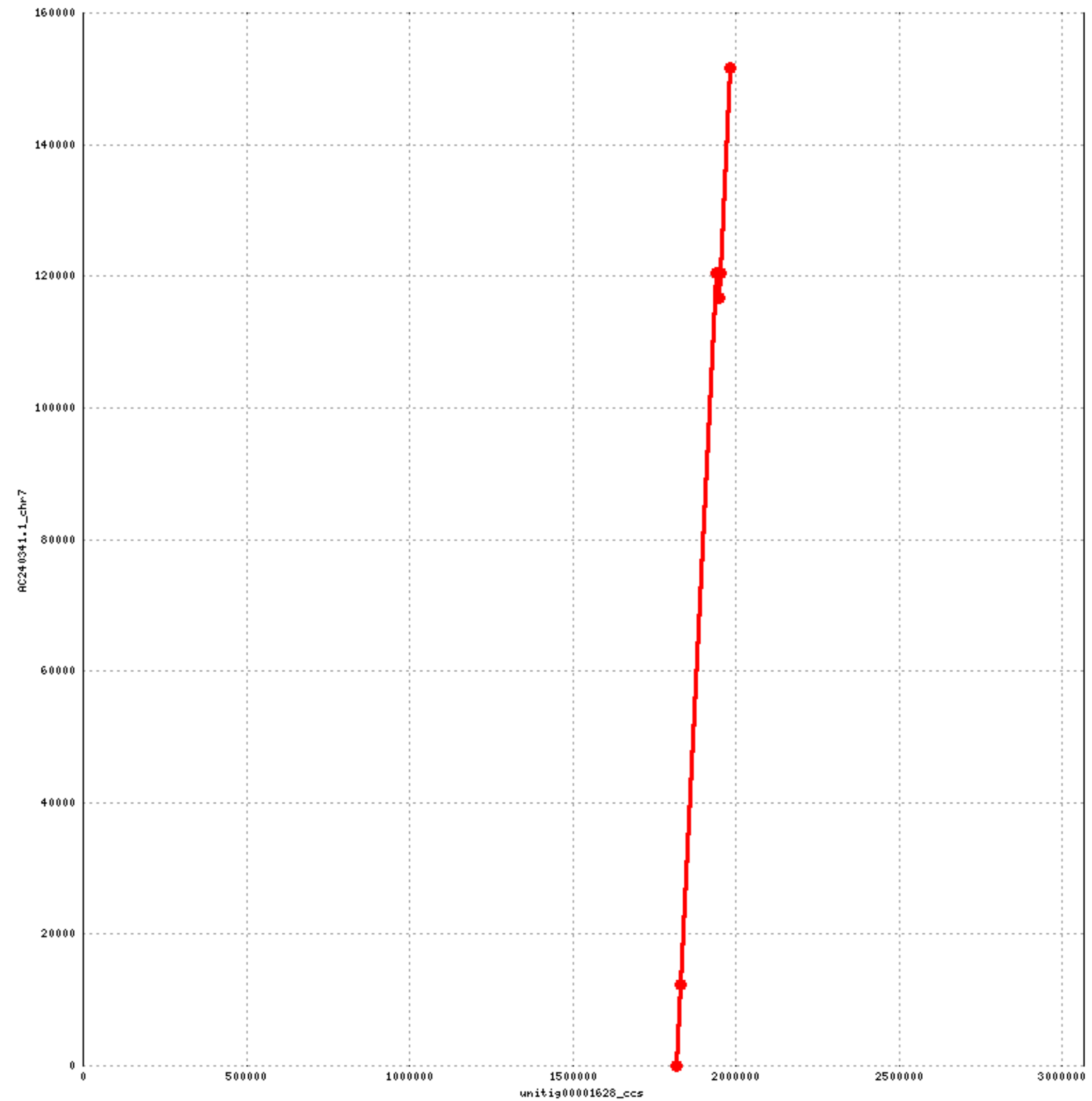

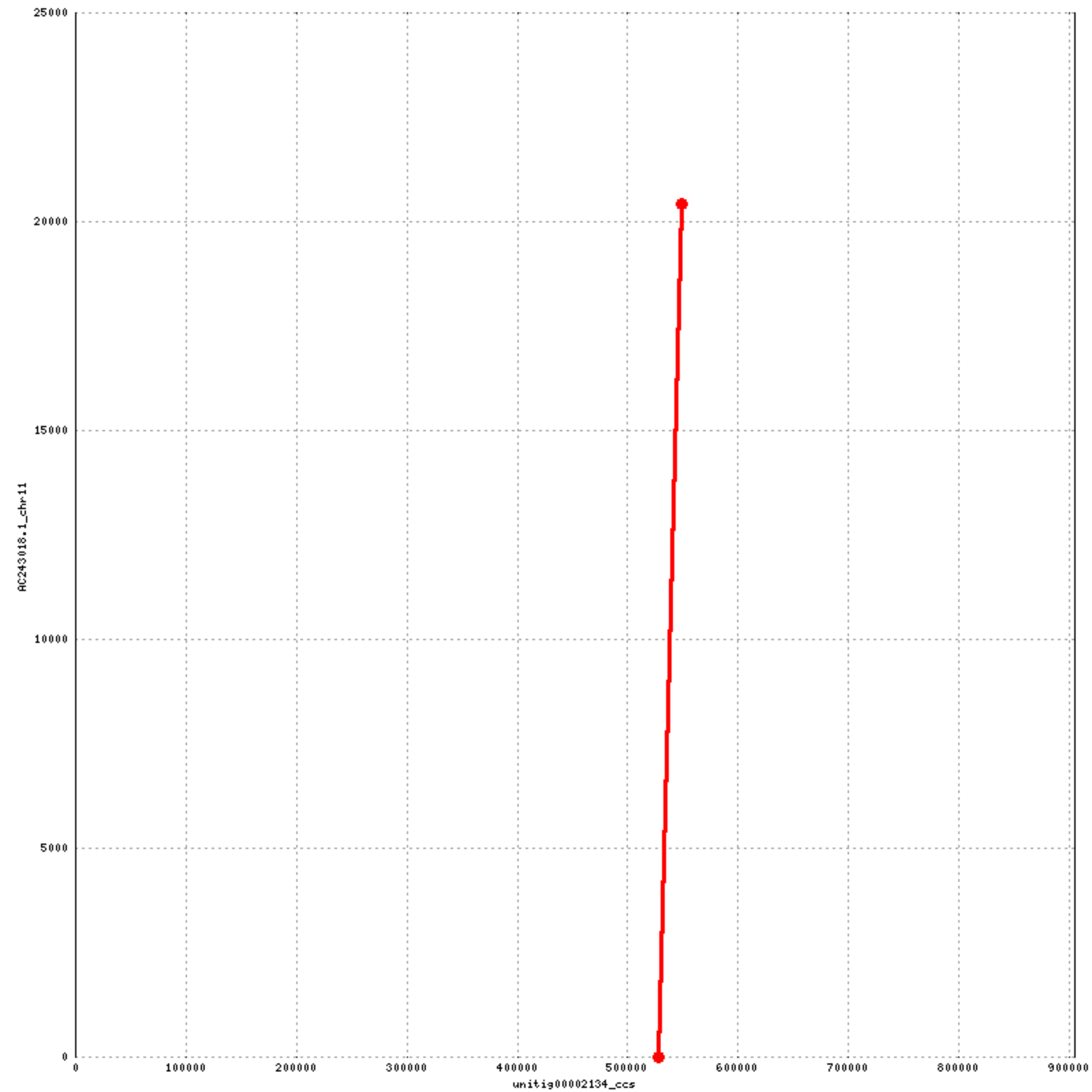

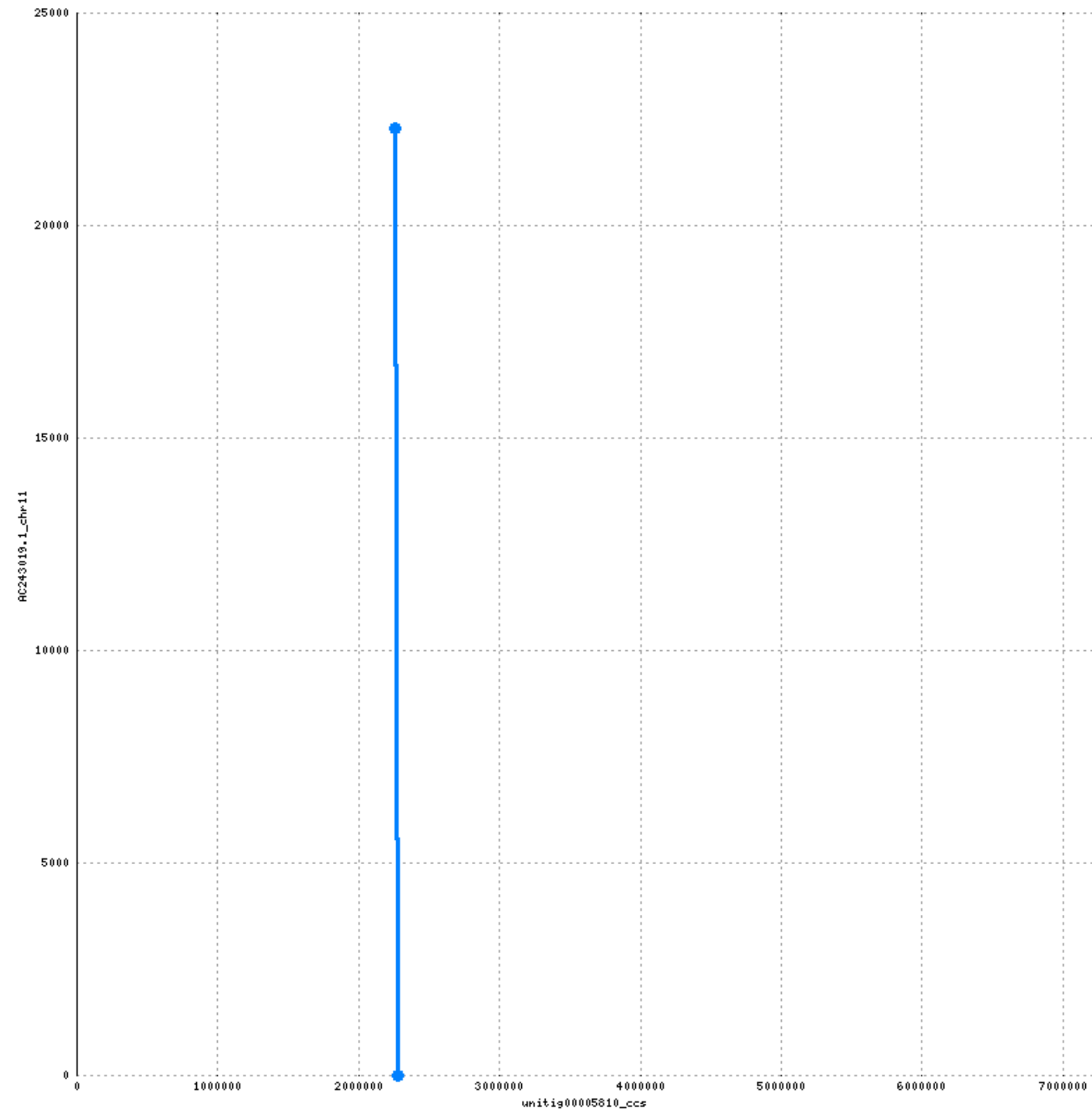

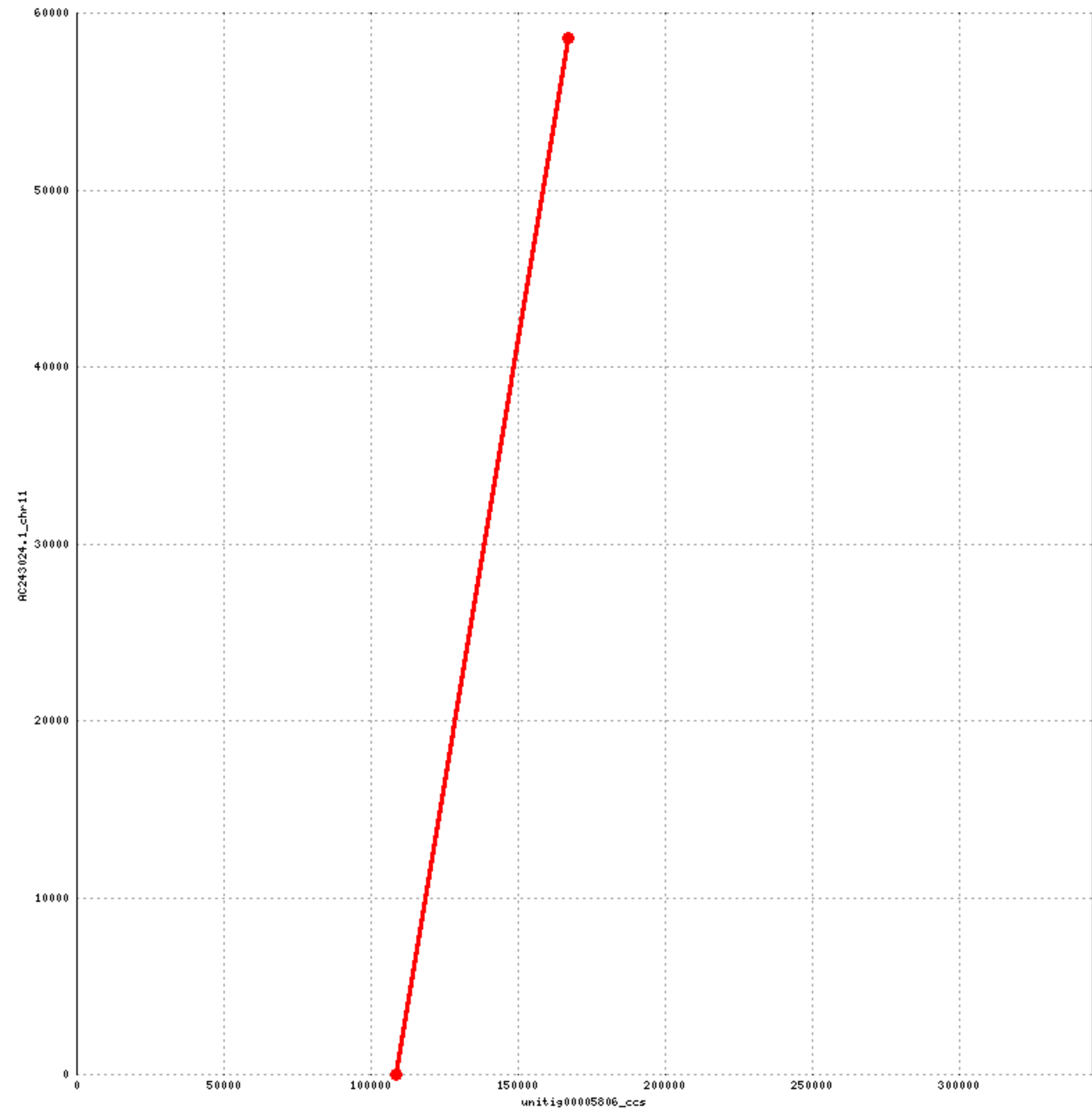

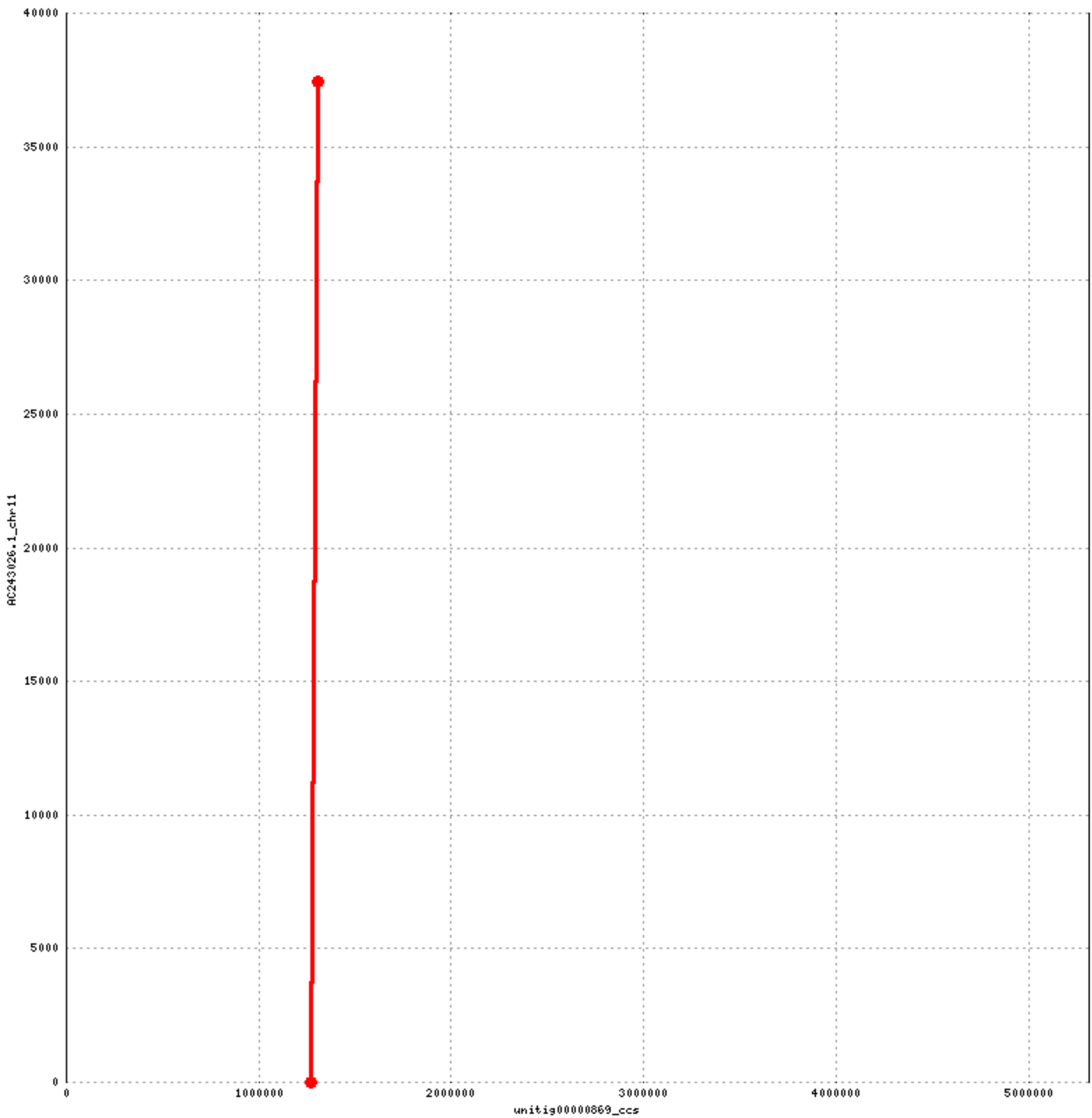

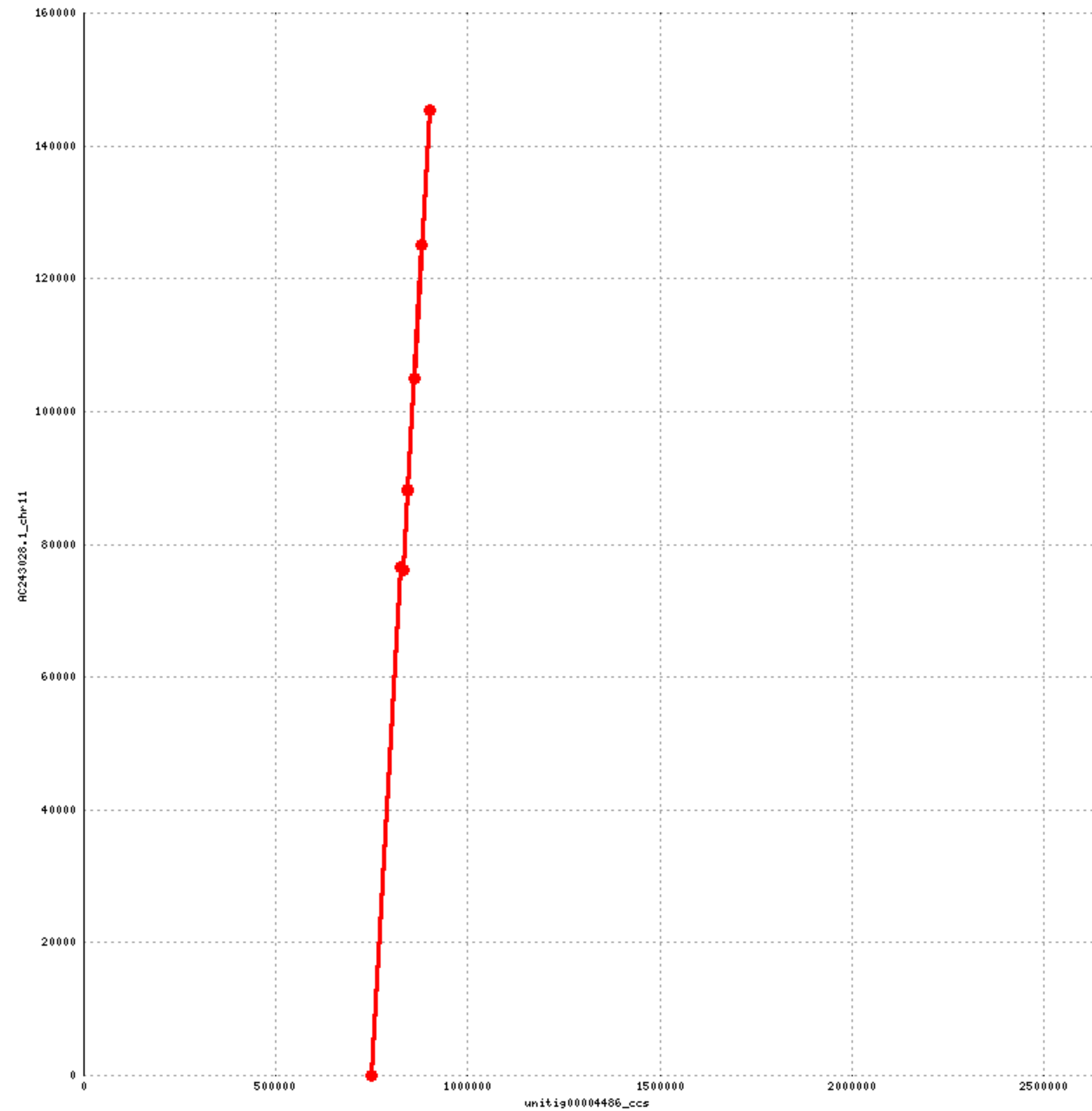

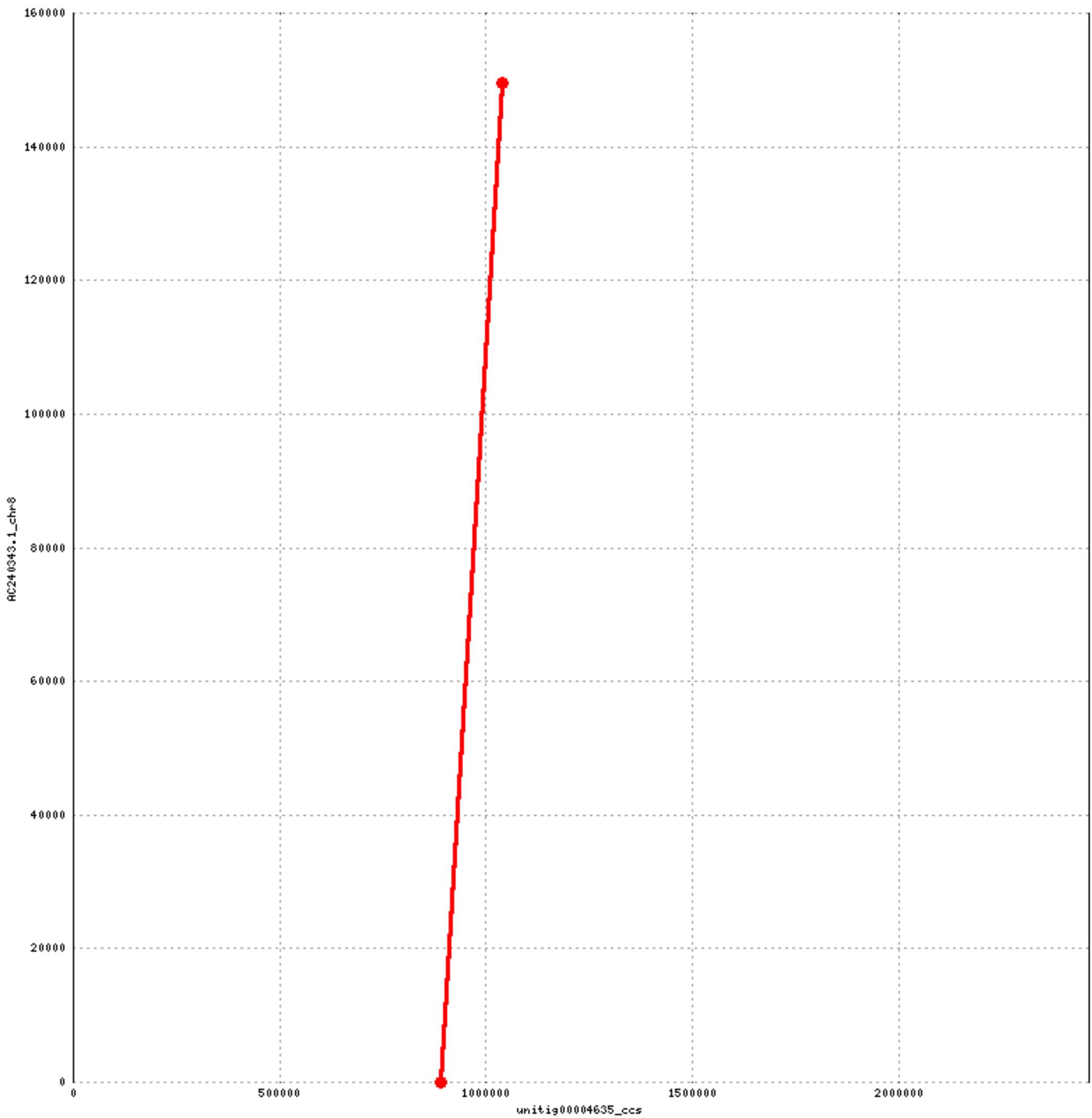

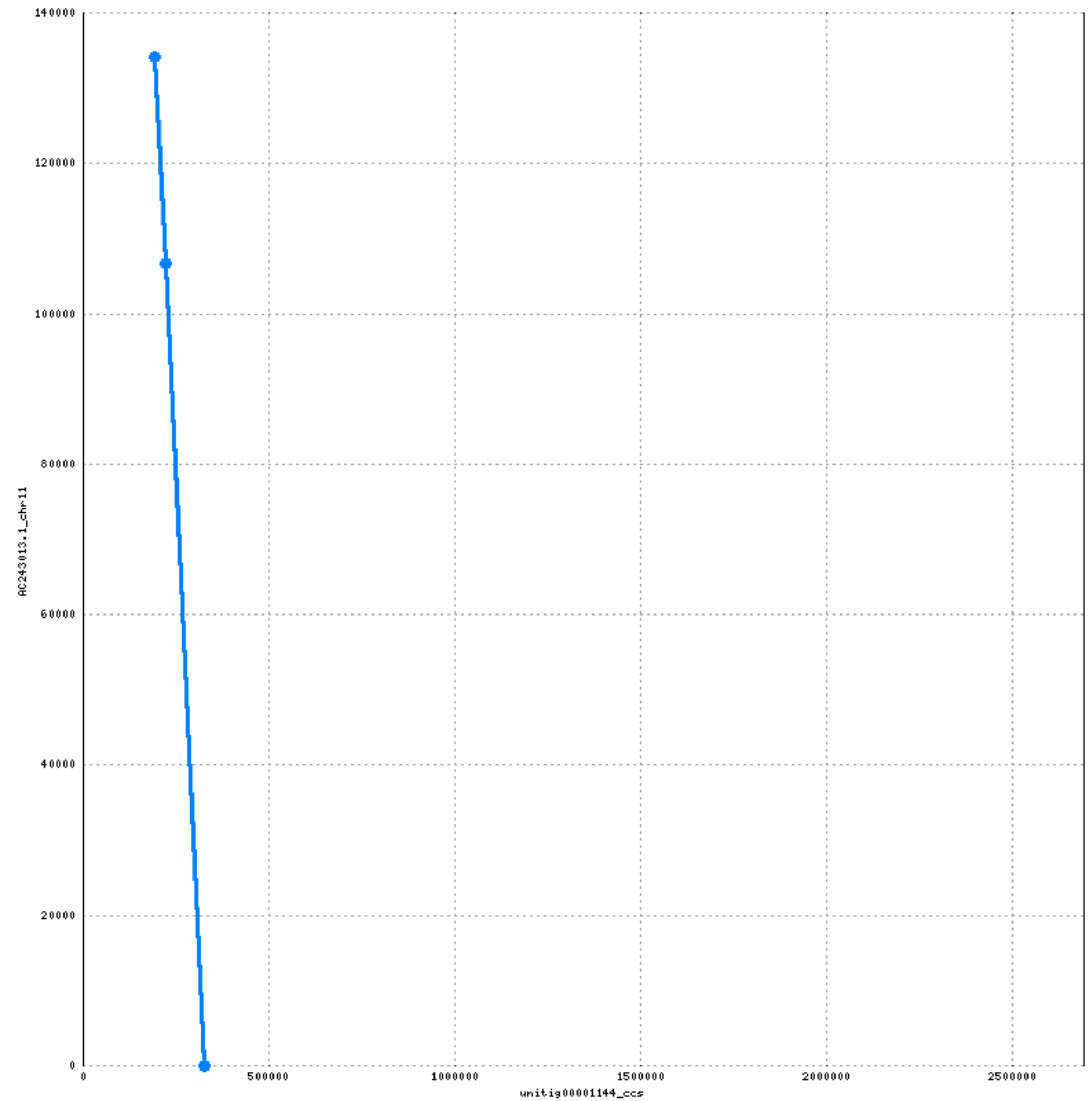

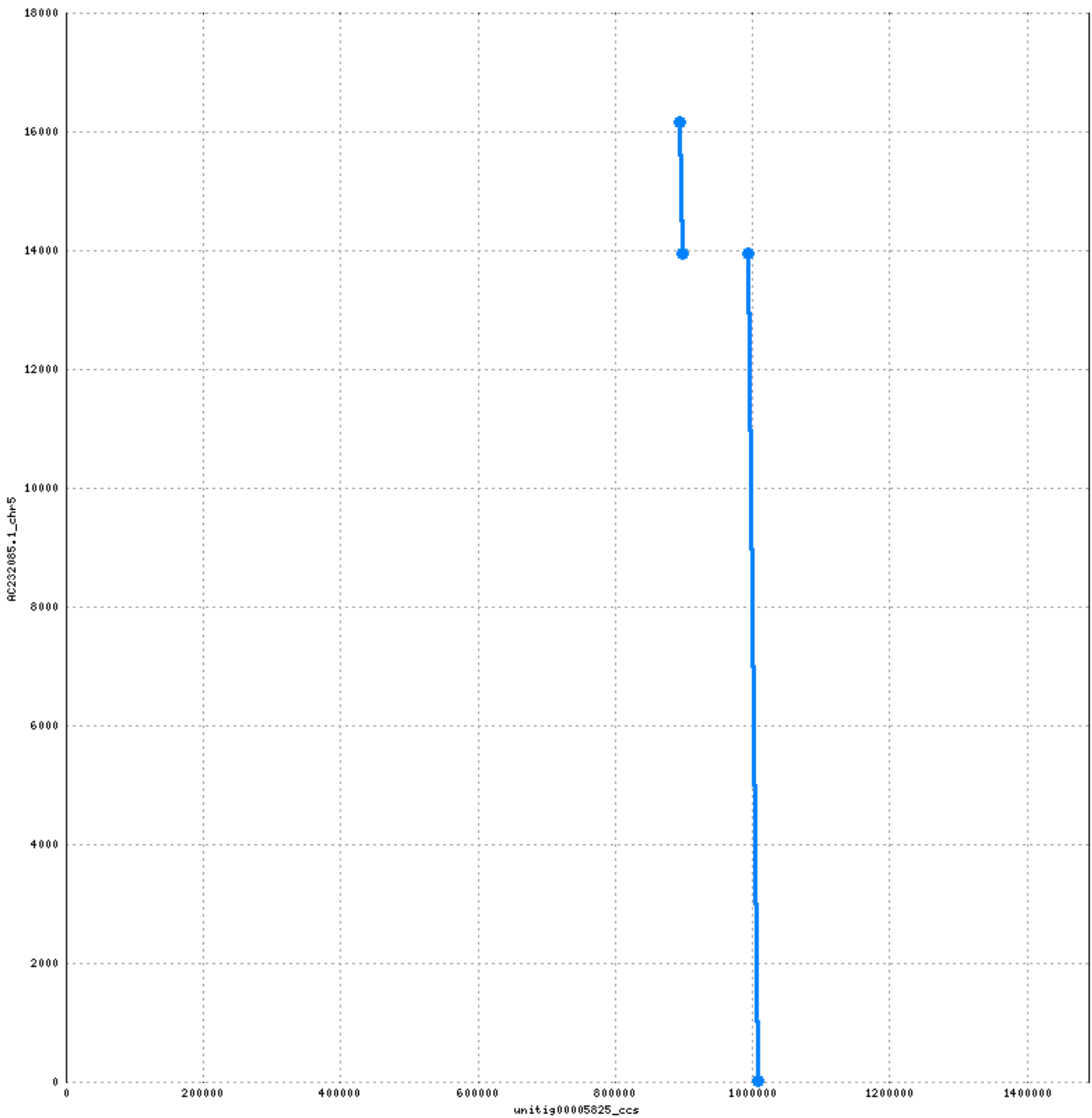

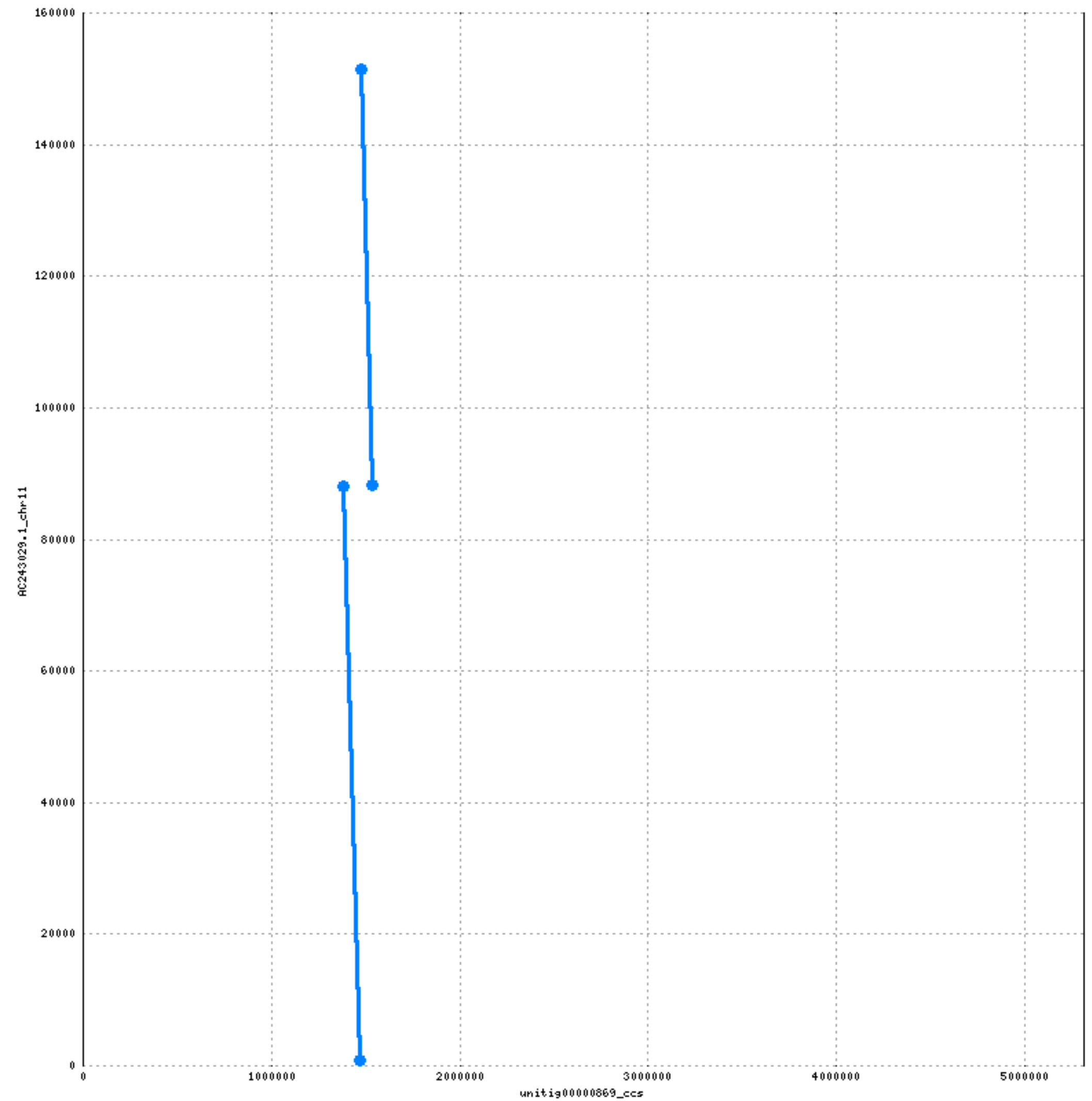

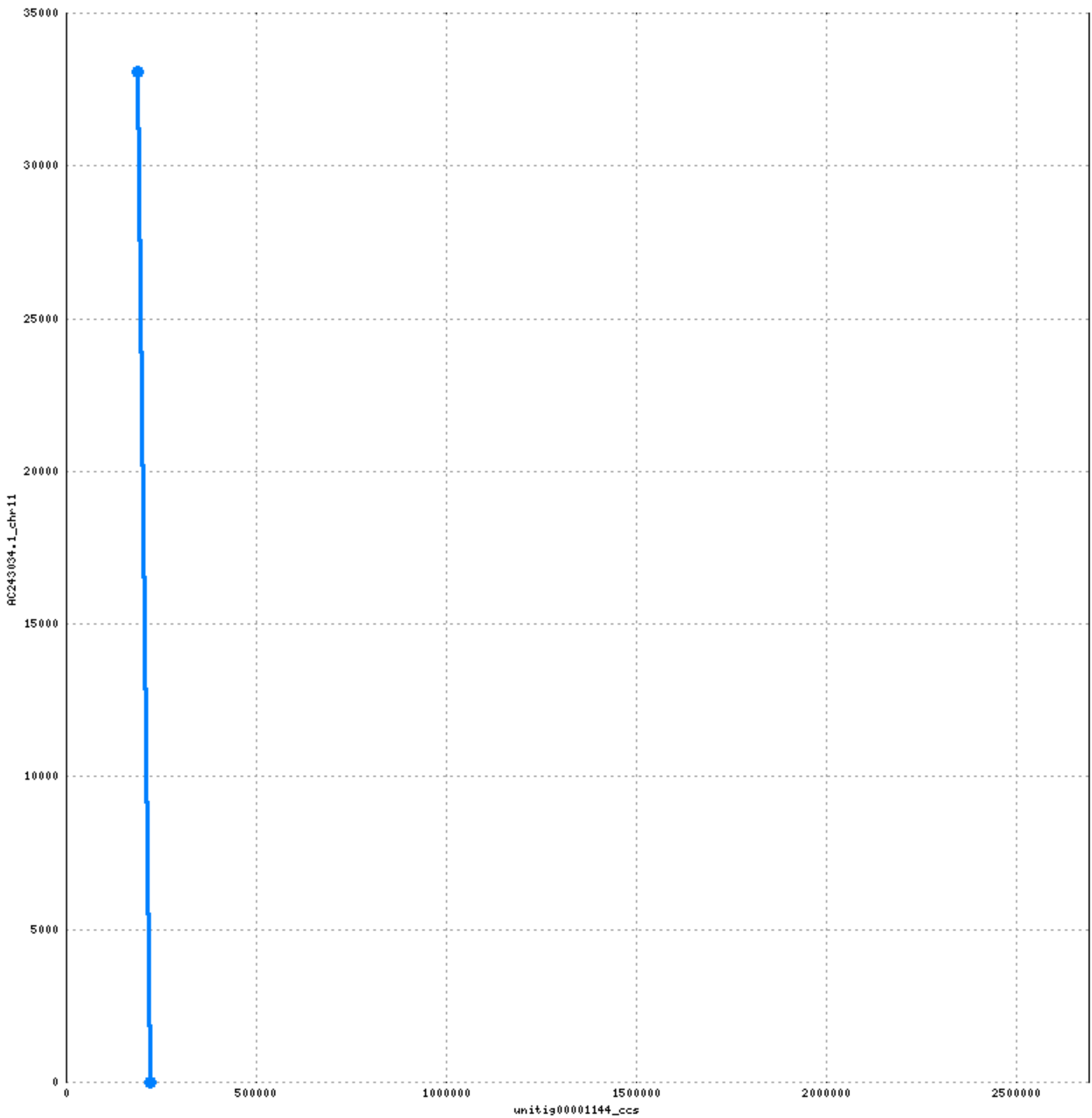

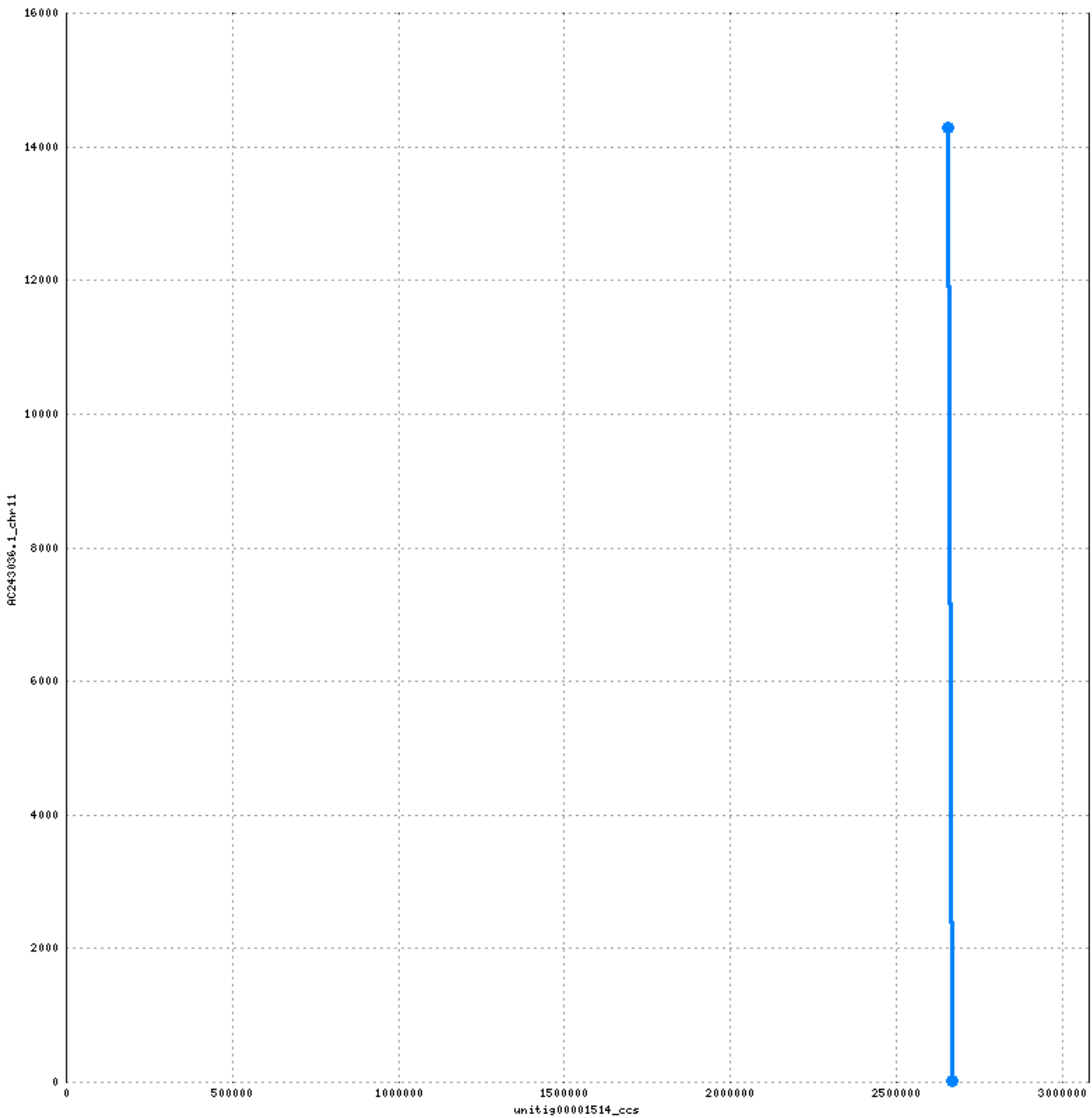

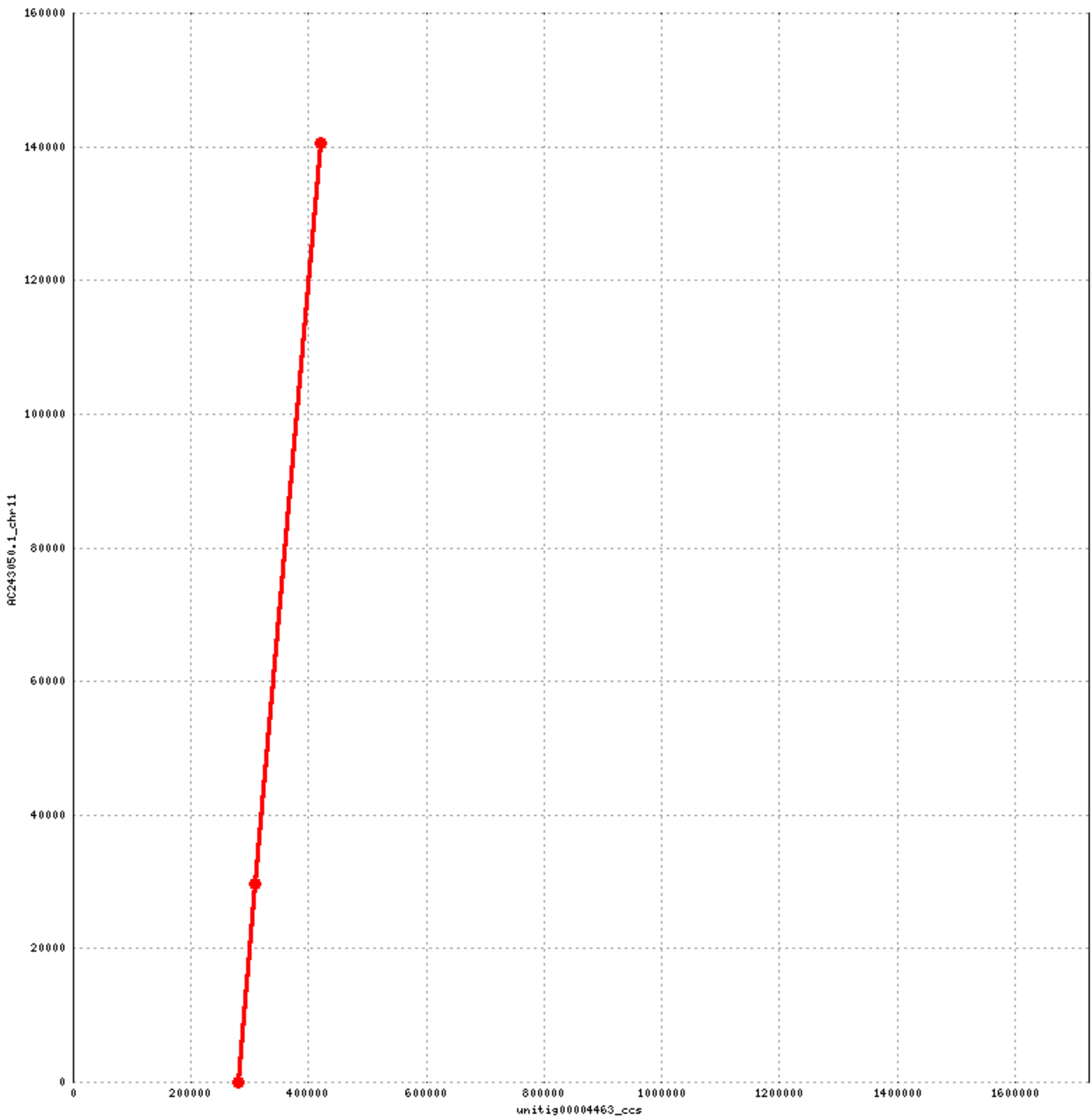

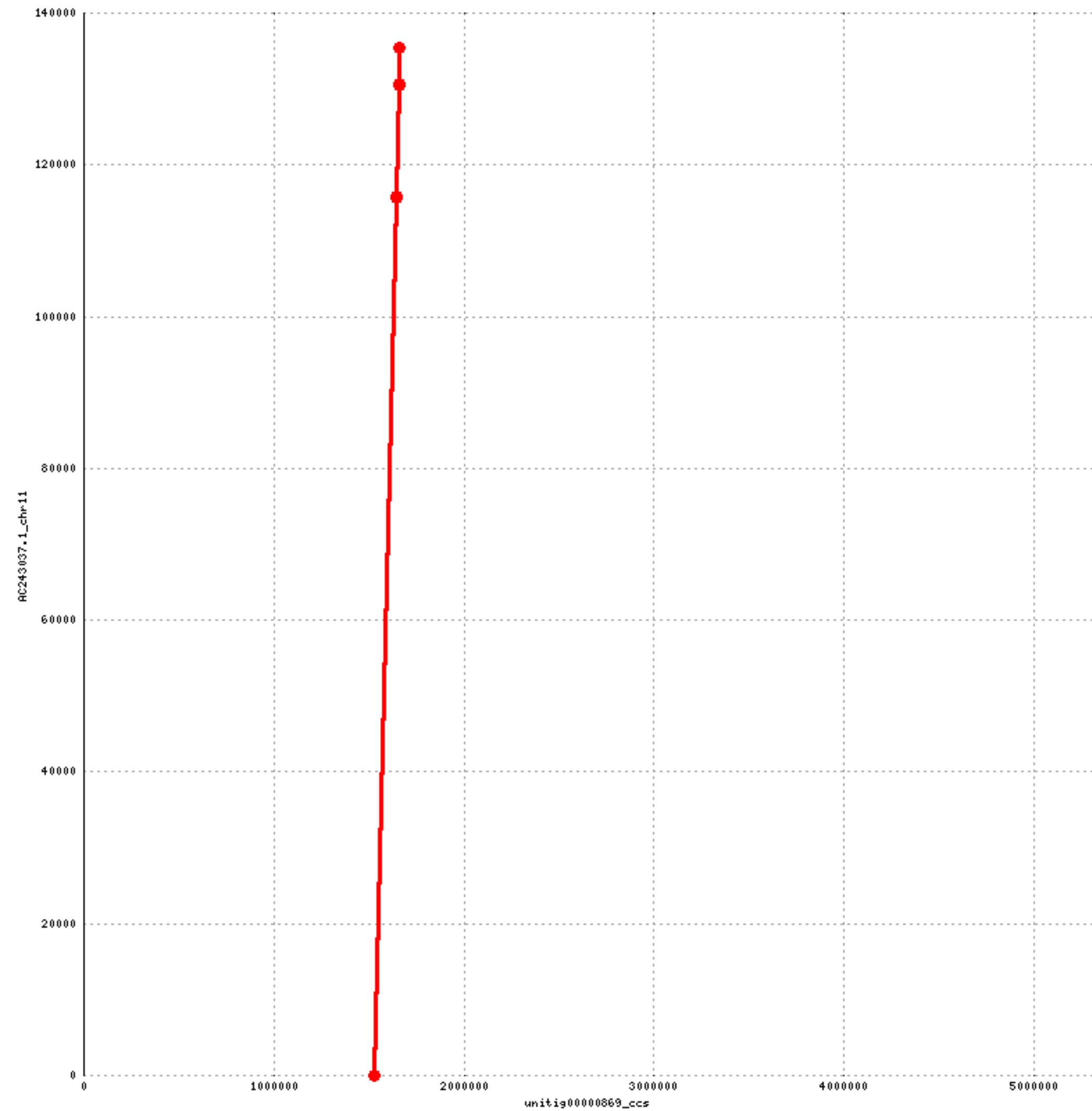

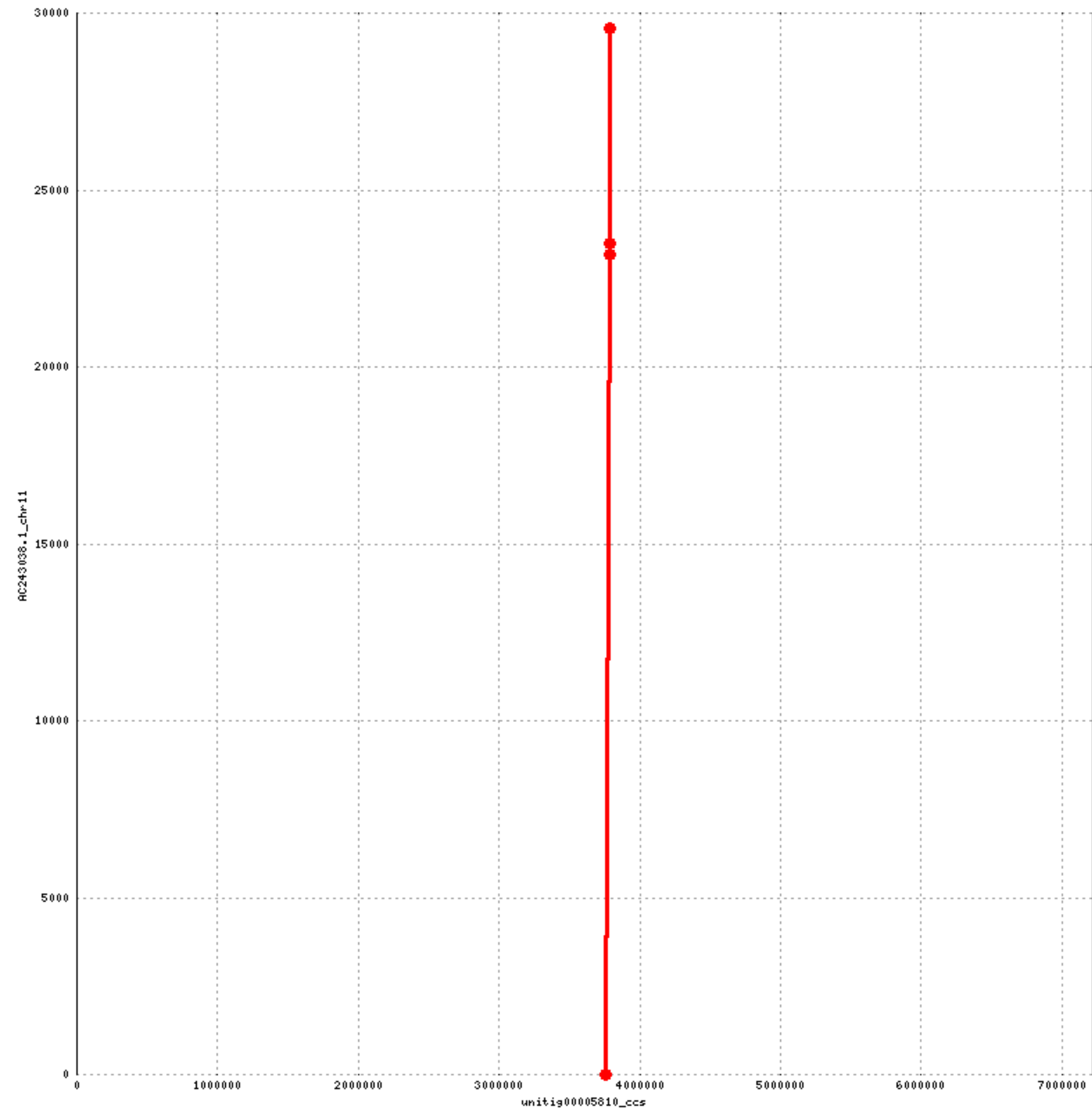

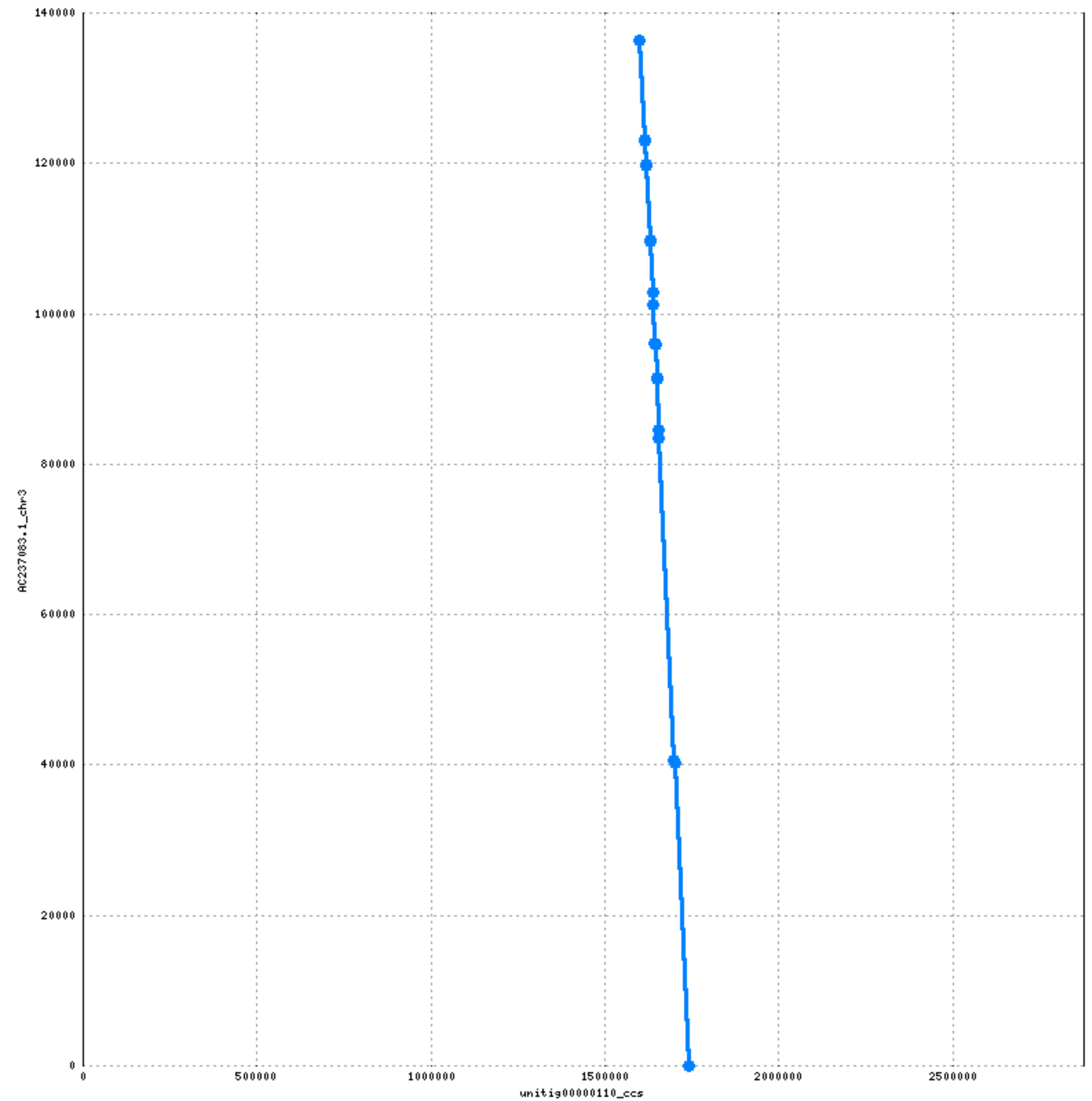

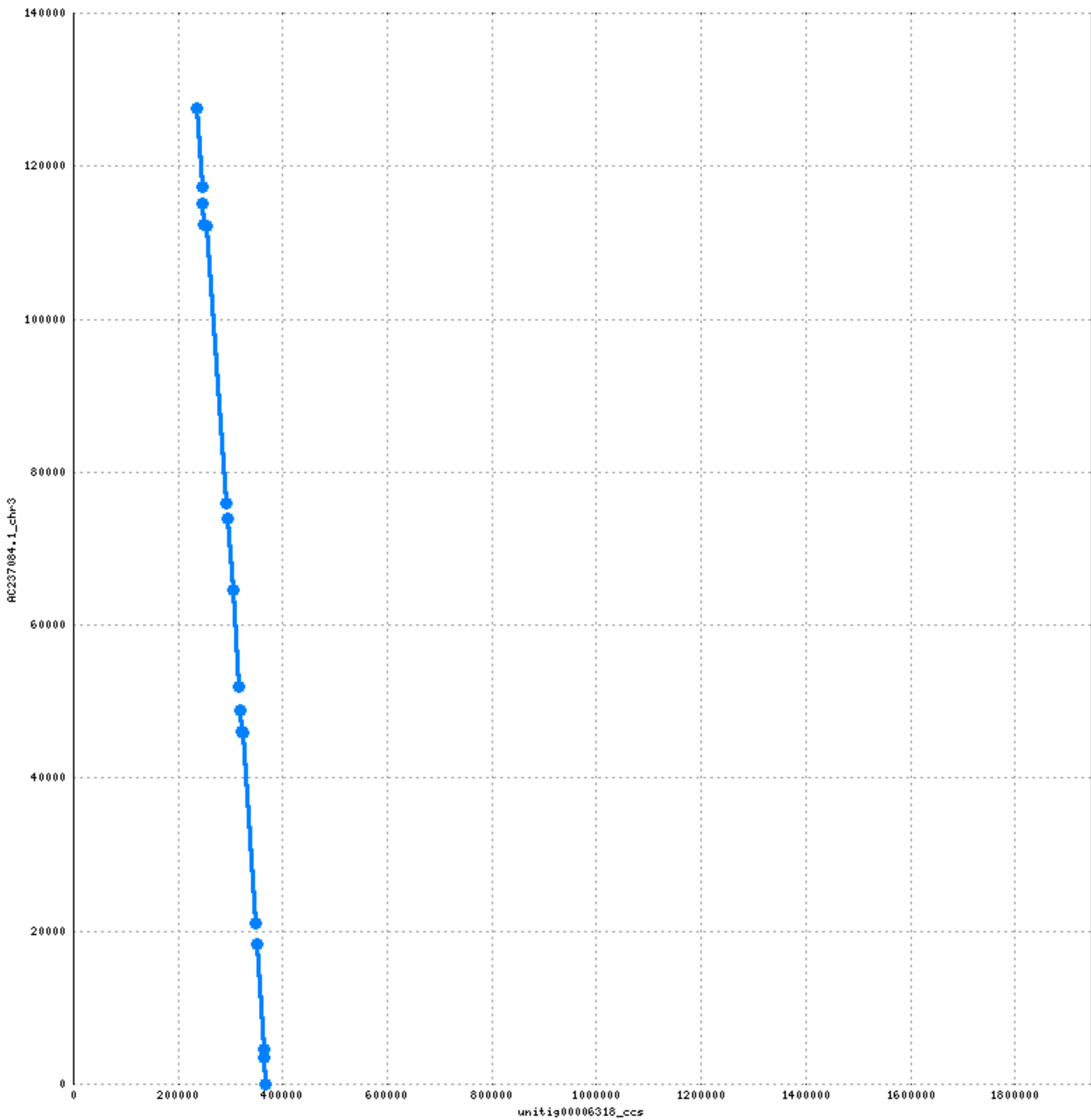

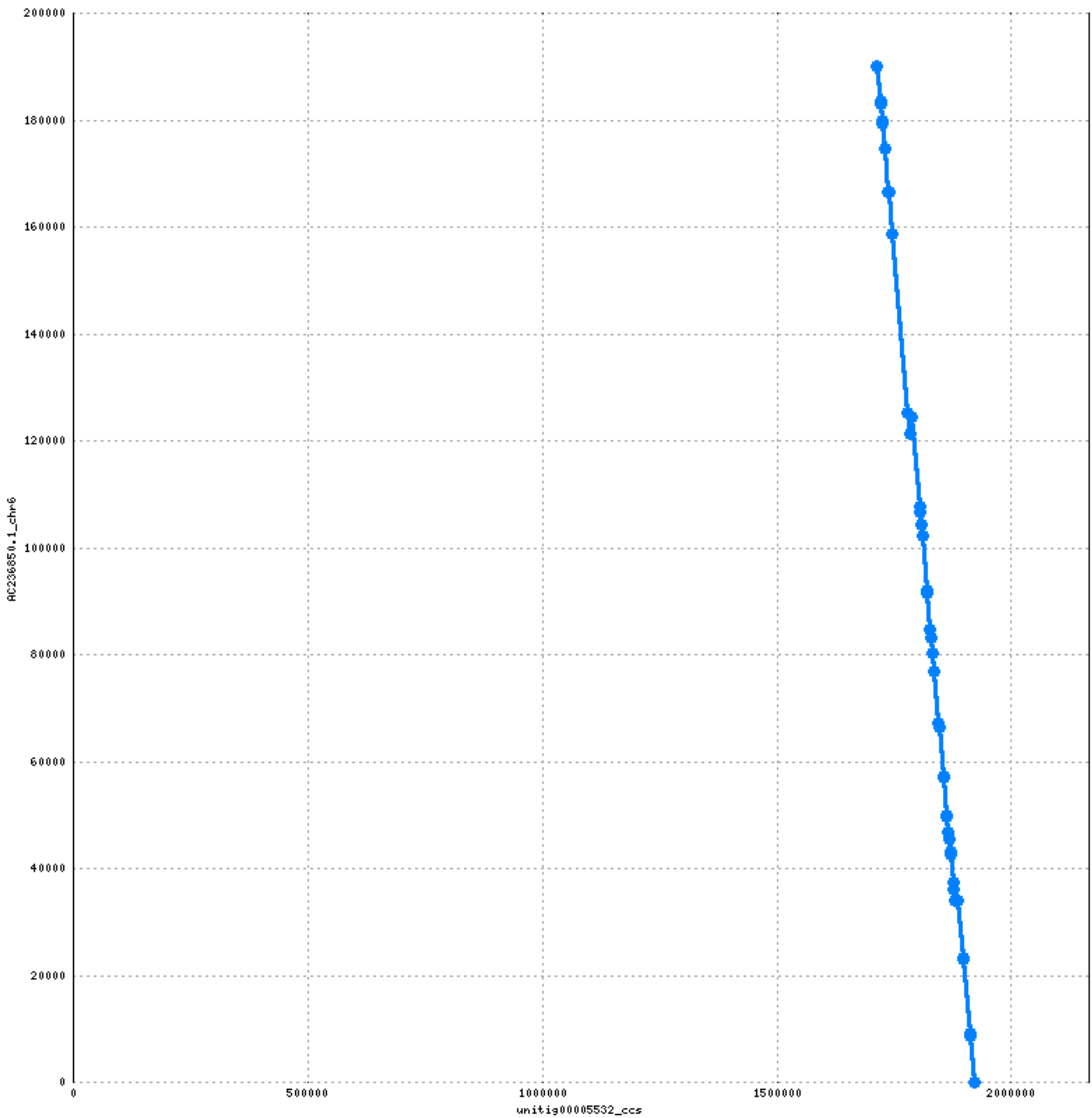

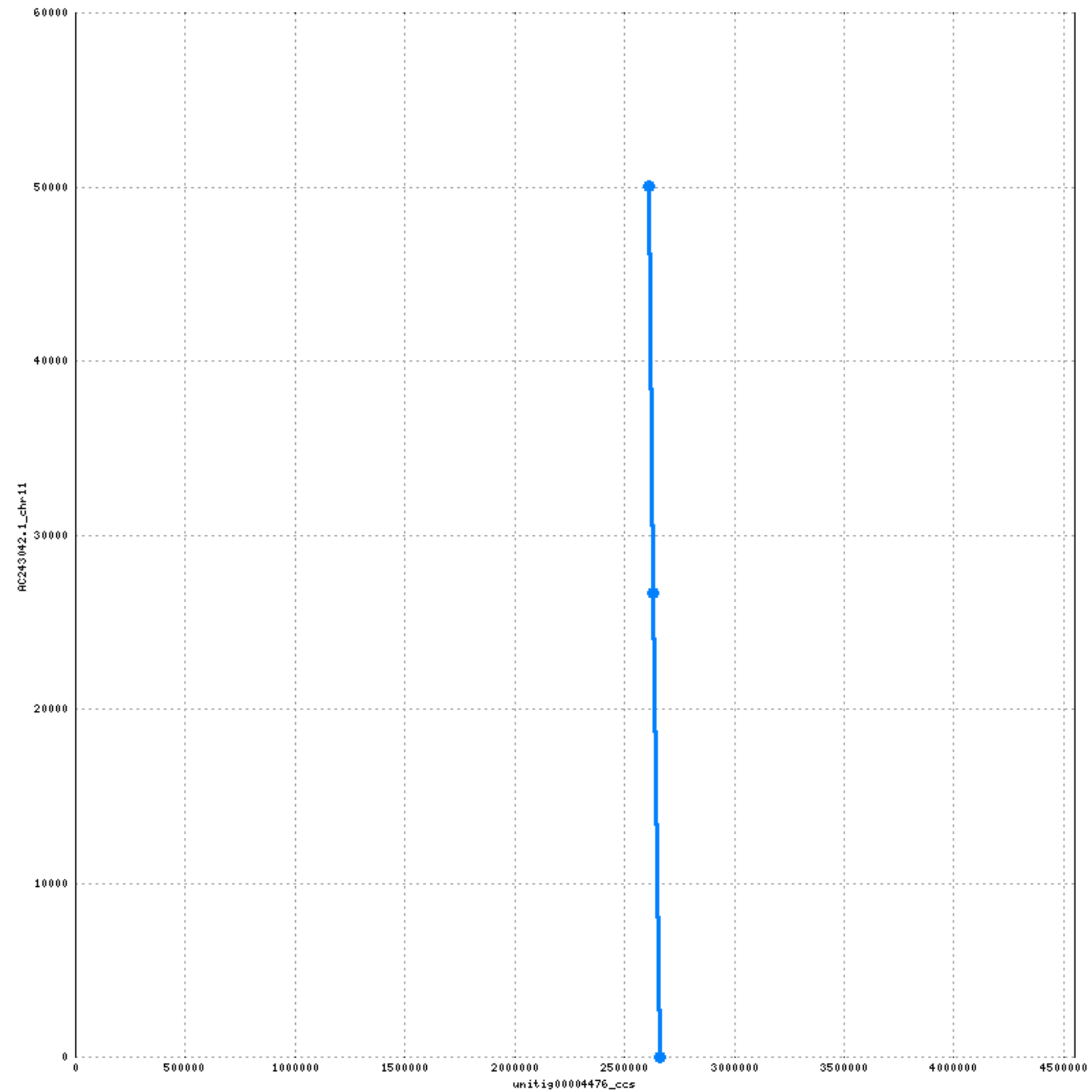

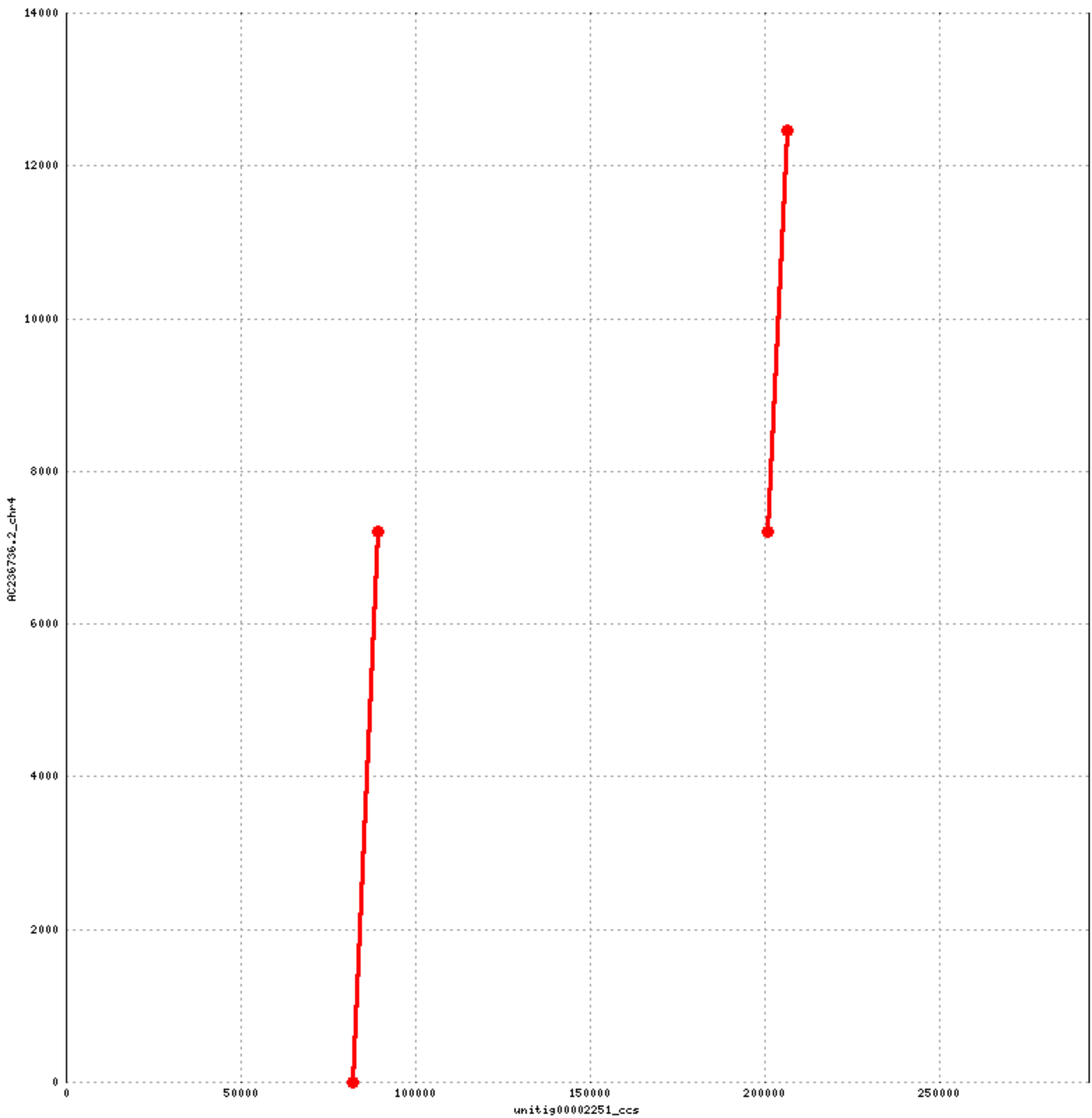

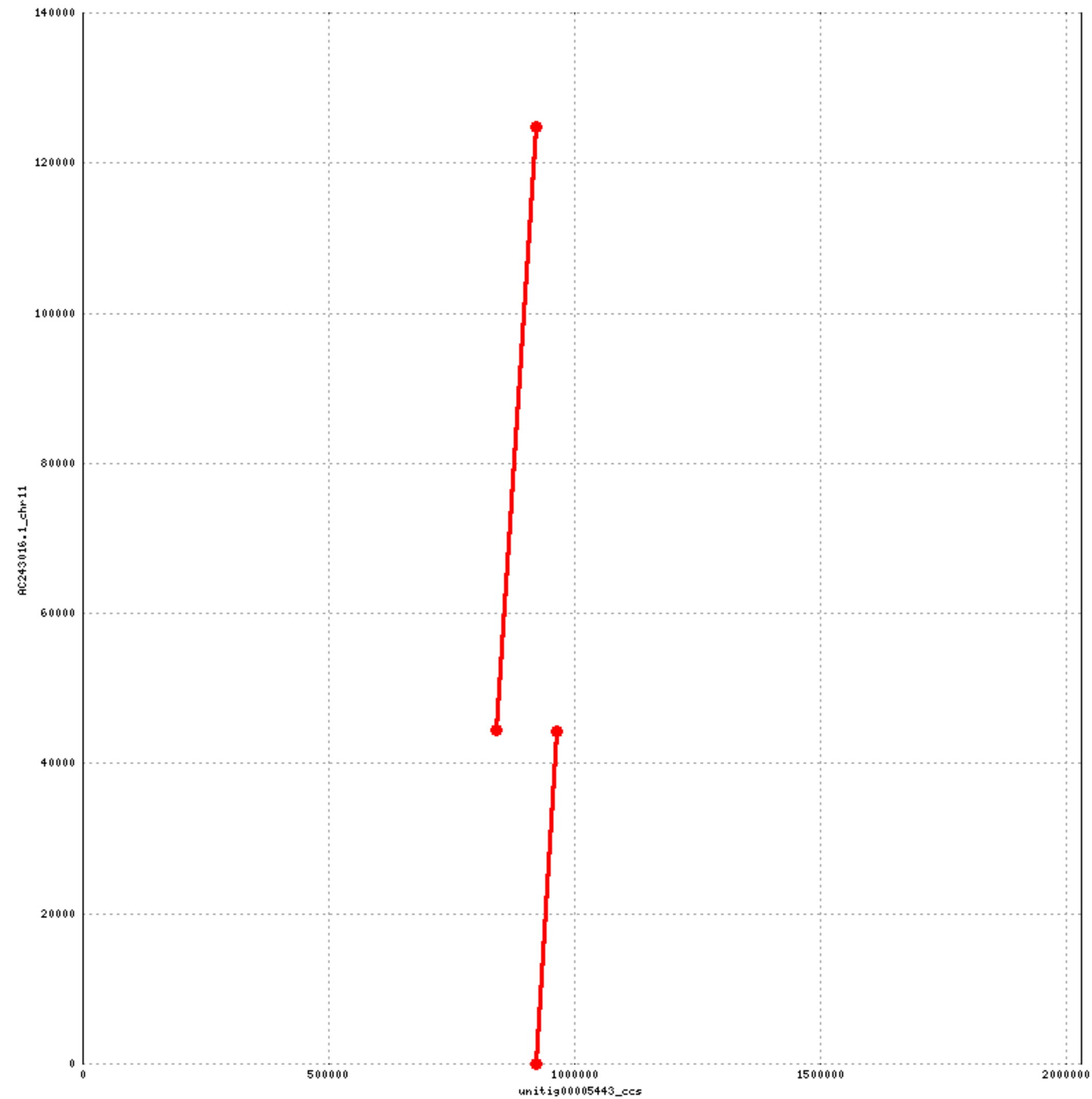

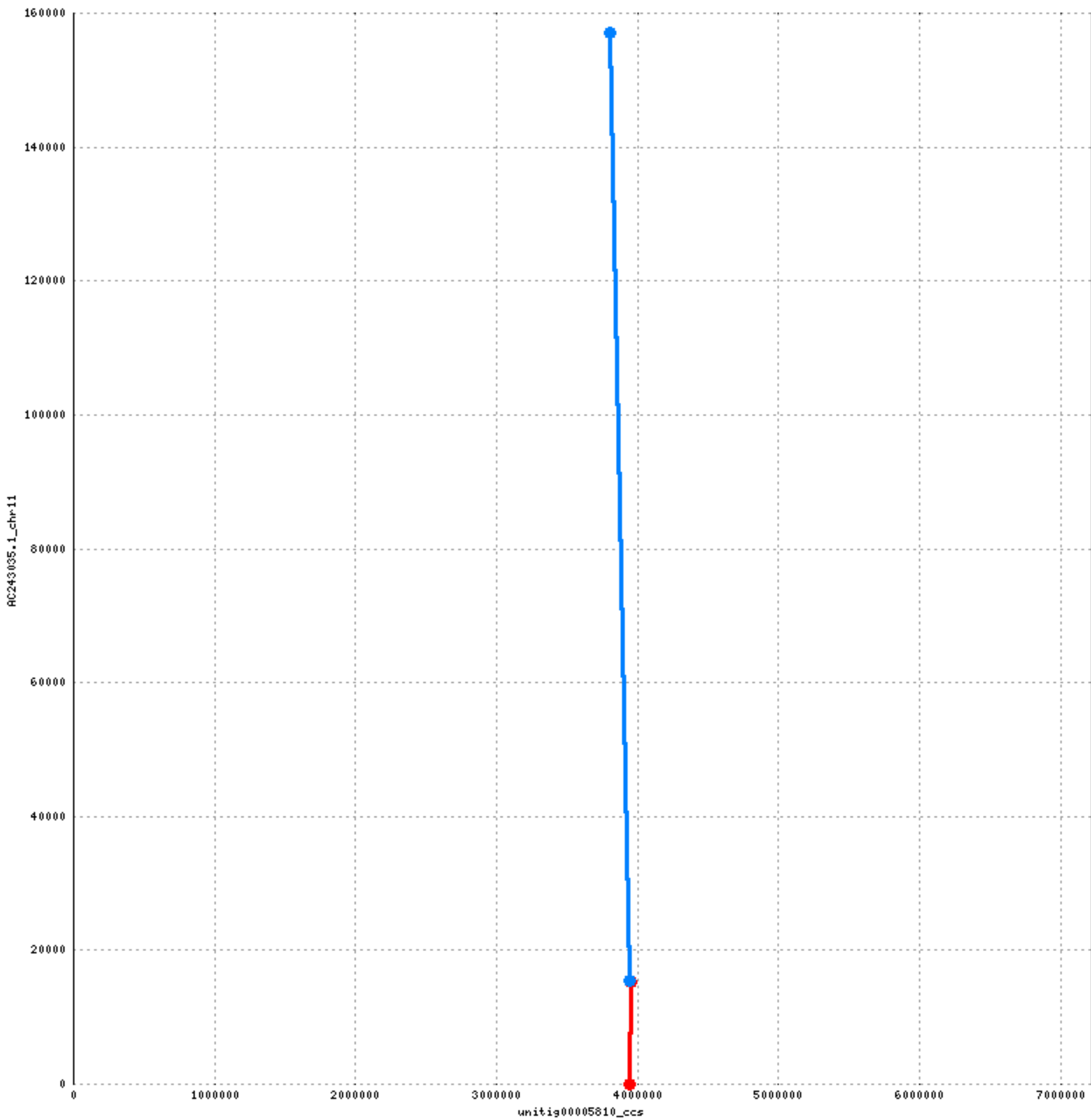

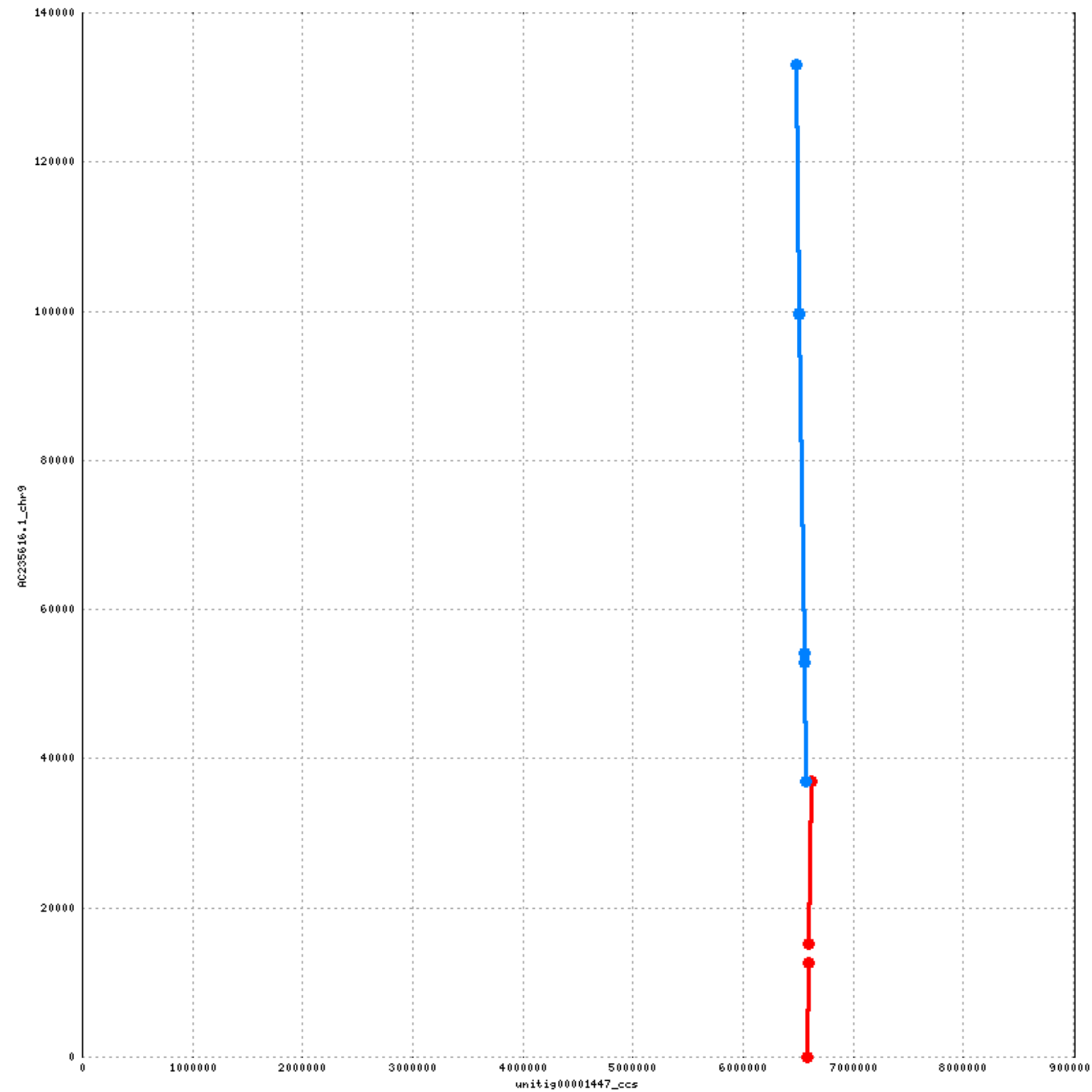

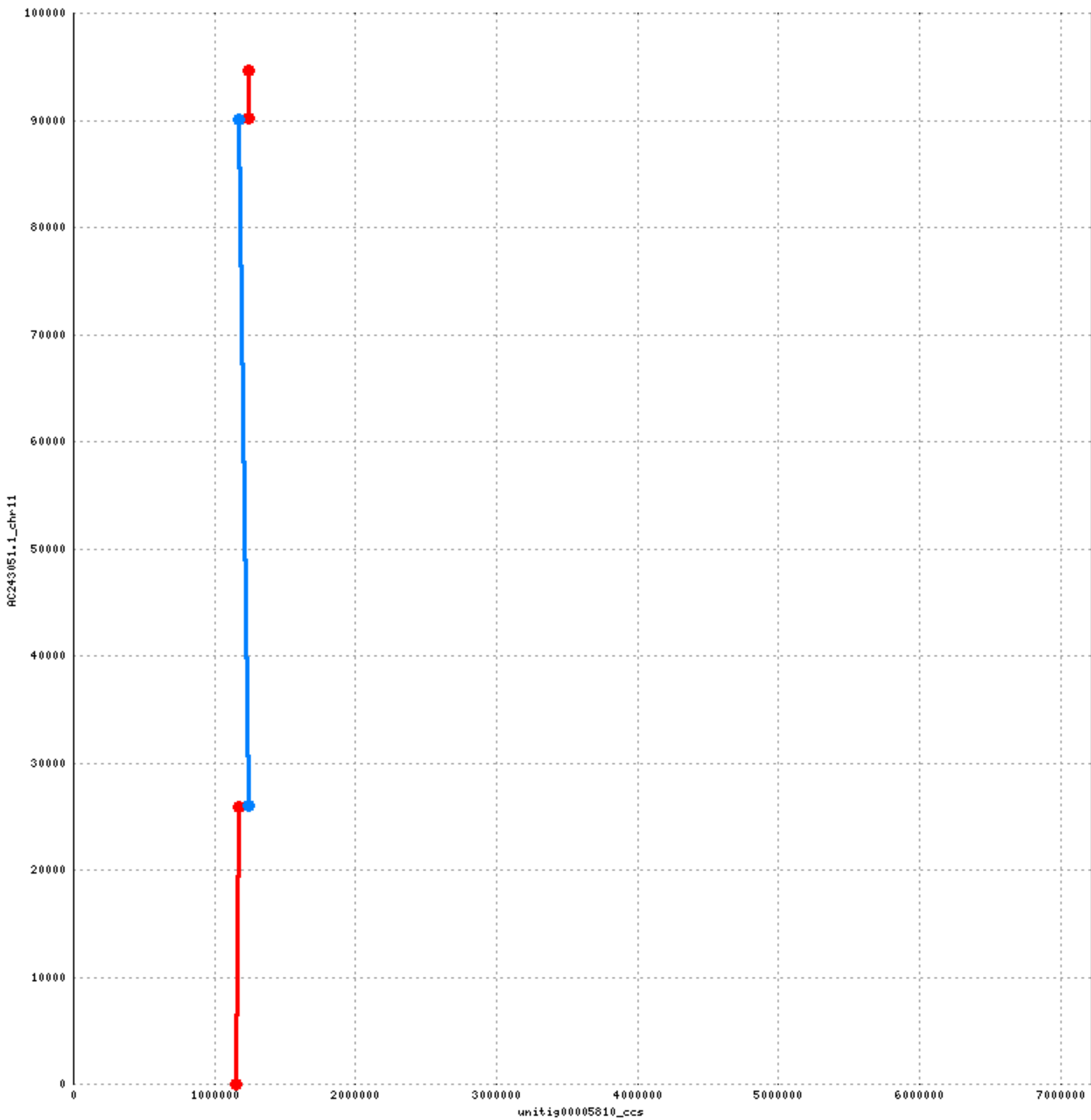

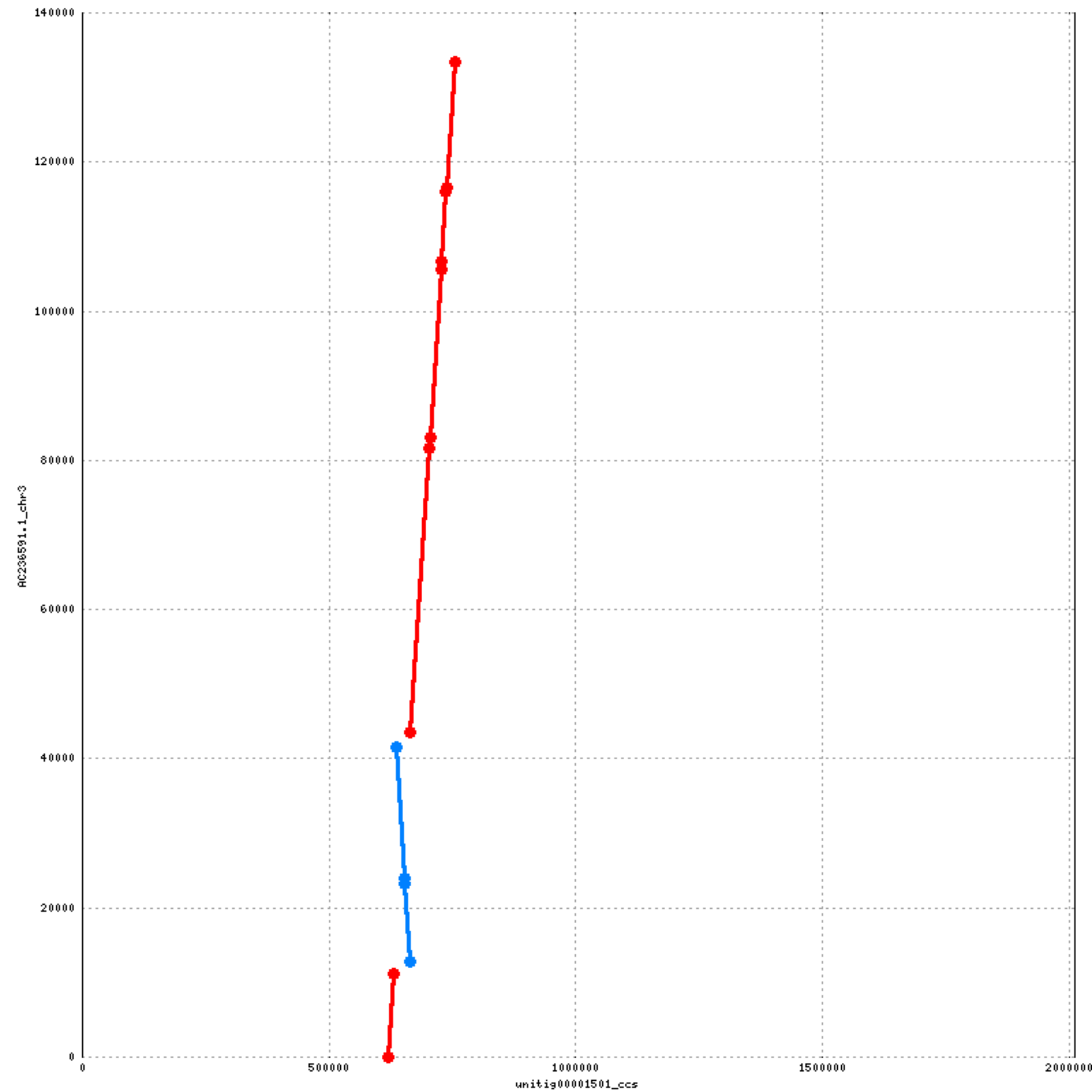

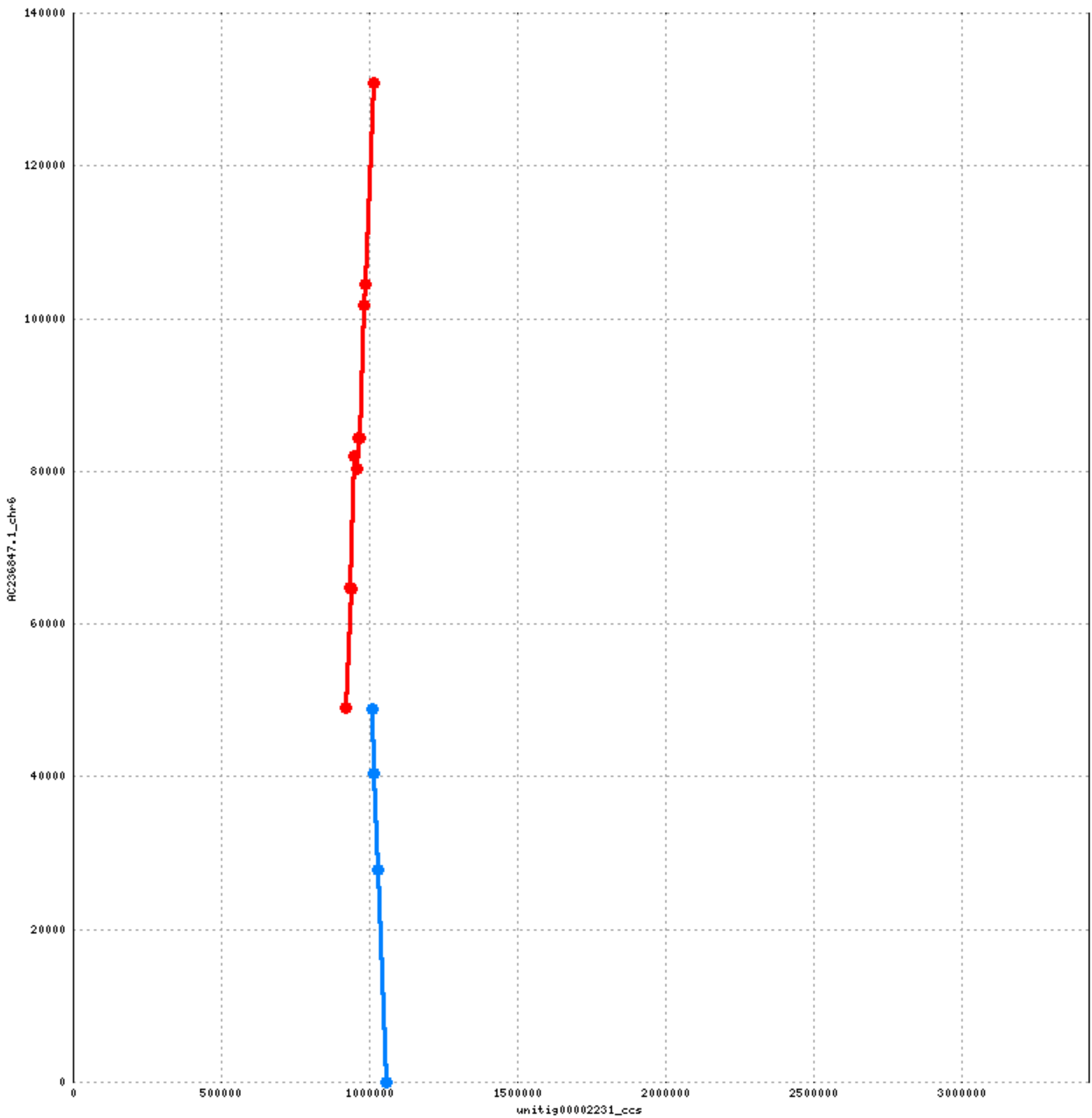

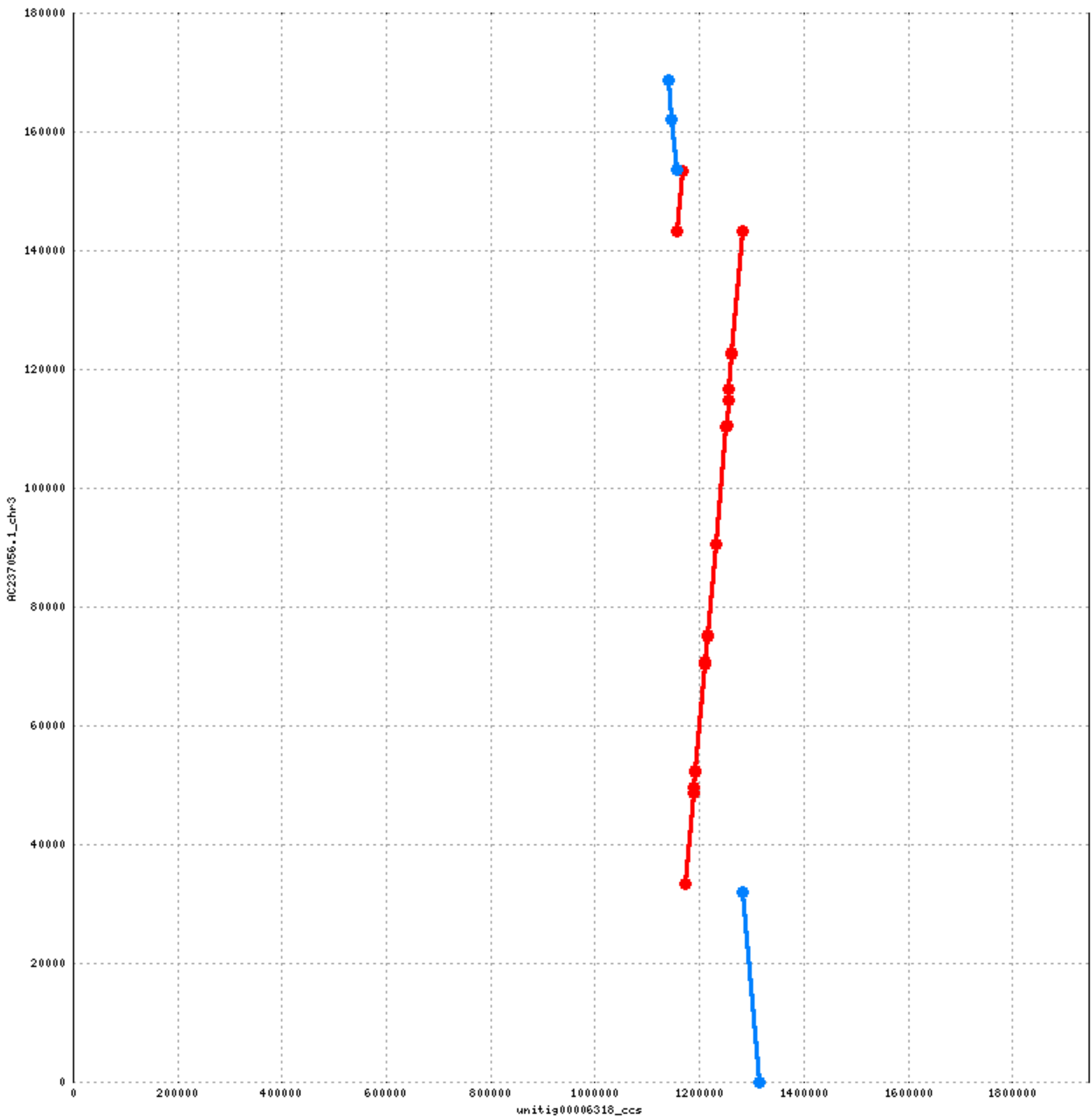

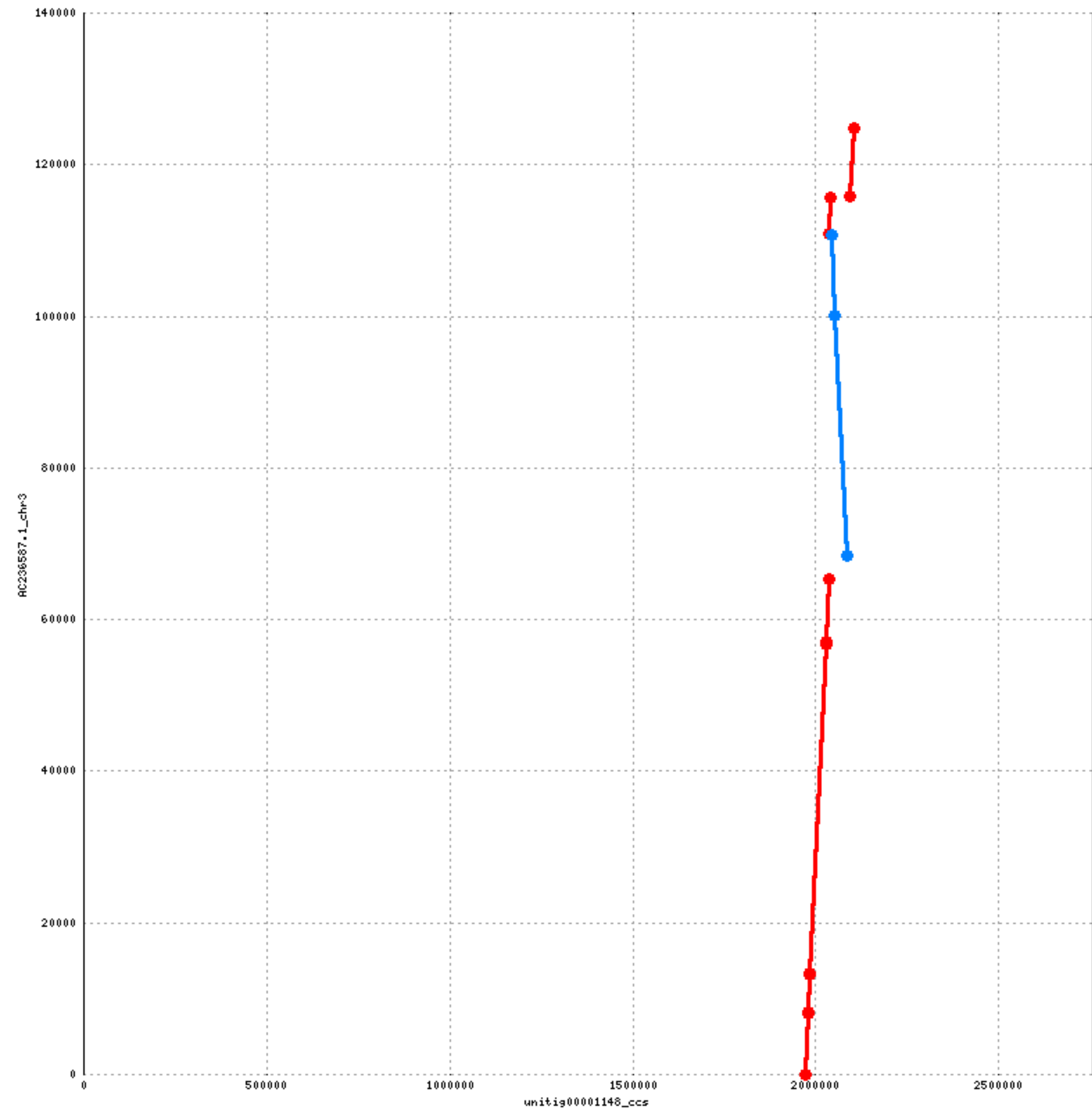

Questionable BAC

RC243046.1\_chr11

unitig00005510\_ccs

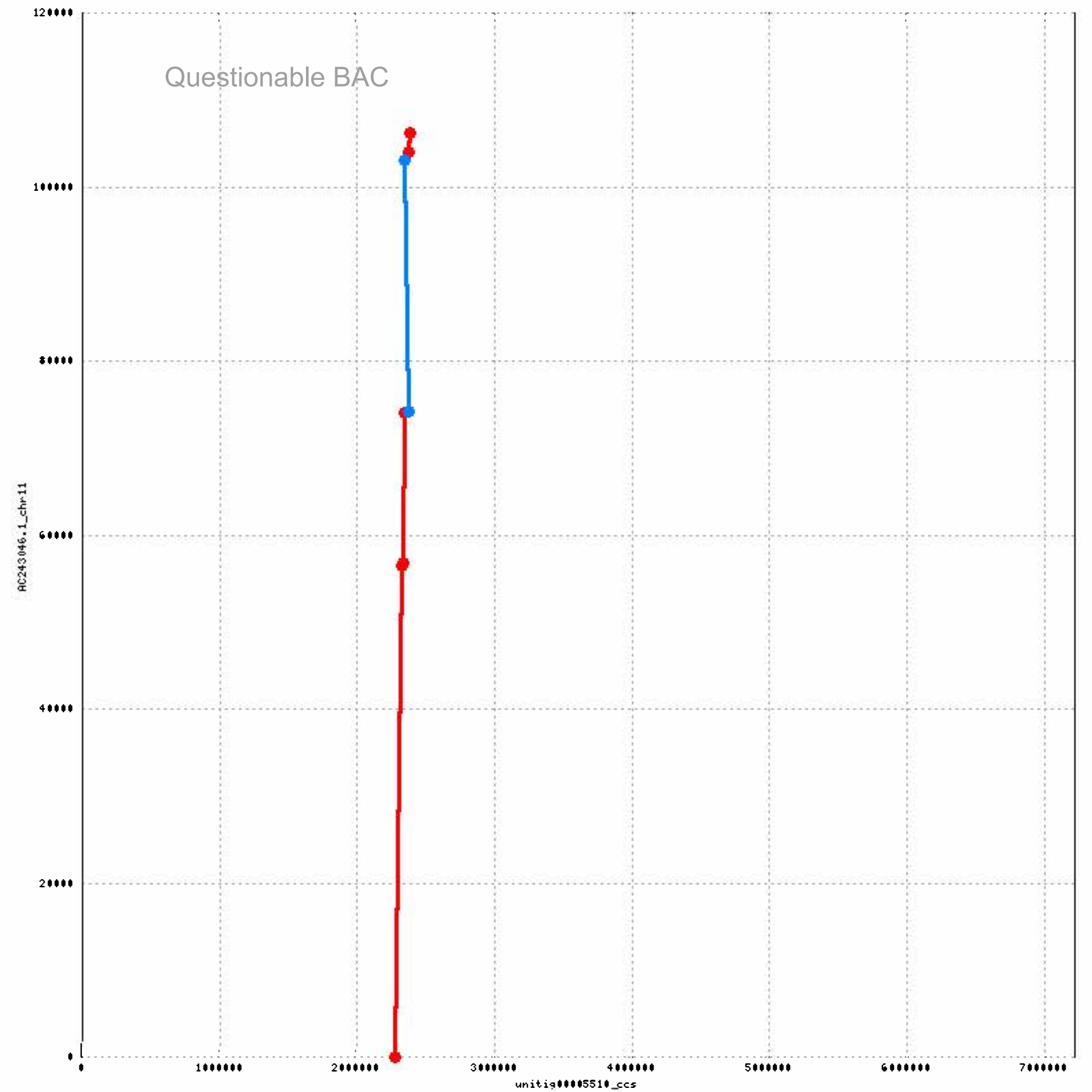

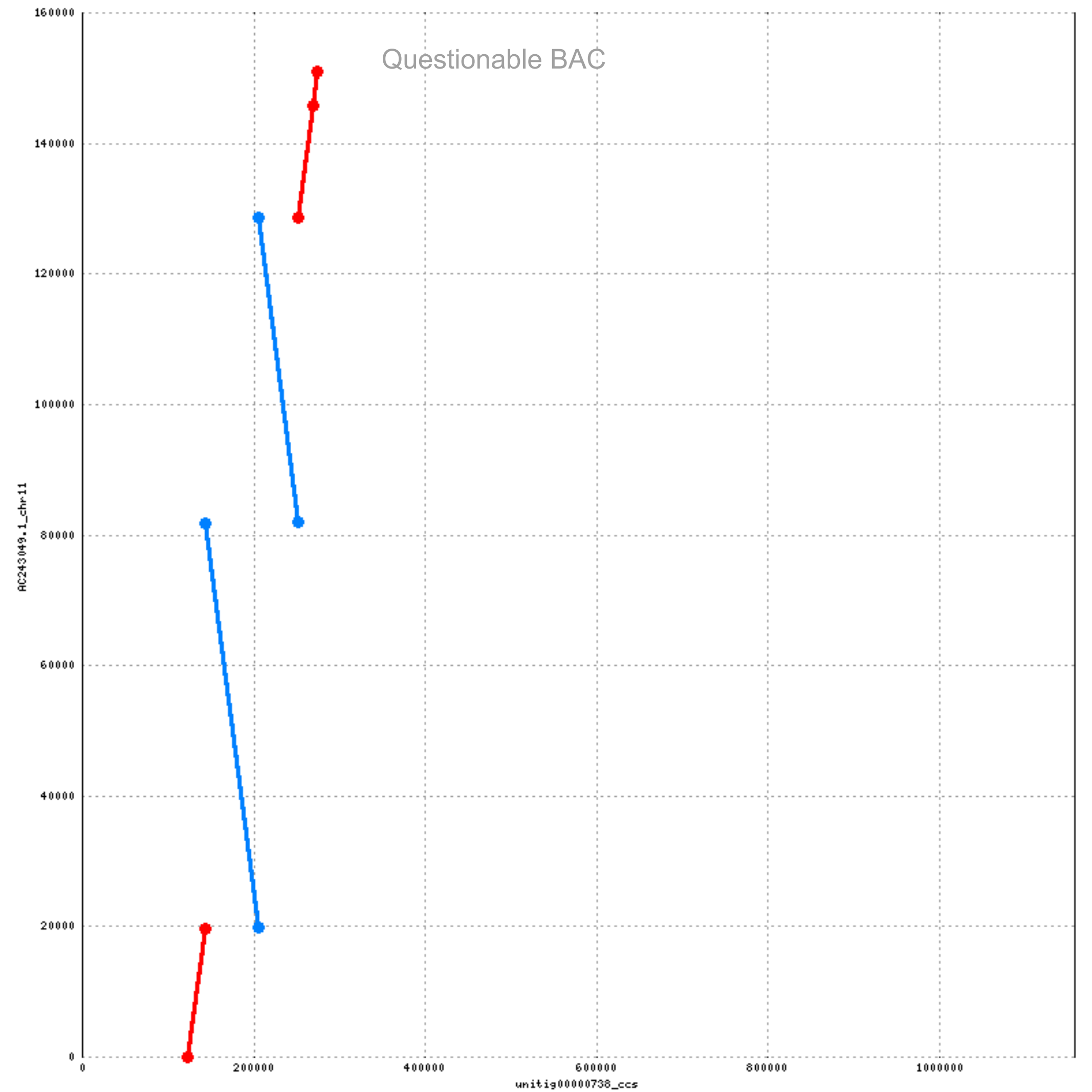

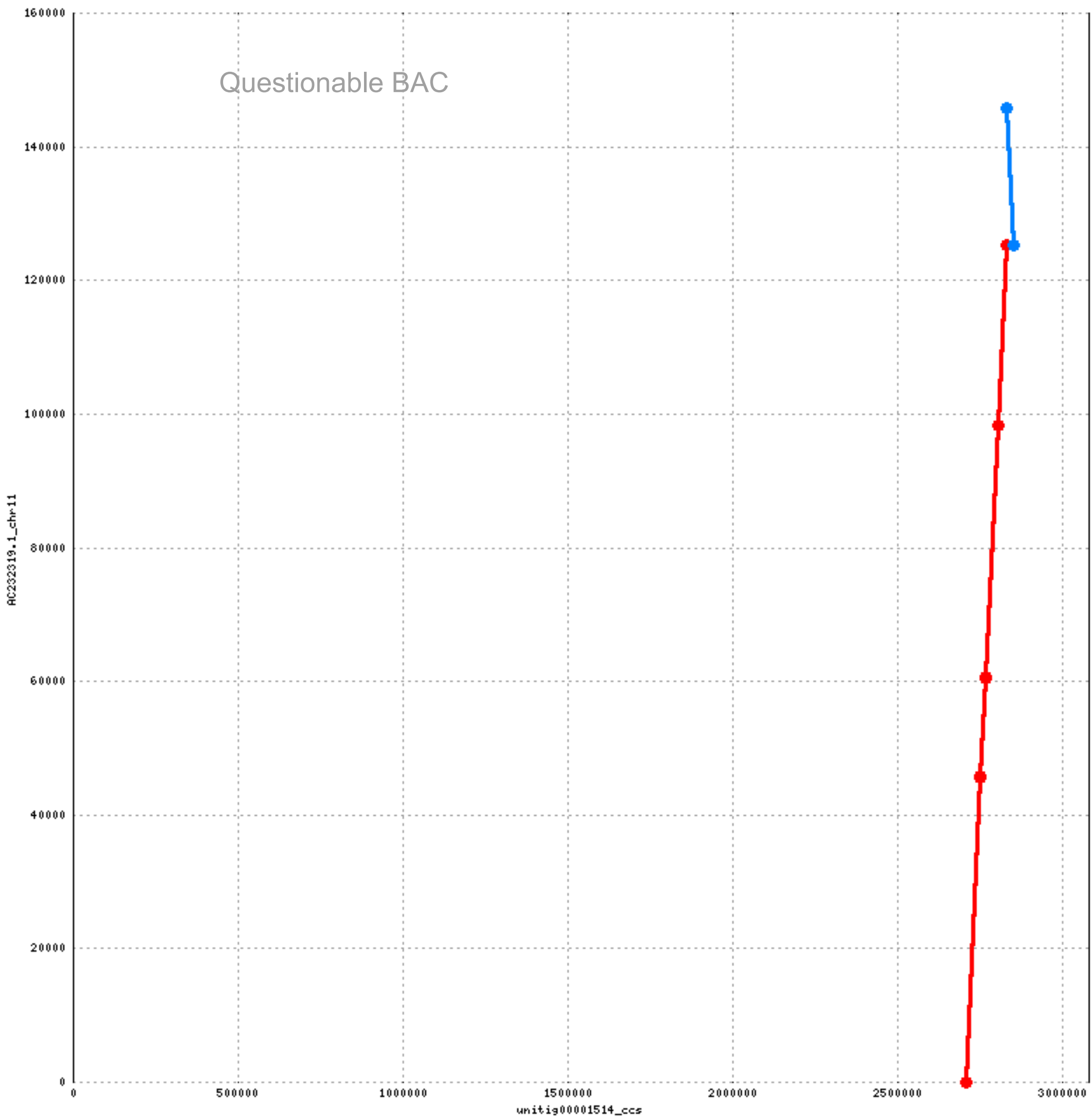

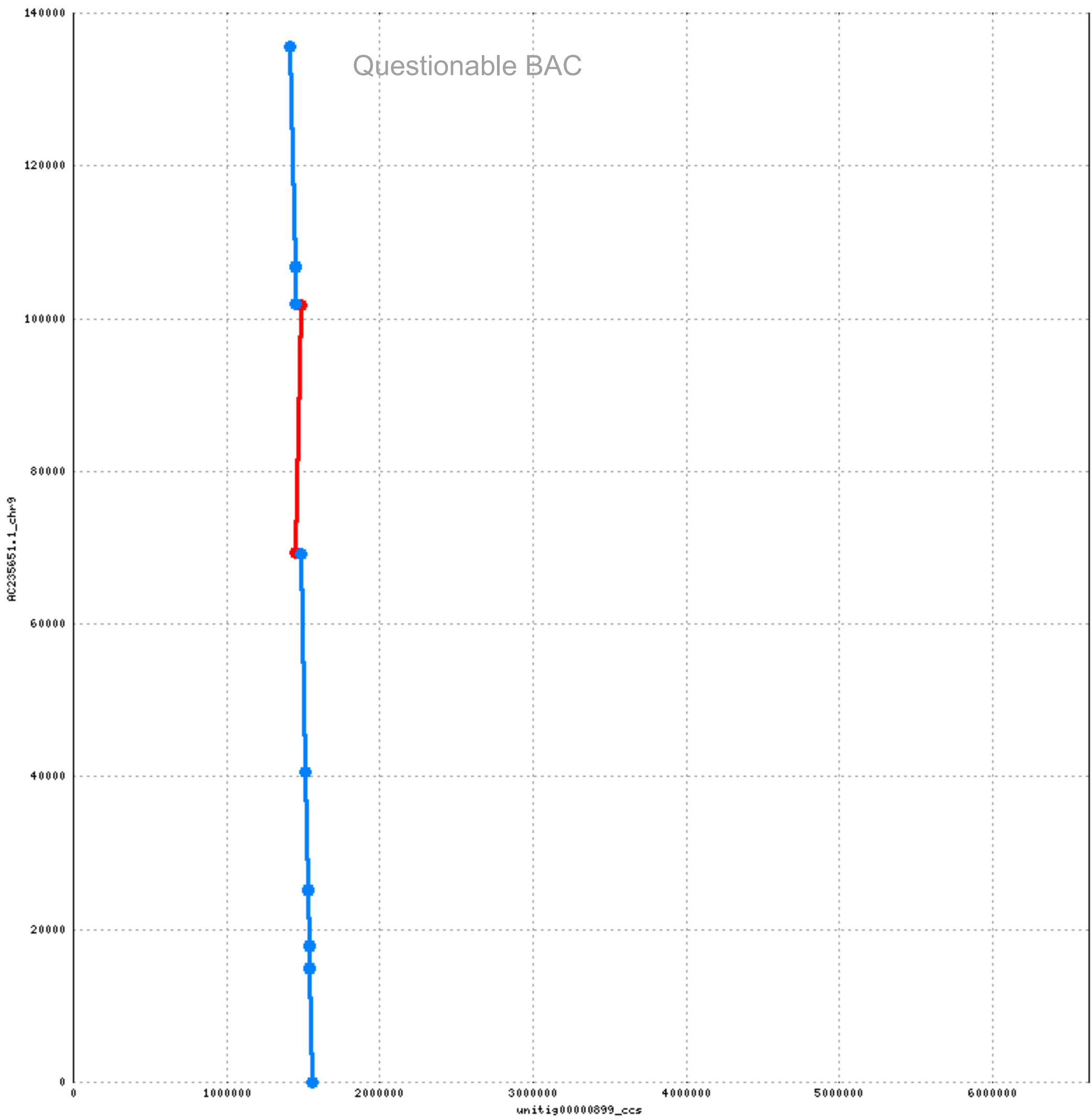

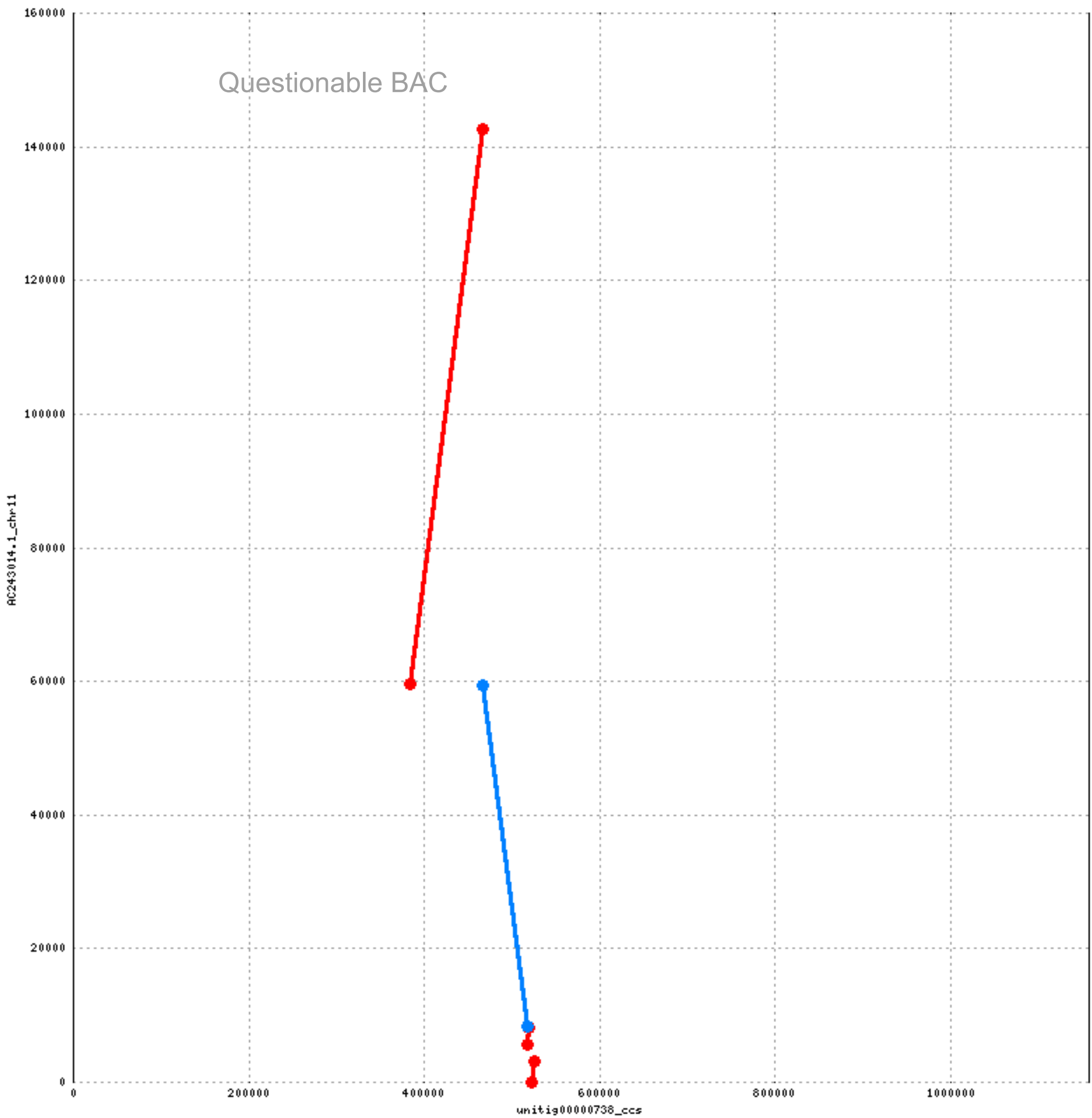

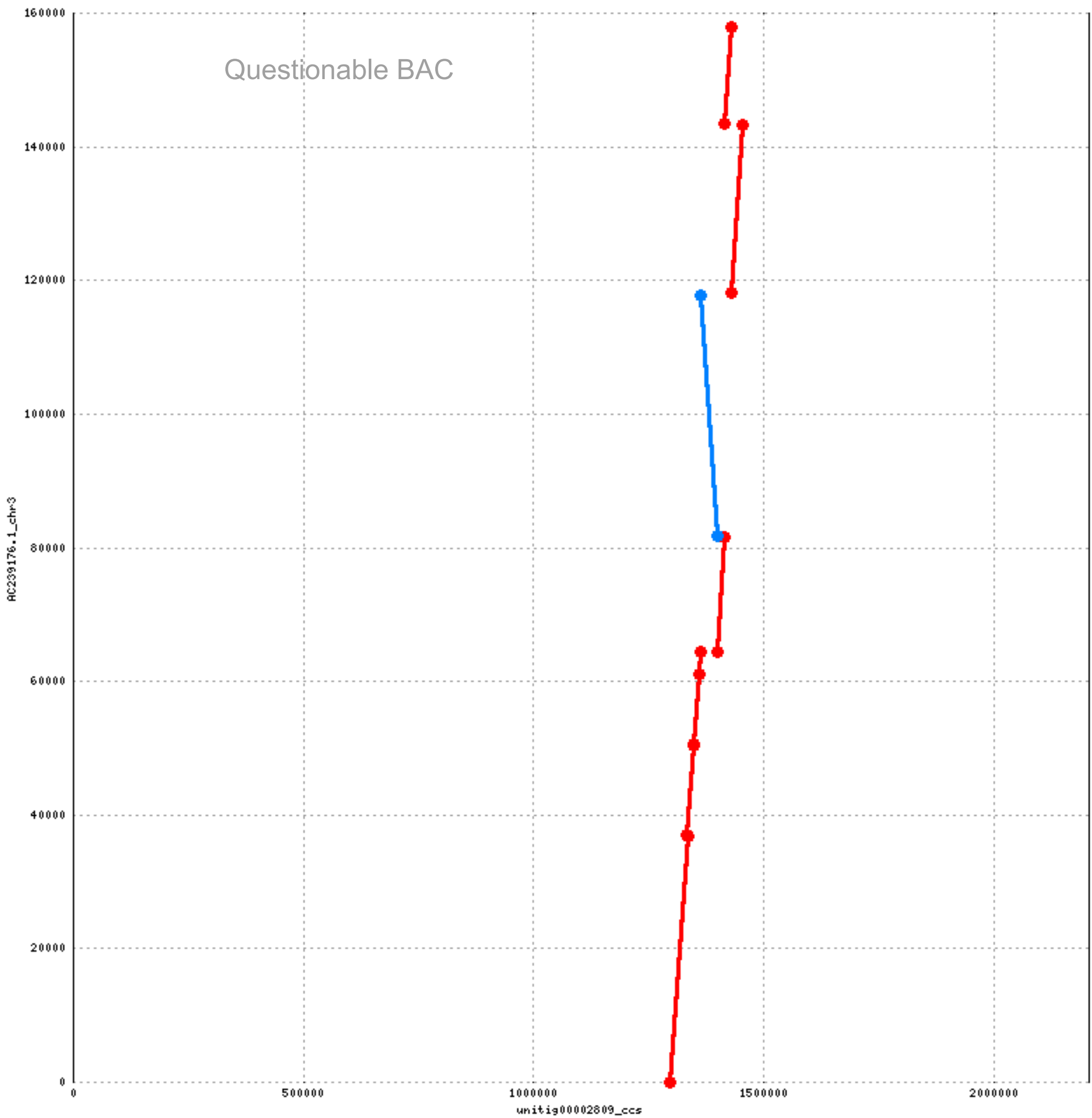

Questionable BAC

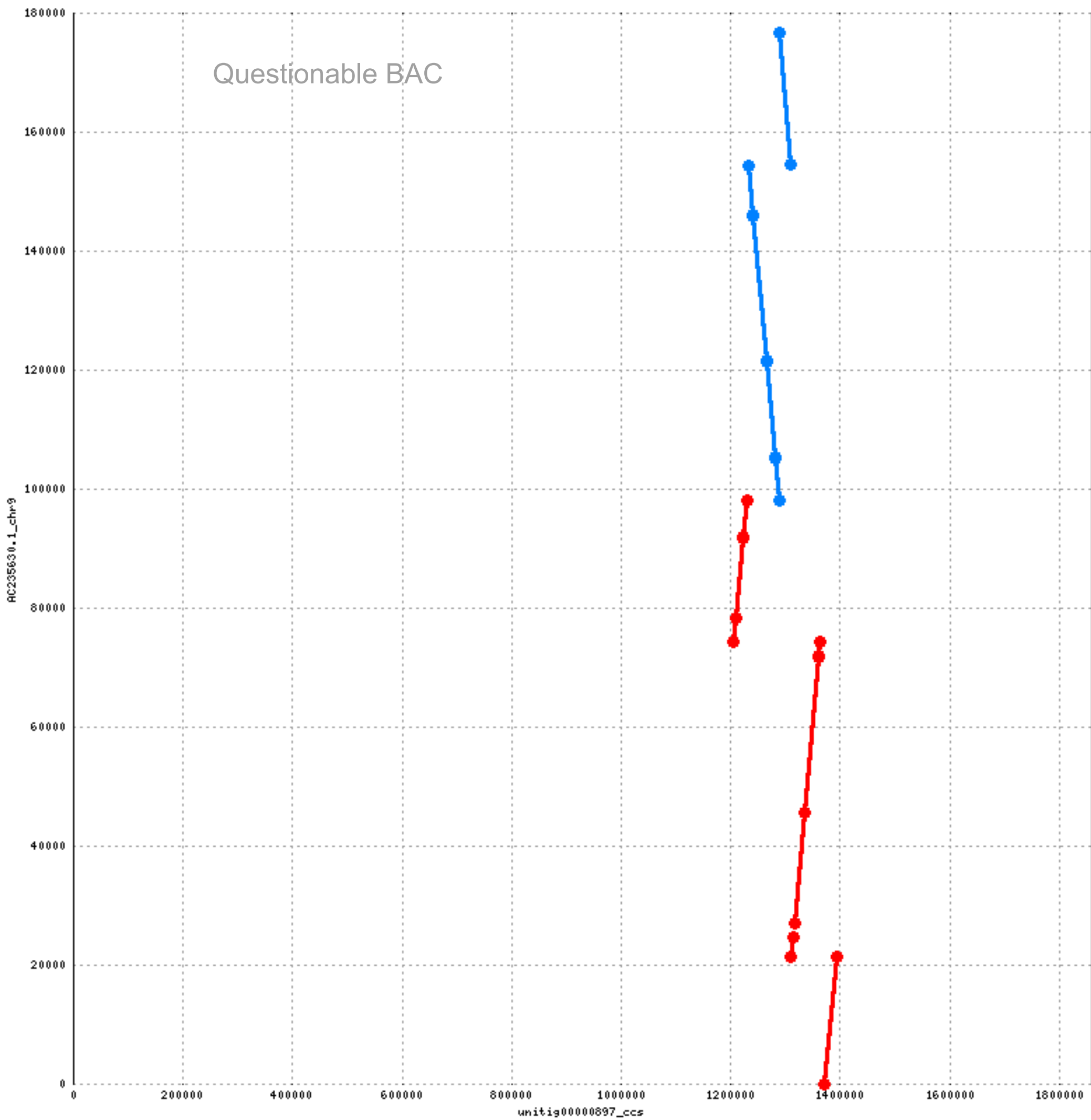

Questionable BAC

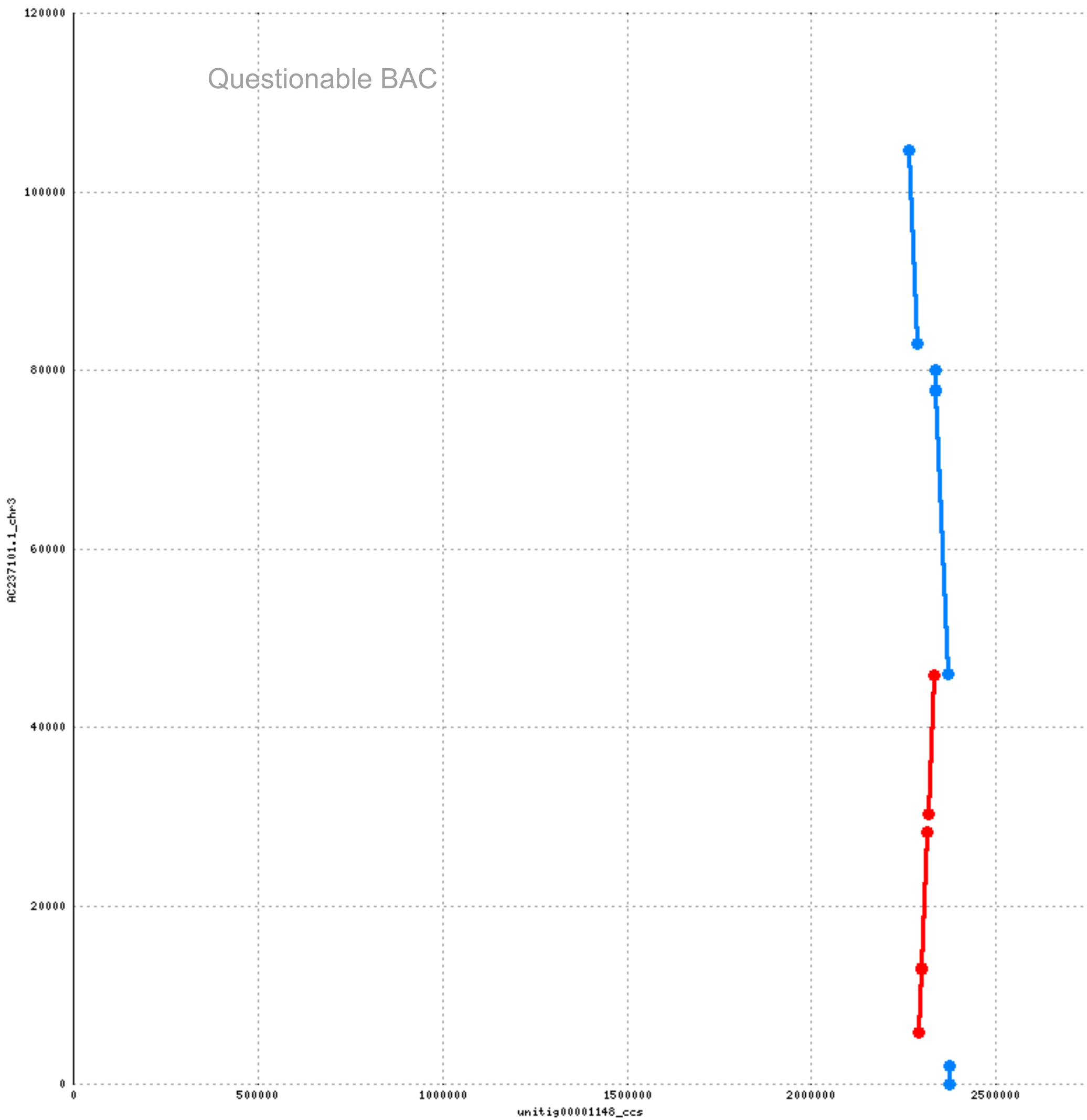

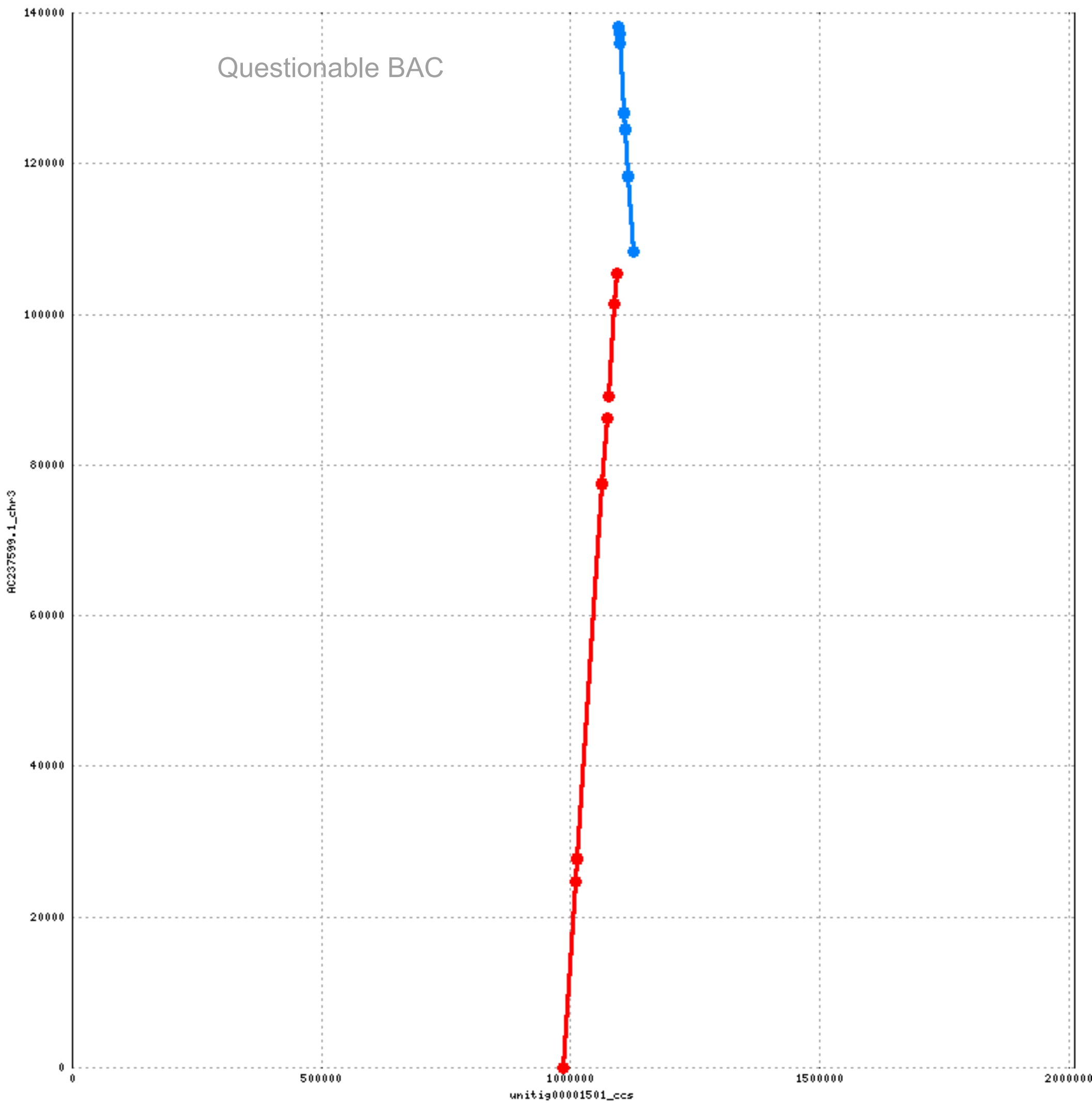

Questionable BAC

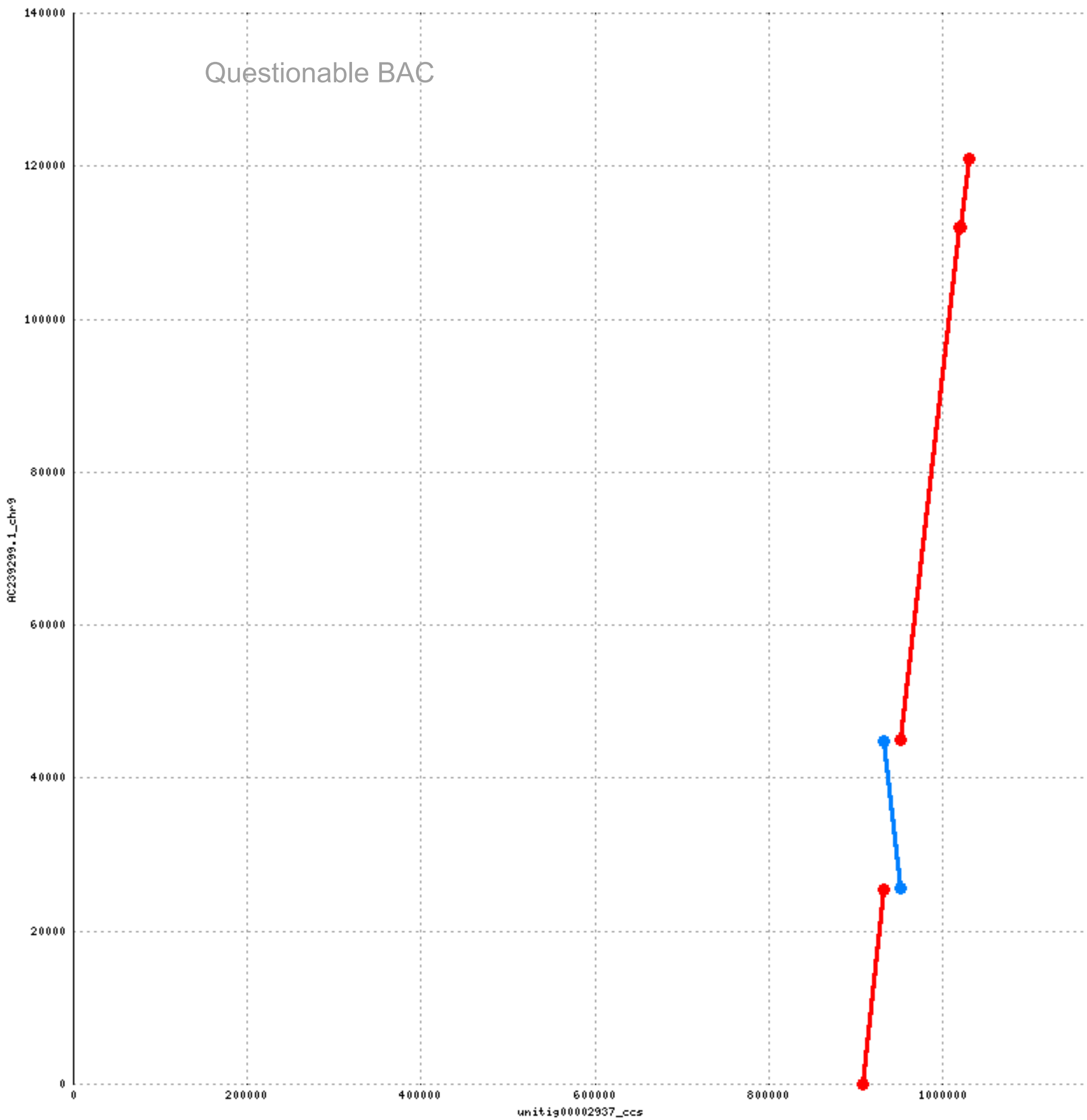

Supplement: Supplementary file 4 — Graphs showing the alignment of BACs (y axis) and RHgv2 unitigs (x axis). [file 41588_2020_699_MOESM4_ESM.pdf]
